# Dynamic clade transitions and the influence of vaccination on the spatiotemporal circulation of SARS-CoV-2 variants

**DOI:** 10.1038/s41541-024-00933-w

**Published:** 2024-08-10

**Authors:** Cecília Artico Banho, Beatriz de Carvalho Marques, Lívia Sacchetto, Ana Karoline Sepedro Lima, Maisa Carla Pereira Parra, Alex Ranieri Jeronimo Lima, Gabriela Ribeiro, Antonio Jorge Martins, Claudia Renata dos Santos Barros, Maria Carolina Elias, Sandra Coccuzzo Sampaio, Svetoslav Nanev Slavov, Evandra Strazza Rodrigues, Elaine Vieira Santos, Dimas Tadeu Covas, Simone Kashima, Ricardo Augusto Brassaloti, Bruna Petry, Luan Gaspar Clemente, Luiz Lehmann Coutinho, Patricia Akemi Assato, Felipe Allan da Silva da Costa, Rejane Maria Tommasini Grotto, Mirele Daiana Poleti, Jessika Cristina Chagas Lesbon, Elisangela Chicaroni Mattos, Heidge Fukumasu, Marta Giovanetti, Luiz Carlos Junior Alcantara, Jayme A. Souza-Neto, Paula Rahal, João Pessoa Araújo, Fernando Rosado Spilki, Benjamin M. Althouse, Nikos Vasilakis, Maurício Lacerda Nogueira

**Affiliations:** 1 Laboratório de Pesquisas em Virologia, Faculdade de Medicina de São José do Rio Preto; São José do Rio Preto, São Paulo, Brazil; 2 Center for Viral Surveillance and Serological Assessment (CeVIVAS), Butantan Institute, São Paulo, Brazil; 3 University of São Paulo, Ribeirão Preto Medical School, Blood Center of Ribeirão Preto, Ribeirão Preto, SP Brazil; 4 University of São Paulo, Centro de Genômica Funcional da ESALQ, Piracicaba, SP Brazil; 5 São Paulo State University (UNESP), School of Agricultural Sciences, Department of Bioprocesses and Biotechnology, Botucatu, Brazil; 6 São Paulo State University (UNESP), School of Agricultural Sciences, Botucatu, Brazil; 7 Molecular Biology Laboratory, Applied Biotechnology Laboratory, Clinical Hospital of the Botucatu Medical School, Botucatu, Brazil; 8 Department of Veterinary Medicine, School of Animal Science and Food Engineering, University of São Paulo, Pirassununga, São Paulo Brazil; 9 Oswaldo Cruz Foundation, FIOCRUZ, Rio de Janeiro, Brazil; 10 Climate Amplified Diseases And Epidemics (CLIMADE), Rio de Janeiro, Brazil; 11 Sciences and Technologies for Sustainable Development and One Health, Universita Campus Bio-Medico di Roma, Selcetta, Italy; 12 Department of Diagnostic Medicine/Pathobiology, College of Veterinary Medicine, Kansas StateUniversity, Manhattan, KS USA; 13 Laboratório de Estudos Genômicos, Departamento de Biologia, Instituto de Biociências Letras e Ciências Exatas (IBILCE), Universidade Estadual Paulista (Unesp), São José do Rio Preto, Brazil; 14 Instituto de Biotecnologia, Universidade Estadual Paulista (Unesp), Botucatu, Brazil; 15 Laboratório de Microbiologia Molecular, Instituto de Ciências da Saúde, Universidade Feevale, Novo Hamburgo, Brazil; 16 Department of Biology, New Mexico State University, Las Cruces, NM USA; 17 Information School, University of Washington, Seattle, WA USA; 18 Department of Pathology, University of Texas Medical Branch, Galveston, TX USA; 19 Center for Vector-Borne and Zoonotic Diseases, University of Texas Medical Branch, Galveston, TX USA; 20 Institute for Human Infection and Immunity, University of Texas Medical Branch, Galveston, TX USA

**Keywords:** Viral infection, SARS-CoV-2

## Abstract

Since 2021, the emergence of variants of concern (VOC) has led Brazil to experience record numbers of in COVID-19 cases and deaths. The expanded spread of the SARS-CoV-2 combined with a low vaccination rate has contributed to the emergence of new mutations that may enhance viral fitness, leading to the persistence of the disease. Due to limitations in the real-time genomic monitoring of new variants in some Brazilian states, we aimed to investigate whether genomic surveillance, coupled with epidemiological data and SARS-CoV-2 variants spatiotemporal spread in a smaller region, can reflect the pandemic progression at a national level. Our findings revealed three SARS-CoV-2 variant replacements from 2021 to early 2022, corresponding to the introduction and increase in the frequency of Gamma, Delta, and Omicron variants, as indicated by peaks of the Effective Reproductive Number (Reff). These distinct clade replacements triggered two waves of COVID-19 cases, influenced by the increasing vaccine uptake over time. Our results indicated that the effectiveness of vaccination in preventing new cases during the Delta and Omicron circulations was six and eleven times higher, respectively, than during the period when Gamma was predominant, and it was highly efficient in reducing the number of deaths. Furthermore, we demonstrated that genomic monitoring at a local level can reflect the national trends in the spread and evolution of SARS-CoV-2.

## Introduction

Several SARS-CoV-2 variants have emerged over the course of the COVID-19 pandemic, driven by an unprecedented transmission rate and the rapid evolution of RNA virus genomes^1–3^. Lineages of SARS-CoV-2 harboring mutations associated with increased transmissibility, pathogenesis, and immune evasion began to appear in different countries in mid-2020. These emerging lineages have been classified by the World Health Organization (WHO) as variants of concern (VOC): Alpha^4^, Beta^5^, Gamma^6^, Delta^7^, and Omicron^8^.

These VOCs have replaced circulating strains, leading to subsequent waves of COVID-19 infection and healthcare system overloads, and the need of frequent vaccine updates^6,9–11^. The increasing number of cases from these new waves has reinforced the need for continuous genomic surveillance of SARS-CoV-2^12–14^. Studies on the epidemiology and the spatiotemporal history of SARS-CoV-2 transmission can effectively contribute to implementing real-time public health measures to contain the viral spread. While WHO has classified SARS-CoV-2 transmission and its disease as a continuous health problem, a robust genomic surveillance, variant identification, and the evaluation of their pathogenic potential, are fundamental elements for policymaking and disease containment^3,10^.

Brazil was an epicenter of the COVID-19 pandemic, and by June 2024, it had recorded 38,815,115 cases and 712,258 deaths^15^. In order to contain or mitigate the pandemic effets, in January 2021 Brazil started the vaccination campain and as of April 2022 76% of the Brazilian population have received two doses of vaccines against COVID-19^16^. In early 2021, the Brazilian Health Regulatory Agency (ANVISA) approved the use of four vaccines, CoronaVac (Sinovac^TM^/Institute Butantan) and AZD1222 (AstraZeneca/Oxford’s^TM^) in January 2021; followed by BNT162b2 (Pfizer/BioNtech^TM^) and Ad26.COV2.S (Johnson/Janssen^TM^)^16^. The vaccines distribution occurred in phases, and it was first administered on people aged ≥60; institutionalized disabled people; indigenous, and health workers^16^. Since the start of Brazilian vaccination campain, several hindrances were observed, due to the high territorial extension of the country, making difficult to deliver vaccines, contributing a delayed vaccination program, as well as low adherence to vaccination, in some regions, due to political issues^17^. In addition to slow vaccination rates, resources for real-time genomic surveillance were limited in most Brazilian states, which makes identifying the emergence of regional variants or mutations challenging in a country the size of Brazil. One strategy to address this challenge is to monitor the lineages that circulate in a smaller region, such as a midsize city, which can reflect the Brazilian epidemiological landscape. São José do Rio Preto (SJdRP) is a suitable location for such monitoring, as the city presented the third highest number of COVID-19 cases and deaths in the state of São Paulo (SP)^18^. Furthermore, it housed one of the country’s largest and most significant hospital complexes, the Hospital de Base de São José do Rio Preto (HB). This reference center, serving more than two million inhabitants, was at the forefront of COVID-19 care and treatment for the state^19^ and, with its constant exchange of SARS-CoV-2 variants, serves as an important model for visualizing and predicting viral transmission patterns. Here, we investigated how the introduction and spread of different SARS-CoV-2 VOCs combined with vaccination rollout have shaped the progression of COVID-19 in a single health district in northwestern São Paulo state.

## Results

### SARS-CoV-2 variant report

**Fig. 1:**
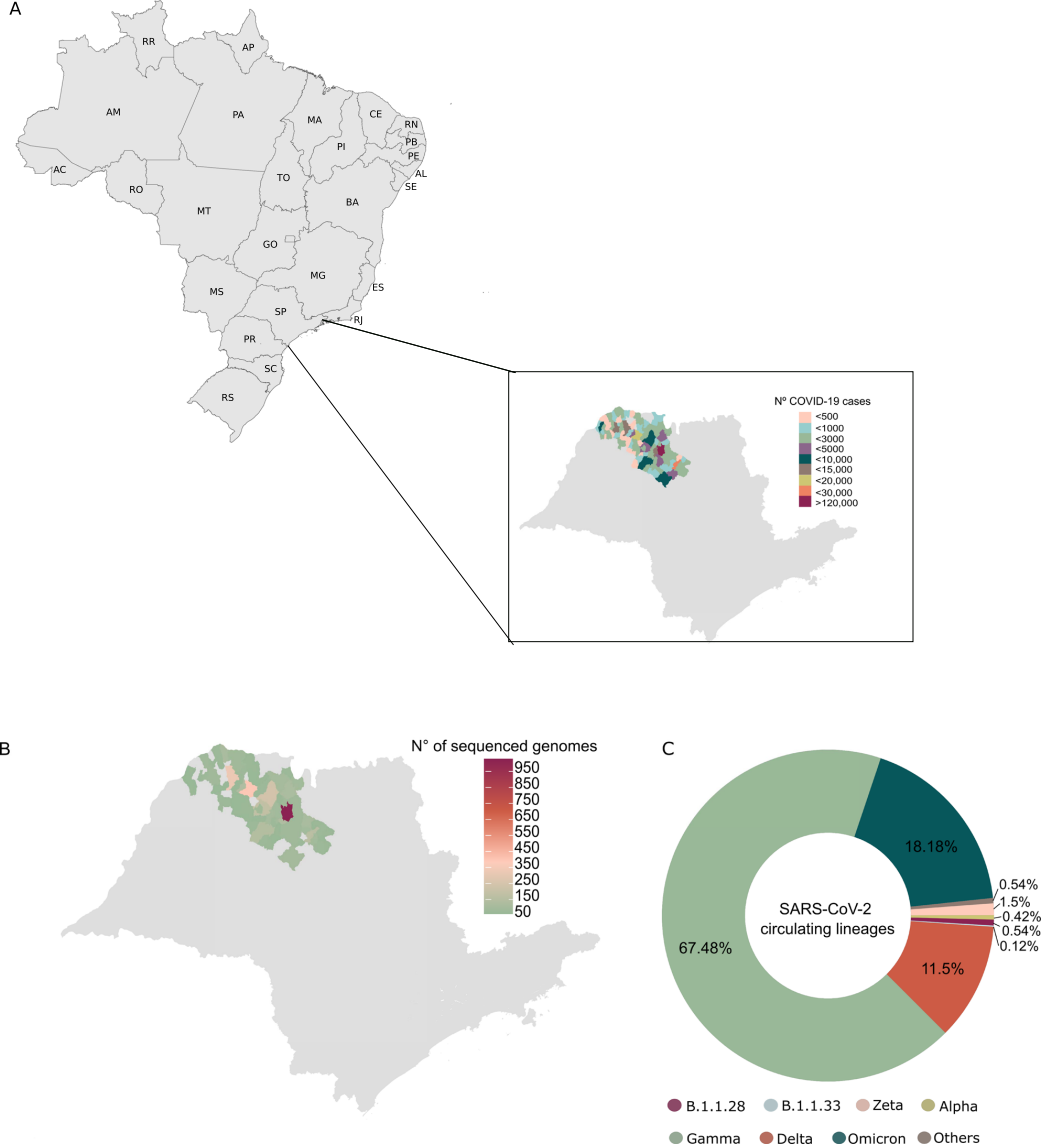
SARS-CoV-2 lineages genomic survaillance in northwestern of São Paulo state. **A** Map of Brazil showing the number of COVID-19 cases reported in the municipalities from the 15th Regional Health District (RHD XV) in northwestern São Paulo state from January 2021 to April 2022. **B** Map of São Paulo state showing the number of sequenced genomes obtained from the municipalities from the RHD XV from January 2021 to April 2022. **C** Prevalence of SARS-CoV-2 lineages detected in the municipalities from the RHD XV from January 2021 to April 2022 by genomic surveillance.

São Paulo state recorded the highest numbers of COVID-19 cases and deaths in Brazil^15,18^ in response to that, the São Paulo State Network for Pandemic Alert of Emerging SARS-CoV-2 Variants (SPNPAESV) was implemented in early 2021 to monitor the real-time evolution of circulating VOC and variants of interest (VOI) within the state. São Paulo state is divided into 17 regional health districts by the State Secretary of Health (SES-SP), facilitating the planning and articulation of health policies in alignment with the Brazilian national public health system (*Sistema Único de Saúde*, SUS)^20^. Owing to substantial efforts from various research centers across each regional district, the SPNPAESV successfully sequenced 3,306 complete SARS-CoV-2 genomes from the Regional Health District (RHD) XV encompassing 102 cities, including SJdRP in the northwestern part of São Paulo state (Fig. 1A).

In our study, SARS-CoV-2 sequences from 85 cities were included (Fig. 1B); sequences from SJdRP were the most representative, accounting for 29.3% of the sequenced genomes, and a total of 14 SARS-CoV-2 lineages were detected from January 2021 to April 2022. Of these lineages, the VOCs comprised most of the sequenced genomes, with Gamma being the most prevalent (*n* = 2,227, 67.48%), followed by Omicron (*n* = 600, 18.18%) and Delta (*n* = 381, 11.5%). Interestingly, the circulation of the Alpha variant (*n* = 1, 0.42%) was limited in the sampled municipalities. Other lineages, mostly detected in early 2021, were present at a low frequencies, including Zeta (*n* = 38, 1.15%), B.1.1.28 (*n* = 18, 0.54%), B.1.1.33 (*n* = 4, 0.12%), and others such as B.1, B.1.1, B.1.2, B.1.1.332, B.1.1.393, N.9, and P.4 (*n* = 18, 0.54%) (Fig. 1C, Supplementary Table 1).

**Fig. 2:**
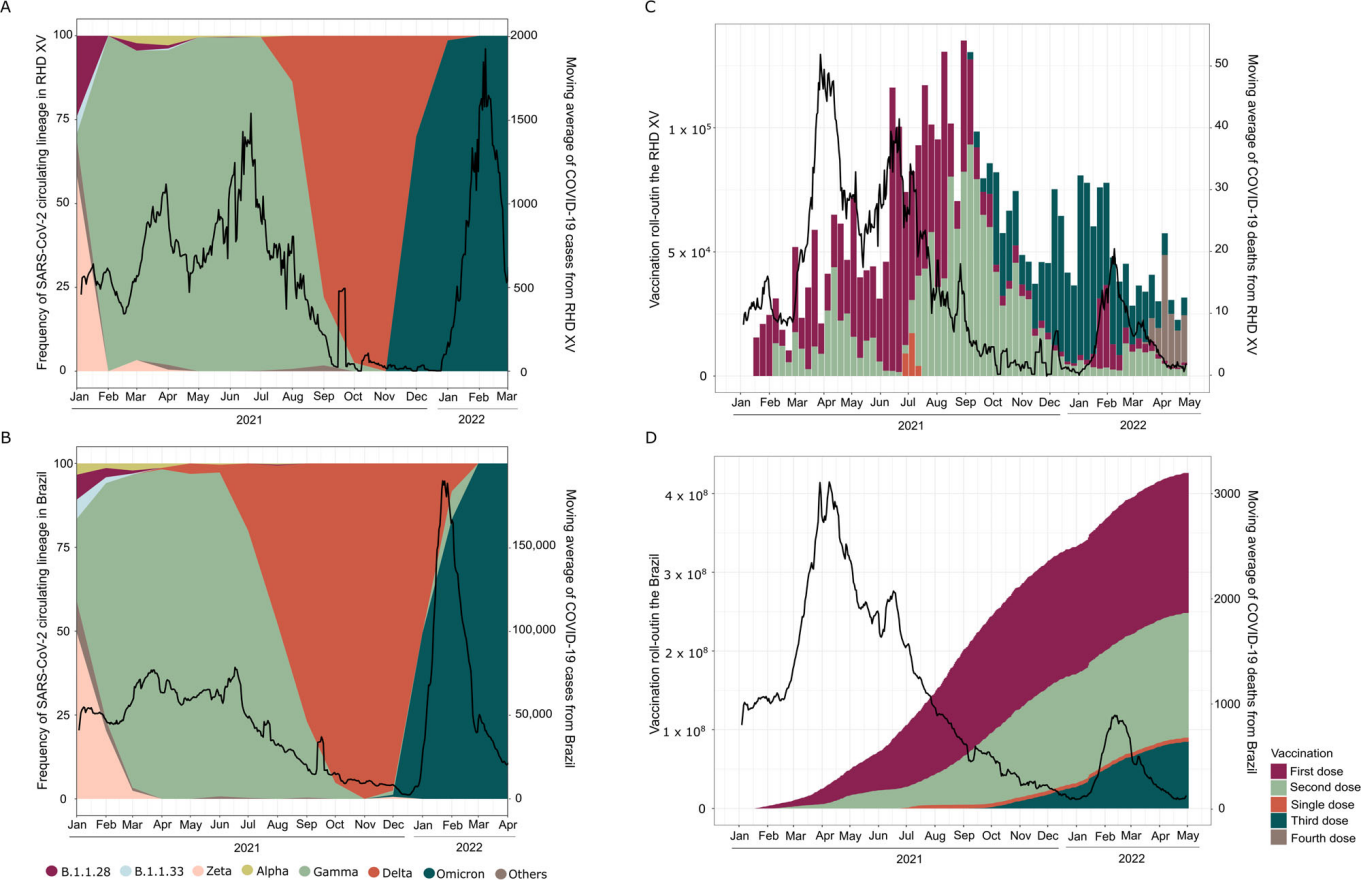
Genomic epidemiology of SARS-CoV-2 epidemics in northwestern São Paulo state versus Brazil. Proportion of SARS-CoV-2 lineages circulating over time and associations with the moving average of COVID-19 cases reported in municipalities from the 15^th^ regional Health District (RHD XV) (**A**) and Brazil (**B**); Vaccination coverage and its effect on the number of deaths reported in the RHD XV (**C**) and Brazil (**D**).

Analyzing the distribution of SARS-CoV-2 lineages over time in the municipalities within the RHD XV, we observed that Zeta was the prevalent VOI in January 2021 (58.32%) and co-circulated with the other five variants (Supplementary Table 1). The introduction of Gamma was detected in late January; from then on, we observed the replacement of nearly all the other circulating SARS-CoV-2 variants, along with a sharp increase in COVID-19 case numbers (Fig. 2A). Consequently, from March to September 2021, the VOC Gamma (P.1 and its sub-lineages) was the predominant lineage within the RHD XV. We identified the introduction of the Delta VOC in early August 2021 (in the city of Jales). This variant rapidly increased in frequency, replacing the Gamma variant by October of that year. Delta remained the predominant variant from October to December 2021, until it was displaced by Omicron (Fig. 2A). By analyzing Brazilian sequences from the same period, we observed a similar pattern of Gamma dominance and an increase in the national COVID-19 case numbers in early 2021 (Fig. 2B). This wave was followed by Delta replacement, which did not correspond to an increase in detected cases or death rates (Fig. 2B, D). Omicron’s subsequent introduction and spread towards the end of 2021 led to an unprecedented surge in COVID-19 cases, accompanied by only a modest increase in deaths (Fig. 2A-D).

**Fig. 3:**
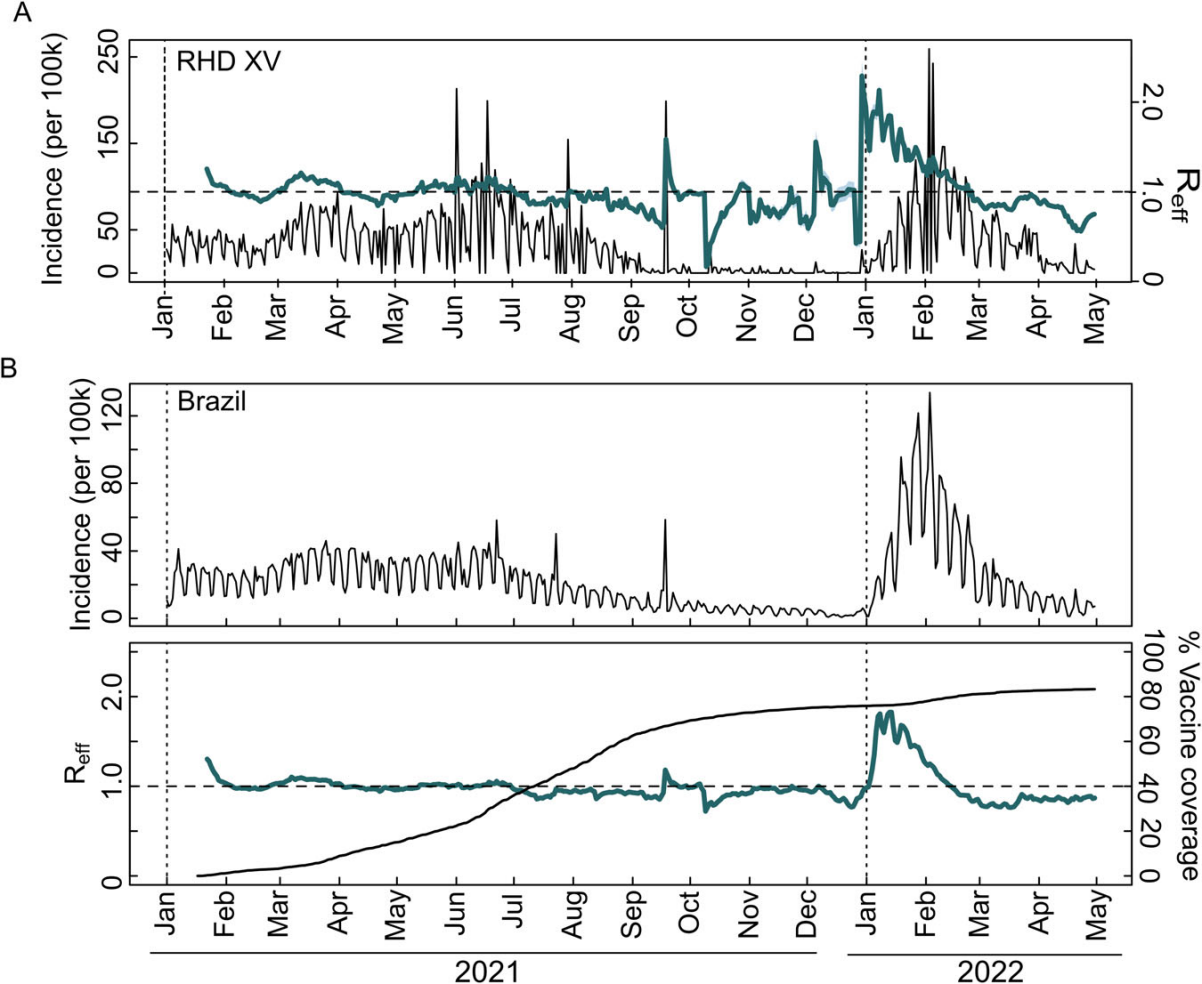
Prevalence and spread of SARS-CoV-2 infections in the 15th Regional Health District and Brazil. **A** Incidence of COVID-19 per 100,000 inhabitants in the RHD XV region on the left y-axis together with estimated effective reproductive number (Reff) for all COVID-19 cases in the region, represented as a blue line on the right y-axis; (**B**) COVID-19 incidence per 100,000 inhabitants for all cases across Brazil and estimated effective reproductive number (Reff) over time (blue line) for all reported COVID-19 cases (right y-axis), including the percentage of vaccine coverage in the entire population during the same timeframe (left y-axis).

Interestingly, despite Delta replacing Gamma in October 2021, there was no corresponding increase in case numbers. This scenario changed in December 2021 with the detection of Omicron, leading to a noticeable shift in case trends (Fig. 2A, B). Reinforcing these findings, the analysis of effective reproduction number (Reff) revealed peaks (Reff > 1) from March to April 2021, late September 2021, and December 2021 to January 2022, indicating a higher transmission rate (Fig. 3A, B). This increase in Reff corresponds with the introduction and growth in frequency of the Gamma, Delta, and Omicron VOCs in RHD XV municipalities and across Brazil (Fig. 3A, B). Notably, even though a large number of cases were reported from January 2022, death only marginally increased. This trend is likely due to the nature of reported cases rather than increased disease severity, contrasting with the period of Gamma dominance and reinforcing the protective effect of vaccination coverage (Fig. 2C, D).

**Fig. 4:**
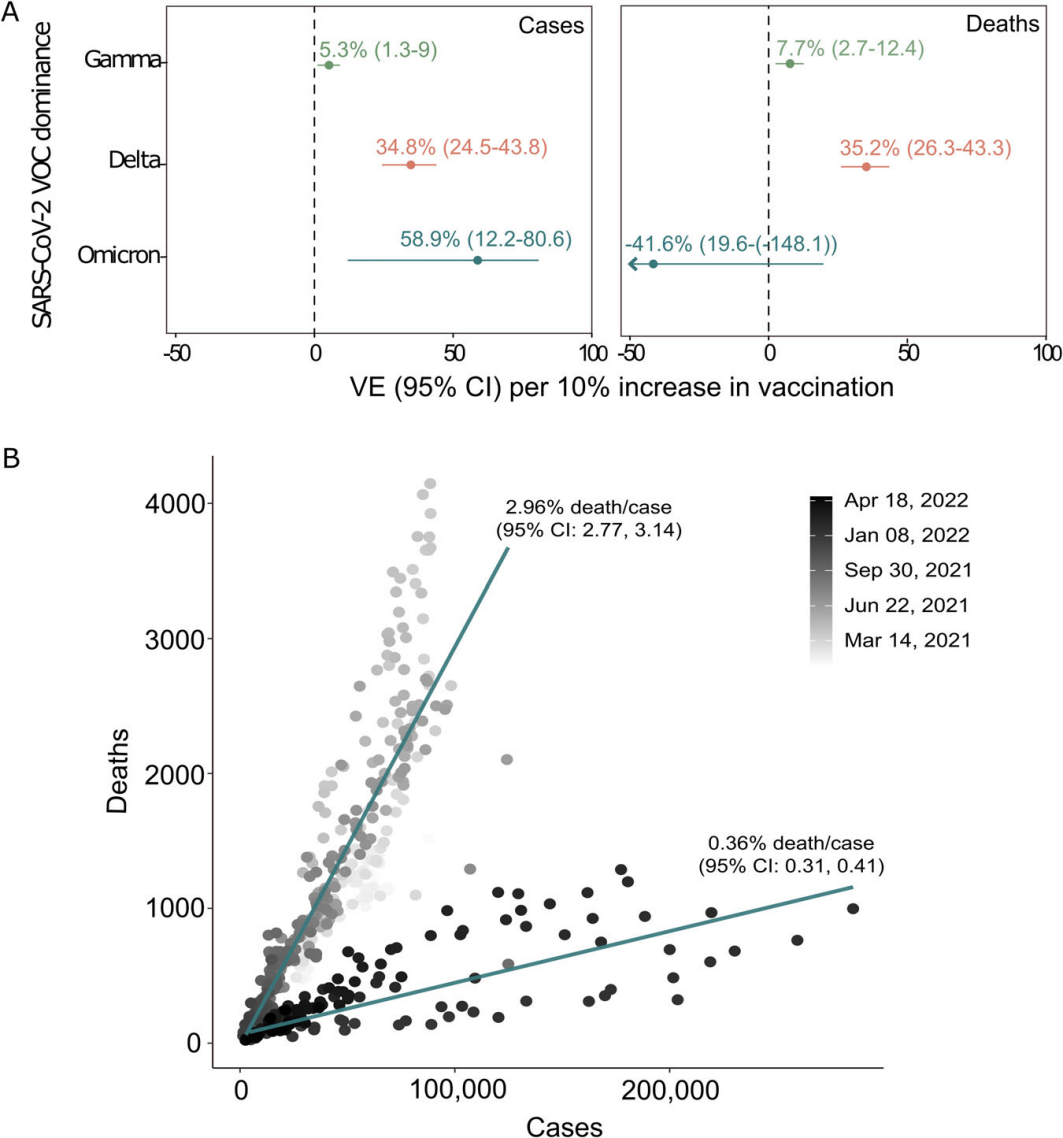
Vaccine effectiveness on the number of COVID-19 cases and deaths in Brazil. **A** Effectiveness of accination per 10% increase in vaccination uptake in preventing new COVID-19 cases and deaths in Brazil. **B** Number of new deaths per new cases observed in 2021 and 2022 in Brazil. VE: Vaccine effectiveness.

To further analyze the role of vaccination in Brazil relative to VOC dominance, we looked at the vaccine effectiveness for every 10% increase in overall vaccination uptake in terms of cases and death numbers, adjusting for the number of tests administered (Fig. 4A). We found that vaccine effectiveness in preventing cases during the period dominated by Delta and Omicron (1 August – 1 November 2021 and 1 December 2021−30 April 2022, respectively) were six and 11 times higher than when Gamma was the dominant variant (1 February−31 July 2021). Similarly, vaccine effectiveness in preventing deaths during Delta’s dominance was higher than Gamma’s. However, vaccination showed no statistical effect in preventing deaths during Omicron’s dominance, which could be anticipated if the case fatality rate varied over time independent of vaccination. This variation is illustrated in Fig. 4B, where the deaths-to-case ratio decreased from 2.96% (95% CI: 2.77, 3.14) before December 2021 to 0.36% (95% CI: 0.31, 0.41) from December 2021 onwards.

### SARS-CoV-2 phylodynamic analysis in the RHD XV region

**Fig. 5:**
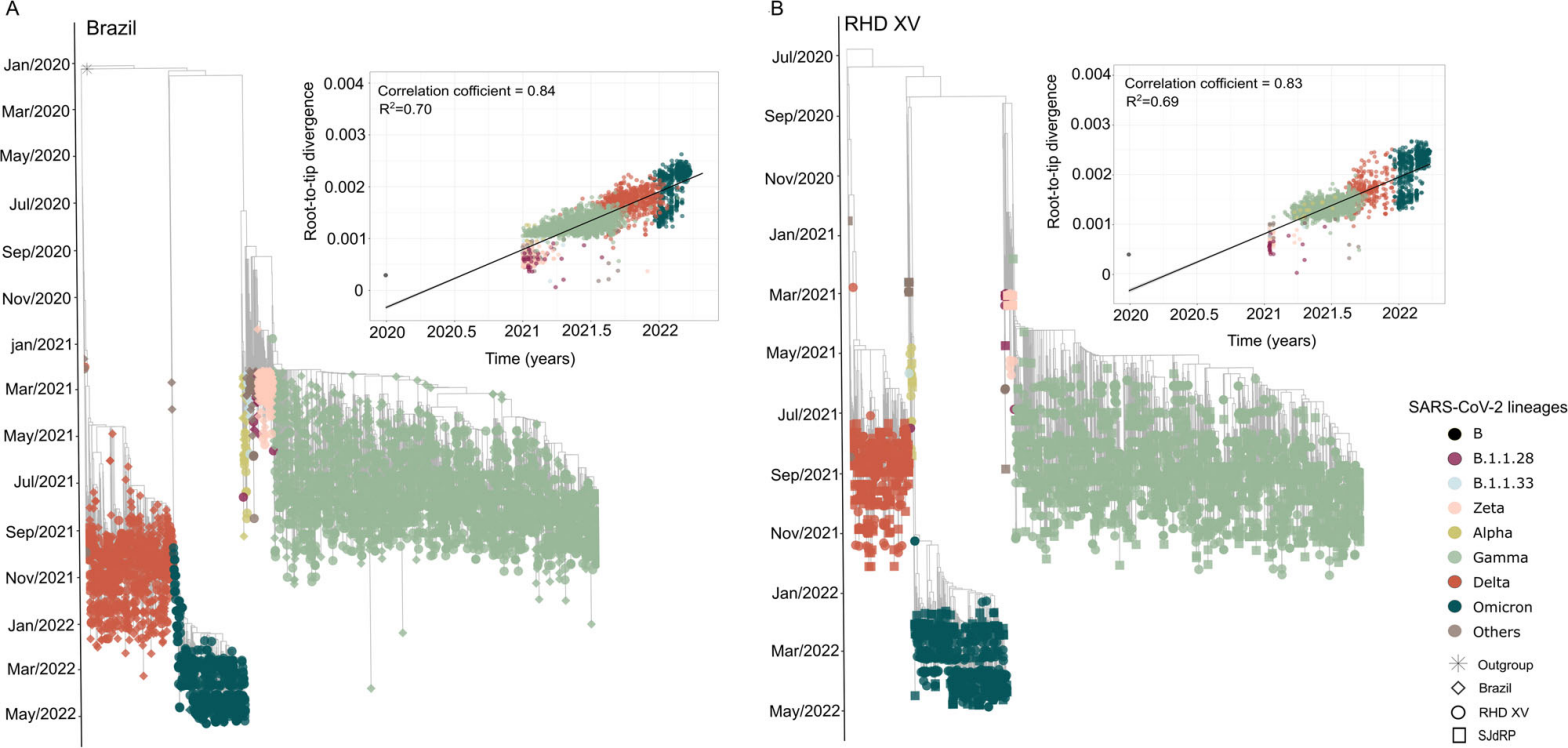
Maximum-likelihood tree for SARS-CoV-2 based on complete genome sequences from the 15th Regional Health District and Brazilian regions. **A** Time-stamped phylogenetic tree reconstructed using 4527 Brazilian complete genomes (3300 from this study) (Supplementary Table 2) and linear regression of root-to-tip genetic distance of different SARS-CoV-2 lineages versus sampling date. Colors represent different lineages and tip shapes represent different locations. **B** Time-stamped phylogenetic tree reconstructed using 3300 genome sequences from the RHD XV region, including 962 from SJdRP (Supplementary Table 1) and linear regression of root-to-tip genetic distance of different SARS-CoV-2 lineages versus sampling date. Colors represent different lineages and tip shapes represent different locations.

To better understand the dynamics of SARS-CoV-2 lineages and their spread in the study area, we investigated the phylodynamics of the different lineages detected in the RHD XV and the lineages circulating in Brazil during the sampled period. We reconstructed a time-scaled maximum likelihood (ML) tree using 1227 genomes from all Brazilian states and 3300 genomes from 85 cities from the RHD XV sequenced in this study (Fig. 5A, Supplementary Table 2). Phylogenetic analysis showed three main clades corresponding to the Gamma, Delta, and Omicron variants depicted the clade replacement events over time in Brazil. Importantly, in the different clades, we observed that RHD XV sequences were interspersed with Brazilian sequences from different geographic regions, suggesting several introduction events in the RHD region (Fig. 5A).

Similarly, to understand the dynamics of SARS-CoV-2 within the region of study, we built a time-scaled ML tree including only sequences from the RHD XV (Supplementary Table 1). Sequences from SJdRP were highlighted since this municipality had the most significant number of cases, sequenced genomes (Fig. 1A, B), and played an essential role during the COVID-19 pandemic. In the RHD XV Maximum Likelihood (ML) tree, we observed the clustering of sequences into three distinct groups, corresponding to the primary lineages of the virus identified in 2021 (Fig. 5B). Additionally, the sequences from SJdRP were found distributed across various clades, along with sequences from other cities in the RHD XV region (Fig. 5B), suggesting multiple virus importation and exportation from these areas.

### Spatiotemporal dispersion of the Gamma, Delta, and Omicron lineages

**Fig. 6:**
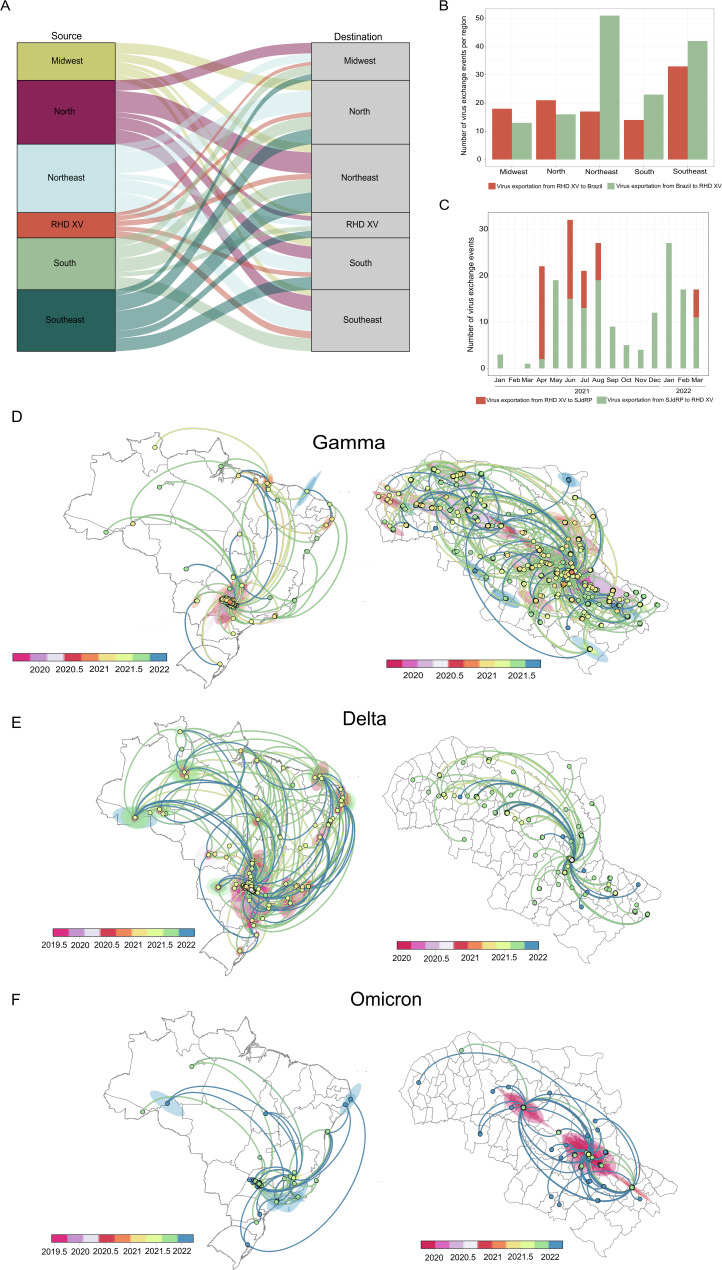
Spatiotemporal spread of Gamma, Delta, and Omicron variants in Brazil and within the 15th Regional Health District (RHD XV). **A** Number of all SARS-CoV-2 lineages exchanged between RHD XV municipalities and all Brazilian regions (North, Northeast, Midwest, Southeast, and South) from 2021 and early 2022. **B** Source of viral exports and imports of all SARS-CoV-2 lineages identified in 2021 and early 2022 from Brazil to cities in RHD XV and (**C**) from RHD XV to SJRP. Phylogeographic reconstruction and dispersion of the (**D**) Gamma variant, (**E**) Delta variant, and (**F**) Omicron variant in Brazil and in RHD XV municipalities. Shaded areas represent the 80% highest posterior density interval and depict the uncertainty of the phylogeographic estimates for each node. Curved lines show the links between nodes and viral movement to different locations. Circles represent nodes of the maximum clade credibility phylogeny, and their colors represent the inferred time of occurrence.

Based on our spatiotemporal analyses, we could infer the number of virus exchanges between the RHD XV and the Brazilian regions and between SJdRP and surrounding cities within the RHD XV region. We found that cities in the RHD XV exchanged SARS-CoV-2 variants with all country regions (Fig. 6A, Supplementary Table 3). The Northeast region of Brazil contributed with the highest number of imported virus variants to the RHD XV region (at least 51 importations) (*p* < 0.001, X^2^ = 28.516, df = 4) compared to all other regions, follwed by Southeastern states (at least 42 importations) (Fig. 6B). Interestingly, most of viral importations from Northeastern Brazil occured in January 2021 (23%), and from several states such as Bahia, Alagoas, Ceará and Pernambuco. In contrast, most variants exported from the RHD XV region were sent to the southeast, accounting for at least 33 exportation events (Fig. 6B, Supplementary Table 3). Analysis of transmission dynamics within the RHD XV showed that SJdRP contributed to virus exchange during 2021 (Fig. 6C). Most virus exchange were exports from SJdRP to other municipalities (at least 157). Interestingly, most of the importation events (at least 120) took place between April and August 2021, coinciding with the wide circulation of the Gamma variant in the RHD XV region (Fig. 6C, Supplementary Table 3).

Next, we investigated the spatiotemporal history of the VOCs involved in the three major clade replacement events that were responsible for at least two significant COVID-19 epidemic waves in Brazil during 2021. Our phylogeographic analyses traced the movement of the Gamma lineage across the different Brazilian regions and its interaction with the RHD XV region. This analysis revealed that the Gamma variant was a prominent presence in Brazil’s epidemiological landscape throughout the period it was prevalent. Additionally, our findings suggest that the initial Gamma sequences identified in the RHD XV region were likely imported from Brazil’s southeastern and northeastern states (Fig. 6D). Moreover, we observed a notable migration pattern for the Gamma lineage within the RHD XV region. The evidence suggests that this variant was first introduced in SJdRP and then rapidly spread to other municipalities, with a pronounced spread to neighboring cities (Fig. 6D).

The Delta variant, which started to replace the Gamma variant in Brazil and specifically in the RHD XV region by September 2021 (Fig. 2A, B), exhibited intense spread across all Brazilian regions. It notably moved between the southeastern, northeastern, and northern states and the RHD XV region (Fig. 6E). However, when analyzing virus movement through the RHD XV region, we observed a predominance of exportation events from SJdRP, but to a lesser extent compared to Gamma, which may reflect the lower number of cases identified in that period (Fig. 6E).

Importation and exportation of Omicron were observed among the RHD XV region and a few states in all Brazilian regions (Fig. 6F). As with Delta, Omicron spread mainly from SJdRP to several cities within the RHD XV region (Fig. 6C, F); despite the large number of cases reported after the introduction of Omicron, fewer importation and exportation events were observed compared to Gamma, which could suggest that most transmission occurred locally.

## Discussion

In a continent-spanning country like Brazil, limitations on real-time surveillance of SARS-CoV-2 variants have been reported due to different regional sequencing efforts^21^, which posed a severe challenge to monitor the origin of new mutations that may enhance virus fitness and persistence of the pandemic at the national level. A possible alternative is to implement genomic surveillance in a small region that exhibits constant virus exchange with several geographic locations and may reflect the progression of the pandemic nationally.

Our findings demonstrated successive clade replacement events, corresponding to the introduction and increased frequency of the SARS-CoV-2 VOCs Gamma, Delta, and Omicron, respectively. This scenario is concerning during a pandemic since subsequent introductions of new lineages may contribute to the resurgence of cases and continuation of the disease, as reported for dengue and influenza^22–26^. We found that the three SARS-CoV-2 variant replacements in 2021 and early 2022 were linked to two waves of COVID-19, as observed in other studies at local and national levels^19,21,27–29^. The Gamma lineage emerged in late 2020 in Manaus, the capital of Amazonas state^6,30^, and it rapidly spread throughout Brazil, replacing Zeta and all other circulating SARS-CoV-2 variants to become the dominant lineage within six months (March–August 2021)^19,21^. Additionally, this VOC’s higher transmissibility rates and immune evasion led to reinfections and breakthrough infections^31–37^. The negative impact of Gamma across Brazil was amplified by the slow vaccination rate of early 2021, its greater severity of the disease, and elevated mortality risk in non-vaccinated patients, factors combined to overload health care systems and leading to a record number of deaths^19,29^. Our data also showed that the increased vaccination coverage from July 2021 helped decrease the number of COVID-19 cases and deaths in the RHD XV region as well as in other regions of Brazil. As a result, the spread of Delta was reduced, leading to a significant decrease in the number of cases and deaths at the national level. This finding becomes even more evident considering that previous infection with the Gamma variant itself did not confer a neutralizing antibody titer against the Delta variant as high as the one achieved by vaccination^38^. These observations contrasted with what was observed internationally, as the emergence of Delta triggered a new epidemic wave in several countries with varying degrees of vaccine coverage, such as India, Indonesia, Thailand, Myanmar, Nepal, several African nations, the United Kingdom, and Israel^39–45^. The most likely reason was the high vaccine coverage in the population when Delta was imported to Brazil, as shown in our study. Reinforcing these findings, our Reff analysis demonstrated that when Delta replaced Gamma in September 2021, there was a slight increase in the reproduction number for SARS-CoV-2, quickly followed by a decrease, but this did not impact the number of cases reported at the time, suggesting that higher immune protection of the population helped contain virus spread. Indeed, by the time Delta increased in frequency in the RHD XV, most of the population had been fully vaccinated with two doses of the inactivated-virus Sinovac/CoronaVac vaccines, and booster distribution had begun. Our study shows that these efforts effectively prevented severe cases and deaths. Moreover, these results are reinforced by studies demonstrating the effectiveness of the CoronaVac booster in inducing a potent immune response and elevated virus-specific antibody levels, increasing Delta variant neutralization activity, and subsequently preventing infection and severe outcomes^38,46–48^.

After the introduction and spread of Omicron, we observed a new resurgence of cases (January 2022); however, there was only a slight increase in the number of deaths in municipalities within the RHD XV region, corroborating the epidemiological landscape seen in Brazil as a whole. The Omicron variant was detected in South Africa and Botswana in November 2021, alongside an exponential rise in the incidence of COVID-19^8^. Phylogenetic analyses estimated that Omicron emerged in October 2021 with a significant R0, contributing to its vast and rapid spread. Indeed^,^ our analyses showed a sharp peak in the Reff for SARS-CoV-2 (>2) when Omicron was introduced and increased in frequency, which was not observed when Gamma and Delta were the dominant variants, reinforcing the transmissibility of Omicron. The higher transmission rate of this variant is related to the constellation of mutations it displays: over 30 mutations in the spike glycoprotein, several in the receptor binding domain and N-terminal domain, which reduced its sensitivity to neutralization by anti-SARS-CoV-2 antibodies induced by previous infection or vaccination^8^. Despite Omicron’s pronounced transmissibility compared to Delta^49^ and higher immune evasion compared to previous VOCs^50^, it exhibited reduced severity, leading to lower hospitalization rates^51,52^. Our results showed this same pattern; even though the number of cases rose abruptly from January 2022 in cities in the RHD XV region and throughout Brazil, the number of deaths was much lower than previously observed. The main factor underlying this pattern is likely attributed to the high rates of booster vaccination among the population, which is shown to promote higher titers of neutralizing antibodies^53–56^ and strong protection against severe disease and death^57^.

Analyzing the spread of the different VOC in the RDH XV region and Brazil, our data showed that most of viral importation events to RHD XV region were from several states from Northeast region. Moreover, a more detailed analysis showed that the majority of the importations occurred in January 2021, a holiday and vacation periods in Brazil, in which a decrease in the social index isolation was observed in SJdRP^19^. Additionally, we observed lower virus exchange among the different Brazilian regions when Gamma was the dominant variant compared to Delta. One explanation is that by the time Gamma was circulating (March–August 2021), Brazil implemented more severe restrictions and social isolation, and vaccination was initiated^29^. However, when Delta replaced Gamma, the restriction measures had been loosened or abandoned, leading to higher virus exchange among the Brazilian states despite the lower numbers of notified COVID-19 cases. Nevertheless, an opposite pattern was observed in RHD XV since a more intense viral movement was reported when Gamma was the prevalent SARS-CoV-2 lineage. This likely resulted from the burst of COVID-19 cases reported from April 2021 that led to a record number of deaths and intensive care unit (ICU) occupancy rates. Because SJdRP has one of the most important centers for COVID-19 care and treatment, the city received people from various municipalities in the RHD XV, leading to higher rates of SARS-CoV-2 importation and exportation; still, when Delta was predominant, the population had been vaccinated full or partially that was fundamental for the decrease of transmission rates. Banho, Sacchetto, et al.^19^ showed that ICU occupancy reached 100% in SJdRP when Gamma was prevalent in the RHD XV, suggesting that the intense virus importations and exportations observed in 2021 were related to SJRP’s role as the headquarters of RHD XV and home to the main hospital responsible for SARS-CoV-2 diagnosis, receiving over 5700 admissions up to June 2021^19^. During Delta circulation, the vaccine coverage was high, which helped to decrease the severity of the disease, probably leading to lower demand for treatment in more specialized healthcare centers such as HB, and consequently impacting virus circulation and transmission among the cities within the district.

Interestingly, while we expected Omicron to display the same pattern as Delta in Brazil, we observed significantly less virus exchange. This may be related to the sampling coverage nationwide, since from December 2021 to April 2022, only 933 full-coverage complete genomes of SARS-CoV-2 classified as Omicron were available at GISAID (https://www.epicov.org/). The most Brazilian states from which Omicron genomes were retrieved showed poor sampling, which may have influenced the analysis and is a limitation of the study. Moreover, it is important to highlight that sampling representativeness differed for Gamma, Delta, and Omicron since SARS-CoV-2 genomes from 84, 41, and 28 RHD XV municipalities were sampled when these variants were circulating, respectively. This difference may limit the analysis of the spatiotemporal spread of SARS-CoV-2 whithin the RHD XV region. However, at the same time, it suggests that when infected people had milder symptoms (due to vaccine efficiency), fewer sought diagnoses in the health system, thus reducing the availability of collected samples for genomic surveillance.

Here we demonstrated that genomic surveillance of SARS-CoV-2 variants in SJdRP and neighboring cities can bring general insights about the national scenario of SARS-CoV-2 evolutionary course and transmission, mainly in midsize and major cities. However, it is important to highlight that this study has limitations, considering the spatiotemporal dynamic and virus importation and exportation events. This because, our data were based on SARS-CoV-2 genome sequences from capital and major cities of each state, meaning that, perhaps it does not reflect the pandemic progression in smaller cities of different Brazilian regions, that are located far away from larger and more populated municipalities, and which often have limited access to vaccination or other public health measures to contain the pandemic. Thus, additional analysis considering SARS-CoV-2 genomes from smaller cities of all Brazilian states are needed to reinforce our findings.

Therefore, using epidemiological data, genomic sequencing, and phylogeographic analyses, we demonstrated three well-defined clade replacement events in the RHD XV region, corresponding to the introduction and spread of the Gamma, Delta, and Omicron variants. The rapidly increasing prevalence of these VOCs triggered two COVID-19 epidemic waves, which were significantly influenced by the vaccination landscape. Our study revealed that the effectiveness of vaccination in mitigating new cases during Delta and Omicron circulation was six and eleven times higher, respectively, than during Gamma’s dominance. Additionally, vaccination coverage and booster doses were highly effective in reducing cases and deaths at local and national level. Thoroughly, our results revealed that SJdRP played a pivotal role in disseminating SARS-CoV-2 lineages to neighboring cities within the district. This underscores SJdRP’s suitability as a focal point for genomic surveillance, providing a reliable reflection of the national pattern of SARS-CoV-2 spread and evolution in midsize and major Brazilian cities.

## Material and methods

### Ethics statement

This study was approved by the Ethics Committee of the São José do Rio Preto School of Medicine (FAMERP) (protocol number: CAAE #31588920.0.0000.5415, on November 29, 2021). Written informed consent was waived by the institutional review board (IRB) since all samples were collected for routine diagnosis, and the data were analyzed anonymously, ensuring total confidentiality for all participants.

### Epidemiological data

Data from reported and confirmed COVID-19 cases in Brazil were provided by the Brazilian Ministry of Health and are available at https://github.com/wcota/covid19br^58^. To perform the analyses, we added the geographical locations according to Brazilian regions (North, Northeast, Southeast, South, Midwest, the Regional Health District XV (RHD XV) and São José do Rio Preto (SJdRP)). The effective reproduction number (Reff) for SARS-CoV-2 over the study period was estimated using the EpiEstim^59^ in R version 4.3.1^60^. We fit the time-varying Reff, assuming a parametric serial interval with a mean of five days using a 21-day sliding window. The Reff reflects the behavior of an epidemic, and by definition is the average number of secondary infections caused by an infected person at a given time, where *R* > 1 indicates a growing epidemic, while an *R* < 1 indicates a decrease in transmission. Negative binomial regressions of new cases or deaths by day were run with a 10% increase in the percentage of the vaccinated population, adjusting for the number of new tests for that day. Vaccine effectiveness and 95% confidence intervals (CI) were estimated as 1-exp(coefficient)) or 1-exp(lower or upper CI).

### Clinical samples and molecular investigation

To monitor the epidemiological profile and spatiotemporal dynamics of the SARS-CoV-2 variants that circulated during 2021–2022, convenience samples, collected from January 2021 to April 2022, presenting positive diagnoses for COVID-19 in residents of municipalities within the RHD XV were randomly selected by the SPNPAESV for whole-genome sequencing based on the cycle threshold value (≤30) and availability of epidemiological metadata such as date of sample collection and municipality of residence to perform phylogeographic analyses.

For COVID-19 diagnosis, viral RNA was extracted, from nasopharyngeal samples, using the Extracta kit fast DNA and RNA viral (MVXA-P096 FAST; Loccus, Brazil) according to the manufacturer’s instructions, utilizing an Extracta 96 DNA and RNA extractor and purifier (Loccus, Brazil). Reverse transcription followed by real-time polymerase chain reaction (RT-qPCR) was performed with the GeneFinder COVID-19 Plus RealAmp kit (OSANG Healthcare, Korea), targeting the RNA-dependent RNA polymerase (RdRp), envelope (E), and nucleocapsid (N) genes of the SARS-CoV-2 genome and the human RNAse P. The RT-qPCR was conducted in a QuantStudio 5 Real-Time PCR System (Thermo Fisher Scientific, USA), and the results were analyzed in QuantStudio 5 software v1.5.1 (Thermo Fisher Scientific, USA) interpreted as cycle threshold value (Ct) less or equal to 40 as positive.

### Whole-genome sequencing, genome assembling and lineage assignment

Samples presenting Ct value less or equal to 30 were randomly selected for whole-genome sequencing. Whole-genome amplification, and library preparation were performed using Illumina CovidSeq Test (Illumina Inc, USA), according to the instructions provided. The quality and size of the libraries were checked by Agilent 4150 TapeStation (Agilent Technologies Inc, USA). Libraries were pooled in equimolar concentrations, and the sequencing was conducted in the Illumina MiSeq System (Illumina Inc, USA), using MiSeq Reagent Kit v2 (2 × 150 bp cycles) (Illumina Inc, USA). The quality of the raw sequencing data was checked using FastQC software v. 0.11.9^61^ and trimmed with Trimmomatic v. 0.39^62^ to filter low-quality reads, low-quality bases, and reads with at least 75 base pairs (bp). The cleaned paired-end reads were mapped against the Wuhan-Hu-1 reference genome (NC_045512.2) using BWA mem v. 0.7.17 software^63^ and SAMtools v. 1.10^64^ for read sorting and indexing. Next, Pilon software^65^ was used to improve insertion and deletion detection. Finally, SAMtools v. 1.10^64^ was used to access the position depths in the BAM alignment, and SAMtools mpileup and iVar v. 1.3.1^66^ were used to generate the consensus genomes (nucleotide positions presenting read depth <10 were considered ‘N’). The generated genomes were subjected to the Pangolin COVID-19 Lineage Assigner Tool version v. 4.0.5^67^ to confirm the variant classification.

### Phylogenetic analysis

The datasets used for the phylogenetic analysis included Brazilian SARS-CoV-2 complete genome sequences of collected from January 2021 to April 2022 and the SARS-CoV-2 reference genome retrieved from the GISAID database^68^. For the Brazilian phylogeny, we used a total of 4520 whole genomes, and for the RHD XV tree, we used a total of 3293 whole-genome sequences obtained in this study (Supplementary Tables 1 and 2). All the Brazilian genomes used in this study were downloaded from the GISAID database^68^, based on the criteria high-coverage and metadata availability. Next, the sequences were selected according to the collection date (from January 2021 to April 2022) and the location (capital cities of each Brazilian state). Nucleotide sequences were aligned using MAFFT v. 7.271^69^. Time-scale phylogenetic trees using the maximum-likelihood (ML) method were reconstructed in IQ-TREE v. 2.0.3.7^70^, using the best-fit model of nucleotide substitution according to the Bayesian information criterion (BIC) inferred by the ModelFinder tool^71^. The reliability of branching patterns was tested using a combination of ultrafast bootstrap (UFBoot) and the SH-like approximate likelihood-ratio test (SH-aLRT)^72^. To investigate the temporal signal from the ML trees, we regressed root-to-tip genetic distances against sample collection dates using the TempEst tool v. 1.5.1^73^, considering correlation coefficient >0.4 to accept temporal structure^74^. Next, generated phylogenies were submitted to TreeTime v. 0.9.3^75^ to convert the raw ML trees into time-scaled trees, considering a constant molecular rate of 8.0 × 10^−4^ nucleotide substitutions per site per year, according to Giovanetti et al.^29^. Finally, we used the time-scaled tree topologies to infer the number of viral exchange events between the five Brazilian regions and the RHD XV as well as within the RHD XV using TreeTime mugration v. 0.9.3, and by mapping the locations to tips and internal nodes from the annotated tree topology we were able to estimate the number of transition events (virus importations and exportations) among regions/cities^75^.

### Phylogeography analysis

To better understand the spatiotemporal history of SARS-CoV-2 spread and transmission within the RHD XV and between this district and other regions of Brazil, we investigated the main variants circulating in the region in 2021 and early 2022. To do so, we identified monophyletic clades in the time-scaled phylogenetic trees for the main VOC circulating in 2021 (Gamma, Delta, and Omicron) and randomly extracted sequences from all different clades in each monophyletic group using the Microreact web application^76^ to infer continuous phylogeography histories using the Markov chain Monte Carlo (MCMC) method in BEAST v1.10.4 software^77^, as described by Giovanetti et al.^29^. As a result of this, for each lineage we reconstructed an ML tree and accessed the molecular clock signal using the root-to-tip regression method implemented in TempEst v. 1.5.3^73^ and removed outliers that may violate the molecular clock assumption. Next, we down-sampled the lineages to <600 taxa per clade to infer the phylogeography history using BEAST v1.10.4^77^ and employing HKY as the nucleotide substitution model, a strict molecular clock and Bayesian skyline model as the coalescent tree prior. We also utilized a flexible relaxed random walk diffusion model^78,79^ with Cauchy distribution and jitter window site of 0.01 to model the phylogenetic diffusion and spread of each lineage among the Brazilian regions and within the RHD XV. The MCMC chains were run for 250 million interactions and sampled every 25,000 steps. Convergence was assessed in Tracer v. 1.7^80^, and maximum clade credibility trees were summarized using Treeannotator v. 1.6.1 after discarding the initial 10% of steps as burn-in. Finally, SERAPHIM^81^, a package in R software v. 4.2.3^60^, was used to extract and map the spatiotemporal information in the posterior trees.

### Geoprocessing

Databases that included the number of COVID-19 cases per municipality and the number of SARS-CoV-2 genomes sequenced per city were created according to the location/municipality of origin and sample collection date. Maps were created using R software version 3.6.3^82^. The shapefiles used in this study are available at: https://www.ibge.gov.br/geociencias/organizacao-do-territorio/malhas-territoriais/15774-malhas.html^61^.

### Supplementary information

Supplementary Information

## Data Availability

All SARS-CoV-2 genomes generated and analyzed in this study are available in the EpiCoV database in GISAID^68^, and their respective access numbers are provided in the Supplementary Tables 1 and 2.

## Code availability

Code for analysis and figures is publicly available on (https://github.com/cab1992/SARS-CoV-2_genomic_surveillance).
