# Supplementary material for: Dynamic clade transitions and the influence of vaccination on the spatiotemporal circulation of SARS-CoV-2 variants
Source: NPJ Vaccines. 2024 Aug 10;9:145. doi: 10.1038/s41541-024-00933-w (PMC11316783; doi:10.1038/s41541-024-00933-w)
Supplement: Supplementary file 1 — Supplementary Information [file 41541_2024_933_MOESM1_ESM.pdf]

Supplementary Table 1

Supplementar Table 1. SARS-CoV-2 whole genome sequences generated in this study, from January 2021 to April 2022.

GISAID Access Number	Collection Date	Brazilian Region Classification	Lineage Classification
EPI_ISL_2156471	2021-01-14	RHD XV	B.1.1.28
EPI_ISL_2544854	2021-01-14	RHD XV	Zeta
EPI_ISL_2544855	2021-01-15	RHD XV	Zeta
EPI_ISL_2155088	2021-01-16	RHD XV	B.1.1.28
EPI_ISL_2544848	2021-01-16	RHD XV	B.1.1.28
EPI_ISL_2544849	2021-01-16	RHD XV	B.1.1.28
EPI_ISL_2544838	2021-01-16	RHD XV	B.1.1.28
EPI_ISL_2544851	2021-01-16	RHD XV	B.1.1.28
EPI_ISL_2107304	2021-01-16	RHD XV	B.1.1.28
EPI_ISL_1785610	2021-01-16	RHD XV	B.1.1.28
EPI_ISL_2544837	2021-01-16	RHD XV	B.1.1.33
EPI_ISL_2544841	2021-01-16	RHD XV	Others
EPI_ISL_2544842	2021-01-16	RHD XV	Others
EPI_ISL_2013855	2021-01-16	RHD XV	Others
EPI_ISL_2156096	2021-01-16	RHD XV	Others
EPI_ISL_2544853	2021-01-16	RHD XV	Others
EPI_ISL_1785611	2021-01-16	RHD XV	Zeta
EPI_ISL_2544840	2021-01-16	RHD XV	Zeta
EPI_ISL_2543759	2021-01-16	RHD XV	Zeta
EPI_ISL_1785613	2021-01-16	RHD XV	Zeta
EPI_ISL_2013854	2021-01-16	RHD XV	Zeta
EPI_ISL_2155377	2021-01-16	RHD XV	Zeta
EPI_ISL_2155841	2021-01-16	RHD XV	Zeta
EPI_ISL_2156051	2021-01-16	RHD XV	Zeta
EPI_ISL_2544843	2021-01-16	RHD XV	Zeta
EPI_ISL_2544844	2021-01-16	RHD XV	Zeta
EPI_ISL_2544845	2021-01-16	RHD XV	Zeta
EPI_ISL_2544846	2021-01-16	RHD XV	Zeta
EPI_ISL_2544847	2021-01-16	RHD XV	Zeta
EPI_ISL_2544850	2021-01-16	RHD XV	Zeta
EPI_ISL_2156152	2021-01-16	RHD XV	Zeta
EPI_ISL_2156261	2021-01-16	RHD XV	Zeta
EPI_ISL_2544852	2021-01-16	RHD XV	Zeta
EPI_ISL_1785614	2021-01-16	RHD XV	Zeta
EPI_ISL_1785609	2021-01-16	RHD XV	Zeta
EPI_ISL_2544839	2021-01-16	RHD XV	Zeta
EPI_ISL_2544856	2021-01-17	RHD XV	B.1.1.28
EPI_ISL_2544857	2021-01-18	RHD XV	B.1.1.28
EPI_ISL_2544858	2021-01-18	RHD XV	B.1.1.28
EPI_ISL_2008939	2021-01-18	RHD XV	B.1.1.28
EPI_ISL_2544874	2021-01-25	RHD XV	B.1.1.33
EPI_ISL_2544875	2021-01-25	RHD XV	Zeta
EPI_ISL_2544862	2021-01-26	RHD XV	B.1.1.28
EPI_ISL_2544873	2021-01-26	RHD XV	B.1.1.28
EPI_ISL_2544876	2021-01-26	RHD XV	B.1.1.33
EPI_ISL_2544864	2021-01-26	RHD XV	Gamma
EPI_ISL_2544870	2021-01-26	RHD XV	Others
EPI_ISL_2544859	2021-01-26	RHD XV	Zeta
EPI_ISL_2544860	2021-01-26	RHD XV	Zeta
EPI_ISL_2544861	2021-01-26	RHD XV	Zeta
EPI_ISL_2544863	2021-01-26	RHD XV	Zeta
EPI_ISL_2544865	2021-01-26	RHD XV	Zeta
EPI_ISL_2544866	2021-01-26	RHD XV	Zeta
EPI_ISL_2544867	2021-01-26	RHD XV	Zeta
EPI_ISL_2544868	2021-01-26	RHD XV	Zeta
EPI_ISL_2544869	2021-01-26	RHD XV	Zeta
EPI_ISL_2544871	2021-01-26	RHD XV	Zeta
EPI_ISL_2544872	2021-01-26	RHD XV	Zeta
EPI_ISL_1941583	2021-02-26	RHD XV	Gamma
EPI_ISL_2008938	2021-02-27	RHD XV	Gamma

EPI_ISL_3761750	2021-03-05	RHD XV	Gamma
EPI_ISL_3761751	2021-03-05	RHD XV	Gamma
EPI_ISL_1785612	2021-03-08	RHD XV	B.1.1.28
EPI_ISL_1754186	2021-03-11	RHD XV	Gamma
EPI_ISL_5801938	2021-03-17	RHD XV	Gamma
EPI_ISL_5801939	2021-03-17	RHD XV	Gamma
EPI_ISL_5801940	2021-03-17	RHD XV	Gamma
EPI_ISL_5801941	2021-03-17	RHD XV	Gamma
EPI_ISL_5801942	2021-03-18	RHD XV	Gamma
EPI_ISL_5801943	2021-03-18	RHD XV	Gamma
EPI_ISL_5801944	2021-03-18	RHD XV	Gamma
EPI_ISL_1795233	2021-03-21	RHD XV	Gamma
EPI_ISL_1795276	2021-03-21	RHD XV	Gamma
EPI_ISL_1795277	2021-03-22	RHD XV	Gamma
EPI_ISL_1795279	2021-03-22	RHD XV	Gamma
EPI_ISL_1795280	2021-03-22	RHD XV	Gamma
EPI_ISL_1795281	2021-03-22	RHD XV	Gamma
EPI_ISL_1795282	2021-03-22	RHD XV	Gamma
EPI_ISL_1795283	2021-03-22	RHD XV	Gamma
EPI_ISL_1795285	2021-03-22	RHD XV	Gamma
EPI_ISL_1795286	2021-03-22	RHD XV	Gamma
EPI_ISL_1795232	2021-03-23	RHD XV	Gamma
EPI_ISL_1795239	2021-03-23	RHD XV	Gamma
EPI_ISL_1795242	2021-03-23	RHD XV	Gamma
EPI_ISL_1795243	2021-03-23	RHD XV	Gamma
EPI_ISL_1795244	2021-03-23	RHD XV	Gamma
EPI_ISL_1795245	2021-03-23	RHD XV	Gamma
EPI_ISL_1795246	2021-03-23	RHD XV	Gamma
EPI_ISL_1795247	2021-03-23	RHD XV	Gamma
EPI_ISL_1795248	2021-03-23	RHD XV	Gamma
EPI_ISL_1795249	2021-03-23	RHD XV	Gamma
EPI_ISL_1795250	2021-03-23	RHD XV	Gamma
EPI_ISL_1795251	2021-03-23	RHD XV	Gamma
EPI_ISL_1795252	2021-03-23	RHD XV	Gamma
EPI_ISL_1795253	2021-03-23	RHD XV	Gamma
EPI_ISL_1795254	2021-03-23	RHD XV	Gamma
EPI_ISL_1795255	2021-03-23	RHD XV	Gamma
EPI_ISL_1795256	2021-03-23	RHD XV	Gamma
EPI_ISL_1795257	2021-03-23	RHD XV	Gamma
EPI_ISL_1795266	2021-03-23	RHD XV	Gamma
EPI_ISL_1795269	2021-03-23	RHD XV	Gamma
EPI_ISL_1795270	2021-03-23	RHD XV	Gamma
EPI_ISL_1795272	2021-03-23	RHD XV	Gamma
EPI_ISL_1795278	2021-03-23	RHD XV	Gamma
EPI_ISL_1795284	2021-03-23	RHD XV	Gamma
EPI_ISL_5802040	2021-03-23	RHD XV	Gamma
EPI_ISL_1795415	2021-03-23	RHD XV	Zeta
EPI_ISL_1795079	2021-03-24	RHD XV	Alpha
EPI_ISL_1795234	2021-03-24	RHD XV	Gamma
EPI_ISL_1795235	2021-03-24	RHD XV	Gamma
EPI_ISL_1795236	2021-03-24	RHD XV	Gamma
EPI_ISL_1795237	2021-03-24	RHD XV	Gamma
EPI_ISL_1795238	2021-03-24	RHD XV	Gamma
EPI_ISL_1795240	2021-03-24	RHD XV	Gamma
EPI_ISL_1795241	2021-03-24	RHD XV	Gamma
EPI_ISL_1795389	2021-03-24	RHD XV	Gamma
EPI_ISL_1795258	2021-03-24	RHD XV	Gamma
EPI_ISL_1795259	2021-03-24	RHD XV	Gamma
EPI_ISL_1795260	2021-03-24	RHD XV	Gamma
EPI_ISL_1795261	2021-03-24	RHD XV	Gamma
EPI_ISL_1795262	2021-03-24	RHD XV	Gamma
EPI_ISL_1795263	2021-03-24	RHD XV	Gamma
EPI_ISL_1795264	2021-03-24	RHD XV	Gamma

EPI_ISL_1795265	2021-03-24	RHD XV	Gamma
EPI_ISL_1795267	2021-03-24	RHD XV	Gamma
EPI_ISL_1795268	2021-03-24	RHD XV	Gamma
EPI_ISL_1795271	2021-03-24	RHD XV	Gamma
EPI_ISL_1795273	2021-03-24	RHD XV	Gamma
EPI_ISL_1795287	2021-03-24	RHD XV	Gamma
EPI_ISL_1795288	2021-03-24	RHD XV	Gamma
EPI_ISL_1795289	2021-03-24	RHD XV	Gamma
EPI_ISL_2008940	2021-03-24	RHD XV	Gamma
EPI_ISL_1795416	2021-03-24	RHD XV	Zeta
EPI_ISL_2154703	2021-03-25	RHD XV	Gamma
EPI_ISL_2154956	2021-03-25	RHD XV	Gamma
EPI_ISL_5802165	2021-03-30	RHD XV	Alpha
EPI_ISL_5802149	2021-03-30	RHD XV	Gamma
EPI_ISL_5802150	2021-03-30	RHD XV	Gamma
EPI_ISL_5802154	2021-03-30	RHD XV	Gamma
EPI_ISL_5802160	2021-03-30	RHD XV	Gamma
EPI_ISL_5802162	2021-03-30	RHD XV	Gamma
EPI_ISL_5802166	2021-03-30	RHD XV	Gamma
EPI_ISL_5802169	2021-03-30	RHD XV	Gamma
EPI_ISL_5802170	2021-03-30	RHD XV	Gamma
EPI_ISL_5802171	2021-03-30	RHD XV	Gamma
EPI_ISL_5802175	2021-03-30	RHD XV	Others
EPI_ISL_5802174	2021-03-30	RHD XV	Zeta
EPI_ISL_5802167	2021-03-31	RHD XV	Gamma
EPI_ISL_5802172	2021-03-31	RHD XV	Gamma
EPI_ISL_5802173	2021-03-31	RHD XV	Gamma
EPI_ISL_5800168	2021-04-07	RHD XV	Gamma
EPI_ISL_5800176	2021-04-07	RHD XV	Gamma
EPI_ISL_5800177	2021-04-07	RHD XV	Gamma
EPI_ISL_5800178	2021-04-07	RHD XV	Gamma
EPI_ISL_5800185	2021-04-07	RHD XV	Gamma
EPI_ISL_5800186	2021-04-07	RHD XV	Gamma
EPI_ISL_5800187	2021-04-07	RHD XV	Gamma
EPI_ISL_5800188	2021-04-07	RHD XV	Gamma
EPI_ISL_5800189	2021-04-07	RHD XV	Gamma
EPI_ISL_5800190	2021-04-07	RHD XV	Gamma
EPI_ISL_5800191	2021-04-07	RHD XV	Gamma
EPI_ISL_5800193	2021-04-07	RHD XV	Gamma
EPI_ISL_5800195	2021-04-07	RHD XV	Gamma
EPI_ISL_5800196	2021-04-07	RHD XV	Gamma
EPI_ISL_5800198	2021-04-07	RHD XV	Gamma
EPI_ISL_5800199	2021-04-07	RHD XV	Gamma
EPI_ISL_5800180	2021-04-07	RHD XV	Zeta
EPI_ISL_5800200	2021-04-08	RHD XV	Gamma
EPI_ISL_2443061	2021-04-09	RHD XV	Alpha
EPI_ISL_2443059	2021-04-09	RHD XV	Gamma
EPI_ISL_2443058	2021-04-09	RHD XV	Gamma
EPI_ISL_2443065	2021-04-09	RHD XV	Gamma
EPI_ISL_2443060	2021-04-09	RHD XV	Gamma
EPI_ISL_2443064	2021-04-09	RHD XV	Gamma
EPI_ISL_2443063	2021-04-09	RHD XV	Gamma
EPI_ISL_2443062	2021-04-09	RHD XV	Gamma
EPI_ISL_5800451	2021-04-14	RHD XV	Gamma
EPI_ISL_5800452	2021-04-14	RHD XV	Gamma
EPI_ISL_5800453	2021-04-14	RHD XV	Gamma
EPI_ISL_5800458	2021-04-14	RHD XV	Gamma
EPI_ISL_5800459	2021-04-14	RHD XV	Gamma
EPI_ISL_5800460	2021-04-14	RHD XV	Gamma
EPI_ISL_5800467	2021-04-14	RHD XV	Gamma
EPI_ISL_5800477	2021-04-14	RHD XV	Gamma
EPI_ISL_5800485	2021-04-14	RHD XV	Gamma
EPI_ISL_5800486	2021-04-14	RHD XV	Gamma

EPI_ISL_3048955	2021-04-14	RHD XV	Gamma
EPI_ISL_5800481	2021-04-15	RHD XV	Alpha
EPI_ISL_5800490	2021-04-15	RHD XV	B.1.1.28
EPI_ISL_5800466	2021-04-15	RHD XV	Gamma
EPI_ISL_5800471	2021-04-15	RHD XV	Gamma
EPI_ISL_5800474	2021-04-15	RHD XV	Gamma
EPI_ISL_5800475	2021-04-15	RHD XV	Gamma
EPI_ISL_5800478	2021-04-15	RHD XV	Gamma
EPI_ISL_5800480	2021-04-15	RHD XV	Gamma
EPI_ISL_5800482	2021-04-15	RHD XV	Gamma
EPI_ISL_5800483	2021-04-15	RHD XV	Gamma
EPI_ISL_5800484	2021-04-15	RHD XV	Gamma
EPI_ISL_5800487	2021-04-15	RHD XV	Gamma
EPI_ISL_5800489	2021-04-15	RHD XV	Gamma
EPI_ISL_5800491	2021-04-15	RHD XV	Gamma
EPI_ISL_5800492	2021-04-15	RHD XV	Gamma
EPI_ISL_5800493	2021-04-15	RHD XV	Gamma
EPI_ISL_3048954	2021-04-15	RHD XV	Gamma
EPI_ISL_2107305	2021-04-19	RHD XV	B.1.1.33
EPI_ISL_3048956	2021-04-19	RHD XV	Gamma
EPI_ISL_3048957	2021-04-19	RHD XV	Gamma
EPI_ISL_3761525	2021-04-19	RHD XV	Gamma
EPI_ISL_3048958	2021-04-19	RHD XV	Gamma
EPI_ISL_2008944	2021-04-19	RHD XV	Gamma
EPI_ISL_2008945	2021-04-19	RHD XV	Gamma
EPI_ISL_2008946	2021-04-19	RHD XV	Gamma
EPI_ISL_2008947	2021-04-19	RHD XV	Gamma
EPI_ISL_2008948	2021-04-19	RHD XV	Gamma
EPI_ISL_2008949	2021-04-19	RHD XV	Gamma
EPI_ISL_2008950	2021-04-19	RHD XV	Gamma
EPI_ISL_2008951	2021-04-19	RHD XV	Gamma
EPI_ISL_2107303	2021-04-19	RHD XV	Gamma
EPI_ISL_2008952	2021-04-19	RHD XV	Gamma
EPI_ISL_2008953	2021-04-19	RHD XV	Gamma
EPI_ISL_2008954	2021-04-19	RHD XV	Gamma
EPI_ISL_2008955	2021-04-19	RHD XV	Gamma
EPI_ISL_2008956	2021-04-19	RHD XV	Gamma
EPI_ISL_2008957	2021-04-19	RHD XV	Gamma
EPI_ISL_2008958	2021-04-19	RHD XV	Gamma
EPI_ISL_2008959	2021-04-19	RHD XV	Gamma
EPI_ISL_2008960	2021-04-19	RHD XV	Gamma
EPI_ISL_2008961	2021-04-19	RHD XV	Gamma
EPI_ISL_2107302	2021-04-19	RHD XV	Gamma
EPI_ISL_2008962	2021-04-19	RHD XV	Gamma
EPI_ISL_2008963	2021-04-19	RHD XV	Gamma
EPI_ISL_2008964	2021-04-19	RHD XV	Gamma
EPI_ISL_2008965	2021-04-19	RHD XV	Gamma
EPI_ISL_2008966	2021-04-19	RHD XV	Gamma
EPI_ISL_2008967	2021-04-19	RHD XV	Gamma
EPI_ISL_5800757	2021-04-20	RHD XV	Alpha
EPI_ISL_5800822	2021-04-20	RHD XV	Alpha
EPI_ISL_5800801	2021-04-20	RHD XV	B.1.1.28
EPI_ISL_5800759	2021-04-20	RHD XV	Gamma
EPI_ISL_5800760	2021-04-20	RHD XV	Gamma
EPI_ISL_5800779	2021-04-20	RHD XV	Gamma
EPI_ISL_5800780	2021-04-20	RHD XV	Gamma
EPI_ISL_5800791	2021-04-20	RHD XV	Gamma
EPI_ISL_5800797	2021-04-20	RHD XV	Gamma
EPI_ISL_5800813	2021-04-20	RHD XV	Gamma
EPI_ISL_5800818	2021-04-20	RHD XV	Gamma
EPI_ISL_5800821	2021-04-20	RHD XV	Gamma
EPI_ISL_5800826	2021-04-20	RHD XV	Gamma
EPI_ISL_5800827	2021-04-20	RHD XV	Gamma

EPI_ISL_5800829	2021-04-20	RHD XV	Gamma
EPI_ISL_5800831	2021-04-20	RHD XV	Gamma
EPI_ISL_5800847	2021-04-20	RHD XV	Gamma
EPI_ISL_3761749	2021-04-20	RHD XV	Gamma
EPI_ISL_5800794	2021-04-21	RHD XV	Gamma
EPI_ISL_5800802	2021-04-21	RHD XV	Gamma
EPI_ISL_5800803	2021-04-21	RHD XV	Gamma
EPI_ISL_5800804	2021-04-21	RHD XV	Gamma
EPI_ISL_5800805	2021-04-21	RHD XV	Gamma
EPI_ISL_5800809	2021-04-21	RHD XV	Gamma
EPI_ISL_5800810	2021-04-21	RHD XV	Gamma
EPI_ISL_5800811	2021-04-21	RHD XV	Gamma
EPI_ISL_5800812	2021-04-21	RHD XV	Gamma
EPI_ISL_5800828	2021-04-21	RHD XV	Gamma
EPI_ISL_5800835	2021-04-21	RHD XV	Gamma
EPI_ISL_5800837	2021-04-21	RHD XV	Gamma
EPI_ISL_5800844	2021-04-21	RHD XV	Gamma
EPI_ISL_5800849	2021-04-21	RHD XV	Gamma
EPI_ISL_5800792	2021-04-21	RHD XV	Others
EPI_ISL_5800793	2021-04-21	RHD XV	Others
EPI_ISL_5800814	2021-04-22	RHD XV	Gamma
EPI_ISL_5800816	2021-04-22	RHD XV	Gamma
EPI_ISL_5800823	2021-04-22	RHD XV	Gamma
EPI_ISL_5800824	2021-04-22	RHD XV	Gamma
EPI_ISL_5800825	2021-04-22	RHD XV	Gamma
EPI_ISL_5800830	2021-04-22	RHD XV	Gamma
EPI_ISL_5800833	2021-04-22	RHD XV	Gamma
EPI_ISL_5800834	2021-04-22	RHD XV	Gamma
EPI_ISL_5800836	2021-04-22	RHD XV	Gamma
EPI_ISL_5800838	2021-04-22	RHD XV	Gamma
EPI_ISL_5800839	2021-04-22	RHD XV	Gamma
EPI_ISL_5800841	2021-04-22	RHD XV	Gamma
EPI_ISL_5800842	2021-04-22	RHD XV	Gamma
EPI_ISL_5800850	2021-04-22	RHD XV	Gamma
EPI_ISL_5800853	2021-04-22	RHD XV	Gamma
EPI_ISL_5800819	2021-04-22	RHD XV	Others
EPI_ISL_2444156	2021-04-26	RHD XV	Alpha
EPI_ISL_2444168	2021-04-26	RHD XV	Gamma
EPI_ISL_2444158	2021-04-26	RHD XV	Gamma
EPI_ISL_2444160	2021-04-26	RHD XV	Gamma
EPI_ISL_2444159	2021-04-26	RHD XV	Gamma
EPI_ISL_2444167	2021-04-26	RHD XV	Gamma
EPI_ISL_2444153	2021-04-26	RHD XV	Gamma
EPI_ISL_2444169	2021-04-26	RHD XV	Gamma
EPI_ISL_2444162	2021-04-26	RHD XV	Gamma
EPI_ISL_2444166	2021-04-26	RHD XV	Gamma
EPI_ISL_2444161	2021-04-26	RHD XV	Gamma
EPI_ISL_2444171	2021-04-26	RHD XV	Gamma
EPI_ISL_2444172	2021-04-26	RHD XV	Gamma
EPI_ISL_2444163	2021-04-26	RHD XV	Gamma
EPI_ISL_2444154	2021-04-26	RHD XV	Gamma
EPI_ISL_2444165	2021-04-26	RHD XV	Gamma
EPI_ISL_2444164	2021-04-26	RHD XV	Gamma
EPI_ISL_2444170	2021-04-26	RHD XV	Gamma
EPI_ISL_2444157	2021-04-26	RHD XV	Gamma
EPI_ISL_3048959	2021-04-26	RHD XV	Gamma
EPI_ISL_3048960	2021-04-26	RHD XV	Gamma
EPI_ISL_2444176	2021-04-27	RHD XV	Gamma
EPI_ISL_2444177	2021-04-27	RHD XV	Gamma
EPI_ISL_2444185	2021-04-27	RHD XV	Gamma
EPI_ISL_2444178	2021-04-27	RHD XV	Gamma
EPI_ISL_2444179	2021-04-27	RHD XV	Gamma
EPI_ISL_2444182	2021-04-27	RHD XV	Gamma

EPI_ISL_2444180	2021-04-27	RHD XV	Gamma
EPI_ISL_2444188	2021-04-27	RHD XV	Gamma
EPI_ISL_2455396	2021-04-27	RHD XV	Gamma
EPI_ISL_2444175	2021-04-27	RHD XV	Gamma
EPI_ISL_2444181	2021-04-27	RHD XV	Gamma
EPI_ISL_2444183	2021-04-27	RHD XV	Gamma
EPI_ISL_2444189	2021-04-27	RHD XV	Gamma
EPI_ISL_2444187	2021-04-27	RHD XV	Gamma
EPI_ISL_2444174	2021-04-27	RHD XV	Gamma
EPI_ISL_2008906	2021-04-27	RHD XV	Gamma
EPI_ISL_2008907	2021-04-27	RHD XV	Gamma
EPI_ISL_2008908	2021-04-27	RHD XV	Gamma
EPI_ISL_2008909	2021-04-27	RHD XV	Gamma
EPI_ISL_2008910	2021-04-27	RHD XV	Gamma
EPI_ISL_2008911	2021-04-27	RHD XV	Gamma
EPI_ISL_2008912	2021-04-27	RHD XV	Gamma
EPI_ISL_2008913	2021-04-27	RHD XV	Gamma
EPI_ISL_2008914	2021-04-27	RHD XV	Gamma
EPI_ISL_2008915	2021-04-27	RHD XV	Gamma
EPI_ISL_2008916	2021-04-27	RHD XV	Gamma
EPI_ISL_2008917	2021-04-27	RHD XV	Gamma
EPI_ISL_2008918	2021-04-27	RHD XV	Gamma
EPI_ISL_2008919	2021-04-27	RHD XV	Gamma
EPI_ISL_2008920	2021-04-27	RHD XV	Gamma
EPI_ISL_2008921	2021-04-27	RHD XV	Gamma
EPI_ISL_2008922	2021-04-27	RHD XV	Gamma
EPI_ISL_2008923	2021-04-27	RHD XV	Gamma
EPI_ISL_2008924	2021-04-27	RHD XV	Gamma
EPI_ISL_2008925	2021-04-27	RHD XV	Gamma
EPI_ISL_2008926	2021-04-27	RHD XV	Gamma
EPI_ISL_2008927	2021-04-27	RHD XV	Gamma
EPI_ISL_2008928	2021-04-27	RHD XV	Gamma
EPI_ISL_2107292	2021-04-27	RHD XV	Gamma
EPI_ISL_2008929	2021-04-27	RHD XV	Gamma
EPI_ISL_2107293	2021-04-27	RHD XV	Gamma
EPI_ISL_2107294	2021-04-27	RHD XV	Gamma
EPI_ISL_2008930	2021-04-27	RHD XV	Gamma
EPI_ISL_2008931	2021-04-27	RHD XV	Gamma
EPI_ISL_2107301	2021-04-27	RHD XV	Gamma
EPI_ISL_2107296	2021-04-27	RHD XV	Gamma
EPI_ISL_2008932	2021-04-27	RHD XV	Gamma
EPI_ISL_2107298	2021-04-27	RHD XV	Gamma
EPI_ISL_2107295	2021-04-27	RHD XV	Gamma
EPI_ISL_2008933	2021-04-27	RHD XV	Gamma
EPI_ISL_2008934	2021-04-27	RHD XV	Gamma
EPI_ISL_2107297	2021-04-27	RHD XV	Gamma
EPI_ISL_2008935	2021-04-27	RHD XV	Gamma
EPI_ISL_2444191	2021-04-28	RHD XV	Alpha
EPI_ISL_2444190	2021-04-28	RHD XV	Gamma
EPI_ISL_2444207	2021-05-02	RHD XV	Gamma
EPI_ISL_2444200	2021-05-02	RHD XV	Gamma
EPI_ISL_2444208	2021-05-02	RHD XV	Gamma
EPI_ISL_2444204	2021-05-02	RHD XV	Gamma
EPI_ISL_2444205	2021-05-02	RHD XV	Gamma
EPI_ISL_2444198	2021-05-02	RHD XV	Gamma
EPI_ISL_2444194	2021-05-02	RHD XV	Gamma
EPI_ISL_2444209	2021-05-02	RHD XV	Gamma
EPI_ISL_2444196	2021-05-02	RHD XV	Gamma
EPI_ISL_2444197	2021-05-02	RHD XV	Gamma
EPI_ISL_2455397	2021-05-02	RHD XV	Gamma
EPI_ISL_2444201	2021-05-02	RHD XV	Gamma
EPI_ISL_2444193	2021-05-02	RHD XV	Gamma
EPI_ISL_2444206	2021-05-02	RHD XV	Gamma

EPI_ISL_2444217	2021-05-03	RHD XV	Alpha
EPI_ISL_2444215	2021-05-03	RHD XV	Gamma
EPI_ISL_2444214	2021-05-03	RHD XV	Gamma
EPI_ISL_2444219	2021-05-03	RHD XV	Gamma
EPI_ISL_2444218	2021-05-03	RHD XV	Gamma
EPI_ISL_2444222	2021-05-03	RHD XV	Gamma
EPI_ISL_2444213	2021-05-03	RHD XV	Gamma
EPI_ISL_2444220	2021-05-03	RHD XV	Gamma
EPI_ISL_2444221	2021-05-03	RHD XV	Gamma
EPI_ISL_2444216	2021-05-03	RHD XV	Gamma
EPI_ISL_2444211	2021-05-03	RHD XV	Gamma
EPI_ISL_3048961	2021-05-03	RHD XV	Gamma
EPI_ISL_3761763	2021-05-03	RHD XV	Gamma
EPI_ISL_3048962	2021-05-03	RHD XV	Gamma
EPI_ISL_3048963	2021-05-04	RHD XV	Gamma
EPI_ISL_3048964	2021-05-05	RHD XV	Gamma
EPI_ISL_3048965	2021-05-05	RHD XV	Gamma
EPI_ISL_3048966	2021-05-06	RHD XV	Gamma
EPI_ISL_3761752	2021-05-06	RHD XV	Gamma
EPI_ISL_3048967	2021-05-06	RHD XV	Gamma
EPI_ISL_3761636	2021-05-06	RHD XV	Gamma
EPI_ISL_2445529	2021-05-10	RHD XV	Gamma
EPI_ISL_2445530	2021-05-10	RHD XV	Gamma
EPI_ISL_3048968	2021-05-10	RHD XV	Gamma
EPI_ISL_3048969	2021-05-10	RHD XV	Gamma
EPI_ISL_3048970	2021-05-10	RHD XV	Gamma
EPI_ISL_3048971	2021-05-10	RHD XV	Gamma
EPI_ISL_3761613	2021-05-10	RHD XV	Gamma
EPI_ISL_2445531	2021-05-11	RHD XV	Gamma
EPI_ISL_3761576	2021-05-11	RHD XV	Gamma
EPI_ISL_2445513	2021-05-12	RHD XV	Alpha
EPI_ISL_2445526	2021-05-12	RHD XV	Gamma
EPI_ISL_2445516	2021-05-12	RHD XV	Gamma
EPI_ISL_2445508	2021-05-12	RHD XV	Gamma
EPI_ISL_2445525	2021-05-12	RHD XV	Gamma
EPI_ISL_2445522	2021-05-12	RHD XV	Gamma
EPI_ISL_2473788	2021-05-12	RHD XV	Gamma
EPI_ISL_2445528	2021-05-12	RHD XV	Gamma
EPI_ISL_2445524	2021-05-12	RHD XV	Gamma
EPI_ISL_2473693	2021-05-12	RHD XV	Gamma
EPI_ISL_2445533	2021-05-12	RHD XV	Gamma
EPI_ISL_2445523	2021-05-12	RHD XV	Gamma
EPI_ISL_2445520	2021-05-12	RHD XV	Gamma
EPI_ISL_2445527	2021-05-12	RHD XV	Gamma
EPI_ISL_2445521	2021-05-12	RHD XV	Gamma
EPI_ISL_2445512	2021-05-12	RHD XV	Gamma
EPI_ISL_2473787	2021-05-12	RHD XV	Gamma
EPI_ISL_2445539	2021-05-12	RHD XV	Gamma
EPI_ISL_2445519	2021-05-12	RHD XV	Gamma
EPI_ISL_2445515	2021-05-12	RHD XV	Gamma
EPI_ISL_2445532	2021-05-12	RHD XV	Gamma
EPI_ISL_2473789	2021-05-12	RHD XV	Gamma
EPI_ISL_2445514	2021-05-12	RHD XV	Gamma
EPI_ISL_2445518	2021-05-12	RHD XV	Gamma
EPI_ISL_2445534	2021-05-12	RHD XV	Gamma
EPI_ISL_2473786	2021-05-12	RHD XV	Gamma
EPI_ISL_2473696	2021-05-12	RHD XV	Gamma
EPI_ISL_3761616	2021-05-12	RHD XV	Gamma
EPI_ISL_2473790	2021-05-13	RHD XV	Gamma
EPI_ISL_2473792	2021-05-13	RHD XV	Gamma
EPI_ISL_2473791	2021-05-13	RHD XV	Gamma
EPI_ISL_2445543	2021-05-13	RHD XV	Gamma
EPI_ISL_2445538	2021-05-13	RHD XV	Gamma

EPI_ISL_2445542	2021-05-13	RHD XV	Gamma
EPI_ISL_2445536	2021-05-13	RHD XV	Gamma
EPI_ISL_2445535	2021-05-13	RHD XV	Gamma
EPI_ISL_2445544	2021-05-13	RHD XV	Gamma
EPI_ISL_2445537	2021-05-13	RHD XV	Gamma
EPI_ISL_3048972	2021-05-13	RHD XV	Gamma
EPI_ISL_3761646	2021-05-15	RHD XV	Gamma
EPI_ISL_3048973	2021-05-17	RHD XV	Gamma
EPI_ISL_3048974	2021-05-17	RHD XV	Gamma
EPI_ISL_3048975	2021-05-17	RHD XV	Gamma
EPI_ISL_3048976	2021-05-18	RHD XV	Gamma
EPI_ISL_2473718	2021-05-19	RHD XV	Gamma
EPI_ISL_2473719	2021-05-19	RHD XV	Gamma
EPI_ISL_2445200	2021-05-19	RHD XV	Gamma
EPI_ISL_2473715	2021-05-19	RHD XV	Gamma
EPI_ISL_2445213	2021-05-19	RHD XV	Gamma
EPI_ISL_2445199	2021-05-19	RHD XV	Gamma
EPI_ISL_2445201	2021-05-19	RHD XV	Gamma
EPI_ISL_2445214	2021-05-19	RHD XV	Gamma
EPI_ISL_2473716	2021-05-19	RHD XV	Gamma
EPI_ISL_2445215	2021-05-19	RHD XV	Gamma
EPI_ISL_2445203	2021-05-19	RHD XV	Gamma
EPI_ISL_2445217	2021-05-19	RHD XV	Gamma
EPI_ISL_2445207	2021-05-19	RHD XV	Gamma
EPI_ISL_2473818	2021-05-19	RHD XV	Gamma
EPI_ISL_2473817	2021-05-19	RHD XV	Gamma
EPI_ISL_2445218	2021-05-19	RHD XV	Gamma
EPI_ISL_2473723	2021-05-19	RHD XV	Gamma
EPI_ISL_2445205	2021-05-19	RHD XV	Gamma
EPI_ISL_2445202	2021-05-19	RHD XV	Gamma
EPI_ISL_2473721	2021-05-19	RHD XV	Gamma
EPI_ISL_2445208	2021-05-19	RHD XV	Gamma
EPI_ISL_2445198	2021-05-19	RHD XV	Gamma
EPI_ISL_2445204	2021-05-19	RHD XV	Gamma
EPI_ISL_2445206	2021-05-19	RHD XV	Gamma
EPI_ISL_2473714	2021-05-19	RHD XV	Gamma
EPI_ISL_2473717	2021-05-19	RHD XV	Gamma
EPI_ISL_2473722	2021-05-19	RHD XV	Gamma
EPI_ISL_2500975	2021-05-19	RHD XV	Gamma
EPI_ISL_2493161	2021-05-19	RHD XV	Gamma
EPI_ISL_2493177	2021-05-19	RHD XV	Gamma
EPI_ISL_2494337	2021-05-19	RHD XV	Gamma
EPI_ISL_2493426	2021-05-19	RHD XV	Gamma
EPI_ISL_2493180	2021-05-19	RHD XV	Gamma
EPI_ISL_2493413	2021-05-19	RHD XV	Gamma
EPI_ISL_2493405	2021-05-19	RHD XV	Gamma
EPI_ISL_2493414	2021-05-19	RHD XV	Gamma
EPI_ISL_2493416	2021-05-19	RHD XV	Gamma
EPI_ISL_2493422	2021-05-19	RHD XV	Gamma
EPI_ISL_2493408	2021-05-19	RHD XV	Gamma
EPI_ISL_2493412	2021-05-19	RHD XV	Gamma
EPI_ISL_2493423	2021-05-19	RHD XV	Gamma
EPI_ISL_2493402	2021-05-19	RHD XV	Gamma
EPI_ISL_2493429	2021-05-19	RHD XV	Gamma
EPI_ISL_2493424	2021-05-19	RHD XV	Gamma
EPI_ISL_2493420	2021-05-19	RHD XV	Gamma
EPI_ISL_2493400	2021-05-19	RHD XV	Gamma
EPI_ISL_2493406	2021-05-19	RHD XV	Gamma
EPI_ISL_2493404	2021-05-19	RHD XV	Gamma
EPI_ISL_2493418	2021-05-19	RHD XV	Gamma
EPI_ISL_2493425	2021-05-19	RHD XV	Gamma
EPI_ISL_2493409	2021-05-19	RHD XV	Gamma
EPI_ISL_2493415	2021-05-19	RHD XV	Gamma

EPI_ISL_2493410	2021-05-19	RHD XV	Gamma
EPI_ISL_2493401	2021-05-19	RHD XV	Gamma
EPI_ISL_2493421	2021-05-19	RHD XV	Gamma
EPI_ISL_2493430	2021-05-19	RHD XV	Gamma
EPI_ISL_2493431	2021-05-19	RHD XV	Gamma
EPI_ISL_2493417	2021-05-19	RHD XV	Gamma
EPI_ISL_2493411	2021-05-19	RHD XV	Gamma
EPI_ISL_2493407	2021-05-19	RHD XV	Gamma
EPI_ISL_2493419	2021-05-19	RHD XV	Gamma
EPI_ISL_2493663	2021-05-19	RHD XV	Gamma
EPI_ISL_2493655	2021-05-19	RHD XV	Gamma
EPI_ISL_2493661	2021-05-19	RHD XV	Gamma
EPI_ISL_2493662	2021-05-19	RHD XV	Gamma
EPI_ISL_2493656	2021-05-19	RHD XV	Gamma
EPI_ISL_5645966	2021-05-19	RHD XV	Gamma
EPI_ISL_5645969	2021-05-19	RHD XV	Gamma
EPI_ISL_5645974	2021-05-19	RHD XV	Gamma
EPI_ISL_5645975	2021-05-19	RHD XV	Gamma
EPI_ISL_5645976	2021-05-19	RHD XV	Gamma
EPI_ISL_5645977	2021-05-19	RHD XV	Gamma
EPI_ISL_5645979	2021-05-19	RHD XV	Gamma
EPI_ISL_5645981	2021-05-19	RHD XV	Gamma
EPI_ISL_5645982	2021-05-19	RHD XV	Gamma
EPI_ISL_5645984	2021-05-19	RHD XV	Gamma
EPI_ISL_5645986	2021-05-19	RHD XV	Gamma
EPI_ISL_5645987	2021-05-19	RHD XV	Gamma
EPI_ISL_5645988	2021-05-19	RHD XV	Gamma
EPI_ISL_5645989	2021-05-19	RHD XV	Gamma
EPI_ISL_5645993	2021-05-19	RHD XV	Gamma
EPI_ISL_5645994	2021-05-19	RHD XV	Gamma
EPI_ISL_5645995	2021-05-19	RHD XV	Gamma
EPI_ISL_5645997	2021-05-19	RHD XV	Gamma
EPI_ISL_5645998	2021-05-19	RHD XV	Gamma
EPI_ISL_5646000	2021-05-19	RHD XV	Gamma
EPI_ISL_5646003	2021-05-19	RHD XV	Gamma
EPI_ISL_5646004	2021-05-19	RHD XV	Gamma
EPI_ISL_5646005	2021-05-19	RHD XV	Gamma
EPI_ISL_5646006	2021-05-19	RHD XV	Gamma
EPI_ISL_5646007	2021-05-19	RHD XV	Gamma
EPI_ISL_5646010	2021-05-19	RHD XV	Gamma
EPI_ISL_5646011	2021-05-19	RHD XV	Gamma
EPI_ISL_5646012	2021-05-19	RHD XV	Gamma
EPI_ISL_5646013	2021-05-19	RHD XV	Gamma
EPI_ISL_5646014	2021-05-19	RHD XV	Gamma
EPI_ISL_5646015	2021-05-19	RHD XV	Gamma
EPI_ISL_5646018	2021-05-19	RHD XV	Gamma
EPI_ISL_5646023	2021-05-19	RHD XV	Gamma
EPI_ISL_5646024	2021-05-19	RHD XV	Gamma
EPI_ISL_5646029	2021-05-19	RHD XV	Gamma
EPI_ISL_5646031	2021-05-19	RHD XV	Gamma
EPI_ISL_5646032	2021-05-19	RHD XV	Gamma
EPI_ISL_5646033	2021-05-19	RHD XV	Gamma
EPI_ISL_3048977	2021-05-19	RHD XV	Gamma
EPI_ISL_3761645	2021-05-19	RHD XV	Gamma
EPI_ISL_2378751	2021-05-19	RHD XV	Gamma
EPI_ISL_2473720	2021-05-20	RHD XV	Gamma
EPI_ISL_2445210	2021-05-20	RHD XV	Gamma
EPI_ISL_2445209	2021-05-20	RHD XV	Gamma
EPI_ISL_2445211	2021-05-20	RHD XV	Gamma
EPI_ISL_2445212	2021-05-20	RHD XV	Gamma
EPI_ISL_2445216	2021-05-20	RHD XV	Gamma
EPI_ISL_2493664	2021-05-20	RHD XV	Gamma
EPI_ISL_2493659	2021-05-20	RHD XV	Gamma

EPI_ISL_2494297	2021-05-20	RHD XV	Gamma
EPI_ISL_2493658	2021-05-20	RHD XV	Gamma
EPI_ISL_2493666	2021-05-20	RHD XV	Gamma
EPI_ISL_2493665	2021-05-20	RHD XV	Gamma
EPI_ISL_5646016	2021-05-20	RHD XV	Gamma
EPI_ISL_5646025	2021-05-20	RHD XV	Gamma
EPI_ISL_5646028	2021-05-20	RHD XV	Gamma
EPI_ISL_3048978	2021-05-20	RHD XV	Gamma
EPI_ISL_3761570	2021-05-20	RHD XV	Gamma
EPI_ISL_2378753	2021-05-20	RHD XV	Gamma
EPI_ISL_2378754	2021-05-20	RHD XV	Gamma
EPI_ISL_2378744	2021-05-20	RHD XV	Gamma
EPI_ISL_3048979	2021-05-24	RHD XV	Gamma
EPI_ISL_3048980	2021-05-25	RHD XV	Gamma
EPI_ISL_3761637	2021-05-25	RHD XV	Gamma
EPI_ISL_5649576	2021-05-26	RHD XV	Gamma
EPI_ISL_5649585	2021-05-26	RHD XV	Gamma
EPI_ISL_5650052	2021-05-26	RHD XV	Gamma
EPI_ISL_5649594	2021-05-26	RHD XV	Gamma
EPI_ISL_5649653	2021-05-26	RHD XV	Gamma
EPI_ISL_5649579	2021-05-26	RHD XV	Gamma
EPI_ISL_5649624	2021-05-26	RHD XV	Gamma
EPI_ISL_5649586	2021-05-26	RHD XV	Gamma
EPI_ISL_5650067	2021-05-26	RHD XV	Gamma
EPI_ISL_5649584	2021-05-26	RHD XV	Gamma
EPI_ISL_5649654	2021-05-26	RHD XV	Gamma
EPI_ISL_5649625	2021-05-26	RHD XV	Gamma
EPI_ISL_5650074	2021-05-26	RHD XV	Gamma
EPI_ISL_5650076	2021-05-26	RHD XV	Gamma
EPI_ISL_5650081	2021-05-26	RHD XV	Gamma
EPI_ISL_5649678	2021-05-26	RHD XV	Gamma
EPI_ISL_5649609	2021-05-26	RHD XV	Gamma
EPI_ISL_5649626	2021-05-26	RHD XV	Gamma
EPI_ISL_5649677	2021-05-26	RHD XV	Gamma
EPI_ISL_5649666	2021-05-26	RHD XV	Gamma
EPI_ISL_5649685	2021-05-26	RHD XV	Gamma
EPI_ISL_5649686	2021-05-26	RHD XV	Gamma
EPI_ISL_5649687	2021-05-26	RHD XV	Gamma
EPI_ISL_5650084	2021-05-26	RHD XV	Gamma
EPI_ISL_5650085	2021-05-26	RHD XV	Gamma
EPI_ISL_5649655	2021-05-26	RHD XV	Gamma
EPI_ISL_5649600	2021-05-26	RHD XV	Gamma
EPI_ISL_5650099	2021-05-26	RHD XV	Gamma
EPI_ISL_5650107	2021-05-26	RHD XV	Gamma
EPI_ISL_5650112	2021-05-26	RHD XV	Gamma
EPI_ISL_5649656	2021-05-26	RHD XV	Gamma
EPI_ISL_5649601	2021-05-26	RHD XV	Gamma
EPI_ISL_5650120	2021-05-26	RHD XV	Gamma
EPI_ISL_5650134	2021-05-26	RHD XV	Gamma
EPI_ISL_5650141	2021-05-26	RHD XV	Gamma
EPI_ISL_5650150	2021-05-26	RHD XV	Gamma
EPI_ISL_5650165	2021-05-26	RHD XV	Gamma
EPI_ISL_5650166	2021-05-26	RHD XV	Gamma
EPI_ISL_5650167	2021-05-26	RHD XV	Gamma
EPI_ISL_5650168	2021-05-26	RHD XV	Gamma
EPI_ISL_5650169	2021-05-26	RHD XV	Gamma
EPI_ISL_5650170	2021-05-26	RHD XV	Gamma
EPI_ISL_5650174	2021-05-26	RHD XV	Gamma
EPI_ISL_5650175	2021-05-26	RHD XV	Gamma
EPI_ISL_5650176	2021-05-26	RHD XV	Gamma
EPI_ISL_5649658	2021-05-26	RHD XV	Gamma
EPI_ISL_5650178	2021-05-26	RHD XV	Gamma
EPI_ISL_5650179	2021-05-26	RHD XV	Gamma

EPI_ISL_5650180	2021-05-26	RHD XV	Gamma
EPI_ISL_5650410	2021-05-26	RHD XV	Gamma
EPI_ISL_5650182	2021-05-26	RHD XV	Gamma
EPI_ISL_5650183	2021-05-26	RHD XV	Gamma
EPI_ISL_5650184	2021-05-26	RHD XV	Gamma
EPI_ISL_5650185	2021-05-26	RHD XV	Gamma
EPI_ISL_5649659	2021-05-26	RHD XV	Gamma
EPI_ISL_5649610	2021-05-26	RHD XV	Gamma
EPI_ISL_5650188	2021-05-26	RHD XV	Gamma
EPI_ISL_5650189	2021-05-26	RHD XV	Gamma
EPI_ISL_5649660	2021-05-26	RHD XV	Gamma
EPI_ISL_5650193	2021-05-26	RHD XV	Gamma
EPI_ISL_5650194	2021-05-26	RHD XV	Gamma
EPI_ISL_5650195	2021-05-26	RHD XV	Gamma
EPI_ISL_5650196	2021-05-26	RHD XV	Gamma
EPI_ISL_5650197	2021-05-26	RHD XV	Gamma
EPI_ISL_5650198	2021-05-26	RHD XV	Gamma
EPI_ISL_5650199	2021-05-26	RHD XV	Gamma
EPI_ISL_5650200	2021-05-26	RHD XV	Gamma
EPI_ISL_5649607	2021-05-26	RHD XV	Gamma
EPI_ISL_2494144	2021-05-26	RHD XV	Gamma
EPI_ISL_3761617	2021-05-26	RHD XV	Gamma
EPI_ISL_3761638	2021-05-26	RHD XV	Gamma
EPI_ISL_3761639	2021-05-26	RHD XV	Gamma
EPI_ISL_3761640	2021-05-26	RHD XV	Gamma
EPI_ISL_3761641	2021-05-26	RHD XV	Gamma
EPI_ISL_3761642	2021-05-26	RHD XV	Gamma
EPI_ISL_5649566	2021-05-27	RHD XV	Gamma
EPI_ISL_5649639	2021-05-27	RHD XV	Gamma
EPI_ISL_5649667	2021-05-27	RHD XV	Gamma
EPI_ISL_5649619	2021-05-27	RHD XV	Gamma
EPI_ISL_5650058	2021-05-27	RHD XV	Gamma
EPI_ISL_5649636	2021-05-27	RHD XV	Gamma
EPI_ISL_5649567	2021-05-27	RHD XV	Gamma
EPI_ISL_5649587	2021-05-27	RHD XV	Gamma
EPI_ISL_5649621	2021-05-27	RHD XV	Gamma
EPI_ISL_5650092	2021-05-27	RHD XV	Gamma
EPI_ISL_5649602	2021-05-27	RHD XV	Gamma
EPI_ISL_5650126	2021-05-27	RHD XV	Gamma
EPI_ISL_5649645	2021-05-27	RHD XV	Gamma
EPI_ISL_5649657	2021-05-27	RHD XV	Gamma
EPI_ISL_5650157	2021-05-27	RHD XV	Gamma
EPI_ISL_5650159	2021-05-27	RHD XV	Gamma
EPI_ISL_5650171	2021-05-27	RHD XV	Gamma
EPI_ISL_5650172	2021-05-27	RHD XV	Gamma
EPI_ISL_5650173	2021-05-27	RHD XV	Gamma
EPI_ISL_5650177	2021-05-27	RHD XV	Gamma
EPI_ISL_5650181	2021-05-27	RHD XV	Gamma
EPI_ISL_5649679	2021-05-27	RHD XV	Gamma
EPI_ISL_5650186	2021-05-27	RHD XV	Gamma
EPI_ISL_5650187	2021-05-27	RHD XV	Gamma
EPI_ISL_5650190	2021-05-27	RHD XV	Gamma
EPI_ISL_5650191	2021-05-27	RHD XV	Gamma
EPI_ISL_5650192	2021-05-27	RHD XV	Gamma
EPI_ISL_3761643	2021-05-27	RHD XV	Gamma
EPI_ISL_3761644	2021-05-27	RHD XV	Gamma
EPI_ISL_3048997	2021-05-30	RHD XV	Gamma
EPI_ISL_5650416	2021-05-31	RHD XV	Gamma
EPI_ISL_5650415	2021-05-31	RHD XV	Gamma
EPI_ISL_5649569	2021-05-31	RHD XV	Gamma
EPI_ISL_5649634	2021-05-31	RHD XV	Gamma
EPI_ISL_5649629	2021-05-31	RHD XV	Gamma
EPI_ISL_5649603	2021-05-31	RHD XV	Gamma

EPI_ISL_5649581	2021-05-31	RHD XV	Gamma
EPI_ISL_5650203	2021-05-31	RHD XV	Gamma
EPI_ISL_5649583	2021-05-31	RHD XV	Gamma
EPI_ISL_5649571	2021-05-31	RHD XV	Gamma
EPI_ISL_5650208	2021-05-31	RHD XV	Gamma
EPI_ISL_5649642	2021-05-31	RHD XV	Gamma
EPI_ISL_5649683	2021-05-31	RHD XV	Gamma
EPI_ISL_5650216	2021-05-31	RHD XV	Gamma
EPI_ISL_5650220	2021-05-31	RHD XV	Gamma
EPI_ISL_5650222	2021-05-31	RHD XV	Gamma
EPI_ISL_5650224	2021-05-31	RHD XV	Gamma
EPI_ISL_5650226	2021-05-31	RHD XV	Gamma
EPI_ISL_5650230	2021-05-31	RHD XV	Gamma
EPI_ISL_5650335	2021-05-31	RHD XV	Gamma
EPI_ISL_5650337	2021-05-31	RHD XV	Gamma
EPI_ISL_5649591	2021-05-31	RHD XV	Gamma
EPI_ISL_5650356	2021-05-31	RHD XV	Gamma
EPI_ISL_5650358	2021-05-31	RHD XV	Gamma
EPI_ISL_5650359	2021-05-31	RHD XV	Gamma
EPI_ISL_5650377	2021-05-31	RHD XV	Gamma
EPI_ISL_5645571	2021-05-31	RHD XV	Gamma
EPI_ISL_5645573	2021-05-31	RHD XV	Gamma
EPI_ISL_5645575	2021-05-31	RHD XV	Gamma
EPI_ISL_5645576	2021-05-31	RHD XV	Gamma
EPI_ISL_5646304	2021-05-31	RHD XV	Gamma
EPI_ISL_5646309	2021-05-31	RHD XV	Gamma
EPI_ISL_5646319	2021-05-31	RHD XV	Gamma
EPI_ISL_5646320	2021-05-31	RHD XV	Gamma
EPI_ISL_5646321	2021-05-31	RHD XV	Gamma
EPI_ISL_5646322	2021-05-31	RHD XV	Gamma
EPI_ISL_5646323	2021-05-31	RHD XV	Gamma
EPI_ISL_5646329	2021-05-31	RHD XV	Gamma
EPI_ISL_5646332	2021-05-31	RHD XV	Gamma
EPI_ISL_5646345	2021-05-31	RHD XV	Gamma
EPI_ISL_5646355	2021-05-31	RHD XV	Gamma
EPI_ISL_5646363	2021-05-31	RHD XV	Gamma
EPI_ISL_5646368	2021-05-31	RHD XV	Gamma
EPI_ISL_5646369	2021-05-31	RHD XV	Gamma
EPI_ISL_5646374	2021-05-31	RHD XV	Gamma
EPI_ISL_5646382	2021-05-31	RHD XV	Gamma
EPI_ISL_5646383	2021-05-31	RHD XV	Gamma
EPI_ISL_5647110	2021-05-31	RHD XV	Gamma
EPI_ISL_5647114	2021-05-31	RHD XV	Gamma
EPI_ISL_5647115	2021-05-31	RHD XV	Gamma
EPI_ISL_5647117	2021-05-31	RHD XV	Gamma
EPI_ISL_5647119	2021-05-31	RHD XV	Gamma
EPI_ISL_5647121	2021-05-31	RHD XV	Gamma
EPI_ISL_5647123	2021-05-31	RHD XV	Gamma
EPI_ISL_5647129	2021-05-31	RHD XV	Gamma
EPI_ISL_5647132	2021-05-31	RHD XV	Gamma
EPI_ISL_5647133	2021-05-31	RHD XV	Gamma
EPI_ISL_5647136	2021-05-31	RHD XV	Gamma
EPI_ISL_5647137	2021-05-31	RHD XV	Gamma
EPI_ISL_5647147	2021-05-31	RHD XV	Gamma
EPI_ISL_5647150	2021-05-31	RHD XV	Gamma
EPI_ISL_5647153	2021-05-31	RHD XV	Gamma
EPI_ISL_5647154	2021-05-31	RHD XV	Gamma
EPI_ISL_5647156	2021-05-31	RHD XV	Gamma
EPI_ISL_5647157	2021-05-31	RHD XV	Gamma
EPI_ISL_5647168	2021-05-31	RHD XV	Gamma
EPI_ISL_5647169	2021-05-31	RHD XV	Gamma
EPI_ISL_5647171	2021-05-31	RHD XV	Gamma
EPI_ISL_5647177	2021-05-31	RHD XV	Gamma

EPI_ISL_5647187	2021-05-31	RHD XV	Gamma
EPI_ISL_5647189	2021-05-31	RHD XV	Gamma
EPI_ISL_5647190	2021-05-31	RHD XV	Gamma
EPI_ISL_5647191	2021-05-31	RHD XV	Gamma
EPI_ISL_5647193	2021-05-31	RHD XV	Gamma
EPI_ISL_3048981	2021-05-31	RHD XV	Gamma
EPI_ISL_3048982	2021-05-31	RHD XV	Gamma
EPI_ISL_3048983	2021-05-31	RHD XV	Gamma
EPI_ISL_3048984	2021-05-31	RHD XV	Gamma
EPI_ISL_3048985	2021-05-31	RHD XV	Gamma
EPI_ISL_3048986	2021-05-31	RHD XV	Gamma
EPI_ISL_3048987	2021-05-31	RHD XV	Gamma
EPI_ISL_3048988	2021-05-31	RHD XV	Gamma
EPI_ISL_3048989	2021-05-31	RHD XV	Gamma
EPI_ISL_3048990	2021-05-31	RHD XV	Gamma
EPI_ISL_3048991	2021-05-31	RHD XV	Gamma
EPI_ISL_3048992	2021-05-31	RHD XV	Gamma
EPI_ISL_3048993	2021-05-31	RHD XV	Gamma
EPI_ISL_3048994	2021-05-31	RHD XV	Gamma
EPI_ISL_3048995	2021-05-31	RHD XV	Gamma
EPI_ISL_3048996	2021-05-31	RHD XV	Gamma
EPI_ISL_3761760	2021-06	RHD XV	Gamma
EPI_ISL_5650333	2021-06-01	RHD XV	Gamma
EPI_ISL_5650334	2021-06-01	RHD XV	Gamma
EPI_ISL_5650341	2021-06-01	RHD XV	Gamma
EPI_ISL_5650344	2021-06-01	RHD XV	Gamma
EPI_ISL_5649589	2021-06-01	RHD XV	Gamma
EPI_ISL_5650352	2021-06-01	RHD XV	Gamma
EPI_ISL_5650353	2021-06-01	RHD XV	Gamma
EPI_ISL_5650357	2021-06-01	RHD XV	Gamma
EPI_ISL_5649592	2021-06-01	RHD XV	Gamma
EPI_ISL_5650361	2021-06-01	RHD XV	Gamma
EPI_ISL_5650371	2021-06-01	RHD XV	Gamma
EPI_ISL_5650374	2021-06-01	RHD XV	Gamma
EPI_ISL_5650381	2021-06-01	RHD XV	Gamma
EPI_ISL_5650385	2021-06-01	RHD XV	Gamma
EPI_ISL_5650386	2021-06-01	RHD XV	Gamma
EPI_ISL_5645574	2021-06-01	RHD XV	Gamma
EPI_ISL_5645577	2021-06-01	RHD XV	Gamma
EPI_ISL_5645579	2021-06-01	RHD XV	Gamma
EPI_ISL_5647113	2021-06-01	RHD XV	Gamma
EPI_ISL_5647124	2021-06-01	RHD XV	Gamma
EPI_ISL_5647127	2021-06-01	RHD XV	Gamma
EPI_ISL_5647158	2021-06-01	RHD XV	Gamma
EPI_ISL_5647173	2021-06-01	RHD XV	Gamma
EPI_ISL_5647197	2021-06-01	RHD XV	Gamma
EPI_ISL_5646979	2021-06-06	RHD XV	Gamma
EPI_ISL_5646982	2021-06-06	RHD XV	Gamma
EPI_ISL_5646987	2021-06-06	RHD XV	Gamma
EPI_ISL_5646995	2021-06-06	RHD XV	Gamma
EPI_ISL_5647002	2021-06-06	RHD XV	Gamma
EPI_ISL_5647003	2021-06-06	RHD XV	Gamma
EPI_ISL_5647216	2021-06-06	RHD XV	Gamma
EPI_ISL_5647228	2021-06-06	RHD XV	Gamma
EPI_ISL_5647233	2021-06-06	RHD XV	Gamma
EPI_ISL_5647240	2021-06-06	RHD XV	Gamma
EPI_ISL_5647243	2021-06-06	RHD XV	Gamma
EPI_ISL_5647249	2021-06-06	RHD XV	Gamma
EPI_ISL_5647204	2021-06-07	RHD XV	Alpha
EPI_ISL_5647205	2021-06-07	RHD XV	Alpha
EPI_ISL_5646871	2021-06-07	RHD XV	Gamma
EPI_ISL_5646875	2021-06-07	RHD XV	Gamma
EPI_ISL_5646877	2021-06-07	RHD XV	Gamma

EPI_ISL_5646881	2021-06-07	RHD XV	Gamma
EPI_ISL_5646882	2021-06-07	RHD XV	Gamma
EPI_ISL_5646884	2021-06-07	RHD XV	Gamma
EPI_ISL_5646885	2021-06-07	RHD XV	Gamma
EPI_ISL_5646888	2021-06-07	RHD XV	Gamma
EPI_ISL_5646889	2021-06-07	RHD XV	Gamma
EPI_ISL_5646897	2021-06-07	RHD XV	Gamma
EPI_ISL_5646901	2021-06-07	RHD XV	Gamma
EPI_ISL_5646902	2021-06-07	RHD XV	Gamma
EPI_ISL_5646910	2021-06-07	RHD XV	Gamma
EPI_ISL_5646916	2021-06-07	RHD XV	Gamma
EPI_ISL_5646917	2021-06-07	RHD XV	Gamma
EPI_ISL_5646921	2021-06-07	RHD XV	Gamma
EPI_ISL_5646931	2021-06-07	RHD XV	Gamma
EPI_ISL_5646932	2021-06-07	RHD XV	Gamma
EPI_ISL_5646933	2021-06-07	RHD XV	Gamma
EPI_ISL_5646943	2021-06-07	RHD XV	Gamma
EPI_ISL_5646962	2021-06-07	RHD XV	Gamma
EPI_ISL_5646963	2021-06-07	RHD XV	Gamma
EPI_ISL_5646967	2021-06-07	RHD XV	Gamma
EPI_ISL_5646968	2021-06-07	RHD XV	Gamma
EPI_ISL_5646976	2021-06-07	RHD XV	Gamma
EPI_ISL_5646977	2021-06-07	RHD XV	Gamma
EPI_ISL_5646983	2021-06-07	RHD XV	Gamma
EPI_ISL_5646986	2021-06-07	RHD XV	Gamma
EPI_ISL_5646993	2021-06-07	RHD XV	Gamma
EPI_ISL_5647209	2021-06-07	RHD XV	Gamma
EPI_ISL_5647212	2021-06-07	RHD XV	Gamma
EPI_ISL_5647213	2021-06-07	RHD XV	Gamma
EPI_ISL_5647219	2021-06-07	RHD XV	Gamma
EPI_ISL_5647220	2021-06-07	RHD XV	Gamma
EPI_ISL_5647227	2021-06-07	RHD XV	Gamma
EPI_ISL_5647229	2021-06-07	RHD XV	Gamma
EPI_ISL_5647236	2021-06-07	RHD XV	Gamma
EPI_ISL_5647241	2021-06-07	RHD XV	Gamma
EPI_ISL_5647244	2021-06-07	RHD XV	Gamma
EPI_ISL_5647245	2021-06-07	RHD XV	Gamma
EPI_ISL_5647247	2021-06-07	RHD XV	Gamma
EPI_ISL_5647251	2021-06-07	RHD XV	Gamma
EPI_ISL_5647254	2021-06-07	RHD XV	Gamma
EPI_ISL_5647259	2021-06-07	RHD XV	Gamma
EPI_ISL_5647260	2021-06-07	RHD XV	Gamma
EPI_ISL_5647261	2021-06-07	RHD XV	Gamma
EPI_ISL_5647263	2021-06-07	RHD XV	Gamma
EPI_ISL_5647266	2021-06-07	RHD XV	Gamma
EPI_ISL_5647268	2021-06-07	RHD XV	Gamma
EPI_ISL_5647272	2021-06-07	RHD XV	Gamma
EPI_ISL_5647292	2021-06-07	RHD XV	Gamma
EPI_ISL_5647294	2021-06-07	RHD XV	Gamma
EPI_ISL_5646876	2021-06-08	RHD XV	Gamma
EPI_ISL_5646880	2021-06-08	RHD XV	Gamma
EPI_ISL_5646894	2021-06-08	RHD XV	Gamma
EPI_ISL_5646896	2021-06-08	RHD XV	Gamma
EPI_ISL_5646907	2021-06-08	RHD XV	Gamma
EPI_ISL_5646913	2021-06-08	RHD XV	Gamma
EPI_ISL_5646914	2021-06-08	RHD XV	Gamma
EPI_ISL_5646915	2021-06-08	RHD XV	Gamma
EPI_ISL_5646930	2021-06-08	RHD XV	Gamma
EPI_ISL_5647210	2021-06-08	RHD XV	Gamma
EPI_ISL_5647214	2021-06-08	RHD XV	Gamma
EPI_ISL_5647257	2021-06-08	RHD XV	Gamma
EPI_ISL_5647262	2021-06-08	RHD XV	Gamma
EPI_ISL_5647267	2021-06-08	RHD XV	Gamma

EPI_ISL_5647271	2021-06-08	RHD XV	Gamma
EPI_ISL_5647275	2021-06-08	RHD XV	Gamma
EPI_ISL_3048998	2021-06-09	RHD XV	Gamma
EPI_ISL_3048999	2021-06-09	RHD XV	Gamma
EPI_ISL_3049000	2021-06-09	RHD XV	Gamma
EPI_ISL_3761761	2021-06-12	RHD XV	Gamma
EPI_ISL_5648047	2021-06-13	RHD XV	Gamma
EPI_ISL_5648129	2021-06-13	RHD XV	Gamma
EPI_ISL_5647663	2021-06-14	RHD XV	Gamma
EPI_ISL_5647689	2021-06-14	RHD XV	Gamma
EPI_ISL_5647698	2021-06-14	RHD XV	Gamma
EPI_ISL_5647715	2021-06-14	RHD XV	Gamma
EPI_ISL_5647733	2021-06-14	RHD XV	Gamma
EPI_ISL_5647736	2021-06-14	RHD XV	Gamma
EPI_ISL_5647754	2021-06-14	RHD XV	Gamma
EPI_ISL_5647757	2021-06-14	RHD XV	Gamma
EPI_ISL_5647759	2021-06-14	RHD XV	Gamma
EPI_ISL_5647762	2021-06-14	RHD XV	Gamma
EPI_ISL_5647776	2021-06-14	RHD XV	Gamma
EPI_ISL_5647779	2021-06-14	RHD XV	Gamma
EPI_ISL_5647781	2021-06-14	RHD XV	Gamma
EPI_ISL_5647786	2021-06-14	RHD XV	Gamma
EPI_ISL_5647787	2021-06-14	RHD XV	Gamma
EPI_ISL_5647790	2021-06-14	RHD XV	Gamma
EPI_ISL_5647792	2021-06-14	RHD XV	Gamma
EPI_ISL_5647793	2021-06-14	RHD XV	Gamma
EPI_ISL_5647800	2021-06-14	RHD XV	Gamma
EPI_ISL_5647803	2021-06-14	RHD XV	Gamma
EPI_ISL_5647811	2021-06-14	RHD XV	Gamma
EPI_ISL_5647812	2021-06-14	RHD XV	Gamma
EPI_ISL_5647813	2021-06-14	RHD XV	Gamma
EPI_ISL_5647831	2021-06-14	RHD XV	Gamma
EPI_ISL_5647832	2021-06-14	RHD XV	Gamma
EPI_ISL_5647835	2021-06-14	RHD XV	Gamma
EPI_ISL_5647836	2021-06-14	RHD XV	Gamma
EPI_ISL_5647848	2021-06-14	RHD XV	Gamma
EPI_ISL_5647850	2021-06-14	RHD XV	Gamma
EPI_ISL_5647852	2021-06-14	RHD XV	Gamma
EPI_ISL_5647859	2021-06-14	RHD XV	Gamma
EPI_ISL_5647860	2021-06-14	RHD XV	Gamma
EPI_ISL_5647958	2021-06-14	RHD XV	Gamma
EPI_ISL_5647962	2021-06-14	RHD XV	Gamma
EPI_ISL_5647968	2021-06-14	RHD XV	Gamma
EPI_ISL_5647979	2021-06-14	RHD XV	Gamma
EPI_ISL_5647983	2021-06-14	RHD XV	Gamma
EPI_ISL_5647999	2021-06-14	RHD XV	Gamma
EPI_ISL_5648011	2021-06-14	RHD XV	Gamma
EPI_ISL_5648032	2021-06-14	RHD XV	Gamma
EPI_ISL_5648033	2021-06-14	RHD XV	Gamma
EPI_ISL_5648038	2021-06-14	RHD XV	Gamma
EPI_ISL_5648044	2021-06-14	RHD XV	Gamma
EPI_ISL_5648050	2021-06-14	RHD XV	Gamma
EPI_ISL_5648131	2021-06-14	RHD XV	Gamma
EPI_ISL_5648138	2021-06-14	RHD XV	Gamma
EPI_ISL_5648140	2021-06-14	RHD XV	Gamma
EPI_ISL_5648141	2021-06-14	RHD XV	Gamma
EPI_ISL_5648144	2021-06-14	RHD XV	Gamma
EPI_ISL_5648145	2021-06-14	RHD XV	Gamma
EPI_ISL_5648146	2021-06-14	RHD XV	Gamma
EPI_ISL_5648147	2021-06-14	RHD XV	Gamma
EPI_ISL_5648149	2021-06-14	RHD XV	Gamma
EPI_ISL_5648150	2021-06-14	RHD XV	Gamma
EPI_ISL_5648151	2021-06-14	RHD XV	Gamma

EPI_ISL_5648153	2021-06-14	RHD XV	Gamma
EPI_ISL_5648155	2021-06-14	RHD XV	Gamma
EPI_ISL_5648156	2021-06-14	RHD XV	Gamma
EPI_ISL_5648157	2021-06-14	RHD XV	Gamma
EPI_ISL_5648158	2021-06-14	RHD XV	Gamma
EPI_ISL_5648159	2021-06-14	RHD XV	Gamma
EPI_ISL_5648171	2021-06-14	RHD XV	Gamma
EPI_ISL_5648173	2021-06-14	RHD XV	Gamma
EPI_ISL_5648175	2021-06-14	RHD XV	Gamma
EPI_ISL_5648180	2021-06-14	RHD XV	Gamma
EPI_ISL_5648184	2021-06-14	RHD XV	Gamma
EPI_ISL_5648185	2021-06-14	RHD XV	Gamma
EPI_ISL_5648188	2021-06-14	RHD XV	Gamma
EPI_ISL_5648195	2021-06-14	RHD XV	Gamma
EPI_ISL_5648196	2021-06-14	RHD XV	Gamma
EPI_ISL_5648197	2021-06-14	RHD XV	Gamma
EPI_ISL_5648198	2021-06-14	RHD XV	Gamma
EPI_ISL_5648200	2021-06-14	RHD XV	Gamma
EPI_ISL_5648202	2021-06-14	RHD XV	Gamma
EPI_ISL_5648206	2021-06-14	RHD XV	Gamma
EPI_ISL_5648207	2021-06-14	RHD XV	Gamma
EPI_ISL_5648208	2021-06-14	RHD XV	Gamma
EPI_ISL_5648209	2021-06-14	RHD XV	Gamma
EPI_ISL_5648212	2021-06-14	RHD XV	Gamma
EPI_ISL_5648214	2021-06-14	RHD XV	Gamma
EPI_ISL_5648215	2021-06-14	RHD XV	Gamma
EPI_ISL_5648216	2021-06-14	RHD XV	Gamma
EPI_ISL_5648217	2021-06-14	RHD XV	Gamma
EPI_ISL_5648218	2021-06-14	RHD XV	Gamma
EPI_ISL_5648219	2021-06-14	RHD XV	Gamma
EPI_ISL_5648220	2021-06-14	RHD XV	Gamma
EPI_ISL_5648221	2021-06-14	RHD XV	Gamma
EPI_ISL_5648223	2021-06-14	RHD XV	Gamma
EPI_ISL_3055544	2021-06-14	RHD XV	Gamma
EPI_ISL_5647657	2021-06-14	RHD XV	Others
EPI_ISL_5647676	2021-06-15	RHD XV	Gamma
EPI_ISL_5647687	2021-06-15	RHD XV	Gamma
EPI_ISL_5647697	2021-06-15	RHD XV	Gamma
EPI_ISL_5647704	2021-06-15	RHD XV	Gamma
EPI_ISL_5647710	2021-06-15	RHD XV	Gamma
EPI_ISL_5647716	2021-06-15	RHD XV	Gamma
EPI_ISL_5647717	2021-06-15	RHD XV	Gamma
EPI_ISL_5647721	2021-06-15	RHD XV	Gamma
EPI_ISL_5647724	2021-06-15	RHD XV	Gamma
EPI_ISL_5647729	2021-06-15	RHD XV	Gamma
EPI_ISL_5647731	2021-06-15	RHD XV	Gamma
EPI_ISL_5647760	2021-06-15	RHD XV	Gamma
EPI_ISL_5647761	2021-06-15	RHD XV	Gamma
EPI_ISL_5647774	2021-06-15	RHD XV	Gamma
EPI_ISL_5647778	2021-06-15	RHD XV	Gamma
EPI_ISL_5647791	2021-06-15	RHD XV	Gamma
EPI_ISL_5647807	2021-06-15	RHD XV	Gamma
EPI_ISL_5647820	2021-06-15	RHD XV	Gamma
EPI_ISL_5647843	2021-06-15	RHD XV	Gamma
EPI_ISL_5648134	2021-06-15	RHD XV	Gamma
EPI_ISL_5648135	2021-06-15	RHD XV	Gamma
EPI_ISL_5648139	2021-06-15	RHD XV	Gamma
EPI_ISL_5648174	2021-06-15	RHD XV	Gamma
EPI_ISL_5648177	2021-06-15	RHD XV	Gamma
EPI_ISL_5648210	2021-06-15	RHD XV	Gamma
EPI_ISL_5648211	2021-06-15	RHD XV	Gamma
EPI_ISL_3049002	2021-06-15	RHD XV	Gamma
EPI_ISL_3049003	2021-06-15	RHD XV	Gamma

EPI_ISL_3049004	2021-06-15	RHD XV	Gamma
EPI_ISL_3049005	2021-06-15	RHD XV	Gamma
EPI_ISL_3049006	2021-06-16	RHD XV	Gamma
EPI_ISL_3049007	2021-06-16	RHD XV	Gamma
EPI_ISL_3761753	2021-06-16	RHD XV	Gamma
EPI_ISL_3049008	2021-06-16	RHD XV	Gamma
EPI_ISL_3055542	2021-06-16	RHD XV	Gamma
EPI_ISL_3049009	2021-06-16	RHD XV	Gamma
EPI_ISL_3049010	2021-06-16	RHD XV	Gamma
EPI_ISL_3049011	2021-06-17	RHD XV	Gamma
EPI_ISL_3049012	2021-06-17	RHD XV	Gamma
EPI_ISL_5659563	2021-06-20	RHD XV	Gamma
EPI_ISL_5659593	2021-06-20	RHD XV	Gamma
EPI_ISL_5648266	2021-06-21	RHD XV	Gamma
EPI_ISL_5648278	2021-06-21	RHD XV	Gamma
EPI_ISL_5648305	2021-06-21	RHD XV	Gamma
EPI_ISL_5648388	2021-06-21	RHD XV	Gamma
EPI_ISL_5648434	2021-06-21	RHD XV	Gamma
EPI_ISL_5648437	2021-06-21	RHD XV	Gamma
EPI_ISL_5648461	2021-06-21	RHD XV	Gamma
EPI_ISL_5648488	2021-06-21	RHD XV	Gamma
EPI_ISL_5648498	2021-06-21	RHD XV	Gamma
EPI_ISL_5648505	2021-06-21	RHD XV	Gamma
EPI_ISL_5648508	2021-06-21	RHD XV	Gamma
EPI_ISL_5648572	2021-06-21	RHD XV	Gamma
EPI_ISL_5648580	2021-06-21	RHD XV	Gamma
EPI_ISL_5648581	2021-06-21	RHD XV	Gamma
EPI_ISL_5648588	2021-06-21	RHD XV	Gamma
EPI_ISL_5648592	2021-06-21	RHD XV	Gamma
EPI_ISL_5648595	2021-06-21	RHD XV	Gamma
EPI_ISL_5648598	2021-06-21	RHD XV	Gamma
EPI_ISL_5648606	2021-06-21	RHD XV	Gamma
EPI_ISL_5648612	2021-06-21	RHD XV	Gamma
EPI_ISL_5648655	2021-06-21	RHD XV	Gamma
EPI_ISL_5648663	2021-06-21	RHD XV	Gamma
EPI_ISL_5648691	2021-06-21	RHD XV	Gamma
EPI_ISL_5648699	2021-06-21	RHD XV	Gamma
EPI_ISL_5648728	2021-06-21	RHD XV	Gamma
EPI_ISL_5648732	2021-06-21	RHD XV	Gamma
EPI_ISL_5648733	2021-06-21	RHD XV	Gamma
EPI_ISL_5648741	2021-06-21	RHD XV	Gamma
EPI_ISL_5648745	2021-06-21	RHD XV	Gamma
EPI_ISL_5648746	2021-06-21	RHD XV	Gamma
EPI_ISL_5648750	2021-06-21	RHD XV	Gamma
EPI_ISL_5648752	2021-06-21	RHD XV	Gamma
EPI_ISL_5648767	2021-06-21	RHD XV	Gamma
EPI_ISL_5648772	2021-06-21	RHD XV	Gamma
EPI_ISL_5648779	2021-06-21	RHD XV	Gamma
EPI_ISL_5648800	2021-06-21	RHD XV	Gamma
EPI_ISL_5648809	2021-06-21	RHD XV	Gamma
EPI_ISL_5648861	2021-06-21	RHD XV	Gamma
EPI_ISL_5648872	2021-06-21	RHD XV	Gamma
EPI_ISL_5648907	2021-06-21	RHD XV	Gamma
EPI_ISL_5648938	2021-06-21	RHD XV	Gamma
EPI_ISL_5648943	2021-06-21	RHD XV	Gamma
EPI_ISL_5648971	2021-06-21	RHD XV	Gamma
EPI_ISL_5648972	2021-06-21	RHD XV	Gamma
EPI_ISL_5648978	2021-06-21	RHD XV	Gamma
EPI_ISL_5648980	2021-06-21	RHD XV	Gamma
EPI_ISL_5649005	2021-06-21	RHD XV	Gamma
EPI_ISL_5649015	2021-06-21	RHD XV	Gamma
EPI_ISL_5649026	2021-06-21	RHD XV	Gamma
EPI_ISL_5649031	2021-06-21	RHD XV	Gamma

EPI_ISL_5649038	2021-06-21	RHD XV	Gamma
EPI_ISL_5649039	2021-06-21	RHD XV	Gamma
EPI_ISL_5649051	2021-06-21	RHD XV	Gamma
EPI_ISL_5649052	2021-06-21	RHD XV	Gamma
EPI_ISL_5649083	2021-06-21	RHD XV	Gamma
EPI_ISL_5649136	2021-06-21	RHD XV	Gamma
EPI_ISL_5649139	2021-06-21	RHD XV	Gamma
EPI_ISL_5649433	2021-06-21	RHD XV	Gamma
EPI_ISL_5649445	2021-06-21	RHD XV	Gamma
EPI_ISL_5649457	2021-06-21	RHD XV	Gamma
EPI_ISL_5649464	2021-06-21	RHD XV	Gamma
EPI_ISL_5649465	2021-06-21	RHD XV	Gamma
EPI_ISL_5649484	2021-06-21	RHD XV	Gamma
EPI_ISL_5659520	2021-06-21	RHD XV	Gamma
EPI_ISL_5659533	2021-06-21	RHD XV	Gamma
EPI_ISL_5659536	2021-06-21	RHD XV	Gamma
EPI_ISL_5659537	2021-06-21	RHD XV	Gamma
EPI_ISL_5659538	2021-06-21	RHD XV	Gamma
EPI_ISL_5659539	2021-06-21	RHD XV	Gamma
EPI_ISL_5659541	2021-06-21	RHD XV	Gamma
EPI_ISL_5659542	2021-06-21	RHD XV	Gamma
EPI_ISL_5659543	2021-06-21	RHD XV	Gamma
EPI_ISL_5659544	2021-06-21	RHD XV	Gamma
EPI_ISL_5659545	2021-06-21	RHD XV	Gamma
EPI_ISL_5659546	2021-06-21	RHD XV	Gamma
EPI_ISL_5659547	2021-06-21	RHD XV	Gamma
EPI_ISL_5659548	2021-06-21	RHD XV	Gamma
EPI_ISL_5659549	2021-06-21	RHD XV	Gamma
EPI_ISL_5659550	2021-06-21	RHD XV	Gamma
EPI_ISL_5659551	2021-06-21	RHD XV	Gamma
EPI_ISL_5659554	2021-06-21	RHD XV	Gamma
EPI_ISL_5659555	2021-06-21	RHD XV	Gamma
EPI_ISL_5659556	2021-06-21	RHD XV	Gamma
EPI_ISL_5659557	2021-06-21	RHD XV	Gamma
EPI_ISL_5659558	2021-06-21	RHD XV	Gamma
EPI_ISL_5659559	2021-06-21	RHD XV	Gamma
EPI_ISL_5659560	2021-06-21	RHD XV	Gamma
EPI_ISL_5659561	2021-06-21	RHD XV	Gamma
EPI_ISL_5659562	2021-06-21	RHD XV	Gamma
EPI_ISL_5659564	2021-06-21	RHD XV	Gamma
EPI_ISL_5659565	2021-06-21	RHD XV	Gamma
EPI_ISL_5659567	2021-06-21	RHD XV	Gamma
EPI_ISL_5659568	2021-06-21	RHD XV	Gamma
EPI_ISL_5659569	2021-06-21	RHD XV	Gamma
EPI_ISL_5659570	2021-06-21	RHD XV	Gamma
EPI_ISL_5659571	2021-06-21	RHD XV	Gamma
EPI_ISL_5659573	2021-06-21	RHD XV	Gamma
EPI_ISL_5659574	2021-06-21	RHD XV	Gamma
EPI_ISL_5659575	2021-06-21	RHD XV	Gamma
EPI_ISL_5659577	2021-06-21	RHD XV	Gamma
EPI_ISL_5659579	2021-06-21	RHD XV	Gamma
EPI_ISL_5659580	2021-06-21	RHD XV	Gamma
EPI_ISL_5659582	2021-06-21	RHD XV	Gamma
EPI_ISL_5659583	2021-06-21	RHD XV	Gamma
EPI_ISL_5659584	2021-06-21	RHD XV	Gamma
EPI_ISL_5659585	2021-06-21	RHD XV	Gamma
EPI_ISL_5659587	2021-06-21	RHD XV	Gamma
EPI_ISL_5659588	2021-06-21	RHD XV	Gamma
EPI_ISL_5659589	2021-06-21	RHD XV	Gamma
EPI_ISL_5659590	2021-06-21	RHD XV	Gamma
EPI_ISL_5659591	2021-06-21	RHD XV	Gamma
EPI_ISL_5659592	2021-06-21	RHD XV	Gamma
EPI_ISL_5659595	2021-06-21	RHD XV	Gamma

EPI_ISL_5659596	2021-06-21	RHD XV	Gamma
EPI_ISL_5659597	2021-06-21	RHD XV	Gamma
EPI_ISL_5659600	2021-06-21	RHD XV	Gamma
EPI_ISL_5659601	2021-06-21	RHD XV	Gamma
EPI_ISL_5659603	2021-06-21	RHD XV	Gamma
EPI_ISL_5659604	2021-06-21	RHD XV	Gamma
EPI_ISL_5659605	2021-06-21	RHD XV	Gamma
EPI_ISL_5659607	2021-06-21	RHD XV	Gamma
EPI_ISL_5659609	2021-06-21	RHD XV	Gamma
EPI_ISL_5659610	2021-06-21	RHD XV	Gamma
EPI_ISL_5659611	2021-06-21	RHD XV	Gamma
EPI_ISL_5659612	2021-06-21	RHD XV	Gamma
EPI_ISL_5659616	2021-06-21	RHD XV	Gamma
EPI_ISL_5659617	2021-06-21	RHD XV	Gamma
EPI_ISL_5659618	2021-06-21	RHD XV	Gamma
EPI_ISL_5659619	2021-06-21	RHD XV	Gamma
EPI_ISL_5659620	2021-06-21	RHD XV	Gamma
EPI_ISL_5659621	2021-06-21	RHD XV	Gamma
EPI_ISL_5659622	2021-06-21	RHD XV	Gamma
EPI_ISL_5659623	2021-06-21	RHD XV	Gamma
EPI_ISL_5659624	2021-06-21	RHD XV	Gamma
EPI_ISL_5659625	2021-06-21	RHD XV	Gamma
EPI_ISL_5659627	2021-06-21	RHD XV	Gamma
EPI_ISL_5659628	2021-06-21	RHD XV	Gamma
EPI_ISL_5659629	2021-06-21	RHD XV	Gamma
EPI_ISL_5659630	2021-06-21	RHD XV	Gamma
EPI_ISL_5659631	2021-06-21	RHD XV	Gamma
EPI_ISL_5659632	2021-06-21	RHD XV	Gamma
EPI_ISL_5659633	2021-06-21	RHD XV	Gamma
EPI_ISL_5659634	2021-06-21	RHD XV	Gamma
EPI_ISL_5659635	2021-06-21	RHD XV	Gamma
EPI_ISL_5659636	2021-06-21	RHD XV	Gamma
EPI_ISL_5659637	2021-06-21	RHD XV	Gamma
EPI_ISL_5659638	2021-06-21	RHD XV	Gamma
EPI_ISL_5659640	2021-06-21	RHD XV	Gamma
EPI_ISL_3049013	2021-06-21	RHD XV	Gamma
EPI_ISL_3049014	2021-06-21	RHD XV	Gamma
EPI_ISL_3049015	2021-06-21	RHD XV	Gamma
EPI_ISL_3049016	2021-06-21	RHD XV	Gamma
EPI_ISL_3049017	2021-06-21	RHD XV	Gamma
EPI_ISL_3049018	2021-06-21	RHD XV	Gamma
EPI_ISL_3049019	2021-06-21	RHD XV	Gamma
EPI_ISL_3049020	2021-06-21	RHD XV	Gamma
EPI_ISL_3761647	2021-06-21	RHD XV	Gamma
EPI_ISL_3055529	2021-06-21	RHD XV	Gamma
EPI_ISL_3761648	2021-06-21	RHD XV	Gamma
EPI_ISL_3761649	2021-06-21	RHD XV	Gamma
EPI_ISL_5648381	2021-06-22	RHD XV	Gamma
EPI_ISL_5648438	2021-06-22	RHD XV	Gamma
EPI_ISL_5648449	2021-06-22	RHD XV	Gamma
EPI_ISL_5648484	2021-06-22	RHD XV	Gamma
EPI_ISL_5648503	2021-06-22	RHD XV	Gamma
EPI_ISL_5648546	2021-06-22	RHD XV	Gamma
EPI_ISL_5648570	2021-06-22	RHD XV	Gamma
EPI_ISL_5648623	2021-06-22	RHD XV	Gamma
EPI_ISL_5648624	2021-06-22	RHD XV	Gamma
EPI_ISL_5648670	2021-06-22	RHD XV	Gamma
EPI_ISL_5648722	2021-06-22	RHD XV	Gamma
EPI_ISL_5648776	2021-06-22	RHD XV	Gamma
EPI_ISL_5648806	2021-06-22	RHD XV	Gamma
EPI_ISL_5648837	2021-06-22	RHD XV	Gamma
EPI_ISL_5648839	2021-06-22	RHD XV	Gamma
EPI_ISL_5648898	2021-06-22	RHD XV	Gamma

EPI_ISL_5648964	2021-06-22	RHD XV	Gamma
EPI_ISL_5649032	2021-06-22	RHD XV	Gamma
EPI_ISL_5649101	2021-06-22	RHD XV	Gamma
EPI_ISL_5649135	2021-06-22	RHD XV	Gamma
EPI_ISL_5659552	2021-06-22	RHD XV	Gamma
EPI_ISL_5659553	2021-06-22	RHD XV	Gamma
EPI_ISL_5659566	2021-06-22	RHD XV	Gamma
EPI_ISL_5659572	2021-06-22	RHD XV	Gamma
EPI_ISL_5659576	2021-06-22	RHD XV	Gamma
EPI_ISL_5659578	2021-06-22	RHD XV	Gamma
EPI_ISL_5659581	2021-06-22	RHD XV	Gamma
EPI_ISL_5659586	2021-06-22	RHD XV	Gamma
EPI_ISL_5659594	2021-06-22	RHD XV	Gamma
EPI_ISL_5659598	2021-06-22	RHD XV	Gamma
EPI_ISL_5659599	2021-06-22	RHD XV	Gamma
EPI_ISL_5659602	2021-06-22	RHD XV	Gamma
EPI_ISL_5659606	2021-06-22	RHD XV	Gamma
EPI_ISL_5659608	2021-06-22	RHD XV	Gamma
EPI_ISL_5659626	2021-06-22	RHD XV	Gamma
EPI_ISL_5659639	2021-06-22	RHD XV	Gamma
EPI_ISL_3761650	2021-06-22	RHD XV	Gamma
EPI_ISL_3761651	2021-06-22	RHD XV	Gamma
EPI_ISL_3761652	2021-06-22	RHD XV	Gamma
EPI_ISL_3761653	2021-06-22	RHD XV	Gamma
EPI_ISL_3761654	2021-06-22	RHD XV	Gamma
EPI_ISL_3761655	2021-06-22	RHD XV	Gamma
EPI_ISL_3761656	2021-06-22	RHD XV	Gamma
EPI_ISL_3761657	2021-06-22	RHD XV	Gamma
EPI_ISL_3761587	2021-06-23	RHD XV	Gamma
EPI_ISL_3761658	2021-06-23	RHD XV	Gamma
EPI_ISL_3761659	2021-06-23	RHD XV	Gamma
EPI_ISL_3761660	2021-06-23	RHD XV	Gamma
EPI_ISL_3761661	2021-06-23	RHD XV	Gamma
EPI_ISL_3761662	2021-06-23	RHD XV	Gamma
EPI_ISL_3761663	2021-06-23	RHD XV	Gamma
EPI_ISL_3761599	2021-06-23	RHD XV	Gamma
EPI_ISL_3761664	2021-06-23	RHD XV	Gamma
EPI_ISL_3761535	2021-06-23	RHD XV	Gamma
EPI_ISL_3761665	2021-06-24	RHD XV	Gamma
EPI_ISL_3761666	2021-06-24	RHD XV	Gamma
EPI_ISL_3761667	2021-06-24	RHD XV	Gamma
EPI_ISL_3761668	2021-06-24	RHD XV	Gamma
EPI_ISL_3761669	2021-06-24	RHD XV	Gamma
EPI_ISL_3761670	2021-06-24	RHD XV	Gamma
EPI_ISL_3761671	2021-06-24	RHD XV	Gamma
EPI_ISL_3761672	2021-06-24	RHD XV	Gamma
EPI_ISL_3761673	2021-06-24	RHD XV	Gamma
EPI_ISL_5666225	2021-06-27	RHD XV	Gamma
EPI_ISL_5659656	2021-06-28	RHD XV	Gamma
EPI_ISL_5659686	2021-06-28	RHD XV	Gamma
EPI_ISL_5660317	2021-06-28	RHD XV	Gamma
EPI_ISL_5660351	2021-06-28	RHD XV	Gamma
EPI_ISL_5660447	2021-06-28	RHD XV	Gamma
EPI_ISL_5660670	2021-06-28	RHD XV	Gamma
EPI_ISL_5661059	2021-06-28	RHD XV	Gamma
EPI_ISL_5661108	2021-06-28	RHD XV	Gamma
EPI_ISL_5661123	2021-06-28	RHD XV	Gamma
EPI_ISL_5666184	2021-06-28	RHD XV	Gamma
EPI_ISL_5666189	2021-06-28	RHD XV	Gamma
EPI_ISL_5666208	2021-06-28	RHD XV	Gamma
EPI_ISL_5666213	2021-06-28	RHD XV	Gamma
EPI_ISL_5666237	2021-06-28	RHD XV	Gamma
EPI_ISL_5666249	2021-06-28	RHD XV	Gamma

EPI_ISL_5666253	2021-06-28	RHD XV	Gamma
EPI_ISL_5666274	2021-06-28	RHD XV	Gamma
EPI_ISL_5666281	2021-06-28	RHD XV	Gamma
EPI_ISL_5666312	2021-06-28	RHD XV	Gamma
EPI_ISL_5666322	2021-06-28	RHD XV	Gamma
EPI_ISL_5666329	2021-06-28	RHD XV	Gamma
EPI_ISL_5666335	2021-06-28	RHD XV	Gamma
EPI_ISL_5666342	2021-06-28	RHD XV	Gamma
EPI_ISL_5666390	2021-06-28	RHD XV	Gamma
EPI_ISL_5666400	2021-06-28	RHD XV	Gamma
EPI_ISL_5666403	2021-06-28	RHD XV	Gamma
EPI_ISL_5666414	2021-06-28	RHD XV	Gamma
EPI_ISL_5666427	2021-06-28	RHD XV	Gamma
EPI_ISL_5666434	2021-06-28	RHD XV	Gamma
EPI_ISL_5666437	2021-06-28	RHD XV	Gamma
EPI_ISL_5666469	2021-06-28	RHD XV	Gamma
EPI_ISL_5666479	2021-06-28	RHD XV	Gamma
EPI_ISL_5666487	2021-06-28	RHD XV	Gamma
EPI_ISL_5666494	2021-06-28	RHD XV	Gamma
EPI_ISL_5666502	2021-06-28	RHD XV	Gamma
EPI_ISL_5666509	2021-06-28	RHD XV	Gamma
EPI_ISL_5666517	2021-06-28	RHD XV	Gamma
EPI_ISL_5666531	2021-06-28	RHD XV	Gamma
EPI_ISL_5666540	2021-06-28	RHD XV	Gamma
EPI_ISL_5666564	2021-06-28	RHD XV	Gamma
EPI_ISL_5666569	2021-06-28	RHD XV	Gamma
EPI_ISL_5666585	2021-06-28	RHD XV	Gamma
EPI_ISL_5666601	2021-06-28	RHD XV	Gamma
EPI_ISL_5666608	2021-06-28	RHD XV	Gamma
EPI_ISL_5666619	2021-06-28	RHD XV	Gamma
EPI_ISL_5666629	2021-06-28	RHD XV	Gamma
EPI_ISL_5667268	2021-06-28	RHD XV	Gamma
EPI_ISL_5667282	2021-06-28	RHD XV	Gamma
EPI_ISL_5667291	2021-06-28	RHD XV	Gamma
EPI_ISL_5667297	2021-06-28	RHD XV	Gamma
EPI_ISL_5667315	2021-06-28	RHD XV	Gamma
EPI_ISL_5667318	2021-06-28	RHD XV	Gamma
EPI_ISL_5667325	2021-06-28	RHD XV	Gamma
EPI_ISL_5667337	2021-06-28	RHD XV	Gamma
EPI_ISL_5667356	2021-06-28	RHD XV	Gamma
EPI_ISL_5667368	2021-06-28	RHD XV	Gamma
EPI_ISL_5667381	2021-06-28	RHD XV	Gamma
EPI_ISL_5667387	2021-06-28	RHD XV	Gamma
EPI_ISL_5667393	2021-06-28	RHD XV	Gamma
EPI_ISL_5667406	2021-06-28	RHD XV	Gamma
EPI_ISL_5667423	2021-06-28	RHD XV	Gamma
EPI_ISL_5667431	2021-06-28	RHD XV	Gamma
EPI_ISL_5667457	2021-06-28	RHD XV	Gamma
EPI_ISL_5667490	2021-06-28	RHD XV	Gamma
EPI_ISL_5667495	2021-06-28	RHD XV	Gamma
EPI_ISL_5667507	2021-06-28	RHD XV	Gamma
EPI_ISL_5667526	2021-06-28	RHD XV	Gamma
EPI_ISL_5667534	2021-06-28	RHD XV	Gamma
EPI_ISL_5667543	2021-06-28	RHD XV	Gamma
EPI_ISL_5667562	2021-06-28	RHD XV	Gamma
EPI_ISL_5667580	2021-06-28	RHD XV	Gamma
EPI_ISL_5667593	2021-06-28	RHD XV	Gamma
EPI_ISL_5667605	2021-06-28	RHD XV	Gamma
EPI_ISL_5667652	2021-06-28	RHD XV	Gamma
EPI_ISL_5667682	2021-06-28	RHD XV	Gamma
EPI_ISL_5667685	2021-06-28	RHD XV	Gamma
EPI_ISL_5667687	2021-06-28	RHD XV	Gamma
EPI_ISL_5667698	2021-06-28	RHD XV	Gamma

EPI_ISL_5667708	2021-06-28	RHD XV	Gamma
EPI_ISL_5667723	2021-06-28	RHD XV	Gamma
EPI_ISL_5667727	2021-06-28	RHD XV	Gamma
EPI_ISL_5667737	2021-06-28	RHD XV	Gamma
EPI_ISL_5667747	2021-06-28	RHD XV	Gamma
EPI_ISL_5667752	2021-06-28	RHD XV	Gamma
EPI_ISL_5667757	2021-06-28	RHD XV	Gamma
EPI_ISL_5667763	2021-06-28	RHD XV	Gamma
EPI_ISL_5667773	2021-06-28	RHD XV	Gamma
EPI_ISL_5667776	2021-06-28	RHD XV	Gamma
EPI_ISL_5667783	2021-06-28	RHD XV	Gamma
EPI_ISL_5667794	2021-06-28	RHD XV	Gamma
EPI_ISL_5667870	2021-06-28	RHD XV	Gamma
EPI_ISL_5667949	2021-06-28	RHD XV	Gamma
EPI_ISL_5667962	2021-06-28	RHD XV	Gamma
EPI_ISL_5667977	2021-06-28	RHD XV	Gamma
EPI_ISL_5668014	2021-06-28	RHD XV	Gamma
EPI_ISL_5668168	2021-06-28	RHD XV	Gamma
EPI_ISL_5668212	2021-06-28	RHD XV	Gamma
EPI_ISL_5668300	2021-06-28	RHD XV	Gamma
EPI_ISL_5668369	2021-06-28	RHD XV	Gamma
EPI_ISL_5668378	2021-06-28	RHD XV	Gamma
EPI_ISL_11940591	2021-06-28	RHD XV	Gamma
EPI_ISL_3761754	2021-06-28	RHD XV	Gamma
EPI_ISL_5659660	2021-06-29	RHD XV	Gamma
EPI_ISL_5659819	2021-06-29	RHD XV	Gamma
EPI_ISL_5659939	2021-06-29	RHD XV	Gamma
EPI_ISL_5660106	2021-06-29	RHD XV	Gamma
EPI_ISL_5660147	2021-06-29	RHD XV	Gamma
EPI_ISL_5660203	2021-06-29	RHD XV	Gamma
EPI_ISL_5660292	2021-06-29	RHD XV	Gamma
EPI_ISL_5660631	2021-06-29	RHD XV	Gamma
EPI_ISL_5660878	2021-06-29	RHD XV	Gamma
EPI_ISL_5660981	2021-06-29	RHD XV	Gamma
EPI_ISL_5666199	2021-06-29	RHD XV	Gamma
EPI_ISL_5666265	2021-06-29	RHD XV	Gamma
EPI_ISL_5666349	2021-06-29	RHD XV	Gamma
EPI_ISL_5666374	2021-06-29	RHD XV	Gamma
EPI_ISL_5666557	2021-06-29	RHD XV	Gamma
EPI_ISL_5667374	2021-06-29	RHD XV	Gamma
EPI_ISL_5667399	2021-06-29	RHD XV	Gamma
EPI_ISL_5667445	2021-06-29	RHD XV	Gamma
EPI_ISL_5667572	2021-06-29	RHD XV	Gamma
EPI_ISL_5667654	2021-06-29	RHD XV	Gamma
EPI_ISL_5667702	2021-06-29	RHD XV	Gamma
EPI_ISL_5667803	2021-06-29	RHD XV	Gamma
EPI_ISL_5667809	2021-06-29	RHD XV	Gamma
EPI_ISL_5667887	2021-06-29	RHD XV	Gamma
EPI_ISL_5667902	2021-06-29	RHD XV	Gamma
EPI_ISL_5667941	2021-06-29	RHD XV	Gamma
EPI_ISL_5667983	2021-06-29	RHD XV	Gamma
EPI_ISL_5668123	2021-06-29	RHD XV	Gamma
EPI_ISL_5668145	2021-06-29	RHD XV	Gamma
EPI_ISL_5668151	2021-06-29	RHD XV	Gamma
EPI_ISL_5668253	2021-06-29	RHD XV	Gamma
EPI_ISL_5668347	2021-06-29	RHD XV	Gamma
EPI_ISL_5668359	2021-06-29	RHD XV	Gamma
EPI_ISL_5668375	2021-06-29	RHD XV	Gamma
EPI_ISL_5668394	2021-06-29	RHD XV	Gamma
EPI_ISL_5668402	2021-06-29	RHD XV	Gamma
EPI_ISL_3049021	2021-06-29	RHD XV	Gamma
EPI_ISL_3049023	2021-06-29	RHD XV	Gamma
EPI_ISL_3049024	2021-06-29	RHD XV	Gamma

EPI_ISL_3049025	2021-06-29	RHD XV	Gamma
EPI_ISL_3049026	2021-06-29	RHD XV	Gamma
EPI_ISL_3049027	2021-06-29	RHD XV	Gamma
EPI_ISL_3049028	2021-06-29	RHD XV	Gamma
EPI_ISL_3049029	2021-06-29	RHD XV	Gamma
EPI_ISL_3055532	2021-06-29	RHD XV	Gamma
EPI_ISL_3055533	2021-06-29	RHD XV	Gamma
EPI_ISL_3049030	2021-06-29	RHD XV	Gamma
EPI_ISL_3761755	2021-06-29	RHD XV	Gamma
EPI_ISL_3761586	2021-06-29	RHD XV	Gamma
EPI_ISL_3761756	2021-06-29	RHD XV	Gamma
EPI_ISL_3761523	2021-06-30	RHD XV	Gamma
EPI_ISL_3761757	2021-06-30	RHD XV	Gamma
EPI_ISL_3761758	2021-06-30	RHD XV	Gamma
EPI_ISL_3761528	2021-06-30	RHD XV	Gamma
EPI_ISL_3761759	2021-06-30	RHD XV	Gamma
EPI_ISL_3761563	2021-07-01	RHD XV	Gamma
EPI_ISL_3761522	2021-07-01	RHD XV	Gamma
EPI_ISL_3761674	2021-07-01	RHD XV	Gamma
EPI_ISL_3761588	2021-07-01	RHD XV	Gamma
EPI_ISL_3761675	2021-07-01	RHD XV	Gamma
EPI_ISL_3761558	2021-07-01	RHD XV	Gamma
EPI_ISL_3761676	2021-07-01	RHD XV	Gamma
EPI_ISL_3761539	2021-07-01	RHD XV	Gamma
EPI_ISL_3761677	2021-07-01	RHD XV	Gamma
EPI_ISL_3761678	2021-07-01	RHD XV	Gamma
EPI_ISL_3761679	2021-07-01	RHD XV	Gamma
EPI_ISL_3761680	2021-07-01	RHD XV	Gamma
EPI_ISL_3761573	2021-07-01	RHD XV	Gamma
EPI_ISL_5673245	2021-07-05	RHD XV	Alpha
EPI_ISL_5672042	2021-07-05	RHD XV	Gamma
EPI_ISL_5672056	2021-07-05	RHD XV	Gamma
EPI_ISL_5672085	2021-07-05	RHD XV	Gamma
EPI_ISL_5672090	2021-07-05	RHD XV	Gamma
EPI_ISL_5672120	2021-07-05	RHD XV	Gamma
EPI_ISL_5672129	2021-07-05	RHD XV	Gamma
EPI_ISL_5672134	2021-07-05	RHD XV	Gamma
EPI_ISL_5672174	2021-07-05	RHD XV	Gamma
EPI_ISL_5672180	2021-07-05	RHD XV	Gamma
EPI_ISL_5672185	2021-07-05	RHD XV	Gamma
EPI_ISL_5672289	2021-07-05	RHD XV	Gamma
EPI_ISL_5672319	2021-07-05	RHD XV	Gamma
EPI_ISL_5672334	2021-07-05	RHD XV	Gamma
EPI_ISL_5672370	2021-07-05	RHD XV	Gamma
EPI_ISL_5672422	2021-07-05	RHD XV	Gamma
EPI_ISL_5672434	2021-07-05	RHD XV	Gamma
EPI_ISL_5672485	2021-07-05	RHD XV	Gamma
EPI_ISL_5672497	2021-07-05	RHD XV	Gamma
EPI_ISL_5672502	2021-07-05	RHD XV	Gamma
EPI_ISL_5672514	2021-07-05	RHD XV	Gamma
EPI_ISL_5672530	2021-07-05	RHD XV	Gamma
EPI_ISL_5672550	2021-07-05	RHD XV	Gamma
EPI_ISL_5673293	2021-07-05	RHD XV	Gamma
EPI_ISL_5673342	2021-07-05	RHD XV	Gamma
EPI_ISL_5673352	2021-07-05	RHD XV	Gamma
EPI_ISL_5673372	2021-07-05	RHD XV	Gamma
EPI_ISL_5673393	2021-07-05	RHD XV	Gamma
EPI_ISL_5673399	2021-07-05	RHD XV	Gamma
EPI_ISL_5673420	2021-07-05	RHD XV	Gamma
EPI_ISL_5673429	2021-07-05	RHD XV	Gamma
EPI_ISL_5673454	2021-07-05	RHD XV	Gamma
EPI_ISL_5673464	2021-07-05	RHD XV	Gamma
EPI_ISL_5673562	2021-07-05	RHD XV	Gamma

EPI_ISL_5673583	2021-07-05	RHD XV	Gamma
EPI_ISL_5673637	2021-07-05	RHD XV	Gamma
EPI_ISL_5673654	2021-07-05	RHD XV	Gamma
EPI_ISL_5673679	2021-07-05	RHD XV	Gamma
EPI_ISL_5673686	2021-07-05	RHD XV	Gamma
EPI_ISL_5673714	2021-07-05	RHD XV	Gamma
EPI_ISL_5673742	2021-07-05	RHD XV	Gamma
EPI_ISL_5673758	2021-07-05	RHD XV	Gamma
EPI_ISL_5673773	2021-07-05	RHD XV	Gamma
EPI_ISL_5673807	2021-07-05	RHD XV	Gamma
EPI_ISL_5673867	2021-07-05	RHD XV	Gamma
EPI_ISL_5673883	2021-07-05	RHD XV	Gamma
EPI_ISL_5673888	2021-07-05	RHD XV	Gamma
EPI_ISL_5673920	2021-07-05	RHD XV	Gamma
EPI_ISL_5674030	2021-07-05	RHD XV	Gamma
EPI_ISL_5674086	2021-07-05	RHD XV	Gamma
EPI_ISL_5674112	2021-07-05	RHD XV	Gamma
EPI_ISL_5674177	2021-07-05	RHD XV	Gamma
EPI_ISL_5674220	2021-07-05	RHD XV	Gamma
EPI_ISL_5674400	2021-07-05	RHD XV	Gamma
EPI_ISL_5674434	2021-07-05	RHD XV	Gamma
EPI_ISL_5674515	2021-07-05	RHD XV	Gamma
EPI_ISL_5674624	2021-07-05	RHD XV	Gamma
EPI_ISL_5674630	2021-07-05	RHD XV	Gamma
EPI_ISL_5674809	2021-07-05	RHD XV	Gamma
EPI_ISL_5674869	2021-07-05	RHD XV	Gamma
EPI_ISL_5674874	2021-07-05	RHD XV	Gamma
EPI_ISL_5674983	2021-07-05	RHD XV	Gamma
EPI_ISL_5674987	2021-07-05	RHD XV	Gamma
EPI_ISL_5675204	2021-07-05	RHD XV	Gamma
EPI_ISL_5675352	2021-07-05	RHD XV	Gamma
EPI_ISL_5675359	2021-07-05	RHD XV	Gamma
EPI_ISL_5675365	2021-07-05	RHD XV	Gamma
EPI_ISL_5675370	2021-07-05	RHD XV	Gamma
EPI_ISL_5675381	2021-07-05	RHD XV	Gamma
EPI_ISL_5675394	2021-07-05	RHD XV	Gamma
EPI_ISL_5675399	2021-07-05	RHD XV	Gamma
EPI_ISL_5675408	2021-07-05	RHD XV	Gamma
EPI_ISL_5675411	2021-07-05	RHD XV	Gamma
EPI_ISL_5675415	2021-07-05	RHD XV	Gamma
EPI_ISL_5675454	2021-07-05	RHD XV	Gamma
EPI_ISL_5675464	2021-07-05	RHD XV	Gamma
EPI_ISL_5675470	2021-07-05	RHD XV	Gamma
EPI_ISL_5675487	2021-07-05	RHD XV	Gamma
EPI_ISL_5675494	2021-07-05	RHD XV	Gamma
EPI_ISL_3761681	2021-07-05	RHD XV	Gamma
EPI_ISL_3761682	2021-07-05	RHD XV	Gamma
EPI_ISL_3761683	2021-07-05	RHD XV	Gamma
EPI_ISL_3761530	2021-07-05	RHD XV	Gamma
EPI_ISL_3761684	2021-07-05	RHD XV	Gamma
EPI_ISL_3761685	2021-07-05	RHD XV	Gamma
EPI_ISL_3761686	2021-07-05	RHD XV	Gamma
EPI_ISL_3761614	2021-07-05	RHD XV	Gamma
EPI_ISL_3761687	2021-07-05	RHD XV	Gamma
EPI_ISL_5673763	2021-07-06	RHD XV	Gamma
EPI_ISL_5673922	2021-07-06	RHD XV	Gamma
EPI_ISL_5674148	2021-07-06	RHD XV	Gamma
EPI_ISL_5674278	2021-07-06	RHD XV	Gamma
EPI_ISL_5675387	2021-07-06	RHD XV	Gamma
EPI_ISL_3761688	2021-07-06	RHD XV	Gamma
EPI_ISL_3761689	2021-07-06	RHD XV	Gamma
EPI_ISL_3761690	2021-07-06	RHD XV	Gamma
EPI_ISL_3761691	2021-07-06	RHD XV	Gamma

EPI_ISL_3761605	2021-07-06	RHD XV	Gamma
EPI_ISL_3761589	2021-07-07	RHD XV	Gamma
EPI_ISL_3761537	2021-07-07	RHD XV	Gamma
EPI_ISL_3761597	2021-07-07	RHD XV	Gamma
EPI_ISL_3761692	2021-07-07	RHD XV	Gamma
EPI_ISL_3761693	2021-07-07	RHD XV	Gamma
EPI_ISL_3761567	2021-07-07	RHD XV	Gamma
EPI_ISL_3761694	2021-07-07	RHD XV	Gamma
EPI_ISL_3761695	2021-07-07	RHD XV	Gamma
EPI_ISL_3761546	2021-07-07	RHD XV	Gamma
EPI_ISL_3761696	2021-07-07	RHD XV	Gamma
EPI_ISL_3761697	2021-07-07	RHD XV	Gamma
EPI_ISL_3761545	2021-07-07	RHD XV	Gamma
EPI_ISL_3761561	2021-07-07	RHD XV	Gamma
EPI_ISL_3761554	2021-07-07	RHD XV	Gamma
EPI_ISL_3761547	2021-07-07	RHD XV	Gamma
EPI_ISL_3761608	2021-07-07	RHD XV	Gamma
EPI_ISL_3761698	2021-07-07	RHD XV	Gamma
EPI_ISL_3761600	2021-07-07	RHD XV	Gamma
EPI_ISL_3761569	2021-07-07	RHD XV	Gamma
EPI_ISL_3761544	2021-07-07	RHD XV	Gamma
EPI_ISL_3761556	2021-07-07	RHD XV	Gamma
EPI_ISL_3761699	2021-07-07	RHD XV	Gamma
EPI_ISL_3761572	2021-07-12	RHD XV	Alpha
EPI_ISL_3761701	2021-07-12	RHD XV	B.1.1.28
EPI_ISL_5675588	2021-07-12	RHD XV	Gamma
EPI_ISL_5675709	2021-07-12	RHD XV	Gamma
EPI_ISL_5675719	2021-07-12	RHD XV	Gamma
EPI_ISL_5675774	2021-07-12	RHD XV	Gamma
EPI_ISL_5675793	2021-07-12	RHD XV	Gamma
EPI_ISL_5675937	2021-07-12	RHD XV	Gamma
EPI_ISL_5675943	2021-07-12	RHD XV	Gamma
EPI_ISL_5676069	2021-07-12	RHD XV	Gamma
EPI_ISL_5676108	2021-07-12	RHD XV	Gamma
EPI_ISL_5676155	2021-07-12	RHD XV	Gamma
EPI_ISL_5676188	2021-07-12	RHD XV	Gamma
EPI_ISL_5676211	2021-07-12	RHD XV	Gamma
EPI_ISL_5676361	2021-07-12	RHD XV	Gamma
EPI_ISL_5676375	2021-07-12	RHD XV	Gamma
EPI_ISL_5676582	2021-07-12	RHD XV	Gamma
EPI_ISL_5676643	2021-07-12	RHD XV	Gamma
EPI_ISL_5676683	2021-07-12	RHD XV	Gamma
EPI_ISL_5676806	2021-07-12	RHD XV	Gamma
EPI_ISL_5676834	2021-07-12	RHD XV	Gamma
EPI_ISL_5676848	2021-07-12	RHD XV	Gamma
EPI_ISL_5676849	2021-07-12	RHD XV	Gamma
EPI_ISL_5676853	2021-07-12	RHD XV	Gamma
EPI_ISL_5676855	2021-07-12	RHD XV	Gamma
EPI_ISL_5676865	2021-07-12	RHD XV	Gamma
EPI_ISL_5676939	2021-07-12	RHD XV	Gamma
EPI_ISL_5677013	2021-07-12	RHD XV	Gamma
EPI_ISL_5677044	2021-07-12	RHD XV	Gamma
EPI_ISL_5677126	2021-07-12	RHD XV	Gamma
EPI_ISL_5677156	2021-07-12	RHD XV	Gamma
EPI_ISL_5677164	2021-07-12	RHD XV	Gamma
EPI_ISL_5677214	2021-07-12	RHD XV	Gamma
EPI_ISL_5677220	2021-07-12	RHD XV	Gamma
EPI_ISL_5677241	2021-07-12	RHD XV	Gamma
EPI_ISL_5677292	2021-07-12	RHD XV	Gamma
EPI_ISL_5677294	2021-07-12	RHD XV	Gamma
EPI_ISL_5677355	2021-07-12	RHD XV	Gamma
EPI_ISL_5677397	2021-07-12	RHD XV	Gamma
EPI_ISL_5677496	2021-07-12	RHD XV	Gamma

EPI_ISL_5677513	2021-07-12	RHD XV	Gamma
EPI_ISL_5677539	2021-07-12	RHD XV	Gamma
EPI_ISL_5677591	2021-07-12	RHD XV	Gamma
EPI_ISL_5677596	2021-07-12	RHD XV	Gamma
EPI_ISL_5677602	2021-07-12	RHD XV	Gamma
EPI_ISL_5677632	2021-07-12	RHD XV	Gamma
EPI_ISL_5677640	2021-07-12	RHD XV	Gamma
EPI_ISL_5677680	2021-07-12	RHD XV	Gamma
EPI_ISL_5677690	2021-07-12	RHD XV	Gamma
EPI_ISL_5677698	2021-07-12	RHD XV	Gamma
EPI_ISL_5677716	2021-07-12	RHD XV	Gamma
EPI_ISL_5677719	2021-07-12	RHD XV	Gamma
EPI_ISL_5677730	2021-07-12	RHD XV	Gamma
EPI_ISL_5678004	2021-07-12	RHD XV	Gamma
EPI_ISL_5678082	2021-07-12	RHD XV	Gamma
EPI_ISL_5678358	2021-07-12	RHD XV	Gamma
EPI_ISL_5678549	2021-07-12	RHD XV	Gamma
EPI_ISL_5678590	2021-07-12	RHD XV	Gamma
EPI_ISL_5678641	2021-07-12	RHD XV	Gamma
EPI_ISL_5678672	2021-07-12	RHD XV	Gamma
EPI_ISL_5678759	2021-07-12	RHD XV	Gamma
EPI_ISL_5678791	2021-07-12	RHD XV	Gamma
EPI_ISL_5678843	2021-07-12	RHD XV	Gamma
EPI_ISL_5678874	2021-07-12	RHD XV	Gamma
EPI_ISL_5678888	2021-07-12	RHD XV	Gamma
EPI_ISL_5678947	2021-07-12	RHD XV	Gamma
EPI_ISL_5679027	2021-07-12	RHD XV	Gamma
EPI_ISL_5679073	2021-07-12	RHD XV	Gamma
EPI_ISL_5679078	2021-07-12	RHD XV	Gamma
EPI_ISL_5679162	2021-07-12	RHD XV	Gamma
EPI_ISL_5679546	2021-07-12	RHD XV	Gamma
EPI_ISL_5679742	2021-07-12	RHD XV	Gamma
EPI_ISL_5679751	2021-07-12	RHD XV	Gamma
EPI_ISL_5679792	2021-07-12	RHD XV	Gamma
EPI_ISL_5679863	2021-07-12	RHD XV	Gamma
EPI_ISL_5679987	2021-07-12	RHD XV	Gamma
EPI_ISL_5680023	2021-07-12	RHD XV	Gamma
EPI_ISL_5680027	2021-07-12	RHD XV	Gamma
EPI_ISL_5680060	2021-07-12	RHD XV	Gamma
EPI_ISL_5680098	2021-07-12	RHD XV	Gamma
EPI_ISL_5680114	2021-07-12	RHD XV	Gamma
EPI_ISL_5680120	2021-07-12	RHD XV	Gamma
EPI_ISL_5680124	2021-07-12	RHD XV	Gamma
EPI_ISL_5680127	2021-07-12	RHD XV	Gamma
EPI_ISL_5680139	2021-07-12	RHD XV	Gamma
EPI_ISL_5680146	2021-07-12	RHD XV	Gamma
EPI_ISL_5680156	2021-07-12	RHD XV	Gamma
EPI_ISL_5680165	2021-07-12	RHD XV	Gamma
EPI_ISL_5680172	2021-07-12	RHD XV	Gamma
EPI_ISL_5680182	2021-07-12	RHD XV	Gamma
EPI_ISL_5680189	2021-07-12	RHD XV	Gamma
EPI_ISL_5680195	2021-07-12	RHD XV	Gamma
EPI_ISL_5680202	2021-07-12	RHD XV	Gamma
EPI_ISL_5680214	2021-07-12	RHD XV	Gamma
EPI_ISL_5680218	2021-07-12	RHD XV	Gamma
EPI_ISL_5680223	2021-07-12	RHD XV	Gamma
EPI_ISL_3761565	2021-07-12	RHD XV	Gamma
EPI_ISL_3761700	2021-07-12	RHD XV	Gamma
EPI_ISL_3761609	2021-07-12	RHD XV	Gamma
EPI_ISL_3761702	2021-07-12	RHD XV	Gamma
EPI_ISL_3761764	2021-07-12	RHD XV	Gamma
EPI_ISL_3761703	2021-07-12	RHD XV	Gamma
EPI_ISL_3761704	2021-07-12	RHD XV	Gamma

EPI_ISL_3761705	2021-07-12	RHD XV	Gamma
EPI_ISL_3761706	2021-07-12	RHD XV	Gamma
EPI_ISL_3761707	2021-07-12	RHD XV	Gamma
EPI_ISL_3761708	2021-07-12	RHD XV	Gamma
EPI_ISL_3761709	2021-07-12	RHD XV	Gamma
EPI_ISL_5679647	2021-07-13	RHD XV	Gamma
EPI_ISL_3761555	2021-07-13	RHD XV	Gamma
EPI_ISL_3761531	2021-07-13	RHD XV	Gamma
EPI_ISL_3761536	2021-07-13	RHD XV	Gamma
EPI_ISL_3761710	2021-07-13	RHD XV	Gamma
EPI_ISL_3761551	2021-07-13	RHD XV	Gamma
EPI_ISL_3761519	2021-07-13	RHD XV	Gamma
EPI_ISL_3761524	2021-07-13	RHD XV	Gamma
EPI_ISL_3761568	2021-07-13	RHD XV	Gamma
EPI_ISL_3761526	2021-07-13	RHD XV	Gamma
EPI_ISL_3761711	2021-07-13	RHD XV	Gamma
EPI_ISL_3761712	2021-07-13	RHD XV	Gamma
EPI_ISL_3761713	2021-07-13	RHD XV	Gamma
EPI_ISL_3761714	2021-07-14	RHD XV	Gamma
EPI_ISL_3761715	2021-07-14	RHD XV	Gamma
EPI_ISL_3761716	2021-07-14	RHD XV	Gamma
EPI_ISL_3761610	2021-07-14	RHD XV	Gamma
EPI_ISL_3761717	2021-07-14	RHD XV	Gamma
EPI_ISL_3761601	2021-07-14	RHD XV	Gamma
EPI_ISL_3761718	2021-07-14	RHD XV	Gamma
EPI_ISL_3761719	2021-07-14	RHD XV	Gamma
EPI_ISL_3761602	2021-07-14	RHD XV	Gamma
EPI_ISL_3761532	2021-07-14	RHD XV	Gamma
EPI_ISL_3761720	2021-07-14	RHD XV	Gamma
EPI_ISL_3761615	2021-07-14	RHD XV	Gamma
EPI_ISL_3761721	2021-07-14	RHD XV	Gamma
EPI_ISL_3761564	2021-07-14	RHD XV	Gamma
EPI_ISL_3761548	2021-07-14	RHD XV	Gamma
EPI_ISL_3761722	2021-07-14	RHD XV	Gamma
EPI_ISL_3761590	2021-07-14	RHD XV	Gamma
EPI_ISL_3761723	2021-07-14	RHD XV	Gamma
EPI_ISL_3761724	2021-07-15	RHD XV	Gamma
EPI_ISL_3761725	2021-07-15	RHD XV	Gamma
EPI_ISL_3761726	2021-07-15	RHD XV	Gamma
EPI_ISL_3761603	2021-07-15	RHD XV	Gamma
EPI_ISL_3761585	2021-07-15	RHD XV	Gamma
EPI_ISL_3761727	2021-07-15	RHD XV	Gamma
EPI_ISL_3761728	2021-07-15	RHD XV	Gamma
EPI_ISL_3761729	2021-07-15	RHD XV	Gamma
EPI_ISL_3761730	2021-07-15	RHD XV	Gamma
EPI_ISL_3761731	2021-07-15	RHD XV	Gamma
EPI_ISL_3761732	2021-07-15	RHD XV	Gamma
EPI_ISL_3761733	2021-07-15	RHD XV	Gamma
EPI_ISL_3761518	2021-07-15	RHD XV	Gamma
EPI_ISL_5652021	2021-07-17	RHD XV	Gamma
EPI_ISL_5653794	2021-07-19	RHD XV	Gamma
EPI_ISL_5651828	2021-07-19	RHD XV	Gamma
EPI_ISL_5651841	2021-07-19	RHD XV	Gamma
EPI_ISL_5651847	2021-07-19	RHD XV	Gamma
EPI_ISL_5651855	2021-07-19	RHD XV	Gamma
EPI_ISL_5651866	2021-07-19	RHD XV	Gamma
EPI_ISL_5651917	2021-07-19	RHD XV	Gamma
EPI_ISL_5650469	2021-07-19	RHD XV	Gamma
EPI_ISL_5650433	2021-07-19	RHD XV	Gamma
EPI_ISL_5651920	2021-07-19	RHD XV	Gamma
EPI_ISL_5650652	2021-07-19	RHD XV	Gamma
EPI_ISL_5651921	2021-07-19	RHD XV	Gamma
EPI_ISL_5651922	2021-07-19	RHD XV	Gamma

EPI_ISL_5651930	2021-07-19	RHD XV	Gamma
EPI_ISL_5651931	2021-07-19	RHD XV	Gamma
EPI_ISL_5651932	2021-07-19	RHD XV	Gamma
EPI_ISL_5651936	2021-07-19	RHD XV	Gamma
EPI_ISL_5651937	2021-07-19	RHD XV	Gamma
EPI_ISL_5651938	2021-07-19	RHD XV	Gamma
EPI_ISL_5651941	2021-07-19	RHD XV	Gamma
EPI_ISL_5651948	2021-07-19	RHD XV	Gamma
EPI_ISL_5651949	2021-07-19	RHD XV	Gamma
EPI_ISL_5651950	2021-07-19	RHD XV	Gamma
EPI_ISL_5651952	2021-07-19	RHD XV	Gamma
EPI_ISL_5651955	2021-07-19	RHD XV	Gamma
EPI_ISL_5651958	2021-07-19	RHD XV	Gamma
EPI_ISL_5651961	2021-07-19	RHD XV	Gamma
EPI_ISL_5650430	2021-07-19	RHD XV	Gamma
EPI_ISL_5651966	2021-07-19	RHD XV	Gamma
EPI_ISL_5651968	2021-07-19	RHD XV	Gamma
EPI_ISL_5651969	2021-07-19	RHD XV	Gamma
EPI_ISL_5651970	2021-07-19	RHD XV	Gamma
EPI_ISL_5651971	2021-07-19	RHD XV	Gamma
EPI_ISL_5651972	2021-07-19	RHD XV	Gamma
EPI_ISL_5650500	2021-07-19	RHD XV	Gamma
EPI_ISL_5651974	2021-07-19	RHD XV	Gamma
EPI_ISL_5651975	2021-07-19	RHD XV	Gamma
EPI_ISL_5651976	2021-07-19	RHD XV	Gamma
EPI_ISL_5651978	2021-07-19	RHD XV	Gamma
EPI_ISL_5651980	2021-07-19	RHD XV	Gamma
EPI_ISL_5651982	2021-07-19	RHD XV	Gamma
EPI_ISL_5651983	2021-07-19	RHD XV	Gamma
EPI_ISL_5651985	2021-07-19	RHD XV	Gamma
EPI_ISL_5651986	2021-07-19	RHD XV	Gamma
EPI_ISL_5655103	2021-07-19	RHD XV	Gamma
EPI_ISL_5655042	2021-07-19	RHD XV	Gamma
EPI_ISL_5654099	2021-07-19	RHD XV	Gamma
EPI_ISL_5652012	2021-07-19	RHD XV	Gamma
EPI_ISL_5652016	2021-07-19	RHD XV	Gamma
EPI_ISL_5652017	2021-07-19	RHD XV	Gamma
EPI_ISL_5652019	2021-07-19	RHD XV	Gamma
EPI_ISL_5653868	2021-07-19	RHD XV	Gamma
EPI_ISL_5652020	2021-07-19	RHD XV	Gamma
EPI_ISL_5652022	2021-07-19	RHD XV	Gamma
EPI_ISL_5650451	2021-07-19	RHD XV	Gamma
EPI_ISL_5650597	2021-07-19	RHD XV	Gamma
EPI_ISL_5650598	2021-07-19	RHD XV	Gamma
EPI_ISL_5652025	2021-07-19	RHD XV	Gamma
EPI_ISL_5652026	2021-07-19	RHD XV	Gamma
EPI_ISL_5650599	2021-07-19	RHD XV	Gamma
EPI_ISL_5652028	2021-07-19	RHD XV	Gamma
EPI_ISL_5652029	2021-07-19	RHD XV	Gamma
EPI_ISL_5652030	2021-07-19	RHD XV	Gamma
EPI_ISL_5652031	2021-07-19	RHD XV	Gamma
EPI_ISL_5652033	2021-07-19	RHD XV	Gamma
EPI_ISL_5652034	2021-07-19	RHD XV	Gamma
EPI_ISL_5652035	2021-07-19	RHD XV	Gamma
EPI_ISL_5652038	2021-07-19	RHD XV	Gamma
EPI_ISL_5650600	2021-07-19	RHD XV	Gamma
EPI_ISL_5650452	2021-07-19	RHD XV	Gamma
EPI_ISL_5650653	2021-07-19	RHD XV	Gamma
EPI_ISL_5650453	2021-07-19	RHD XV	Gamma
EPI_ISL_5650650	2021-07-19	RHD XV	Gamma
EPI_ISL_5652047	2021-07-19	RHD XV	Gamma
EPI_ISL_5652048	2021-07-19	RHD XV	Gamma
EPI_ISL_5652050	2021-07-19	RHD XV	Gamma

EPI_ISL_5652051	2021-07-19	RHD XV	Gamma
EPI_ISL_5652057	2021-07-19	RHD XV	Gamma
EPI_ISL_5654212	2021-07-19	RHD XV	Gamma
EPI_ISL_5652060	2021-07-19	RHD XV	Gamma
EPI_ISL_5652061	2021-07-19	RHD XV	Gamma
EPI_ISL_5652063	2021-07-19	RHD XV	Gamma
EPI_ISL_5652064	2021-07-19	RHD XV	Gamma
EPI_ISL_5652065	2021-07-19	RHD XV	Gamma
EPI_ISL_5652066	2021-07-19	RHD XV	Gamma
EPI_ISL_5652067	2021-07-19	RHD XV	Gamma
EPI_ISL_5652069	2021-07-19	RHD XV	Gamma
EPI_ISL_5652070	2021-07-19	RHD XV	Gamma
EPI_ISL_5652071	2021-07-19	RHD XV	Gamma
EPI_ISL_5652072	2021-07-19	RHD XV	Gamma
EPI_ISL_5652073	2021-07-19	RHD XV	Gamma
EPI_ISL_5652074	2021-07-19	RHD XV	Gamma
EPI_ISL_3761521	2021-07-19	RHD XV	Gamma
EPI_ISL_3761540	2021-07-19	RHD XV	Gamma
EPI_ISL_3761734	2021-07-19	RHD XV	Gamma
EPI_ISL_3761611	2021-07-19	RHD XV	Gamma
EPI_ISL_3761735	2021-07-19	RHD XV	Gamma
EPI_ISL_3761736	2021-07-19	RHD XV	Gamma
EPI_ISL_3761598	2021-07-19	RHD XV	Gamma
EPI_ISL_3761762	2021-07-19	RHD XV	Gamma
EPI_ISL_3761737	2021-07-19	RHD XV	Gamma
EPI_ISL_3761738	2021-07-19	RHD XV	Gamma
EPI_ISL_3761739	2021-07-19	RHD XV	Gamma
EPI_ISL_3761740	2021-07-19	RHD XV	Gamma
EPI_ISL_3761741	2021-07-19	RHD XV	Gamma
EPI_ISL_3761742	2021-07-19	RHD XV	Gamma
EPI_ISL_5650668	2021-07-20	RHD XV	Gamma
EPI_ISL_5651924	2021-07-20	RHD XV	Gamma
EPI_ISL_5651925	2021-07-20	RHD XV	Gamma
EPI_ISL_5651929	2021-07-20	RHD XV	Gamma
EPI_ISL_5650523	2021-07-20	RHD XV	Gamma
EPI_ISL_5651935	2021-07-20	RHD XV	Gamma
EPI_ISL_5651939	2021-07-20	RHD XV	Gamma
EPI_ISL_5650427	2021-07-20	RHD XV	Gamma
EPI_ISL_5651945	2021-07-20	RHD XV	Gamma
EPI_ISL_5651954	2021-07-20	RHD XV	Gamma
EPI_ISL_5651956	2021-07-20	RHD XV	Gamma
EPI_ISL_5651963	2021-07-20	RHD XV	Gamma
EPI_ISL_5652056	2021-07-20	RHD XV	Gamma
EPI_ISL_3761577	2021-07-20	RHD XV	Gamma
EPI_ISL_3761571	2021-07-20	RHD XV	Gamma
EPI_ISL_3761743	2021-07-20	RHD XV	Gamma
EPI_ISL_3761744	2021-07-20	RHD XV	Gamma
EPI_ISL_3761552	2021-07-20	RHD XV	Gamma
EPI_ISL_3761562	2021-07-20	RHD XV	Gamma
EPI_ISL_3761745	2021-07-20	RHD XV	Gamma
EPI_ISL_3761612	2021-07-20	RHD XV	Gamma
EPI_ISL_3761543	2021-07-20	RHD XV	Gamma
EPI_ISL_3761560	2021-07-20	RHD XV	Gamma
EPI_ISL_3761578	2021-07-20	RHD XV	Gamma
EPI_ISL_3761557	2021-07-20	RHD XV	Gamma
EPI_ISL_3761746	2021-07-20	RHD XV	Gamma
EPI_ISL_3761542	2021-07-20	RHD XV	Gamma
EPI_ISL_3761575	2021-07-20	RHD XV	Gamma
EPI_ISL_3761747	2021-07-20	RHD XV	Gamma
EPI_ISL_3761553	2021-07-20	RHD XV	Gamma
EPI_ISL_3761748	2021-07-20	RHD XV	Gamma
EPI_ISL_5652092	2021-07-25	RHD XV	Gamma
EPI_ISL_5652098	2021-07-25	RHD XV	Gamma

EPI_ISL_5652103	2021-07-25	RHD XV	Gamma
EPI_ISL_5652105	2021-07-25	RHD XV	Gamma
EPI_ISL_5652139	2021-07-25	RHD XV	Gamma
EPI_ISL_5652168	2021-07-25	RHD XV	Gamma
EPI_ISL_5653764	2021-07-26	RHD XV	Gamma
EPI_ISL_5654855	2021-07-26	RHD XV	Gamma
EPI_ISL_5654727	2021-07-26	RHD XV	Gamma
EPI_ISL_5654439	2021-07-26	RHD XV	Gamma
EPI_ISL_5652086	2021-07-26	RHD XV	Gamma
EPI_ISL_5652090	2021-07-26	RHD XV	Gamma
EPI_ISL_5650601	2021-07-26	RHD XV	Gamma
EPI_ISL_5652095	2021-07-26	RHD XV	Gamma
EPI_ISL_5650556	2021-07-26	RHD XV	Gamma
EPI_ISL_5653869	2021-07-26	RHD XV	Gamma
EPI_ISL_5652129	2021-07-26	RHD XV	Gamma
EPI_ISL_5650635	2021-07-26	RHD XV	Gamma
EPI_ISL_5652142	2021-07-26	RHD XV	Gamma
EPI_ISL_5652150	2021-07-26	RHD XV	Gamma
EPI_ISL_5652158	2021-07-26	RHD XV	Gamma
EPI_ISL_5652163	2021-07-26	RHD XV	Gamma
EPI_ISL_5652167	2021-07-26	RHD XV	Gamma
EPI_ISL_5652170	2021-07-26	RHD XV	Gamma
EPI_ISL_5652171	2021-07-26	RHD XV	Gamma
EPI_ISL_5652177	2021-07-26	RHD XV	Gamma
EPI_ISL_5652179	2021-07-26	RHD XV	Gamma
EPI_ISL_5652185	2021-07-26	RHD XV	Gamma
EPI_ISL_5652194	2021-07-26	RHD XV	Gamma
EPI_ISL_5652217	2021-07-26	RHD XV	Gamma
EPI_ISL_5652219	2021-07-26	RHD XV	Gamma
EPI_ISL_5652220	2021-07-26	RHD XV	Gamma
EPI_ISL_5652224	2021-07-26	RHD XV	Gamma
EPI_ISL_5652247	2021-07-26	RHD XV	Gamma
EPI_ISL_5652255	2021-07-26	RHD XV	Gamma
EPI_ISL_5652258	2021-07-26	RHD XV	Gamma
EPI_ISL_5652269	2021-07-26	RHD XV	Gamma
EPI_ISL_5652272	2021-07-26	RHD XV	Gamma
EPI_ISL_5655121	2021-07-26	RHD XV	Gamma
EPI_ISL_5655111	2021-07-26	RHD XV	Gamma
EPI_ISL_5652284	2021-07-26	RHD XV	Gamma
EPI_ISL_5652286	2021-07-26	RHD XV	Gamma
EPI_ISL_5652307	2021-07-26	RHD XV	Gamma
EPI_ISL_5652310	2021-07-26	RHD XV	Gamma
EPI_ISL_5652311	2021-07-26	RHD XV	Gamma
EPI_ISL_5652312	2021-07-26	RHD XV	Gamma
EPI_ISL_5652313	2021-07-26	RHD XV	Gamma
EPI_ISL_5652314	2021-07-26	RHD XV	Gamma
EPI_ISL_5653801	2021-07-26	RHD XV	Gamma
EPI_ISL_5655087	2021-07-26	RHD XV	Gamma
EPI_ISL_5655071	2021-07-26	RHD XV	Gamma
EPI_ISL_5654863	2021-07-26	RHD XV	Gamma
EPI_ISL_5652319	2021-07-26	RHD XV	Gamma
EPI_ISL_5652320	2021-07-26	RHD XV	Gamma
EPI_ISL_5652330	2021-07-26	RHD XV	Gamma
EPI_ISL_5650637	2021-07-26	RHD XV	Gamma
EPI_ISL_5652343	2021-07-26	RHD XV	Gamma
EPI_ISL_5650454	2021-07-26	RHD XV	Gamma
EPI_ISL_5650605	2021-07-26	RHD XV	Gamma
EPI_ISL_5652368	2021-07-26	RHD XV	Gamma
EPI_ISL_5652375	2021-07-26	RHD XV	Gamma
EPI_ISL_5652376	2021-07-26	RHD XV	Gamma
EPI_ISL_5652377	2021-07-26	RHD XV	Gamma
EPI_ISL_5652381	2021-07-26	RHD XV	Gamma
EPI_ISL_5652398	2021-07-26	RHD XV	Gamma

EPI_ISL_5650455	2021-07-26	RHD XV	Gamma
EPI_ISL_5650606	2021-07-26	RHD XV	Gamma
EPI_ISL_5652457	2021-07-26	RHD XV	Gamma
EPI_ISL_5652486	2021-07-26	RHD XV	Gamma
EPI_ISL_5652487	2021-07-26	RHD XV	Gamma
EPI_ISL_5652488	2021-07-26	RHD XV	Gamma
EPI_ISL_5652492	2021-07-26	RHD XV	Gamma
EPI_ISL_5652499	2021-07-26	RHD XV	Gamma
EPI_ISL_5652504	2021-07-26	RHD XV	Gamma
EPI_ISL_5653871	2021-07-26	RHD XV	Gamma
EPI_ISL_5652651	2021-07-26	RHD XV	Gamma
EPI_ISL_5654905	2021-07-27	RHD XV	Gamma
EPI_ISL_5652181	2021-07-27	RHD XV	Gamma
EPI_ISL_5652209	2021-07-27	RHD XV	Gamma
EPI_ISL_5652218	2021-07-27	RHD XV	Gamma
EPI_ISL_5652263	2021-07-27	RHD XV	Gamma
EPI_ISL_5655101	2021-07-27	RHD XV	Gamma
EPI_ISL_5652282	2021-07-27	RHD XV	Gamma
EPI_ISL_5652296	2021-07-27	RHD XV	Gamma
EPI_ISL_5652413	2021-07-27	RHD XV	Gamma
EPI_ISL_5652536	2021-07-27	RHD XV	Gamma
EPI_ISL_5652753	2021-07-27	RHD XV	Gamma
EPI_ISL_5652772	2021-07-27	RHD XV	Gamma
EPI_ISL_5653215	2021-08-01	RHD XV	Gamma
EPI_ISL_5653219	2021-08-01	RHD XV	Gamma
EPI_ISL_5653228	2021-08-01	RHD XV	Gamma
EPI_ISL_5653345	2021-08-01	RHD XV	Gamma
EPI_ISL_5653347	2021-08-01	RHD XV	Gamma
EPI_ISL_5653401	2021-08-01	RHD XV	Gamma
EPI_ISL_5653420	2021-08-01	RHD XV	Gamma
EPI_ISL_3579343	2021-08-02	RHD XV	Delta
EPI_ISL_5653633	2021-08-02	RHD XV	Gamma
EPI_ISL_5654733	2021-08-02	RHD XV	Gamma
EPI_ISL_5654446	2021-08-02	RHD XV	Gamma
EPI_ISL_5654107	2021-08-02	RHD XV	Gamma
EPI_ISL_5654047	2021-08-02	RHD XV	Gamma
EPI_ISL_5654124	2021-08-02	RHD XV	Gamma
EPI_ISL_5654009	2021-08-02	RHD XV	Gamma
EPI_ISL_5654055	2021-08-02	RHD XV	Gamma
EPI_ISL_5654022	2021-08-02	RHD XV	Gamma
EPI_ISL_5653207	2021-08-02	RHD XV	Gamma
EPI_ISL_5650457	2021-08-02	RHD XV	Gamma
EPI_ISL_5650458	2021-08-02	RHD XV	Gamma
EPI_ISL_5650459	2021-08-02	RHD XV	Gamma
EPI_ISL_5650587	2021-08-02	RHD XV	Gamma
EPI_ISL_5653244	2021-08-02	RHD XV	Gamma
EPI_ISL_5653245	2021-08-02	RHD XV	Gamma
EPI_ISL_5653254	2021-08-02	RHD XV	Gamma
EPI_ISL_5653266	2021-08-02	RHD XV	Gamma
EPI_ISL_5650503	2021-08-02	RHD XV	Gamma
EPI_ISL_5653272	2021-08-02	RHD XV	Gamma
EPI_ISL_5650448	2021-08-02	RHD XV	Gamma
EPI_ISL_5653280	2021-08-02	RHD XV	Gamma
EPI_ISL_5653281	2021-08-02	RHD XV	Gamma
EPI_ISL_5653284	2021-08-02	RHD XV	Gamma
EPI_ISL_5653286	2021-08-02	RHD XV	Gamma
EPI_ISL_5653292	2021-08-02	RHD XV	Gamma
EPI_ISL_5653317	2021-08-02	RHD XV	Gamma
EPI_ISL_5653318	2021-08-02	RHD XV	Gamma
EPI_ISL_5650460	2021-08-02	RHD XV	Gamma
EPI_ISL_5653332	2021-08-02	RHD XV	Gamma
EPI_ISL_5653334	2021-08-02	RHD XV	Gamma
EPI_ISL_5653348	2021-08-02	RHD XV	Gamma

EPI_ISL_5650461	2021-08-02	RHD XV	Gamma
EPI_ISL_5653354	2021-08-02	RHD XV	Gamma
EPI_ISL_5650612	2021-08-02	RHD XV	Gamma
EPI_ISL_5653358	2021-08-02	RHD XV	Gamma
EPI_ISL_5653360	2021-08-02	RHD XV	Gamma
EPI_ISL_5653362	2021-08-02	RHD XV	Gamma
EPI_ISL_5650462	2021-08-02	RHD XV	Gamma
EPI_ISL_5653367	2021-08-02	RHD XV	Gamma
EPI_ISL_5653377	2021-08-02	RHD XV	Gamma
EPI_ISL_5650613	2021-08-02	RHD XV	Gamma
EPI_ISL_5650588	2021-08-02	RHD XV	Gamma
EPI_ISL_5653380	2021-08-02	RHD XV	Gamma
EPI_ISL_5653385	2021-08-02	RHD XV	Gamma
EPI_ISL_5650614	2021-08-02	RHD XV	Gamma
EPI_ISL_5653408	2021-08-02	RHD XV	Gamma
EPI_ISL_5653410	2021-08-02	RHD XV	Gamma
EPI_ISL_5653416	2021-08-02	RHD XV	Gamma
EPI_ISL_5653417	2021-08-02	RHD XV	Gamma
EPI_ISL_5653418	2021-08-02	RHD XV	Gamma
EPI_ISL_5653422	2021-08-02	RHD XV	Gamma
EPI_ISL_5653818	2021-08-02	RHD XV	Gamma
EPI_ISL_5655036	2021-08-02	RHD XV	Gamma
EPI_ISL_5654706	2021-08-02	RHD XV	Gamma
EPI_ISL_5654238	2021-08-02	RHD XV	Gamma
EPI_ISL_5654037	2021-08-02	RHD XV	Gamma
EPI_ISL_5653433	2021-08-02	RHD XV	Gamma
EPI_ISL_5653435	2021-08-02	RHD XV	Gamma
EPI_ISL_5653441	2021-08-02	RHD XV	Gamma
EPI_ISL_5653444	2021-08-02	RHD XV	Gamma
EPI_ISL_5653448	2021-08-02	RHD XV	Gamma
EPI_ISL_5653453	2021-08-02	RHD XV	Gamma
EPI_ISL_5653458	2021-08-02	RHD XV	Gamma
EPI_ISL_5653461	2021-08-02	RHD XV	Gamma
EPI_ISL_5653465	2021-08-02	RHD XV	Gamma
EPI_ISL_5653466	2021-08-02	RHD XV	Gamma
EPI_ISL_5653467	2021-08-02	RHD XV	Gamma
EPI_ISL_5653469	2021-08-02	RHD XV	Gamma
EPI_ISL_5653473	2021-08-02	RHD XV	Gamma
EPI_ISL_3579312	2021-08-02	RHD XV	Gamma
EPI_ISL_5654747	2021-08-03	RHD XV	Gamma
EPI_ISL_5654011	2021-08-03	RHD XV	Gamma
EPI_ISL_5653223	2021-08-03	RHD XV	Gamma
EPI_ISL_5653255	2021-08-03	RHD XV	Gamma
EPI_ISL_5653271	2021-08-03	RHD XV	Gamma
EPI_ISL_5653278	2021-08-03	RHD XV	Gamma
EPI_ISL_5653316	2021-08-03	RHD XV	Gamma
EPI_ISL_5653323	2021-08-03	RHD XV	Gamma
EPI_ISL_5653389	2021-08-03	RHD XV	Gamma
EPI_ISL_5653474	2021-08-03	RHD XV	Gamma
EPI_ISL_11940593	2021-08-08	RHD XV	Gamma
EPI_ISL_11940594	2021-08-09	RHD XV	Gamma
EPI_ISL_11940595	2021-08-09	RHD XV	Gamma
EPI_ISL_11940596	2021-08-09	RHD XV	Gamma
EPI_ISL_11940597	2021-08-09	RHD XV	Gamma
EPI_ISL_11940598	2021-08-09	RHD XV	Gamma
EPI_ISL_11940599	2021-08-09	RHD XV	Gamma
EPI_ISL_11940600	2021-08-09	RHD XV	Gamma
EPI_ISL_11940601	2021-08-09	RHD XV	Gamma
EPI_ISL_11940602	2021-08-09	RHD XV	Gamma
EPI_ISL_11940603	2021-08-09	RHD XV	Gamma
EPI_ISL_11940604	2021-08-09	RHD XV	Gamma
EPI_ISL_11940605	2021-08-09	RHD XV	Gamma
EPI_ISL_11940606	2021-08-09	RHD XV	Gamma

EPI_ISL_11940607	2021-08-09	RHD XV	Gamma
EPI_ISL_11940608	2021-08-09	RHD XV	Gamma
EPI_ISL_11940614	2021-08-09	RHD XV	Gamma
EPI_ISL_11940615	2021-08-09	RHD XV	Gamma
EPI_ISL_11940616	2021-08-09	RHD XV	Gamma
EPI_ISL_11940617	2021-08-09	RHD XV	Gamma
EPI_ISL_11940619	2021-08-09	RHD XV	Gamma
EPI_ISL_11940620	2021-08-09	RHD XV	Gamma
EPI_ISL_11940621	2021-08-09	RHD XV	Gamma
EPI_ISL_11940622	2021-08-09	RHD XV	Gamma
EPI_ISL_11940623	2021-08-09	RHD XV	Gamma
EPI_ISL_11940624	2021-08-09	RHD XV	Gamma
EPI_ISL_11940625	2021-08-09	RHD XV	Gamma
EPI_ISL_11940626	2021-08-09	RHD XV	Gamma
EPI_ISL_3761591	2021-08-09	RHD XV	Gamma
EPI_ISL_3761549	2021-08-09	RHD XV	Gamma
EPI_ISL_3761550	2021-08-09	RHD XV	Gamma
EPI_ISL_3761592	2021-08-09	RHD XV	Gamma
EPI_ISL_3761593	2021-08-09	RHD XV	Gamma
EPI_ISL_3761594	2021-08-09	RHD XV	Gamma
EPI_ISL_3761595	2021-08-09	RHD XV	Gamma
EPI_ISL_3761529	2021-08-10	RHD XV	Delta
EPI_ISL_3761619	2021-08-10	RHD XV	Delta
EPI_ISL_3761621	2021-08-10	RHD XV	Delta
EPI_ISL_11940592	2021-08-10	RHD XV	Gamma
EPI_ISL_11940609	2021-08-10	RHD XV	Gamma
EPI_ISL_11940610	2021-08-10	RHD XV	Gamma
EPI_ISL_11940611	2021-08-10	RHD XV	Gamma
EPI_ISL_11940612	2021-08-10	RHD XV	Gamma
EPI_ISL_11940613	2021-08-10	RHD XV	Gamma
EPI_ISL_3761618	2021-08-10	RHD XV	Gamma
EPI_ISL_3761620	2021-08-10	RHD XV	Gamma
EPI_ISL_3761559	2021-08-10	RHD XV	Gamma
EPI_ISL_3761566	2021-08-10	RHD XV	Gamma
EPI_ISL_3761534	2021-08-10	RHD XV	Gamma
EPI_ISL_3761622	2021-08-10	RHD XV	Gamma
EPI_ISL_3761623	2021-08-10	RHD XV	Gamma
EPI_ISL_3761635	2021-08-10	RHD XV	Gamma
EPI_ISL_3761765	2021-08-10	RHD XV	Gamma
EPI_ISL_3761538	2021-08-10	RHD XV	Gamma
EPI_ISL_3761527	2021-08-10	RHD XV	Gamma
EPI_ISL_3761766	2021-08-10	RHD XV	Gamma
EPI_ISL_3761541	2021-08-10	RHD XV	Gamma
EPI_ISL_3761625	2021-08-11	RHD XV	Delta
EPI_ISL_3761533	2021-08-11	RHD XV	Gamma
EPI_ISL_3761624	2021-08-11	RHD XV	Gamma
EPI_ISL_3761626	2021-08-11	RHD XV	Gamma
EPI_ISL_3761627	2021-08-11	RHD XV	Gamma
EPI_ISL_3761628	2021-08-11	RHD XV	Gamma
EPI_ISL_3761596	2021-08-11	RHD XV	Gamma
EPI_ISL_3761629	2021-08-11	RHD XV	Gamma
EPI_ISL_3761630	2021-08-11	RHD XV	Gamma
EPI_ISL_3761574	2021-08-11	RHD XV	Gamma
EPI_ISL_3761631	2021-08-11	RHD XV	Gamma
EPI_ISL_3761632	2021-08-11	RHD XV	Gamma
EPI_ISL_3761604	2021-08-11	RHD XV	Gamma
EPI_ISL_3761633	2021-08-11	RHD XV	Gamma
EPI_ISL_3761606	2021-08-11	RHD XV	Gamma
EPI_ISL_3761634	2021-08-11	RHD XV	Gamma
EPI_ISL_3944899	2021-08-16	RHD XV	Delta
EPI_ISL_3944866	2021-08-16	RHD XV	Delta
EPI_ISL_11940628	2021-08-16	RHD XV	Gamma
EPI_ISL_11940629	2021-08-16	RHD XV	Gamma

EPI_ISL_11940630	2021-08-16	RHD XV	Gamma
EPI_ISL_11940631	2021-08-16	RHD XV	Gamma
EPI_ISL_11940632	2021-08-16	RHD XV	Gamma
EPI_ISL_11940633	2021-08-16	RHD XV	Gamma
EPI_ISL_11940634	2021-08-16	RHD XV	Gamma
EPI_ISL_11940635	2021-08-16	RHD XV	Gamma
EPI_ISL_11940636	2021-08-16	RHD XV	Gamma
EPI_ISL_11940637	2021-08-16	RHD XV	Gamma
EPI_ISL_11940638	2021-08-16	RHD XV	Gamma
EPI_ISL_11940640	2021-08-16	RHD XV	Gamma
EPI_ISL_11940643	2021-08-16	RHD XV	Gamma
EPI_ISL_11940644	2021-08-16	RHD XV	Gamma
EPI_ISL_11940645	2021-08-16	RHD XV	Gamma
EPI_ISL_11940650	2021-08-16	RHD XV	Gamma
EPI_ISL_11940652	2021-08-16	RHD XV	Gamma
EPI_ISL_11940653	2021-08-16	RHD XV	Gamma
EPI_ISL_11940654	2021-08-16	RHD XV	Gamma
EPI_ISL_11940655	2021-08-16	RHD XV	Gamma
EPI_ISL_11940656	2021-08-16	RHD XV	Gamma
EPI_ISL_11940657	2021-08-16	RHD XV	Gamma
EPI_ISL_11940658	2021-08-16	RHD XV	Gamma
EPI_ISL_11940659	2021-08-16	RHD XV	Gamma
EPI_ISL_11940660	2021-08-16	RHD XV	Gamma
EPI_ISL_11940661	2021-08-16	RHD XV	Gamma
EPI_ISL_11940662	2021-08-16	RHD XV	Gamma
EPI_ISL_11940663	2021-08-16	RHD XV	Gamma
EPI_ISL_11940664	2021-08-16	RHD XV	Gamma
EPI_ISL_11940665	2021-08-16	RHD XV	Gamma
EPI_ISL_11940666	2021-08-16	RHD XV	Gamma
EPI_ISL_11940667	2021-08-16	RHD XV	Gamma
EPI_ISL_11940668	2021-08-16	RHD XV	Gamma
EPI_ISL_11940669	2021-08-16	RHD XV	Gamma
EPI_ISL_11940670	2021-08-16	RHD XV	Gamma
EPI_ISL_11940671	2021-08-16	RHD XV	Gamma
EPI_ISL_11940672	2021-08-16	RHD XV	Gamma
EPI_ISL_11940627	2021-08-17	RHD XV	Gamma
EPI_ISL_11940641	2021-08-17	RHD XV	Gamma
EPI_ISL_11940642	2021-08-17	RHD XV	Gamma
EPI_ISL_11940646	2021-08-17	RHD XV	Gamma
EPI_ISL_11940647	2021-08-17	RHD XV	Gamma
EPI_ISL_11940648	2021-08-17	RHD XV	Gamma
EPI_ISL_11940649	2021-08-17	RHD XV	Gamma
EPI_ISL_11940651	2021-08-17	RHD XV	Gamma
EPI_ISL_11940744	2021-08-18	RHD XV	Delta
EPI_ISL_11940694	2021-08-18	RHD XV	Gamma
EPI_ISL_11940695	2021-08-18	RHD XV	Gamma
EPI_ISL_11940696	2021-08-18	RHD XV	Gamma
EPI_ISL_11940697	2021-08-18	RHD XV	Gamma
EPI_ISL_11940698	2021-08-18	RHD XV	Gamma
EPI_ISL_11940699	2021-08-18	RHD XV	Gamma
EPI_ISL_11940700	2021-08-18	RHD XV	Gamma
EPI_ISL_11940701	2021-08-18	RHD XV	Gamma
EPI_ISL_11940702	2021-08-18	RHD XV	Gamma
EPI_ISL_11940703	2021-08-18	RHD XV	Gamma
EPI_ISL_11940704	2021-08-18	RHD XV	Gamma
EPI_ISL_11940705	2021-08-18	RHD XV	Gamma
EPI_ISL_11940706	2021-08-18	RHD XV	Gamma
EPI_ISL_11940707	2021-08-18	RHD XV	Gamma
EPI_ISL_11940708	2021-08-18	RHD XV	Gamma
EPI_ISL_11940710	2021-08-18	RHD XV	Gamma
EPI_ISL_11940711	2021-08-18	RHD XV	Gamma
EPI_ISL_11940712	2021-08-18	RHD XV	Gamma
EPI_ISL_11940713	2021-08-18	RHD XV	Gamma

EPI_ISL_11940714	2021-08-18	RHD XV	Gamma
EPI_ISL_11940727	2021-08-18	RHD XV	Gamma
EPI_ISL_11940728	2021-08-18	RHD XV	Gamma
EPI_ISL_11940729	2021-08-18	RHD XV	Gamma
EPI_ISL_11940731	2021-08-18	RHD XV	Gamma
EPI_ISL_11940732	2021-08-18	RHD XV	Gamma
EPI_ISL_11940733	2021-08-18	RHD XV	Gamma
EPI_ISL_11940734	2021-08-18	RHD XV	Gamma
EPI_ISL_11940735	2021-08-18	RHD XV	Gamma
EPI_ISL_11940736	2021-08-18	RHD XV	Gamma
EPI_ISL_11940675	2021-08-18	RHD XV	Others
EPI_ISL_11940737	2021-08-19	RHD XV	Delta
EPI_ISL_11940738	2021-08-19	RHD XV	Delta
EPI_ISL_11940739	2021-08-19	RHD XV	Delta
EPI_ISL_11940740	2021-08-19	RHD XV	Delta
EPI_ISL_11940741	2021-08-19	RHD XV	Delta
EPI_ISL_11940742	2021-08-19	RHD XV	Delta
EPI_ISL_11940743	2021-08-19	RHD XV	Delta
EPI_ISL_11940745	2021-08-19	RHD XV	Delta
EPI_ISL_11940746	2021-08-19	RHD XV	Delta
EPI_ISL_11940747	2021-08-19	RHD XV	Delta
EPI_ISL_11940676	2021-08-19	RHD XV	Gamma
EPI_ISL_11940677	2021-08-19	RHD XV	Gamma
EPI_ISL_11940678	2021-08-19	RHD XV	Gamma
EPI_ISL_11940679	2021-08-19	RHD XV	Gamma
EPI_ISL_11940680	2021-08-19	RHD XV	Gamma
EPI_ISL_11940681	2021-08-19	RHD XV	Gamma
EPI_ISL_11940682	2021-08-19	RHD XV	Gamma
EPI_ISL_11940683	2021-08-19	RHD XV	Gamma
EPI_ISL_11940684	2021-08-19	RHD XV	Gamma
EPI_ISL_11940685	2021-08-19	RHD XV	Gamma
EPI_ISL_11940686	2021-08-19	RHD XV	Gamma
EPI_ISL_11940687	2021-08-19	RHD XV	Gamma
EPI_ISL_11940688	2021-08-19	RHD XV	Gamma
EPI_ISL_11940689	2021-08-19	RHD XV	Gamma
EPI_ISL_11940690	2021-08-19	RHD XV	Gamma
EPI_ISL_11940691	2021-08-19	RHD XV	Gamma
EPI_ISL_11940692	2021-08-19	RHD XV	Gamma
EPI_ISL_11940693	2021-08-19	RHD XV	Gamma
EPI_ISL_11940709	2021-08-19	RHD XV	Gamma
EPI_ISL_11940715	2021-08-19	RHD XV	Gamma
EPI_ISL_11940716	2021-08-19	RHD XV	Gamma
EPI_ISL_11940717	2021-08-19	RHD XV	Gamma
EPI_ISL_11940718	2021-08-19	RHD XV	Gamma
EPI_ISL_11940719	2021-08-19	RHD XV	Gamma
EPI_ISL_11940720	2021-08-19	RHD XV	Gamma
EPI_ISL_11940721	2021-08-19	RHD XV	Gamma
EPI_ISL_11940722	2021-08-19	RHD XV	Gamma
EPI_ISL_11940723	2021-08-19	RHD XV	Gamma
EPI_ISL_11940724	2021-08-19	RHD XV	Gamma
EPI_ISL_11940725	2021-08-19	RHD XV	Gamma
EPI_ISL_11940726	2021-08-19	RHD XV	Gamma
EPI_ISL_11940730	2021-08-19	RHD XV	Gamma
EPI_ISL_11940673	2021-08-19	RHD XV	Others
EPI_ISL_11940674	2021-08-19	RHD XV	Others
EPI_ISL_4168740	2021-08-23	RHD XV	Delta
EPI_ISL_4168738	2021-08-23	RHD XV	Delta
EPI_ISL_4168706	2021-08-23	RHD XV	Delta
EPI_ISL_4468882	2021-08-23	RHD XV	Delta
EPI_ISL_4468880	2021-08-23	RHD XV	Delta
EPI_ISL_4468879	2021-08-23	RHD XV	Delta
EPI_ISL_4468878	2021-08-23	RHD XV	Delta
EPI_ISL_4468877	2021-08-23	RHD XV	Delta

EPI_ISL_4468881	2021-08-23	RHD XV	Delta
EPI_ISL_11940752	2021-08-23	RHD XV	Gamma
EPI_ISL_11940753	2021-08-23	RHD XV	Gamma
EPI_ISL_11940755	2021-08-23	RHD XV	Gamma
EPI_ISL_11940756	2021-08-23	RHD XV	Gamma
EPI_ISL_11940757	2021-08-23	RHD XV	Gamma
EPI_ISL_11940758	2021-08-23	RHD XV	Gamma
EPI_ISL_11940759	2021-08-23	RHD XV	Gamma
EPI_ISL_11940760	2021-08-23	RHD XV	Gamma
EPI_ISL_11940761	2021-08-23	RHD XV	Gamma
EPI_ISL_11940762	2021-08-23	RHD XV	Gamma
EPI_ISL_11940763	2021-08-23	RHD XV	Gamma
EPI_ISL_11940764	2021-08-23	RHD XV	Gamma
EPI_ISL_11940765	2021-08-23	RHD XV	Gamma
EPI_ISL_11940766	2021-08-23	RHD XV	Gamma
EPI_ISL_11940767	2021-08-23	RHD XV	Gamma
EPI_ISL_11940768	2021-08-23	RHD XV	Gamma
EPI_ISL_11940769	2021-08-23	RHD XV	Gamma
EPI_ISL_11940770	2021-08-23	RHD XV	Gamma
EPI_ISL_11940771	2021-08-23	RHD XV	Gamma
EPI_ISL_11940775	2021-08-23	RHD XV	Gamma
EPI_ISL_11940776	2021-08-23	RHD XV	Gamma
EPI_ISL_11940790	2021-08-23	RHD XV	Gamma
EPI_ISL_11940791	2021-08-23	RHD XV	Gamma
EPI_ISL_11940792	2021-08-23	RHD XV	Gamma
EPI_ISL_11940793	2021-08-23	RHD XV	Gamma
EPI_ISL_11940794	2021-08-23	RHD XV	Gamma
EPI_ISL_11940795	2021-08-23	RHD XV	Gamma
EPI_ISL_11940796	2021-08-23	RHD XV	Gamma
EPI_ISL_11940797	2021-08-23	RHD XV	Gamma
EPI_ISL_11940798	2021-08-23	RHD XV	Gamma
EPI_ISL_11940799	2021-08-23	RHD XV	Gamma
EPI_ISL_11940800	2021-08-23	RHD XV	Gamma
EPI_ISL_11940801	2021-08-23	RHD XV	Gamma
EPI_ISL_11940802	2021-08-23	RHD XV	Gamma
EPI_ISL_11940812	2021-08-23	RHD XV	Gamma
EPI_ISL_11940813	2021-08-23	RHD XV	Gamma
EPI_ISL_11940814	2021-08-23	RHD XV	Gamma
EPI_ISL_11940815	2021-08-23	RHD XV	Gamma
EPI_ISL_11940816	2021-08-23	RHD XV	Gamma
EPI_ISL_11940817	2021-08-23	RHD XV	Gamma
EPI_ISL_11940818	2021-08-23	RHD XV	Gamma
EPI_ISL_11940819	2021-08-23	RHD XV	Gamma
EPI_ISL_4468884	2021-08-24	RHD XV	Delta
EPI_ISL_4468883	2021-08-24	RHD XV	Delta
EPI_ISL_11940748	2021-08-24	RHD XV	Gamma
EPI_ISL_11940749	2021-08-24	RHD XV	Gamma
EPI_ISL_11940750	2021-08-24	RHD XV	Gamma
EPI_ISL_11940751	2021-08-24	RHD XV	Gamma
EPI_ISL_11940772	2021-08-24	RHD XV	Gamma
EPI_ISL_4468889	2021-08-25	RHD XV	Delta
EPI_ISL_4468887	2021-08-25	RHD XV	Delta
EPI_ISL_4468886	2021-08-25	RHD XV	Delta
EPI_ISL_4468885	2021-08-25	RHD XV	Delta
EPI_ISL_4468891	2021-08-25	RHD XV	Delta
EPI_ISL_4468890	2021-08-25	RHD XV	Delta
EPI_ISL_4468888	2021-08-25	RHD XV	Delta
EPI_ISL_11940773	2021-08-25	RHD XV	Gamma
EPI_ISL_11940774	2021-08-25	RHD XV	Gamma
EPI_ISL_11940777	2021-08-25	RHD XV	Gamma
EPI_ISL_11940778	2021-08-25	RHD XV	Gamma
EPI_ISL_11940779	2021-08-25	RHD XV	Gamma
EPI_ISL_11940780	2021-08-25	RHD XV	Gamma

EPI_ISL_11940781	2021-08-25	RHD XV	Gamma
EPI_ISL_11940782	2021-08-25	RHD XV	Gamma
EPI_ISL_11940783	2021-08-25	RHD XV	Gamma
EPI_ISL_11940784	2021-08-25	RHD XV	Gamma
EPI_ISL_11940785	2021-08-25	RHD XV	Gamma
EPI_ISL_11940786	2021-08-25	RHD XV	Gamma
EPI_ISL_11940787	2021-08-25	RHD XV	Gamma
EPI_ISL_11940788	2021-08-25	RHD XV	Gamma
EPI_ISL_11940789	2021-08-25	RHD XV	Gamma
EPI_ISL_11940803	2021-08-25	RHD XV	Gamma
EPI_ISL_11940804	2021-08-25	RHD XV	Gamma
EPI_ISL_11940805	2021-08-25	RHD XV	Gamma
EPI_ISL_11940806	2021-08-25	RHD XV	Gamma
EPI_ISL_11940807	2021-08-25	RHD XV	Gamma
EPI_ISL_11940808	2021-08-25	RHD XV	Gamma
EPI_ISL_11940809	2021-08-25	RHD XV	Gamma
EPI_ISL_11940810	2021-08-25	RHD XV	Gamma
EPI_ISL_11940811	2021-08-25	RHD XV	Gamma
EPI_ISL_4273252	2021-08-30	RHD XV	Delta
EPI_ISL_4275644	2021-08-30	RHD XV	Delta
EPI_ISL_4275643	2021-08-30	RHD XV	Delta
EPI_ISL_4275642	2021-08-30	RHD XV	Delta
EPI_ISL_11940830	2021-08-30	RHD XV	Gamma
EPI_ISL_11940831	2021-08-30	RHD XV	Gamma
EPI_ISL_11940832	2021-08-30	RHD XV	Gamma
EPI_ISL_11940850	2021-08-30	RHD XV	Gamma
EPI_ISL_4275645	2021-08-31	RHD XV	Delta
EPI_ISL_4275650	2021-08-31	RHD XV	Delta
EPI_ISL_4275653	2021-08-31	RHD XV	Delta
EPI_ISL_4275651	2021-08-31	RHD XV	Delta
EPI_ISL_4275652	2021-08-31	RHD XV	Delta
EPI_ISL_4275649	2021-08-31	RHD XV	Delta
EPI_ISL_4275648	2021-08-31	RHD XV	Delta
EPI_ISL_4275646	2021-08-31	RHD XV	Delta
EPI_ISL_4275647	2021-08-31	RHD XV	Delta
EPI_ISL_4275641	2021-08-31	RHD XV	Delta
EPI_ISL_4275640	2021-08-31	RHD XV	Delta
EPI_ISL_4275635	2021-08-31	RHD XV	Delta
EPI_ISL_4275629	2021-08-31	RHD XV	Delta
EPI_ISL_4275617	2021-08-31	RHD XV	Delta
EPI_ISL_4275610	2021-08-31	RHD XV	Delta
EPI_ISL_4275593	2021-08-31	RHD XV	Delta
EPI_ISL_4275592	2021-08-31	RHD XV	Delta
EPI_ISL_11940829	2021-08-31	RHD XV	Delta
EPI_ISL_11940827	2021-08-31	RHD XV	Gamma
EPI_ISL_11940828	2021-08-31	RHD XV	Gamma
EPI_ISL_11940833	2021-08-31	RHD XV	Gamma
EPI_ISL_11940834	2021-08-31	RHD XV	Gamma
EPI_ISL_11940835	2021-08-31	RHD XV	Gamma
EPI_ISL_11940836	2021-08-31	RHD XV	Gamma
EPI_ISL_11940838	2021-08-31	RHD XV	Gamma
EPI_ISL_11940839	2021-08-31	RHD XV	Gamma
EPI_ISL_11940840	2021-08-31	RHD XV	Gamma
EPI_ISL_11940844	2021-08-31	RHD XV	Gamma
EPI_ISL_11940845	2021-08-31	RHD XV	Gamma
EPI_ISL_11940846	2021-08-31	RHD XV	Gamma
EPI_ISL_11940847	2021-08-31	RHD XV	Gamma
EPI_ISL_11940848	2021-08-31	RHD XV	Gamma
EPI_ISL_11940849	2021-08-31	RHD XV	Gamma
EPI_ISL_11940851	2021-08-31	RHD XV	Gamma
EPI_ISL_4275661	2021-09-01	RHD XV	Delta
EPI_ISL_4275660	2021-09-01	RHD XV	Delta
EPI_ISL_4275659	2021-09-01	RHD XV	Delta

EPI_ISL_4275658	2021-09-01	RHD XV	Delta
EPI_ISL_4275657	2021-09-01	RHD XV	Delta
EPI_ISL_4275656	2021-09-01	RHD XV	Delta
EPI_ISL_4275655	2021-09-01	RHD XV	Delta
EPI_ISL_4275654	2021-09-01	RHD XV	Delta
EPI_ISL_4275628	2021-09-01	RHD XV	Delta
EPI_ISL_4275627	2021-09-01	RHD XV	Delta
EPI_ISL_18620411	2021-09-01	RHD XV	Delta
EPI_ISL_11940820	2021-09-01	RHD XV	Gamma
EPI_ISL_11940821	2021-09-01	RHD XV	Gamma
EPI_ISL_11940823	2021-09-01	RHD XV	Gamma
EPI_ISL_11940824	2021-09-01	RHD XV	Gamma
EPI_ISL_11940825	2021-09-01	RHD XV	Gamma
EPI_ISL_11940826	2021-09-01	RHD XV	Gamma
EPI_ISL_11940837	2021-09-01	RHD XV	Gamma
EPI_ISL_11940841	2021-09-01	RHD XV	Gamma
EPI_ISL_11940842	2021-09-01	RHD XV	Gamma
EPI_ISL_11940843	2021-09-01	RHD XV	Gamma
EPI_ISL_11940822	2021-09-01	RHD XV	Others
EPI_ISL_4746839	2021-09-04	RHD XV	Delta
EPI_ISL_4746838	2021-09-04	RHD XV	Delta
EPI_ISL_11940855	2021-09-04	RHD XV	Gamma
EPI_ISL_11940858	2021-09-04	RHD XV	Gamma
EPI_ISL_4746786	2021-09-05	RHD XV	Delta
EPI_ISL_4746534	2021-09-05	RHD XV	Delta
EPI_ISL_4746692	2021-09-05	RHD XV	Delta
EPI_ISL_4746726	2021-09-05	RHD XV	Delta
EPI_ISL_18620412	2021-09-05	RHD XV	Delta
EPI_ISL_11940857	2021-09-05	RHD XV	Gamma
EPI_ISL_11940859	2021-09-05	RHD XV	Gamma
EPI_ISL_4746703	2021-09-06	RHD XV	Delta
EPI_ISL_4747281	2021-09-06	RHD XV	Delta
EPI_ISL_4746633	2021-09-06	RHD XV	Delta
EPI_ISL_11940861	2021-09-06	RHD XV	Gamma
EPI_ISL_11940862	2021-09-06	RHD XV	Gamma
EPI_ISL_11940863	2021-09-06	RHD XV	Gamma
EPI_ISL_4746615	2021-09-07	RHD XV	Delta
EPI_ISL_4746841	2021-09-07	RHD XV	Delta
EPI_ISL_4746480	2021-09-07	RHD XV	Delta
EPI_ISL_4747285	2021-09-07	RHD XV	Delta
EPI_ISL_4747284	2021-09-07	RHD XV	Delta
EPI_ISL_4746447	2021-09-07	RHD XV	Delta
EPI_ISL_11940852	2021-09-07	RHD XV	Gamma
EPI_ISL_11940853	2021-09-07	RHD XV	Gamma
EPI_ISL_11940860	2021-09-07	RHD XV	Gamma
EPI_ISL_4746454	2021-09-08	RHD XV	Delta
EPI_ISL_4746785	2021-09-08	RHD XV	Delta
EPI_ISL_4746702	2021-09-08	RHD XV	Delta
EPI_ISL_4746595	2021-09-08	RHD XV	Delta
EPI_ISL_4746840	2021-09-08	RHD XV	Delta
EPI_ISL_4746705	2021-09-08	RHD XV	Delta
EPI_ISL_4746449	2021-09-08	RHD XV	Delta
EPI_ISL_4746679	2021-09-08	RHD XV	Delta
EPI_ISL_4746712	2021-09-08	RHD XV	Delta
EPI_ISL_4746732	2021-09-08	RHD XV	Delta
EPI_ISL_4747280	2021-09-08	RHD XV	Delta
EPI_ISL_18620413	2021-09-08	RHD XV	Delta
EPI_ISL_11940854	2021-09-08	RHD XV	Gamma
EPI_ISL_11940856	2021-09-08	RHD XV	Gamma
EPI_ISL_5021564	2021-09-11	RHD XV	Gamma
EPI_ISL_5021574	2021-09-12	RHD XV	Gamma
EPI_ISL_5021575	2021-09-12	RHD XV	Gamma
EPI_ISL_5020930	2021-09-13	RHD XV	Delta

EPI_ISL_5020945	2021-09-13	RHD XV	Delta
EPI_ISL_5020952	2021-09-13	RHD XV	Delta
EPI_ISL_5020970	2021-09-13	RHD XV	Delta
EPI_ISL_5020994	2021-09-13	RHD XV	Delta
EPI_ISL_5020996	2021-09-13	RHD XV	Delta
EPI_ISL_5020998	2021-09-13	RHD XV	Delta
EPI_ISL_5021002	2021-09-13	RHD XV	Delta
EPI_ISL_5021081	2021-09-13	RHD XV	Delta
EPI_ISL_5021094	2021-09-13	RHD XV	Delta
EPI_ISL_5021222	2021-09-13	RHD XV	Delta
EPI_ISL_5021505	2021-09-13	RHD XV	Delta
EPI_ISL_5021511	2021-09-13	RHD XV	Delta
EPI_ISL_5021517	2021-09-13	RHD XV	Delta
EPI_ISL_5021521	2021-09-13	RHD XV	Delta
EPI_ISL_5021530	2021-09-13	RHD XV	Delta
EPI_ISL_5021535	2021-09-13	RHD XV	Delta
EPI_ISL_5021539	2021-09-13	RHD XV	Delta
EPI_ISL_5021540	2021-09-13	RHD XV	Delta
EPI_ISL_5021543	2021-09-13	RHD XV	Delta
EPI_ISL_5021549	2021-09-13	RHD XV	Delta
EPI_ISL_5021582	2021-09-13	RHD XV	Delta
EPI_ISL_5049591	2021-09-13	RHD XV	Delta
EPI_ISL_5049593	2021-09-13	RHD XV	Delta
EPI_ISL_5020917	2021-09-13	RHD XV	Gamma
EPI_ISL_5020965	2021-09-13	RHD XV	Gamma
EPI_ISL_5021504	2021-09-13	RHD XV	Gamma
EPI_ISL_5021548	2021-09-13	RHD XV	Gamma
EPI_ISL_5021579	2021-09-13	RHD XV	Gamma
EPI_ISL_5021583	2021-09-13	RHD XV	Gamma
EPI_ISL_5020990	2021-09-13	RHD XV	Others
EPI_ISL_5021522	2021-09-13	RHD XV	Others
EPI_ISL_5021069	2021-09-14	RHD XV	Delta
EPI_ISL_5021071	2021-09-14	RHD XV	Delta
EPI_ISL_5021248	2021-09-14	RHD XV	Delta
EPI_ISL_5021556	2021-09-14	RHD XV	Delta
EPI_ISL_5049594	2021-09-14	RHD XV	Delta
EPI_ISL_5049595	2021-09-14	RHD XV	Delta
EPI_ISL_5049597	2021-09-14	RHD XV	Delta
EPI_ISL_5049598	2021-09-14	RHD XV	Delta
EPI_ISL_5049600	2021-09-14	RHD XV	Delta
EPI_ISL_5049601	2021-09-14	RHD XV	Delta
EPI_ISL_5021073	2021-09-14	RHD XV	Gamma
EPI_ISL_5049592	2021-09-14	RHD XV	Gamma
EPI_ISL_5049599	2021-09-14	RHD XV	Gamma
EPI_ISL_5049596	2021-09-15	RHD XV	Delta
EPI_ISL_5049603	2021-09-15	RHD XV	Delta
EPI_ISL_5049604	2021-09-15	RHD XV	Delta
EPI_ISL_5049605	2021-09-15	RHD XV	Delta
EPI_ISL_5049606	2021-09-15	RHD XV	Delta
EPI_ISL_5049608	2021-09-15	RHD XV	Delta
EPI_ISL_5049609	2021-09-15	RHD XV	Delta
EPI_ISL_5049611	2021-09-15	RHD XV	Delta
EPI_ISL_5049622	2021-09-15	RHD XV	Delta
EPI_ISL_5049623	2021-09-15	RHD XV	Delta
EPI_ISL_5049624	2021-09-15	RHD XV	Delta
EPI_ISL_5049607	2021-09-15	RHD XV	Gamma
EPI_ISL_5049610	2021-09-15	RHD XV	Gamma
EPI_ISL_5049619	2021-09-15	RHD XV	Gamma
EPI_ISL_5049620	2021-09-15	RHD XV	Gamma
EPI_ISL_5049621	2021-09-15	RHD XV	Gamma
EPI_ISL_5049602	2021-09-15	RHD XV	Others
EPI_ISL_5049612	2021-09-16	RHD XV	Delta
EPI_ISL_5049613	2021-09-16	RHD XV	Delta

EPI_ISL_5049614	2021-09-16	RHD XV	Delta
EPI_ISL_5049618	2021-09-16	RHD XV	Delta
EPI_ISL_5254491	2021-09-19	RHD XV	Gamma
EPI_ISL_5254492	2021-09-20	RHD XV	Delta
EPI_ISL_5254493	2021-09-20	RHD XV	Delta
EPI_ISL_5254494	2021-09-20	RHD XV	Delta
EPI_ISL_5254495	2021-09-20	RHD XV	Delta
EPI_ISL_5254496	2021-09-20	RHD XV	Delta
EPI_ISL_5254497	2021-09-20	RHD XV	Delta
EPI_ISL_5254498	2021-09-20	RHD XV	Delta
EPI_ISL_5254499	2021-09-20	RHD XV	Delta
EPI_ISL_5254500	2021-09-20	RHD XV	Delta
EPI_ISL_5254501	2021-09-20	RHD XV	Delta
EPI_ISL_5254502	2021-09-20	RHD XV	Delta
EPI_ISL_5254503	2021-09-20	RHD XV	Delta
EPI_ISL_5254504	2021-09-20	RHD XV	Delta
EPI_ISL_5254559	2021-09-20	RHD XV	Delta
EPI_ISL_5254564	2021-09-20	RHD XV	Delta
EPI_ISL_5254505	2021-09-20	RHD XV	Delta
EPI_ISL_5254558	2021-09-20	RHD XV	Delta
EPI_ISL_5254555	2021-09-20	RHD XV	Delta
EPI_ISL_5254556	2021-09-20	RHD XV	Delta
EPI_ISL_5254557	2021-09-20	RHD XV	Delta
EPI_ISL_7614791	2021-09-21	RHD XV	Delta
EPI_ISL_7614788	2021-09-21	RHD XV	Gamma
EPI_ISL_5254506	2021-09-22	RHD XV	Delta
EPI_ISL_5254507	2021-09-22	RHD XV	Delta
EPI_ISL_5254508	2021-09-22	RHD XV	Delta
EPI_ISL_5254509	2021-09-22	RHD XV	Delta
EPI_ISL_5254510	2021-09-22	RHD XV	Delta
EPI_ISL_5254511	2021-09-22	RHD XV	Delta
EPI_ISL_5254512	2021-09-22	RHD XV	Delta
EPI_ISL_5254513	2021-09-22	RHD XV	Delta
EPI_ISL_5254514	2021-09-22	RHD XV	Delta
EPI_ISL_5254515	2021-09-22	RHD XV	Delta
EPI_ISL_5254516	2021-09-22	RHD XV	Delta
EPI_ISL_5254517	2021-09-22	RHD XV	Delta
EPI_ISL_5254518	2021-09-22	RHD XV	Delta
EPI_ISL_5254519	2021-09-22	RHD XV	Delta
EPI_ISL_5254520	2021-09-22	RHD XV	Delta
EPI_ISL_5254521	2021-09-22	RHD XV	Delta
EPI_ISL_5254523	2021-09-22	RHD XV	Delta
EPI_ISL_5254524	2021-09-22	RHD XV	Delta
EPI_ISL_5254525	2021-09-22	RHD XV	Delta
EPI_ISL_5254565	2021-09-22	RHD XV	Delta
EPI_ISL_5254527	2021-09-22	RHD XV	Delta
EPI_ISL_5254528	2021-09-22	RHD XV	Delta
EPI_ISL_5254561	2021-09-22	RHD XV	Delta
EPI_ISL_5254563	2021-09-22	RHD XV	Delta
EPI_ISL_5254562	2021-09-22	RHD XV	Delta
EPI_ISL_5254522	2021-09-22	RHD XV	Gamma
EPI_ISL_5254526	2021-09-22	RHD XV	Gamma
EPI_ISL_7614801	2021-09-23	RHD XV	Delta
EPI_ISL_7614802	2021-09-23	RHD XV	Delta
EPI_ISL_7614795	2021-09-23	RHD XV	Delta
EPI_ISL_7614803	2021-09-23	RHD XV	Delta
EPI_ISL_5689136	2021-09-27	RHD XV	Delta
EPI_ISL_5689137	2021-09-27	RHD XV	Delta
EPI_ISL_5688395	2021-09-27	RHD XV	Delta
EPI_ISL_5689259	2021-09-27	RHD XV	Delta
EPI_ISL_5688526	2021-09-27	RHD XV	Delta
EPI_ISL_5688641	2021-09-27	RHD XV	Delta
EPI_ISL_5688651	2021-09-27	RHD XV	Delta

EPI_ISL_5689107	2021-09-27	RHD XV	Gamma
EPI_ISL_5689106	2021-09-27	RHD XV	Gamma
EPI_ISL_5689266	2021-09-27	RHD XV	Gamma
EPI_ISL_5688458	2021-09-28	RHD XV	Delta
EPI_ISL_5688468	2021-09-28	RHD XV	Delta
EPI_ISL_5688470	2021-09-28	RHD XV	Delta
EPI_ISL_7494055	2021-09-28	RHD XV	Delta
EPI_ISL_7494134	2021-09-28	RHD XV	Delta
EPI_ISL_5254529	2021-09-29	RHD XV	Delta
EPI_ISL_5254530	2021-09-29	RHD XV	Delta
EPI_ISL_5254531	2021-09-29	RHD XV	Delta
EPI_ISL_5254532	2021-09-29	RHD XV	Delta
EPI_ISL_5254533	2021-09-29	RHD XV	Delta
EPI_ISL_5254534	2021-09-29	RHD XV	Delta
EPI_ISL_5254535	2021-09-29	RHD XV	Delta
EPI_ISL_5254536	2021-09-29	RHD XV	Delta
EPI_ISL_5254537	2021-09-29	RHD XV	Delta
EPI_ISL_5254538	2021-09-29	RHD XV	Delta
EPI_ISL_5254539	2021-09-29	RHD XV	Delta
EPI_ISL_5254540	2021-09-29	RHD XV	Delta
EPI_ISL_5254541	2021-09-29	RHD XV	Delta
EPI_ISL_5254542	2021-09-29	RHD XV	Delta
EPI_ISL_5254543	2021-09-29	RHD XV	Delta
EPI_ISL_5254544	2021-09-29	RHD XV	Delta
EPI_ISL_5254545	2021-09-29	RHD XV	Delta
EPI_ISL_5254546	2021-09-29	RHD XV	Delta
EPI_ISL_5254547	2021-09-29	RHD XV	Delta
EPI_ISL_5254548	2021-09-29	RHD XV	Delta
EPI_ISL_5254560	2021-09-29	RHD XV	Delta
EPI_ISL_5254549	2021-09-29	RHD XV	Delta
EPI_ISL_5254550	2021-09-29	RHD XV	Delta
EPI_ISL_5254551	2021-09-29	RHD XV	Delta
EPI_ISL_5254552	2021-09-29	RHD XV	Delta
EPI_ISL_5254553	2021-09-29	RHD XV	Delta
EPI_ISL_5254554	2021-09-29	RHD XV	Delta
EPI_ISL_7494052	2021-09-29	RHD XV	Delta
EPI_ISL_7494151	2021-09-29	RHD XV	Delta
EPI_ISL_7614800	2021-09-30	RHD XV	Delta
EPI_ISL_7614796	2021-09-30	RHD XV	Delta
EPI_ISL_7494054	2021-09-30	RHD XV	Delta
EPI_ISL_7494130	2021-09-30	RHD XV	Delta
EPI_ISL_5898585	2021-10-03	RHD XV	Gamma
EPI_ISL_5898586	2021-10-04	RHD XV	Delta
EPI_ISL_5898815	2021-10-04	RHD XV	Delta
EPI_ISL_5898840	2021-10-04	RHD XV	Delta
EPI_ISL_5898881	2021-10-04	RHD XV	Delta
EPI_ISL_5898884	2021-10-04	RHD XV	Delta
EPI_ISL_5898888	2021-10-04	RHD XV	Delta
EPI_ISL_5898584	2021-10-04	RHD XV	Gamma
EPI_ISL_5898772	2021-10-05	RHD XV	Delta
EPI_ISL_6509911	2021-10-11	RHD XV	Delta
EPI_ISL_6510529	2021-10-11	RHD XV	Delta
EPI_ISL_6509837	2021-10-11	RHD XV	Delta
EPI_ISL_6509912	2021-10-11	RHD XV	Delta
EPI_ISL_6509592	2021-10-11	RHD XV	Delta
EPI_ISL_6509951	2021-10-11	RHD XV	Delta
EPI_ISL_6510567	2021-10-11	RHD XV	Delta
EPI_ISL_6510456	2021-10-11	RHD XV	Delta
EPI_ISL_6510517	2021-10-11	RHD XV	Delta
EPI_ISL_6510486	2021-10-11	RHD XV	Delta
EPI_ISL_6509904	2021-10-11	RHD XV	Delta
EPI_ISL_6509075	2021-10-12	RHD XV	Delta
EPI_ISL_6508692	2021-10-12	RHD XV	Delta

EPI_ISL_6509905	2021-10-12	RHD XV	Delta
EPI_ISL_6510557	2021-10-12	RHD XV	Delta
EPI_ISL_6510496	2021-10-12	RHD XV	Delta
EPI_ISL_6510447	2021-10-12	RHD XV	Delta
EPI_ISL_6508722	2021-10-13	RHD XV	Delta
EPI_ISL_6508724	2021-10-13	RHD XV	Delta
EPI_ISL_6508730	2021-10-13	RHD XV	Delta
EPI_ISL_6509937	2021-10-13	RHD XV	Delta
EPI_ISL_6510558	2021-10-18	RHD XV	Delta
EPI_ISL_6510552	2021-10-18	RHD XV	Delta
EPI_ISL_6509900	2021-10-18	RHD XV	Delta
EPI_ISL_6509972	2021-10-18	RHD XV	Delta
EPI_ISL_6509913	2021-10-18	RHD XV	Delta
EPI_ISL_6509908	2021-10-18	RHD XV	Delta
EPI_ISL_6509891	2021-10-18	RHD XV	Delta
EPI_ISL_6510572	2021-10-18	RHD XV	Delta
EPI_ISL_6510454	2021-10-18	RHD XV	Delta
EPI_ISL_6510508	2021-10-18	RHD XV	Delta
EPI_ISL_6510504	2021-10-18	RHD XV	Delta
EPI_ISL_6510480	2021-10-18	RHD XV	Delta
EPI_ISL_6510509	2021-10-18	RHD XV	Delta
EPI_ISL_6510489	2021-10-18	RHD XV	Delta
EPI_ISL_6510488	2021-10-18	RHD XV	Delta
EPI_ISL_6509857	2021-10-18	RHD XV	Delta
EPI_ISL_6509882	2021-10-18	RHD XV	Delta
EPI_ISL_6509766	2021-10-18	RHD XV	Delta
EPI_ISL_6894231	2021-10-23	RHD XV	Delta
EPI_ISL_6894232	2021-10-23	RHD XV	Delta
EPI_ISL_6894238	2021-10-23	RHD XV	Delta
EPI_ISL_6894219	2021-10-25	RHD XV	Delta
EPI_ISL_6894226	2021-10-25	RHD XV	Delta
EPI_ISL_6894242	2021-10-25	RHD XV	Delta
EPI_ISL_6894246	2021-10-25	RHD XV	Delta
EPI_ISL_6570455	2021-10-26	RHD XV	Delta
EPI_ISL_6570078	2021-10-26	RHD XV	Delta
EPI_ISL_6570106	2021-10-26	RHD XV	Delta
EPI_ISL_6570107	2021-10-26	RHD XV	Delta
EPI_ISL_6894210	2021-10-26	RHD XV	Delta
EPI_ISL_6894252	2021-10-26	RHD XV	Delta
EPI_ISL_18620414	2021-10-26	RHD XV	Delta
EPI_ISL_6570076	2021-10-27	RHD XV	Delta
EPI_ISL_6894178	2021-10-27	RHD XV	Delta
EPI_ISL_6894187	2021-10-27	RHD XV	Delta
EPI_ISL_6894190	2021-10-27	RHD XV	Delta
EPI_ISL_6894196	2021-10-27	RHD XV	Delta
EPI_ISL_6894203	2021-10-27	RHD XV	Delta
EPI_ISL_6894204	2021-10-27	RHD XV	Delta
EPI_ISL_6894214	2021-10-27	RHD XV	Delta
EPI_ISL_18620415	2021-10-27	RHD XV	Delta
EPI_ISL_18620396	2021-10-29	RHD XV	Delta
EPI_ISL_18620418	2021-10-29	RHD XV	Delta
EPI_ISL_18620419	2021-10-29	RHD XV	Delta
EPI_ISL_18620420	2021-10-29	RHD XV	Delta
EPI_ISL_18620421	2021-10-29	RHD XV	Delta
EPI_ISL_18620422	2021-10-29	RHD XV	Delta
EPI_ISL_18620416	2021-10-31	RHD XV	Delta
EPI_ISL_18620417	2021-10-31	RHD XV	Delta
EPI_ISL_6813370	2021-11-01	RHD XV	Delta
EPI_ISL_6813376	2021-11-01	RHD XV	Delta
EPI_ISL_18620397	2021-11-01	RHD XV	Delta
EPI_ISL_18620423	2021-11-01	RHD XV	Delta
EPI_ISL_18620424	2021-11-01	RHD XV	Delta
EPI_ISL_18620425	2021-11-01	RHD XV	Delta

EPI_ISL_6813261	2021-11-02	RHD XV	Delta
EPI_ISL_18620398	2021-11-02	RHD XV	Delta
EPI_ISL_18620399	2021-11-02	RHD XV	Delta
EPI_ISL_18620400	2021-11-02	RHD XV	Delta
EPI_ISL_18620426	2021-11-02	RHD XV	Delta
EPI_ISL_6813614	2021-11-03	RHD XV	Delta
EPI_ISL_6896380	2021-11-08	RHD XV	Delta
EPI_ISL_18620427	2021-11-08	RHD XV	Delta
EPI_ISL_7132225	2021-11-16	RHD XV	Delta
EPI_ISL_7132482	2021-11-16	RHD XV	Delta
EPI_ISL_18620428	2021-11-16	RHD XV	Delta
EPI_ISL_7132523	2021-11-19	RHD XV	Delta
EPI_ISL_7132711	2021-11-20	RHD XV	Delta
EPI_ISL_7132491	2021-11-21	RHD XV	Delta
EPI_ISL_7132480	2021-11-22	RHD XV	Delta
EPI_ISL_7665166	2021-11-22	RHD XV	Delta
EPI_ISL_7665165	2021-11-22	RHD XV	Delta
EPI_ISL_7665139	2021-11-22	RHD XV	Delta
EPI_ISL_7666253	2021-11-23	RHD XV	Delta
EPI_ISL_7666698	2021-11-24	RHD XV	Delta
EPI_ISL_7666523	2021-11-24	RHD XV	Delta
EPI_ISL_7666486	2021-11-25	RHD XV	Delta
EPI_ISL_18620433	2021-11-25	RHD XV	Delta
EPI_ISL_7666520	2021-11-26	RHD XV	Delta
EPI_ISL_7666647	2021-11-26	RHD XV	Delta
EPI_ISL_7666541	2021-11-26	RHD XV	Delta
EPI_ISL_7666603	2021-11-26	RHD XV	Delta
EPI_ISL_18620429	2021-11-26	RHD XV	Delta
EPI_ISL_18620430	2021-11-26	RHD XV	Delta
EPI_ISL_18620431	2021-11-26	RHD XV	Delta
EPI_ISL_18620432	2021-11-26	RHD XV	Delta
EPI_ISL_18620434	2021-11-26	RHD XV	Delta
EPI_ISL_7666458	2021-11-28	RHD XV	Delta
EPI_ISL_7809144	2021-11-29	RHD XV	Delta
EPI_ISL_7809172	2021-11-29	RHD XV	Delta
EPI_ISL_7809383	2021-11-29	RHD XV	Delta
EPI_ISL_7809386	2021-11-29	RHD XV	Delta
EPI_ISL_7809145	2021-11-30	RHD XV	Delta
EPI_ISL_7809334	2021-11-30	RHD XV	Delta
EPI_ISL_18620435	2021-11-30	RHD XV	Delta
EPI_ISL_7899761	2021-12-08	RHD XV	Omicron
EPI_ISL_18620503	2021-12-09	RHD XV	Omicron
EPI_ISL_18620402	2021-12-10	RHD XV	Delta
EPI_ISL_18620403	2021-12-12	RHD XV	Delta
EPI_ISL_8401504	2021-12-13	RHD XV	Delta
EPI_ISL_8401525	2021-12-13	RHD XV	Delta
EPI_ISL_8401538	2021-12-13	RHD XV	Delta
EPI_ISL_18620404	2021-12-13	RHD XV	Delta
EPI_ISL_18620437	2021-12-13	RHD XV	Delta
EPI_ISL_18620449	2021-12-15	RHD XV	Delta
EPI_ISL_18620409	2021-12-15	RHD XV	Delta
EPI_ISL_18620438	2021-12-17	RHD XV	Delta
EPI_ISL_18620439	2021-12-17	RHD XV	Delta
EPI_ISL_18620440	2021-12-18	RHD XV	Omicron
EPI_ISL_18620441	2021-12-20	RHD XV	Delta
EPI_ISL_18620451	2021-12-20	RHD XV	Delta
EPI_ISL_18620442	2021-12-20	RHD XV	Delta
EPI_ISL_18620443	2021-12-20	RHD XV	Omicron
EPI_ISL_18620450	2021-12-20	RHD XV	Omicron
EPI_ISL_18620452	2021-12-20	RHD XV	Omicron
EPI_ISL_18620410	2021-12-20	RHD XV	Omicron
EPI_ISL_18620453	2021-12-20	RHD XV	Omicron
EPI_ISL_18620444	2021-12-21	RHD XV	Omicron

EPI_ISL_18620445	2021-12-21	RHD XV	Omicron
EPI_ISL_18620446	2021-12-21	RHD XV	Omicron
EPI_ISL_18620447	2021-12-21	RHD XV	Omicron
EPI_ISL_18620454	2021-12-22	RHD XV	Omicron
EPI_ISL_18620455	2021-12-22	RHD XV	Omicron
EPI_ISL_18620456	2021-12-22	RHD XV	Omicron
EPI_ISL_9305188	2021-12-29	RHD XV	Omicron
EPI_ISL_18620504	2021-12-29	RHD XV	Omicron
EPI_ISL_9305178	2021-12-30	RHD XV	Omicron
EPI_ISL_9305179	2021-12-30	RHD XV	Omicron
EPI_ISL_9305180	2021-12-30	RHD XV	Omicron
EPI_ISL_9305182	2021-12-30	RHD XV	Omicron
EPI_ISL_9305183	2021-12-30	RHD XV	Omicron
EPI_ISL_9305184	2021-12-30	RHD XV	Omicron
EPI_ISL_9305181	2021-12-30	RHD XV	Omicron
EPI_ISL_9305175	2021-12-31	RHD XV	Omicron
EPI_ISL_9305176	2021-12-31	RHD XV	Omicron
EPI_ISL_9305186	2021-12-31	RHD XV	Omicron
EPI_ISL_9305187	2021-12-31	RHD XV	Omicron
EPI_ISL_9304344	2021-12-31	RHD XV	Omicron
EPI_ISL_9304397	2021-12-31	RHD XV	Omicron
EPI_ISL_9304398	2021-12-31	RHD XV	Omicron
EPI_ISL_9304399	2021-12-31	RHD XV	Omicron
EPI_ISL_9304400	2021-12-31	RHD XV	Omicron
EPI_ISL_9304401	2021-12-31	RHD XV	Omicron
EPI_ISL_9304402	2021-12-31	RHD XV	Omicron
EPI_ISL_9305185	2022-01-01	RHD XV	Omicron
EPI_ISL_9304407	2022-01-01	RHD XV	Omicron
EPI_ISL_9304423	2022-01-01	RHD XV	Omicron
EPI_ISL_9304403	2022-01-02	RHD XV	Omicron
EPI_ISL_9304404	2022-01-02	RHD XV	Omicron
EPI_ISL_9304405	2022-01-02	RHD XV	Omicron
EPI_ISL_9304408	2022-01-02	RHD XV	Omicron
EPI_ISL_9304418	2022-01-02	RHD XV	Omicron
EPI_ISL_9304419	2022-01-02	RHD XV	Omicron
EPI_ISL_9304421	2022-01-02	RHD XV	Omicron
EPI_ISL_18620436	2022-01-02	RHD XV	Omicron
EPI_ISL_18620458	2022-01-03	RHD XV	Delta
EPI_ISL_18620463	2022-01-03	RHD XV	Delta
EPI_ISL_18620471	2022-01-03	RHD XV	Delta
EPI_ISL_9304406	2022-01-03	RHD XV	Omicron
EPI_ISL_9304409	2022-01-03	RHD XV	Omicron
EPI_ISL_9304410	2022-01-03	RHD XV	Omicron
EPI_ISL_9304411	2022-01-03	RHD XV	Omicron
EPI_ISL_9304412	2022-01-03	RHD XV	Omicron
EPI_ISL_9304413	2022-01-03	RHD XV	Omicron
EPI_ISL_9304414	2022-01-03	RHD XV	Omicron
EPI_ISL_9304415	2022-01-03	RHD XV	Omicron
EPI_ISL_9304422	2022-01-03	RHD XV	Omicron
EPI_ISL_9304437	2022-01-03	RHD XV	Omicron
EPI_ISL_18620401	2022-01-03	RHD XV	Omicron
EPI_ISL_18620494	2022-01-03	RHD XV	Omicron
EPI_ISL_18620457	2022-01-03	RHD XV	Omicron
EPI_ISL_18620506	2022-01-03	RHD XV	Omicron
EPI_ISL_18620459	2022-01-03	RHD XV	Omicron
EPI_ISL_18620460	2022-01-03	RHD XV	Omicron
EPI_ISL_18620461	2022-01-03	RHD XV	Omicron
EPI_ISL_18620462	2022-01-03	RHD XV	Omicron
EPI_ISL_18620495	2022-01-03	RHD XV	Omicron
EPI_ISL_18620464	2022-01-03	RHD XV	Omicron
EPI_ISL_18620465	2022-01-03	RHD XV	Omicron
EPI_ISL_18620493	2022-01-03	RHD XV	Omicron
EPI_ISL_18620466	2022-01-03	RHD XV	Omicron

EPI_ISL_18620467	2022-01-03	RHD XV	Omicron
EPI_ISL_18620496	2022-01-03	RHD XV	Omicron
EPI_ISL_18620505	2022-01-03	RHD XV	Omicron
EPI_ISL_18620468	2022-01-03	RHD XV	Omicron
EPI_ISL_18620469	2022-01-03	RHD XV	Omicron
EPI_ISL_18620470	2022-01-03	RHD XV	Omicron
EPI_ISL_9304416	2022-01-04	RHD XV	Omicron
EPI_ISL_18620472	2022-01-05	RHD XV	Omicron
EPI_ISL_18620473	2022-01-05	RHD XV	Omicron
EPI_ISL_18620474	2022-01-05	RHD XV	Omicron
EPI_ISL_18620475	2022-01-05	RHD XV	Omicron
EPI_ISL_18620476	2022-01-05	RHD XV	Omicron
EPI_ISL_18620498	2022-01-05	RHD XV	Omicron
EPI_ISL_18620499	2022-01-05	RHD XV	Omicron
EPI_ISL_18620477	2022-01-05	RHD XV	Omicron
EPI_ISL_18620478	2022-01-05	RHD XV	Omicron
EPI_ISL_18620479	2022-01-05	RHD XV	Omicron
EPI_ISL_18620480	2022-01-05	RHD XV	Omicron
EPI_ISL_18620497	2022-01-05	RHD XV	Omicron
EPI_ISL_18620481	2022-01-05	RHD XV	Omicron
EPI_ISL_18620482	2022-01-05	RHD XV	Omicron
EPI_ISL_18620483	2022-01-05	RHD XV	Omicron
EPI_ISL_18620484	2022-01-05	RHD XV	Omicron
EPI_ISL_18620485	2022-01-05	RHD XV	Omicron
EPI_ISL_18620486	2022-01-05	RHD XV	Omicron
EPI_ISL_18620487	2022-01-05	RHD XV	Omicron
EPI_ISL_18620488	2022-01-05	RHD XV	Omicron
EPI_ISL_18620489	2022-01-05	RHD XV	Omicron
EPI_ISL_18620490	2022-01-05	RHD XV	Omicron
EPI_ISL_18620491	2022-01-05	RHD XV	Omicron
EPI_ISL_18620492	2022-01-05	RHD XV	Omicron
EPI_ISL_9304439	2022-01-06	RHD XV	Omicron
EPI_ISL_9304442	2022-01-06	RHD XV	Omicron
EPI_ISL_9304443	2022-01-06	RHD XV	Omicron
EPI_ISL_9304444	2022-01-06	RHD XV	Omicron
EPI_ISL_9304445	2022-01-06	RHD XV	Omicron
EPI_ISL_9304446	2022-01-06	RHD XV	Omicron
EPI_ISL_9304447	2022-01-06	RHD XV	Omicron
EPI_ISL_9304449	2022-01-06	RHD XV	Omicron
EPI_ISL_9304448	2022-01-06	RHD XV	Omicron
EPI_ISL_9304438	2022-01-07	RHD XV	Omicron
EPI_ISL_9304450	2022-01-09	RHD XV	Omicron
EPI_ISL_9476206	2022-01-09	RHD XV	Omicron
EPI_ISL_9304456	2022-01-10	RHD XV	Omicron
EPI_ISL_9304458	2022-01-10	RHD XV	Omicron
EPI_ISL_9304459	2022-01-10	RHD XV	Omicron
EPI_ISL_9476251	2022-01-10	RHD XV	Omicron
EPI_ISL_9476254	2022-01-10	RHD XV	Omicron
EPI_ISL_9476255	2022-01-10	RHD XV	Omicron
EPI_ISL_9476256	2022-01-10	RHD XV	Omicron
EPI_ISL_9304470	2022-01-11	RHD XV	Omicron
EPI_ISL_9304481	2022-01-11	RHD XV	Omicron
EPI_ISL_9304482	2022-01-11	RHD XV	Omicron
EPI_ISL_9304486	2022-01-11	RHD XV	Omicron
EPI_ISL_9304487	2022-01-11	RHD XV	Omicron
EPI_ISL_9304492	2022-01-11	RHD XV	Omicron
EPI_ISL_9304493	2022-01-11	RHD XV	Omicron
EPI_ISL_9304494	2022-01-11	RHD XV	Omicron
EPI_ISL_9304495	2022-01-11	RHD XV	Omicron
EPI_ISL_9304496	2022-01-11	RHD XV	Omicron
EPI_ISL_9304499	2022-01-11	RHD XV	Omicron
EPI_ISL_9304500	2022-01-11	RHD XV	Omicron
EPI_ISL_9304501	2022-01-11	RHD XV	Omicron

EPI_ISL_9304502	2022-01-11	RHD XV	Omicron
EPI_ISL_9476205	2022-01-11	RHD XV	Omicron
EPI_ISL_9476208	2022-01-11	RHD XV	Omicron
EPI_ISL_9476209	2022-01-11	RHD XV	Omicron
EPI_ISL_9476212	2022-01-11	RHD XV	Omicron
EPI_ISL_9476232	2022-01-11	RHD XV	Omicron
EPI_ISL_9476239	2022-01-11	RHD XV	Omicron
EPI_ISL_9476250	2022-01-11	RHD XV	Omicron
EPI_ISL_18620500	2022-01-11	RHD XV	Omicron
EPI_ISL_18620501	2022-01-11	RHD XV	Omicron
EPI_ISL_18620502	2022-01-11	RHD XV	Omicron
EPI_ISL_18620448	2022-01-11	RHD XV	Omicron
EPI_ISL_18620408	2022-01-11	RHD XV	Omicron
EPI_ISL_9304460	2022-01-12	RHD XV	Omicron
EPI_ISL_9304462	2022-01-12	RHD XV	Omicron
EPI_ISL_9304463	2022-01-12	RHD XV	Omicron
EPI_ISL_9304465	2022-01-12	RHD XV	Omicron
EPI_ISL_9304466	2022-01-12	RHD XV	Omicron
EPI_ISL_9304468	2022-01-12	RHD XV	Omicron
EPI_ISL_9304469	2022-01-12	RHD XV	Omicron
EPI_ISL_9304476	2022-01-12	RHD XV	Omicron
EPI_ISL_9304477	2022-01-12	RHD XV	Omicron
EPI_ISL_9304478	2022-01-12	RHD XV	Omicron
EPI_ISL_9304479	2022-01-12	RHD XV	Omicron
EPI_ISL_9304480	2022-01-12	RHD XV	Omicron
EPI_ISL_9304488	2022-01-12	RHD XV	Omicron
EPI_ISL_9304489	2022-01-12	RHD XV	Omicron
EPI_ISL_9304498	2022-01-12	RHD XV	Omicron
EPI_ISL_9476199	2022-01-12	RHD XV	Omicron
EPI_ISL_9476200	2022-01-12	RHD XV	Omicron
EPI_ISL_9476201	2022-01-12	RHD XV	Omicron
EPI_ISL_9476202	2022-01-12	RHD XV	Omicron
EPI_ISL_9476211	2022-01-12	RHD XV	Omicron
EPI_ISL_9476213	2022-01-12	RHD XV	Omicron
EPI_ISL_9476214	2022-01-12	RHD XV	Omicron
EPI_ISL_9476215	2022-01-12	RHD XV	Omicron
EPI_ISL_9476216	2022-01-12	RHD XV	Omicron
EPI_ISL_9476247	2022-01-12	RHD XV	Omicron
EPI_ISL_9304461	2022-01-13	RHD XV	Omicron
EPI_ISL_9304452	2022-01-14	RHD XV	Omicron
EPI_ISL_9304453	2022-01-14	RHD XV	Omicron
EPI_ISL_9304454	2022-01-14	RHD XV	Omicron
EPI_ISL_9304451	2022-01-14	RHD XV	Omicron
EPI_ISL_9476186	2022-01-18	RHD XV	Omicron
EPI_ISL_9476187	2022-01-18	RHD XV	Omicron
EPI_ISL_9476188	2022-01-18	RHD XV	Omicron
EPI_ISL_9476189	2022-01-18	RHD XV	Omicron
EPI_ISL_9476229	2022-01-18	RHD XV	Omicron
EPI_ISL_9476230	2022-01-18	RHD XV	Omicron
EPI_ISL_9476231	2022-01-18	RHD XV	Omicron
EPI_ISL_9476240	2022-01-18	RHD XV	Omicron
EPI_ISL_9476185	2022-01-19	RHD XV	Omicron
EPI_ISL_9476190	2022-01-19	RHD XV	Omicron
EPI_ISL_9476217	2022-01-19	RHD XV	Omicron
EPI_ISL_9476218	2022-01-19	RHD XV	Omicron
EPI_ISL_9476219	2022-01-19	RHD XV	Omicron
EPI_ISL_9476220	2022-01-19	RHD XV	Omicron
EPI_ISL_9476221	2022-01-19	RHD XV	Omicron
EPI_ISL_9476223	2022-01-19	RHD XV	Omicron
EPI_ISL_9476224	2022-01-19	RHD XV	Omicron
EPI_ISL_9476226	2022-01-19	RHD XV	Omicron
EPI_ISL_9476227	2022-01-19	RHD XV	Omicron
EPI_ISL_9476489	2022-01-19	RHD XV	Omicron

EPI_ISL_9476260	2022-01-19	RHD XV	Omicron
EPI_ISL_9476263	2022-01-19	RHD XV	Omicron
EPI_ISL_9476264	2022-01-19	RHD XV	Omicron
EPI_ISL_18620406	2022-01-19	RHD XV	Omicron
EPI_ISL_18620407	2022-01-19	RHD XV	Omicron
EPI_ISL_9476191	2022-01-20	RHD XV	Omicron
EPI_ISL_9476192	2022-01-20	RHD XV	Omicron
EPI_ISL_9476193	2022-01-20	RHD XV	Omicron
EPI_ISL_9476194	2022-01-20	RHD XV	Omicron
EPI_ISL_9476195	2022-01-20	RHD XV	Omicron
EPI_ISL_9476196	2022-01-20	RHD XV	Omicron
EPI_ISL_9476439	2022-01-20	RHD XV	Omicron
EPI_ISL_9476242	2022-01-20	RHD XV	Omicron
EPI_ISL_9476243	2022-01-20	RHD XV	Omicron
EPI_ISL_9476244	2022-01-20	RHD XV	Omicron
EPI_ISL_9805204	2022-01-27	RHD XV	Omicron
EPI_ISL_9805381	2022-01-27	RHD XV	Omicron
EPI_ISL_9805336	2022-01-27	RHD XV	Omicron
EPI_ISL_9805294	2022-01-27	RHD XV	Omicron
EPI_ISL_9805308	2022-01-27	RHD XV	Omicron
EPI_ISL_9805219	2022-01-27	RHD XV	Omicron
EPI_ISL_9805412	2022-01-27	RHD XV	Omicron
EPI_ISL_9805196	2022-01-27	RHD XV	Omicron
EPI_ISL_9805398	2022-01-27	RHD XV	Omicron
EPI_ISL_9805194	2022-01-27	RHD XV	Omicron
EPI_ISL_9805193	2022-01-27	RHD XV	Omicron
EPI_ISL_9805382	2022-01-27	RHD XV	Omicron
EPI_ISL_9805222	2022-01-27	RHD XV	Omicron
EPI_ISL_9805340	2022-01-27	RHD XV	Omicron
EPI_ISL_9805391	2022-01-27	RHD XV	Omicron
EPI_ISL_9805189	2022-01-27	RHD XV	Omicron
EPI_ISL_9805300	2022-01-27	RHD XV	Omicron
EPI_ISL_9805311	2022-01-27	RHD XV	Omicron
EPI_ISL_9805147	2022-01-27	RHD XV	Omicron
EPI_ISL_9805372	2022-01-27	RHD XV	Omicron
EPI_ISL_9805285	2022-01-27	RHD XV	Omicron
EPI_ISL_9805149	2022-01-27	RHD XV	Omicron
EPI_ISL_9805228	2022-01-27	RHD XV	Omicron
EPI_ISL_9805383	2022-01-28	RHD XV	Omicron
EPI_ISL_9805156	2022-01-28	RHD XV	Omicron
EPI_ISL_9805251	2022-01-28	RHD XV	Omicron
EPI_ISL_9805320	2022-01-28	RHD XV	Omicron
EPI_ISL_9805352	2022-01-28	RHD XV	Omicron
EPI_ISL_9805388	2022-01-28	RHD XV	Omicron
EPI_ISL_9805210	2022-01-28	RHD XV	Omicron
EPI_ISL_9805386	2022-01-28	RHD XV	Omicron
EPI_ISL_9805343	2022-01-28	RHD XV	Omicron
EPI_ISL_9805208	2022-01-28	RHD XV	Omicron
EPI_ISL_9805221	2022-01-28	RHD XV	Omicron
EPI_ISL_9805212	2022-01-28	RHD XV	Omicron
EPI_ISL_9805330	2022-01-28	RHD XV	Omicron
EPI_ISL_9805191	2022-01-28	RHD XV	Omicron
EPI_ISL_9805377	2022-01-28	RHD XV	Omicron
EPI_ISL_9805232	2022-01-28	RHD XV	Omicron
EPI_ISL_9805199	2022-01-28	RHD XV	Omicron
EPI_ISL_10309541	2022-02-01	RHD XV	Omicron
EPI_ISL_10309111	2022-02-02	RHD XV	Omicron
EPI_ISL_10309468	2022-02-02	RHD XV	Omicron
EPI_ISL_10309100	2022-02-02	RHD XV	Omicron
EPI_ISL_10309069	2022-02-02	RHD XV	Omicron
EPI_ISL_10309041	2022-02-02	RHD XV	Omicron
EPI_ISL_10309296	2022-02-02	RHD XV	Omicron
EPI_ISL_10309240	2022-02-02	RHD XV	Omicron

EPI_ISL_10309012	2022-02-03	RHD XV	Omicron
EPI_ISL_10309521	2022-02-03	RHD XV	Omicron
EPI_ISL_10309373	2022-02-03	RHD XV	Omicron
EPI_ISL_10309016	2022-02-03	RHD XV	Omicron
EPI_ISL_10309281	2022-02-03	RHD XV	Omicron
EPI_ISL_10309250	2022-02-03	RHD XV	Omicron
EPI_ISL_10309065	2022-02-03	RHD XV	Omicron
EPI_ISL_10309029	2022-02-03	RHD XV	Omicron
EPI_ISL_10309072	2022-02-03	RHD XV	Omicron
EPI_ISL_10309444	2022-02-03	RHD XV	Omicron
EPI_ISL_10309386	2022-02-03	RHD XV	Omicron
EPI_ISL_10309092	2022-02-03	RHD XV	Omicron
EPI_ISL_10309023	2022-02-03	RHD XV	Omicron
EPI_ISL_10309062	2022-02-03	RHD XV	Omicron
EPI_ISL_10309043	2022-02-03	RHD XV	Omicron
EPI_ISL_10309484	2022-02-03	RHD XV	Omicron
EPI_ISL_18620405	2022-02-03	RHD XV	Omicron
EPI_ISL_10309477	2022-02-04	RHD XV	Omicron
EPI_ISL_10309026	2022-02-04	RHD XV	Omicron
EPI_ISL_10309279	2022-02-04	RHD XV	Omicron
EPI_ISL_10309420	2022-02-04	RHD XV	Omicron
EPI_ISL_10309060	2022-02-04	RHD XV	Omicron
EPI_ISL_10309159	2022-02-04	RHD XV	Omicron
EPI_ISL_10309353	2022-02-04	RHD XV	Omicron
EPI_ISL_10309537	2022-02-04	RHD XV	Omicron
EPI_ISL_10309013	2022-02-04	RHD XV	Omicron
EPI_ISL_10309509	2022-02-04	RHD XV	Omicron
EPI_ISL_10309058	2022-02-04	RHD XV	Omicron
EPI_ISL_10309456	2022-02-04	RHD XV	Omicron
EPI_ISL_10309419	2022-02-04	RHD XV	Omicron
EPI_ISL_10309063	2022-02-04	RHD XV	Omicron
EPI_ISL_10309494	2022-02-04	RHD XV	Omicron
EPI_ISL_10309215	2022-02-04	RHD XV	Omicron
EPI_ISL_10309220	2022-02-04	RHD XV	Omicron
EPI_ISL_10309257	2022-02-04	RHD XV	Omicron
EPI_ISL_10309269	2022-02-04	RHD XV	Omicron
EPI_ISL_10309140	2022-02-04	RHD XV	Omicron
EPI_ISL_10309255	2022-02-04	RHD XV	Omicron
EPI_ISL_10309103	2022-02-04	RHD XV	Omicron
EPI_ISL_10309539	2022-02-04	RHD XV	Omicron
EPI_ISL_10309385	2022-02-05	RHD XV	Omicron
EPI_ISL_10309899	2022-02-05	RHD XV	Omicron
EPI_ISL_10309698	2022-02-05	RHD XV	Omicron
EPI_ISL_10309200	2022-02-05	RHD XV	Omicron
EPI_ISL_10309900	2022-02-05	RHD XV	Omicron
EPI_ISL_10309289	2022-02-05	RHD XV	Omicron
EPI_ISL_10309180	2022-02-05	RHD XV	Omicron
EPI_ISL_10309309	2022-02-05	RHD XV	Omicron
EPI_ISL_10309699	2022-02-05	RHD XV	Omicron
EPI_ISL_10309367	2022-02-06	RHD XV	Omicron
EPI_ISL_10309015	2022-02-06	RHD XV	Omicron
EPI_ISL_10309047	2022-02-06	RHD XV	Omicron
EPI_ISL_10309384	2022-02-06	RHD XV	Omicron
EPI_ISL_10309901	2022-02-06	RHD XV	Omicron
EPI_ISL_10309422	2022-02-07	RHD XV	Omicron
EPI_ISL_10309102	2022-02-07	RHD XV	Omicron
EPI_ISL_10309370	2022-02-07	RHD XV	Omicron
EPI_ISL_11002724	2022-02-16	RHD XV	Omicron
EPI_ISL_11002699	2022-02-16	RHD XV	Omicron
EPI_ISL_11002765	2022-02-16	RHD XV	Omicron
EPI_ISL_11002689	2022-02-16	RHD XV	Omicron
EPI_ISL_11002707	2022-02-16	RHD XV	Omicron
EPI_ISL_11002430	2022-02-17	RHD XV	Omicron

EPI_ISL_11002758	2022-02-17	RHD XV	Omicron
EPI_ISL_11002704	2022-02-17	RHD XV	Omicron
EPI_ISL_11002683	2022-02-17	RHD XV	Omicron
EPI_ISL_11002700	2022-02-17	RHD XV	Omicron
EPI_ISL_11002710	2022-02-17	RHD XV	Omicron
EPI_ISL_11002775	2022-02-17	RHD XV	Omicron
EPI_ISL_11002695	2022-02-17	RHD XV	Omicron
EPI_ISL_11002763	2022-02-17	RHD XV	Omicron
EPI_ISL_11002711	2022-02-17	RHD XV	Omicron
EPI_ISL_11002395	2022-02-17	RHD XV	Omicron
EPI_ISL_11002757	2022-02-17	RHD XV	Omicron
EPI_ISL_11002694	2022-02-17	RHD XV	Omicron
EPI_ISL_11002717	2022-02-17	RHD XV	Omicron
EPI_ISL_11002697	2022-02-17	RHD XV	Omicron
EPI_ISL_11002732	2022-02-17	RHD XV	Omicron
EPI_ISL_11002728	2022-02-17	RHD XV	Omicron
EPI_ISL_11002737	2022-02-17	RHD XV	Omicron
EPI_ISL_11002702	2022-02-17	RHD XV	Omicron
EPI_ISL_11002692	2022-02-17	RHD XV	Omicron
EPI_ISL_11002722	2022-02-17	RHD XV	Omicron
EPI_ISL_11002762	2022-02-18	RHD XV	Omicron
EPI_ISL_11002770	2022-02-18	RHD XV	Omicron
EPI_ISL_11002714	2022-02-18	RHD XV	Omicron
EPI_ISL_11002693	2022-02-18	RHD XV	Omicron
EPI_ISL_11002709	2022-02-18	RHD XV	Omicron
EPI_ISL_11002706	2022-02-18	RHD XV	Omicron
EPI_ISL_11002713	2022-02-18	RHD XV	Omicron
EPI_ISL_11002690	2022-02-18	RHD XV	Omicron
EPI_ISL_11002721	2022-02-18	RHD XV	Omicron
EPI_ISL_11002705	2022-02-18	RHD XV	Omicron
EPI_ISL_11002715	2022-02-18	RHD XV	Omicron
EPI_ISL_11002691	2022-02-18	RHD XV	Omicron
EPI_ISL_11002688	2022-02-18	RHD XV	Omicron
EPI_ISL_11002684	2022-02-18	RHD XV	Omicron
EPI_ISL_11002733	2022-02-18	RHD XV	Omicron
EPI_ISL_11002407	2022-02-18	RHD XV	Omicron
EPI_ISL_11002767	2022-02-18	RHD XV	Omicron
EPI_ISL_11002768	2022-02-18	RHD XV	Omicron
EPI_ISL_11002725	2022-02-18	RHD XV	Omicron
EPI_ISL_11002753	2022-02-18	RHD XV	Omicron
EPI_ISL_11002774	2022-02-19	RHD XV	Omicron
EPI_ISL_11002746	2022-02-19	RHD XV	Omicron
EPI_ISL_11002696	2022-02-19	RHD XV	Omicron
EPI_ISL_11002698	2022-02-19	RHD XV	Omicron
EPI_ISL_11002687	2022-02-19	RHD XV	Omicron
EPI_ISL_11002771	2022-02-19	RHD XV	Omicron
EPI_ISL_11002759	2022-02-19	RHD XV	Omicron
EPI_ISL_11027301	2022-02-20	RHD XV	Omicron
EPI_ISL_11027781	2022-02-20	RHD XV	Omicron
EPI_ISL_11027833	2022-02-20	RHD XV	Omicron
EPI_ISL_11027267	2022-02-20	RHD XV	Omicron
EPI_ISL_11027268	2022-02-20	RHD XV	Omicron
EPI_ISL_11027768	2022-02-20	RHD XV	Omicron
EPI_ISL_11027306	2022-02-20	RHD XV	Omicron
EPI_ISL_11027307	2022-02-20	RHD XV	Omicron
EPI_ISL_11027871	2022-02-20	RHD XV	Omicron
EPI_ISL_11027269	2022-02-20	RHD XV	Omicron
EPI_ISL_11027821	2022-02-20	RHD XV	Omicron
EPI_ISL_11027839	2022-02-20	RHD XV	Omicron
EPI_ISL_11027642	2022-02-20	RHD XV	Omicron
EPI_ISL_11027308	2022-02-20	RHD XV	Omicron
EPI_ISL_11027648	2022-02-21	RHD XV	Omicron
EPI_ISL_11027297	2022-02-21	RHD XV	Omicron

EPI_ISL_11027298	2022-02-21	RHD XV	Omicron
EPI_ISL_11027884	2022-02-21	RHD XV	Omicron
EPI_ISL_11027299	2022-02-21	RHD XV	Omicron
EPI_ISL_11027300	2022-02-21	RHD XV	Omicron
EPI_ISL_11027641	2022-02-21	RHD XV	Omicron
EPI_ISL_11027842	2022-02-21	RHD XV	Omicron
EPI_ISL_11027303	2022-02-21	RHD XV	Omicron
EPI_ISL_11027304	2022-02-21	RHD XV	Omicron
EPI_ISL_11027775	2022-02-21	RHD XV	Omicron
EPI_ISL_11027782	2022-02-21	RHD XV	Omicron
EPI_ISL_11027690	2022-02-21	RHD XV	Omicron
EPI_ISL_11027843	2022-02-21	RHD XV	Omicron
EPI_ISL_11027469	2022-02-21	RHD XV	Omicron
EPI_ISL_11027494	2022-02-22	RHD XV	Omicron
EPI_ISL_11027889	2022-02-22	RHD XV	Omicron
EPI_ISL_11027439	2022-02-22	RHD XV	Omicron
EPI_ISL_11027428	2022-02-22	RHD XV	Omicron
EPI_ISL_11027851	2022-02-23	RHD XV	Omicron
EPI_ISL_11027246	2022-02-24	RHD XV	Omicron
EPI_ISL_11027631	2022-02-24	RHD XV	Omicron
EPI_ISL_11027940	2022-02-24	RHD XV	Omicron
EPI_ISL_11027472	2022-02-24	RHD XV	Omicron
EPI_ISL_11027826	2022-02-24	RHD XV	Omicron
EPI_ISL_11027475	2022-02-24	RHD XV	Omicron
EPI_ISL_11027471	2022-02-24	RHD XV	Omicron
EPI_ISL_11027785	2022-02-24	RHD XV	Omicron
EPI_ISL_11027779	2022-02-24	RHD XV	Omicron
EPI_ISL_11027482	2022-02-24	RHD XV	Omicron
EPI_ISL_11027773	2022-02-24	RHD XV	Omicron
EPI_ISL_11027470	2022-02-24	RHD XV	Omicron
EPI_ISL_11027777	2022-02-24	RHD XV	Omicron
EPI_ISL_11027783	2022-02-24	RHD XV	Omicron
EPI_ISL_11027621	2022-02-24	RHD XV	Omicron
EPI_ISL_11027798	2022-02-24	RHD XV	Omicron
EPI_ISL_11027473	2022-02-24	RHD XV	Omicron
EPI_ISL_11027479	2022-02-24	RHD XV	Omicron
EPI_ISL_11027666	2022-02-24	RHD XV	Omicron
EPI_ISL_11027845	2022-02-24	RHD XV	Omicron
EPI_ISL_11027801	2022-02-24	RHD XV	Omicron
EPI_ISL_11027766	2022-02-24	RHD XV	Omicron
EPI_ISL_11027477	2022-02-24	RHD XV	Omicron
EPI_ISL_11027784	2022-02-24	RHD XV	Omicron
EPI_ISL_11027474	2022-02-24	RHD XV	Omicron
EPI_ISL_11027847	2022-02-24	RHD XV	Omicron
EPI_ISL_11027778	2022-02-24	RHD XV	Omicron
EPI_ISL_11027905	2022-02-24	RHD XV	Omicron
EPI_ISL_11027453	2022-02-24	RHD XV	Omicron
EPI_ISL_11027478	2022-02-24	RHD XV	Omicron
EPI_ISL_11027774	2022-02-25	RHD XV	Omicron
EPI_ISL_11027796	2022-02-25	RHD XV	Omicron
EPI_ISL_11027647	2022-02-25	RHD XV	Omicron
EPI_ISL_11027772	2022-02-25	RHD XV	Omicron
EPI_ISL_11027442	2022-02-25	RHD XV	Omicron
EPI_ISL_11027305	2022-02-25	RHD XV	Omicron
EPI_ISL_11027302	2022-02-26	RHD XV	Omicron
EPI_ISL_11115455	2022-02-27	RHD XV	Omicron
EPI_ISL_11115460	2022-02-27	RHD XV	Omicron
EPI_ISL_11115474	2022-02-27	RHD XV	Omicron
EPI_ISL_11115476	2022-02-27	RHD XV	Omicron
EPI_ISL_11115477	2022-02-27	RHD XV	Omicron
EPI_ISL_11115453	2022-02-28	RHD XV	Omicron
EPI_ISL_11115454	2022-02-28	RHD XV	Omicron
EPI_ISL_11115470	2022-02-28	RHD XV	Omicron

EPI_ISL_11115473	2022-02-28	RHD XV	Omicron
EPI_ISL_11115452	2022-03-01	RHD XV	Omicron
EPI_ISL_11115459	2022-03-01	RHD XV	Omicron
EPI_ISL_11115471	2022-03-01	RHD XV	Omicron
EPI_ISL_11115432	2022-03-02	RHD XV	Omicron
EPI_ISL_11115437	2022-03-02	RHD XV	Omicron
EPI_ISL_11115438	2022-03-02	RHD XV	Omicron
EPI_ISL_11115440	2022-03-02	RHD XV	Omicron
EPI_ISL_11115442	2022-03-02	RHD XV	Omicron
EPI_ISL_11115456	2022-03-02	RHD XV	Omicron
EPI_ISL_11115461	2022-03-02	RHD XV	Omicron
EPI_ISL_11115462	2022-03-02	RHD XV	Omicron
EPI_ISL_11115463	2022-03-02	RHD XV	Omicron
EPI_ISL_11115464	2022-03-02	RHD XV	Omicron
EPI_ISL_11115465	2022-03-02	RHD XV	Omicron
EPI_ISL_11115472	2022-03-02	RHD XV	Omicron
EPI_ISL_11115475	2022-03-02	RHD XV	Omicron
EPI_ISL_11115478	2022-03-02	RHD XV	Omicron
EPI_ISL_11115449	2022-03-03	RHD XV	Omicron
EPI_ISL_11115450	2022-03-03	RHD XV	Omicron
EPI_ISL_11115451	2022-03-03	RHD XV	Omicron
EPI_ISL_11115431	2022-03-04	RHD XV	Omicron
EPI_ISL_11115435	2022-03-04	RHD XV	Omicron
EPI_ISL_11115436	2022-03-04	RHD XV	Omicron
EPI_ISL_11115444	2022-03-04	RHD XV	Omicron
EPI_ISL_11115445	2022-03-04	RHD XV	Omicron
EPI_ISL_11115446	2022-03-04	RHD XV	Omicron
EPI_ISL_11115447	2022-03-04	RHD XV	Omicron
EPI_ISL_11115448	2022-03-04	RHD XV	Omicron
EPI_ISL_11115458	2022-03-04	RHD XV	Omicron
EPI_ISL_11115468	2022-03-04	RHD XV	Omicron
EPI_ISL_11115469	2022-03-04	RHD XV	Omicron
EPI_ISL_11115441	2022-03-05	RHD XV	Omicron
EPI_ISL_11115457	2022-03-05	RHD XV	Omicron
EPI_ISL_11115466	2022-03-05	RHD XV	Omicron
EPI_ISL_11412077	2022-03-06	RHD XV	Omicron
EPI_ISL_11412111	2022-03-06	RHD XV	Omicron
EPI_ISL_11412076	2022-03-07	RHD XV	Omicron
EPI_ISL_11412078	2022-03-07	RHD XV	Omicron
EPI_ISL_11412089	2022-03-07	RHD XV	Omicron
EPI_ISL_11412092	2022-03-07	RHD XV	Omicron
EPI_ISL_11412096	2022-03-07	RHD XV	Omicron
EPI_ISL_11412097	2022-03-07	RHD XV	Omicron
EPI_ISL_11412098	2022-03-07	RHD XV	Omicron
EPI_ISL_11412110	2022-03-07	RHD XV	Omicron
EPI_ISL_11412112	2022-03-07	RHD XV	Omicron
EPI_ISL_11412114	2022-03-07	RHD XV	Omicron
EPI_ISL_11412120	2022-03-07	RHD XV	Omicron
EPI_ISL_11412132	2022-03-07	RHD XV	Omicron
EPI_ISL_11412133	2022-03-07	RHD XV	Omicron
EPI_ISL_11412134	2022-03-07	RHD XV	Omicron
EPI_ISL_11412138	2022-03-07	RHD XV	Omicron
EPI_ISL_11412141	2022-03-07	RHD XV	Omicron
EPI_ISL_11412107	2022-03-08	RHD XV	Omicron
EPI_ISL_11412108	2022-03-08	RHD XV	Omicron
EPI_ISL_11412109	2022-03-08	RHD XV	Omicron
EPI_ISL_11412139	2022-03-08	RHD XV	Omicron
EPI_ISL_11412140	2022-03-08	RHD XV	Omicron
EPI_ISL_11412142	2022-03-08	RHD XV	Omicron
EPI_ISL_11412143	2022-03-08	RHD XV	Omicron
EPI_ISL_11412144	2022-03-08	RHD XV	Omicron
EPI_ISL_11412145	2022-03-08	RHD XV	Omicron
EPI_ISL_11412088	2022-03-09	RHD XV	Omicron

EPI_ISL_11412093	2022-03-09	RHD XV	Omicron
EPI_ISL_11412094	2022-03-09	RHD XV	Omicron
EPI_ISL_11412095	2022-03-09	RHD XV	Omicron
EPI_ISL_11412099	2022-03-09	RHD XV	Omicron
EPI_ISL_11412100	2022-03-09	RHD XV	Omicron
EPI_ISL_11412101	2022-03-09	RHD XV	Omicron
EPI_ISL_11412102	2022-03-09	RHD XV	Omicron
EPI_ISL_11412103	2022-03-09	RHD XV	Omicron
EPI_ISL_11412104	2022-03-09	RHD XV	Omicron
EPI_ISL_11412105	2022-03-09	RHD XV	Omicron
EPI_ISL_11412123	2022-03-09	RHD XV	Omicron
EPI_ISL_11412124	2022-03-09	RHD XV	Omicron
EPI_ISL_11412125	2022-03-09	RHD XV	Omicron
EPI_ISL_11412126	2022-03-09	RHD XV	Omicron
EPI_ISL_11412130	2022-03-09	RHD XV	Omicron
EPI_ISL_11412131	2022-03-09	RHD XV	Omicron
EPI_ISL_11412135	2022-03-09	RHD XV	Omicron
EPI_ISL_11412136	2022-03-09	RHD XV	Omicron
EPI_ISL_11412137	2022-03-09	RHD XV	Omicron
EPI_ISL_11412087	2022-03-10	RHD XV	Omicron
EPI_ISL_11412090	2022-03-11	RHD XV	Omicron
EPI_ISL_11412091	2022-03-11	RHD XV	Omicron
EPI_ISL_11412127	2022-03-11	RHD XV	Omicron
EPI_ISL_11412128	2022-03-11	RHD XV	Omicron
EPI_ISL_11412129	2022-03-11	RHD XV	Omicron
EPI_ISL_11412079	2022-03-12	RHD XV	Omicron
EPI_ISL_11412080	2022-03-12	RHD XV	Omicron
EPI_ISL_11412081	2022-03-12	RHD XV	Omicron
EPI_ISL_11412082	2022-03-12	RHD XV	Omicron
EPI_ISL_11412083	2022-03-12	RHD XV	Omicron
EPI_ISL_11412084	2022-03-12	RHD XV	Omicron
EPI_ISL_11412085	2022-03-12	RHD XV	Omicron
EPI_ISL_11412086	2022-03-12	RHD XV	Omicron
EPI_ISL_11412113	2022-03-12	RHD XV	Omicron
EPI_ISL_11412115	2022-03-12	RHD XV	Omicron
EPI_ISL_11412116	2022-03-12	RHD XV	Omicron
EPI_ISL_11412117	2022-03-12	RHD XV	Omicron
EPI_ISL_11412118	2022-03-12	RHD XV	Omicron
EPI_ISL_11412119	2022-03-12	RHD XV	Omicron
EPI_ISL_11412121	2022-03-12	RHD XV	Omicron
EPI_ISL_11412122	2022-03-12	RHD XV	Omicron
EPI_ISL_11763898	2022-03-14	RHD XV	Omicron
EPI_ISL_11763899	2022-03-14	RHD XV	Omicron
EPI_ISL_11763900	2022-03-14	RHD XV	Omicron
EPI_ISL_11763904	2022-03-14	RHD XV	Omicron
EPI_ISL_11763905	2022-03-14	RHD XV	Omicron
EPI_ISL_11763906	2022-03-14	RHD XV	Omicron
EPI_ISL_11763910	2022-03-14	RHD XV	Omicron
EPI_ISL_11763892	2022-03-15	RHD XV	Omicron
EPI_ISL_11763893	2022-03-15	RHD XV	Omicron
EPI_ISL_11763894	2022-03-15	RHD XV	Omicron
EPI_ISL_11763895	2022-03-15	RHD XV	Omicron
EPI_ISL_11763896	2022-03-15	RHD XV	Omicron
EPI_ISL_11763897	2022-03-15	RHD XV	Omicron
EPI_ISL_11763901	2022-03-15	RHD XV	Omicron
EPI_ISL_11763903	2022-03-15	RHD XV	Omicron
EPI_ISL_11763891	2022-03-16	RHD XV	Omicron
EPI_ISL_11763907	2022-03-16	RHD XV	Omicron
EPI_ISL_11763909	2022-03-16	RHD XV	Omicron
EPI_ISL_11763888	2022-03-17	RHD XV	Omicron
EPI_ISL_11763889	2022-03-17	RHD XV	Omicron
EPI_ISL_11763902	2022-03-17	RHD XV	Omicron
EPI_ISL_11763884	2022-03-19	RHD XV	Omicron

EPI_ISL_11763885	2022-03-19	RHD XV	Omicron
EPI_ISL_11763886	2022-03-19	RHD XV	Omicron
EPI_ISL_11763887	2022-03-19	RHD XV	Omicron
EPI_ISL_11763908	2022-03-19	RHD XV	Omicron
EPI_ISL_11814950	2022-03-20	RHD XV	Omicron
EPI_ISL_11814907	2022-03-21	RHD XV	Omicron
EPI_ISL_11814927	2022-03-21	RHD XV	Omicron
EPI_ISL_11814928	2022-03-21	RHD XV	Omicron
EPI_ISL_11814929	2022-03-21	RHD XV	Omicron
EPI_ISL_11814948	2022-03-21	RHD XV	Omicron
EPI_ISL_11814949	2022-03-21	RHD XV	Omicron
EPI_ISL_11814977	2022-03-21	RHD XV	Omicron
EPI_ISL_11814995	2022-03-21	RHD XV	Omicron
EPI_ISL_11814996	2022-03-21	RHD XV	Omicron
EPI_ISL_11815005	2022-03-22	RHD XV	Omicron
EPI_ISL_11814990	2022-03-23	RHD XV	Omicron
EPI_ISL_11815004	2022-03-23	RHD XV	Omicron
EPI_ISL_11815020	2022-03-23	RHD XV	Omicron
EPI_ISL_11815021	2022-03-23	RHD XV	Omicron
EPI_ISL_11814997	2022-03-24	RHD XV	Omicron
EPI_ISL_11814999	2022-03-24	RHD XV	Omicron
EPI_ISL_11815000	2022-03-24	RHD XV	Omicron
EPI_ISL_11815001	2022-03-24	RHD XV	Omicron
EPI_ISL_11815002	2022-03-24	RHD XV	Omicron
EPI_ISL_11815003	2022-03-24	RHD XV	Omicron
EPI_ISL_11814993	2022-03-26	RHD XV	Omicron
EPI_ISL_11814994	2022-03-26	RHD XV	Omicron

Supplementary Table 2. SARS-CoV-2 whole genome sequences generated in this study (RHD XV region) and retrieved from EpiC

GISAID Access Number	Collection Date	Brazilian Region Classification	Lineage Classification
NC_045512	2019-12-30	Outgroup	B
EPI_ISL_2777495	2021-01-01	North	Gamma
EPI_ISL_1181414	2021-01-01	Northeast	Others
EPI_ISL_2661782	2021-01-01	South	Others
EPI_ISL_2017318	2021-01-01	Midwest	Zeta
EPI_ISL_2308423	2021-01-01	Northeast	Zeta
EPI_ISL_2248779	2021-01-01	Northeast	Zeta
EPI_ISL_2677250	2021-01-01	South	Zeta
EPI_ISL_2245073	2021-01-02	North	Zeta
EPI_ISL_2677249	2021-01-02	South	Zeta
EPI_ISL_861684	2021-01-02	Southeast	Zeta
EPI_ISL_2249355	2021-01-03	South	B.1.1.28
EPI_ISL_2645594	2021-01-03	Southeast	B.1.1.33
EPI_ISL_2298762	2021-01-03	North	Gamma
EPI_ISL_1465274	2021-01-03	Northeast	Others
EPI_ISL_1181413	2021-01-03	Northeast	Others
EPI_ISL_2983087	2021-01-03	North	Zeta
EPI_ISL_1182575	2021-01-03	South	Zeta
EPI_ISL_2614134	2021-01-03	Southeast	Zeta
EPI_ISL_2298750	2021-01-04	Northeast	B.1.1.28
EPI_ISL_3828018	2021-01-04	Northeast	B.1.1.33
EPI_ISL_1181411	2021-01-04	South	B.1.1.33
EPI_ISL_1068259	2021-01-04	North	Zeta
EPI_ISL_1493600	2021-01-04	North	Zeta
EPI_ISL_2756453	2021-01-04	North	Zeta
EPI_ISL_2308461	2021-01-04	Northeast	Zeta
EPI_ISL_3190272	2021-01-04	Southeast	Zeta
EPI_ISL_2017309	2021-01-05	Midwest	B.1.1.28
EPI_ISL_2612348	2021-01-05	North	B.1.1.28
EPI_ISL_1493599	2021-01-05	North	B.1.1.33
EPI_ISL_1068261	2021-01-05	North	Gamma
EPI_ISL_2612347	2021-01-05	North	Others
EPI_ISL_2491772	2021-01-05	Northeast	Zeta
EPI_ISL_8005483	2021-01-05	South	Zeta
EPI_ISL_1239124	2021-01-05	Southeast	Zeta
EPI_ISL_2241609	2021-01-06	Northeast	B.1.1.28
EPI_ISL_2758712	2021-01-06	South	B.1.1.28
EPI_ISL_2425443	2021-01-06	Southeast	B.1.1.28
EPI_ISL_2298739	2021-01-07	Northeast	B.1.1.33
EPI_ISL_2298752	2021-01-07	Northeast	Gamma
EPI_ISL_1181362	2021-01-07	Northeast	Others
EPI_ISL_3434811	2021-01-07	Northeast	Zeta
EPI_ISL_15720262	2021-01-07	Northeast	Zeta
EPI_ISL_2660684	2021-01-07	Northeast	Zeta
EPI_ISL_1133261	2021-01-08	Northeast	Alpha
EPI_ISL_2612351	2021-01-08	North	B.1.1.33
EPI_ISL_1068275	2021-01-08	North	Gamma
EPI_ISL_985318	2021-01-08	South	Gamma
EPI_ISL_2983089	2021-01-08	North	Zeta
EPI_ISL_1213439	2021-01-09	Northeast	Zeta
EPI_ISL_2661794	2021-01-09	South	Zeta
EPI_ISL_2677098	2021-01-09	South	Zeta
EPI_ISL_1121316	2021-01-10	South	Gamma
EPI_ISL_2221873	2021-01-10	Northeast	Others
EPI_ISL_2645608	2021-01-10	Southeast	Zeta
EPI_ISL_1133265	2021-01-11	Southeast	Alpha
EPI_ISL_1068271	2021-01-11	North	Gamma
EPI_ISL_6208347	2021-01-11	Northeast	Others
EPI_ISL_1465268	2021-01-11	Northeast	Others
EPI_ISL_2298790	2021-01-11	North	Zeta

EPI_ISL_2758698	2021-01-11	South	Zeta
EPI_ISL_2614158	2021-01-11	Southeast	Zeta
EPI_ISL_882669	2021-01-11	Southeast	Zeta
EPI_ISL_882670	2021-01-12	Southeast	Alpha
EPI_ISL_1465267	2021-01-12	Northeast	Others
EPI_ISL_2157578	2021-01-12	Northeast	Others
EPI_ISL_2187709	2021-01-12	Midwest	Zeta
EPI_ISL_2614165	2021-01-12	Southeast	Zeta
EPI_ISL_1133272	2021-01-13	Southeast	Alpha
EPI_ISL_2187714	2021-01-13	Midwest	B.1.1.28
EPI_ISL_2677285	2021-01-13	South	Others
EPI_ISL_2821266	2021-01-13	Northeast	Zeta
EPI_ISL_2156471	2021-01-14	RHD XV	B.1.1.28
EPI_ISL_1239134	2021-01-14	Southeast	B.1.1.33
EPI_ISL_2777473	2021-01-14	North	Gamma
EPI_ISL_6840960	2021-01-14	Southeast	Others
EPI_ISL_2544854	2021-01-14	RHD XV	Zeta
EPI_ISL_2612355	2021-01-15	North	B.1.1.28
EPI_ISL_3912433	2021-01-15	Northeast	B.1.1.28
EPI_ISL_2017298	2021-01-15	Midwest	Gamma
EPI_ISL_2612407	2021-01-15	North	Gamma
EPI_ISL_2777405	2021-01-15	North	Gamma
EPI_ISL_875689	2021-01-15	Southeast	Gamma
EPI_ISL_1181401	2021-01-15	Northeast	Others
EPI_ISL_2645607	2021-01-15	Southeast	Others
EPI_ISL_2241573	2021-01-15	Northeast	Zeta
EPI_ISL_2241584	2021-01-15	Northeast	Zeta
EPI_ISL_2544855	2021-01-15	RHD XV	Zeta
EPI_ISL_2443580	2021-01-15	Southeast	Zeta
EPI_ISL_2155088	2021-01-16	RHD XV	B.1.1.28
EPI_ISL_2544848	2021-01-16	RHD XV	B.1.1.28
EPI_ISL_2544849	2021-01-16	RHD XV	B.1.1.28
EPI_ISL_2544838	2021-01-16	RHD XV	B.1.1.28
EPI_ISL_2544851	2021-01-16	RHD XV	B.1.1.28
EPI_ISL_2107304	2021-01-16	RHD XV	B.1.1.28
EPI_ISL_1785610	2021-01-16	RHD XV	B.1.1.28
EPI_ISL_2187732	2021-01-16	Midwest	B.1.1.33
EPI_ISL_2544837	2021-01-16	RHD XV	B.1.1.33
EPI_ISL_2544841	2021-01-16	RHD XV	Others
EPI_ISL_2544842	2021-01-16	RHD XV	Others
EPI_ISL_2013855	2021-01-16	RHD XV	Others
EPI_ISL_2156096	2021-01-16	RHD XV	Others
EPI_ISL_2544853	2021-01-16	RHD XV	Others
EPI_ISL_2298793	2021-01-16	North	Zeta
EPI_ISL_1785611	2021-01-16	RHD XV	Zeta
EPI_ISL_2544840	2021-01-16	RHD XV	Zeta
EPI_ISL_2543759	2021-01-16	RHD XV	Zeta
EPI_ISL_1785613	2021-01-16	RHD XV	Zeta
EPI_ISL_2013854	2021-01-16	RHD XV	Zeta
EPI_ISL_2155377	2021-01-16	RHD XV	Zeta
EPI_ISL_2155841	2021-01-16	RHD XV	Zeta
EPI_ISL_2156051	2021-01-16	RHD XV	Zeta
EPI_ISL_2544843	2021-01-16	RHD XV	Zeta
EPI_ISL_2544844	2021-01-16	RHD XV	Zeta
EPI_ISL_2544845	2021-01-16	RHD XV	Zeta
EPI_ISL_2544846	2021-01-16	RHD XV	Zeta
EPI_ISL_2544847	2021-01-16	RHD XV	Zeta
EPI_ISL_2544850	2021-01-16	RHD XV	Zeta
EPI_ISL_2156152	2021-01-16	RHD XV	Zeta
EPI_ISL_2156261	2021-01-16	RHD XV	Zeta
EPI_ISL_2544852	2021-01-16	RHD XV	Zeta
EPI_ISL_1785614	2021-01-16	RHD XV	Zeta
EPI_ISL_1785609	2021-01-16	RHD XV	Zeta

EPI_ISL_2544839	2021-01-16	RHD XV	Zeta
EPI_ISL_1533990	2021-01-17	South	Alpha
EPI_ISL_2544856	2021-01-17	RHD XV	B.1.1.28
EPI_ISL_2612358	2021-01-17	North	Gamma
EPI_ISL_2660692	2021-01-17	Northeast	Gamma
EPI_ISL_2958880	2021-01-17	Midwest	Others
EPI_ISL_2544857	2021-01-18	RHD XV	B.1.1.28
EPI_ISL_2544858	2021-01-18	RHD XV	B.1.1.28
EPI_ISL_2008939	2021-01-18	RHD XV	B.1.1.28
EPI_ISL_1293053	2021-01-18	North	Gamma
EPI_ISL_2983122	2021-01-18	North	Zeta
EPI_ISL_2249372	2021-01-18	South	Zeta
EPI_ISL_2491695	2021-01-19	Northeast	Alpha
EPI_ISL_906073	2021-01-19	Southeast	Alpha
EPI_ISL_2298797	2021-01-19	North	B.1.1.33
EPI_ISL_2661912	2021-01-19	Northeast	B.1.1.33
EPI_ISL_985317	2021-01-19	Midwest	Gamma
EPI_ISL_1303502	2021-01-19	North	Gamma
EPI_ISL_1465186	2021-01-19	Northeast	Others
EPI_ISL_940613	2021-01-19	Northeast	Others
EPI_ISL_2612360	2021-01-19	North	Zeta
EPI_ISL_2241541	2021-01-20	Northeast	Gamma
EPI_ISL_1465261	2021-01-20	Northeast	Others
EPI_ISL_3869215	2021-01-20	Northeast	Zeta
EPI_ISL_2614183	2021-01-21	Southeast	Alpha
EPI_ISL_7803802	2021-01-21	South	B.1.1.28
EPI_ISL_2344459	2021-01-21	Southeast	B.1.1.28
EPI_ISL_940626	2021-01-21	North	Gamma
EPI_ISL_3102216	2021-01-21	Northeast	Gamma
EPI_ISL_2344423	2021-01-21	South	Zeta
EPI_ISL_11681268	2021-01-22	Northeast	Gamma
EPI_ISL_1493595	2021-01-22	North	Others
EPI_ISL_1465259	2021-01-22	Northeast	Others
EPI_ISL_2491774	2021-01-22	Northeast	Zeta
EPI_ISL_2612361	2021-01-23	North	B.1.1.28
EPI_ISL_1465219	2021-01-23	Northeast	Gamma
EPI_ISL_2677104	2021-01-23	South	Zeta
EPI_ISL_2612324	2021-01-24	North	B.1.1.28
EPI_ISL_2645655	2021-01-24	Northeast	Zeta
EPI_ISL_1213388	2021-01-24	Northeast	Zeta
EPI_ISL_2544874	2021-01-25	RHD XV	B.1.1.33
EPI_ISL_940629	2021-01-25	Southeast	B.1.1.33
EPI_ISL_2614542	2021-01-25	Midwest	Gamma
EPI_ISL_2491713	2021-01-25	Northeast	Gamma
EPI_ISL_3102239	2021-01-25	Northeast	Gamma
EPI_ISL_11359367	2021-01-25	North	Zeta
EPI_ISL_2544875	2021-01-25	RHD XV	Zeta
EPI_ISL_2544862	2021-01-26	RHD XV	B.1.1.28
EPI_ISL_2544873	2021-01-26	RHD XV	B.1.1.28
EPI_ISL_2544876	2021-01-26	RHD XV	B.1.1.33
EPI_ISL_1181394	2021-01-26	South	B.1.1.33
EPI_ISL_2544864	2021-01-26	RHD XV	Gamma
EPI_ISL_2544870	2021-01-26	RHD XV	Others
EPI_ISL_2348597	2021-01-26	Midwest	Zeta
EPI_ISL_2544859	2021-01-26	RHD XV	Zeta
EPI_ISL_2544860	2021-01-26	RHD XV	Zeta
EPI_ISL_2544861	2021-01-26	RHD XV	Zeta
EPI_ISL_2544863	2021-01-26	RHD XV	Zeta
EPI_ISL_2544865	2021-01-26	RHD XV	Zeta
EPI_ISL_2544866	2021-01-26	RHD XV	Zeta
EPI_ISL_2544867	2021-01-26	RHD XV	Zeta
EPI_ISL_2544868	2021-01-26	RHD XV	Zeta
EPI_ISL_2544869	2021-01-26	RHD XV	Zeta

EPI_ISL_2544871	2021-01-26	RHD XV	Zeta
EPI_ISL_2544872	2021-01-26	RHD XV	Zeta
EPI_ISL_1533991	2021-01-26	Southeast	Zeta
EPI_ISL_1181393	2021-01-27	Southeast	B.1.1.33
EPI_ISL_2645660	2021-01-27	Northeast	Gamma
EPI_ISL_2777507	2021-01-28	North	Gamma
EPI_ISL_2245111	2021-01-28	North	Gamma
EPI_ISL_1213329	2021-01-28	Northeast	Zeta
EPI_ISL_2677097	2021-01-28	South	Zeta
EPI_ISL_2645844	2021-01-29	North	Gamma
EPI_ISL_1213312	2021-01-29	Southeast	Zeta
EPI_ISL_2756449	2021-01-30	North	Gamma
EPI_ISL_1533697	2021-01-30	Southeast	Others
EPI_ISL_2983129	2021-01-30	North	Zeta
EPI_ISL_3912419	2021-01-30	Northeast	Zeta
EPI_ISL_8005494	2021-01-30	South	Zeta
EPI_ISL_1182555	2021-01-30	Southeast	Zeta
EPI_ISL_2777509	2021-01-31	North	Gamma
EPI_ISL_11396740	2021-01-31	North	Gamma
EPI_ISL_2308441	2021-01-31	Northeast	Zeta
EPI_ISL_8005495	2021-01-31	South	Zeta
EPI_ISL_2777698	2021-02-01	North	Gamma
EPI_ISL_2298765	2021-02-01	North	Gamma
EPI_ISL_1303509	2021-02-01	North	Gamma
EPI_ISL_1213173	2021-02-01	Northeast	Gamma
EPI_ISL_4880357	2021-02-01	South	Gamma
EPI_ISL_2298772	2021-02-01	Northeast	Others
EPI_ISL_2187755	2021-02-01	Midwest	Zeta
EPI_ISL_1495041	2021-02-01	Southeast	Zeta
EPI_ISL_2614084	2021-02-01	Southeast	Zeta
EPI_ISL_2551534	2021-02-01	Southeast	Zeta
EPI_ISL_1533997	2021-02-02	South	Alpha
EPI_ISL_2777508	2021-02-02	North	Gamma
EPI_ISL_2298764	2021-02-02	North	Gamma
EPI_ISL_3912254	2021-02-02	Northeast	Gamma
EPI_ISL_2614196	2021-02-02	Southeast	Gamma
EPI_ISL_2645669	2021-02-02	Northeast	Others
EPI_ISL_12402106	2021-02-02	Northeast	Zeta
EPI_ISL_8005225	2021-02-02	South	Zeta
EPI_ISL_1121324	2021-02-02	Southeast	Zeta
EPI_ISL_2017288	2021-02-03	Midwest	Gamma
EPI_ISL_2777484	2021-02-03	North	Gamma
EPI_ISL_1465215	2021-02-03	Northeast	Gamma
EPI_ISL_1358309	2021-02-03	Midwest	Others
EPI_ISL_11396741	2021-02-03	North	Zeta
EPI_ISL_3912448	2021-02-03	Northeast	Zeta
EPI_ISL_2241598	2021-02-04	Northeast	B.1.1.33
EPI_ISL_2187764	2021-02-04	Midwest	Gamma
EPI_ISL_2660627	2021-02-04	Northeast	Gamma
EPI_ISL_2677121	2021-02-04	South	Gamma
EPI_ISL_8005227	2021-02-05	South	B.1.1.28
EPI_ISL_2241599	2021-02-05	Northeast	B.1.1.33
EPI_ISL_2612325	2021-02-05	North	Gamma
EPI_ISL_2614074	2021-02-05	Southeast	Gamma
EPI_ISL_2241508	2021-02-06	Northeast	B.1.1.28
EPI_ISL_2298758	2021-02-06	North	Gamma
EPI_ISL_1465255	2021-02-06	Northeast	Zeta
EPI_ISL_11396742	2021-02-07	North	Gamma
EPI_ISL_1465208	2021-02-07	Northeast	Gamma
EPI_ISL_3254337	2021-02-08	Midwest	Alpha
EPI_ISL_11681278	2021-02-08	Northeast	Gamma
EPI_ISL_2677134	2021-02-08	South	Gamma
EPI_ISL_3946595	2021-02-08	Midwest	Zeta

EPI_ISL_2677126	2021-02-09	South	B.1.1.28
EPI_ISL_2298778	2021-02-09	Northeast	B.1.1.33
EPI_ISL_1494924	2021-02-09	North	Gamma
EPI_ISL_2612330	2021-02-09	North	Others
EPI_ISL_2222881	2021-02-09	Midwest	Zeta
EPI_ISL_3946598	2021-02-09	Midwest	Zeta
EPI_ISL_11396746	2021-02-09	North	Zeta
EPI_ISL_1494975	2021-02-09	Southeast	Zeta
EPI_ISL_2777719	2021-02-10	North	Gamma
EPI_ISL_2241547	2021-02-10	North	Gamma
EPI_ISL_2661863	2021-02-10	South	Zeta
EPI_ISL_2614215	2021-02-10	Southeast	Zeta
EPI_ISL_2187783	2021-02-11	Midwest	B.1.1.28
EPI_ISL_2645688	2021-02-11	Northeast	B.1.1.28
EPI_ISL_2491700	2021-02-11	Northeast	Gamma
EPI_ISL_2677128	2021-02-11	South	Gamma
EPI_ISL_2645832	2021-02-12	North	Gamma
EPI_ISL_1465201	2021-02-12	Northeast	Gamma
EPI_ISL_2661843	2021-02-12	South	Gamma
EPI_ISL_1493584	2021-02-12	North	Zeta
EPI_ISL_2645697	2021-02-12	Northeast	Zeta
EPI_ISL_8005232	2021-02-13	South	Gamma
EPI_ISL_2828704	2021-02-13	Midwest	Zeta
EPI_ISL_4880331	2021-02-14	South	Zeta
EPI_ISL_3048770	2021-02-14	South	Zeta
EPI_ISL_2245108	2021-02-15	North	Gamma
EPI_ISL_2756456	2021-02-15	North	Gamma
EPI_ISL_1465250	2021-02-15	Northeast	Others
EPI_ISL_2645728	2021-02-15	Northeast	Zeta
EPI_ISL_2536305	2021-02-16	Northeast	Gamma
EPI_ISL_2777728	2021-02-17	North	Gamma
EPI_ISL_1608166	2021-02-17	Northeast	Gamma
EPI_ISL_3102240	2021-02-17	Northeast	Gamma
EPI_ISL_4600555	2021-02-17	Northeast	Gamma
EPI_ISL_2645711	2021-02-18	Northeast	B.1.1.33
EPI_ISL_2017468	2021-02-18	Midwest	Gamma
EPI_ISL_2820233	2021-02-19	Midwest	Gamma
EPI_ISL_2298821	2021-02-19	North	Gamma
EPI_ISL_2298836	2021-02-20	Northeast	B.1.1.33
EPI_ISL_2614271	2021-02-20	Southeast	B.1.1.33
EPI_ISL_2157380	2021-02-20	Northeast	Gamma
EPI_ISL_8005241	2021-02-20	South	Gamma
EPI_ISL_2551533	2021-02-20	Southeast	Others
EPI_ISL_2187824	2021-02-21	Midwest	Gamma
EPI_ISL_3102467	2021-02-21	Northeast	Gamma
EPI_ISL_8005240	2021-02-21	South	Gamma
EPI_ISL_2614274	2021-02-21	Southeast	Gamma
EPI_ISL_1533707	2021-02-21	Southeast	Gamma
EPI_ISL_2017304	2021-02-22	Midwest	Gamma
EPI_ISL_1358301	2021-02-22	North	Gamma
EPI_ISL_2663260	2021-02-22	Northeast	Gamma
EPI_ISL_2536306	2021-02-22	Northeast	Gamma
EPI_ISL_2241555	2021-02-22	Northeast	Gamma
EPI_ISL_2841607	2021-02-22	Southeast	Gamma
EPI_ISL_3912321	2021-02-23	Northeast	Gamma
EPI_ISL_1465191	2021-02-23	Northeast	Gamma
EPI_ISL_2821268	2021-02-23	Northeast	Gamma
EPI_ISL_2660617	2021-02-23	Northeast	Gamma
EPI_ISL_1358303	2021-02-23	North	Zeta
EPI_ISL_6229637	2021-02-23	Northeast	Zeta
EPI_ISL_2614079	2021-02-23	Southeast	Zeta
EPI_ISL_2249357	2021-02-24	South	Gamma
EPI_ISL_2677150	2021-02-24	South	Gamma

EPI_ISL_3835353	2021-02-24	Northeast	Zeta
EPI_ISL_3061855	2021-02-25	Southeast	Alpha
EPI_ISL_2612365	2021-02-25	North	Gamma
EPI_ISL_2612366	2021-02-25	North	Gamma
EPI_ISL_2777758	2021-02-25	North	Gamma
EPI_ISL_1493579	2021-02-25	North	Gamma
EPI_ISL_3048775	2021-02-25	South	Gamma
EPI_ISL_2274091	2021-02-26	Midwest	Gamma
EPI_ISL_3150016	2021-02-26	North	Gamma
EPI_ISL_1493578	2021-02-26	North	Gamma
EPI_ISL_2196265	2021-02-26	Northeast	Gamma
EPI_ISL_1941583	2021-02-26	RHD XV	Gamma
EPI_ISL_1494977	2021-02-26	Southeast	Gamma
EPI_ISL_2308476	2021-02-27	Northeast	Gamma
EPI_ISL_2008938	2021-02-27	RHD XV	Gamma
EPI_ISL_2983163	2021-02-28	North	Gamma
EPI_ISL_2645887	2021-02-28	North	Gamma
EPI_ISL_5529981	2021-02-28	Northeast	Gamma
EPI_ISL_2308454	2021-02-28	Northeast	Gamma
EPI_ISL_1464677	2021-03-01	Southeast	B.1.1.28
EPI_ISL_2274085	2021-03-01	Northeast	B.1.1.33
EPI_ISL_2983165	2021-03-01	North	Gamma
EPI_ISL_6573847	2021-03-01	Northeast	Gamma
EPI_ISL_8005252	2021-03-01	South	Gamma
EPI_ISL_2536265	2021-03-01	South	Gamma
EPI_ISL_3356369	2021-03-01	North	Zeta
EPI_ISL_2187829	2021-03-02	Midwest	Gamma
EPI_ISL_2777544	2021-03-02	North	Gamma
EPI_ISL_1520108	2021-03-02	North	Gamma
EPI_ISL_5529991	2021-03-02	Northeast	Gamma
EPI_ISL_2274082	2021-03-02	Northeast	Gamma
EPI_ISL_2614360	2021-03-02	Southeast	Gamma
EPI_ISL_1381068	2021-03-02	Southeast	Gamma
EPI_ISL_2157591	2021-03-02	Northeast	Zeta
EPI_ISL_13459840	2021-03-02	South	Zeta
EPI_ISL_3254345	2021-03-03	Midwest	Alpha
EPI_ISL_2614310	2021-03-03	Southeast	Alpha
EPI_ISL_3149999	2021-03-03	North	Gamma
EPI_ISL_2983169	2021-03-03	North	Gamma
EPI_ISL_2645888	2021-03-03	North	Gamma
EPI_ISL_1520109	2021-03-03	North	Gamma
EPI_ISL_1495026	2021-03-03	Southeast	Gamma
EPI_ISL_3368626	2021-03-04	North	Gamma
EPI_ISL_2157412	2021-03-04	Northeast	Gamma
EPI_ISL_5529998	2021-03-04	Northeast	Gamma
EPI_ISL_2443556	2021-03-04	Northeast	Gamma
EPI_ISL_2221885	2021-03-04	Northeast	Gamma
EPI_ISL_2629740	2021-03-04	Southeast	Gamma
EPI_ISL_1464661	2021-03-04	Southeast	Gamma
EPI_ISL_2017286	2021-03-05	Midwest	Gamma
EPI_ISL_3761750	2021-03-05	RHD XV	Gamma
EPI_ISL_3761751	2021-03-05	RHD XV	Gamma
EPI_ISL_2614089	2021-03-05	Southeast	Gamma
EPI_ISL_2274089	2021-03-05	Northeast	Others
EPI_ISL_2983172	2021-03-06	North	Gamma
EPI_ISL_2274100	2021-03-07	Southeast	Gamma
EPI_ISL_3671913	2021-03-07	Midwest	Zeta
EPI_ISL_3155972	2021-03-08	Southeast	Alpha
EPI_ISL_1785612	2021-03-08	RHD XV	B.1.1.28
EPI_ISL_2612372	2021-03-08	North	Gamma
EPI_ISL_2663295	2021-03-08	Northeast	Gamma
EPI_ISL_11681279	2021-03-08	Northeast	Gamma
EPI_ISL_1464652	2021-03-08	Southeast	Gamma

EPI_ISL_2645891	2021-03-09	North	Gamma
EPI_ISL_2274077	2021-03-09	Northeast	Gamma
EPI_ISL_2157427	2021-03-09	Northeast	Gamma
EPI_ISL_3031316	2021-03-09	Southeast	Gamma
EPI_ISL_1464650	2021-03-09	Southeast	Gamma
EPI_ISL_2187876	2021-03-10	Midwest	Gamma
EPI_ISL_2612408	2021-03-10	North	Gamma
EPI_ISL_2777799	2021-03-10	North	Gamma
EPI_ISL_2157451	2021-03-10	Northeast	Gamma
EPI_ISL_2663297	2021-03-10	Northeast	Gamma
EPI_ISL_2196263	2021-03-10	Northeast	Gamma
EPI_ISL_2274090	2021-03-10	Northeast	Gamma
EPI_ISL_2536312	2021-03-10	Northeast	Gamma
EPI_ISL_2241575	2021-03-10	Northeast	Gamma
EPI_ISL_2157454	2021-03-10	Northeast	Gamma
EPI_ISL_2139495	2021-03-10	South	Gamma
EPI_ISL_2756436	2021-03-11	North	Gamma
EPI_ISL_1754186	2021-03-11	RHD XV	Gamma
EPI_ISL_14307715	2021-03-11	Southeast	Gamma
EPI_ISL_2274083	2021-03-12	Northeast	Gamma
EPI_ISL_2536318	2021-03-12	Northeast	Gamma
EPI_ISL_8005510	2021-03-12	South	Gamma
EPI_ISL_2777620	2021-03-13	North	Gamma
EPI_ISL_2983132	2021-03-13	North	Gamma
EPI_ISL_2645518	2021-03-13	Southeast	Gamma
EPI_ISL_2557391	2021-03-13	Southeast	Gamma
EPI_ISL_12425219	2021-03-14	South	Gamma
EPI_ISL_2677149	2021-03-14	South	Gamma
EPI_ISL_3356377	2021-03-15	North	B.1.1.33
EPI_ISL_12140005	2021-03-15	North	Gamma
EPI_ISL_1464673	2021-03-15	Southeast	Zeta
EPI_ISL_2157490	2021-03-16	Northeast	Gamma
EPI_ISL_12140036	2021-03-16	Northeast	Gamma
EPI_ISL_2661768	2021-03-16	South	Gamma
EPI_ISL_12140058	2021-03-17	North	Gamma
EPI_ISL_5801938	2021-03-17	RHD XV	Gamma
EPI_ISL_5801939	2021-03-17	RHD XV	Gamma
EPI_ISL_5801940	2021-03-17	RHD XV	Gamma
EPI_ISL_5801941	2021-03-17	RHD XV	Gamma
EPI_ISL_2139496	2021-03-17	South	Gamma
EPI_ISL_1752650	2021-03-17	Southeast	Gamma
EPI_ISL_8005513	2021-03-18	South	B.1.1.28
EPI_ISL_2777713	2021-03-18	North	Gamma
EPI_ISL_11681270	2021-03-18	Northeast	Gamma
EPI_ISL_5801942	2021-03-18	RHD XV	Gamma
EPI_ISL_5801943	2021-03-18	RHD XV	Gamma
EPI_ISL_5801944	2021-03-18	RHD XV	Gamma
EPI_ISL_2443672	2021-03-18	South	Gamma
EPI_ISL_2612377	2021-03-19	North	Gamma
EPI_ISL_2612378	2021-03-19	North	Gamma
EPI_ISL_12402087	2021-03-19	Northeast	Gamma
EPI_ISL_2801327	2021-03-19	Northeast	Gamma
EPI_ISL_2196268	2021-03-19	Northeast	Gamma
EPI_ISL_2196250	2021-03-19	South	Gamma
EPI_ISL_8005292	2021-03-20	South	Alpha
EPI_ISL_3031302	2021-03-20	Southeast	Gamma
EPI_ISL_2645519	2021-03-21	Southeast	Alpha
EPI_ISL_2157508	2021-03-21	Northeast	Gamma
EPI_ISL_1795233	2021-03-21	RHD XV	Gamma
EPI_ISL_1795276	2021-03-21	RHD XV	Gamma
EPI_ISL_2536264	2021-03-21	South	Gamma
EPI_ISL_2756488	2021-03-22	Midwest	Gamma
EPI_ISL_12140002	2021-03-22	North	Gamma

EPI_ISL_1795277	2021-03-22	RHD XV	Gamma
EPI_ISL_1795279	2021-03-22	RHD XV	Gamma
EPI_ISL_1795280	2021-03-22	RHD XV	Gamma
EPI_ISL_1795281	2021-03-22	RHD XV	Gamma
EPI_ISL_1795282	2021-03-22	RHD XV	Gamma
EPI_ISL_1795283	2021-03-22	RHD XV	Gamma
EPI_ISL_1795285	2021-03-22	RHD XV	Gamma
EPI_ISL_1795286	2021-03-22	RHD XV	Gamma
EPI_ISL_2614369	2021-03-22	Southeast	Gamma
EPI_ISL_2017428	2021-03-23	Midwest	Gamma
EPI_ISL_2958886	2021-03-23	Midwest	Gamma
EPI_ISL_2612379	2021-03-23	North	Gamma
EPI_ISL_1795232	2021-03-23	RHD XV	Gamma
EPI_ISL_1795239	2021-03-23	RHD XV	Gamma
EPI_ISL_1795242	2021-03-23	RHD XV	Gamma
EPI_ISL_1795243	2021-03-23	RHD XV	Gamma
EPI_ISL_1795244	2021-03-23	RHD XV	Gamma
EPI_ISL_1795245	2021-03-23	RHD XV	Gamma
EPI_ISL_1795246	2021-03-23	RHD XV	Gamma
EPI_ISL_1795247	2021-03-23	RHD XV	Gamma
EPI_ISL_1795248	2021-03-23	RHD XV	Gamma
EPI_ISL_1795249	2021-03-23	RHD XV	Gamma
EPI_ISL_1795250	2021-03-23	RHD XV	Gamma
EPI_ISL_1795251	2021-03-23	RHD XV	Gamma
EPI_ISL_1795252	2021-03-23	RHD XV	Gamma
EPI_ISL_1795253	2021-03-23	RHD XV	Gamma
EPI_ISL_1795254	2021-03-23	RHD XV	Gamma
EPI_ISL_1795255	2021-03-23	RHD XV	Gamma
EPI_ISL_1795256	2021-03-23	RHD XV	Gamma
EPI_ISL_1795257	2021-03-23	RHD XV	Gamma
EPI_ISL_1795266	2021-03-23	RHD XV	Gamma
EPI_ISL_1795269	2021-03-23	RHD XV	Gamma
EPI_ISL_1795270	2021-03-23	RHD XV	Gamma
EPI_ISL_1795272	2021-03-23	RHD XV	Gamma
EPI_ISL_1795278	2021-03-23	RHD XV	Gamma
EPI_ISL_1795284	2021-03-23	RHD XV	Gamma
EPI_ISL_5802040	2021-03-23	RHD XV	Gamma
EPI_ISL_1731602	2021-03-23	Southeast	Gamma
EPI_ISL_1795415	2021-03-23	RHD XV	Zeta
EPI_ISL_1795079	2021-03-24	RHD XV	Alpha
EPI_ISL_6508478	2021-03-24	Southeast	B.1.1.28
EPI_ISL_1795234	2021-03-24	RHD XV	Gamma
EPI_ISL_1795235	2021-03-24	RHD XV	Gamma
EPI_ISL_1795236	2021-03-24	RHD XV	Gamma
EPI_ISL_1795237	2021-03-24	RHD XV	Gamma
EPI_ISL_1795238	2021-03-24	RHD XV	Gamma
EPI_ISL_1795240	2021-03-24	RHD XV	Gamma
EPI_ISL_1795241	2021-03-24	RHD XV	Gamma
EPI_ISL_1795389	2021-03-24	RHD XV	Gamma
EPI_ISL_1795258	2021-03-24	RHD XV	Gamma
EPI_ISL_1795259	2021-03-24	RHD XV	Gamma
EPI_ISL_1795260	2021-03-24	RHD XV	Gamma
EPI_ISL_1795261	2021-03-24	RHD XV	Gamma
EPI_ISL_1795262	2021-03-24	RHD XV	Gamma
EPI_ISL_1795263	2021-03-24	RHD XV	Gamma
EPI_ISL_1795264	2021-03-24	RHD XV	Gamma
EPI_ISL_1795265	2021-03-24	RHD XV	Gamma
EPI_ISL_1795267	2021-03-24	RHD XV	Gamma
EPI_ISL_1795268	2021-03-24	RHD XV	Gamma
EPI_ISL_1795271	2021-03-24	RHD XV	Gamma
EPI_ISL_1795273	2021-03-24	RHD XV	Gamma
EPI_ISL_1795287	2021-03-24	RHD XV	Gamma
EPI_ISL_1795288	2021-03-24	RHD XV	Gamma

EPI_ISL_1795289	2021-03-24	RHD XV	Gamma
EPI_ISL_2008940	2021-03-24	RHD XV	Gamma
EPI_ISL_1795416	2021-03-24	RHD XV	Zeta
EPI_ISL_2777795	2021-03-25	North	Gamma
EPI_ISL_2154703	2021-03-25	RHD XV	Gamma
EPI_ISL_2154956	2021-03-25	RHD XV	Gamma
EPI_ISL_3102368	2021-03-26	Northeast	Gamma
EPI_ISL_2536333	2021-03-26	Northeast	Gamma
EPI_ISL_4488044	2021-03-26	Northeast	Gamma
EPI_ISL_2274081	2021-03-26	Northeast	Others
EPI_ISL_6508480	2021-03-26	Southeast	Zeta
EPI_ISL_3691387	2021-03-27	Midwest	Gamma
EPI_ISL_13459865	2021-03-27	South	Gamma
EPI_ISL_3102531	2021-03-28	Northeast	Gamma
EPI_ISL_12425297	2021-03-28	South	Gamma
EPI_ISL_1858878	2021-03-28	Southeast	Gamma
EPI_ISL_2017477	2021-03-29	Midwest	Gamma
EPI_ISL_3368625	2021-03-29	North	Gamma
EPI_ISL_2157519	2021-03-29	Northeast	Gamma
EPI_ISL_2157520	2021-03-29	Northeast	Gamma
EPI_ISL_8630335	2021-03-29	Southeast	Gamma
EPI_ISL_5802165	2021-03-30	RHD XV	Alpha
EPI_ISL_3316173	2021-03-30	Midwest	Gamma
EPI_ISL_2663308	2021-03-30	Northeast	Gamma
EPI_ISL_5802149	2021-03-30	RHD XV	Gamma
EPI_ISL_5802150	2021-03-30	RHD XV	Gamma
EPI_ISL_5802154	2021-03-30	RHD XV	Gamma
EPI_ISL_5802160	2021-03-30	RHD XV	Gamma
EPI_ISL_5802162	2021-03-30	RHD XV	Gamma
EPI_ISL_5802166	2021-03-30	RHD XV	Gamma
EPI_ISL_5802169	2021-03-30	RHD XV	Gamma
EPI_ISL_5802170	2021-03-30	RHD XV	Gamma
EPI_ISL_5802171	2021-03-30	RHD XV	Gamma
EPI_ISL_12425177	2021-03-30	South	Gamma
EPI_ISL_5802175	2021-03-30	RHD XV	Others
EPI_ISL_5802174	2021-03-30	RHD XV	Zeta
EPI_ISL_2536327	2021-03-31	Northeast	Alpha
EPI_ISL_2017396	2021-03-31	Midwest	Gamma
EPI_ISL_2308416	2021-03-31	Northeast	Gamma
EPI_ISL_5802167	2021-03-31	RHD XV	Gamma
EPI_ISL_5802172	2021-03-31	RHD XV	Gamma
EPI_ISL_5802173	2021-03-31	RHD XV	Gamma
EPI_ISL_1731604	2021-03-31	Southeast	Gamma
EPI_ISL_3691386	2021-04-01	Midwest	Gamma
EPI_ISL_3316224	2021-04-01	Midwest	Gamma
EPI_ISL_2777609	2021-04-01	North	Gamma
EPI_ISL_2919228	2021-04-02	Midwest	Gamma
EPI_ISL_2983280	2021-04-02	Northeast	Gamma
EPI_ISL_2919230	2021-04-03	Midwest	Gamma
EPI_ISL_2777662	2021-04-03	North	Gamma
EPI_ISL_3061884	2021-04-03	Northeast	Gamma
EPI_ISL_7744032	2021-04-03	South	Gamma
EPI_ISL_3553533	2021-04-04	Southeast	Gamma
EPI_ISL_2187948	2021-04-05	Midwest	Gamma
EPI_ISL_12425170	2021-04-05	South	Gamma
EPI_ISL_3912197	2021-04-06	Northeast	Gamma
EPI_ISL_8005302	2021-04-06	South	Gamma
EPI_ISL_2157525	2021-04-06	Southeast	Gamma
EPI_ISL_2614571	2021-04-06	Southeast	Gamma
EPI_ISL_8005304	2021-04-07	South	Alpha
EPI_ISL_12140062	2021-04-07	North	Gamma
EPI_ISL_2756471	2021-04-07	North	Gamma
EPI_ISL_5800168	2021-04-07	RHD XV	Gamma

EPI_ISL_5800176	2021-04-07	RHD XV	Gamma
EPI_ISL_5800177	2021-04-07	RHD XV	Gamma
EPI_ISL_5800178	2021-04-07	RHD XV	Gamma
EPI_ISL_5800185	2021-04-07	RHD XV	Gamma
EPI_ISL_5800186	2021-04-07	RHD XV	Gamma
EPI_ISL_5800187	2021-04-07	RHD XV	Gamma
EPI_ISL_5800188	2021-04-07	RHD XV	Gamma
EPI_ISL_5800189	2021-04-07	RHD XV	Gamma
EPI_ISL_5800190	2021-04-07	RHD XV	Gamma
EPI_ISL_5800191	2021-04-07	RHD XV	Gamma
EPI_ISL_5800193	2021-04-07	RHD XV	Gamma
EPI_ISL_5800195	2021-04-07	RHD XV	Gamma
EPI_ISL_5800196	2021-04-07	RHD XV	Gamma
EPI_ISL_5800198	2021-04-07	RHD XV	Gamma
EPI_ISL_5800199	2021-04-07	RHD XV	Gamma
EPI_ISL_2551530	2021-04-07	Southeast	Gamma
EPI_ISL_5800180	2021-04-07	RHD XV	Zeta
EPI_ISL_2274054	2021-04-08	Northeast	Gamma
EPI_ISL_2466205	2021-04-08	Northeast	Gamma
EPI_ISL_5800200	2021-04-08	RHD XV	Gamma
EPI_ISL_7744025	2021-04-08	South	Gamma
EPI_ISL_2101661	2021-04-08	Southeast	Gamma
EPI_ISL_2443061	2021-04-09	RHD XV	Alpha
EPI_ISL_2777699	2021-04-09	North	Gamma
EPI_ISL_2983211	2021-04-09	Northeast	Gamma
EPI_ISL_2536345	2021-04-09	Northeast	Gamma
EPI_ISL_5530113	2021-04-09	Northeast	Gamma
EPI_ISL_2443059	2021-04-09	RHD XV	Gamma
EPI_ISL_2443058	2021-04-09	RHD XV	Gamma
EPI_ISL_2443065	2021-04-09	RHD XV	Gamma
EPI_ISL_2443060	2021-04-09	RHD XV	Gamma
EPI_ISL_2443064	2021-04-09	RHD XV	Gamma
EPI_ISL_2443063	2021-04-09	RHD XV	Gamma
EPI_ISL_2443062	2021-04-09	RHD XV	Gamma
EPI_ISL_2612343	2021-04-10	North	Gamma
EPI_ISL_6908396	2021-04-10	Northeast	Gamma
EPI_ISL_3102535	2021-04-10	Northeast	Gamma
EPI_ISL_3061887	2021-04-10	Northeast	Gamma
EPI_ISL_3048793	2021-04-10	South	Gamma
EPI_ISL_15458350	2021-04-11	North	Delta
EPI_ISL_2983258	2021-04-11	Northeast	Gamma
EPI_ISL_2536336	2021-04-11	Northeast	Gamma
EPI_ISL_2614572	2021-04-11	Southeast	Gamma
EPI_ISL_2612391	2021-04-12	North	Gamma
EPI_ISL_2612392	2021-04-12	North	Gamma
EPI_ISL_2777815	2021-04-12	North	Gamma
EPI_ISL_2101670	2021-04-12	Southeast	Gamma
EPI_ISL_2017336	2021-04-14	Midwest	Gamma
EPI_ISL_3912176	2021-04-14	Northeast	Gamma
EPI_ISL_2983261	2021-04-14	Northeast	Gamma
EPI_ISL_5800451	2021-04-14	RHD XV	Gamma
EPI_ISL_5800452	2021-04-14	RHD XV	Gamma
EPI_ISL_5800453	2021-04-14	RHD XV	Gamma
EPI_ISL_5800458	2021-04-14	RHD XV	Gamma
EPI_ISL_5800459	2021-04-14	RHD XV	Gamma
EPI_ISL_5800460	2021-04-14	RHD XV	Gamma
EPI_ISL_5800467	2021-04-14	RHD XV	Gamma
EPI_ISL_5800477	2021-04-14	RHD XV	Gamma
EPI_ISL_5800485	2021-04-14	RHD XV	Gamma
EPI_ISL_5800486	2021-04-14	RHD XV	Gamma
EPI_ISL_3048955	2021-04-14	RHD XV	Gamma
EPI_ISL_12425267	2021-04-14	South	Gamma
EPI_ISL_5800481	2021-04-15	RHD XV	Alpha

Supplementary Table 2

EPI_ISL_5800490	2021-04-15	RHD XV	B.1.1.28
EPI_ISL_2919224	2021-04-15	North	Gamma
EPI_ISL_5800466	2021-04-15	RHD XV	Gamma
EPI_ISL_5800471	2021-04-15	RHD XV	Gamma
EPI_ISL_5800474	2021-04-15	RHD XV	Gamma
EPI_ISL_5800475	2021-04-15	RHD XV	Gamma
EPI_ISL_5800478	2021-04-15	RHD XV	Gamma
EPI_ISL_5800480	2021-04-15	RHD XV	Gamma
EPI_ISL_5800482	2021-04-15	RHD XV	Gamma
EPI_ISL_5800483	2021-04-15	RHD XV	Gamma
EPI_ISL_5800484	2021-04-15	RHD XV	Gamma
EPI_ISL_5800487	2021-04-15	RHD XV	Gamma
EPI_ISL_5800489	2021-04-15	RHD XV	Gamma
EPI_ISL_5800491	2021-04-15	RHD XV	Gamma
EPI_ISL_5800492	2021-04-15	RHD XV	Gamma
EPI_ISL_5800493	2021-04-15	RHD XV	Gamma
EPI_ISL_3048954	2021-04-15	RHD XV	Gamma
EPI_ISL_2983265	2021-04-16	Northeast	Gamma
EPI_ISL_11681274	2021-04-16	Northeast	Gamma
EPI_ISL_2614580	2021-04-16	Southeast	Gamma
EPI_ISL_2157541	2021-04-17	Northeast	Gamma
EPI_ISL_2107305	2021-04-19	RHD XV	B.1.1.33
EPI_ISL_2777874	2021-04-19	North	Gamma
EPI_ISL_3048956	2021-04-19	RHD XV	Gamma
EPI_ISL_3048957	2021-04-19	RHD XV	Gamma
EPI_ISL_3761525	2021-04-19	RHD XV	Gamma
EPI_ISL_3048958	2021-04-19	RHD XV	Gamma
EPI_ISL_2008944	2021-04-19	RHD XV	Gamma
EPI_ISL_2008945	2021-04-19	RHD XV	Gamma
EPI_ISL_2008946	2021-04-19	RHD XV	Gamma
EPI_ISL_2008947	2021-04-19	RHD XV	Gamma
EPI_ISL_2008948	2021-04-19	RHD XV	Gamma
EPI_ISL_2008949	2021-04-19	RHD XV	Gamma
EPI_ISL_2008950	2021-04-19	RHD XV	Gamma
EPI_ISL_2008951	2021-04-19	RHD XV	Gamma
EPI_ISL_2107303	2021-04-19	RHD XV	Gamma
EPI_ISL_2008952	2021-04-19	RHD XV	Gamma
EPI_ISL_2008953	2021-04-19	RHD XV	Gamma
EPI_ISL_2008954	2021-04-19	RHD XV	Gamma
EPI_ISL_2008955	2021-04-19	RHD XV	Gamma
EPI_ISL_2008956	2021-04-19	RHD XV	Gamma
EPI_ISL_2008957	2021-04-19	RHD XV	Gamma
EPI_ISL_2008958	2021-04-19	RHD XV	Gamma
EPI_ISL_2008959	2021-04-19	RHD XV	Gamma
EPI_ISL_2008960	2021-04-19	RHD XV	Gamma
EPI_ISL_2008961	2021-04-19	RHD XV	Gamma
EPI_ISL_2107302	2021-04-19	RHD XV	Gamma
EPI_ISL_2008962	2021-04-19	RHD XV	Gamma
EPI_ISL_2008963	2021-04-19	RHD XV	Gamma
EPI_ISL_2008964	2021-04-19	RHD XV	Gamma
EPI_ISL_2008965	2021-04-19	RHD XV	Gamma
EPI_ISL_2008966	2021-04-19	RHD XV	Gamma
EPI_ISL_2008967	2021-04-19	RHD XV	Gamma
EPI_ISL_12425257	2021-04-19	South	Gamma
EPI_ISL_2629824	2021-04-19	Southeast	Gamma
EPI_ISL_5800757	2021-04-20	RHD XV	Alpha
EPI_ISL_5800822	2021-04-20	RHD XV	Alpha
EPI_ISL_2196242	2021-04-20	Southeast	Alpha
EPI_ISL_5800801	2021-04-20	RHD XV	B.1.1.28
EPI_ISL_2983268	2021-04-20	Northeast	Gamma
EPI_ISL_2983274	2021-04-20	Northeast	Gamma
EPI_ISL_5800759	2021-04-20	RHD XV	Gamma
EPI_ISL_5800760	2021-04-20	RHD XV	Gamma

EPI_ISL_5800779	2021-04-20	RHD XV	Gamma
EPI_ISL_5800780	2021-04-20	RHD XV	Gamma
EPI_ISL_5800791	2021-04-20	RHD XV	Gamma
EPI_ISL_5800797	2021-04-20	RHD XV	Gamma
EPI_ISL_5800813	2021-04-20	RHD XV	Gamma
EPI_ISL_5800818	2021-04-20	RHD XV	Gamma
EPI_ISL_5800821	2021-04-20	RHD XV	Gamma
EPI_ISL_5800826	2021-04-20	RHD XV	Gamma
EPI_ISL_5800827	2021-04-20	RHD XV	Gamma
EPI_ISL_5800829	2021-04-20	RHD XV	Gamma
EPI_ISL_5800831	2021-04-20	RHD XV	Gamma
EPI_ISL_5800847	2021-04-20	RHD XV	Gamma
EPI_ISL_3761749	2021-04-20	RHD XV	Gamma
EPI_ISL_2614581	2021-04-20	Southeast	Gamma
EPI_ISL_5800794	2021-04-21	RHD XV	Gamma
EPI_ISL_5800802	2021-04-21	RHD XV	Gamma
EPI_ISL_5800803	2021-04-21	RHD XV	Gamma
EPI_ISL_5800804	2021-04-21	RHD XV	Gamma
EPI_ISL_5800805	2021-04-21	RHD XV	Gamma
EPI_ISL_5800809	2021-04-21	RHD XV	Gamma
EPI_ISL_5800810	2021-04-21	RHD XV	Gamma
EPI_ISL_5800811	2021-04-21	RHD XV	Gamma
EPI_ISL_5800812	2021-04-21	RHD XV	Gamma
EPI_ISL_5800828	2021-04-21	RHD XV	Gamma
EPI_ISL_5800835	2021-04-21	RHD XV	Gamma
EPI_ISL_5800837	2021-04-21	RHD XV	Gamma
EPI_ISL_5800844	2021-04-21	RHD XV	Gamma
EPI_ISL_5800849	2021-04-21	RHD XV	Gamma
EPI_ISL_5800792	2021-04-21	RHD XV	Others
EPI_ISL_5800793	2021-04-21	RHD XV	Others
EPI_ISL_6908434	2021-04-22	Northeast	Gamma
EPI_ISL_2983304	2021-04-22	Northeast	Gamma
EPI_ISL_5800814	2021-04-22	RHD XV	Gamma
EPI_ISL_5800816	2021-04-22	RHD XV	Gamma
EPI_ISL_5800823	2021-04-22	RHD XV	Gamma
EPI_ISL_5800824	2021-04-22	RHD XV	Gamma
EPI_ISL_5800825	2021-04-22	RHD XV	Gamma
EPI_ISL_5800830	2021-04-22	RHD XV	Gamma
EPI_ISL_5800833	2021-04-22	RHD XV	Gamma
EPI_ISL_5800834	2021-04-22	RHD XV	Gamma
EPI_ISL_5800836	2021-04-22	RHD XV	Gamma
EPI_ISL_5800838	2021-04-22	RHD XV	Gamma
EPI_ISL_5800839	2021-04-22	RHD XV	Gamma
EPI_ISL_5800841	2021-04-22	RHD XV	Gamma
EPI_ISL_5800842	2021-04-22	RHD XV	Gamma
EPI_ISL_5800850	2021-04-22	RHD XV	Gamma
EPI_ISL_5800853	2021-04-22	RHD XV	Gamma
EPI_ISL_2196333	2021-04-22	Southeast	Gamma
EPI_ISL_5800819	2021-04-22	RHD XV	Others
EPI_ISL_4030335	2021-04-23	North	Gamma
EPI_ISL_3102513	2021-04-23	Northeast	Gamma
EPI_ISL_2983276	2021-04-23	Northeast	Gamma
EPI_ISL_5926861	2021-04-24	North	Gamma
EPI_ISL_5926862	2021-04-24	North	Gamma
EPI_ISL_2466246	2021-04-24	Northeast	Gamma
EPI_ISL_3061882	2021-04-24	Northeast	Gamma
EPI_ISL_2017430	2021-04-26	Midwest	Alpha
EPI_ISL_2444156	2021-04-26	RHD XV	Alpha
EPI_ISL_12140007	2021-04-26	North	Gamma
EPI_ISL_2983285	2021-04-26	Northeast	Gamma
EPI_ISL_4488032	2021-04-26	Northeast	Gamma
EPI_ISL_2444168	2021-04-26	RHD XV	Gamma
EPI_ISL_2444158	2021-04-26	RHD XV	Gamma

EPI_ISL_2444160	2021-04-26	RHD XV	Gamma
EPI_ISL_2444159	2021-04-26	RHD XV	Gamma
EPI_ISL_2444167	2021-04-26	RHD XV	Gamma
EPI_ISL_2444153	2021-04-26	RHD XV	Gamma
EPI_ISL_2444169	2021-04-26	RHD XV	Gamma
EPI_ISL_2444162	2021-04-26	RHD XV	Gamma
EPI_ISL_2444166	2021-04-26	RHD XV	Gamma
EPI_ISL_2444161	2021-04-26	RHD XV	Gamma
EPI_ISL_2444171	2021-04-26	RHD XV	Gamma
EPI_ISL_2444172	2021-04-26	RHD XV	Gamma
EPI_ISL_2444163	2021-04-26	RHD XV	Gamma
EPI_ISL_2444154	2021-04-26	RHD XV	Gamma
EPI_ISL_2444165	2021-04-26	RHD XV	Gamma
EPI_ISL_2444164	2021-04-26	RHD XV	Gamma
EPI_ISL_2444170	2021-04-26	RHD XV	Gamma
EPI_ISL_2444157	2021-04-26	RHD XV	Gamma
EPI_ISL_3048959	2021-04-26	RHD XV	Gamma
EPI_ISL_3048960	2021-04-26	RHD XV	Gamma
EPI_ISL_3048808	2021-04-26	South	Gamma
EPI_ISL_2017434	2021-04-27	Midwest	Gamma
EPI_ISL_2444176	2021-04-27	RHD XV	Gamma
EPI_ISL_2444177	2021-04-27	RHD XV	Gamma
EPI_ISL_2444185	2021-04-27	RHD XV	Gamma
EPI_ISL_2444178	2021-04-27	RHD XV	Gamma
EPI_ISL_2444179	2021-04-27	RHD XV	Gamma
EPI_ISL_2444182	2021-04-27	RHD XV	Gamma
EPI_ISL_2444180	2021-04-27	RHD XV	Gamma
EPI_ISL_2444188	2021-04-27	RHD XV	Gamma
EPI_ISL_2455396	2021-04-27	RHD XV	Gamma
EPI_ISL_2444175	2021-04-27	RHD XV	Gamma
EPI_ISL_2444181	2021-04-27	RHD XV	Gamma
EPI_ISL_2444183	2021-04-27	RHD XV	Gamma
EPI_ISL_2444189	2021-04-27	RHD XV	Gamma
EPI_ISL_2444187	2021-04-27	RHD XV	Gamma
EPI_ISL_2444174	2021-04-27	RHD XV	Gamma
EPI_ISL_2008906	2021-04-27	RHD XV	Gamma
EPI_ISL_2008907	2021-04-27	RHD XV	Gamma
EPI_ISL_2008908	2021-04-27	RHD XV	Gamma
EPI_ISL_2008909	2021-04-27	RHD XV	Gamma
EPI_ISL_2008910	2021-04-27	RHD XV	Gamma
EPI_ISL_2008911	2021-04-27	RHD XV	Gamma
EPI_ISL_2008912	2021-04-27	RHD XV	Gamma
EPI_ISL_2008913	2021-04-27	RHD XV	Gamma
EPI_ISL_2008914	2021-04-27	RHD XV	Gamma
EPI_ISL_2008915	2021-04-27	RHD XV	Gamma
EPI_ISL_2008916	2021-04-27	RHD XV	Gamma
EPI_ISL_2008917	2021-04-27	RHD XV	Gamma
EPI_ISL_2008918	2021-04-27	RHD XV	Gamma
EPI_ISL_2008919	2021-04-27	RHD XV	Gamma
EPI_ISL_2008920	2021-04-27	RHD XV	Gamma
EPI_ISL_2008921	2021-04-27	RHD XV	Gamma
EPI_ISL_2008922	2021-04-27	RHD XV	Gamma
EPI_ISL_2008923	2021-04-27	RHD XV	Gamma
EPI_ISL_2008924	2021-04-27	RHD XV	Gamma
EPI_ISL_2008925	2021-04-27	RHD XV	Gamma
EPI_ISL_2008926	2021-04-27	RHD XV	Gamma
EPI_ISL_2008927	2021-04-27	RHD XV	Gamma
EPI_ISL_2008928	2021-04-27	RHD XV	Gamma
EPI_ISL_2107292	2021-04-27	RHD XV	Gamma
EPI_ISL_2008929	2021-04-27	RHD XV	Gamma
EPI_ISL_2107293	2021-04-27	RHD XV	Gamma
EPI_ISL_2107294	2021-04-27	RHD XV	Gamma
EPI_ISL_2008930	2021-04-27	RHD XV	Gamma

EPI_ISL_2008931	2021-04-27	RHD XV	Gamma
EPI_ISL_2107301	2021-04-27	RHD XV	Gamma
EPI_ISL_2107296	2021-04-27	RHD XV	Gamma
EPI_ISL_2008932	2021-04-27	RHD XV	Gamma
EPI_ISL_2107298	2021-04-27	RHD XV	Gamma
EPI_ISL_2107295	2021-04-27	RHD XV	Gamma
EPI_ISL_2008933	2021-04-27	RHD XV	Gamma
EPI_ISL_2008934	2021-04-27	RHD XV	Gamma
EPI_ISL_2107297	2021-04-27	RHD XV	Gamma
EPI_ISL_2008935	2021-04-27	RHD XV	Gamma
EPI_ISL_2444191	2021-04-28	RHD XV	Alpha
EPI_ISL_2777881	2021-04-28	North	Gamma
EPI_ISL_2444190	2021-04-28	RHD XV	Gamma
EPI_ISL_2196262	2021-04-28	Southeast	Gamma
EPI_ISL_6908848	2021-04-29	Northeast	Gamma
EPI_ISL_12425121	2021-04-29	South	Gamma
EPI_ISL_2385709	2021-04-29	Southeast	Gamma
EPI_ISL_2497439	2021-04-30	Midwest	Gamma
EPI_ISL_4080767	2021-04-30	North	Gamma
EPI_ISL_2466232	2021-04-30	Northeast	Gamma
EPI_ISL_3447589	2021-04-30	Northeast	Gamma
EPI_ISL_2443606	2021-04-30	Southeast	Gamma
EPI_ISL_2557319	2021-05-01	Northeast	Gamma
EPI_ISL_2983290	2021-05-01	Northeast	Gamma
EPI_ISL_2777703	2021-05-02	North	Gamma
EPI_ISL_2536323	2021-05-02	Northeast	Gamma
EPI_ISL_2444207	2021-05-02	RHD XV	Gamma
EPI_ISL_2444200	2021-05-02	RHD XV	Gamma
EPI_ISL_2444208	2021-05-02	RHD XV	Gamma
EPI_ISL_2444204	2021-05-02	RHD XV	Gamma
EPI_ISL_2444205	2021-05-02	RHD XV	Gamma
EPI_ISL_2444198	2021-05-02	RHD XV	Gamma
EPI_ISL_2444194	2021-05-02	RHD XV	Gamma
EPI_ISL_2444209	2021-05-02	RHD XV	Gamma
EPI_ISL_2444196	2021-05-02	RHD XV	Gamma
EPI_ISL_2444197	2021-05-02	RHD XV	Gamma
EPI_ISL_2455397	2021-05-02	RHD XV	Gamma
EPI_ISL_2444201	2021-05-02	RHD XV	Gamma
EPI_ISL_2444193	2021-05-02	RHD XV	Gamma
EPI_ISL_2444206	2021-05-02	RHD XV	Gamma
EPI_ISL_2444217	2021-05-03	RHD XV	Alpha
EPI_ISL_2187992	2021-05-03	Midwest	Gamma
EPI_ISL_13960027	2021-05-03	Midwest	Gamma
EPI_ISL_2777612	2021-05-03	North	Gamma
EPI_ISL_5193578	2021-05-03	North	Gamma
EPI_ISL_2801357	2021-05-03	Northeast	Gamma
EPI_ISL_2821274	2021-05-03	Northeast	Gamma
EPI_ISL_2385528	2021-05-03	Northeast	Gamma
EPI_ISL_2444215	2021-05-03	RHD XV	Gamma
EPI_ISL_2444214	2021-05-03	RHD XV	Gamma
EPI_ISL_2444219	2021-05-03	RHD XV	Gamma
EPI_ISL_2444218	2021-05-03	RHD XV	Gamma
EPI_ISL_2444222	2021-05-03	RHD XV	Gamma
EPI_ISL_2444213	2021-05-03	RHD XV	Gamma
EPI_ISL_2444220	2021-05-03	RHD XV	Gamma
EPI_ISL_2444221	2021-05-03	RHD XV	Gamma
EPI_ISL_2444216	2021-05-03	RHD XV	Gamma
EPI_ISL_2444211	2021-05-03	RHD XV	Gamma
EPI_ISL_3048961	2021-05-03	RHD XV	Gamma
EPI_ISL_3761763	2021-05-03	RHD XV	Gamma
EPI_ISL_3048962	2021-05-03	RHD XV	Gamma
EPI_ISL_2443673	2021-05-03	South	Gamma
EPI_ISL_3061899	2021-05-03	South	Gamma

EPI_ISL_2645424	2021-05-04	North	Gamma
EPI_ISL_2557320	2021-05-04	Northeast	Gamma
EPI_ISL_2983295	2021-05-04	Northeast	Gamma
EPI_ISL_2983296	2021-05-04	Northeast	Gamma
EPI_ISL_3048963	2021-05-04	RHD XV	Gamma
EPI_ISL_6172741	2021-05-04	Southeast	Gamma
EPI_ISL_2188023	2021-05-05	Midwest	Gamma
EPI_ISL_5926864	2021-05-05	North	Gamma
EPI_ISL_5193575	2021-05-05	North	Gamma
EPI_ISL_2557322	2021-05-05	Northeast	Gamma
EPI_ISL_3048964	2021-05-05	RHD XV	Gamma
EPI_ISL_3048965	2021-05-05	RHD XV	Gamma
EPI_ISL_13459805	2021-05-05	South	Gamma
EPI_ISL_5193573	2021-05-06	North	Gamma
EPI_ISL_3048966	2021-05-06	RHD XV	Gamma
EPI_ISL_3761752	2021-05-06	RHD XV	Gamma
EPI_ISL_3048967	2021-05-06	RHD XV	Gamma
EPI_ISL_3761636	2021-05-06	RHD XV	Gamma
EPI_ISL_2348607	2021-05-07	Midwest	Gamma
EPI_ISL_5926872	2021-05-07	North	Gamma
EPI_ISL_2777919	2021-05-08	North	Gamma
EPI_ISL_2983297	2021-05-08	Northeast	Gamma
EPI_ISL_4487962	2021-05-08	Northeast	Gamma
EPI_ISL_2863625	2021-05-08	Northeast	Gamma
EPI_ISL_3545812	2021-05-09	Midwest	Gamma
EPI_ISL_2777923	2021-05-09	North	Gamma
EPI_ISL_2983299	2021-05-09	Northeast	Gamma
EPI_ISL_12425078	2021-05-09	South	Gamma
EPI_ISL_3316226	2021-05-10	Midwest	Gamma
EPI_ISL_5193568	2021-05-10	North	Gamma
EPI_ISL_2919207	2021-05-10	North	Gamma
EPI_ISL_2445529	2021-05-10	RHD XV	Gamma
EPI_ISL_2445530	2021-05-10	RHD XV	Gamma
EPI_ISL_3048968	2021-05-10	RHD XV	Gamma
EPI_ISL_3048969	2021-05-10	RHD XV	Gamma
EPI_ISL_3048970	2021-05-10	RHD XV	Gamma
EPI_ISL_3048971	2021-05-10	RHD XV	Gamma
EPI_ISL_3761613	2021-05-10	RHD XV	Gamma
EPI_ISL_2443634	2021-05-10	Southeast	Gamma
EPI_ISL_3545811	2021-05-11	Midwest	Gamma
EPI_ISL_3134661	2021-05-11	Northeast	Gamma
EPI_ISL_4487931	2021-05-11	Northeast	Gamma
EPI_ISL_2445531	2021-05-11	RHD XV	Gamma
EPI_ISL_3761576	2021-05-11	RHD XV	Gamma
EPI_ISL_2445513	2021-05-12	RHD XV	Alpha
EPI_ISL_6908891	2021-05-12	Northeast	Gamma
EPI_ISL_2445526	2021-05-12	RHD XV	Gamma
EPI_ISL_2445516	2021-05-12	RHD XV	Gamma
EPI_ISL_2445508	2021-05-12	RHD XV	Gamma
EPI_ISL_2445525	2021-05-12	RHD XV	Gamma
EPI_ISL_2445522	2021-05-12	RHD XV	Gamma
EPI_ISL_2473788	2021-05-12	RHD XV	Gamma
EPI_ISL_2445528	2021-05-12	RHD XV	Gamma
EPI_ISL_2445524	2021-05-12	RHD XV	Gamma
EPI_ISL_2473693	2021-05-12	RHD XV	Gamma
EPI_ISL_2445533	2021-05-12	RHD XV	Gamma
EPI_ISL_2445523	2021-05-12	RHD XV	Gamma
EPI_ISL_2445520	2021-05-12	RHD XV	Gamma
EPI_ISL_2445527	2021-05-12	RHD XV	Gamma
EPI_ISL_2445521	2021-05-12	RHD XV	Gamma
EPI_ISL_2445512	2021-05-12	RHD XV	Gamma
EPI_ISL_2473787	2021-05-12	RHD XV	Gamma
EPI_ISL_2445539	2021-05-12	RHD XV	Gamma

EPI_ISL_2445519	2021-05-12	RHD XV	Gamma
EPI_ISL_2445515	2021-05-12	RHD XV	Gamma
EPI_ISL_2445532	2021-05-12	RHD XV	Gamma
EPI_ISL_2473789	2021-05-12	RHD XV	Gamma
EPI_ISL_2445514	2021-05-12	RHD XV	Gamma
EPI_ISL_2445518	2021-05-12	RHD XV	Gamma
EPI_ISL_2445534	2021-05-12	RHD XV	Gamma
EPI_ISL_2473786	2021-05-12	RHD XV	Gamma
EPI_ISL_2473696	2021-05-12	RHD XV	Gamma
EPI_ISL_3761616	2021-05-12	RHD XV	Gamma
EPI_ISL_6970988	2021-05-12	Southeast	Gamma
EPI_ISL_2348615	2021-05-13	Midwest	Gamma
EPI_ISL_2645427	2021-05-13	North	Gamma
EPI_ISL_2645390	2021-05-13	Northeast	Gamma
EPI_ISL_2473790	2021-05-13	RHD XV	Gamma
EPI_ISL_2473792	2021-05-13	RHD XV	Gamma
EPI_ISL_2473791	2021-05-13	RHD XV	Gamma
EPI_ISL_2445543	2021-05-13	RHD XV	Gamma
EPI_ISL_2445538	2021-05-13	RHD XV	Gamma
EPI_ISL_2445542	2021-05-13	RHD XV	Gamma
EPI_ISL_2445536	2021-05-13	RHD XV	Gamma
EPI_ISL_2445535	2021-05-13	RHD XV	Gamma
EPI_ISL_2445544	2021-05-13	RHD XV	Gamma
EPI_ISL_2445537	2021-05-13	RHD XV	Gamma
EPI_ISL_3048972	2021-05-13	RHD XV	Gamma
EPI_ISL_2777650	2021-05-14	North	Gamma
EPI_ISL_2919211	2021-05-14	North	Gamma
EPI_ISL_8630348	2021-05-14	Southeast	Gamma
EPI_ISL_2691210	2021-05-15	Midwest	Gamma
EPI_ISL_3761646	2021-05-15	RHD XV	Gamma
EPI_ISL_4237345	2021-05-15	Southeast	Gamma
EPI_ISL_2645414	2021-05-16	Northeast	Delta
EPI_ISL_2645418	2021-05-16	Northeast	Delta
EPI_ISL_5915287	2021-05-16	Northeast	Gamma
EPI_ISL_2466451	2021-05-17	Midwest	Gamma
EPI_ISL_2777441	2021-05-17	North	Gamma
EPI_ISL_3048973	2021-05-17	RHD XV	Gamma
EPI_ISL_3048974	2021-05-17	RHD XV	Gamma
EPI_ISL_3048975	2021-05-17	RHD XV	Gamma
EPI_ISL_8630344	2021-05-17	Southeast	Gamma
EPI_ISL_3048976	2021-05-18	RHD XV	Gamma
EPI_ISL_4237342	2021-05-18	Southeast	Gamma
EPI_ISL_5926880	2021-05-19	North	Gamma
EPI_ISL_8127599	2021-05-19	Northeast	Gamma
EPI_ISL_2473718	2021-05-19	RHD XV	Gamma
EPI_ISL_2473719	2021-05-19	RHD XV	Gamma
EPI_ISL_2445200	2021-05-19	RHD XV	Gamma
EPI_ISL_2473715	2021-05-19	RHD XV	Gamma
EPI_ISL_2445213	2021-05-19	RHD XV	Gamma
EPI_ISL_2445199	2021-05-19	RHD XV	Gamma
EPI_ISL_2445201	2021-05-19	RHD XV	Gamma
EPI_ISL_2445214	2021-05-19	RHD XV	Gamma
EPI_ISL_2473716	2021-05-19	RHD XV	Gamma
EPI_ISL_2445215	2021-05-19	RHD XV	Gamma
EPI_ISL_2445203	2021-05-19	RHD XV	Gamma
EPI_ISL_2445217	2021-05-19	RHD XV	Gamma
EPI_ISL_2445207	2021-05-19	RHD XV	Gamma
EPI_ISL_2473818	2021-05-19	RHD XV	Gamma
EPI_ISL_2473817	2021-05-19	RHD XV	Gamma
EPI_ISL_2445218	2021-05-19	RHD XV	Gamma
EPI_ISL_2473723	2021-05-19	RHD XV	Gamma
EPI_ISL_2445205	2021-05-19	RHD XV	Gamma
EPI_ISL_2445202	2021-05-19	RHD XV	Gamma

EPI_ISL_2473721	2021-05-19	RHD XV	Gamma
EPI_ISL_2445208	2021-05-19	RHD XV	Gamma
EPI_ISL_2445198	2021-05-19	RHD XV	Gamma
EPI_ISL_2445204	2021-05-19	RHD XV	Gamma
EPI_ISL_2445206	2021-05-19	RHD XV	Gamma
EPI_ISL_2473714	2021-05-19	RHD XV	Gamma
EPI_ISL_2473717	2021-05-19	RHD XV	Gamma
EPI_ISL_2473722	2021-05-19	RHD XV	Gamma
EPI_ISL_2500975	2021-05-19	RHD XV	Gamma
EPI_ISL_2493161	2021-05-19	RHD XV	Gamma
EPI_ISL_2493177	2021-05-19	RHD XV	Gamma
EPI_ISL_2494337	2021-05-19	RHD XV	Gamma
EPI_ISL_2493426	2021-05-19	RHD XV	Gamma
EPI_ISL_2493180	2021-05-19	RHD XV	Gamma
EPI_ISL_2493413	2021-05-19	RHD XV	Gamma
EPI_ISL_2493405	2021-05-19	RHD XV	Gamma
EPI_ISL_2493414	2021-05-19	RHD XV	Gamma
EPI_ISL_2493416	2021-05-19	RHD XV	Gamma
EPI_ISL_2493422	2021-05-19	RHD XV	Gamma
EPI_ISL_2493408	2021-05-19	RHD XV	Gamma
EPI_ISL_2493412	2021-05-19	RHD XV	Gamma
EPI_ISL_2493423	2021-05-19	RHD XV	Gamma
EPI_ISL_2493402	2021-05-19	RHD XV	Gamma
EPI_ISL_2493429	2021-05-19	RHD XV	Gamma
EPI_ISL_2493424	2021-05-19	RHD XV	Gamma
EPI_ISL_2493420	2021-05-19	RHD XV	Gamma
EPI_ISL_2493400	2021-05-19	RHD XV	Gamma
EPI_ISL_2493406	2021-05-19	RHD XV	Gamma
EPI_ISL_2493404	2021-05-19	RHD XV	Gamma
EPI_ISL_2493418	2021-05-19	RHD XV	Gamma
EPI_ISL_2493425	2021-05-19	RHD XV	Gamma
EPI_ISL_2493409	2021-05-19	RHD XV	Gamma
EPI_ISL_2493415	2021-05-19	RHD XV	Gamma
EPI_ISL_2493410	2021-05-19	RHD XV	Gamma
EPI_ISL_2493401	2021-05-19	RHD XV	Gamma
EPI_ISL_2493421	2021-05-19	RHD XV	Gamma
EPI_ISL_2493430	2021-05-19	RHD XV	Gamma
EPI_ISL_2493431	2021-05-19	RHD XV	Gamma
EPI_ISL_2493417	2021-05-19	RHD XV	Gamma
EPI_ISL_2493411	2021-05-19	RHD XV	Gamma
EPI_ISL_2493407	2021-05-19	RHD XV	Gamma
EPI_ISL_2493419	2021-05-19	RHD XV	Gamma
EPI_ISL_2493663	2021-05-19	RHD XV	Gamma
EPI_ISL_2493655	2021-05-19	RHD XV	Gamma
EPI_ISL_2493661	2021-05-19	RHD XV	Gamma
EPI_ISL_2493662	2021-05-19	RHD XV	Gamma
EPI_ISL_2493656	2021-05-19	RHD XV	Gamma
EPI_ISL_5645966	2021-05-19	RHD XV	Gamma
EPI_ISL_5645969	2021-05-19	RHD XV	Gamma
EPI_ISL_5645974	2021-05-19	RHD XV	Gamma
EPI_ISL_5645975	2021-05-19	RHD XV	Gamma
EPI_ISL_5645976	2021-05-19	RHD XV	Gamma
EPI_ISL_5645977	2021-05-19	RHD XV	Gamma
EPI_ISL_5645979	2021-05-19	RHD XV	Gamma
EPI_ISL_5645981	2021-05-19	RHD XV	Gamma
EPI_ISL_5645982	2021-05-19	RHD XV	Gamma
EPI_ISL_5645984	2021-05-19	RHD XV	Gamma
EPI_ISL_5645986	2021-05-19	RHD XV	Gamma
EPI_ISL_5645987	2021-05-19	RHD XV	Gamma
EPI_ISL_5645988	2021-05-19	RHD XV	Gamma
EPI_ISL_5645989	2021-05-19	RHD XV	Gamma
EPI_ISL_5645993	2021-05-19	RHD XV	Gamma
EPI_ISL_5645994	2021-05-19	RHD XV	Gamma

EPI_ISL_5645995	2021-05-19	RHD XV	Gamma
EPI_ISL_5645997	2021-05-19	RHD XV	Gamma
EPI_ISL_5645998	2021-05-19	RHD XV	Gamma
EPI_ISL_5646000	2021-05-19	RHD XV	Gamma
EPI_ISL_5646003	2021-05-19	RHD XV	Gamma
EPI_ISL_5646004	2021-05-19	RHD XV	Gamma
EPI_ISL_5646005	2021-05-19	RHD XV	Gamma
EPI_ISL_5646006	2021-05-19	RHD XV	Gamma
EPI_ISL_5646007	2021-05-19	RHD XV	Gamma
EPI_ISL_5646010	2021-05-19	RHD XV	Gamma
EPI_ISL_5646011	2021-05-19	RHD XV	Gamma
EPI_ISL_5646012	2021-05-19	RHD XV	Gamma
EPI_ISL_5646013	2021-05-19	RHD XV	Gamma
EPI_ISL_5646014	2021-05-19	RHD XV	Gamma
EPI_ISL_5646015	2021-05-19	RHD XV	Gamma
EPI_ISL_5646018	2021-05-19	RHD XV	Gamma
EPI_ISL_5646023	2021-05-19	RHD XV	Gamma
EPI_ISL_5646024	2021-05-19	RHD XV	Gamma
EPI_ISL_5646029	2021-05-19	RHD XV	Gamma
EPI_ISL_5646031	2021-05-19	RHD XV	Gamma
EPI_ISL_5646032	2021-05-19	RHD XV	Gamma
EPI_ISL_5646033	2021-05-19	RHD XV	Gamma
EPI_ISL_3048977	2021-05-19	RHD XV	Gamma
EPI_ISL_3761645	2021-05-19	RHD XV	Gamma
EPI_ISL_2378751	2021-05-19	RHD XV	Gamma
EPI_ISL_3190350	2021-05-20	Midwest	Gamma
EPI_ISL_5926877	2021-05-20	North	Gamma
EPI_ISL_5193556	2021-05-20	North	Gamma
EPI_ISL_2473720	2021-05-20	RHD XV	Gamma
EPI_ISL_2445210	2021-05-20	RHD XV	Gamma
EPI_ISL_2445209	2021-05-20	RHD XV	Gamma
EPI_ISL_2445211	2021-05-20	RHD XV	Gamma
EPI_ISL_2445212	2021-05-20	RHD XV	Gamma
EPI_ISL_2445216	2021-05-20	RHD XV	Gamma
EPI_ISL_2493664	2021-05-20	RHD XV	Gamma
EPI_ISL_2493659	2021-05-20	RHD XV	Gamma
EPI_ISL_2494297	2021-05-20	RHD XV	Gamma
EPI_ISL_2493658	2021-05-20	RHD XV	Gamma
EPI_ISL_2493666	2021-05-20	RHD XV	Gamma
EPI_ISL_2493665	2021-05-20	RHD XV	Gamma
EPI_ISL_5646016	2021-05-20	RHD XV	Gamma
EPI_ISL_5646025	2021-05-20	RHD XV	Gamma
EPI_ISL_5646028	2021-05-20	RHD XV	Gamma
EPI_ISL_3048978	2021-05-20	RHD XV	Gamma
EPI_ISL_3761570	2021-05-20	RHD XV	Gamma
EPI_ISL_3045462	2021-05-20	Southeast	Gamma
EPI_ISL_2378753	2021-05-20	RHD XV	Gamma
EPI_ISL_2378754	2021-05-20	RHD XV	Gamma
EPI_ISL_2378744	2021-05-20	RHD XV	Gamma
EPI_ISL_5926896	2021-05-21	North	Gamma
EPI_ISL_2777961	2021-05-21	North	Gamma
EPI_ISL_2645402	2021-05-22	Northeast	Gamma
EPI_ISL_3102522	2021-05-22	Northeast	Gamma
EPI_ISL_3447590	2021-05-22	Northeast	Gamma
EPI_ISL_8005364	2021-05-22	South	Gamma
EPI_ISL_14670064	2021-05-24	Midwest	Delta
EPI_ISL_2645404	2021-05-24	Northeast	Gamma
EPI_ISL_8127633	2021-05-24	Northeast	Gamma
EPI_ISL_3048979	2021-05-24	RHD XV	Gamma
EPI_ISL_2691615	2021-05-24	Southeast	Gamma
EPI_ISL_2497457	2021-05-25	Midwest	Gamma
EPI_ISL_5926883	2021-05-25	North	Gamma
EPI_ISL_2691575	2021-05-25	Northeast	Gamma

EPI_ISL_3048980	2021-05-25	RHD XV	Gamma
EPI_ISL_3761637	2021-05-25	RHD XV	Gamma
EPI_ISL_8005365	2021-05-25	South	Gamma
EPI_ISL_12140068	2021-05-26	Northeast	Gamma
EPI_ISL_11406416	2021-05-26	Northeast	Gamma
EPI_ISL_5649576	2021-05-26	RHD XV	Gamma
EPI_ISL_5649585	2021-05-26	RHD XV	Gamma
EPI_ISL_5650052	2021-05-26	RHD XV	Gamma
EPI_ISL_5649594	2021-05-26	RHD XV	Gamma
EPI_ISL_5649653	2021-05-26	RHD XV	Gamma
EPI_ISL_5649579	2021-05-26	RHD XV	Gamma
EPI_ISL_5649624	2021-05-26	RHD XV	Gamma
EPI_ISL_5649586	2021-05-26	RHD XV	Gamma
EPI_ISL_5650067	2021-05-26	RHD XV	Gamma
EPI_ISL_5649584	2021-05-26	RHD XV	Gamma
EPI_ISL_5649654	2021-05-26	RHD XV	Gamma
EPI_ISL_5649625	2021-05-26	RHD XV	Gamma
EPI_ISL_5650074	2021-05-26	RHD XV	Gamma
EPI_ISL_5650076	2021-05-26	RHD XV	Gamma
EPI_ISL_5650081	2021-05-26	RHD XV	Gamma
EPI_ISL_5649678	2021-05-26	RHD XV	Gamma
EPI_ISL_5649609	2021-05-26	RHD XV	Gamma
EPI_ISL_5649626	2021-05-26	RHD XV	Gamma
EPI_ISL_5649677	2021-05-26	RHD XV	Gamma
EPI_ISL_5649666	2021-05-26	RHD XV	Gamma
EPI_ISL_5649685	2021-05-26	RHD XV	Gamma
EPI_ISL_5649686	2021-05-26	RHD XV	Gamma
EPI_ISL_5649687	2021-05-26	RHD XV	Gamma
EPI_ISL_5650084	2021-05-26	RHD XV	Gamma
EPI_ISL_5650085	2021-05-26	RHD XV	Gamma
EPI_ISL_5649655	2021-05-26	RHD XV	Gamma
EPI_ISL_5649600	2021-05-26	RHD XV	Gamma
EPI_ISL_5650099	2021-05-26	RHD XV	Gamma
EPI_ISL_5650107	2021-05-26	RHD XV	Gamma
EPI_ISL_5650112	2021-05-26	RHD XV	Gamma
EPI_ISL_5649656	2021-05-26	RHD XV	Gamma
EPI_ISL_5649601	2021-05-26	RHD XV	Gamma
EPI_ISL_5650120	2021-05-26	RHD XV	Gamma
EPI_ISL_5650134	2021-05-26	RHD XV	Gamma
EPI_ISL_5650141	2021-05-26	RHD XV	Gamma
EPI_ISL_5650150	2021-05-26	RHD XV	Gamma
EPI_ISL_5650165	2021-05-26	RHD XV	Gamma
EPI_ISL_5650166	2021-05-26	RHD XV	Gamma
EPI_ISL_5650167	2021-05-26	RHD XV	Gamma
EPI_ISL_5650168	2021-05-26	RHD XV	Gamma
EPI_ISL_5650169	2021-05-26	RHD XV	Gamma
EPI_ISL_5650170	2021-05-26	RHD XV	Gamma
EPI_ISL_5650174	2021-05-26	RHD XV	Gamma
EPI_ISL_5650175	2021-05-26	RHD XV	Gamma
EPI_ISL_5650176	2021-05-26	RHD XV	Gamma
EPI_ISL_5649658	2021-05-26	RHD XV	Gamma
EPI_ISL_5650178	2021-05-26	RHD XV	Gamma
EPI_ISL_5650179	2021-05-26	RHD XV	Gamma
EPI_ISL_5650180	2021-05-26	RHD XV	Gamma
EPI_ISL_5650410	2021-05-26	RHD XV	Gamma
EPI_ISL_5650182	2021-05-26	RHD XV	Gamma
EPI_ISL_5650183	2021-05-26	RHD XV	Gamma
EPI_ISL_5650184	2021-05-26	RHD XV	Gamma
EPI_ISL_5650185	2021-05-26	RHD XV	Gamma
EPI_ISL_5649659	2021-05-26	RHD XV	Gamma
EPI_ISL_5649610	2021-05-26	RHD XV	Gamma
EPI_ISL_5650188	2021-05-26	RHD XV	Gamma
EPI_ISL_5650189	2021-05-26	RHD XV	Gamma

EPI_ISL_5649660	2021-05-26	RHD XV	Gamma
EPI_ISL_5650193	2021-05-26	RHD XV	Gamma
EPI_ISL_5650194	2021-05-26	RHD XV	Gamma
EPI_ISL_5650195	2021-05-26	RHD XV	Gamma
EPI_ISL_5650196	2021-05-26	RHD XV	Gamma
EPI_ISL_5650197	2021-05-26	RHD XV	Gamma
EPI_ISL_5650198	2021-05-26	RHD XV	Gamma
EPI_ISL_5650199	2021-05-26	RHD XV	Gamma
EPI_ISL_5650200	2021-05-26	RHD XV	Gamma
EPI_ISL_5649607	2021-05-26	RHD XV	Gamma
EPI_ISL_2494144	2021-05-26	RHD XV	Gamma
EPI_ISL_3761617	2021-05-26	RHD XV	Gamma
EPI_ISL_3761638	2021-05-26	RHD XV	Gamma
EPI_ISL_3761639	2021-05-26	RHD XV	Gamma
EPI_ISL_3761640	2021-05-26	RHD XV	Gamma
EPI_ISL_3761641	2021-05-26	RHD XV	Gamma
EPI_ISL_3761642	2021-05-26	RHD XV	Gamma
EPI_ISL_13833556	2021-05-26	Southeast	Gamma
EPI_ISL_2777942	2021-05-27	North	Gamma
EPI_ISL_2863626	2021-05-27	Northeast	Gamma
EPI_ISL_5649566	2021-05-27	RHD XV	Gamma
EPI_ISL_5649639	2021-05-27	RHD XV	Gamma
EPI_ISL_5649667	2021-05-27	RHD XV	Gamma
EPI_ISL_5649619	2021-05-27	RHD XV	Gamma
EPI_ISL_5650058	2021-05-27	RHD XV	Gamma
EPI_ISL_5649636	2021-05-27	RHD XV	Gamma
EPI_ISL_5649567	2021-05-27	RHD XV	Gamma
EPI_ISL_5649587	2021-05-27	RHD XV	Gamma
EPI_ISL_5649621	2021-05-27	RHD XV	Gamma
EPI_ISL_5650092	2021-05-27	RHD XV	Gamma
EPI_ISL_5649602	2021-05-27	RHD XV	Gamma
EPI_ISL_5650126	2021-05-27	RHD XV	Gamma
EPI_ISL_5649645	2021-05-27	RHD XV	Gamma
EPI_ISL_5649657	2021-05-27	RHD XV	Gamma
EPI_ISL_5650157	2021-05-27	RHD XV	Gamma
EPI_ISL_5650159	2021-05-27	RHD XV	Gamma
EPI_ISL_5650171	2021-05-27	RHD XV	Gamma
EPI_ISL_5650172	2021-05-27	RHD XV	Gamma
EPI_ISL_5650173	2021-05-27	RHD XV	Gamma
EPI_ISL_5650177	2021-05-27	RHD XV	Gamma
EPI_ISL_5650181	2021-05-27	RHD XV	Gamma
EPI_ISL_5649679	2021-05-27	RHD XV	Gamma
EPI_ISL_5650186	2021-05-27	RHD XV	Gamma
EPI_ISL_5650187	2021-05-27	RHD XV	Gamma
EPI_ISL_5650190	2021-05-27	RHD XV	Gamma
EPI_ISL_5650191	2021-05-27	RHD XV	Gamma
EPI_ISL_5650192	2021-05-27	RHD XV	Gamma
EPI_ISL_3761643	2021-05-27	RHD XV	Gamma
EPI_ISL_3761644	2021-05-27	RHD XV	Gamma
EPI_ISL_2983428	2021-05-27	South	Gamma
EPI_ISL_5926866	2021-05-28	North	Gamma
EPI_ISL_2603532	2021-05-28	South	Gamma
EPI_ISL_3048997	2021-05-30	RHD XV	Gamma
EPI_ISL_8004636	2021-05-30	Southeast	Gamma
EPI_ISL_5650416	2021-05-31	RHD XV	Gamma
EPI_ISL_5650415	2021-05-31	RHD XV	Gamma
EPI_ISL_5649569	2021-05-31	RHD XV	Gamma
EPI_ISL_5649634	2021-05-31	RHD XV	Gamma
EPI_ISL_5649629	2021-05-31	RHD XV	Gamma
EPI_ISL_5649603	2021-05-31	RHD XV	Gamma
EPI_ISL_5649581	2021-05-31	RHD XV	Gamma
EPI_ISL_5650203	2021-05-31	RHD XV	Gamma
EPI_ISL_5649583	2021-05-31	RHD XV	Gamma

EPI_ISL_5649571	2021-05-31	RHD XV	Gamma
EPI_ISL_5650208	2021-05-31	RHD XV	Gamma
EPI_ISL_5649642	2021-05-31	RHD XV	Gamma
EPI_ISL_5649683	2021-05-31	RHD XV	Gamma
EPI_ISL_5650216	2021-05-31	RHD XV	Gamma
EPI_ISL_5650220	2021-05-31	RHD XV	Gamma
EPI_ISL_5650222	2021-05-31	RHD XV	Gamma
EPI_ISL_5650224	2021-05-31	RHD XV	Gamma
EPI_ISL_5650226	2021-05-31	RHD XV	Gamma
EPI_ISL_5650230	2021-05-31	RHD XV	Gamma
EPI_ISL_5650335	2021-05-31	RHD XV	Gamma
EPI_ISL_5650337	2021-05-31	RHD XV	Gamma
EPI_ISL_5649591	2021-05-31	RHD XV	Gamma
EPI_ISL_5650356	2021-05-31	RHD XV	Gamma
EPI_ISL_5650358	2021-05-31	RHD XV	Gamma
EPI_ISL_5650359	2021-05-31	RHD XV	Gamma
EPI_ISL_5650377	2021-05-31	RHD XV	Gamma
EPI_ISL_5645571	2021-05-31	RHD XV	Gamma
EPI_ISL_5645573	2021-05-31	RHD XV	Gamma
EPI_ISL_5645575	2021-05-31	RHD XV	Gamma
EPI_ISL_5645576	2021-05-31	RHD XV	Gamma
EPI_ISL_5646304	2021-05-31	RHD XV	Gamma
EPI_ISL_5646309	2021-05-31	RHD XV	Gamma
EPI_ISL_5646319	2021-05-31	RHD XV	Gamma
EPI_ISL_5646320	2021-05-31	RHD XV	Gamma
EPI_ISL_5646321	2021-05-31	RHD XV	Gamma
EPI_ISL_5646322	2021-05-31	RHD XV	Gamma
EPI_ISL_5646323	2021-05-31	RHD XV	Gamma
EPI_ISL_5646329	2021-05-31	RHD XV	Gamma
EPI_ISL_5646332	2021-05-31	RHD XV	Gamma
EPI_ISL_5646345	2021-05-31	RHD XV	Gamma
EPI_ISL_5646355	2021-05-31	RHD XV	Gamma
EPI_ISL_5646363	2021-05-31	RHD XV	Gamma
EPI_ISL_5646368	2021-05-31	RHD XV	Gamma
EPI_ISL_5646369	2021-05-31	RHD XV	Gamma
EPI_ISL_5646374	2021-05-31	RHD XV	Gamma
EPI_ISL_5646382	2021-05-31	RHD XV	Gamma
EPI_ISL_5646383	2021-05-31	RHD XV	Gamma
EPI_ISL_5647110	2021-05-31	RHD XV	Gamma
EPI_ISL_5647114	2021-05-31	RHD XV	Gamma
EPI_ISL_5647115	2021-05-31	RHD XV	Gamma
EPI_ISL_5647117	2021-05-31	RHD XV	Gamma
EPI_ISL_5647119	2021-05-31	RHD XV	Gamma
EPI_ISL_5647121	2021-05-31	RHD XV	Gamma
EPI_ISL_5647123	2021-05-31	RHD XV	Gamma
EPI_ISL_5647129	2021-05-31	RHD XV	Gamma
EPI_ISL_5647132	2021-05-31	RHD XV	Gamma
EPI_ISL_5647133	2021-05-31	RHD XV	Gamma
EPI_ISL_5647136	2021-05-31	RHD XV	Gamma
EPI_ISL_5647137	2021-05-31	RHD XV	Gamma
EPI_ISL_5647147	2021-05-31	RHD XV	Gamma
EPI_ISL_5647150	2021-05-31	RHD XV	Gamma
EPI_ISL_5647153	2021-05-31	RHD XV	Gamma
EPI_ISL_5647154	2021-05-31	RHD XV	Gamma
EPI_ISL_5647156	2021-05-31	RHD XV	Gamma
EPI_ISL_5647157	2021-05-31	RHD XV	Gamma
EPI_ISL_5647168	2021-05-31	RHD XV	Gamma
EPI_ISL_5647169	2021-05-31	RHD XV	Gamma
EPI_ISL_5647171	2021-05-31	RHD XV	Gamma
EPI_ISL_5647177	2021-05-31	RHD XV	Gamma
EPI_ISL_5647187	2021-05-31	RHD XV	Gamma
EPI_ISL_5647189	2021-05-31	RHD XV	Gamma
EPI_ISL_5647190	2021-05-31	RHD XV	Gamma

EPI_ISL_5647191	2021-05-31	RHD XV	Gamma
EPI_ISL_5647193	2021-05-31	RHD XV	Gamma
EPI_ISL_3048981	2021-05-31	RHD XV	Gamma
EPI_ISL_3048982	2021-05-31	RHD XV	Gamma
EPI_ISL_3048983	2021-05-31	RHD XV	Gamma
EPI_ISL_3048984	2021-05-31	RHD XV	Gamma
EPI_ISL_3048985	2021-05-31	RHD XV	Gamma
EPI_ISL_3048986	2021-05-31	RHD XV	Gamma
EPI_ISL_3048987	2021-05-31	RHD XV	Gamma
EPI_ISL_3048988	2021-05-31	RHD XV	Gamma
EPI_ISL_3048989	2021-05-31	RHD XV	Gamma
EPI_ISL_3048990	2021-05-31	RHD XV	Gamma
EPI_ISL_3048991	2021-05-31	RHD XV	Gamma
EPI_ISL_3048992	2021-05-31	RHD XV	Gamma
EPI_ISL_3048993	2021-05-31	RHD XV	Gamma
EPI_ISL_3048994	2021-05-31	RHD XV	Gamma
EPI_ISL_3048995	2021-05-31	RHD XV	Gamma
EPI_ISL_3048996	2021-05-31	RHD XV	Gamma
EPI_ISL_8005380	2021-05-31	South	Gamma
EPI_ISL_3761760	2021-06	RHD XV	Gamma
EPI_ISL_3254386	2021-06-01	Midwest	Gamma
EPI_ISL_3050312	2021-06-01	North	Gamma
EPI_ISL_3536326	2021-06-01	Northeast	Gamma
EPI_ISL_5650333	2021-06-01	RHD XV	Gamma
EPI_ISL_5650334	2021-06-01	RHD XV	Gamma
EPI_ISL_5650341	2021-06-01	RHD XV	Gamma
EPI_ISL_5650344	2021-06-01	RHD XV	Gamma
EPI_ISL_5649589	2021-06-01	RHD XV	Gamma
EPI_ISL_5650352	2021-06-01	RHD XV	Gamma
EPI_ISL_5650353	2021-06-01	RHD XV	Gamma
EPI_ISL_5650357	2021-06-01	RHD XV	Gamma
EPI_ISL_5649592	2021-06-01	RHD XV	Gamma
EPI_ISL_5650361	2021-06-01	RHD XV	Gamma
EPI_ISL_5650371	2021-06-01	RHD XV	Gamma
EPI_ISL_5650374	2021-06-01	RHD XV	Gamma
EPI_ISL_5650381	2021-06-01	RHD XV	Gamma
EPI_ISL_5650385	2021-06-01	RHD XV	Gamma
EPI_ISL_5650386	2021-06-01	RHD XV	Gamma
EPI_ISL_5645574	2021-06-01	RHD XV	Gamma
EPI_ISL_5645577	2021-06-01	RHD XV	Gamma
EPI_ISL_5645579	2021-06-01	RHD XV	Gamma
EPI_ISL_5647113	2021-06-01	RHD XV	Gamma
EPI_ISL_5647124	2021-06-01	RHD XV	Gamma
EPI_ISL_5647127	2021-06-01	RHD XV	Gamma
EPI_ISL_5647158	2021-06-01	RHD XV	Gamma
EPI_ISL_5647173	2021-06-01	RHD XV	Gamma
EPI_ISL_5647197	2021-06-01	RHD XV	Gamma
EPI_ISL_8005384	2021-06-01	South	Gamma
EPI_ISL_5193544	2021-06-02	North	Gamma
EPI_ISL_4061453	2021-06-02	Northeast	Gamma
EPI_ISL_12425799	2021-06-02	South	Gamma
EPI_ISL_5926868	2021-06-03	North	Gamma
EPI_ISL_5926869	2021-06-03	North	Gamma
EPI_ISL_5926894	2021-06-03	North	Gamma
EPI_ISL_8004651	2021-06-03	Southeast	Gamma
EPI_ISL_3254413	2021-06-04	Midwest	Gamma
EPI_ISL_12059875	2021-06-04	South	Gamma
EPI_ISL_3050332	2021-06-05	North	Gamma
EPI_ISL_2983075	2021-06-05	Northeast	Gamma
EPI_ISL_3536324	2021-06-05	Northeast	Gamma
EPI_ISL_3102397	2021-06-06	Northeast	Gamma
EPI_ISL_5646979	2021-06-06	RHD XV	Gamma
EPI_ISL_5646982	2021-06-06	RHD XV	Gamma

EPI_ISL_5646987	2021-06-06	RHD XV	Gamma
EPI_ISL_5646995	2021-06-06	RHD XV	Gamma
EPI_ISL_5647002	2021-06-06	RHD XV	Gamma
EPI_ISL_5647003	2021-06-06	RHD XV	Gamma
EPI_ISL_5647216	2021-06-06	RHD XV	Gamma
EPI_ISL_5647228	2021-06-06	RHD XV	Gamma
EPI_ISL_5647233	2021-06-06	RHD XV	Gamma
EPI_ISL_5647240	2021-06-06	RHD XV	Gamma
EPI_ISL_5647243	2021-06-06	RHD XV	Gamma
EPI_ISL_5647249	2021-06-06	RHD XV	Gamma
EPI_ISL_5647204	2021-06-07	RHD XV	Alpha
EPI_ISL_5647205	2021-06-07	RHD XV	Alpha
EPI_ISL_4271189	2021-06-07	Southeast	Alpha
EPI_ISL_5193542	2021-06-07	North	Gamma
EPI_ISL_11468167	2021-06-07	Northeast	Gamma
EPI_ISL_5646871	2021-06-07	RHD XV	Gamma
EPI_ISL_5646875	2021-06-07	RHD XV	Gamma
EPI_ISL_5646877	2021-06-07	RHD XV	Gamma
EPI_ISL_5646881	2021-06-07	RHD XV	Gamma
EPI_ISL_5646882	2021-06-07	RHD XV	Gamma
EPI_ISL_5646884	2021-06-07	RHD XV	Gamma
EPI_ISL_5646885	2021-06-07	RHD XV	Gamma
EPI_ISL_5646888	2021-06-07	RHD XV	Gamma
EPI_ISL_5646889	2021-06-07	RHD XV	Gamma
EPI_ISL_5646897	2021-06-07	RHD XV	Gamma
EPI_ISL_5646901	2021-06-07	RHD XV	Gamma
EPI_ISL_5646902	2021-06-07	RHD XV	Gamma
EPI_ISL_5646910	2021-06-07	RHD XV	Gamma
EPI_ISL_5646916	2021-06-07	RHD XV	Gamma
EPI_ISL_5646917	2021-06-07	RHD XV	Gamma
EPI_ISL_5646921	2021-06-07	RHD XV	Gamma
EPI_ISL_5646931	2021-06-07	RHD XV	Gamma
EPI_ISL_5646932	2021-06-07	RHD XV	Gamma
EPI_ISL_5646933	2021-06-07	RHD XV	Gamma
EPI_ISL_5646943	2021-06-07	RHD XV	Gamma
EPI_ISL_5646962	2021-06-07	RHD XV	Gamma
EPI_ISL_5646963	2021-06-07	RHD XV	Gamma
EPI_ISL_5646967	2021-06-07	RHD XV	Gamma
EPI_ISL_5646968	2021-06-07	RHD XV	Gamma
EPI_ISL_5646976	2021-06-07	RHD XV	Gamma
EPI_ISL_5646977	2021-06-07	RHD XV	Gamma
EPI_ISL_5646983	2021-06-07	RHD XV	Gamma
EPI_ISL_5646986	2021-06-07	RHD XV	Gamma
EPI_ISL_5646993	2021-06-07	RHD XV	Gamma
EPI_ISL_5647209	2021-06-07	RHD XV	Gamma
EPI_ISL_5647212	2021-06-07	RHD XV	Gamma
EPI_ISL_5647213	2021-06-07	RHD XV	Gamma
EPI_ISL_5647219	2021-06-07	RHD XV	Gamma
EPI_ISL_5647220	2021-06-07	RHD XV	Gamma
EPI_ISL_5647227	2021-06-07	RHD XV	Gamma
EPI_ISL_5647229	2021-06-07	RHD XV	Gamma
EPI_ISL_5647236	2021-06-07	RHD XV	Gamma
EPI_ISL_5647241	2021-06-07	RHD XV	Gamma
EPI_ISL_5647244	2021-06-07	RHD XV	Gamma
EPI_ISL_5647245	2021-06-07	RHD XV	Gamma
EPI_ISL_5647247	2021-06-07	RHD XV	Gamma
EPI_ISL_5647251	2021-06-07	RHD XV	Gamma
EPI_ISL_5647254	2021-06-07	RHD XV	Gamma
EPI_ISL_5647259	2021-06-07	RHD XV	Gamma
EPI_ISL_5647260	2021-06-07	RHD XV	Gamma
EPI_ISL_5647261	2021-06-07	RHD XV	Gamma
EPI_ISL_5647263	2021-06-07	RHD XV	Gamma
EPI_ISL_5647266	2021-06-07	RHD XV	Gamma

EPI_ISL_5647268	2021-06-07	RHD XV	Gamma
EPI_ISL_5647272	2021-06-07	RHD XV	Gamma
EPI_ISL_5647292	2021-06-07	RHD XV	Gamma
EPI_ISL_5647294	2021-06-07	RHD XV	Gamma
EPI_ISL_2617624	2021-06-08	Midwest	Gamma
EPI_ISL_6840926	2021-06-08	North	Gamma
EPI_ISL_11681283	2021-06-08	Northeast	Gamma
EPI_ISL_5646876	2021-06-08	RHD XV	Gamma
EPI_ISL_5646880	2021-06-08	RHD XV	Gamma
EPI_ISL_5646894	2021-06-08	RHD XV	Gamma
EPI_ISL_5646896	2021-06-08	RHD XV	Gamma
EPI_ISL_5646907	2021-06-08	RHD XV	Gamma
EPI_ISL_5646913	2021-06-08	RHD XV	Gamma
EPI_ISL_5646914	2021-06-08	RHD XV	Gamma
EPI_ISL_5646915	2021-06-08	RHD XV	Gamma
EPI_ISL_5646930	2021-06-08	RHD XV	Gamma
EPI_ISL_5647210	2021-06-08	RHD XV	Gamma
EPI_ISL_5647214	2021-06-08	RHD XV	Gamma
EPI_ISL_5647257	2021-06-08	RHD XV	Gamma
EPI_ISL_5647262	2021-06-08	RHD XV	Gamma
EPI_ISL_5647267	2021-06-08	RHD XV	Gamma
EPI_ISL_5647271	2021-06-08	RHD XV	Gamma
EPI_ISL_5647275	2021-06-08	RHD XV	Gamma
EPI_ISL_2982725	2021-06-08	Southeast	Gamma
EPI_ISL_4271185	2021-06-08	Southeast	Gamma
EPI_ISL_3254437	2021-06-09	Midwest	Gamma
EPI_ISL_3050353	2021-06-09	North	Gamma
EPI_ISL_8127686	2021-06-09	Northeast	Gamma
EPI_ISL_3536205	2021-06-09	Northeast	Gamma
EPI_ISL_4206971	2021-06-09	Northeast	Gamma
EPI_ISL_3048998	2021-06-09	RHD XV	Gamma
EPI_ISL_3048999	2021-06-09	RHD XV	Gamma
EPI_ISL_3049000	2021-06-09	RHD XV	Gamma
EPI_ISL_2982786	2021-06-09	South	Gamma
EPI_ISL_2919268	2021-06-09	Southeast	Gamma
EPI_ISL_13287026	2021-06-10	North	Delta
EPI_ISL_5193537	2021-06-10	North	Gamma
EPI_ISL_8005401	2021-06-10	South	Gamma
EPI_ISL_2919269	2021-06-10	Southeast	Gamma
EPI_ISL_3045470	2021-06-11	Southeast	Gamma
EPI_ISL_2919275	2021-06-11	Southeast	Gamma
EPI_ISL_3190310	2021-06-12	Northeast	Gamma
EPI_ISL_2863641	2021-06-12	Northeast	Gamma
EPI_ISL_3761761	2021-06-12	RHD XV	Gamma
EPI_ISL_3434898	2021-06-13	North	Gamma
EPI_ISL_5648047	2021-06-13	RHD XV	Gamma
EPI_ISL_5648129	2021-06-13	RHD XV	Gamma
EPI_ISL_2919287	2021-06-13	Southeast	Gamma
EPI_ISL_3434756	2021-06-14	Northeast	Gamma
EPI_ISL_5647663	2021-06-14	RHD XV	Gamma
EPI_ISL_5647689	2021-06-14	RHD XV	Gamma
EPI_ISL_5647698	2021-06-14	RHD XV	Gamma
EPI_ISL_5647715	2021-06-14	RHD XV	Gamma
EPI_ISL_5647733	2021-06-14	RHD XV	Gamma
EPI_ISL_5647736	2021-06-14	RHD XV	Gamma
EPI_ISL_5647754	2021-06-14	RHD XV	Gamma
EPI_ISL_5647757	2021-06-14	RHD XV	Gamma
EPI_ISL_5647759	2021-06-14	RHD XV	Gamma
EPI_ISL_5647762	2021-06-14	RHD XV	Gamma
EPI_ISL_5647776	2021-06-14	RHD XV	Gamma
EPI_ISL_5647779	2021-06-14	RHD XV	Gamma
EPI_ISL_5647781	2021-06-14	RHD XV	Gamma
EPI_ISL_5647786	2021-06-14	RHD XV	Gamma

EPI_ISL_5647787	2021-06-14	RHD XV	Gamma
EPI_ISL_5647790	2021-06-14	RHD XV	Gamma
EPI_ISL_5647792	2021-06-14	RHD XV	Gamma
EPI_ISL_5647793	2021-06-14	RHD XV	Gamma
EPI_ISL_5647800	2021-06-14	RHD XV	Gamma
EPI_ISL_5647803	2021-06-14	RHD XV	Gamma
EPI_ISL_5647811	2021-06-14	RHD XV	Gamma
EPI_ISL_5647812	2021-06-14	RHD XV	Gamma
EPI_ISL_5647813	2021-06-14	RHD XV	Gamma
EPI_ISL_5647831	2021-06-14	RHD XV	Gamma
EPI_ISL_5647832	2021-06-14	RHD XV	Gamma
EPI_ISL_5647835	2021-06-14	RHD XV	Gamma
EPI_ISL_5647836	2021-06-14	RHD XV	Gamma
EPI_ISL_5647848	2021-06-14	RHD XV	Gamma
EPI_ISL_5647850	2021-06-14	RHD XV	Gamma
EPI_ISL_5647852	2021-06-14	RHD XV	Gamma
EPI_ISL_5647859	2021-06-14	RHD XV	Gamma
EPI_ISL_5647860	2021-06-14	RHD XV	Gamma
EPI_ISL_5647958	2021-06-14	RHD XV	Gamma
EPI_ISL_5647962	2021-06-14	RHD XV	Gamma
EPI_ISL_5647968	2021-06-14	RHD XV	Gamma
EPI_ISL_5647979	2021-06-14	RHD XV	Gamma
EPI_ISL_5647983	2021-06-14	RHD XV	Gamma
EPI_ISL_5647999	2021-06-14	RHD XV	Gamma
EPI_ISL_5648011	2021-06-14	RHD XV	Gamma
EPI_ISL_5648032	2021-06-14	RHD XV	Gamma
EPI_ISL_5648033	2021-06-14	RHD XV	Gamma
EPI_ISL_5648038	2021-06-14	RHD XV	Gamma
EPI_ISL_5648044	2021-06-14	RHD XV	Gamma
EPI_ISL_5648050	2021-06-14	RHD XV	Gamma
EPI_ISL_5648131	2021-06-14	RHD XV	Gamma
EPI_ISL_5648138	2021-06-14	RHD XV	Gamma
EPI_ISL_5648140	2021-06-14	RHD XV	Gamma
EPI_ISL_5648141	2021-06-14	RHD XV	Gamma
EPI_ISL_5648144	2021-06-14	RHD XV	Gamma
EPI_ISL_5648145	2021-06-14	RHD XV	Gamma
EPI_ISL_5648146	2021-06-14	RHD XV	Gamma
EPI_ISL_5648147	2021-06-14	RHD XV	Gamma
EPI_ISL_5648149	2021-06-14	RHD XV	Gamma
EPI_ISL_5648150	2021-06-14	RHD XV	Gamma
EPI_ISL_5648151	2021-06-14	RHD XV	Gamma
EPI_ISL_5648153	2021-06-14	RHD XV	Gamma
EPI_ISL_5648155	2021-06-14	RHD XV	Gamma
EPI_ISL_5648156	2021-06-14	RHD XV	Gamma
EPI_ISL_5648157	2021-06-14	RHD XV	Gamma
EPI_ISL_5648158	2021-06-14	RHD XV	Gamma
EPI_ISL_5648159	2021-06-14	RHD XV	Gamma
EPI_ISL_5648171	2021-06-14	RHD XV	Gamma
EPI_ISL_5648173	2021-06-14	RHD XV	Gamma
EPI_ISL_5648175	2021-06-14	RHD XV	Gamma
EPI_ISL_5648180	2021-06-14	RHD XV	Gamma
EPI_ISL_5648184	2021-06-14	RHD XV	Gamma
EPI_ISL_5648185	2021-06-14	RHD XV	Gamma
EPI_ISL_5648188	2021-06-14	RHD XV	Gamma
EPI_ISL_5648195	2021-06-14	RHD XV	Gamma
EPI_ISL_5648196	2021-06-14	RHD XV	Gamma
EPI_ISL_5648197	2021-06-14	RHD XV	Gamma
EPI_ISL_5648198	2021-06-14	RHD XV	Gamma
EPI_ISL_5648200	2021-06-14	RHD XV	Gamma
EPI_ISL_5648202	2021-06-14	RHD XV	Gamma
EPI_ISL_5648206	2021-06-14	RHD XV	Gamma
EPI_ISL_5648207	2021-06-14	RHD XV	Gamma
EPI_ISL_5648208	2021-06-14	RHD XV	Gamma

EPI_ISL_5648209	2021-06-14	RHD XV	Gamma
EPI_ISL_5648212	2021-06-14	RHD XV	Gamma
EPI_ISL_5648214	2021-06-14	RHD XV	Gamma
EPI_ISL_5648215	2021-06-14	RHD XV	Gamma
EPI_ISL_5648216	2021-06-14	RHD XV	Gamma
EPI_ISL_5648217	2021-06-14	RHD XV	Gamma
EPI_ISL_5648218	2021-06-14	RHD XV	Gamma
EPI_ISL_5648219	2021-06-14	RHD XV	Gamma
EPI_ISL_5648220	2021-06-14	RHD XV	Gamma
EPI_ISL_5648221	2021-06-14	RHD XV	Gamma
EPI_ISL_5648223	2021-06-14	RHD XV	Gamma
EPI_ISL_3055544	2021-06-14	RHD XV	Gamma
EPI_ISL_3827994	2021-06-14	Southeast	Gamma
EPI_ISL_5647657	2021-06-14	RHD XV	Others
EPI_ISL_3254519	2021-06-15	Midwest	Gamma
EPI_ISL_3536223	2021-06-15	Northeast	Gamma
EPI_ISL_5647676	2021-06-15	RHD XV	Gamma
EPI_ISL_5647687	2021-06-15	RHD XV	Gamma
EPI_ISL_5647697	2021-06-15	RHD XV	Gamma
EPI_ISL_5647704	2021-06-15	RHD XV	Gamma
EPI_ISL_5647710	2021-06-15	RHD XV	Gamma
EPI_ISL_5647716	2021-06-15	RHD XV	Gamma
EPI_ISL_5647717	2021-06-15	RHD XV	Gamma
EPI_ISL_5647721	2021-06-15	RHD XV	Gamma
EPI_ISL_5647724	2021-06-15	RHD XV	Gamma
EPI_ISL_5647729	2021-06-15	RHD XV	Gamma
EPI_ISL_5647731	2021-06-15	RHD XV	Gamma
EPI_ISL_5647760	2021-06-15	RHD XV	Gamma
EPI_ISL_5647761	2021-06-15	RHD XV	Gamma
EPI_ISL_5647774	2021-06-15	RHD XV	Gamma
EPI_ISL_5647778	2021-06-15	RHD XV	Gamma
EPI_ISL_5647791	2021-06-15	RHD XV	Gamma
EPI_ISL_5647807	2021-06-15	RHD XV	Gamma
EPI_ISL_5647820	2021-06-15	RHD XV	Gamma
EPI_ISL_5647843	2021-06-15	RHD XV	Gamma
EPI_ISL_5648134	2021-06-15	RHD XV	Gamma
EPI_ISL_5648135	2021-06-15	RHD XV	Gamma
EPI_ISL_5648139	2021-06-15	RHD XV	Gamma
EPI_ISL_5648174	2021-06-15	RHD XV	Gamma
EPI_ISL_5648177	2021-06-15	RHD XV	Gamma
EPI_ISL_5648210	2021-06-15	RHD XV	Gamma
EPI_ISL_5648211	2021-06-15	RHD XV	Gamma
EPI_ISL_3049002	2021-06-15	RHD XV	Gamma
EPI_ISL_3049003	2021-06-15	RHD XV	Gamma
EPI_ISL_3049004	2021-06-15	RHD XV	Gamma
EPI_ISL_3049005	2021-06-15	RHD XV	Gamma
EPI_ISL_3050433	2021-06-16	North	Gamma
EPI_ISL_2983422	2021-06-16	North	Gamma
EPI_ISL_3049006	2021-06-16	RHD XV	Gamma
EPI_ISL_3049007	2021-06-16	RHD XV	Gamma
EPI_ISL_3761753	2021-06-16	RHD XV	Gamma
EPI_ISL_3049008	2021-06-16	RHD XV	Gamma
EPI_ISL_3055542	2021-06-16	RHD XV	Gamma
EPI_ISL_3049009	2021-06-16	RHD XV	Gamma
EPI_ISL_3049010	2021-06-16	RHD XV	Gamma
EPI_ISL_3434957	2021-06-17	North	Gamma
EPI_ISL_2983418	2021-06-17	North	Gamma
EPI_ISL_3049011	2021-06-17	RHD XV	Gamma
EPI_ISL_3049012	2021-06-17	RHD XV	Gamma
EPI_ISL_3259737	2021-06-17	Southeast	Gamma
EPI_ISL_2896237	2021-06-18	Midwest	Gamma
EPI_ISL_3045505	2021-06-18	Southeast	Gamma
EPI_ISL_3434959	2021-06-20	North	Gamma

EPI_ISL_3190322	2021-06-20	Northeast	Gamma
EPI_ISL_5659563	2021-06-20	RHD XV	Gamma
EPI_ISL_5659593	2021-06-20	RHD XV	Gamma
EPI_ISL_3050514	2021-06-21	North	Gamma
EPI_ISL_5648266	2021-06-21	RHD XV	Gamma
EPI_ISL_5648278	2021-06-21	RHD XV	Gamma
EPI_ISL_5648305	2021-06-21	RHD XV	Gamma
EPI_ISL_5648388	2021-06-21	RHD XV	Gamma
EPI_ISL_5648434	2021-06-21	RHD XV	Gamma
EPI_ISL_5648437	2021-06-21	RHD XV	Gamma
EPI_ISL_5648461	2021-06-21	RHD XV	Gamma
EPI_ISL_5648488	2021-06-21	RHD XV	Gamma
EPI_ISL_5648498	2021-06-21	RHD XV	Gamma
EPI_ISL_5648505	2021-06-21	RHD XV	Gamma
EPI_ISL_5648508	2021-06-21	RHD XV	Gamma
EPI_ISL_5648572	2021-06-21	RHD XV	Gamma
EPI_ISL_5648580	2021-06-21	RHD XV	Gamma
EPI_ISL_5648581	2021-06-21	RHD XV	Gamma
EPI_ISL_5648588	2021-06-21	RHD XV	Gamma
EPI_ISL_5648592	2021-06-21	RHD XV	Gamma
EPI_ISL_5648595	2021-06-21	RHD XV	Gamma
EPI_ISL_5648598	2021-06-21	RHD XV	Gamma
EPI_ISL_5648606	2021-06-21	RHD XV	Gamma
EPI_ISL_5648612	2021-06-21	RHD XV	Gamma
EPI_ISL_5648655	2021-06-21	RHD XV	Gamma
EPI_ISL_5648663	2021-06-21	RHD XV	Gamma
EPI_ISL_5648691	2021-06-21	RHD XV	Gamma
EPI_ISL_5648699	2021-06-21	RHD XV	Gamma
EPI_ISL_5648728	2021-06-21	RHD XV	Gamma
EPI_ISL_5648732	2021-06-21	RHD XV	Gamma
EPI_ISL_5648733	2021-06-21	RHD XV	Gamma
EPI_ISL_5648741	2021-06-21	RHD XV	Gamma
EPI_ISL_5648745	2021-06-21	RHD XV	Gamma
EPI_ISL_5648746	2021-06-21	RHD XV	Gamma
EPI_ISL_5648750	2021-06-21	RHD XV	Gamma
EPI_ISL_5648752	2021-06-21	RHD XV	Gamma
EPI_ISL_5648767	2021-06-21	RHD XV	Gamma
EPI_ISL_5648772	2021-06-21	RHD XV	Gamma
EPI_ISL_5648779	2021-06-21	RHD XV	Gamma
EPI_ISL_5648800	2021-06-21	RHD XV	Gamma
EPI_ISL_5648809	2021-06-21	RHD XV	Gamma
EPI_ISL_5648861	2021-06-21	RHD XV	Gamma
EPI_ISL_5648872	2021-06-21	RHD XV	Gamma
EPI_ISL_5648907	2021-06-21	RHD XV	Gamma
EPI_ISL_5648938	2021-06-21	RHD XV	Gamma
EPI_ISL_5648943	2021-06-21	RHD XV	Gamma
EPI_ISL_5648971	2021-06-21	RHD XV	Gamma
EPI_ISL_5648972	2021-06-21	RHD XV	Gamma
EPI_ISL_5648978	2021-06-21	RHD XV	Gamma
EPI_ISL_5648980	2021-06-21	RHD XV	Gamma
EPI_ISL_5649005	2021-06-21	RHD XV	Gamma
EPI_ISL_5649015	2021-06-21	RHD XV	Gamma
EPI_ISL_5649026	2021-06-21	RHD XV	Gamma
EPI_ISL_5649031	2021-06-21	RHD XV	Gamma
EPI_ISL_5649038	2021-06-21	RHD XV	Gamma
EPI_ISL_5649039	2021-06-21	RHD XV	Gamma
EPI_ISL_5649051	2021-06-21	RHD XV	Gamma
EPI_ISL_5649052	2021-06-21	RHD XV	Gamma
EPI_ISL_5649083	2021-06-21	RHD XV	Gamma
EPI_ISL_5649136	2021-06-21	RHD XV	Gamma
EPI_ISL_5649139	2021-06-21	RHD XV	Gamma
EPI_ISL_5649433	2021-06-21	RHD XV	Gamma
EPI_ISL_5649445	2021-06-21	RHD XV	Gamma

EPI_ISL_5649457	2021-06-21	RHD XV	Gamma
EPI_ISL_5649464	2021-06-21	RHD XV	Gamma
EPI_ISL_5649465	2021-06-21	RHD XV	Gamma
EPI_ISL_5649484	2021-06-21	RHD XV	Gamma
EPI_ISL_5659520	2021-06-21	RHD XV	Gamma
EPI_ISL_5659533	2021-06-21	RHD XV	Gamma
EPI_ISL_5659536	2021-06-21	RHD XV	Gamma
EPI_ISL_5659537	2021-06-21	RHD XV	Gamma
EPI_ISL_5659538	2021-06-21	RHD XV	Gamma
EPI_ISL_5659539	2021-06-21	RHD XV	Gamma
EPI_ISL_5659541	2021-06-21	RHD XV	Gamma
EPI_ISL_5659542	2021-06-21	RHD XV	Gamma
EPI_ISL_5659543	2021-06-21	RHD XV	Gamma
EPI_ISL_5659544	2021-06-21	RHD XV	Gamma
EPI_ISL_5659545	2021-06-21	RHD XV	Gamma
EPI_ISL_5659546	2021-06-21	RHD XV	Gamma
EPI_ISL_5659547	2021-06-21	RHD XV	Gamma
EPI_ISL_5659548	2021-06-21	RHD XV	Gamma
EPI_ISL_5659549	2021-06-21	RHD XV	Gamma
EPI_ISL_5659550	2021-06-21	RHD XV	Gamma
EPI_ISL_5659551	2021-06-21	RHD XV	Gamma
EPI_ISL_5659554	2021-06-21	RHD XV	Gamma
EPI_ISL_5659555	2021-06-21	RHD XV	Gamma
EPI_ISL_5659556	2021-06-21	RHD XV	Gamma
EPI_ISL_5659557	2021-06-21	RHD XV	Gamma
EPI_ISL_5659558	2021-06-21	RHD XV	Gamma
EPI_ISL_5659559	2021-06-21	RHD XV	Gamma
EPI_ISL_5659560	2021-06-21	RHD XV	Gamma
EPI_ISL_5659561	2021-06-21	RHD XV	Gamma
EPI_ISL_5659562	2021-06-21	RHD XV	Gamma
EPI_ISL_5659564	2021-06-21	RHD XV	Gamma
EPI_ISL_5659565	2021-06-21	RHD XV	Gamma
EPI_ISL_5659567	2021-06-21	RHD XV	Gamma
EPI_ISL_5659568	2021-06-21	RHD XV	Gamma
EPI_ISL_5659569	2021-06-21	RHD XV	Gamma
EPI_ISL_5659570	2021-06-21	RHD XV	Gamma
EPI_ISL_5659571	2021-06-21	RHD XV	Gamma
EPI_ISL_5659573	2021-06-21	RHD XV	Gamma
EPI_ISL_5659574	2021-06-21	RHD XV	Gamma
EPI_ISL_5659575	2021-06-21	RHD XV	Gamma
EPI_ISL_5659577	2021-06-21	RHD XV	Gamma
EPI_ISL_5659579	2021-06-21	RHD XV	Gamma
EPI_ISL_5659580	2021-06-21	RHD XV	Gamma
EPI_ISL_5659582	2021-06-21	RHD XV	Gamma
EPI_ISL_5659583	2021-06-21	RHD XV	Gamma
EPI_ISL_5659584	2021-06-21	RHD XV	Gamma
EPI_ISL_5659585	2021-06-21	RHD XV	Gamma
EPI_ISL_5659587	2021-06-21	RHD XV	Gamma
EPI_ISL_5659588	2021-06-21	RHD XV	Gamma
EPI_ISL_5659589	2021-06-21	RHD XV	Gamma
EPI_ISL_5659590	2021-06-21	RHD XV	Gamma
EPI_ISL_5659591	2021-06-21	RHD XV	Gamma
EPI_ISL_5659592	2021-06-21	RHD XV	Gamma
EPI_ISL_5659595	2021-06-21	RHD XV	Gamma
EPI_ISL_5659596	2021-06-21	RHD XV	Gamma
EPI_ISL_5659597	2021-06-21	RHD XV	Gamma
EPI_ISL_5659600	2021-06-21	RHD XV	Gamma
EPI_ISL_5659601	2021-06-21	RHD XV	Gamma
EPI_ISL_5659603	2021-06-21	RHD XV	Gamma
EPI_ISL_5659604	2021-06-21	RHD XV	Gamma
EPI_ISL_5659605	2021-06-21	RHD XV	Gamma
EPI_ISL_5659607	2021-06-21	RHD XV	Gamma
EPI_ISL_5659609	2021-06-21	RHD XV	Gamma

EPI_ISL_5659610	2021-06-21	RHD XV	Gamma
EPI_ISL_5659611	2021-06-21	RHD XV	Gamma
EPI_ISL_5659612	2021-06-21	RHD XV	Gamma
EPI_ISL_5659616	2021-06-21	RHD XV	Gamma
EPI_ISL_5659617	2021-06-21	RHD XV	Gamma
EPI_ISL_5659618	2021-06-21	RHD XV	Gamma
EPI_ISL_5659619	2021-06-21	RHD XV	Gamma
EPI_ISL_5659620	2021-06-21	RHD XV	Gamma
EPI_ISL_5659621	2021-06-21	RHD XV	Gamma
EPI_ISL_5659622	2021-06-21	RHD XV	Gamma
EPI_ISL_5659623	2021-06-21	RHD XV	Gamma
EPI_ISL_5659624	2021-06-21	RHD XV	Gamma
EPI_ISL_5659625	2021-06-21	RHD XV	Gamma
EPI_ISL_5659627	2021-06-21	RHD XV	Gamma
EPI_ISL_5659628	2021-06-21	RHD XV	Gamma
EPI_ISL_5659629	2021-06-21	RHD XV	Gamma
EPI_ISL_5659630	2021-06-21	RHD XV	Gamma
EPI_ISL_5659631	2021-06-21	RHD XV	Gamma
EPI_ISL_5659632	2021-06-21	RHD XV	Gamma
EPI_ISL_5659633	2021-06-21	RHD XV	Gamma
EPI_ISL_5659634	2021-06-21	RHD XV	Gamma
EPI_ISL_5659635	2021-06-21	RHD XV	Gamma
EPI_ISL_5659636	2021-06-21	RHD XV	Gamma
EPI_ISL_5659637	2021-06-21	RHD XV	Gamma
EPI_ISL_5659638	2021-06-21	RHD XV	Gamma
EPI_ISL_5659640	2021-06-21	RHD XV	Gamma
EPI_ISL_3049013	2021-06-21	RHD XV	Gamma
EPI_ISL_3049014	2021-06-21	RHD XV	Gamma
EPI_ISL_3049015	2021-06-21	RHD XV	Gamma
EPI_ISL_3049016	2021-06-21	RHD XV	Gamma
EPI_ISL_3049017	2021-06-21	RHD XV	Gamma
EPI_ISL_3049018	2021-06-21	RHD XV	Gamma
EPI_ISL_3049019	2021-06-21	RHD XV	Gamma
EPI_ISL_3049020	2021-06-21	RHD XV	Gamma
EPI_ISL_3761647	2021-06-21	RHD XV	Gamma
EPI_ISL_3055529	2021-06-21	RHD XV	Gamma
EPI_ISL_3761648	2021-06-21	RHD XV	Gamma
EPI_ISL_3761649	2021-06-21	RHD XV	Gamma
EPI_ISL_3828011	2021-06-21	Southeast	Gamma
EPI_ISL_3259744	2021-06-21	Southeast	Gamma
EPI_ISL_2894547	2021-06-22	Midwest	Gamma
EPI_ISL_2896234	2021-06-22	Midwest	Gamma
EPI_ISL_5193524	2021-06-22	North	Gamma
EPI_ISL_5648381	2021-06-22	RHD XV	Gamma
EPI_ISL_5648438	2021-06-22	RHD XV	Gamma
EPI_ISL_5648449	2021-06-22	RHD XV	Gamma
EPI_ISL_5648484	2021-06-22	RHD XV	Gamma
EPI_ISL_5648503	2021-06-22	RHD XV	Gamma
EPI_ISL_5648546	2021-06-22	RHD XV	Gamma
EPI_ISL_5648570	2021-06-22	RHD XV	Gamma
EPI_ISL_5648623	2021-06-22	RHD XV	Gamma
EPI_ISL_5648624	2021-06-22	RHD XV	Gamma
EPI_ISL_5648670	2021-06-22	RHD XV	Gamma
EPI_ISL_5648722	2021-06-22	RHD XV	Gamma
EPI_ISL_5648776	2021-06-22	RHD XV	Gamma
EPI_ISL_5648806	2021-06-22	RHD XV	Gamma
EPI_ISL_5648837	2021-06-22	RHD XV	Gamma
EPI_ISL_5648839	2021-06-22	RHD XV	Gamma
EPI_ISL_5648898	2021-06-22	RHD XV	Gamma
EPI_ISL_5648964	2021-06-22	RHD XV	Gamma
EPI_ISL_5649032	2021-06-22	RHD XV	Gamma
EPI_ISL_5649101	2021-06-22	RHD XV	Gamma
EPI_ISL_5649135	2021-06-22	RHD XV	Gamma

EPI_ISL_5659552	2021-06-22	RHD XV	Gamma
EPI_ISL_5659553	2021-06-22	RHD XV	Gamma
EPI_ISL_5659566	2021-06-22	RHD XV	Gamma
EPI_ISL_5659572	2021-06-22	RHD XV	Gamma
EPI_ISL_5659576	2021-06-22	RHD XV	Gamma
EPI_ISL_5659578	2021-06-22	RHD XV	Gamma
EPI_ISL_5659581	2021-06-22	RHD XV	Gamma
EPI_ISL_5659586	2021-06-22	RHD XV	Gamma
EPI_ISL_5659594	2021-06-22	RHD XV	Gamma
EPI_ISL_5659598	2021-06-22	RHD XV	Gamma
EPI_ISL_5659599	2021-06-22	RHD XV	Gamma
EPI_ISL_5659602	2021-06-22	RHD XV	Gamma
EPI_ISL_5659606	2021-06-22	RHD XV	Gamma
EPI_ISL_5659608	2021-06-22	RHD XV	Gamma
EPI_ISL_5659626	2021-06-22	RHD XV	Gamma
EPI_ISL_5659639	2021-06-22	RHD XV	Gamma
EPI_ISL_3761650	2021-06-22	RHD XV	Gamma
EPI_ISL_3761651	2021-06-22	RHD XV	Gamma
EPI_ISL_3761652	2021-06-22	RHD XV	Gamma
EPI_ISL_3761653	2021-06-22	RHD XV	Gamma
EPI_ISL_3761654	2021-06-22	RHD XV	Gamma
EPI_ISL_3761655	2021-06-22	RHD XV	Gamma
EPI_ISL_3761656	2021-06-22	RHD XV	Gamma
EPI_ISL_3761657	2021-06-22	RHD XV	Gamma
EPI_ISL_3190244	2021-06-22	Southeast	Gamma
EPI_ISL_5195473	2021-06-22	Southeast	Gamma
EPI_ISL_2896239	2021-06-23	Midwest	Gamma
EPI_ISL_3761587	2021-06-23	RHD XV	Gamma
EPI_ISL_3761658	2021-06-23	RHD XV	Gamma
EPI_ISL_3761659	2021-06-23	RHD XV	Gamma
EPI_ISL_3761660	2021-06-23	RHD XV	Gamma
EPI_ISL_3761661	2021-06-23	RHD XV	Gamma
EPI_ISL_3761662	2021-06-23	RHD XV	Gamma
EPI_ISL_3761663	2021-06-23	RHD XV	Gamma
EPI_ISL_3761599	2021-06-23	RHD XV	Gamma
EPI_ISL_3761664	2021-06-23	RHD XV	Gamma
EPI_ISL_3761535	2021-06-23	RHD XV	Gamma
EPI_ISL_3761665	2021-06-24	RHD XV	Gamma
EPI_ISL_3761666	2021-06-24	RHD XV	Gamma
EPI_ISL_3761667	2021-06-24	RHD XV	Gamma
EPI_ISL_3761668	2021-06-24	RHD XV	Gamma
EPI_ISL_3761669	2021-06-24	RHD XV	Gamma
EPI_ISL_3761670	2021-06-24	RHD XV	Gamma
EPI_ISL_3761671	2021-06-24	RHD XV	Gamma
EPI_ISL_3761672	2021-06-24	RHD XV	Gamma
EPI_ISL_3761673	2021-06-24	RHD XV	Gamma
EPI_ISL_8005541	2021-06-24	South	Gamma
EPI_ISL_2982735	2021-06-25	Southeast	Delta
EPI_ISL_3190330	2021-06-25	Northeast	Gamma
EPI_ISL_3190246	2021-06-25	Southeast	Gamma
EPI_ISL_3316270	2021-06-25	Southeast	Gamma
EPI_ISL_3801852	2021-06-26	South	Delta
EPI_ISL_3254590	2021-06-26	Midwest	Gamma
EPI_ISL_3190333	2021-06-26	Northeast	Gamma
EPI_ISL_4487679	2021-06-26	Northeast	Gamma
EPI_ISL_3245340	2021-06-26	Southeast	Gamma
EPI_ISL_3539747	2021-06-27	North	Gamma
EPI_ISL_5666225	2021-06-27	RHD XV	Gamma
EPI_ISL_3190411	2021-06-28	North	Gamma
EPI_ISL_3190392	2021-06-28	Northeast	Gamma
EPI_ISL_5659656	2021-06-28	RHD XV	Gamma
EPI_ISL_5659686	2021-06-28	RHD XV	Gamma
EPI_ISL_5660317	2021-06-28	RHD XV	Gamma

EPI_ISL_5660351	2021-06-28	RHD XV	Gamma
EPI_ISL_5660447	2021-06-28	RHD XV	Gamma
EPI_ISL_5660670	2021-06-28	RHD XV	Gamma
EPI_ISL_5661059	2021-06-28	RHD XV	Gamma
EPI_ISL_5661108	2021-06-28	RHD XV	Gamma
EPI_ISL_5661123	2021-06-28	RHD XV	Gamma
EPI_ISL_5666184	2021-06-28	RHD XV	Gamma
EPI_ISL_5666189	2021-06-28	RHD XV	Gamma
EPI_ISL_5666208	2021-06-28	RHD XV	Gamma
EPI_ISL_5666213	2021-06-28	RHD XV	Gamma
EPI_ISL_5666237	2021-06-28	RHD XV	Gamma
EPI_ISL_5666249	2021-06-28	RHD XV	Gamma
EPI_ISL_5666253	2021-06-28	RHD XV	Gamma
EPI_ISL_5666274	2021-06-28	RHD XV	Gamma
EPI_ISL_5666281	2021-06-28	RHD XV	Gamma
EPI_ISL_5666312	2021-06-28	RHD XV	Gamma
EPI_ISL_5666322	2021-06-28	RHD XV	Gamma
EPI_ISL_5666329	2021-06-28	RHD XV	Gamma
EPI_ISL_5666335	2021-06-28	RHD XV	Gamma
EPI_ISL_5666342	2021-06-28	RHD XV	Gamma
EPI_ISL_5666390	2021-06-28	RHD XV	Gamma
EPI_ISL_5666400	2021-06-28	RHD XV	Gamma
EPI_ISL_5666403	2021-06-28	RHD XV	Gamma
EPI_ISL_5666414	2021-06-28	RHD XV	Gamma
EPI_ISL_5666427	2021-06-28	RHD XV	Gamma
EPI_ISL_5666434	2021-06-28	RHD XV	Gamma
EPI_ISL_5666437	2021-06-28	RHD XV	Gamma
EPI_ISL_5666469	2021-06-28	RHD XV	Gamma
EPI_ISL_5666479	2021-06-28	RHD XV	Gamma
EPI_ISL_5666487	2021-06-28	RHD XV	Gamma
EPI_ISL_5666494	2021-06-28	RHD XV	Gamma
EPI_ISL_5666502	2021-06-28	RHD XV	Gamma
EPI_ISL_5666509	2021-06-28	RHD XV	Gamma
EPI_ISL_5666517	2021-06-28	RHD XV	Gamma
EPI_ISL_5666531	2021-06-28	RHD XV	Gamma
EPI_ISL_5666540	2021-06-28	RHD XV	Gamma
EPI_ISL_5666564	2021-06-28	RHD XV	Gamma
EPI_ISL_5666569	2021-06-28	RHD XV	Gamma
EPI_ISL_5666585	2021-06-28	RHD XV	Gamma
EPI_ISL_5666601	2021-06-28	RHD XV	Gamma
EPI_ISL_5666608	2021-06-28	RHD XV	Gamma
EPI_ISL_5666619	2021-06-28	RHD XV	Gamma
EPI_ISL_5666629	2021-06-28	RHD XV	Gamma
EPI_ISL_5667268	2021-06-28	RHD XV	Gamma
EPI_ISL_5667282	2021-06-28	RHD XV	Gamma
EPI_ISL_5667291	2021-06-28	RHD XV	Gamma
EPI_ISL_5667297	2021-06-28	RHD XV	Gamma
EPI_ISL_5667315	2021-06-28	RHD XV	Gamma
EPI_ISL_5667318	2021-06-28	RHD XV	Gamma
EPI_ISL_5667325	2021-06-28	RHD XV	Gamma
EPI_ISL_5667337	2021-06-28	RHD XV	Gamma
EPI_ISL_5667356	2021-06-28	RHD XV	Gamma
EPI_ISL_5667368	2021-06-28	RHD XV	Gamma
EPI_ISL_5667381	2021-06-28	RHD XV	Gamma
EPI_ISL_5667387	2021-06-28	RHD XV	Gamma
EPI_ISL_5667393	2021-06-28	RHD XV	Gamma
EPI_ISL_5667406	2021-06-28	RHD XV	Gamma
EPI_ISL_5667423	2021-06-28	RHD XV	Gamma
EPI_ISL_5667431	2021-06-28	RHD XV	Gamma
EPI_ISL_5667457	2021-06-28	RHD XV	Gamma
EPI_ISL_5667490	2021-06-28	RHD XV	Gamma
EPI_ISL_5667495	2021-06-28	RHD XV	Gamma
EPI_ISL_5667507	2021-06-28	RHD XV	Gamma

EPI_ISL_5667526	2021-06-28	RHD XV	Gamma
EPI_ISL_5667534	2021-06-28	RHD XV	Gamma
EPI_ISL_5667543	2021-06-28	RHD XV	Gamma
EPI_ISL_5667562	2021-06-28	RHD XV	Gamma
EPI_ISL_5667580	2021-06-28	RHD XV	Gamma
EPI_ISL_5667593	2021-06-28	RHD XV	Gamma
EPI_ISL_5667605	2021-06-28	RHD XV	Gamma
EPI_ISL_5667652	2021-06-28	RHD XV	Gamma
EPI_ISL_5667682	2021-06-28	RHD XV	Gamma
EPI_ISL_5667685	2021-06-28	RHD XV	Gamma
EPI_ISL_5667687	2021-06-28	RHD XV	Gamma
EPI_ISL_5667698	2021-06-28	RHD XV	Gamma
EPI_ISL_5667708	2021-06-28	RHD XV	Gamma
EPI_ISL_5667723	2021-06-28	RHD XV	Gamma
EPI_ISL_5667727	2021-06-28	RHD XV	Gamma
EPI_ISL_5667737	2021-06-28	RHD XV	Gamma
EPI_ISL_5667747	2021-06-28	RHD XV	Gamma
EPI_ISL_5667752	2021-06-28	RHD XV	Gamma
EPI_ISL_5667757	2021-06-28	RHD XV	Gamma
EPI_ISL_5667763	2021-06-28	RHD XV	Gamma
EPI_ISL_5667773	2021-06-28	RHD XV	Gamma
EPI_ISL_5667776	2021-06-28	RHD XV	Gamma
EPI_ISL_5667783	2021-06-28	RHD XV	Gamma
EPI_ISL_5667794	2021-06-28	RHD XV	Gamma
EPI_ISL_5667870	2021-06-28	RHD XV	Gamma
EPI_ISL_5667949	2021-06-28	RHD XV	Gamma
EPI_ISL_5667962	2021-06-28	RHD XV	Gamma
EPI_ISL_5667977	2021-06-28	RHD XV	Gamma
EPI_ISL_5668014	2021-06-28	RHD XV	Gamma
EPI_ISL_5668168	2021-06-28	RHD XV	Gamma
EPI_ISL_5668212	2021-06-28	RHD XV	Gamma
EPI_ISL_5668300	2021-06-28	RHD XV	Gamma
EPI_ISL_5668369	2021-06-28	RHD XV	Gamma
EPI_ISL_5668378	2021-06-28	RHD XV	Gamma
EPI_ISL_11940591	2021-06-28	RHD XV	Gamma
EPI_ISL_3761754	2021-06-28	RHD XV	Gamma
EPI_ISL_5195477	2021-06-28	Southeast	Gamma
EPI_ISL_5659660	2021-06-29	RHD XV	Gamma
EPI_ISL_5659819	2021-06-29	RHD XV	Gamma
EPI_ISL_5659939	2021-06-29	RHD XV	Gamma
EPI_ISL_5660106	2021-06-29	RHD XV	Gamma
EPI_ISL_5660147	2021-06-29	RHD XV	Gamma
EPI_ISL_5660203	2021-06-29	RHD XV	Gamma
EPI_ISL_5660292	2021-06-29	RHD XV	Gamma
EPI_ISL_5660631	2021-06-29	RHD XV	Gamma
EPI_ISL_5660878	2021-06-29	RHD XV	Gamma
EPI_ISL_5660981	2021-06-29	RHD XV	Gamma
EPI_ISL_5666199	2021-06-29	RHD XV	Gamma
EPI_ISL_5666265	2021-06-29	RHD XV	Gamma
EPI_ISL_5666349	2021-06-29	RHD XV	Gamma
EPI_ISL_5666374	2021-06-29	RHD XV	Gamma
EPI_ISL_5666557	2021-06-29	RHD XV	Gamma
EPI_ISL_5667374	2021-06-29	RHD XV	Gamma
EPI_ISL_5667399	2021-06-29	RHD XV	Gamma
EPI_ISL_5667445	2021-06-29	RHD XV	Gamma
EPI_ISL_5667572	2021-06-29	RHD XV	Gamma
EPI_ISL_5667654	2021-06-29	RHD XV	Gamma
EPI_ISL_5667702	2021-06-29	RHD XV	Gamma
EPI_ISL_5667803	2021-06-29	RHD XV	Gamma
EPI_ISL_5667809	2021-06-29	RHD XV	Gamma
EPI_ISL_5667887	2021-06-29	RHD XV	Gamma
EPI_ISL_5667902	2021-06-29	RHD XV	Gamma
EPI_ISL_5667941	2021-06-29	RHD XV	Gamma

EPI_ISL_5667983	2021-06-29	RHD XV	Gamma
EPI_ISL_5668123	2021-06-29	RHD XV	Gamma
EPI_ISL_5668145	2021-06-29	RHD XV	Gamma
EPI_ISL_5668151	2021-06-29	RHD XV	Gamma
EPI_ISL_5668253	2021-06-29	RHD XV	Gamma
EPI_ISL_5668347	2021-06-29	RHD XV	Gamma
EPI_ISL_5668359	2021-06-29	RHD XV	Gamma
EPI_ISL_5668375	2021-06-29	RHD XV	Gamma
EPI_ISL_5668394	2021-06-29	RHD XV	Gamma
EPI_ISL_5668402	2021-06-29	RHD XV	Gamma
EPI_ISL_3049021	2021-06-29	RHD XV	Gamma
EPI_ISL_3049023	2021-06-29	RHD XV	Gamma
EPI_ISL_3049024	2021-06-29	RHD XV	Gamma
EPI_ISL_3049025	2021-06-29	RHD XV	Gamma
EPI_ISL_3049026	2021-06-29	RHD XV	Gamma
EPI_ISL_3049027	2021-06-29	RHD XV	Gamma
EPI_ISL_3049028	2021-06-29	RHD XV	Gamma
EPI_ISL_3049029	2021-06-29	RHD XV	Gamma
EPI_ISL_3055532	2021-06-29	RHD XV	Gamma
EPI_ISL_3055533	2021-06-29	RHD XV	Gamma
EPI_ISL_3049030	2021-06-29	RHD XV	Gamma
EPI_ISL_3761755	2021-06-29	RHD XV	Gamma
EPI_ISL_3761586	2021-06-29	RHD XV	Gamma
EPI_ISL_3761756	2021-06-29	RHD XV	Gamma
EPI_ISL_3050593	2021-06-30	North	Gamma
EPI_ISL_3434968	2021-06-30	North	Gamma
EPI_ISL_6908032	2021-06-30	Northeast	Gamma
EPI_ISL_3761523	2021-06-30	RHD XV	Gamma
EPI_ISL_3761757	2021-06-30	RHD XV	Gamma
EPI_ISL_3761758	2021-06-30	RHD XV	Gamma
EPI_ISL_3761528	2021-06-30	RHD XV	Gamma
EPI_ISL_3761759	2021-06-30	RHD XV	Gamma
EPI_ISL_8005421	2021-06-30	South	Gamma
EPI_ISL_3014464	2021-07-01	Southeast	Delta
EPI_ISL_3050605	2021-07-01	North	Gamma
EPI_ISL_3235314	2021-07-01	North	Gamma
EPI_ISL_3536175	2021-07-01	Northeast	Gamma
EPI_ISL_3046288	2021-07-01	Northeast	Gamma
EPI_ISL_3761563	2021-07-01	RHD XV	Gamma
EPI_ISL_3761522	2021-07-01	RHD XV	Gamma
EPI_ISL_3761674	2021-07-01	RHD XV	Gamma
EPI_ISL_3761588	2021-07-01	RHD XV	Gamma
EPI_ISL_3761675	2021-07-01	RHD XV	Gamma
EPI_ISL_3761558	2021-07-01	RHD XV	Gamma
EPI_ISL_3761676	2021-07-01	RHD XV	Gamma
EPI_ISL_3761539	2021-07-01	RHD XV	Gamma
EPI_ISL_3761677	2021-07-01	RHD XV	Gamma
EPI_ISL_3761678	2021-07-01	RHD XV	Gamma
EPI_ISL_3761679	2021-07-01	RHD XV	Gamma
EPI_ISL_3761680	2021-07-01	RHD XV	Gamma
EPI_ISL_3761573	2021-07-01	RHD XV	Gamma
EPI_ISL_3190384	2021-07-01	South	Gamma
EPI_ISL_5195483	2021-07-01	Southeast	Gamma
EPI_ISL_4061673	2021-07-02	Midwest	Gamma
EPI_ISL_4520384	2021-07-02	Northeast	Gamma
EPI_ISL_13131323	2021-07-03	Midwest	Gamma
EPI_ISL_3235306	2021-07-03	North	Gamma
EPI_ISL_3434835	2021-07-03	Northeast	Gamma
EPI_ISL_3190250	2021-07-03	Southeast	Gamma
EPI_ISL_9800143	2021-07-04	Midwest	Gamma
EPI_ISL_3434970	2021-07-04	North	Gamma
EPI_ISL_7982770	2021-07-04	North	Gamma
EPI_ISL_5673245	2021-07-05	RHD XV	Alpha

EPI_ISL_13131324	2021-07-05	Midwest	Gamma
EPI_ISL_3434838	2021-07-05	Northeast	Gamma
EPI_ISL_5672042	2021-07-05	RHD XV	Gamma
EPI_ISL_5672056	2021-07-05	RHD XV	Gamma
EPI_ISL_5672085	2021-07-05	RHD XV	Gamma
EPI_ISL_5672090	2021-07-05	RHD XV	Gamma
EPI_ISL_5672120	2021-07-05	RHD XV	Gamma
EPI_ISL_5672129	2021-07-05	RHD XV	Gamma
EPI_ISL_5672134	2021-07-05	RHD XV	Gamma
EPI_ISL_5672174	2021-07-05	RHD XV	Gamma
EPI_ISL_5672180	2021-07-05	RHD XV	Gamma
EPI_ISL_5672185	2021-07-05	RHD XV	Gamma
EPI_ISL_5672289	2021-07-05	RHD XV	Gamma
EPI_ISL_5672319	2021-07-05	RHD XV	Gamma
EPI_ISL_5672334	2021-07-05	RHD XV	Gamma
EPI_ISL_5672370	2021-07-05	RHD XV	Gamma
EPI_ISL_5672422	2021-07-05	RHD XV	Gamma
EPI_ISL_5672434	2021-07-05	RHD XV	Gamma
EPI_ISL_5672485	2021-07-05	RHD XV	Gamma
EPI_ISL_5672497	2021-07-05	RHD XV	Gamma
EPI_ISL_5672502	2021-07-05	RHD XV	Gamma
EPI_ISL_5672514	2021-07-05	RHD XV	Gamma
EPI_ISL_5672530	2021-07-05	RHD XV	Gamma
EPI_ISL_5672550	2021-07-05	RHD XV	Gamma
EPI_ISL_5673293	2021-07-05	RHD XV	Gamma
EPI_ISL_5673342	2021-07-05	RHD XV	Gamma
EPI_ISL_5673352	2021-07-05	RHD XV	Gamma
EPI_ISL_5673372	2021-07-05	RHD XV	Gamma
EPI_ISL_5673393	2021-07-05	RHD XV	Gamma
EPI_ISL_5673399	2021-07-05	RHD XV	Gamma
EPI_ISL_5673420	2021-07-05	RHD XV	Gamma
EPI_ISL_5673429	2021-07-05	RHD XV	Gamma
EPI_ISL_5673454	2021-07-05	RHD XV	Gamma
EPI_ISL_5673464	2021-07-05	RHD XV	Gamma
EPI_ISL_5673562	2021-07-05	RHD XV	Gamma
EPI_ISL_5673583	2021-07-05	RHD XV	Gamma
EPI_ISL_5673637	2021-07-05	RHD XV	Gamma
EPI_ISL_5673654	2021-07-05	RHD XV	Gamma
EPI_ISL_5673679	2021-07-05	RHD XV	Gamma
EPI_ISL_5673686	2021-07-05	RHD XV	Gamma
EPI_ISL_5673714	2021-07-05	RHD XV	Gamma
EPI_ISL_5673742	2021-07-05	RHD XV	Gamma
EPI_ISL_5673758	2021-07-05	RHD XV	Gamma
EPI_ISL_5673773	2021-07-05	RHD XV	Gamma
EPI_ISL_5673807	2021-07-05	RHD XV	Gamma
EPI_ISL_5673867	2021-07-05	RHD XV	Gamma
EPI_ISL_5673883	2021-07-05	RHD XV	Gamma
EPI_ISL_5673888	2021-07-05	RHD XV	Gamma
EPI_ISL_5673920	2021-07-05	RHD XV	Gamma
EPI_ISL_5674030	2021-07-05	RHD XV	Gamma
EPI_ISL_5674086	2021-07-05	RHD XV	Gamma
EPI_ISL_5674112	2021-07-05	RHD XV	Gamma
EPI_ISL_5674177	2021-07-05	RHD XV	Gamma
EPI_ISL_5674220	2021-07-05	RHD XV	Gamma
EPI_ISL_5674400	2021-07-05	RHD XV	Gamma
EPI_ISL_5674434	2021-07-05	RHD XV	Gamma
EPI_ISL_5674515	2021-07-05	RHD XV	Gamma
EPI_ISL_5674624	2021-07-05	RHD XV	Gamma
EPI_ISL_5674630	2021-07-05	RHD XV	Gamma
EPI_ISL_5674809	2021-07-05	RHD XV	Gamma
EPI_ISL_5674869	2021-07-05	RHD XV	Gamma
EPI_ISL_5674874	2021-07-05	RHD XV	Gamma
EPI_ISL_5674983	2021-07-05	RHD XV	Gamma

EPI_ISL_5674987	2021-07-05	RHD XV	Gamma
EPI_ISL_5675204	2021-07-05	RHD XV	Gamma
EPI_ISL_5675352	2021-07-05	RHD XV	Gamma
EPI_ISL_5675359	2021-07-05	RHD XV	Gamma
EPI_ISL_5675365	2021-07-05	RHD XV	Gamma
EPI_ISL_5675370	2021-07-05	RHD XV	Gamma
EPI_ISL_5675381	2021-07-05	RHD XV	Gamma
EPI_ISL_5675394	2021-07-05	RHD XV	Gamma
EPI_ISL_5675399	2021-07-05	RHD XV	Gamma
EPI_ISL_5675408	2021-07-05	RHD XV	Gamma
EPI_ISL_5675411	2021-07-05	RHD XV	Gamma
EPI_ISL_5675415	2021-07-05	RHD XV	Gamma
EPI_ISL_5675454	2021-07-05	RHD XV	Gamma
EPI_ISL_5675464	2021-07-05	RHD XV	Gamma
EPI_ISL_5675470	2021-07-05	RHD XV	Gamma
EPI_ISL_5675487	2021-07-05	RHD XV	Gamma
EPI_ISL_5675494	2021-07-05	RHD XV	Gamma
EPI_ISL_3761681	2021-07-05	RHD XV	Gamma
EPI_ISL_3761682	2021-07-05	RHD XV	Gamma
EPI_ISL_3761683	2021-07-05	RHD XV	Gamma
EPI_ISL_3761530	2021-07-05	RHD XV	Gamma
EPI_ISL_3761684	2021-07-05	RHD XV	Gamma
EPI_ISL_3761685	2021-07-05	RHD XV	Gamma
EPI_ISL_3761686	2021-07-05	RHD XV	Gamma
EPI_ISL_3761614	2021-07-05	RHD XV	Gamma
EPI_ISL_3761687	2021-07-05	RHD XV	Gamma
EPI_ISL_8004710	2021-07-05	Southeast	Gamma
EPI_ISL_4520308	2021-07-06	North	Gamma
EPI_ISL_3434974	2021-07-06	North	Gamma
EPI_ISL_5658401	2021-07-06	North	Gamma
EPI_ISL_5193512	2021-07-06	North	Gamma
EPI_ISL_3536348	2021-07-06	Northeast	Gamma
EPI_ISL_3134705	2021-07-06	Northeast	Gamma
EPI_ISL_5673763	2021-07-06	RHD XV	Gamma
EPI_ISL_5673922	2021-07-06	RHD XV	Gamma
EPI_ISL_5674148	2021-07-06	RHD XV	Gamma
EPI_ISL_5674278	2021-07-06	RHD XV	Gamma
EPI_ISL_5675387	2021-07-06	RHD XV	Gamma
EPI_ISL_3761688	2021-07-06	RHD XV	Gamma
EPI_ISL_3761689	2021-07-06	RHD XV	Gamma
EPI_ISL_3761690	2021-07-06	RHD XV	Gamma
EPI_ISL_3761691	2021-07-06	RHD XV	Gamma
EPI_ISL_3761605	2021-07-06	RHD XV	Gamma
EPI_ISL_5195478	2021-07-06	Southeast	Gamma
EPI_ISL_12425946	2021-07-07	South	Delta
EPI_ISL_4520317	2021-07-07	North	Gamma
EPI_ISL_5658396	2021-07-07	North	Gamma
EPI_ISL_7249088	2021-07-07	North	Gamma
EPI_ISL_3761589	2021-07-07	RHD XV	Gamma
EPI_ISL_3761537	2021-07-07	RHD XV	Gamma
EPI_ISL_3761597	2021-07-07	RHD XV	Gamma
EPI_ISL_3761692	2021-07-07	RHD XV	Gamma
EPI_ISL_3761693	2021-07-07	RHD XV	Gamma
EPI_ISL_3761567	2021-07-07	RHD XV	Gamma
EPI_ISL_3761694	2021-07-07	RHD XV	Gamma
EPI_ISL_3761695	2021-07-07	RHD XV	Gamma
EPI_ISL_3761546	2021-07-07	RHD XV	Gamma
EPI_ISL_3761696	2021-07-07	RHD XV	Gamma
EPI_ISL_3761697	2021-07-07	RHD XV	Gamma
EPI_ISL_3761545	2021-07-07	RHD XV	Gamma
EPI_ISL_3761561	2021-07-07	RHD XV	Gamma
EPI_ISL_3761554	2021-07-07	RHD XV	Gamma
EPI_ISL_3761547	2021-07-07	RHD XV	Gamma

EPI_ISL_3761608	2021-07-07	RHD XV	Gamma
EPI_ISL_3761698	2021-07-07	RHD XV	Gamma
EPI_ISL_3761600	2021-07-07	RHD XV	Gamma
EPI_ISL_3761569	2021-07-07	RHD XV	Gamma
EPI_ISL_3761544	2021-07-07	RHD XV	Gamma
EPI_ISL_3761556	2021-07-07	RHD XV	Gamma
EPI_ISL_3761699	2021-07-07	RHD XV	Gamma
EPI_ISL_12059845	2021-07-07	Southeast	Gamma
EPI_ISL_12425944	2021-07-08	South	Delta
EPI_ISL_3254669	2021-07-08	Midwest	Gamma
EPI_ISL_5193508	2021-07-08	North	Gamma
EPI_ISL_3536174	2021-07-08	Northeast	Gamma
EPI_ISL_3274747	2021-07-09	Midwest	Delta
EPI_ISL_9800154	2021-07-09	Midwest	Gamma
EPI_ISL_4212839	2021-07-09	Northeast	Gamma
EPI_ISL_5195485	2021-07-09	Southeast	Gamma
EPI_ISL_9800071	2021-07-10	Midwest	Gamma
EPI_ISL_3235278	2021-07-10	South	Gamma
EPI_ISL_3435015	2021-07-10	Southeast	Gamma
EPI_ISL_5030099	2021-07-11	Southeast	Gamma
EPI_ISL_3761572	2021-07-12	RHD XV	Alpha
EPI_ISL_3761701	2021-07-12	RHD XV	B.1.1.28
EPI_ISL_3539796	2021-07-12	North	Gamma
EPI_ISL_6173117	2021-07-12	Northeast	Gamma
EPI_ISL_5675588	2021-07-12	RHD XV	Gamma
EPI_ISL_5675709	2021-07-12	RHD XV	Gamma
EPI_ISL_5675719	2021-07-12	RHD XV	Gamma
EPI_ISL_5675774	2021-07-12	RHD XV	Gamma
EPI_ISL_5675793	2021-07-12	RHD XV	Gamma
EPI_ISL_5675937	2021-07-12	RHD XV	Gamma
EPI_ISL_5675943	2021-07-12	RHD XV	Gamma
EPI_ISL_5676069	2021-07-12	RHD XV	Gamma
EPI_ISL_5676108	2021-07-12	RHD XV	Gamma
EPI_ISL_5676155	2021-07-12	RHD XV	Gamma
EPI_ISL_5676188	2021-07-12	RHD XV	Gamma
EPI_ISL_5676211	2021-07-12	RHD XV	Gamma
EPI_ISL_5676361	2021-07-12	RHD XV	Gamma
EPI_ISL_5676375	2021-07-12	RHD XV	Gamma
EPI_ISL_5676582	2021-07-12	RHD XV	Gamma
EPI_ISL_5676643	2021-07-12	RHD XV	Gamma
EPI_ISL_5676683	2021-07-12	RHD XV	Gamma
EPI_ISL_5676806	2021-07-12	RHD XV	Gamma
EPI_ISL_5676834	2021-07-12	RHD XV	Gamma
EPI_ISL_5676848	2021-07-12	RHD XV	Gamma
EPI_ISL_5676849	2021-07-12	RHD XV	Gamma
EPI_ISL_5676853	2021-07-12	RHD XV	Gamma
EPI_ISL_5676855	2021-07-12	RHD XV	Gamma
EPI_ISL_5676865	2021-07-12	RHD XV	Gamma
EPI_ISL_5676939	2021-07-12	RHD XV	Gamma
EPI_ISL_5677013	2021-07-12	RHD XV	Gamma
EPI_ISL_5677044	2021-07-12	RHD XV	Gamma
EPI_ISL_5677126	2021-07-12	RHD XV	Gamma
EPI_ISL_5677156	2021-07-12	RHD XV	Gamma
EPI_ISL_5677164	2021-07-12	RHD XV	Gamma
EPI_ISL_5677214	2021-07-12	RHD XV	Gamma
EPI_ISL_5677220	2021-07-12	RHD XV	Gamma
EPI_ISL_5677241	2021-07-12	RHD XV	Gamma
EPI_ISL_5677292	2021-07-12	RHD XV	Gamma
EPI_ISL_5677294	2021-07-12	RHD XV	Gamma
EPI_ISL_5677355	2021-07-12	RHD XV	Gamma
EPI_ISL_5677397	2021-07-12	RHD XV	Gamma
EPI_ISL_5677496	2021-07-12	RHD XV	Gamma
EPI_ISL_5677513	2021-07-12	RHD XV	Gamma

EPI_ISL_5677539	2021-07-12	RHD XV	Gamma
EPI_ISL_5677591	2021-07-12	RHD XV	Gamma
EPI_ISL_5677596	2021-07-12	RHD XV	Gamma
EPI_ISL_5677602	2021-07-12	RHD XV	Gamma
EPI_ISL_5677632	2021-07-12	RHD XV	Gamma
EPI_ISL_5677640	2021-07-12	RHD XV	Gamma
EPI_ISL_5677680	2021-07-12	RHD XV	Gamma
EPI_ISL_5677690	2021-07-12	RHD XV	Gamma
EPI_ISL_5677698	2021-07-12	RHD XV	Gamma
EPI_ISL_5677716	2021-07-12	RHD XV	Gamma
EPI_ISL_5677719	2021-07-12	RHD XV	Gamma
EPI_ISL_5677730	2021-07-12	RHD XV	Gamma
EPI_ISL_5678004	2021-07-12	RHD XV	Gamma
EPI_ISL_5678082	2021-07-12	RHD XV	Gamma
EPI_ISL_5678358	2021-07-12	RHD XV	Gamma
EPI_ISL_5678549	2021-07-12	RHD XV	Gamma
EPI_ISL_5678590	2021-07-12	RHD XV	Gamma
EPI_ISL_5678641	2021-07-12	RHD XV	Gamma
EPI_ISL_5678672	2021-07-12	RHD XV	Gamma
EPI_ISL_5678759	2021-07-12	RHD XV	Gamma
EPI_ISL_5678791	2021-07-12	RHD XV	Gamma
EPI_ISL_5678843	2021-07-12	RHD XV	Gamma
EPI_ISL_5678874	2021-07-12	RHD XV	Gamma
EPI_ISL_5678888	2021-07-12	RHD XV	Gamma
EPI_ISL_5678947	2021-07-12	RHD XV	Gamma
EPI_ISL_5679027	2021-07-12	RHD XV	Gamma
EPI_ISL_5679073	2021-07-12	RHD XV	Gamma
EPI_ISL_5679078	2021-07-12	RHD XV	Gamma
EPI_ISL_5679162	2021-07-12	RHD XV	Gamma
EPI_ISL_5679546	2021-07-12	RHD XV	Gamma
EPI_ISL_5679742	2021-07-12	RHD XV	Gamma
EPI_ISL_5679751	2021-07-12	RHD XV	Gamma
EPI_ISL_5679792	2021-07-12	RHD XV	Gamma
EPI_ISL_5679863	2021-07-12	RHD XV	Gamma
EPI_ISL_5679987	2021-07-12	RHD XV	Gamma
EPI_ISL_5680023	2021-07-12	RHD XV	Gamma
EPI_ISL_5680027	2021-07-12	RHD XV	Gamma
EPI_ISL_5680060	2021-07-12	RHD XV	Gamma
EPI_ISL_5680098	2021-07-12	RHD XV	Gamma
EPI_ISL_5680114	2021-07-12	RHD XV	Gamma
EPI_ISL_5680120	2021-07-12	RHD XV	Gamma
EPI_ISL_5680124	2021-07-12	RHD XV	Gamma
EPI_ISL_5680127	2021-07-12	RHD XV	Gamma
EPI_ISL_5680139	2021-07-12	RHD XV	Gamma
EPI_ISL_5680146	2021-07-12	RHD XV	Gamma
EPI_ISL_5680156	2021-07-12	RHD XV	Gamma
EPI_ISL_5680165	2021-07-12	RHD XV	Gamma
EPI_ISL_5680172	2021-07-12	RHD XV	Gamma
EPI_ISL_5680182	2021-07-12	RHD XV	Gamma
EPI_ISL_5680189	2021-07-12	RHD XV	Gamma
EPI_ISL_5680195	2021-07-12	RHD XV	Gamma
EPI_ISL_5680202	2021-07-12	RHD XV	Gamma
EPI_ISL_5680214	2021-07-12	RHD XV	Gamma
EPI_ISL_5680218	2021-07-12	RHD XV	Gamma
EPI_ISL_5680223	2021-07-12	RHD XV	Gamma
EPI_ISL_3761565	2021-07-12	RHD XV	Gamma
EPI_ISL_3761700	2021-07-12	RHD XV	Gamma
EPI_ISL_3761609	2021-07-12	RHD XV	Gamma
EPI_ISL_3761702	2021-07-12	RHD XV	Gamma
EPI_ISL_3761764	2021-07-12	RHD XV	Gamma
EPI_ISL_3761703	2021-07-12	RHD XV	Gamma
EPI_ISL_3761704	2021-07-12	RHD XV	Gamma
EPI_ISL_3761705	2021-07-12	RHD XV	Gamma

Supplementary Table 2

EPI_ISL_3761706	2021-07-12	RHD XV	Gamma
EPI_ISL_3761707	2021-07-12	RHD XV	Gamma
EPI_ISL_3761708	2021-07-12	RHD XV	Gamma
EPI_ISL_3761709	2021-07-12	RHD XV	Gamma
EPI_ISL_3245453	2021-07-12	Southeast	Gamma
EPI_ISL_5529917	2021-07-13	South	Delta
EPI_ISL_3434943	2021-07-13	Northeast	Gamma
EPI_ISL_5679647	2021-07-13	RHD XV	Gamma
EPI_ISL_3761555	2021-07-13	RHD XV	Gamma
EPI_ISL_3761531	2021-07-13	RHD XV	Gamma
EPI_ISL_3761536	2021-07-13	RHD XV	Gamma
EPI_ISL_3761710	2021-07-13	RHD XV	Gamma
EPI_ISL_3761551	2021-07-13	RHD XV	Gamma
EPI_ISL_3761519	2021-07-13	RHD XV	Gamma
EPI_ISL_3761524	2021-07-13	RHD XV	Gamma
EPI_ISL_3761568	2021-07-13	RHD XV	Gamma
EPI_ISL_3761526	2021-07-13	RHD XV	Gamma
EPI_ISL_3761711	2021-07-13	RHD XV	Gamma
EPI_ISL_3761712	2021-07-13	RHD XV	Gamma
EPI_ISL_3761713	2021-07-13	RHD XV	Gamma
EPI_ISL_3761714	2021-07-14	RHD XV	Gamma
EPI_ISL_3761715	2021-07-14	RHD XV	Gamma
EPI_ISL_3761716	2021-07-14	RHD XV	Gamma
EPI_ISL_3761610	2021-07-14	RHD XV	Gamma
EPI_ISL_3761717	2021-07-14	RHD XV	Gamma
EPI_ISL_3761601	2021-07-14	RHD XV	Gamma
EPI_ISL_3761718	2021-07-14	RHD XV	Gamma
EPI_ISL_3761719	2021-07-14	RHD XV	Gamma
EPI_ISL_3761602	2021-07-14	RHD XV	Gamma
EPI_ISL_3761532	2021-07-14	RHD XV	Gamma
EPI_ISL_3761720	2021-07-14	RHD XV	Gamma
EPI_ISL_3761615	2021-07-14	RHD XV	Gamma
EPI_ISL_3761721	2021-07-14	RHD XV	Gamma
EPI_ISL_3761564	2021-07-14	RHD XV	Gamma
EPI_ISL_3761548	2021-07-14	RHD XV	Gamma
EPI_ISL_3761722	2021-07-14	RHD XV	Gamma
EPI_ISL_3761590	2021-07-14	RHD XV	Gamma
EPI_ISL_3761723	2021-07-14	RHD XV	Gamma
EPI_ISL_12425966	2021-07-14	South	Gamma
EPI_ISL_5195490	2021-07-14	Southeast	Gamma
EPI_ISL_4081136	2021-07-14	Southeast	Gamma
EPI_ISL_4520349	2021-07-15	North	Gamma
EPI_ISL_3761724	2021-07-15	RHD XV	Gamma
EPI_ISL_3761725	2021-07-15	RHD XV	Gamma
EPI_ISL_3761726	2021-07-15	RHD XV	Gamma
EPI_ISL_3761603	2021-07-15	RHD XV	Gamma
EPI_ISL_3761585	2021-07-15	RHD XV	Gamma
EPI_ISL_3761727	2021-07-15	RHD XV	Gamma
EPI_ISL_3761728	2021-07-15	RHD XV	Gamma
EPI_ISL_3761729	2021-07-15	RHD XV	Gamma
EPI_ISL_3761730	2021-07-15	RHD XV	Gamma
EPI_ISL_3761731	2021-07-15	RHD XV	Gamma
EPI_ISL_3761732	2021-07-15	RHD XV	Gamma
EPI_ISL_3761733	2021-07-15	RHD XV	Gamma
EPI_ISL_3761518	2021-07-15	RHD XV	Gamma
EPI_ISL_3703704	2021-07-16	Northeast	Delta
EPI_ISL_16085136	2021-07-16	Southeast	Delta
EPI_ISL_3259358	2021-07-16	Midwest	Gamma
EPI_ISL_3536368	2021-07-16	Northeast	Gamma
EPI_ISL_3540020	2021-07-17	South	Delta
EPI_ISL_3539814	2021-07-17	North	Gamma
EPI_ISL_5652021	2021-07-17	RHD XV	Gamma
EPI_ISL_3802930	2021-07-18	Northeast	Gamma

EPI_ISL_4081143	2021-07-18	Southeast	Gamma
EPI_ISL_9800174	2021-07-19	Midwest	Gamma
EPI_ISL_5926890	2021-07-19	North	Gamma
EPI_ISL_6721832	2021-07-19	North	Gamma
EPI_ISL_5653794	2021-07-19	RHD XV	Gamma
EPI_ISL_5651828	2021-07-19	RHD XV	Gamma
EPI_ISL_5651841	2021-07-19	RHD XV	Gamma
EPI_ISL_5651847	2021-07-19	RHD XV	Gamma
EPI_ISL_5651855	2021-07-19	RHD XV	Gamma
EPI_ISL_5651866	2021-07-19	RHD XV	Gamma
EPI_ISL_5651917	2021-07-19	RHD XV	Gamma
EPI_ISL_5650469	2021-07-19	RHD XV	Gamma
EPI_ISL_5650433	2021-07-19	RHD XV	Gamma
EPI_ISL_5651920	2021-07-19	RHD XV	Gamma
EPI_ISL_5650652	2021-07-19	RHD XV	Gamma
EPI_ISL_5651921	2021-07-19	RHD XV	Gamma
EPI_ISL_5651922	2021-07-19	RHD XV	Gamma
EPI_ISL_5651930	2021-07-19	RHD XV	Gamma
EPI_ISL_5651931	2021-07-19	RHD XV	Gamma
EPI_ISL_5651932	2021-07-19	RHD XV	Gamma
EPI_ISL_5651936	2021-07-19	RHD XV	Gamma
EPI_ISL_5651937	2021-07-19	RHD XV	Gamma
EPI_ISL_5651938	2021-07-19	RHD XV	Gamma
EPI_ISL_5651941	2021-07-19	RHD XV	Gamma
EPI_ISL_5651948	2021-07-19	RHD XV	Gamma
EPI_ISL_5651949	2021-07-19	RHD XV	Gamma
EPI_ISL_5651950	2021-07-19	RHD XV	Gamma
EPI_ISL_5651952	2021-07-19	RHD XV	Gamma
EPI_ISL_5651955	2021-07-19	RHD XV	Gamma
EPI_ISL_5651958	2021-07-19	RHD XV	Gamma
EPI_ISL_5651961	2021-07-19	RHD XV	Gamma
EPI_ISL_5650430	2021-07-19	RHD XV	Gamma
EPI_ISL_5651966	2021-07-19	RHD XV	Gamma
EPI_ISL_5651968	2021-07-19	RHD XV	Gamma
EPI_ISL_5651969	2021-07-19	RHD XV	Gamma
EPI_ISL_5651970	2021-07-19	RHD XV	Gamma
EPI_ISL_5651971	2021-07-19	RHD XV	Gamma
EPI_ISL_5651972	2021-07-19	RHD XV	Gamma
EPI_ISL_5650500	2021-07-19	RHD XV	Gamma
EPI_ISL_5651974	2021-07-19	RHD XV	Gamma
EPI_ISL_5651975	2021-07-19	RHD XV	Gamma
EPI_ISL_5651976	2021-07-19	RHD XV	Gamma
EPI_ISL_5651978	2021-07-19	RHD XV	Gamma
EPI_ISL_5651980	2021-07-19	RHD XV	Gamma
EPI_ISL_5651982	2021-07-19	RHD XV	Gamma
EPI_ISL_5651983	2021-07-19	RHD XV	Gamma
EPI_ISL_5651985	2021-07-19	RHD XV	Gamma
EPI_ISL_5651986	2021-07-19	RHD XV	Gamma
EPI_ISL_5655103	2021-07-19	RHD XV	Gamma
EPI_ISL_5655042	2021-07-19	RHD XV	Gamma
EPI_ISL_5654099	2021-07-19	RHD XV	Gamma
EPI_ISL_5652012	2021-07-19	RHD XV	Gamma
EPI_ISL_5652016	2021-07-19	RHD XV	Gamma
EPI_ISL_5652017	2021-07-19	RHD XV	Gamma
EPI_ISL_5652019	2021-07-19	RHD XV	Gamma
EPI_ISL_5653868	2021-07-19	RHD XV	Gamma
EPI_ISL_5652020	2021-07-19	RHD XV	Gamma
EPI_ISL_5652022	2021-07-19	RHD XV	Gamma
EPI_ISL_5650451	2021-07-19	RHD XV	Gamma
EPI_ISL_5650597	2021-07-19	RHD XV	Gamma
EPI_ISL_5650598	2021-07-19	RHD XV	Gamma
EPI_ISL_5652025	2021-07-19	RHD XV	Gamma
EPI_ISL_5652026	2021-07-19	RHD XV	Gamma

EPI_ISL_5650599	2021-07-19	RHD XV	Gamma
EPI_ISL_5652028	2021-07-19	RHD XV	Gamma
EPI_ISL_5652029	2021-07-19	RHD XV	Gamma
EPI_ISL_5652030	2021-07-19	RHD XV	Gamma
EPI_ISL_5652031	2021-07-19	RHD XV	Gamma
EPI_ISL_5652033	2021-07-19	RHD XV	Gamma
EPI_ISL_5652034	2021-07-19	RHD XV	Gamma
EPI_ISL_5652035	2021-07-19	RHD XV	Gamma
EPI_ISL_5652038	2021-07-19	RHD XV	Gamma
EPI_ISL_5650600	2021-07-19	RHD XV	Gamma
EPI_ISL_5650452	2021-07-19	RHD XV	Gamma
EPI_ISL_5650653	2021-07-19	RHD XV	Gamma
EPI_ISL_5650453	2021-07-19	RHD XV	Gamma
EPI_ISL_5650650	2021-07-19	RHD XV	Gamma
EPI_ISL_5652047	2021-07-19	RHD XV	Gamma
EPI_ISL_5652048	2021-07-19	RHD XV	Gamma
EPI_ISL_5652050	2021-07-19	RHD XV	Gamma
EPI_ISL_5652051	2021-07-19	RHD XV	Gamma
EPI_ISL_5652057	2021-07-19	RHD XV	Gamma
EPI_ISL_5654212	2021-07-19	RHD XV	Gamma
EPI_ISL_5652060	2021-07-19	RHD XV	Gamma
EPI_ISL_5652061	2021-07-19	RHD XV	Gamma
EPI_ISL_5652063	2021-07-19	RHD XV	Gamma
EPI_ISL_5652064	2021-07-19	RHD XV	Gamma
EPI_ISL_5652065	2021-07-19	RHD XV	Gamma
EPI_ISL_5652066	2021-07-19	RHD XV	Gamma
EPI_ISL_5652067	2021-07-19	RHD XV	Gamma
EPI_ISL_5652069	2021-07-19	RHD XV	Gamma
EPI_ISL_5652070	2021-07-19	RHD XV	Gamma
EPI_ISL_5652071	2021-07-19	RHD XV	Gamma
EPI_ISL_5652072	2021-07-19	RHD XV	Gamma
EPI_ISL_5652073	2021-07-19	RHD XV	Gamma
EPI_ISL_5652074	2021-07-19	RHD XV	Gamma
EPI_ISL_3761521	2021-07-19	RHD XV	Gamma
EPI_ISL_3761540	2021-07-19	RHD XV	Gamma
EPI_ISL_3761734	2021-07-19	RHD XV	Gamma
EPI_ISL_3761611	2021-07-19	RHD XV	Gamma
EPI_ISL_3761735	2021-07-19	RHD XV	Gamma
EPI_ISL_3761736	2021-07-19	RHD XV	Gamma
EPI_ISL_3761598	2021-07-19	RHD XV	Gamma
EPI_ISL_3761762	2021-07-19	RHD XV	Gamma
EPI_ISL_3761737	2021-07-19	RHD XV	Gamma
EPI_ISL_3761738	2021-07-19	RHD XV	Gamma
EPI_ISL_3761739	2021-07-19	RHD XV	Gamma
EPI_ISL_3761740	2021-07-19	RHD XV	Gamma
EPI_ISL_3761741	2021-07-19	RHD XV	Gamma
EPI_ISL_3761742	2021-07-19	RHD XV	Gamma
EPI_ISL_3539915	2021-07-20	South	Delta
EPI_ISL_3801884	2021-07-20	Southeast	Delta
EPI_ISL_5193492	2021-07-20	North	Gamma
EPI_ISL_5650668	2021-07-20	RHD XV	Gamma
EPI_ISL_5651924	2021-07-20	RHD XV	Gamma
EPI_ISL_5651925	2021-07-20	RHD XV	Gamma
EPI_ISL_5651929	2021-07-20	RHD XV	Gamma
EPI_ISL_5650523	2021-07-20	RHD XV	Gamma
EPI_ISL_5651935	2021-07-20	RHD XV	Gamma
EPI_ISL_5651939	2021-07-20	RHD XV	Gamma
EPI_ISL_5650427	2021-07-20	RHD XV	Gamma
EPI_ISL_5651945	2021-07-20	RHD XV	Gamma
EPI_ISL_5651954	2021-07-20	RHD XV	Gamma
EPI_ISL_5651956	2021-07-20	RHD XV	Gamma
EPI_ISL_5651963	2021-07-20	RHD XV	Gamma
EPI_ISL_5652056	2021-07-20	RHD XV	Gamma

EPI_ISL_3761577	2021-07-20	RHD XV	Gamma
EPI_ISL_3761571	2021-07-20	RHD XV	Gamma
EPI_ISL_3761743	2021-07-20	RHD XV	Gamma
EPI_ISL_3761744	2021-07-20	RHD XV	Gamma
EPI_ISL_3761552	2021-07-20	RHD XV	Gamma
EPI_ISL_3761562	2021-07-20	RHD XV	Gamma
EPI_ISL_3761745	2021-07-20	RHD XV	Gamma
EPI_ISL_3761612	2021-07-20	RHD XV	Gamma
EPI_ISL_3761543	2021-07-20	RHD XV	Gamma
EPI_ISL_3761560	2021-07-20	RHD XV	Gamma
EPI_ISL_3761578	2021-07-20	RHD XV	Gamma
EPI_ISL_3761557	2021-07-20	RHD XV	Gamma
EPI_ISL_3761746	2021-07-20	RHD XV	Gamma
EPI_ISL_3761542	2021-07-20	RHD XV	Gamma
EPI_ISL_3761575	2021-07-20	RHD XV	Gamma
EPI_ISL_3761747	2021-07-20	RHD XV	Gamma
EPI_ISL_3761553	2021-07-20	RHD XV	Gamma
EPI_ISL_3761748	2021-07-20	RHD XV	Gamma
EPI_ISL_5488685	2021-07-21	North	Gamma
EPI_ISL_3539749	2021-07-22	North	Gamma
EPI_ISL_4212838	2021-07-22	Northeast	Gamma
EPI_ISL_12425983	2021-07-22	South	Gamma
EPI_ISL_8004764	2021-07-22	Southeast	Gamma
EPI_ISL_5926887	2021-07-23	North	Gamma
EPI_ISL_12425992	2021-07-23	South	Others
EPI_ISL_3539238	2021-07-24	North	Gamma
EPI_ISL_3539239	2021-07-25	North	Gamma
EPI_ISL_5652092	2021-07-25	RHD XV	Gamma
EPI_ISL_5652098	2021-07-25	RHD XV	Gamma
EPI_ISL_5652103	2021-07-25	RHD XV	Gamma
EPI_ISL_5652105	2021-07-25	RHD XV	Gamma
EPI_ISL_5652139	2021-07-25	RHD XV	Gamma
EPI_ISL_5652168	2021-07-25	RHD XV	Gamma
EPI_ISL_3827996	2021-07-25	Southeast	Gamma
EPI_ISL_5195467	2021-07-26	Southeast	Delta
EPI_ISL_5653764	2021-07-26	RHD XV	Gamma
EPI_ISL_5654855	2021-07-26	RHD XV	Gamma
EPI_ISL_5654727	2021-07-26	RHD XV	Gamma
EPI_ISL_5654439	2021-07-26	RHD XV	Gamma
EPI_ISL_5652086	2021-07-26	RHD XV	Gamma
EPI_ISL_5652090	2021-07-26	RHD XV	Gamma
EPI_ISL_5650601	2021-07-26	RHD XV	Gamma
EPI_ISL_5652095	2021-07-26	RHD XV	Gamma
EPI_ISL_5650556	2021-07-26	RHD XV	Gamma
EPI_ISL_5653869	2021-07-26	RHD XV	Gamma
EPI_ISL_5652129	2021-07-26	RHD XV	Gamma
EPI_ISL_5650635	2021-07-26	RHD XV	Gamma
EPI_ISL_5652142	2021-07-26	RHD XV	Gamma
EPI_ISL_5652150	2021-07-26	RHD XV	Gamma
EPI_ISL_5652158	2021-07-26	RHD XV	Gamma
EPI_ISL_5652163	2021-07-26	RHD XV	Gamma
EPI_ISL_5652167	2021-07-26	RHD XV	Gamma
EPI_ISL_5652170	2021-07-26	RHD XV	Gamma
EPI_ISL_5652171	2021-07-26	RHD XV	Gamma
EPI_ISL_5652177	2021-07-26	RHD XV	Gamma
EPI_ISL_5652179	2021-07-26	RHD XV	Gamma
EPI_ISL_5652185	2021-07-26	RHD XV	Gamma
EPI_ISL_5652194	2021-07-26	RHD XV	Gamma
EPI_ISL_5652217	2021-07-26	RHD XV	Gamma
EPI_ISL_5652219	2021-07-26	RHD XV	Gamma
EPI_ISL_5652220	2021-07-26	RHD XV	Gamma
EPI_ISL_5652224	2021-07-26	RHD XV	Gamma
EPI_ISL_5652247	2021-07-26	RHD XV	Gamma

EPI_ISL_5652255	2021-07-26	RHD XV	Gamma
EPI_ISL_5652258	2021-07-26	RHD XV	Gamma
EPI_ISL_5652269	2021-07-26	RHD XV	Gamma
EPI_ISL_5652272	2021-07-26	RHD XV	Gamma
EPI_ISL_5655121	2021-07-26	RHD XV	Gamma
EPI_ISL_5655111	2021-07-26	RHD XV	Gamma
EPI_ISL_5652284	2021-07-26	RHD XV	Gamma
EPI_ISL_5652286	2021-07-26	RHD XV	Gamma
EPI_ISL_5652307	2021-07-26	RHD XV	Gamma
EPI_ISL_5652310	2021-07-26	RHD XV	Gamma
EPI_ISL_5652311	2021-07-26	RHD XV	Gamma
EPI_ISL_5652312	2021-07-26	RHD XV	Gamma
EPI_ISL_5652313	2021-07-26	RHD XV	Gamma
EPI_ISL_5652314	2021-07-26	RHD XV	Gamma
EPI_ISL_5653801	2021-07-26	RHD XV	Gamma
EPI_ISL_5655087	2021-07-26	RHD XV	Gamma
EPI_ISL_5655071	2021-07-26	RHD XV	Gamma
EPI_ISL_5654863	2021-07-26	RHD XV	Gamma
EPI_ISL_5652319	2021-07-26	RHD XV	Gamma
EPI_ISL_5652320	2021-07-26	RHD XV	Gamma
EPI_ISL_5652330	2021-07-26	RHD XV	Gamma
EPI_ISL_5650637	2021-07-26	RHD XV	Gamma
EPI_ISL_5652343	2021-07-26	RHD XV	Gamma
EPI_ISL_5650454	2021-07-26	RHD XV	Gamma
EPI_ISL_5650605	2021-07-26	RHD XV	Gamma
EPI_ISL_5652368	2021-07-26	RHD XV	Gamma
EPI_ISL_5652375	2021-07-26	RHD XV	Gamma
EPI_ISL_5652376	2021-07-26	RHD XV	Gamma
EPI_ISL_5652377	2021-07-26	RHD XV	Gamma
EPI_ISL_5652381	2021-07-26	RHD XV	Gamma
EPI_ISL_5652398	2021-07-26	RHD XV	Gamma
EPI_ISL_5650455	2021-07-26	RHD XV	Gamma
EPI_ISL_5650606	2021-07-26	RHD XV	Gamma
EPI_ISL_5652457	2021-07-26	RHD XV	Gamma
EPI_ISL_5652486	2021-07-26	RHD XV	Gamma
EPI_ISL_5652487	2021-07-26	RHD XV	Gamma
EPI_ISL_5652488	2021-07-26	RHD XV	Gamma
EPI_ISL_5652492	2021-07-26	RHD XV	Gamma
EPI_ISL_5652499	2021-07-26	RHD XV	Gamma
EPI_ISL_5652504	2021-07-26	RHD XV	Gamma
EPI_ISL_5653871	2021-07-26	RHD XV	Gamma
EPI_ISL_5652651	2021-07-26	RHD XV	Gamma
EPI_ISL_5491028	2021-07-27	North	Delta
EPI_ISL_8005440	2021-07-27	South	Delta
EPI_ISL_5926885	2021-07-27	North	Gamma
EPI_ISL_5658257	2021-07-27	North	Gamma
EPI_ISL_3912201	2021-07-27	Northeast	Gamma
EPI_ISL_3703726	2021-07-27	Northeast	Gamma
EPI_ISL_5654905	2021-07-27	RHD XV	Gamma
EPI_ISL_5652181	2021-07-27	RHD XV	Gamma
EPI_ISL_5652209	2021-07-27	RHD XV	Gamma
EPI_ISL_5652218	2021-07-27	RHD XV	Gamma
EPI_ISL_5652263	2021-07-27	RHD XV	Gamma
EPI_ISL_5655101	2021-07-27	RHD XV	Gamma
EPI_ISL_5652282	2021-07-27	RHD XV	Gamma
EPI_ISL_5652296	2021-07-27	RHD XV	Gamma
EPI_ISL_5652413	2021-07-27	RHD XV	Gamma
EPI_ISL_5652536	2021-07-27	RHD XV	Gamma
EPI_ISL_5652753	2021-07-27	RHD XV	Gamma
EPI_ISL_5652772	2021-07-27	RHD XV	Gamma
EPI_ISL_5490974	2021-07-28	North	Delta
EPI_ISL_4080803	2021-07-28	North	Gamma
EPI_ISL_5926897	2021-07-29	North	Gamma

EPI_ISL_4061401	2021-07-29	Northeast	Gamma
EPI_ISL_3841319	2021-07-30	Midwest	Delta
EPI_ISL_5529918	2021-07-30	South	Delta
EPI_ISL_8004799	2021-07-30	Southeast	Delta
EPI_ISL_4880287	2021-07-30	Northeast	Gamma
EPI_ISL_13131314	2021-07-31	Midwest	Delta
EPI_ISL_13131315	2021-07-31	Midwest	Delta
EPI_ISL_4212824	2021-07-31	Northeast	Delta
EPI_ISL_8004793	2021-07-31	Southeast	Delta
EPI_ISL_3536478	2021-07-31	Midwest	Gamma
EPI_ISL_9800263	2021-07-31	Midwest	Gamma
EPI_ISL_4081047	2021-07-31	North	Gamma
EPI_ISL_4080802	2021-07-31	North	Gamma
EPI_ISL_6908699	2021-08-01	Northeast	B.1.1.28
EPI_ISL_4220167	2021-08-01	Southeast	Delta
EPI_ISL_5653215	2021-08-01	RHD XV	Gamma
EPI_ISL_5653219	2021-08-01	RHD XV	Gamma
EPI_ISL_5653228	2021-08-01	RHD XV	Gamma
EPI_ISL_5653345	2021-08-01	RHD XV	Gamma
EPI_ISL_5653347	2021-08-01	RHD XV	Gamma
EPI_ISL_5653401	2021-08-01	RHD XV	Gamma
EPI_ISL_5653420	2021-08-01	RHD XV	Gamma
EPI_ISL_12426024	2021-08-01	South	Gamma
EPI_ISL_3912399	2021-08-02	Northeast	Delta
EPI_ISL_3579343	2021-08-02	RHD XV	Delta
EPI_ISL_4271132	2021-08-02	South	Delta
EPI_ISL_14534233	2021-08-02	Southeast	Delta
EPI_ISL_3386131	2021-08-02	Midwest	Gamma
EPI_ISL_9800123	2021-08-02	Midwest	Gamma
EPI_ISL_4061291	2021-08-02	North	Gamma
EPI_ISL_5193488	2021-08-02	North	Gamma
EPI_ISL_4061420	2021-08-02	Northeast	Gamma
EPI_ISL_3835251	2021-08-02	Northeast	Gamma
EPI_ISL_5653633	2021-08-02	RHD XV	Gamma
EPI_ISL_5654733	2021-08-02	RHD XV	Gamma
EPI_ISL_5654446	2021-08-02	RHD XV	Gamma
EPI_ISL_5654107	2021-08-02	RHD XV	Gamma
EPI_ISL_5654047	2021-08-02	RHD XV	Gamma
EPI_ISL_5654124	2021-08-02	RHD XV	Gamma
EPI_ISL_5654009	2021-08-02	RHD XV	Gamma
EPI_ISL_5654055	2021-08-02	RHD XV	Gamma
EPI_ISL_5654022	2021-08-02	RHD XV	Gamma
EPI_ISL_5653207	2021-08-02	RHD XV	Gamma
EPI_ISL_5650457	2021-08-02	RHD XV	Gamma
EPI_ISL_5650458	2021-08-02	RHD XV	Gamma
EPI_ISL_5650459	2021-08-02	RHD XV	Gamma
EPI_ISL_5650587	2021-08-02	RHD XV	Gamma
EPI_ISL_5653244	2021-08-02	RHD XV	Gamma
EPI_ISL_5653245	2021-08-02	RHD XV	Gamma
EPI_ISL_5653254	2021-08-02	RHD XV	Gamma
EPI_ISL_5653266	2021-08-02	RHD XV	Gamma
EPI_ISL_5650503	2021-08-02	RHD XV	Gamma
EPI_ISL_5653272	2021-08-02	RHD XV	Gamma
EPI_ISL_5650448	2021-08-02	RHD XV	Gamma
EPI_ISL_5653280	2021-08-02	RHD XV	Gamma
EPI_ISL_5653281	2021-08-02	RHD XV	Gamma
EPI_ISL_5653284	2021-08-02	RHD XV	Gamma
EPI_ISL_5653286	2021-08-02	RHD XV	Gamma
EPI_ISL_5653292	2021-08-02	RHD XV	Gamma
EPI_ISL_5653317	2021-08-02	RHD XV	Gamma
EPI_ISL_5653318	2021-08-02	RHD XV	Gamma
EPI_ISL_5650460	2021-08-02	RHD XV	Gamma
EPI_ISL_5653332	2021-08-02	RHD XV	Gamma

EPI_ISL_5653334	2021-08-02	RHD XV	Gamma
EPI_ISL_5653348	2021-08-02	RHD XV	Gamma
EPI_ISL_5650461	2021-08-02	RHD XV	Gamma
EPI_ISL_5653354	2021-08-02	RHD XV	Gamma
EPI_ISL_5650612	2021-08-02	RHD XV	Gamma
EPI_ISL_5653358	2021-08-02	RHD XV	Gamma
EPI_ISL_5653360	2021-08-02	RHD XV	Gamma
EPI_ISL_5653362	2021-08-02	RHD XV	Gamma
EPI_ISL_5650462	2021-08-02	RHD XV	Gamma
EPI_ISL_5653367	2021-08-02	RHD XV	Gamma
EPI_ISL_5653377	2021-08-02	RHD XV	Gamma
EPI_ISL_5650613	2021-08-02	RHD XV	Gamma
EPI_ISL_5650588	2021-08-02	RHD XV	Gamma
EPI_ISL_5653380	2021-08-02	RHD XV	Gamma
EPI_ISL_5653385	2021-08-02	RHD XV	Gamma
EPI_ISL_5650614	2021-08-02	RHD XV	Gamma
EPI_ISL_5653408	2021-08-02	RHD XV	Gamma
EPI_ISL_5653410	2021-08-02	RHD XV	Gamma
EPI_ISL_5653416	2021-08-02	RHD XV	Gamma
EPI_ISL_5653417	2021-08-02	RHD XV	Gamma
EPI_ISL_5653418	2021-08-02	RHD XV	Gamma
EPI_ISL_5653422	2021-08-02	RHD XV	Gamma
EPI_ISL_5653818	2021-08-02	RHD XV	Gamma
EPI_ISL_5655036	2021-08-02	RHD XV	Gamma
EPI_ISL_5654706	2021-08-02	RHD XV	Gamma
EPI_ISL_5654238	2021-08-02	RHD XV	Gamma
EPI_ISL_5654037	2021-08-02	RHD XV	Gamma
EPI_ISL_5653433	2021-08-02	RHD XV	Gamma
EPI_ISL_5653435	2021-08-02	RHD XV	Gamma
EPI_ISL_5653441	2021-08-02	RHD XV	Gamma
EPI_ISL_5653444	2021-08-02	RHD XV	Gamma
EPI_ISL_5653448	2021-08-02	RHD XV	Gamma
EPI_ISL_5653453	2021-08-02	RHD XV	Gamma
EPI_ISL_5653458	2021-08-02	RHD XV	Gamma
EPI_ISL_5653461	2021-08-02	RHD XV	Gamma
EPI_ISL_5653465	2021-08-02	RHD XV	Gamma
EPI_ISL_5653466	2021-08-02	RHD XV	Gamma
EPI_ISL_5653467	2021-08-02	RHD XV	Gamma
EPI_ISL_5653469	2021-08-02	RHD XV	Gamma
EPI_ISL_5653473	2021-08-02	RHD XV	Gamma
EPI_ISL_3579312	2021-08-02	RHD XV	Gamma
EPI_ISL_12426033	2021-08-02	South	Gamma
EPI_ISL_4220171	2021-08-02	Southeast	Gamma
EPI_ISL_13131317	2021-08-03	Midwest	Delta
EPI_ISL_5658201	2021-08-03	North	Delta
EPI_ISL_3536441	2021-08-03	Midwest	Gamma
EPI_ISL_5487823	2021-08-03	North	Gamma
EPI_ISL_7359128	2021-08-03	North	Gamma
EPI_ISL_5654747	2021-08-03	RHD XV	Gamma
EPI_ISL_5654011	2021-08-03	RHD XV	Gamma
EPI_ISL_5653223	2021-08-03	RHD XV	Gamma
EPI_ISL_5653255	2021-08-03	RHD XV	Gamma
EPI_ISL_5653271	2021-08-03	RHD XV	Gamma
EPI_ISL_5653278	2021-08-03	RHD XV	Gamma
EPI_ISL_5653316	2021-08-03	RHD XV	Gamma
EPI_ISL_5653323	2021-08-03	RHD XV	Gamma
EPI_ISL_5653389	2021-08-03	RHD XV	Gamma
EPI_ISL_5653474	2021-08-03	RHD XV	Gamma
EPI_ISL_12426042	2021-08-03	South	Gamma
EPI_ISL_12426049	2021-08-04	South	Alpha
EPI_ISL_14674983	2021-08-04	Midwest	Gamma
EPI_ISL_4081042	2021-08-04	North	Gamma
EPI_ISL_6229759	2021-08-04	Northeast	Gamma

Supplementary Table 2

EPI_ISL_5529920	2021-08-05	South	Delta
EPI_ISL_14534256	2021-08-05	Southeast	Delta
EPI_ISL_3835358	2021-08-05	Southeast	Delta
EPI_ISL_3536479	2021-08-05	Midwest	Gamma
EPI_ISL_5658171	2021-08-05	North	Gamma
EPI_ISL_3835286	2021-08-05	Northeast	Gamma
EPI_ISL_4212835	2021-08-05	Northeast	Gamma
EPI_ISL_4271133	2021-08-06	South	Delta
EPI_ISL_9800102	2021-08-06	Midwest	Gamma
EPI_ISL_5658170	2021-08-06	North	Gamma
EPI_ISL_5193479	2021-08-06	North	Gamma
EPI_ISL_11681288	2021-08-06	Northeast	Gamma
EPI_ISL_4220193	2021-08-07	Southeast	Delta
EPI_ISL_8004826	2021-08-08	Southeast	Delta
EPI_ISL_6173108	2021-08-08	Northeast	Gamma
EPI_ISL_11940593	2021-08-08	RHD XV	Gamma
EPI_ISL_6100864	2021-08-09	North	Delta
EPI_ISL_4271136	2021-08-09	South	Delta
EPI_ISL_4081030	2021-08-09	North	Gamma
EPI_ISL_6229756	2021-08-09	Northeast	Gamma
EPI_ISL_11940594	2021-08-09	RHD XV	Gamma
EPI_ISL_11940595	2021-08-09	RHD XV	Gamma
EPI_ISL_11940596	2021-08-09	RHD XV	Gamma
EPI_ISL_11940597	2021-08-09	RHD XV	Gamma
EPI_ISL_11940598	2021-08-09	RHD XV	Gamma
EPI_ISL_11940599	2021-08-09	RHD XV	Gamma
EPI_ISL_11940600	2021-08-09	RHD XV	Gamma
EPI_ISL_11940601	2021-08-09	RHD XV	Gamma
EPI_ISL_11940602	2021-08-09	RHD XV	Gamma
EPI_ISL_11940603	2021-08-09	RHD XV	Gamma
EPI_ISL_11940604	2021-08-09	RHD XV	Gamma
EPI_ISL_11940605	2021-08-09	RHD XV	Gamma
EPI_ISL_11940606	2021-08-09	RHD XV	Gamma
EPI_ISL_11940607	2021-08-09	RHD XV	Gamma
EPI_ISL_11940608	2021-08-09	RHD XV	Gamma
EPI_ISL_11940614	2021-08-09	RHD XV	Gamma
EPI_ISL_11940615	2021-08-09	RHD XV	Gamma
EPI_ISL_11940616	2021-08-09	RHD XV	Gamma
EPI_ISL_11940617	2021-08-09	RHD XV	Gamma
EPI_ISL_11940619	2021-08-09	RHD XV	Gamma
EPI_ISL_11940620	2021-08-09	RHD XV	Gamma
EPI_ISL_11940621	2021-08-09	RHD XV	Gamma
EPI_ISL_11940622	2021-08-09	RHD XV	Gamma
EPI_ISL_11940623	2021-08-09	RHD XV	Gamma
EPI_ISL_11940624	2021-08-09	RHD XV	Gamma
EPI_ISL_11940625	2021-08-09	RHD XV	Gamma
EPI_ISL_11940626	2021-08-09	RHD XV	Gamma
EPI_ISL_3761591	2021-08-09	RHD XV	Gamma
EPI_ISL_3761549	2021-08-09	RHD XV	Gamma
EPI_ISL_3761550	2021-08-09	RHD XV	Gamma
EPI_ISL_3761592	2021-08-09	RHD XV	Gamma
EPI_ISL_3761593	2021-08-09	RHD XV	Gamma
EPI_ISL_3761594	2021-08-09	RHD XV	Gamma
EPI_ISL_3761595	2021-08-09	RHD XV	Gamma
EPI_ISL_4486991	2021-08-10	Northeast	Delta
EPI_ISL_3761529	2021-08-10	RHD XV	Delta
EPI_ISL_3761619	2021-08-10	RHD XV	Delta
EPI_ISL_3761621	2021-08-10	RHD XV	Delta
EPI_ISL_3758092	2021-08-10	Midwest	Gamma
EPI_ISL_4061295	2021-08-10	North	Gamma
EPI_ISL_11940592	2021-08-10	RHD XV	Gamma
EPI_ISL_11940609	2021-08-10	RHD XV	Gamma
EPI_ISL_11940610	2021-08-10	RHD XV	Gamma

EPI_ISL_11940611	2021-08-10	RHD XV	Gamma
EPI_ISL_11940612	2021-08-10	RHD XV	Gamma
EPI_ISL_11940613	2021-08-10	RHD XV	Gamma
EPI_ISL_3761618	2021-08-10	RHD XV	Gamma
EPI_ISL_3761620	2021-08-10	RHD XV	Gamma
EPI_ISL_3761559	2021-08-10	RHD XV	Gamma
EPI_ISL_3761566	2021-08-10	RHD XV	Gamma
EPI_ISL_3761534	2021-08-10	RHD XV	Gamma
EPI_ISL_3761622	2021-08-10	RHD XV	Gamma
EPI_ISL_3761623	2021-08-10	RHD XV	Gamma
EPI_ISL_3761635	2021-08-10	RHD XV	Gamma
EPI_ISL_3761765	2021-08-10	RHD XV	Gamma
EPI_ISL_3761538	2021-08-10	RHD XV	Gamma
EPI_ISL_3761527	2021-08-10	RHD XV	Gamma
EPI_ISL_3761766	2021-08-10	RHD XV	Gamma
EPI_ISL_3761541	2021-08-10	RHD XV	Gamma
EPI_ISL_3761625	2021-08-11	RHD XV	Delta
EPI_ISL_4237179	2021-08-11	South	Delta
EPI_ISL_5195507	2021-08-11	Southeast	Delta
EPI_ISL_3761533	2021-08-11	RHD XV	Gamma
EPI_ISL_3761624	2021-08-11	RHD XV	Gamma
EPI_ISL_3761626	2021-08-11	RHD XV	Gamma
EPI_ISL_3761627	2021-08-11	RHD XV	Gamma
EPI_ISL_3761628	2021-08-11	RHD XV	Gamma
EPI_ISL_3761596	2021-08-11	RHD XV	Gamma
EPI_ISL_3761629	2021-08-11	RHD XV	Gamma
EPI_ISL_3761630	2021-08-11	RHD XV	Gamma
EPI_ISL_3761574	2021-08-11	RHD XV	Gamma
EPI_ISL_3761631	2021-08-11	RHD XV	Gamma
EPI_ISL_3761632	2021-08-11	RHD XV	Gamma
EPI_ISL_3761604	2021-08-11	RHD XV	Gamma
EPI_ISL_3761633	2021-08-11	RHD XV	Gamma
EPI_ISL_3761606	2021-08-11	RHD XV	Gamma
EPI_ISL_3761634	2021-08-11	RHD XV	Gamma
EPI_ISL_11403608	2021-08-12	North	Delta
EPI_ISL_4170355	2021-08-12	South	Delta
EPI_ISL_9800105	2021-08-13	Midwest	Gamma
EPI_ISL_8004876	2021-08-13	Southeast	Gamma
EPI_ISL_5254446	2021-08-14	Northeast	Delta
EPI_ISL_8004871	2021-08-14	Southeast	Delta
EPI_ISL_4220240	2021-08-15	Southeast	Delta
EPI_ISL_3944899	2021-08-16	RHD XV	Delta
EPI_ISL_3944866	2021-08-16	RHD XV	Delta
EPI_ISL_5490846	2021-08-16	North	Gamma
EPI_ISL_4345455	2021-08-16	North	Gamma
EPI_ISL_4474541	2021-08-16	Northeast	Gamma
EPI_ISL_11940628	2021-08-16	RHD XV	Gamma
EPI_ISL_11940629	2021-08-16	RHD XV	Gamma
EPI_ISL_11940630	2021-08-16	RHD XV	Gamma
EPI_ISL_11940631	2021-08-16	RHD XV	Gamma
EPI_ISL_11940632	2021-08-16	RHD XV	Gamma
EPI_ISL_11940633	2021-08-16	RHD XV	Gamma
EPI_ISL_11940634	2021-08-16	RHD XV	Gamma
EPI_ISL_11940635	2021-08-16	RHD XV	Gamma
EPI_ISL_11940636	2021-08-16	RHD XV	Gamma
EPI_ISL_11940637	2021-08-16	RHD XV	Gamma
EPI_ISL_11940638	2021-08-16	RHD XV	Gamma
EPI_ISL_11940640	2021-08-16	RHD XV	Gamma
EPI_ISL_11940643	2021-08-16	RHD XV	Gamma
EPI_ISL_11940644	2021-08-16	RHD XV	Gamma
EPI_ISL_11940645	2021-08-16	RHD XV	Gamma
EPI_ISL_11940650	2021-08-16	RHD XV	Gamma
EPI_ISL_11940652	2021-08-16	RHD XV	Gamma

EPI_ISL_11940653	2021-08-16	RHD XV	Gamma
EPI_ISL_11940654	2021-08-16	RHD XV	Gamma
EPI_ISL_11940655	2021-08-16	RHD XV	Gamma
EPI_ISL_11940656	2021-08-16	RHD XV	Gamma
EPI_ISL_11940657	2021-08-16	RHD XV	Gamma
EPI_ISL_11940658	2021-08-16	RHD XV	Gamma
EPI_ISL_11940659	2021-08-16	RHD XV	Gamma
EPI_ISL_11940660	2021-08-16	RHD XV	Gamma
EPI_ISL_11940661	2021-08-16	RHD XV	Gamma
EPI_ISL_11940662	2021-08-16	RHD XV	Gamma
EPI_ISL_11940663	2021-08-16	RHD XV	Gamma
EPI_ISL_11940664	2021-08-16	RHD XV	Gamma
EPI_ISL_11940665	2021-08-16	RHD XV	Gamma
EPI_ISL_11940666	2021-08-16	RHD XV	Gamma
EPI_ISL_11940667	2021-08-16	RHD XV	Gamma
EPI_ISL_11940668	2021-08-16	RHD XV	Gamma
EPI_ISL_11940669	2021-08-16	RHD XV	Gamma
EPI_ISL_11940670	2021-08-16	RHD XV	Gamma
EPI_ISL_11940671	2021-08-16	RHD XV	Gamma
EPI_ISL_11940672	2021-08-16	RHD XV	Gamma
EPI_ISL_6907016	2021-08-17	Northeast	Gamma
EPI_ISL_11940627	2021-08-17	RHD XV	Gamma
EPI_ISL_11940641	2021-08-17	RHD XV	Gamma
EPI_ISL_11940642	2021-08-17	RHD XV	Gamma
EPI_ISL_11940646	2021-08-17	RHD XV	Gamma
EPI_ISL_11940647	2021-08-17	RHD XV	Gamma
EPI_ISL_11940648	2021-08-17	RHD XV	Gamma
EPI_ISL_11940649	2021-08-17	RHD XV	Gamma
EPI_ISL_11940651	2021-08-17	RHD XV	Gamma
EPI_ISL_6100871	2021-08-18	North	Delta
EPI_ISL_11940744	2021-08-18	RHD XV	Delta
EPI_ISL_5658131	2021-08-18	North	Gamma
EPI_ISL_11940694	2021-08-18	RHD XV	Gamma
EPI_ISL_11940695	2021-08-18	RHD XV	Gamma
EPI_ISL_11940696	2021-08-18	RHD XV	Gamma
EPI_ISL_11940697	2021-08-18	RHD XV	Gamma
EPI_ISL_11940698	2021-08-18	RHD XV	Gamma
EPI_ISL_11940699	2021-08-18	RHD XV	Gamma
EPI_ISL_11940700	2021-08-18	RHD XV	Gamma
EPI_ISL_11940701	2021-08-18	RHD XV	Gamma
EPI_ISL_11940702	2021-08-18	RHD XV	Gamma
EPI_ISL_11940703	2021-08-18	RHD XV	Gamma
EPI_ISL_11940704	2021-08-18	RHD XV	Gamma
EPI_ISL_11940705	2021-08-18	RHD XV	Gamma
EPI_ISL_11940706	2021-08-18	RHD XV	Gamma
EPI_ISL_11940707	2021-08-18	RHD XV	Gamma
EPI_ISL_11940708	2021-08-18	RHD XV	Gamma
EPI_ISL_11940710	2021-08-18	RHD XV	Gamma
EPI_ISL_11940711	2021-08-18	RHD XV	Gamma
EPI_ISL_11940712	2021-08-18	RHD XV	Gamma
EPI_ISL_11940713	2021-08-18	RHD XV	Gamma
EPI_ISL_11940714	2021-08-18	RHD XV	Gamma
EPI_ISL_11940727	2021-08-18	RHD XV	Gamma
EPI_ISL_11940728	2021-08-18	RHD XV	Gamma
EPI_ISL_11940729	2021-08-18	RHD XV	Gamma
EPI_ISL_11940731	2021-08-18	RHD XV	Gamma
EPI_ISL_11940732	2021-08-18	RHD XV	Gamma
EPI_ISL_11940733	2021-08-18	RHD XV	Gamma
EPI_ISL_11940734	2021-08-18	RHD XV	Gamma
EPI_ISL_11940735	2021-08-18	RHD XV	Gamma
EPI_ISL_11940736	2021-08-18	RHD XV	Gamma
EPI_ISL_11940675	2021-08-18	RHD XV	Others
EPI_ISL_4486962	2021-08-19	Northeast	Delta

EPI_ISL_11940737	2021-08-19	RHD XV	Delta
EPI_ISL_11940738	2021-08-19	RHD XV	Delta
EPI_ISL_11940739	2021-08-19	RHD XV	Delta
EPI_ISL_11940740	2021-08-19	RHD XV	Delta
EPI_ISL_11940741	2021-08-19	RHD XV	Delta
EPI_ISL_11940742	2021-08-19	RHD XV	Delta
EPI_ISL_11940743	2021-08-19	RHD XV	Delta
EPI_ISL_11940745	2021-08-19	RHD XV	Delta
EPI_ISL_11940746	2021-08-19	RHD XV	Delta
EPI_ISL_11940747	2021-08-19	RHD XV	Delta
EPI_ISL_11403541	2021-08-19	North	Gamma
EPI_ISL_11940676	2021-08-19	RHD XV	Gamma
EPI_ISL_11940677	2021-08-19	RHD XV	Gamma
EPI_ISL_11940678	2021-08-19	RHD XV	Gamma
EPI_ISL_11940679	2021-08-19	RHD XV	Gamma
EPI_ISL_11940680	2021-08-19	RHD XV	Gamma
EPI_ISL_11940681	2021-08-19	RHD XV	Gamma
EPI_ISL_11940682	2021-08-19	RHD XV	Gamma
EPI_ISL_11940683	2021-08-19	RHD XV	Gamma
EPI_ISL_11940684	2021-08-19	RHD XV	Gamma
EPI_ISL_11940685	2021-08-19	RHD XV	Gamma
EPI_ISL_11940686	2021-08-19	RHD XV	Gamma
EPI_ISL_11940687	2021-08-19	RHD XV	Gamma
EPI_ISL_11940688	2021-08-19	RHD XV	Gamma
EPI_ISL_11940689	2021-08-19	RHD XV	Gamma
EPI_ISL_11940690	2021-08-19	RHD XV	Gamma
EPI_ISL_11940691	2021-08-19	RHD XV	Gamma
EPI_ISL_11940692	2021-08-19	RHD XV	Gamma
EPI_ISL_11940693	2021-08-19	RHD XV	Gamma
EPI_ISL_11940709	2021-08-19	RHD XV	Gamma
EPI_ISL_11940715	2021-08-19	RHD XV	Gamma
EPI_ISL_11940716	2021-08-19	RHD XV	Gamma
EPI_ISL_11940717	2021-08-19	RHD XV	Gamma
EPI_ISL_11940718	2021-08-19	RHD XV	Gamma
EPI_ISL_11940719	2021-08-19	RHD XV	Gamma
EPI_ISL_11940720	2021-08-19	RHD XV	Gamma
EPI_ISL_11940721	2021-08-19	RHD XV	Gamma
EPI_ISL_11940722	2021-08-19	RHD XV	Gamma
EPI_ISL_11940723	2021-08-19	RHD XV	Gamma
EPI_ISL_11940724	2021-08-19	RHD XV	Gamma
EPI_ISL_11940725	2021-08-19	RHD XV	Gamma
EPI_ISL_11940726	2021-08-19	RHD XV	Gamma
EPI_ISL_11940730	2021-08-19	RHD XV	Gamma
EPI_ISL_11940673	2021-08-19	RHD XV	Others
EPI_ISL_11940674	2021-08-19	RHD XV	Others
EPI_ISL_7657567	2021-08-20	North	Delta
EPI_ISL_4220296	2021-08-20	Northeast	Delta
EPI_ISL_4237360	2021-08-20	South	Delta
EPI_ISL_4880252	2021-08-20	Southeast	Delta
EPI_ISL_6945663	2021-08-20	Northeast	Gamma
EPI_ISL_6908542	2021-08-21	Northeast	Delta
EPI_ISL_4212861	2021-08-21	Southeast	Delta
EPI_ISL_8004902	2021-08-21	Southeast	Delta
EPI_ISL_5193468	2021-08-21	North	Gamma
EPI_ISL_5603196	2021-08-22	Northeast	Delta
EPI_ISL_4212763	2021-08-22	Southeast	Delta
EPI_ISL_9800439	2021-08-23	Midwest	Delta
EPI_ISL_4632922	2021-08-23	North	Delta
EPI_ISL_4633834	2021-08-23	Northeast	Delta
EPI_ISL_4168740	2021-08-23	RHD XV	Delta
EPI_ISL_4168738	2021-08-23	RHD XV	Delta
EPI_ISL_4168706	2021-08-23	RHD XV	Delta
EPI_ISL_4468882	2021-08-23	RHD XV	Delta

EPI_ISL_4468880	2021-08-23	RHD XV	Delta
EPI_ISL_4468879	2021-08-23	RHD XV	Delta
EPI_ISL_4468878	2021-08-23	RHD XV	Delta
EPI_ISL_4468877	2021-08-23	RHD XV	Delta
EPI_ISL_4468881	2021-08-23	RHD XV	Delta
EPI_ISL_3922238	2021-08-23	Midwest	Gamma
EPI_ISL_6907060	2021-08-23	Northeast	Gamma
EPI_ISL_11940752	2021-08-23	RHD XV	Gamma
EPI_ISL_11940753	2021-08-23	RHD XV	Gamma
EPI_ISL_11940755	2021-08-23	RHD XV	Gamma
EPI_ISL_11940756	2021-08-23	RHD XV	Gamma
EPI_ISL_11940757	2021-08-23	RHD XV	Gamma
EPI_ISL_11940758	2021-08-23	RHD XV	Gamma
EPI_ISL_11940759	2021-08-23	RHD XV	Gamma
EPI_ISL_11940760	2021-08-23	RHD XV	Gamma
EPI_ISL_11940761	2021-08-23	RHD XV	Gamma
EPI_ISL_11940762	2021-08-23	RHD XV	Gamma
EPI_ISL_11940763	2021-08-23	RHD XV	Gamma
EPI_ISL_11940764	2021-08-23	RHD XV	Gamma
EPI_ISL_11940765	2021-08-23	RHD XV	Gamma
EPI_ISL_11940766	2021-08-23	RHD XV	Gamma
EPI_ISL_11940767	2021-08-23	RHD XV	Gamma
EPI_ISL_11940768	2021-08-23	RHD XV	Gamma
EPI_ISL_11940769	2021-08-23	RHD XV	Gamma
EPI_ISL_11940770	2021-08-23	RHD XV	Gamma
EPI_ISL_11940771	2021-08-23	RHD XV	Gamma
EPI_ISL_11940775	2021-08-23	RHD XV	Gamma
EPI_ISL_11940776	2021-08-23	RHD XV	Gamma
EPI_ISL_11940790	2021-08-23	RHD XV	Gamma
EPI_ISL_11940791	2021-08-23	RHD XV	Gamma
EPI_ISL_11940792	2021-08-23	RHD XV	Gamma
EPI_ISL_11940793	2021-08-23	RHD XV	Gamma
EPI_ISL_11940794	2021-08-23	RHD XV	Gamma
EPI_ISL_11940795	2021-08-23	RHD XV	Gamma
EPI_ISL_11940796	2021-08-23	RHD XV	Gamma
EPI_ISL_11940797	2021-08-23	RHD XV	Gamma
EPI_ISL_11940798	2021-08-23	RHD XV	Gamma
EPI_ISL_11940799	2021-08-23	RHD XV	Gamma
EPI_ISL_11940800	2021-08-23	RHD XV	Gamma
EPI_ISL_11940801	2021-08-23	RHD XV	Gamma
EPI_ISL_11940802	2021-08-23	RHD XV	Gamma
EPI_ISL_11940812	2021-08-23	RHD XV	Gamma
EPI_ISL_11940813	2021-08-23	RHD XV	Gamma
EPI_ISL_11940814	2021-08-23	RHD XV	Gamma
EPI_ISL_11940815	2021-08-23	RHD XV	Gamma
EPI_ISL_11940816	2021-08-23	RHD XV	Gamma
EPI_ISL_11940817	2021-08-23	RHD XV	Gamma
EPI_ISL_11940818	2021-08-23	RHD XV	Gamma
EPI_ISL_11940819	2021-08-23	RHD XV	Gamma
EPI_ISL_6945652	2021-08-24	Northeast	Delta
EPI_ISL_4468884	2021-08-24	RHD XV	Delta
EPI_ISL_4468883	2021-08-24	RHD XV	Delta
EPI_ISL_4633269	2021-08-24	Southeast	Delta
EPI_ISL_11940748	2021-08-24	RHD XV	Gamma
EPI_ISL_11940749	2021-08-24	RHD XV	Gamma
EPI_ISL_11940750	2021-08-24	RHD XV	Gamma
EPI_ISL_11940751	2021-08-24	RHD XV	Gamma
EPI_ISL_11940772	2021-08-24	RHD XV	Gamma
EPI_ISL_5329029	2021-08-24	South	Gamma
EPI_ISL_7132996	2021-08-24	Southeast	Gamma
EPI_ISL_7657568	2021-08-25	North	Delta
EPI_ISL_4418877	2021-08-25	Northeast	Delta
EPI_ISL_4468889	2021-08-25	RHD XV	Delta

EPI_ISL_4468887	2021-08-25	RHD XV	Delta
EPI_ISL_4468886	2021-08-25	RHD XV	Delta
EPI_ISL_4468885	2021-08-25	RHD XV	Delta
EPI_ISL_4468891	2021-08-25	RHD XV	Delta
EPI_ISL_4468890	2021-08-25	RHD XV	Delta
EPI_ISL_4468888	2021-08-25	RHD XV	Delta
EPI_ISL_7132914	2021-08-25	Southeast	Delta
EPI_ISL_4418862	2021-08-25	North	Gamma
EPI_ISL_5658094	2021-08-25	North	Gamma
EPI_ISL_5193464	2021-08-25	North	Gamma
EPI_ISL_11940773	2021-08-25	RHD XV	Gamma
EPI_ISL_11940774	2021-08-25	RHD XV	Gamma
EPI_ISL_11940777	2021-08-25	RHD XV	Gamma
EPI_ISL_11940778	2021-08-25	RHD XV	Gamma
EPI_ISL_11940779	2021-08-25	RHD XV	Gamma
EPI_ISL_11940780	2021-08-25	RHD XV	Gamma
EPI_ISL_11940781	2021-08-25	RHD XV	Gamma
EPI_ISL_11940782	2021-08-25	RHD XV	Gamma
EPI_ISL_11940783	2021-08-25	RHD XV	Gamma
EPI_ISL_11940784	2021-08-25	RHD XV	Gamma
EPI_ISL_11940785	2021-08-25	RHD XV	Gamma
EPI_ISL_11940786	2021-08-25	RHD XV	Gamma
EPI_ISL_11940787	2021-08-25	RHD XV	Gamma
EPI_ISL_11940788	2021-08-25	RHD XV	Gamma
EPI_ISL_11940789	2021-08-25	RHD XV	Gamma
EPI_ISL_11940803	2021-08-25	RHD XV	Gamma
EPI_ISL_11940804	2021-08-25	RHD XV	Gamma
EPI_ISL_11940805	2021-08-25	RHD XV	Gamma
EPI_ISL_11940806	2021-08-25	RHD XV	Gamma
EPI_ISL_11940807	2021-08-25	RHD XV	Gamma
EPI_ISL_11940808	2021-08-25	RHD XV	Gamma
EPI_ISL_11940809	2021-08-25	RHD XV	Gamma
EPI_ISL_11940810	2021-08-25	RHD XV	Gamma
EPI_ISL_11940811	2021-08-25	RHD XV	Gamma
EPI_ISL_12426127	2021-08-25	South	Gamma
EPI_ISL_11407010	2021-08-26	Midwest	Delta
EPI_ISL_4516028	2021-08-26	North	Delta
EPI_ISL_5825590	2021-08-26	Northeast	Delta
EPI_ISL_6908601	2021-08-26	Northeast	Delta
EPI_ISL_9800168	2021-08-26	Midwest	Gamma
EPI_ISL_6908622	2021-08-27	Northeast	Delta
EPI_ISL_4633830	2021-08-27	Northeast	Delta
EPI_ISL_5328913	2021-08-27	Northeast	Delta
EPI_ISL_4220331	2021-08-27	Southeast	Delta
EPI_ISL_4513324	2021-08-27	Midwest	Gamma
EPI_ISL_6172442	2021-08-28	Northeast	Delta
EPI_ISL_4728673	2021-08-28	Southeast	Delta
EPI_ISL_4516199	2021-08-28	North	Gamma
EPI_ISL_3997066	2021-08-30	Midwest	Delta
EPI_ISL_4273252	2021-08-30	RHD XV	Delta
EPI_ISL_4275644	2021-08-30	RHD XV	Delta
EPI_ISL_4275643	2021-08-30	RHD XV	Delta
EPI_ISL_4275642	2021-08-30	RHD XV	Delta
EPI_ISL_6172968	2021-08-30	South	Delta
EPI_ISL_5640179	2021-08-30	Southeast	Delta
EPI_ISL_11940830	2021-08-30	RHD XV	Gamma
EPI_ISL_11940831	2021-08-30	RHD XV	Gamma
EPI_ISL_11940832	2021-08-30	RHD XV	Gamma
EPI_ISL_11940850	2021-08-30	RHD XV	Gamma
EPI_ISL_6945647	2021-08-31	Northeast	Delta
EPI_ISL_5254347	2021-08-31	Northeast	Delta
EPI_ISL_5329051	2021-08-31	Northeast	Delta
EPI_ISL_4275645	2021-08-31	RHD XV	Delta

EPI_ISL_4275650	2021-08-31	RHD XV	Delta
EPI_ISL_4275653	2021-08-31	RHD XV	Delta
EPI_ISL_4275651	2021-08-31	RHD XV	Delta
EPI_ISL_4275652	2021-08-31	RHD XV	Delta
EPI_ISL_4275649	2021-08-31	RHD XV	Delta
EPI_ISL_4275648	2021-08-31	RHD XV	Delta
EPI_ISL_4275646	2021-08-31	RHD XV	Delta
EPI_ISL_4275647	2021-08-31	RHD XV	Delta
EPI_ISL_4275641	2021-08-31	RHD XV	Delta
EPI_ISL_4275640	2021-08-31	RHD XV	Delta
EPI_ISL_4275635	2021-08-31	RHD XV	Delta
EPI_ISL_4275629	2021-08-31	RHD XV	Delta
EPI_ISL_4275617	2021-08-31	RHD XV	Delta
EPI_ISL_4275610	2021-08-31	RHD XV	Delta
EPI_ISL_4275593	2021-08-31	RHD XV	Delta
EPI_ISL_4275592	2021-08-31	RHD XV	Delta
EPI_ISL_11940829	2021-08-31	RHD XV	Delta
EPI_ISL_6898933	2021-08-31	Southeast	Delta
EPI_ISL_11940827	2021-08-31	RHD XV	Gamma
EPI_ISL_11940828	2021-08-31	RHD XV	Gamma
EPI_ISL_11940833	2021-08-31	RHD XV	Gamma
EPI_ISL_11940834	2021-08-31	RHD XV	Gamma
EPI_ISL_11940835	2021-08-31	RHD XV	Gamma
EPI_ISL_11940836	2021-08-31	RHD XV	Gamma
EPI_ISL_11940838	2021-08-31	RHD XV	Gamma
EPI_ISL_11940839	2021-08-31	RHD XV	Gamma
EPI_ISL_11940840	2021-08-31	RHD XV	Gamma
EPI_ISL_11940844	2021-08-31	RHD XV	Gamma
EPI_ISL_11940845	2021-08-31	RHD XV	Gamma
EPI_ISL_11940846	2021-08-31	RHD XV	Gamma
EPI_ISL_11940847	2021-08-31	RHD XV	Gamma
EPI_ISL_11940848	2021-08-31	RHD XV	Gamma
EPI_ISL_11940849	2021-08-31	RHD XV	Gamma
EPI_ISL_11940851	2021-08-31	RHD XV	Gamma
EPI_ISL_6172970	2021-08-31	South	Gamma
EPI_ISL_4275661	2021-09-01	RHD XV	Delta
EPI_ISL_4275660	2021-09-01	RHD XV	Delta
EPI_ISL_4275659	2021-09-01	RHD XV	Delta
EPI_ISL_4275658	2021-09-01	RHD XV	Delta
EPI_ISL_4275657	2021-09-01	RHD XV	Delta
EPI_ISL_4275656	2021-09-01	RHD XV	Delta
EPI_ISL_4275655	2021-09-01	RHD XV	Delta
EPI_ISL_4275654	2021-09-01	RHD XV	Delta
EPI_ISL_4275628	2021-09-01	RHD XV	Delta
EPI_ISL_4275627	2021-09-01	RHD XV	Delta
EPI_ISL_18620411	2021-09-01	RHD XV	Delta
EPI_ISL_4212779	2021-09-01	Southeast	Delta
EPI_ISL_5825579	2021-09-01	Northeast	Gamma
EPI_ISL_11940820	2021-09-01	RHD XV	Gamma
EPI_ISL_11940821	2021-09-01	RHD XV	Gamma
EPI_ISL_11940823	2021-09-01	RHD XV	Gamma
EPI_ISL_11940824	2021-09-01	RHD XV	Gamma
EPI_ISL_11940825	2021-09-01	RHD XV	Gamma
EPI_ISL_11940826	2021-09-01	RHD XV	Gamma
EPI_ISL_11940837	2021-09-01	RHD XV	Gamma
EPI_ISL_11940841	2021-09-01	RHD XV	Gamma
EPI_ISL_11940842	2021-09-01	RHD XV	Gamma
EPI_ISL_11940843	2021-09-01	RHD XV	Gamma
EPI_ISL_6940655	2021-09-01	South	Gamma
EPI_ISL_4632931	2021-09-01	South	Gamma
EPI_ISL_11940822	2021-09-01	RHD XV	Others
EPI_ISL_4728681	2021-09-02	Southeast	Delta
EPI_ISL_4516234	2021-09-02	North	Gamma

EPI_ISL_5825576	2021-09-02	Northeast	Gamma
EPI_ISL_6940561	2021-09-03	North	Delta
EPI_ISL_6229679	2021-09-03	Northeast	Delta
EPI_ISL_5915336	2021-09-03	Northeast	Delta
EPI_ISL_6173086	2021-09-03	Northeast	Delta
EPI_ISL_13565059	2021-09-03	South	Delta
EPI_ISL_4414912	2021-09-03	South	Delta
EPI_ISL_5501474	2021-09-03	Southeast	Delta
EPI_ISL_4516241	2021-09-03	North	Gamma
EPI_ISL_7132998	2021-09-03	Southeast	Gamma
EPI_ISL_4515995	2021-09-04	North	Delta
EPI_ISL_6172470	2021-09-04	North	Delta
EPI_ISL_6172907	2021-09-04	Northeast	Delta
EPI_ISL_4746839	2021-09-04	RHD XV	Delta
EPI_ISL_4746838	2021-09-04	RHD XV	Delta
EPI_ISL_6940563	2021-09-04	North	Gamma
EPI_ISL_11940855	2021-09-04	RHD XV	Gamma
EPI_ISL_11940858	2021-09-04	RHD XV	Gamma
EPI_ISL_4746786	2021-09-05	RHD XV	Delta
EPI_ISL_4746534	2021-09-05	RHD XV	Delta
EPI_ISL_4746692	2021-09-05	RHD XV	Delta
EPI_ISL_4746726	2021-09-05	RHD XV	Delta
EPI_ISL_18620412	2021-09-05	RHD XV	Delta
EPI_ISL_11940857	2021-09-05	RHD XV	Gamma
EPI_ISL_11940859	2021-09-05	RHD XV	Gamma
EPI_ISL_4746703	2021-09-06	RHD XV	Delta
EPI_ISL_4747281	2021-09-06	RHD XV	Delta
EPI_ISL_4746633	2021-09-06	RHD XV	Delta
EPI_ISL_6940656	2021-09-06	South	Delta
EPI_ISL_4212701	2021-09-06	Midwest	Gamma
EPI_ISL_6940560	2021-09-06	North	Gamma
EPI_ISL_6172483	2021-09-06	North	Gamma
EPI_ISL_11940861	2021-09-06	RHD XV	Gamma
EPI_ISL_11940862	2021-09-06	RHD XV	Gamma
EPI_ISL_11940863	2021-09-06	RHD XV	Gamma
EPI_ISL_4746615	2021-09-07	RHD XV	Delta
EPI_ISL_4746841	2021-09-07	RHD XV	Delta
EPI_ISL_4746480	2021-09-07	RHD XV	Delta
EPI_ISL_4747285	2021-09-07	RHD XV	Delta
EPI_ISL_4747284	2021-09-07	RHD XV	Delta
EPI_ISL_4746447	2021-09-07	RHD XV	Delta
EPI_ISL_4728783	2021-09-07	Southeast	Delta
EPI_ISL_6172495	2021-09-07	North	Gamma
EPI_ISL_11940852	2021-09-07	RHD XV	Gamma
EPI_ISL_11940853	2021-09-07	RHD XV	Gamma
EPI_ISL_11940860	2021-09-07	RHD XV	Gamma
EPI_ISL_4746454	2021-09-08	RHD XV	Delta
EPI_ISL_4746785	2021-09-08	RHD XV	Delta
EPI_ISL_4746702	2021-09-08	RHD XV	Delta
EPI_ISL_4746595	2021-09-08	RHD XV	Delta
EPI_ISL_4746840	2021-09-08	RHD XV	Delta
EPI_ISL_4746705	2021-09-08	RHD XV	Delta
EPI_ISL_4746449	2021-09-08	RHD XV	Delta
EPI_ISL_4746679	2021-09-08	RHD XV	Delta
EPI_ISL_4746712	2021-09-08	RHD XV	Delta
EPI_ISL_4746732	2021-09-08	RHD XV	Delta
EPI_ISL_4747280	2021-09-08	RHD XV	Delta
EPI_ISL_18620413	2021-09-08	RHD XV	Delta
EPI_ISL_4212693	2021-09-08	Midwest	Gamma
EPI_ISL_9800149	2021-09-08	Midwest	Gamma
EPI_ISL_11940854	2021-09-08	RHD XV	Gamma
EPI_ISL_11940856	2021-09-08	RHD XV	Gamma
EPI_ISL_6172639	2021-09-09	Northeast	Delta

EPI_ISL_5329012	2021-09-09	Northeast	Delta
EPI_ISL_6721723	2021-09-10	North	Delta
EPI_ISL_4474543	2021-09-10	Northeast	Delta
EPI_ISL_8630210	2021-09-10	Southeast	Delta
EPI_ISL_6474515	2021-09-10	Northeast	Gamma
EPI_ISL_11495519	2021-09-10	North	Others
EPI_ISL_4516015	2021-09-11	North	Delta
EPI_ISL_10101939	2021-09-11	Northeast	Delta
EPI_ISL_8055904	2021-09-11	Northeast	Gamma
EPI_ISL_5021564	2021-09-11	RHD XV	Gamma
EPI_ISL_9800471	2021-09-12	Midwest	Delta
EPI_ISL_5640180	2021-09-12	Southeast	Delta
EPI_ISL_5021574	2021-09-12	RHD XV	Gamma
EPI_ISL_5021575	2021-09-12	RHD XV	Gamma
EPI_ISL_6971010	2021-09-13	Northeast	Delta
EPI_ISL_5020930	2021-09-13	RHD XV	Delta
EPI_ISL_5020945	2021-09-13	RHD XV	Delta
EPI_ISL_5020952	2021-09-13	RHD XV	Delta
EPI_ISL_5020970	2021-09-13	RHD XV	Delta
EPI_ISL_5020994	2021-09-13	RHD XV	Delta
EPI_ISL_5020996	2021-09-13	RHD XV	Delta
EPI_ISL_5020998	2021-09-13	RHD XV	Delta
EPI_ISL_5021002	2021-09-13	RHD XV	Delta
EPI_ISL_5021081	2021-09-13	RHD XV	Delta
EPI_ISL_5021094	2021-09-13	RHD XV	Delta
EPI_ISL_5021222	2021-09-13	RHD XV	Delta
EPI_ISL_5021505	2021-09-13	RHD XV	Delta
EPI_ISL_5021511	2021-09-13	RHD XV	Delta
EPI_ISL_5021517	2021-09-13	RHD XV	Delta
EPI_ISL_5021521	2021-09-13	RHD XV	Delta
EPI_ISL_5021530	2021-09-13	RHD XV	Delta
EPI_ISL_5021535	2021-09-13	RHD XV	Delta
EPI_ISL_5021539	2021-09-13	RHD XV	Delta
EPI_ISL_5021540	2021-09-13	RHD XV	Delta
EPI_ISL_5021543	2021-09-13	RHD XV	Delta
EPI_ISL_5021549	2021-09-13	RHD XV	Delta
EPI_ISL_5021582	2021-09-13	RHD XV	Delta
EPI_ISL_5049591	2021-09-13	RHD XV	Delta
EPI_ISL_5049593	2021-09-13	RHD XV	Delta
EPI_ISL_4880262	2021-09-13	Southeast	Delta
EPI_ISL_5020917	2021-09-13	RHD XV	Gamma
EPI_ISL_5020965	2021-09-13	RHD XV	Gamma
EPI_ISL_5021504	2021-09-13	RHD XV	Gamma
EPI_ISL_5021548	2021-09-13	RHD XV	Gamma
EPI_ISL_5021579	2021-09-13	RHD XV	Gamma
EPI_ISL_5021583	2021-09-13	RHD XV	Gamma
EPI_ISL_5020990	2021-09-13	RHD XV	Others
EPI_ISL_5021522	2021-09-13	RHD XV	Others
EPI_ISL_7657560	2021-09-14	North	Delta
EPI_ISL_6474039	2021-09-14	Northeast	Delta
EPI_ISL_5021069	2021-09-14	RHD XV	Delta
EPI_ISL_5021071	2021-09-14	RHD XV	Delta
EPI_ISL_5021248	2021-09-14	RHD XV	Delta
EPI_ISL_5021556	2021-09-14	RHD XV	Delta
EPI_ISL_5049594	2021-09-14	RHD XV	Delta
EPI_ISL_5049595	2021-09-14	RHD XV	Delta
EPI_ISL_5049597	2021-09-14	RHD XV	Delta
EPI_ISL_5049598	2021-09-14	RHD XV	Delta
EPI_ISL_5049600	2021-09-14	RHD XV	Delta
EPI_ISL_5049601	2021-09-14	RHD XV	Delta
EPI_ISL_4955788	2021-09-14	South	Delta
EPI_ISL_5328992	2021-09-14	South	Delta
EPI_ISL_5390609	2021-09-14	Southeast	Delta

EPI_ISL_8630217	2021-09-14	Southeast	Delta
EPI_ISL_5021073	2021-09-14	RHD XV	Gamma
EPI_ISL_5049592	2021-09-14	RHD XV	Gamma
EPI_ISL_5049599	2021-09-14	RHD XV	Gamma
EPI_ISL_5049596	2021-09-15	RHD XV	Delta
EPI_ISL_5049603	2021-09-15	RHD XV	Delta
EPI_ISL_5049604	2021-09-15	RHD XV	Delta
EPI_ISL_5049605	2021-09-15	RHD XV	Delta
EPI_ISL_5049606	2021-09-15	RHD XV	Delta
EPI_ISL_5049608	2021-09-15	RHD XV	Delta
EPI_ISL_5049609	2021-09-15	RHD XV	Delta
EPI_ISL_5049611	2021-09-15	RHD XV	Delta
EPI_ISL_5049622	2021-09-15	RHD XV	Delta
EPI_ISL_5049623	2021-09-15	RHD XV	Delta
EPI_ISL_5049624	2021-09-15	RHD XV	Delta
EPI_ISL_5049607	2021-09-15	RHD XV	Gamma
EPI_ISL_5049610	2021-09-15	RHD XV	Gamma
EPI_ISL_5049619	2021-09-15	RHD XV	Gamma
EPI_ISL_5049620	2021-09-15	RHD XV	Gamma
EPI_ISL_5049621	2021-09-15	RHD XV	Gamma
EPI_ISL_5049602	2021-09-15	RHD XV	Others
EPI_ISL_13960035	2021-09-16	Midwest	Delta
EPI_ISL_8353171	2021-09-16	North	Delta
EPI_ISL_5049612	2021-09-16	RHD XV	Delta
EPI_ISL_5049613	2021-09-16	RHD XV	Delta
EPI_ISL_5049614	2021-09-16	RHD XV	Delta
EPI_ISL_5049618	2021-09-16	RHD XV	Delta
EPI_ISL_6971007	2021-09-17	Northeast	Delta
EPI_ISL_5254352	2021-09-18	Northeast	Delta
EPI_ISL_5254350	2021-09-19	Northeast	Delta
EPI_ISL_5489417	2021-09-19	North	Gamma
EPI_ISL_5254491	2021-09-19	RHD XV	Gamma
EPI_ISL_5254492	2021-09-20	RHD XV	Delta
EPI_ISL_5254493	2021-09-20	RHD XV	Delta
EPI_ISL_5254494	2021-09-20	RHD XV	Delta
EPI_ISL_5254495	2021-09-20	RHD XV	Delta
EPI_ISL_5254496	2021-09-20	RHD XV	Delta
EPI_ISL_5254497	2021-09-20	RHD XV	Delta
EPI_ISL_5254498	2021-09-20	RHD XV	Delta
EPI_ISL_5254499	2021-09-20	RHD XV	Delta
EPI_ISL_5254500	2021-09-20	RHD XV	Delta
EPI_ISL_5254501	2021-09-20	RHD XV	Delta
EPI_ISL_5254502	2021-09-20	RHD XV	Delta
EPI_ISL_5254503	2021-09-20	RHD XV	Delta
EPI_ISL_5254504	2021-09-20	RHD XV	Delta
EPI_ISL_5254559	2021-09-20	RHD XV	Delta
EPI_ISL_5254564	2021-09-20	RHD XV	Delta
EPI_ISL_5254505	2021-09-20	RHD XV	Delta
EPI_ISL_5254558	2021-09-20	RHD XV	Delta
EPI_ISL_5254555	2021-09-20	RHD XV	Delta
EPI_ISL_5254556	2021-09-20	RHD XV	Delta
EPI_ISL_5254557	2021-09-20	RHD XV	Delta
EPI_ISL_7109982	2021-09-20	South	Delta
EPI_ISL_5390689	2021-09-20	Southeast	Delta
EPI_ISL_4889933	2021-09-20	Midwest	Gamma
EPI_ISL_6172477	2021-09-21	North	Delta
EPI_ISL_8430554	2021-09-21	North	Delta
EPI_ISL_6172400	2021-09-21	Northeast	Delta
EPI_ISL_8055911	2021-09-21	Northeast	Delta
EPI_ISL_6943958	2021-09-21	Northeast	Delta
EPI_ISL_7614791	2021-09-21	RHD XV	Delta
EPI_ISL_16085326	2021-09-21	Southeast	Delta
EPI_ISL_7614788	2021-09-21	RHD XV	Gamma

EPI_ISL_5254364	2021-09-22	Northeast	Delta
EPI_ISL_8055915	2021-09-22	Northeast	Delta
EPI_ISL_5254506	2021-09-22	RHD XV	Delta
EPI_ISL_5254507	2021-09-22	RHD XV	Delta
EPI_ISL_5254508	2021-09-22	RHD XV	Delta
EPI_ISL_5254509	2021-09-22	RHD XV	Delta
EPI_ISL_5254510	2021-09-22	RHD XV	Delta
EPI_ISL_5254511	2021-09-22	RHD XV	Delta
EPI_ISL_5254512	2021-09-22	RHD XV	Delta
EPI_ISL_5254513	2021-09-22	RHD XV	Delta
EPI_ISL_5254514	2021-09-22	RHD XV	Delta
EPI_ISL_5254515	2021-09-22	RHD XV	Delta
EPI_ISL_5254516	2021-09-22	RHD XV	Delta
EPI_ISL_5254517	2021-09-22	RHD XV	Delta
EPI_ISL_5254518	2021-09-22	RHD XV	Delta
EPI_ISL_5254519	2021-09-22	RHD XV	Delta
EPI_ISL_5254520	2021-09-22	RHD XV	Delta
EPI_ISL_5254521	2021-09-22	RHD XV	Delta
EPI_ISL_5254523	2021-09-22	RHD XV	Delta
EPI_ISL_5254524	2021-09-22	RHD XV	Delta
EPI_ISL_5254525	2021-09-22	RHD XV	Delta
EPI_ISL_5254565	2021-09-22	RHD XV	Delta
EPI_ISL_5254527	2021-09-22	RHD XV	Delta
EPI_ISL_5254528	2021-09-22	RHD XV	Delta
EPI_ISL_5254561	2021-09-22	RHD XV	Delta
EPI_ISL_5254563	2021-09-22	RHD XV	Delta
EPI_ISL_5254562	2021-09-22	RHD XV	Delta
EPI_ISL_7106318	2021-09-22	South	Delta
EPI_ISL_8630240	2021-09-22	Southeast	Delta
EPI_ISL_5254522	2021-09-22	RHD XV	Gamma
EPI_ISL_5254526	2021-09-22	RHD XV	Gamma
EPI_ISL_5490905	2021-09-23	North	Delta
EPI_ISL_6721745	2021-09-23	North	Delta
EPI_ISL_7614801	2021-09-23	RHD XV	Delta
EPI_ISL_7614802	2021-09-23	RHD XV	Delta
EPI_ISL_7614795	2021-09-23	RHD XV	Delta
EPI_ISL_7614803	2021-09-23	RHD XV	Delta
EPI_ISL_8430557	2021-09-23	North	Gamma
EPI_ISL_8353175	2021-09-24	North	Delta
EPI_ISL_6172667	2021-09-24	Northeast	Delta
EPI_ISL_7106139	2021-09-24	South	Delta
EPI_ISL_14702173	2021-09-24	Southeast	Delta
EPI_ISL_8057715	2021-09-25	Northeast	Delta
EPI_ISL_6971024	2021-09-25	Northeast	Delta
EPI_ISL_6474547	2021-09-25	Northeast	Gamma
EPI_ISL_9527761	2021-09-26	Midwest	Delta
EPI_ISL_6173536	2021-09-26	Northeast	Delta
EPI_ISL_5689778	2021-09-27	North	Delta
EPI_ISL_8360696	2021-09-27	North	Delta
EPI_ISL_8430560	2021-09-27	North	Delta
EPI_ISL_5689136	2021-09-27	RHD XV	Delta
EPI_ISL_5689137	2021-09-27	RHD XV	Delta
EPI_ISL_5688395	2021-09-27	RHD XV	Delta
EPI_ISL_5689259	2021-09-27	RHD XV	Delta
EPI_ISL_5688526	2021-09-27	RHD XV	Delta
EPI_ISL_5688641	2021-09-27	RHD XV	Delta
EPI_ISL_5688651	2021-09-27	RHD XV	Delta
EPI_ISL_8325312	2021-09-27	Southeast	Delta
EPI_ISL_5689107	2021-09-27	RHD XV	Gamma
EPI_ISL_5689106	2021-09-27	RHD XV	Gamma
EPI_ISL_5689266	2021-09-27	RHD XV	Gamma
EPI_ISL_9800406	2021-09-28	Midwest	Delta
EPI_ISL_6474139	2021-09-28	Northeast	Delta

EPI_ISL_5688458	2021-09-28	RHD XV	Delta
EPI_ISL_5688468	2021-09-28	RHD XV	Delta
EPI_ISL_5688470	2021-09-28	RHD XV	Delta
EPI_ISL_7494055	2021-09-28	RHD XV	Delta
EPI_ISL_7494134	2021-09-28	RHD XV	Delta
EPI_ISL_6173403	2021-09-28	South	Delta
EPI_ISL_6854924	2021-09-28	Southeast	Delta
EPI_ISL_6898380	2021-09-29	North	Delta
EPI_ISL_8057730	2021-09-29	Northeast	Delta
EPI_ISL_5254529	2021-09-29	RHD XV	Delta
EPI_ISL_5254530	2021-09-29	RHD XV	Delta
EPI_ISL_5254531	2021-09-29	RHD XV	Delta
EPI_ISL_5254532	2021-09-29	RHD XV	Delta
EPI_ISL_5254533	2021-09-29	RHD XV	Delta
EPI_ISL_5254534	2021-09-29	RHD XV	Delta
EPI_ISL_5254535	2021-09-29	RHD XV	Delta
EPI_ISL_5254536	2021-09-29	RHD XV	Delta
EPI_ISL_5254537	2021-09-29	RHD XV	Delta
EPI_ISL_5254538	2021-09-29	RHD XV	Delta
EPI_ISL_5254539	2021-09-29	RHD XV	Delta
EPI_ISL_5254540	2021-09-29	RHD XV	Delta
EPI_ISL_5254541	2021-09-29	RHD XV	Delta
EPI_ISL_5254542	2021-09-29	RHD XV	Delta
EPI_ISL_5254543	2021-09-29	RHD XV	Delta
EPI_ISL_5254544	2021-09-29	RHD XV	Delta
EPI_ISL_5254545	2021-09-29	RHD XV	Delta
EPI_ISL_5254546	2021-09-29	RHD XV	Delta
EPI_ISL_5254547	2021-09-29	RHD XV	Delta
EPI_ISL_5254548	2021-09-29	RHD XV	Delta
EPI_ISL_5254560	2021-09-29	RHD XV	Delta
EPI_ISL_5254549	2021-09-29	RHD XV	Delta
EPI_ISL_5254550	2021-09-29	RHD XV	Delta
EPI_ISL_5254551	2021-09-29	RHD XV	Delta
EPI_ISL_5254552	2021-09-29	RHD XV	Delta
EPI_ISL_5254553	2021-09-29	RHD XV	Delta
EPI_ISL_5254554	2021-09-29	RHD XV	Delta
EPI_ISL_7494052	2021-09-29	RHD XV	Delta
EPI_ISL_7494151	2021-09-29	RHD XV	Delta
EPI_ISL_6970901	2021-09-30	Northeast	Delta
EPI_ISL_7614800	2021-09-30	RHD XV	Delta
EPI_ISL_7614796	2021-09-30	RHD XV	Delta
EPI_ISL_7494054	2021-09-30	RHD XV	Delta
EPI_ISL_7494130	2021-09-30	RHD XV	Delta
EPI_ISL_16084301	2021-09-30	Southeast	Delta
EPI_ISL_8360723	2021-09-30	North	Gamma
EPI_ISL_5689787	2021-10-01	North	Delta
EPI_ISL_8004525	2021-10-01	South	Delta
EPI_ISL_16084303	2021-10-01	Southeast	Delta
EPI_ISL_5490831	2021-10-01	North	Gamma
EPI_ISL_7455348	2021-10-02	North	Delta
EPI_ISL_6173378	2021-10-02	Northeast	Delta
EPI_ISL_6173539	2021-10-02	Northeast	Delta
EPI_ISL_6173027	2021-10-02	Southeast	Delta
EPI_ISL_6898381	2021-10-03	North	Delta
EPI_ISL_5915338	2021-10-03	Northeast	Delta
EPI_ISL_6474576	2021-10-03	Northeast	Gamma
EPI_ISL_5898585	2021-10-03	RHD XV	Gamma
EPI_ISL_8623163	2021-10-04	North	Delta
EPI_ISL_6173372	2021-10-04	Northeast	Delta
EPI_ISL_5898586	2021-10-04	RHD XV	Delta
EPI_ISL_5898815	2021-10-04	RHD XV	Delta
EPI_ISL_5898840	2021-10-04	RHD XV	Delta
EPI_ISL_5898881	2021-10-04	RHD XV	Delta

EPI_ISL_5898884	2021-10-04	RHD XV	Delta
EPI_ISL_5898888	2021-10-04	RHD XV	Delta
EPI_ISL_5898584	2021-10-04	RHD XV	Gamma
EPI_ISL_5621176	2021-10-05	North	Delta
EPI_ISL_8623174	2021-10-05	North	Delta
EPI_ISL_6573743	2021-10-05	Northeast	Delta
EPI_ISL_8318160	2021-10-05	Northeast	Delta
EPI_ISL_6573754	2021-10-05	Northeast	Delta
EPI_ISL_5898772	2021-10-05	RHD XV	Delta
EPI_ISL_6173437	2021-10-05	South	Delta
EPI_ISL_13287028	2021-10-06	North	Delta
EPI_ISL_13287029	2021-10-06	North	Delta
EPI_ISL_6573677	2021-10-06	Northeast	Delta
EPI_ISL_13690321	2021-10-06	South	Delta
EPI_ISL_8004528	2021-10-06	South	Delta
EPI_ISL_7455358	2021-10-06	North	Gamma
EPI_ISL_8360731	2021-10-06	North	Gamma
EPI_ISL_13287030	2021-10-07	North	Delta
EPI_ISL_7104389	2021-10-07	South	Delta
EPI_ISL_7103189	2021-10-07	Southeast	Delta
EPI_ISL_13287036	2021-10-08	North	Delta
EPI_ISL_13164663	2021-10-08	North	Gamma
EPI_ISL_6173207	2021-10-09	Southeast	Delta
EPI_ISL_13287039	2021-10-10	North	Delta
EPI_ISL_13287040	2021-10-11	North	Delta
EPI_ISL_7456285	2021-10-11	Northeast	Delta
EPI_ISL_6509911	2021-10-11	RHD XV	Delta
EPI_ISL_6510529	2021-10-11	RHD XV	Delta
EPI_ISL_6509837	2021-10-11	RHD XV	Delta
EPI_ISL_6509912	2021-10-11	RHD XV	Delta
EPI_ISL_6509592	2021-10-11	RHD XV	Delta
EPI_ISL_6509951	2021-10-11	RHD XV	Delta
EPI_ISL_6510567	2021-10-11	RHD XV	Delta
EPI_ISL_6510456	2021-10-11	RHD XV	Delta
EPI_ISL_6510517	2021-10-11	RHD XV	Delta
EPI_ISL_6510486	2021-10-11	RHD XV	Delta
EPI_ISL_6509904	2021-10-11	RHD XV	Delta
EPI_ISL_6898917	2021-10-12	Northeast	Delta
EPI_ISL_6810912	2021-10-12	Northeast	Delta
EPI_ISL_6509075	2021-10-12	RHD XV	Delta
EPI_ISL_6508692	2021-10-12	RHD XV	Delta
EPI_ISL_6509905	2021-10-12	RHD XV	Delta
EPI_ISL_6510557	2021-10-12	RHD XV	Delta
EPI_ISL_6510496	2021-10-12	RHD XV	Delta
EPI_ISL_6510447	2021-10-12	RHD XV	Delta
EPI_ISL_13459827	2021-10-12	South	Delta
EPI_ISL_13287042	2021-10-13	North	Delta
EPI_ISL_8360705	2021-10-13	North	Delta
EPI_ISL_7661119	2021-10-13	North	Delta
EPI_ISL_7456287	2021-10-13	Northeast	Delta
EPI_ISL_6508722	2021-10-13	RHD XV	Delta
EPI_ISL_6508724	2021-10-13	RHD XV	Delta
EPI_ISL_6508730	2021-10-13	RHD XV	Delta
EPI_ISL_6509937	2021-10-13	RHD XV	Delta
EPI_ISL_8630269	2021-10-13	Southeast	Delta
EPI_ISL_13287045	2021-10-14	North	Delta
EPI_ISL_13287027	2021-10-14	North	Delta
EPI_ISL_6772675	2021-10-14	North	Delta
EPI_ISL_7661122	2021-10-14	North	Delta
EPI_ISL_10101776	2021-10-14	Northeast	Delta
EPI_ISL_6898942	2021-10-14	Northeast	Delta
EPI_ISL_6173232	2021-10-14	Southeast	Delta
EPI_ISL_6173257	2021-10-15	North	Delta

EPI_ISL_6898943	2021-10-15	Northeast	Delta
EPI_ISL_6771811	2021-10-15	South	Delta
EPI_ISL_6941441	2021-10-16	Northeast	Delta
EPI_ISL_8056203	2021-10-17	North	Delta
EPI_ISL_6573648	2021-10-17	Northeast	Delta
EPI_ISL_6898919	2021-10-17	Northeast	Delta
EPI_ISL_6573715	2021-10-18	Northeast	Delta
EPI_ISL_7456292	2021-10-18	Northeast	Delta
EPI_ISL_6510558	2021-10-18	RHD XV	Delta
EPI_ISL_6510552	2021-10-18	RHD XV	Delta
EPI_ISL_6509900	2021-10-18	RHD XV	Delta
EPI_ISL_6509972	2021-10-18	RHD XV	Delta
EPI_ISL_6509913	2021-10-18	RHD XV	Delta
EPI_ISL_6509908	2021-10-18	RHD XV	Delta
EPI_ISL_6509891	2021-10-18	RHD XV	Delta
EPI_ISL_6510572	2021-10-18	RHD XV	Delta
EPI_ISL_6510454	2021-10-18	RHD XV	Delta
EPI_ISL_6510508	2021-10-18	RHD XV	Delta
EPI_ISL_6510504	2021-10-18	RHD XV	Delta
EPI_ISL_6510480	2021-10-18	RHD XV	Delta
EPI_ISL_6510509	2021-10-18	RHD XV	Delta
EPI_ISL_6510489	2021-10-18	RHD XV	Delta
EPI_ISL_6510488	2021-10-18	RHD XV	Delta
EPI_ISL_6509857	2021-10-18	RHD XV	Delta
EPI_ISL_6509882	2021-10-18	RHD XV	Delta
EPI_ISL_6509766	2021-10-18	RHD XV	Delta
EPI_ISL_8013267	2021-10-18	South	Delta
EPI_ISL_8013041	2021-10-18	Southeast	Delta
EPI_ISL_13459826	2021-10-19	South	Delta
EPI_ISL_13164679	2021-10-20	North	Delta
EPI_ISL_6810934	2021-10-20	Northeast	Delta
EPI_ISL_9800080	2021-10-21	Midwest	Delta
EPI_ISL_6494876	2021-10-21	North	Delta
EPI_ISL_6173455	2021-10-21	Southeast	Delta
EPI_ISL_13459814	2021-10-22	South	Delta
EPI_ISL_8630287	2021-10-22	Southeast	Delta
EPI_ISL_6854925	2021-10-22	Southeast	Delta
EPI_ISL_6894231	2021-10-23	RHD XV	Delta
EPI_ISL_6894232	2021-10-23	RHD XV	Delta
EPI_ISL_6894238	2021-10-23	RHD XV	Delta
EPI_ISL_8266269	2021-10-24	Midwest	Delta
EPI_ISL_8266267	2021-10-25	Midwest	Delta
EPI_ISL_8623229	2021-10-25	North	Delta
EPI_ISL_8360698	2021-10-25	North	Delta
EPI_ISL_6894219	2021-10-25	RHD XV	Delta
EPI_ISL_6894226	2021-10-25	RHD XV	Delta
EPI_ISL_6894242	2021-10-25	RHD XV	Delta
EPI_ISL_6894246	2021-10-25	RHD XV	Delta
EPI_ISL_6570455	2021-10-26	RHD XV	Delta
EPI_ISL_6570078	2021-10-26	RHD XV	Delta
EPI_ISL_6570106	2021-10-26	RHD XV	Delta
EPI_ISL_6570107	2021-10-26	RHD XV	Delta
EPI_ISL_6894210	2021-10-26	RHD XV	Delta
EPI_ISL_6894252	2021-10-26	RHD XV	Delta
EPI_ISL_18620414	2021-10-26	RHD XV	Delta
EPI_ISL_6810977	2021-10-27	Northeast	Delta
EPI_ISL_6570076	2021-10-27	RHD XV	Delta
EPI_ISL_6894178	2021-10-27	RHD XV	Delta
EPI_ISL_6894187	2021-10-27	RHD XV	Delta
EPI_ISL_6894190	2021-10-27	RHD XV	Delta
EPI_ISL_6894196	2021-10-27	RHD XV	Delta
EPI_ISL_6894203	2021-10-27	RHD XV	Delta
EPI_ISL_6894204	2021-10-27	RHD XV	Delta

EPI_ISL_6894214	2021-10-27	RHD XV	Delta
EPI_ISL_18620415	2021-10-27	RHD XV	Delta
EPI_ISL_8747987	2021-10-28	Northeast	Delta
EPI_ISL_6899048	2021-10-28	Southeast	Delta
EPI_ISL_9527750	2021-10-29	Midwest	Delta
EPI_ISL_18620396	2021-10-29	RHD XV	Delta
EPI_ISL_18620418	2021-10-29	RHD XV	Delta
EPI_ISL_18620419	2021-10-29	RHD XV	Delta
EPI_ISL_18620420	2021-10-29	RHD XV	Delta
EPI_ISL_18620421	2021-10-29	RHD XV	Delta
EPI_ISL_18620422	2021-10-29	RHD XV	Delta
EPI_ISL_8325320	2021-10-29	Southeast	Delta
EPI_ISL_6970485	2021-10-29	Southeast	Gamma
EPI_ISL_9800331	2021-10-31	Midwest	Delta
EPI_ISL_11815915	2021-10-31	North	Delta
EPI_ISL_18620416	2021-10-31	RHD XV	Delta
EPI_ISL_18620417	2021-10-31	RHD XV	Delta
EPI_ISL_6970493	2021-10-31	Southeast	Delta
EPI_ISL_12508303	2021-10-31	North	Gamma
EPI_ISL_8056237	2021-11-01	North	Delta
EPI_ISL_7455761	2021-11-01	North	Delta
EPI_ISL_12040004	2021-11-01	North	Delta
EPI_ISL_6813370	2021-11-01	RHD XV	Delta
EPI_ISL_6813376	2021-11-01	RHD XV	Delta
EPI_ISL_18620397	2021-11-01	RHD XV	Delta
EPI_ISL_18620423	2021-11-01	RHD XV	Delta
EPI_ISL_18620424	2021-11-01	RHD XV	Delta
EPI_ISL_18620425	2021-11-01	RHD XV	Delta
EPI_ISL_8880333	2021-11-01	South	Delta
EPI_ISL_7455762	2021-11-02	North	Delta
EPI_ISL_8748033	2021-11-02	Northeast	Delta
EPI_ISL_6813261	2021-11-02	RHD XV	Delta
EPI_ISL_18620398	2021-11-02	RHD XV	Delta
EPI_ISL_18620399	2021-11-02	RHD XV	Delta
EPI_ISL_18620400	2021-11-02	RHD XV	Delta
EPI_ISL_18620426	2021-11-02	RHD XV	Delta
EPI_ISL_12040013	2021-11-03	North	Delta
EPI_ISL_8748034	2021-11-03	Northeast	Delta
EPI_ISL_6813614	2021-11-03	RHD XV	Delta
EPI_ISL_8152435	2021-11-03	South	Delta
EPI_ISL_6771972	2021-11-03	South	Delta
EPI_ISL_9630673	2021-11-04	North	Delta
EPI_ISL_8056268	2021-11-04	North	Delta
EPI_ISL_9414732	2021-11-04	North	Delta
EPI_ISL_7661120	2021-11-04	North	Delta
EPI_ISL_8325195	2021-11-05	Midwest	Delta
EPI_ISL_8630303	2021-11-05	Southeast	Delta
EPI_ISL_8056305	2021-11-06	North	Delta
EPI_ISL_8152439	2021-11-06	South	Delta
EPI_ISL_8332048	2021-11-07	Southeast	Delta
EPI_ISL_9630677	2021-11-08	North	Delta
EPI_ISL_9414738	2021-11-08	North	Delta
EPI_ISL_6896380	2021-11-08	RHD XV	Delta
EPI_ISL_18620427	2021-11-08	RHD XV	Delta
EPI_ISL_8630301	2021-11-08	Southeast	Delta
EPI_ISL_9800318	2021-11-09	Midwest	Delta
EPI_ISL_8056345	2021-11-10	North	Delta
EPI_ISL_7661124	2021-11-11	North	Delta
EPI_ISL_8266368	2021-11-12	Northeast	Delta
EPI_ISL_8025138	2021-11-12	Northeast	Delta
EPI_ISL_9630748	2021-11-13	North	Delta
EPI_ISL_8056375	2021-11-13	North	Delta
EPI_ISL_8056376	2021-11-13	North	Delta

EPI_ISL_8748053	2021-11-13	Northeast	Delta
EPI_ISL_11016285	2021-11-15	Midwest	Delta
EPI_ISL_9630685	2021-11-15	North	Delta
EPI_ISL_8430606	2021-11-15	North	Delta
EPI_ISL_8127883	2021-11-15	Northeast	Delta
EPI_ISL_8056400	2021-11-16	North	Delta
EPI_ISL_8325061	2021-11-16	North	Delta
EPI_ISL_8005701	2021-11-16	Northeast	Delta
EPI_ISL_7132225	2021-11-16	RHD XV	Delta
EPI_ISL_7132482	2021-11-16	RHD XV	Delta
EPI_ISL_18620428	2021-11-16	RHD XV	Delta
EPI_ISL_12862763	2021-11-16	South	Delta
EPI_ISL_8004440	2021-11-16	South	Delta
EPI_ISL_9630688	2021-11-17	North	Delta
EPI_ISL_15729223	2021-11-17	Northeast	Delta
EPI_ISL_7982799	2021-11-17	Southeast	Delta
EPI_ISL_9630692	2021-11-18	North	Delta
EPI_ISL_9630749	2021-11-18	North	Delta
EPI_ISL_8325340	2021-11-18	Southeast	Delta
EPI_ISL_7132523	2021-11-19	RHD XV	Delta
EPI_ISL_8325323	2021-11-19	Southeast	Delta
EPI_ISL_9568493	2021-11-20	Midwest	Delta
EPI_ISL_11815919	2021-11-20	North	Delta
EPI_ISL_8013130	2021-11-20	North	Delta
EPI_ISL_8127894	2021-11-20	Northeast	Delta
EPI_ISL_7132711	2021-11-20	RHD XV	Delta
EPI_ISL_7132491	2021-11-21	RHD XV	Delta
EPI_ISL_7132480	2021-11-22	RHD XV	Delta
EPI_ISL_7665166	2021-11-22	RHD XV	Delta
EPI_ISL_7665165	2021-11-22	RHD XV	Delta
EPI_ISL_7665139	2021-11-22	RHD XV	Delta
EPI_ISL_7982803	2021-11-22	Southeast	Delta
EPI_ISL_8430613	2021-11-23	North	Delta
EPI_ISL_8127903	2021-11-23	Northeast	Delta
EPI_ISL_8025175	2021-11-23	Northeast	Delta
EPI_ISL_7666253	2021-11-23	RHD XV	Delta
EPI_ISL_7898655	2021-11-23	Southeast	Delta
EPI_ISL_9800339	2021-11-24	Midwest	Delta
EPI_ISL_7666698	2021-11-24	RHD XV	Delta
EPI_ISL_7666523	2021-11-24	RHD XV	Delta
EPI_ISL_9800329	2021-11-25	Midwest	Delta
EPI_ISL_7666486	2021-11-25	RHD XV	Delta
EPI_ISL_18620433	2021-11-25	RHD XV	Delta
EPI_ISL_8630319	2021-11-26	North	Delta
EPI_ISL_8266308	2021-11-26	North	Delta
EPI_ISL_7666520	2021-11-26	RHD XV	Delta
EPI_ISL_7666647	2021-11-26	RHD XV	Delta
EPI_ISL_7666541	2021-11-26	RHD XV	Delta
EPI_ISL_7666603	2021-11-26	RHD XV	Delta
EPI_ISL_18620429	2021-11-26	RHD XV	Delta
EPI_ISL_18620430	2021-11-26	RHD XV	Delta
EPI_ISL_18620431	2021-11-26	RHD XV	Delta
EPI_ISL_18620432	2021-11-26	RHD XV	Delta
EPI_ISL_18620434	2021-11-26	RHD XV	Delta
EPI_ISL_8630318	2021-11-26	Southeast	Delta
EPI_ISL_13459851	2021-11-27	South	Delta
EPI_ISL_9793936	2021-11-28	Northeast	Delta
EPI_ISL_7666458	2021-11-28	RHD XV	Delta
EPI_ISL_9568500	2021-11-29	Midwest	Delta
EPI_ISL_8152492	2021-11-29	North	Delta
EPI_ISL_9794029	2021-11-29	Northeast	Delta
EPI_ISL_8633941	2021-11-29	Northeast	Delta
EPI_ISL_7809144	2021-11-29	RHD XV	Delta

EPI_ISL_7809172	2021-11-29	RHD XV	Delta
EPI_ISL_7809383	2021-11-29	RHD XV	Delta
EPI_ISL_7809386	2021-11-29	RHD XV	Delta
EPI_ISL_8466930	2021-11-30	Northeast	Delta
EPI_ISL_8466866	2021-11-30	Northeast	Delta
EPI_ISL_7809145	2021-11-30	RHD XV	Delta
EPI_ISL_7809334	2021-11-30	RHD XV	Delta
EPI_ISL_18620435	2021-11-30	RHD XV	Delta
EPI_ISL_15456882	2021-11-30	South	Delta
EPI_ISL_8056539	2021-12-01	North	Delta
EPI_ISL_15457733	2021-12-01	North	Delta
EPI_ISL_9794026	2021-12-01	Northeast	Delta
EPI_ISL_8633949	2021-12-01	Northeast	Delta
EPI_ISL_8152476	2021-12-01	South	Delta
EPI_ISL_15456884	2021-12-01	South	Delta
EPI_ISL_8630322	2021-12-01	Southeast	Delta
EPI_ISL_14670067	2021-12-01	Midwest	Gamma
EPI_ISL_13960034	2021-12-01	Midwest	Zeta
EPI_ISL_15463294	2021-12-02	North	Delta
EPI_ISL_10901894	2021-12-02	Northeast	Delta
EPI_ISL_9794043	2021-12-02	Northeast	Delta
EPI_ISL_15456886	2021-12-02	South	Delta
EPI_ISL_9670608	2021-12-03	Northeast	Delta
EPI_ISL_9568511	2021-12-05	Midwest	Delta
EPI_ISL_13833618	2021-12-05	Southeast	Delta
EPI_ISL_7796051	2021-12-06	Southeast	Delta
EPI_ISL_9671431	2021-12-07	North	Delta
EPI_ISL_11811106	2021-12-07	North	Delta
EPI_ISL_9274957	2021-12-07	Southeast	Delta
EPI_ISL_15456933	2021-12-08	Midwest	Delta
EPI_ISL_8515157	2021-12-08	North	Delta
EPI_ISL_9794151	2021-12-08	Northeast	Delta
EPI_ISL_8025226	2021-12-08	Northeast	Delta
EPI_ISL_8151561	2021-12-08	South	Delta
EPI_ISL_7899761	2021-12-08	RHD XV	Omicron
EPI_ISL_9630697	2021-12-09	North	Delta
EPI_ISL_18620503	2021-12-09	RHD XV	Omicron
EPI_ISL_18620402	2021-12-10	RHD XV	Delta
EPI_ISL_9274958	2021-12-10	Southeast	Gamma
EPI_ISL_12036871	2021-12-11	North	Delta
EPI_ISL_8466868	2021-12-11	Northeast	Delta
EPI_ISL_13833621	2021-12-11	Southeast	Delta
EPI_ISL_8515172	2021-12-12	North	Delta
EPI_ISL_18620403	2021-12-12	RHD XV	Delta
EPI_ISL_8325212	2021-12-13	Midwest	Delta
EPI_ISL_9630744	2021-12-13	North	Delta
EPI_ISL_9794189	2021-12-13	Northeast	Delta
EPI_ISL_8633965	2021-12-13	Northeast	Delta
EPI_ISL_8325350	2021-12-13	Northeast	Delta
EPI_ISL_8401504	2021-12-13	RHD XV	Delta
EPI_ISL_8401525	2021-12-13	RHD XV	Delta
EPI_ISL_8401538	2021-12-13	RHD XV	Delta
EPI_ISL_18620404	2021-12-13	RHD XV	Delta
EPI_ISL_18620437	2021-12-13	RHD XV	Delta
EPI_ISL_8266439	2021-12-13	South	Delta
EPI_ISL_10083540	2021-12-14	North	Delta
EPI_ISL_15456892	2021-12-14	South	Delta
EPI_ISL_15456935	2021-12-15	Midwest	Delta
EPI_ISL_9630704	2021-12-15	North	Delta
EPI_ISL_18620449	2021-12-15	RHD XV	Delta
EPI_ISL_18620409	2021-12-15	RHD XV	Delta
EPI_ISL_8708704	2021-12-16	Northeast	Delta
EPI_ISL_11330950	2021-12-16	Southeast	Delta

EPI_ISL_15456936	2021-12-17	Midwest	Delta
EPI_ISL_9630746	2021-12-17	North	Delta
EPI_ISL_9636859	2021-12-17	North	Delta
EPI_ISL_18620438	2021-12-17	RHD XV	Delta
EPI_ISL_18620439	2021-12-17	RHD XV	Delta
EPI_ISL_8633975	2021-12-18	Northeast	Delta
EPI_ISL_11330951	2021-12-18	Southeast	Delta
EPI_ISL_18620440	2021-12-18	RHD XV	Omicron
EPI_ISL_14674380	2021-12-20	Midwest	Delta
EPI_ISL_9630747	2021-12-20	North	Delta
EPI_ISL_9215991	2021-12-20	Northeast	Delta
EPI_ISL_18620441	2021-12-20	RHD XV	Delta
EPI_ISL_18620451	2021-12-20	RHD XV	Delta
EPI_ISL_18620442	2021-12-20	RHD XV	Delta
EPI_ISL_18620443	2021-12-20	RHD XV	Omicron
EPI_ISL_18620450	2021-12-20	RHD XV	Omicron
EPI_ISL_18620452	2021-12-20	RHD XV	Omicron
EPI_ISL_18620410	2021-12-20	RHD XV	Omicron
EPI_ISL_18620453	2021-12-20	RHD XV	Omicron
EPI_ISL_9568518	2021-12-21	Midwest	Delta
EPI_ISL_9630710	2021-12-21	North	Delta
EPI_ISL_12862757	2021-12-21	South	Delta
EPI_ISL_18620444	2021-12-21	RHD XV	Omicron
EPI_ISL_18620445	2021-12-21	RHD XV	Omicron
EPI_ISL_18620446	2021-12-21	RHD XV	Omicron
EPI_ISL_18620447	2021-12-21	RHD XV	Omicron
EPI_ISL_8633917	2021-12-22	Northeast	Delta
EPI_ISL_9274972	2021-12-22	Southeast	Delta
EPI_ISL_18620454	2021-12-22	RHD XV	Omicron
EPI_ISL_18620455	2021-12-22	RHD XV	Omicron
EPI_ISL_18620456	2021-12-22	RHD XV	Omicron
EPI_ISL_9216000	2021-12-23	Northeast	Delta
EPI_ISL_11016314	2021-12-23	Northeast	Delta
EPI_ISL_9274978	2021-12-23	Southeast	Omicron
EPI_ISL_12043471	2021-12-24	Midwest	Delta
EPI_ISL_15463432	2021-12-25	Northeast	Delta
EPI_ISL_12838678	2021-12-26	North	Delta
EPI_ISL_9414752	2021-12-27	North	Delta
EPI_ISL_11811126	2021-12-27	North	Delta
EPI_ISL_14674348	2021-12-28	Midwest	Delta
EPI_ISL_9414753	2021-12-28	North	Delta
EPI_ISL_8466870	2021-12-28	Northeast	Delta
EPI_ISL_9305188	2021-12-29	RHD XV	Omicron
EPI_ISL_18620504	2021-12-29	RHD XV	Omicron
EPI_ISL_9568618	2021-12-30	Midwest	Delta
EPI_ISL_8554953	2021-12-30	North	Delta
EPI_ISL_9305178	2021-12-30	RHD XV	Omicron
EPI_ISL_9305179	2021-12-30	RHD XV	Omicron
EPI_ISL_9305180	2021-12-30	RHD XV	Omicron
EPI_ISL_9305182	2021-12-30	RHD XV	Omicron
EPI_ISL_9305183	2021-12-30	RHD XV	Omicron
EPI_ISL_9305184	2021-12-30	RHD XV	Omicron
EPI_ISL_9305181	2021-12-30	RHD XV	Omicron
EPI_ISL_9305175	2021-12-31	RHD XV	Omicron
EPI_ISL_9305176	2021-12-31	RHD XV	Omicron
EPI_ISL_9305186	2021-12-31	RHD XV	Omicron
EPI_ISL_9305187	2021-12-31	RHD XV	Omicron
EPI_ISL_9304344	2021-12-31	RHD XV	Omicron
EPI_ISL_9304397	2021-12-31	RHD XV	Omicron
EPI_ISL_9304398	2021-12-31	RHD XV	Omicron
EPI_ISL_9304399	2021-12-31	RHD XV	Omicron
EPI_ISL_9304400	2021-12-31	RHD XV	Omicron
EPI_ISL_9304401	2021-12-31	RHD XV	Omicron

EPI_ISL_9304402	2021-12-31	RHD XV	Omicron
EPI_ISL_9305185	2022-01-01	RHD XV	Omicron
EPI_ISL_9304407	2022-01-01	RHD XV	Omicron
EPI_ISL_9304423	2022-01-01	RHD XV	Omicron
EPI_ISL_14674388	2022-01-02	Midwest	Delta
EPI_ISL_9304403	2022-01-02	RHD XV	Omicron
EPI_ISL_9304404	2022-01-02	RHD XV	Omicron
EPI_ISL_9304405	2022-01-02	RHD XV	Omicron
EPI_ISL_9304408	2022-01-02	RHD XV	Omicron
EPI_ISL_9304418	2022-01-02	RHD XV	Omicron
EPI_ISL_9304419	2022-01-02	RHD XV	Omicron
EPI_ISL_9304421	2022-01-02	RHD XV	Omicron
EPI_ISL_18620436	2022-01-02	RHD XV	Omicron
EPI_ISL_9194769	2022-01-03	North	Delta
EPI_ISL_9636845	2022-01-03	North	Delta
EPI_ISL_18620458	2022-01-03	RHD XV	Delta
EPI_ISL_18620463	2022-01-03	RHD XV	Delta
EPI_ISL_18620471	2022-01-03	RHD XV	Delta
EPI_ISL_9321075	2022-01-03	Southeast	Delta
EPI_ISL_9304406	2022-01-03	RHD XV	Omicron
EPI_ISL_9304409	2022-01-03	RHD XV	Omicron
EPI_ISL_9304410	2022-01-03	RHD XV	Omicron
EPI_ISL_9304411	2022-01-03	RHD XV	Omicron
EPI_ISL_9304412	2022-01-03	RHD XV	Omicron
EPI_ISL_9304413	2022-01-03	RHD XV	Omicron
EPI_ISL_9304414	2022-01-03	RHD XV	Omicron
EPI_ISL_9304415	2022-01-03	RHD XV	Omicron
EPI_ISL_9304422	2022-01-03	RHD XV	Omicron
EPI_ISL_9304437	2022-01-03	RHD XV	Omicron
EPI_ISL_18620401	2022-01-03	RHD XV	Omicron
EPI_ISL_18620494	2022-01-03	RHD XV	Omicron
EPI_ISL_18620457	2022-01-03	RHD XV	Omicron
EPI_ISL_18620506	2022-01-03	RHD XV	Omicron
EPI_ISL_18620459	2022-01-03	RHD XV	Omicron
EPI_ISL_18620460	2022-01-03	RHD XV	Omicron
EPI_ISL_18620461	2022-01-03	RHD XV	Omicron
EPI_ISL_18620462	2022-01-03	RHD XV	Omicron
EPI_ISL_18620495	2022-01-03	RHD XV	Omicron
EPI_ISL_18620464	2022-01-03	RHD XV	Omicron
EPI_ISL_18620465	2022-01-03	RHD XV	Omicron
EPI_ISL_18620493	2022-01-03	RHD XV	Omicron
EPI_ISL_18620466	2022-01-03	RHD XV	Omicron
EPI_ISL_18620467	2022-01-03	RHD XV	Omicron
EPI_ISL_18620496	2022-01-03	RHD XV	Omicron
EPI_ISL_18620505	2022-01-03	RHD XV	Omicron
EPI_ISL_18620468	2022-01-03	RHD XV	Omicron
EPI_ISL_18620469	2022-01-03	RHD XV	Omicron
EPI_ISL_18620470	2022-01-03	RHD XV	Omicron
EPI_ISL_9275005	2022-01-03	Southeast	Omicron
EPI_ISL_11124707	2022-01-04	North	Delta
EPI_ISL_8708712	2022-01-04	Northeast	Delta
EPI_ISL_9304416	2022-01-04	RHD XV	Omicron
EPI_ISL_15604599	2022-01-04	Southeast	Omicron
EPI_ISL_11124712	2022-01-05	North	Delta
EPI_ISL_9266661	2022-01-05	Northeast	Delta
EPI_ISL_18620472	2022-01-05	RHD XV	Omicron
EPI_ISL_18620473	2022-01-05	RHD XV	Omicron
EPI_ISL_18620474	2022-01-05	RHD XV	Omicron
EPI_ISL_18620475	2022-01-05	RHD XV	Omicron
EPI_ISL_18620476	2022-01-05	RHD XV	Omicron
EPI_ISL_18620498	2022-01-05	RHD XV	Omicron
EPI_ISL_18620499	2022-01-05	RHD XV	Omicron
EPI_ISL_18620477	2022-01-05	RHD XV	Omicron

EPI_ISL_18620478	2022-01-05	RHD XV	Omicron
EPI_ISL_18620479	2022-01-05	RHD XV	Omicron
EPI_ISL_18620480	2022-01-05	RHD XV	Omicron
EPI_ISL_18620497	2022-01-05	RHD XV	Omicron
EPI_ISL_18620481	2022-01-05	RHD XV	Omicron
EPI_ISL_18620482	2022-01-05	RHD XV	Omicron
EPI_ISL_18620483	2022-01-05	RHD XV	Omicron
EPI_ISL_18620484	2022-01-05	RHD XV	Omicron
EPI_ISL_18620485	2022-01-05	RHD XV	Omicron
EPI_ISL_18620486	2022-01-05	RHD XV	Omicron
EPI_ISL_18620487	2022-01-05	RHD XV	Omicron
EPI_ISL_18620488	2022-01-05	RHD XV	Omicron
EPI_ISL_18620489	2022-01-05	RHD XV	Omicron
EPI_ISL_18620490	2022-01-05	RHD XV	Omicron
EPI_ISL_18620491	2022-01-05	RHD XV	Omicron
EPI_ISL_18620492	2022-01-05	RHD XV	Omicron
EPI_ISL_12883430	2022-01-05	Southeast	Omicron
EPI_ISL_9304439	2022-01-06	RHD XV	Omicron
EPI_ISL_9304442	2022-01-06	RHD XV	Omicron
EPI_ISL_9304443	2022-01-06	RHD XV	Omicron
EPI_ISL_9304444	2022-01-06	RHD XV	Omicron
EPI_ISL_9304445	2022-01-06	RHD XV	Omicron
EPI_ISL_9304446	2022-01-06	RHD XV	Omicron
EPI_ISL_9304447	2022-01-06	RHD XV	Omicron
EPI_ISL_9304449	2022-01-06	RHD XV	Omicron
EPI_ISL_9304448	2022-01-06	RHD XV	Omicron
EPI_ISL_10322494	2022-01-07	North	Delta
EPI_ISL_9304438	2022-01-07	RHD XV	Omicron
EPI_ISL_15604608	2022-01-07	Southeast	Omicron
EPI_ISL_12571031	2022-01-08	Northeast	Omicron
EPI_ISL_9568619	2022-01-09	Midwest	Delta
EPI_ISL_12571071	2022-01-09	Northeast	Omicron
EPI_ISL_9304450	2022-01-09	RHD XV	Omicron
EPI_ISL_9476206	2022-01-09	RHD XV	Omicron
EPI_ISL_9521259	2022-01-10	Midwest	Omicron
EPI_ISL_9304456	2022-01-10	RHD XV	Omicron
EPI_ISL_9304458	2022-01-10	RHD XV	Omicron
EPI_ISL_9304459	2022-01-10	RHD XV	Omicron
EPI_ISL_9476251	2022-01-10	RHD XV	Omicron
EPI_ISL_9476254	2022-01-10	RHD XV	Omicron
EPI_ISL_9476255	2022-01-10	RHD XV	Omicron
EPI_ISL_9476256	2022-01-10	RHD XV	Omicron
EPI_ISL_9225849	2022-01-11	Southeast	Delta
EPI_ISL_9304470	2022-01-11	RHD XV	Omicron
EPI_ISL_9304481	2022-01-11	RHD XV	Omicron
EPI_ISL_9304482	2022-01-11	RHD XV	Omicron
EPI_ISL_9304486	2022-01-11	RHD XV	Omicron
EPI_ISL_9304487	2022-01-11	RHD XV	Omicron
EPI_ISL_9304492	2022-01-11	RHD XV	Omicron
EPI_ISL_9304493	2022-01-11	RHD XV	Omicron
EPI_ISL_9304494	2022-01-11	RHD XV	Omicron
EPI_ISL_9304495	2022-01-11	RHD XV	Omicron
EPI_ISL_9304496	2022-01-11	RHD XV	Omicron
EPI_ISL_9304499	2022-01-11	RHD XV	Omicron
EPI_ISL_9304500	2022-01-11	RHD XV	Omicron
EPI_ISL_9304501	2022-01-11	RHD XV	Omicron
EPI_ISL_9304502	2022-01-11	RHD XV	Omicron
EPI_ISL_9476205	2022-01-11	RHD XV	Omicron
EPI_ISL_9476208	2022-01-11	RHD XV	Omicron
EPI_ISL_9476209	2022-01-11	RHD XV	Omicron
EPI_ISL_9476212	2022-01-11	RHD XV	Omicron
EPI_ISL_9476232	2022-01-11	RHD XV	Omicron
EPI_ISL_9476239	2022-01-11	RHD XV	Omicron

EPI_ISL_9476250	2022-01-11	RHD XV	Omicron
EPI_ISL_18620500	2022-01-11	RHD XV	Omicron
EPI_ISL_18620501	2022-01-11	RHD XV	Omicron
EPI_ISL_18620502	2022-01-11	RHD XV	Omicron
EPI_ISL_18620448	2022-01-11	RHD XV	Omicron
EPI_ISL_18620408	2022-01-11	RHD XV	Omicron
EPI_ISL_9225768	2022-01-12	Southeast	Delta
EPI_ISL_9304460	2022-01-12	RHD XV	Omicron
EPI_ISL_9304462	2022-01-12	RHD XV	Omicron
EPI_ISL_9304463	2022-01-12	RHD XV	Omicron
EPI_ISL_9304465	2022-01-12	RHD XV	Omicron
EPI_ISL_9304466	2022-01-12	RHD XV	Omicron
EPI_ISL_9304468	2022-01-12	RHD XV	Omicron
EPI_ISL_9304469	2022-01-12	RHD XV	Omicron
EPI_ISL_9304476	2022-01-12	RHD XV	Omicron
EPI_ISL_9304477	2022-01-12	RHD XV	Omicron
EPI_ISL_9304478	2022-01-12	RHD XV	Omicron
EPI_ISL_9304479	2022-01-12	RHD XV	Omicron
EPI_ISL_9304480	2022-01-12	RHD XV	Omicron
EPI_ISL_9304488	2022-01-12	RHD XV	Omicron
EPI_ISL_9304489	2022-01-12	RHD XV	Omicron
EPI_ISL_9304498	2022-01-12	RHD XV	Omicron
EPI_ISL_9476199	2022-01-12	RHD XV	Omicron
EPI_ISL_9476200	2022-01-12	RHD XV	Omicron
EPI_ISL_9476201	2022-01-12	RHD XV	Omicron
EPI_ISL_9476202	2022-01-12	RHD XV	Omicron
EPI_ISL_9476211	2022-01-12	RHD XV	Omicron
EPI_ISL_9476213	2022-01-12	RHD XV	Omicron
EPI_ISL_9476214	2022-01-12	RHD XV	Omicron
EPI_ISL_9476215	2022-01-12	RHD XV	Omicron
EPI_ISL_9476216	2022-01-12	RHD XV	Omicron
EPI_ISL_9476247	2022-01-12	RHD XV	Omicron
EPI_ISL_9416869	2022-01-13	North	Omicron
EPI_ISL_9304461	2022-01-13	RHD XV	Omicron
EPI_ISL_12883435	2022-01-13	Southeast	Omicron
EPI_ISL_12060046	2022-01-14	South	Delta
EPI_ISL_9304452	2022-01-14	RHD XV	Omicron
EPI_ISL_9304453	2022-01-14	RHD XV	Omicron
EPI_ISL_9304454	2022-01-14	RHD XV	Omicron
EPI_ISL_9304451	2022-01-14	RHD XV	Omicron
EPI_ISL_10706103	2022-01-17	Southeast	Omicron
EPI_ISL_9476186	2022-01-18	RHD XV	Omicron
EPI_ISL_9476187	2022-01-18	RHD XV	Omicron
EPI_ISL_9476188	2022-01-18	RHD XV	Omicron
EPI_ISL_9476189	2022-01-18	RHD XV	Omicron
EPI_ISL_9476229	2022-01-18	RHD XV	Omicron
EPI_ISL_9476230	2022-01-18	RHD XV	Omicron
EPI_ISL_9476231	2022-01-18	RHD XV	Omicron
EPI_ISL_9476240	2022-01-18	RHD XV	Omicron
EPI_ISL_12883418	2022-01-18	Southeast	Omicron
EPI_ISL_9476185	2022-01-19	RHD XV	Omicron
EPI_ISL_9476190	2022-01-19	RHD XV	Omicron
EPI_ISL_9476217	2022-01-19	RHD XV	Omicron
EPI_ISL_9476218	2022-01-19	RHD XV	Omicron
EPI_ISL_9476219	2022-01-19	RHD XV	Omicron
EPI_ISL_9476220	2022-01-19	RHD XV	Omicron
EPI_ISL_9476221	2022-01-19	RHD XV	Omicron
EPI_ISL_9476223	2022-01-19	RHD XV	Omicron
EPI_ISL_9476224	2022-01-19	RHD XV	Omicron
EPI_ISL_9476226	2022-01-19	RHD XV	Omicron
EPI_ISL_9476227	2022-01-19	RHD XV	Omicron
EPI_ISL_9476489	2022-01-19	RHD XV	Omicron
EPI_ISL_9476260	2022-01-19	RHD XV	Omicron

EPI_ISL_9476263	2022-01-19	RHD XV	Omicron
EPI_ISL_9476264	2022-01-19	RHD XV	Omicron
EPI_ISL_18620406	2022-01-19	RHD XV	Omicron
EPI_ISL_18620407	2022-01-19	RHD XV	Omicron
EPI_ISL_9476191	2022-01-20	RHD XV	Omicron
EPI_ISL_9476192	2022-01-20	RHD XV	Omicron
EPI_ISL_9476193	2022-01-20	RHD XV	Omicron
EPI_ISL_9476194	2022-01-20	RHD XV	Omicron
EPI_ISL_9476195	2022-01-20	RHD XV	Omicron
EPI_ISL_9476196	2022-01-20	RHD XV	Omicron
EPI_ISL_9476439	2022-01-20	RHD XV	Omicron
EPI_ISL_9476242	2022-01-20	RHD XV	Omicron
EPI_ISL_9476243	2022-01-20	RHD XV	Omicron
EPI_ISL_9476244	2022-01-20	RHD XV	Omicron
EPI_ISL_9500047	2022-01-21	North	Omicron
EPI_ISL_13833667	2022-01-22	Southeast	Delta
EPI_ISL_13088660	2022-01-23	South	Delta
EPI_ISL_12573107	2022-01-24	Northeast	Omicron
EPI_ISL_12883431	2022-01-25	Southeast	Omicron
EPI_ISL_12780400	2022-01-27	Northeast	Omicron
EPI_ISL_9805204	2022-01-27	RHD XV	Omicron
EPI_ISL_9805381	2022-01-27	RHD XV	Omicron
EPI_ISL_9805336	2022-01-27	RHD XV	Omicron
EPI_ISL_9805294	2022-01-27	RHD XV	Omicron
EPI_ISL_9805308	2022-01-27	RHD XV	Omicron
EPI_ISL_9805219	2022-01-27	RHD XV	Omicron
EPI_ISL_9805412	2022-01-27	RHD XV	Omicron
EPI_ISL_9805196	2022-01-27	RHD XV	Omicron
EPI_ISL_9805398	2022-01-27	RHD XV	Omicron
EPI_ISL_9805194	2022-01-27	RHD XV	Omicron
EPI_ISL_9805193	2022-01-27	RHD XV	Omicron
EPI_ISL_9805382	2022-01-27	RHD XV	Omicron
EPI_ISL_9805222	2022-01-27	RHD XV	Omicron
EPI_ISL_9805340	2022-01-27	RHD XV	Omicron
EPI_ISL_9805391	2022-01-27	RHD XV	Omicron
EPI_ISL_9805189	2022-01-27	RHD XV	Omicron
EPI_ISL_9805300	2022-01-27	RHD XV	Omicron
EPI_ISL_9805311	2022-01-27	RHD XV	Omicron
EPI_ISL_9805147	2022-01-27	RHD XV	Omicron
EPI_ISL_9805372	2022-01-27	RHD XV	Omicron
EPI_ISL_9805285	2022-01-27	RHD XV	Omicron
EPI_ISL_9805149	2022-01-27	RHD XV	Omicron
EPI_ISL_9805228	2022-01-27	RHD XV	Omicron
EPI_ISL_9805383	2022-01-28	RHD XV	Omicron
EPI_ISL_9805156	2022-01-28	RHD XV	Omicron
EPI_ISL_9805251	2022-01-28	RHD XV	Omicron
EPI_ISL_9805320	2022-01-28	RHD XV	Omicron
EPI_ISL_9805352	2022-01-28	RHD XV	Omicron
EPI_ISL_9805388	2022-01-28	RHD XV	Omicron
EPI_ISL_9805210	2022-01-28	RHD XV	Omicron
EPI_ISL_9805386	2022-01-28	RHD XV	Omicron
EPI_ISL_9805343	2022-01-28	RHD XV	Omicron
EPI_ISL_9805208	2022-01-28	RHD XV	Omicron
EPI_ISL_9805221	2022-01-28	RHD XV	Omicron
EPI_ISL_9805212	2022-01-28	RHD XV	Omicron
EPI_ISL_9805330	2022-01-28	RHD XV	Omicron
EPI_ISL_9805191	2022-01-28	RHD XV	Omicron
EPI_ISL_9805377	2022-01-28	RHD XV	Omicron
EPI_ISL_9805232	2022-01-28	RHD XV	Omicron
EPI_ISL_9805199	2022-01-28	RHD XV	Omicron
EPI_ISL_10309541	2022-02-01	RHD XV	Omicron
EPI_ISL_10309111	2022-02-02	RHD XV	Omicron
EPI_ISL_10309468	2022-02-02	RHD XV	Omicron

EPI_ISL_10309100	2022-02-02	RHD XV	Omicron
EPI_ISL_10309069	2022-02-02	RHD XV	Omicron
EPI_ISL_10309041	2022-02-02	RHD XV	Omicron
EPI_ISL_10309296	2022-02-02	RHD XV	Omicron
EPI_ISL_10309240	2022-02-02	RHD XV	Omicron
EPI_ISL_12931434	2022-02-03	North	Omicron
EPI_ISL_12780420	2022-02-03	Northeast	Omicron
EPI_ISL_10309012	2022-02-03	RHD XV	Omicron
EPI_ISL_10309521	2022-02-03	RHD XV	Omicron
EPI_ISL_10309373	2022-02-03	RHD XV	Omicron
EPI_ISL_10309016	2022-02-03	RHD XV	Omicron
EPI_ISL_10309281	2022-02-03	RHD XV	Omicron
EPI_ISL_10309250	2022-02-03	RHD XV	Omicron
EPI_ISL_10309065	2022-02-03	RHD XV	Omicron
EPI_ISL_10309029	2022-02-03	RHD XV	Omicron
EPI_ISL_10309072	2022-02-03	RHD XV	Omicron
EPI_ISL_10309444	2022-02-03	RHD XV	Omicron
EPI_ISL_10309386	2022-02-03	RHD XV	Omicron
EPI_ISL_10309092	2022-02-03	RHD XV	Omicron
EPI_ISL_10309023	2022-02-03	RHD XV	Omicron
EPI_ISL_10309062	2022-02-03	RHD XV	Omicron
EPI_ISL_10309043	2022-02-03	RHD XV	Omicron
EPI_ISL_10309484	2022-02-03	RHD XV	Omicron
EPI_ISL_18620405	2022-02-03	RHD XV	Omicron
EPI_ISL_10309477	2022-02-04	RHD XV	Omicron
EPI_ISL_10309026	2022-02-04	RHD XV	Omicron
EPI_ISL_10309279	2022-02-04	RHD XV	Omicron
EPI_ISL_10309420	2022-02-04	RHD XV	Omicron
EPI_ISL_10309060	2022-02-04	RHD XV	Omicron
EPI_ISL_10309159	2022-02-04	RHD XV	Omicron
EPI_ISL_10309353	2022-02-04	RHD XV	Omicron
EPI_ISL_10309537	2022-02-04	RHD XV	Omicron
EPI_ISL_10309013	2022-02-04	RHD XV	Omicron
EPI_ISL_10309509	2022-02-04	RHD XV	Omicron
EPI_ISL_10309058	2022-02-04	RHD XV	Omicron
EPI_ISL_10309456	2022-02-04	RHD XV	Omicron
EPI_ISL_10309419	2022-02-04	RHD XV	Omicron
EPI_ISL_10309063	2022-02-04	RHD XV	Omicron
EPI_ISL_10309494	2022-02-04	RHD XV	Omicron
EPI_ISL_10309215	2022-02-04	RHD XV	Omicron
EPI_ISL_10309220	2022-02-04	RHD XV	Omicron
EPI_ISL_10309257	2022-02-04	RHD XV	Omicron
EPI_ISL_10309269	2022-02-04	RHD XV	Omicron
EPI_ISL_10309140	2022-02-04	RHD XV	Omicron
EPI_ISL_10309255	2022-02-04	RHD XV	Omicron
EPI_ISL_10309103	2022-02-04	RHD XV	Omicron
EPI_ISL_10309539	2022-02-04	RHD XV	Omicron
EPI_ISL_10309385	2022-02-05	RHD XV	Omicron
EPI_ISL_10309899	2022-02-05	RHD XV	Omicron
EPI_ISL_10309698	2022-02-05	RHD XV	Omicron
EPI_ISL_10309200	2022-02-05	RHD XV	Omicron
EPI_ISL_10309900	2022-02-05	RHD XV	Omicron
EPI_ISL_10309289	2022-02-05	RHD XV	Omicron
EPI_ISL_10309180	2022-02-05	RHD XV	Omicron
EPI_ISL_10309309	2022-02-05	RHD XV	Omicron
EPI_ISL_10309699	2022-02-05	RHD XV	Omicron
EPI_ISL_15146136	2022-02-06	North	Omicron
EPI_ISL_10309367	2022-02-06	RHD XV	Omicron
EPI_ISL_10309015	2022-02-06	RHD XV	Omicron
EPI_ISL_10309047	2022-02-06	RHD XV	Omicron
EPI_ISL_10309384	2022-02-06	RHD XV	Omicron
EPI_ISL_10309901	2022-02-06	RHD XV	Omicron
EPI_ISL_13191431	2022-02-07	North	Omicron

EPI_ISL_10309422	2022-02-07	RHD XV	Omicron
EPI_ISL_10309102	2022-02-07	RHD XV	Omicron
EPI_ISL_10309370	2022-02-07	RHD XV	Omicron
EPI_ISL_10706067	2022-02-07	Southeast	Omicron
EPI_ISL_10706083	2022-02-09	Southeast	Omicron
EPI_ISL_11002724	2022-02-16	RHD XV	Omicron
EPI_ISL_11002699	2022-02-16	RHD XV	Omicron
EPI_ISL_11002765	2022-02-16	RHD XV	Omicron
EPI_ISL_11002689	2022-02-16	RHD XV	Omicron
EPI_ISL_11002707	2022-02-16	RHD XV	Omicron
EPI_ISL_11002430	2022-02-17	RHD XV	Omicron
EPI_ISL_11002758	2022-02-17	RHD XV	Omicron
EPI_ISL_11002704	2022-02-17	RHD XV	Omicron
EPI_ISL_11002683	2022-02-17	RHD XV	Omicron
EPI_ISL_11002700	2022-02-17	RHD XV	Omicron
EPI_ISL_11002710	2022-02-17	RHD XV	Omicron
EPI_ISL_11002775	2022-02-17	RHD XV	Omicron
EPI_ISL_11002695	2022-02-17	RHD XV	Omicron
EPI_ISL_11002763	2022-02-17	RHD XV	Omicron
EPI_ISL_11002711	2022-02-17	RHD XV	Omicron
EPI_ISL_11002395	2022-02-17	RHD XV	Omicron
EPI_ISL_11002757	2022-02-17	RHD XV	Omicron
EPI_ISL_11002694	2022-02-17	RHD XV	Omicron
EPI_ISL_11002717	2022-02-17	RHD XV	Omicron
EPI_ISL_11002697	2022-02-17	RHD XV	Omicron
EPI_ISL_11002732	2022-02-17	RHD XV	Omicron
EPI_ISL_11002728	2022-02-17	RHD XV	Omicron
EPI_ISL_11002737	2022-02-17	RHD XV	Omicron
EPI_ISL_11002702	2022-02-17	RHD XV	Omicron
EPI_ISL_11002692	2022-02-17	RHD XV	Omicron
EPI_ISL_11002722	2022-02-17	RHD XV	Omicron
EPI_ISL_11002762	2022-02-18	RHD XV	Omicron
EPI_ISL_11002770	2022-02-18	RHD XV	Omicron
EPI_ISL_11002714	2022-02-18	RHD XV	Omicron
EPI_ISL_11002693	2022-02-18	RHD XV	Omicron
EPI_ISL_11002709	2022-02-18	RHD XV	Omicron
EPI_ISL_11002706	2022-02-18	RHD XV	Omicron
EPI_ISL_11002713	2022-02-18	RHD XV	Omicron
EPI_ISL_11002690	2022-02-18	RHD XV	Omicron
EPI_ISL_11002721	2022-02-18	RHD XV	Omicron
EPI_ISL_11002705	2022-02-18	RHD XV	Omicron
EPI_ISL_11002715	2022-02-18	RHD XV	Omicron
EPI_ISL_11002691	2022-02-18	RHD XV	Omicron
EPI_ISL_11002688	2022-02-18	RHD XV	Omicron
EPI_ISL_11002684	2022-02-18	RHD XV	Omicron
EPI_ISL_11002733	2022-02-18	RHD XV	Omicron
EPI_ISL_11002407	2022-02-18	RHD XV	Omicron
EPI_ISL_11002767	2022-02-18	RHD XV	Omicron
EPI_ISL_11002768	2022-02-18	RHD XV	Omicron
EPI_ISL_11002725	2022-02-18	RHD XV	Omicron
EPI_ISL_11002753	2022-02-18	RHD XV	Omicron
EPI_ISL_11002774	2022-02-19	RHD XV	Omicron
EPI_ISL_11002746	2022-02-19	RHD XV	Omicron
EPI_ISL_11002696	2022-02-19	RHD XV	Omicron
EPI_ISL_11002698	2022-02-19	RHD XV	Omicron
EPI_ISL_11002687	2022-02-19	RHD XV	Omicron
EPI_ISL_11002771	2022-02-19	RHD XV	Omicron
EPI_ISL_11002759	2022-02-19	RHD XV	Omicron
EPI_ISL_14498244	2022-02-20	Northeast	Gamma
EPI_ISL_11027301	2022-02-20	RHD XV	Omicron
EPI_ISL_11027781	2022-02-20	RHD XV	Omicron
EPI_ISL_11027833	2022-02-20	RHD XV	Omicron
EPI_ISL_11027267	2022-02-20	RHD XV	Omicron

EPI_ISL_11027268	2022-02-20	RHD XV	Omicron
EPI_ISL_11027768	2022-02-20	RHD XV	Omicron
EPI_ISL_11027306	2022-02-20	RHD XV	Omicron
EPI_ISL_11027307	2022-02-20	RHD XV	Omicron
EPI_ISL_11027871	2022-02-20	RHD XV	Omicron
EPI_ISL_11027269	2022-02-20	RHD XV	Omicron
EPI_ISL_11027821	2022-02-20	RHD XV	Omicron
EPI_ISL_11027839	2022-02-20	RHD XV	Omicron
EPI_ISL_11027642	2022-02-20	RHD XV	Omicron
EPI_ISL_11027308	2022-02-20	RHD XV	Omicron
EPI_ISL_13947745	2022-02-21	Northeast	Delta
EPI_ISL_11027648	2022-02-21	RHD XV	Omicron
EPI_ISL_11027297	2022-02-21	RHD XV	Omicron
EPI_ISL_11027298	2022-02-21	RHD XV	Omicron
EPI_ISL_11027884	2022-02-21	RHD XV	Omicron
EPI_ISL_11027299	2022-02-21	RHD XV	Omicron
EPI_ISL_11027300	2022-02-21	RHD XV	Omicron
EPI_ISL_11027641	2022-02-21	RHD XV	Omicron
EPI_ISL_11027842	2022-02-21	RHD XV	Omicron
EPI_ISL_11027303	2022-02-21	RHD XV	Omicron
EPI_ISL_11027304	2022-02-21	RHD XV	Omicron
EPI_ISL_11027775	2022-02-21	RHD XV	Omicron
EPI_ISL_11027782	2022-02-21	RHD XV	Omicron
EPI_ISL_11027690	2022-02-21	RHD XV	Omicron
EPI_ISL_11027843	2022-02-21	RHD XV	Omicron
EPI_ISL_11027469	2022-02-21	RHD XV	Omicron
EPI_ISL_11027494	2022-02-22	RHD XV	Omicron
EPI_ISL_11027889	2022-02-22	RHD XV	Omicron
EPI_ISL_11027439	2022-02-22	RHD XV	Omicron
EPI_ISL_11027428	2022-02-22	RHD XV	Omicron
EPI_ISL_11027851	2022-02-23	RHD XV	Omicron
EPI_ISL_11133082	2022-02-23	Southeast	Omicron
EPI_ISL_11027246	2022-02-24	RHD XV	Omicron
EPI_ISL_11027631	2022-02-24	RHD XV	Omicron
EPI_ISL_11027940	2022-02-24	RHD XV	Omicron
EPI_ISL_11027472	2022-02-24	RHD XV	Omicron
EPI_ISL_11027826	2022-02-24	RHD XV	Omicron
EPI_ISL_11027475	2022-02-24	RHD XV	Omicron
EPI_ISL_11027471	2022-02-24	RHD XV	Omicron
EPI_ISL_11027785	2022-02-24	RHD XV	Omicron
EPI_ISL_11027779	2022-02-24	RHD XV	Omicron
EPI_ISL_11027482	2022-02-24	RHD XV	Omicron
EPI_ISL_11027773	2022-02-24	RHD XV	Omicron
EPI_ISL_11027470	2022-02-24	RHD XV	Omicron
EPI_ISL_11027777	2022-02-24	RHD XV	Omicron
EPI_ISL_11027783	2022-02-24	RHD XV	Omicron
EPI_ISL_11027621	2022-02-24	RHD XV	Omicron
EPI_ISL_11027798	2022-02-24	RHD XV	Omicron
EPI_ISL_11027473	2022-02-24	RHD XV	Omicron
EPI_ISL_11027479	2022-02-24	RHD XV	Omicron
EPI_ISL_11027666	2022-02-24	RHD XV	Omicron
EPI_ISL_11027845	2022-02-24	RHD XV	Omicron
EPI_ISL_11027801	2022-02-24	RHD XV	Omicron
EPI_ISL_11027766	2022-02-24	RHD XV	Omicron
EPI_ISL_11027477	2022-02-24	RHD XV	Omicron
EPI_ISL_11027784	2022-02-24	RHD XV	Omicron
EPI_ISL_11027474	2022-02-24	RHD XV	Omicron
EPI_ISL_11027847	2022-02-24	RHD XV	Omicron
EPI_ISL_11027778	2022-02-24	RHD XV	Omicron
EPI_ISL_11027905	2022-02-24	RHD XV	Omicron
EPI_ISL_11027453	2022-02-24	RHD XV	Omicron
EPI_ISL_11027478	2022-02-24	RHD XV	Omicron
EPI_ISL_11027774	2022-02-25	RHD XV	Omicron

EPI_ISL_11027796	2022-02-25	RHD XV	Omicron
EPI_ISL_11027647	2022-02-25	RHD XV	Omicron
EPI_ISL_11027772	2022-02-25	RHD XV	Omicron
EPI_ISL_11027442	2022-02-25	RHD XV	Omicron
EPI_ISL_11027305	2022-02-25	RHD XV	Omicron
EPI_ISL_11027302	2022-02-26	RHD XV	Omicron
EPI_ISL_11115455	2022-02-27	RHD XV	Omicron
EPI_ISL_11115460	2022-02-27	RHD XV	Omicron
EPI_ISL_11115474	2022-02-27	RHD XV	Omicron
EPI_ISL_11115476	2022-02-27	RHD XV	Omicron
EPI_ISL_11115477	2022-02-27	RHD XV	Omicron
EPI_ISL_11115453	2022-02-28	RHD XV	Omicron
EPI_ISL_11115454	2022-02-28	RHD XV	Omicron
EPI_ISL_11115470	2022-02-28	RHD XV	Omicron
EPI_ISL_11115473	2022-02-28	RHD XV	Omicron
EPI_ISL_12173790	2022-03-01	Northeast	Omicron
EPI_ISL_11115452	2022-03-01	RHD XV	Omicron
EPI_ISL_11115459	2022-03-01	RHD XV	Omicron
EPI_ISL_11115471	2022-03-01	RHD XV	Omicron
EPI_ISL_11115432	2022-03-02	RHD XV	Omicron
EPI_ISL_11115437	2022-03-02	RHD XV	Omicron
EPI_ISL_11115438	2022-03-02	RHD XV	Omicron
EPI_ISL_11115440	2022-03-02	RHD XV	Omicron
EPI_ISL_11115442	2022-03-02	RHD XV	Omicron
EPI_ISL_11115456	2022-03-02	RHD XV	Omicron
EPI_ISL_11115461	2022-03-02	RHD XV	Omicron
EPI_ISL_11115462	2022-03-02	RHD XV	Omicron
EPI_ISL_11115463	2022-03-02	RHD XV	Omicron
EPI_ISL_11115464	2022-03-02	RHD XV	Omicron
EPI_ISL_11115465	2022-03-02	RHD XV	Omicron
EPI_ISL_11115472	2022-03-02	RHD XV	Omicron
EPI_ISL_11115475	2022-03-02	RHD XV	Omicron
EPI_ISL_11115478	2022-03-02	RHD XV	Omicron
EPI_ISL_11115449	2022-03-03	RHD XV	Omicron
EPI_ISL_11115450	2022-03-03	RHD XV	Omicron
EPI_ISL_11115451	2022-03-03	RHD XV	Omicron
EPI_ISL_11115431	2022-03-04	RHD XV	Omicron
EPI_ISL_11115435	2022-03-04	RHD XV	Omicron
EPI_ISL_11115436	2022-03-04	RHD XV	Omicron
EPI_ISL_11115444	2022-03-04	RHD XV	Omicron
EPI_ISL_11115445	2022-03-04	RHD XV	Omicron
EPI_ISL_11115446	2022-03-04	RHD XV	Omicron
EPI_ISL_11115447	2022-03-04	RHD XV	Omicron
EPI_ISL_11115448	2022-03-04	RHD XV	Omicron
EPI_ISL_11115458	2022-03-04	RHD XV	Omicron
EPI_ISL_11115468	2022-03-04	RHD XV	Omicron
EPI_ISL_11115469	2022-03-04	RHD XV	Omicron
EPI_ISL_12173796	2022-03-05	Northeast	Omicron
EPI_ISL_11115441	2022-03-05	RHD XV	Omicron
EPI_ISL_11115457	2022-03-05	RHD XV	Omicron
EPI_ISL_11115466	2022-03-05	RHD XV	Omicron
EPI_ISL_11412077	2022-03-06	RHD XV	Omicron
EPI_ISL_11412111	2022-03-06	RHD XV	Omicron
EPI_ISL_11412076	2022-03-07	RHD XV	Omicron
EPI_ISL_11412078	2022-03-07	RHD XV	Omicron
EPI_ISL_11412089	2022-03-07	RHD XV	Omicron
EPI_ISL_11412092	2022-03-07	RHD XV	Omicron
EPI_ISL_11412096	2022-03-07	RHD XV	Omicron
EPI_ISL_11412097	2022-03-07	RHD XV	Omicron
EPI_ISL_11412098	2022-03-07	RHD XV	Omicron
EPI_ISL_11412110	2022-03-07	RHD XV	Omicron
EPI_ISL_11412112	2022-03-07	RHD XV	Omicron
EPI_ISL_11412114	2022-03-07	RHD XV	Omicron

EPI_ISL_11412120	2022-03-07	RHD XV	Omicron
EPI_ISL_11412132	2022-03-07	RHD XV	Omicron
EPI_ISL_11412133	2022-03-07	RHD XV	Omicron
EPI_ISL_11412134	2022-03-07	RHD XV	Omicron
EPI_ISL_11412138	2022-03-07	RHD XV	Omicron
EPI_ISL_11412141	2022-03-07	RHD XV	Omicron
EPI_ISL_12042531	2022-03-07	Southeast	Omicron
EPI_ISL_13907934	2022-03-08	Northeast	Omicron
EPI_ISL_11412107	2022-03-08	RHD XV	Omicron
EPI_ISL_11412108	2022-03-08	RHD XV	Omicron
EPI_ISL_11412109	2022-03-08	RHD XV	Omicron
EPI_ISL_11412139	2022-03-08	RHD XV	Omicron
EPI_ISL_11412140	2022-03-08	RHD XV	Omicron
EPI_ISL_11412142	2022-03-08	RHD XV	Omicron
EPI_ISL_11412143	2022-03-08	RHD XV	Omicron
EPI_ISL_11412144	2022-03-08	RHD XV	Omicron
EPI_ISL_11412145	2022-03-08	RHD XV	Omicron
EPI_ISL_11412088	2022-03-09	RHD XV	Omicron
EPI_ISL_11412093	2022-03-09	RHD XV	Omicron
EPI_ISL_11412094	2022-03-09	RHD XV	Omicron
EPI_ISL_11412095	2022-03-09	RHD XV	Omicron
EPI_ISL_11412099	2022-03-09	RHD XV	Omicron
EPI_ISL_11412100	2022-03-09	RHD XV	Omicron
EPI_ISL_11412101	2022-03-09	RHD XV	Omicron
EPI_ISL_11412102	2022-03-09	RHD XV	Omicron
EPI_ISL_11412103	2022-03-09	RHD XV	Omicron
EPI_ISL_11412104	2022-03-09	RHD XV	Omicron
EPI_ISL_11412105	2022-03-09	RHD XV	Omicron
EPI_ISL_11412123	2022-03-09	RHD XV	Omicron
EPI_ISL_11412124	2022-03-09	RHD XV	Omicron
EPI_ISL_11412125	2022-03-09	RHD XV	Omicron
EPI_ISL_11412126	2022-03-09	RHD XV	Omicron
EPI_ISL_11412130	2022-03-09	RHD XV	Omicron
EPI_ISL_11412131	2022-03-09	RHD XV	Omicron
EPI_ISL_11412135	2022-03-09	RHD XV	Omicron
EPI_ISL_11412136	2022-03-09	RHD XV	Omicron
EPI_ISL_11412137	2022-03-09	RHD XV	Omicron
EPI_ISL_12862768	2022-03-09	South	Omicron
EPI_ISL_12173799	2022-03-10	Northeast	Omicron
EPI_ISL_11412087	2022-03-10	RHD XV	Omicron
EPI_ISL_11412090	2022-03-11	RHD XV	Omicron
EPI_ISL_11412091	2022-03-11	RHD XV	Omicron
EPI_ISL_11412127	2022-03-11	RHD XV	Omicron
EPI_ISL_11412128	2022-03-11	RHD XV	Omicron
EPI_ISL_11412129	2022-03-11	RHD XV	Omicron
EPI_ISL_11412079	2022-03-12	RHD XV	Omicron
EPI_ISL_11412080	2022-03-12	RHD XV	Omicron
EPI_ISL_11412081	2022-03-12	RHD XV	Omicron
EPI_ISL_11412082	2022-03-12	RHD XV	Omicron
EPI_ISL_11412083	2022-03-12	RHD XV	Omicron
EPI_ISL_11412084	2022-03-12	RHD XV	Omicron
EPI_ISL_11412085	2022-03-12	RHD XV	Omicron
EPI_ISL_11412086	2022-03-12	RHD XV	Omicron
EPI_ISL_11412113	2022-03-12	RHD XV	Omicron
EPI_ISL_11412115	2022-03-12	RHD XV	Omicron
EPI_ISL_11412116	2022-03-12	RHD XV	Omicron
EPI_ISL_11412117	2022-03-12	RHD XV	Omicron
EPI_ISL_11412118	2022-03-12	RHD XV	Omicron
EPI_ISL_11412119	2022-03-12	RHD XV	Omicron
EPI_ISL_11412121	2022-03-12	RHD XV	Omicron
EPI_ISL_11412122	2022-03-12	RHD XV	Omicron
EPI_ISL_11763898	2022-03-14	RHD XV	Omicron
EPI_ISL_11763899	2022-03-14	RHD XV	Omicron

EPI_ISL_11763900	2022-03-14	RHD XV	Omicron
EPI_ISL_11763904	2022-03-14	RHD XV	Omicron
EPI_ISL_11763905	2022-03-14	RHD XV	Omicron
EPI_ISL_11763906	2022-03-14	RHD XV	Omicron
EPI_ISL_11763910	2022-03-14	RHD XV	Omicron
EPI_ISL_11763892	2022-03-15	RHD XV	Omicron
EPI_ISL_11763893	2022-03-15	RHD XV	Omicron
EPI_ISL_11763894	2022-03-15	RHD XV	Omicron
EPI_ISL_11763895	2022-03-15	RHD XV	Omicron
EPI_ISL_11763896	2022-03-15	RHD XV	Omicron
EPI_ISL_11763897	2022-03-15	RHD XV	Omicron
EPI_ISL_11763901	2022-03-15	RHD XV	Omicron
EPI_ISL_11763903	2022-03-15	RHD XV	Omicron
EPI_ISL_12690896	2022-03-16	North	Omicron
EPI_ISL_11763891	2022-03-16	RHD XV	Omicron
EPI_ISL_11763907	2022-03-16	RHD XV	Omicron
EPI_ISL_11763909	2022-03-16	RHD XV	Omicron
EPI_ISL_11763888	2022-03-17	RHD XV	Omicron
EPI_ISL_11763889	2022-03-17	RHD XV	Omicron
EPI_ISL_11763902	2022-03-17	RHD XV	Omicron
EPI_ISL_11763884	2022-03-19	RHD XV	Omicron
EPI_ISL_11763885	2022-03-19	RHD XV	Omicron
EPI_ISL_11763886	2022-03-19	RHD XV	Omicron
EPI_ISL_11763887	2022-03-19	RHD XV	Omicron
EPI_ISL_11763908	2022-03-19	RHD XV	Omicron
EPI_ISL_11814950	2022-03-20	RHD XV	Omicron
EPI_ISL_11814907	2022-03-21	RHD XV	Omicron
EPI_ISL_11814927	2022-03-21	RHD XV	Omicron
EPI_ISL_11814928	2022-03-21	RHD XV	Omicron
EPI_ISL_11814929	2022-03-21	RHD XV	Omicron
EPI_ISL_11814948	2022-03-21	RHD XV	Omicron
EPI_ISL_11814949	2022-03-21	RHD XV	Omicron
EPI_ISL_11814977	2022-03-21	RHD XV	Omicron
EPI_ISL_11814995	2022-03-21	RHD XV	Omicron
EPI_ISL_11814996	2022-03-21	RHD XV	Omicron
EPI_ISL_11815005	2022-03-22	RHD XV	Omicron
EPI_ISL_11814990	2022-03-23	RHD XV	Omicron
EPI_ISL_11815004	2022-03-23	RHD XV	Omicron
EPI_ISL_11815020	2022-03-23	RHD XV	Omicron
EPI_ISL_11815021	2022-03-23	RHD XV	Omicron
EPI_ISL_11814997	2022-03-24	RHD XV	Omicron
EPI_ISL_11814999	2022-03-24	RHD XV	Omicron
EPI_ISL_11815000	2022-03-24	RHD XV	Omicron
EPI_ISL_11815001	2022-03-24	RHD XV	Omicron
EPI_ISL_11815002	2022-03-24	RHD XV	Omicron
EPI_ISL_11815003	2022-03-24	RHD XV	Omicron
EPI_ISL_15039295	2022-03-25	Southeast	Omicron
EPI_ISL_11814993	2022-03-26	RHD XV	Omicron
EPI_ISL_11814994	2022-03-26	RHD XV	Omicron
EPI_ISL_15039306	2022-04-11	Southeast	Omicron
EPI_ISL_15464844	2022-04-13	North	Omicron
EPI_ISL_15464848	2022-04-19	North	Omicron
EPI_ISL_15456464	2022-04-20	South	Omicron
EPI_ISL_15039305	2022-04-23	Southeast	Omicron
EPI_ISL_15039301	2022-04-25	Southeast	Omicron
EPI_ISL_15455261	2022-04-25	Southeast	Omicron
EPI_ISL_15456461	2022-04-25	South	Others
EPI_ISL_15455262	2022-04-28	Southeast	Omicron

Supplementary Table 2

Supplementary Table 3. Virus exchange events among RHD XV and all the Brazilian regions, and between RHD XV and São Jo

GISAID Access Number	Origin	Destination	Collection Date
EPI_ISL_9630673	North	North	2021-11-04
EPI_ISL_9630677	Northeast	North	2021-11-08
EPI_ISL_9630748	North	North	2021-11-13
EPI_ISL_9630685	North	North	2021-11-15
EPI_ISL_9630688	North	North	2021-11-17
EPI_ISL_9630692	North	North	2021-11-18
EPI_ISL_9630749	North	North	2021-11-18
EPI_ISL_9630697	South	North	2021-12-09
EPI_ISL_9630744	North	North	2021-12-13
EPI_ISL_9630704	North	North	2021-12-15
EPI_ISL_9630746	North	North	2021-12-17
EPI_ISL_9630747	North	North	2021-12-20
EPI_ISL_9630710	SJdRP	North	2021-12-21
EPI_ISL_11124707	North	North	2022-01-04
EPI_ISL_11124712	North	North	2022-01-05
EPI_ISL_13287026	North	North	2021-06-10
EPI_ISL_13287028	North	North	2021-10-06
EPI_ISL_13287029	North	North	2021-10-06
EPI_ISL_13287030	North	North	2021-10-07
EPI_ISL_13287036	North	North	2021-10-08
EPI_ISL_13287039	North	North	2021-10-10
EPI_ISL_13287040	North	North	2021-10-11
EPI_ISL_13287042	North	North	2021-10-13
EPI_ISL_13287045	North	North	2021-10-14
EPI_ISL_13287027	South	North	2021-10-14
EPI_ISL_2612324	North	North	2021-01-24
EPI_ISL_2612325	North	North	2021-02-05
EPI_ISL_2612330	North	North	2021-02-09
EPI_ISL_2612343	RHD XV	North	2021-04-10
EPI_ISL_12931434	North	North	2022-02-03
EPI_ISL_13191431	North	North	2022-02-07
EPI_ISL_5926861	Northeast	North	2021-04-24
EPI_ISL_5926862	South	North	2021-04-24
EPI_ISL_5926864	North	North	2021-05-05
EPI_ISL_5926866	Northeast	North	2021-05-28
EPI_ISL_5926868	North	North	2021-06-03
EPI_ISL_5926869	North	North	2021-06-03
EPI_ISL_5926872	North	North	2021-05-07
EPI_ISL_5926877	North	North	2021-05-20
EPI_ISL_5926880	Northeast	North	2021-05-19
EPI_ISL_5926883	North	North	2021-05-25
EPI_ISL_5926885	RHD XV	North	2021-07-27
EPI_ISL_5926887	North	North	2021-07-23
EPI_ISL_5926890	Northeast	North	2021-07-19
EPI_ISL_5926894	North	North	2021-06-03
EPI_ISL_5926896	North	North	2021-05-21
EPI_ISL_5926897	North	North	2021-07-29
EPI_ISL_2612347	North	North	2021-01-05
EPI_ISL_2612348	North	North	2021-01-05
EPI_ISL_2612351	North	North	2021-01-08
EPI_ISL_2612355	North	North	2021-01-15
EPI_ISL_2612358	North	North	2021-01-17
EPI_ISL_2612360	Southeast	North	2021-01-19
EPI_ISL_2612361	North	North	2021-01-23
EPI_ISL_2612365	North	North	2021-02-25
EPI_ISL_2612366	Northeast	North	2021-02-25
EPI_ISL_2612372	North	North	2021-03-08
EPI_ISL_2612377	North	North	2021-03-19
EPI_ISL_2612378	Northeast	North	2021-03-19
EPI_ISL_2612379	North	North	2021-03-23

EPI_ISL_2612391	North	North	2021-04-12
EPI_ISL_2612392	North	North	2021-04-12
EPI_ISL_2612407	North	North	2021-01-15
EPI_ISL_2612408	North	North	2021-03-10
EPI_ISL_2308423	Northeast	Northeast	2021-01-01
EPI_ISL_2308461	Northeast	Northeast	2021-01-04
EPI_ISL_2308441	Northeast	Northeast	2021-01-31
EPI_ISL_2645728	Northeast	Northeast	2021-02-15
EPI_ISL_2157412	Northeast	Northeast	2021-03-04
EPI_ISL_2157451	Northeast	Northeast	2021-03-10
EPI_ISL_2157490	Northeast	Northeast	2021-03-16
EPI_ISL_2157508	Northeast	Northeast	2021-03-21
EPI_ISL_2157519	Northeast	Northeast	2021-03-29
EPI_ISL_2157520	Northeast	Northeast	2021-03-29
EPI_ISL_5825590	Northeast	Northeast	2021-08-26
EPI_ISL_2157541	Northeast	Northeast	2021-04-17
EPI_ISL_6229756	Northeast	Northeast	2021-08-09
EPI_ISL_6229759	Northeast	Northeast	2021-08-04
EPI_ISL_2274054	Northeast	Northeast	2021-04-08
EPI_ISL_6229679	Northeast	Northeast	2021-09-03
EPI_ISL_6229637	Northeast	Northeast	2021-02-23
EPI_ISL_2466246	Northeast	Northeast	2021-04-24
EPI_ISL_2466232	Northeast	Northeast	2021-04-30
EPI_ISL_8057715	Southeast	Northeast	2021-09-25
EPI_ISL_8057730	Northeast	Northeast	2021-09-29
EPI_ISL_2557319	Northeast	Northeast	2021-05-01
EPI_ISL_2557320	Northeast	Northeast	2021-05-04
EPI_ISL_2557322	Northeast	Northeast	2021-05-05
EPI_ISL_2645390	Northeast	Northeast	2021-05-13
EPI_ISL_2645402	RHD XV	Northeast	2021-05-22
EPI_ISL_2645404	Northeast	Northeast	2021-05-24
EPI_ISL_3434811	Northeast	Northeast	2021-01-07
EPI_ISL_2983075	Northeast	Northeast	2021-06-05
EPI_ISL_3190310	Northeast	Northeast	2021-06-12
EPI_ISL_3190322	Northeast	Northeast	2021-06-20
EPI_ISL_3190330	Northeast	Northeast	2021-06-25
EPI_ISL_3190333	Northeast	Northeast	2021-06-26
EPI_ISL_3434835	Northeast	Northeast	2021-07-03
EPI_ISL_3434838	Northeast	Northeast	2021-07-05
EPI_ISL_3434943	North	Northeast	2021-07-13
EPI_ISL_2645655	Northeast	Northeast	2021-01-24
EPI_ISL_3802930	Northeast	Northeast	2021-07-18
EPI_ISL_4061401	Northeast	Northeast	2021-07-29
EPI_ISL_4061420	Northeast	Northeast	2021-08-02
EPI_ISL_2645660	Northeast	Northeast	2021-01-27
EPI_ISL_2645669	Northeast	Northeast	2021-02-02
EPI_ISL_2645688	Northeast	Northeast	2021-02-11
EPI_ISL_2645697	Northeast	Northeast	2021-02-12
EPI_ISL_2645711	North	Northeast	2021-02-18
EPI_ISL_5254446	Northeast	Northeast	2021-08-14
EPI_ISL_6573743	Northeast	Northeast	2021-10-05
EPI_ISL_6573677	Northeast	Northeast	2021-10-06
EPI_ISL_6573648	Northeast	Northeast	2021-10-17
EPI_ISL_6573715	Northeast	Northeast	2021-10-18
EPI_ISL_6810977	SJdRP	Northeast	2021-10-27
EPI_ISL_8466930	Northeast	Northeast	2021-11-30
EPI_ISL_10901894	Northeast	Northeast	2021-12-02
EPI_ISL_13907934	RHD XV	Northeast	2022-03-08
EPI_ISL_940626	North	North	2021-01-21
EPI_ISL_2777405	North	North	2021-01-15
EPI_ISL_2777441	North	North	2021-05-17
EPI_ISL_1068259	North	North	2021-01-04
EPI_ISL_1068261	North	North	2021-01-05

EPI_ISL_1068271	North	North	2021-01-11
EPI_ISL_1068275	North	North	2021-01-08
EPI_ISL_2777473	North	North	2021-01-14
EPI_ISL_2777484	Midwest	North	2021-02-03
EPI_ISL_2777495	North	North	2021-01-01
EPI_ISL_2777507	North	North	2021-01-28
EPI_ISL_2777508	North	North	2021-02-02
EPI_ISL_2777509	North	North	2021-01-31
EPI_ISL_3050353	North	North	2021-06-09
EPI_ISL_3050433	North	North	2021-06-16
EPI_ISL_2777544	North	North	2021-03-02
EPI_ISL_2777923	North	North	2021-05-09
EPI_ISL_2777919	North	North	2021-05-08
EPI_ISL_2777609	North	North	2021-04-01
EPI_ISL_2777612	Northeast	North	2021-05-03
EPI_ISL_2777620	North	North	2021-03-13
EPI_ISL_2777942	North	North	2021-05-27
EPI_ISL_3050332	Northeast	North	2021-06-05
EPI_ISL_2777650	North	North	2021-05-14
EPI_ISL_2777662	North	North	2021-04-03
EPI_ISL_4516234	North	North	2021-09-02
EPI_ISL_3050593	North	North	2021-06-30
EPI_ISL_3050605	Northeast	North	2021-07-01
EPI_ISL_2777698	North	North	2021-02-01
EPI_ISL_2777699	North	North	2021-04-09
EPI_ISL_4520384	North	Northeast	2021-07-02
EPI_ISL_2777703	Northeast	North	2021-05-02
EPI_ISL_2777713	North	North	2021-03-18
EPI_ISL_3050312	North	North	2021-06-01
EPI_ISL_2777719	North	North	2021-02-10
EPI_ISL_4520349	North	North	2021-07-15
EPI_ISL_2777728	North	North	2021-02-17
EPI_ISL_2777758	Northeast	North	2021-02-25
EPI_ISL_4516028	Northeast	North	2021-08-26
EPI_ISL_2777795	RHD XV	North	2021-03-25
EPI_ISL_4515995	North	North	2021-09-04
EPI_ISL_2777799	North	North	2021-03-10
EPI_ISL_2777815	Southeast	North	2021-04-12
EPI_ISL_4516015	North	North	2021-09-11
EPI_ISL_4516199	North	North	2021-08-28
EPI_ISL_4516241	North	North	2021-09-03
EPI_ISL_2777961	North	North	2021-05-21
EPI_ISL_4520317	Northeast	North	2021-07-07
EPI_ISL_2777874	Northeast	North	2021-04-19
EPI_ISL_2777881	North	North	2021-04-28
EPI_ISL_3050514	North	North	2021-06-21
EPI_ISL_4520308	North	North	2021-07-06
EPI_ISL_8630319	North	North	2021-11-26
EPI_ISL_4030335	Midwest	North	2021-04-23
EPI_ISL_5487823	North	North	2021-08-03
EPI_ISL_5488685	North	North	2021-07-21
EPI_ISL_5489417	North	North	2021-09-19
EPI_ISL_5490831	North	North	2021-10-01
EPI_ISL_5490846	North	North	2021-08-16
EPI_ISL_5490905	North	North	2021-09-23
EPI_ISL_5490974	North	North	2021-07-28
EPI_ISL_5491028	North	North	2021-07-27
EPI_ISL_5621176	Northeast	North	2021-10-05
EPI_ISL_6100864	North	North	2021-08-09
EPI_ISL_6100871	North	North	2021-08-18
EPI_ISL_6494876	North	North	2021-10-21
EPI_ISL_8056203	North	North	2021-10-17
EPI_ISL_8056237	North	North	2021-11-01

EPI_ISL_8056268	North	North	2021-11-04
EPI_ISL_8056305	North	North	2021-11-06
EPI_ISL_8056345	North	North	2021-11-10
EPI_ISL_8056375	North	North	2021-11-13
EPI_ISL_8056376	North	North	2021-11-13
EPI_ISL_8056400	North	North	2021-11-16
EPI_ISL_8056539	North	North	2021-12-01
EPI_ISL_8353171	North	North	2021-09-16
EPI_ISL_8353175	Southeast	North	2021-09-24
EPI_ISL_8515157	North	North	2021-12-08
EPI_ISL_8515172	North	North	2021-12-12
EPI_ISL_8554953	North	North	2021-12-30
EPI_ISL_9194769	North	North	2022-01-03
EPI_ISL_9416869	RHD XV	North	2022-01-13
EPI_ISL_9500047	North	North	2022-01-21
EPI_ISL_13164663	North	North	2021-10-08
EPI_ISL_13164679	North	North	2021-10-20
EPI_ISL_11815915	North	North	2021-10-31
EPI_ISL_11815919	North	North	2021-11-20
EPI_ISL_12838678	North	North	2021-12-26
EPI_ISL_3149999	North	North	2021-03-03
EPI_ISL_3150016	North	North	2021-02-26
EPI_ISL_2241547	North	North	2021-02-10
EPI_ISL_2983087	North	North	2021-01-03
EPI_ISL_2983089	North	North	2021-01-08
EPI_ISL_2983122	North	North	2021-01-18
EPI_ISL_2983129	North	North	2021-01-30
EPI_ISL_2983132	North	North	2021-03-13
EPI_ISL_2983163	North	North	2021-02-28
EPI_ISL_2983165	North	North	2021-03-01
EPI_ISL_2983169	North	North	2021-03-03
EPI_ISL_2983172	North	North	2021-03-06
EPI_ISL_3434898	North	North	2021-06-13
EPI_ISL_3434957	North	North	2021-06-17
EPI_ISL_3434959	North	North	2021-06-20
EPI_ISL_3539747	North	North	2021-06-27
EPI_ISL_3434968	North	North	2021-06-30
EPI_ISL_3434970	Northeast	North	2021-07-04
EPI_ISL_3434974	Midwest	North	2021-07-06
EPI_ISL_3539796	North	North	2021-07-12
EPI_ISL_3539814	North	North	2021-07-17
EPI_ISL_3539749	Northeast	North	2021-07-22
EPI_ISL_4081047	North	North	2021-07-31
EPI_ISL_4081042	North	North	2021-08-04
EPI_ISL_4081030	North	North	2021-08-09
EPI_ISL_4345455	North	North	2021-08-16
EPI_ISL_4418862	North	North	2021-08-25
EPI_ISL_6940560	North	North	2021-09-06
EPI_ISL_6940561	South	North	2021-09-03
EPI_ISL_6940563	North	North	2021-09-04
EPI_ISL_6898380	Northeast	North	2021-09-29
EPI_ISL_6898381	North	North	2021-10-03
EPI_ISL_6772675	North	North	2021-10-14
EPI_ISL_8325061	North	North	2021-11-16
EPI_ISL_9671431	North	North	2021-12-07
EPI_ISL_6721723	South	North	2021-09-10
EPI_ISL_6721745	SJdRP	North	2021-09-23
EPI_ISL_2245073	North	North	2021-01-02
EPI_ISL_2298790	North	North	2021-01-11
EPI_ISL_2298793	North	North	2021-01-16
EPI_ISL_2298797	North	North	2021-01-19
EPI_ISL_2298821	North	North	2021-02-19
EPI_ISL_11396740	North	North	2021-01-31

EPI_ISL_11396741	South	North	2021-02-03
EPI_ISL_11396742	North	North	2021-02-07
EPI_ISL_11396746	North	North	2021-02-09
EPI_ISL_15458350	North	North	2021-04-11
EPI_ISL_2491695	South	Northeast	2021-01-19
EPI_ISL_2491772	Northeast	Northeast	2021-01-05
EPI_ISL_4474543	Northeast	Northeast	2021-09-10
EPI_ISL_2491774	Northeast	Northeast	2021-01-22
EPI_ISL_2491713	Northeast	Northeast	2021-01-25
EPI_ISL_8633917	South	Northeast	2021-12-22
EPI_ISL_2491700	Northeast	Northeast	2021-02-11
EPI_ISL_14498244	Northeast	Northeast	2022-02-20
EPI_ISL_12402106	Northeast	Northeast	2021-02-02
EPI_ISL_2663260	Northeast	Northeast	2021-02-22
EPI_ISL_2663295	Northeast	Northeast	2021-03-08
EPI_ISL_2663297	Northeast	Northeast	2021-03-10
EPI_ISL_12402087	Northeast	Northeast	2021-03-19
EPI_ISL_2663308	North	Northeast	2021-03-30
EPI_ISL_6908396	Northeast	Northeast	2021-04-10
EPI_ISL_6908434	Northeast	Northeast	2021-04-22
EPI_ISL_6908848	North	Northeast	2021-04-29
EPI_ISL_6908891	RHD XV	Northeast	2021-05-12
EPI_ISL_8127599	Northeast	Northeast	2021-05-19
EPI_ISL_8127633	Northeast	Northeast	2021-05-24
EPI_ISL_8127686	Northeast	Northeast	2021-06-09
EPI_ISL_6908032	Northeast	Northeast	2021-06-30
EPI_ISL_10101939	Northeast	Northeast	2021-09-11
EPI_ISL_6907060	Northeast	Northeast	2021-08-23
EPI_ISL_6907016	Northeast	Northeast	2021-08-17
EPI_ISL_10101776	North	Northeast	2021-10-14
EPI_ISL_6908622	Northeast	Northeast	2021-08-27
EPI_ISL_6908601	Northeast	Northeast	2021-08-26
EPI_ISL_6908542	Northeast	Northeast	2021-08-21
EPI_ISL_6908699	Northeast	Northeast	2021-08-01
EPI_ISL_6941441	Northeast	Northeast	2021-10-16
EPI_ISL_8127903	North	Northeast	2021-11-23
EPI_ISL_8127894	Southeast	Northeast	2021-11-20
EPI_ISL_8127883	Southeast	Northeast	2021-11-15
EPI_ISL_12573107	Northeast	Northeast	2022-01-24
EPI_ISL_12780400	RHD XV	Northeast	2022-01-27
EPI_ISL_12173790	SJdRP	Northeast	2022-03-01
EPI_ISL_12173796	RHD XV	Northeast	2022-03-05
EPI_ISL_12571031	RHD XV	Northeast	2022-01-08
EPI_ISL_12571071	Northeast	Northeast	2022-01-09
EPI_ISL_1608166	Northeast	Northeast	2021-02-17
EPI_ISL_3102216	Northeast	Northeast	2021-01-21
EPI_ISL_3102239	Northeast	Northeast	2021-01-25
EPI_ISL_3102240	Northeast	Northeast	2021-02-17
EPI_ISL_3102368	Northeast	Northeast	2021-03-26
EPI_ISL_3102397	RHD XV	Northeast	2021-06-06
EPI_ISL_3102467	Northeast	Northeast	2021-02-21
EPI_ISL_3102513	Southeast	Northeast	2021-04-23
EPI_ISL_3102522	Northeast	Northeast	2021-05-22
EPI_ISL_3102531	Northeast	Northeast	2021-03-28
EPI_ISL_3102535	Northeast	Northeast	2021-04-10
EPI_ISL_3536175	Northeast	Northeast	2021-07-01
EPI_ISL_3536205	Northeast	Northeast	2021-06-09
EPI_ISL_3536223	RHD XV	Northeast	2021-06-15
EPI_ISL_3536348	Northeast	Northeast	2021-07-06
EPI_ISL_3536174	Northeast	Northeast	2021-07-08
EPI_ISL_3536324	Northeast	Northeast	2021-06-05
EPI_ISL_3536326	Northeast	Northeast	2021-06-01
EPI_ISL_3536368	North	Northeast	2021-07-16

EPI_ISL_3869215	Southeast	Northeast	2021-01-20
EPI_ISL_3912433	Northeast	Northeast	2021-01-15
EPI_ISL_3912399	Northeast	Northeast	2021-08-02
EPI_ISL_3912201	Northeast	Northeast	2021-07-27
EPI_ISL_3912197	North	Northeast	2021-04-06
EPI_ISL_3912176	Northeast	Northeast	2021-04-14
EPI_ISL_3912419	Northeast	Northeast	2021-01-30
EPI_ISL_3912254	Northeast	Northeast	2021-02-02
EPI_ISL_3912448	Northeast	Northeast	2021-02-03
EPI_ISL_3912321	Northeast	Northeast	2021-02-23
EPI_ISL_5529981	Northeast	Northeast	2021-02-28
EPI_ISL_5529991	Northeast	Northeast	2021-03-02
EPI_ISL_5529998	Northeast	Northeast	2021-03-04
EPI_ISL_5603196	Northeast	Northeast	2021-08-22
EPI_ISL_6945663	Midwest	Northeast	2021-08-20
EPI_ISL_6945652	South	Northeast	2021-08-24
EPI_ISL_5825576	Northeast	Northeast	2021-09-02
EPI_ISL_6945647	Northeast	Northeast	2021-08-31
EPI_ISL_5825579	Northeast	Northeast	2021-09-01
EPI_ISL_6474547	Northeast	Northeast	2021-09-25
EPI_ISL_6474039	Northeast	Northeast	2021-09-14
EPI_ISL_6474139	North	Northeast	2021-09-28
EPI_ISL_6474576	Northeast	Northeast	2021-10-03
EPI_ISL_6474515	Northeast	Northeast	2021-09-10
EPI_ISL_8318160	Northeast	Northeast	2021-10-05
EPI_ISL_8747987	Northeast	Northeast	2021-10-28
EPI_ISL_8748033	Northeast	Northeast	2021-11-02
EPI_ISL_8748034	Northeast	Northeast	2021-11-03
EPI_ISL_8748053	North	Northeast	2021-11-13
EPI_ISL_9793936	Northeast	Northeast	2021-11-28
EPI_ISL_9794026	South	Northeast	2021-12-01
EPI_ISL_9794029	Northeast	Northeast	2021-11-29
EPI_ISL_9794043	Northeast	Northeast	2021-12-02
EPI_ISL_9794151	North	Northeast	2021-12-08
EPI_ISL_9794189	SJdRP	Northeast	2021-12-13
EPI_ISL_9215991	Southeast	Northeast	2021-12-20
EPI_ISL_9216000	Southeast	Northeast	2021-12-23
EPI_ISL_2661912	Northeast	Northeast	2021-01-19
EPI_ISL_2801327	Northeast	Northeast	2021-03-19
EPI_ISL_2801357	Northeast	Northeast	2021-05-03
EPI_ISL_2298739	Northeast	Northeast	2021-01-07
EPI_ISL_15463432	Northeast	Northeast	2021-12-25
EPI_ISL_14307715	Northeast	Southeast	2021-03-11
EPI_ISL_2645518	Southeast	Southeast	2021-03-13
EPI_ISL_2645519	Southeast	Southeast	2021-03-21
EPI_ISL_2196262	Northeast	Southeast	2021-04-28
EPI_ISL_3045462	Midwest	Southeast	2021-05-20
EPI_ISL_3190244	RHD XV	Southeast	2021-06-22
EPI_ISL_3190246	Southeast	Southeast	2021-06-25
EPI_ISL_3190250	Southeast	Southeast	2021-07-03
EPI_ISL_3435015	Northeast	Southeast	2021-07-10
EPI_ISL_3801884	Southeast	Southeast	2021-07-20
EPI_ISL_3827994	RHD XV	Southeast	2021-06-14
EPI_ISL_3828011	Southeast	Southeast	2021-06-21
EPI_ISL_3827996	Northeast	Southeast	2021-07-25
EPI_ISL_4220167	Southeast	Southeast	2021-08-01
EPI_ISL_4220171	SJdRP	Southeast	2021-08-02
EPI_ISL_4220193	Southeast	Southeast	2021-08-07
EPI_ISL_4220240	Southeast	Southeast	2021-08-15
EPI_ISL_4212763	Southeast	Southeast	2021-08-22
EPI_ISL_4212861	Southeast	Southeast	2021-08-21
EPI_ISL_4633269	Southeast	Southeast	2021-08-24
EPI_ISL_4728673	Southeast	Southeast	2021-08-28

EPI_ISL_4728681	Southeast	Southeast	2021-09-02
EPI_ISL_4728783	Southeast	Southeast	2021-09-07
EPI_ISL_5390609	Southeast	Southeast	2021-09-14
EPI_ISL_7103189	South	Southeast	2021-10-07
EPI_ISL_6173207	Southeast	Southeast	2021-10-09
EPI_ISL_6173232	Southeast	Southeast	2021-10-14
EPI_ISL_8013041	Southeast	Southeast	2021-10-18
EPI_ISL_8332048	Southeast	Southeast	2021-11-07
EPI_ISL_8325312	Southeast	Southeast	2021-09-27
EPI_ISL_8325320	Southeast	Southeast	2021-10-29
EPI_ISL_8325340	Southeast	Southeast	2021-11-18
EPI_ISL_8325323	North	Southeast	2021-11-19
EPI_ISL_11330950	North	Southeast	2021-12-16
EPI_ISL_11330951	Southeast	Southeast	2021-12-18
EPI_ISL_2645594	Northeast	Southeast	2021-01-03
EPI_ISL_2645607	Southeast	Southeast	2021-01-15
EPI_ISL_2645608	South	Southeast	2021-01-10
EPI_ISL_1239134	Southeast	Southeast	2021-01-14
EPI_ISL_13833556	RHD XV	Southeast	2021-05-26
EPI_ISL_14702173	Northeast	Southeast	2021-09-24
EPI_ISL_13833618	Southeast	Southeast	2021-12-05
EPI_ISL_13833621	Northeast	Southeast	2021-12-11
EPI_ISL_13833667	North	Southeast	2022-01-22
EPI_ISL_15604599	SJdRP	Southeast	2022-01-04
EPI_ISL_15604608	SJdRP	Southeast	2022-01-07
EPI_ISL_985317	Midwest	Midwest	2021-01-19
EPI_ISL_2614542	Northeast	Midwest	2021-01-25
EPI_ISL_2828704	South	Midwest	2021-02-13
EPI_ISL_2691210	North	Midwest	2021-05-15
EPI_ISL_2919228	Midwest	Midwest	2021-04-02
EPI_ISL_2919230	South	Midwest	2021-04-03
EPI_ISL_9521259	Midwest	Midwest	2022-01-10
EPI_ISL_3841319	Northeast	Midwest	2021-07-30
EPI_ISL_2820233	Midwest	Midwest	2021-02-19
EPI_ISL_3190350	Midwest	Midwest	2021-05-20
EPI_ISL_4513324	North	Midwest	2021-08-27
EPI_ISL_8266269	Midwest	Midwest	2021-10-24
EPI_ISL_8266267	Northeast	Midwest	2021-10-25
EPI_ISL_8325195	Midwest	Midwest	2021-11-05
EPI_ISL_8325212	Northeast	Midwest	2021-12-13
EPI_ISL_11016285	Northeast	Midwest	2021-11-15
EPI_ISL_2017318	Midwest	Midwest	2021-01-01
EPI_ISL_2017309	Midwest	Midwest	2021-01-05
EPI_ISL_2187709	South	Midwest	2021-01-12
EPI_ISL_2187714	Midwest	Midwest	2021-01-13
EPI_ISL_2017298	North	Midwest	2021-01-15
EPI_ISL_2187732	Northeast	Midwest	2021-01-16
EPI_ISL_2348597	Midwest	Midwest	2021-01-26
EPI_ISL_2187755	Midwest	Midwest	2021-02-01
EPI_ISL_2017288	Midwest	Midwest	2021-02-03
EPI_ISL_2187764	Midwest	Midwest	2021-02-04
EPI_ISL_3254337	Southeast	Midwest	2021-02-08
EPI_ISL_2222881	Northeast	Midwest	2021-02-09
EPI_ISL_2187783	Midwest	Midwest	2021-02-11
EPI_ISL_2017468	Midwest	Midwest	2021-02-18
EPI_ISL_2017304	Midwest	Midwest	2021-02-22
EPI_ISL_2187824	Midwest	Midwest	2021-02-21
EPI_ISL_3254345	South	Midwest	2021-03-03
EPI_ISL_2187829	Midwest	Midwest	2021-03-02
EPI_ISL_2017286	Northeast	Midwest	2021-03-05
EPI_ISL_2187876	Midwest	Midwest	2021-03-10
EPI_ISL_2017428	Midwest	Midwest	2021-03-23
EPI_ISL_2017477	Southeast	Midwest	2021-03-29

EPI_ISL_2017396	South	Midwest	2021-03-31
EPI_ISL_2187948	Midwest	Midwest	2021-04-05
EPI_ISL_2017336	Northeast	Midwest	2021-04-14
EPI_ISL_2017430	RHD XV	Midwest	2021-04-26
EPI_ISL_2017434	North	Midwest	2021-04-27
EPI_ISL_2497439	North	Midwest	2021-04-30
EPI_ISL_2187992	RHD XV	Midwest	2021-05-03
EPI_ISL_2188023	Northeast	Midwest	2021-05-05
EPI_ISL_2348607	SJdRP	Midwest	2021-05-07
EPI_ISL_2348615	Northeast	Midwest	2021-05-13
EPI_ISL_2466451	South	Midwest	2021-05-17
EPI_ISL_2497457	RHD XV	Midwest	2021-05-25
EPI_ISL_3254386	North	Midwest	2021-06-01
EPI_ISL_3254413	Northeast	Midwest	2021-06-04
EPI_ISL_2617624	Midwest	Midwest	2021-06-08
EPI_ISL_3254437	Midwest	Midwest	2021-06-09
EPI_ISL_3254519	Northeast	Midwest	2021-06-15
EPI_ISL_3254590	Midwest	Midwest	2021-06-26
EPI_ISL_4061673	Midwest	Midwest	2021-07-02
EPI_ISL_3254669	Midwest	Midwest	2021-07-08
EPI_ISL_3274747	Southeast	Midwest	2021-07-09
EPI_ISL_3259358	North	Midwest	2021-07-16
EPI_ISL_3386131	Midwest	Midwest	2021-08-02
EPI_ISL_3536441	Northeast	Midwest	2021-08-03
EPI_ISL_3536479	RHD XV	Midwest	2021-08-05
EPI_ISL_3758092	South	Midwest	2021-08-10
EPI_ISL_3536478	South	Midwest	2021-07-31
EPI_ISL_3922238	Northeast	Midwest	2021-08-23
EPI_ISL_3997066	Northeast	Midwest	2021-08-30
EPI_ISL_4212701	Midwest	Midwest	2021-09-06
EPI_ISL_4212693	RHD XV	Midwest	2021-09-08
EPI_ISL_4889933	Midwest	Midwest	2021-09-20
EPI_ISL_9568493	Midwest	Midwest	2021-11-20
EPI_ISL_9568500	Northeast	Midwest	2021-11-29
EPI_ISL_9568511	North	Midwest	2021-12-05
EPI_ISL_9568518	Midwest	Midwest	2021-12-21
EPI_ISL_9568618	North	Midwest	2021-12-30
EPI_ISL_9568619	SJdRP	Midwest	2022-01-09
EPI_ISL_9527750	South	Midwest	2021-10-29
EPI_ISL_9527761	Southeast	Midwest	2021-09-26
EPI_ISL_1465215	Northeast	Northeast	2021-02-03
EPI_ISL_1465250	Northeast	Northeast	2021-02-15
EPI_ISL_1465255	Northeast	Northeast	2021-02-06
EPI_ISL_1465208	Northeast	Northeast	2021-02-07
EPI_ISL_1465201	Northeast	Northeast	2021-02-12
EPI_ISL_1465191	Northeast	Northeast	2021-02-23
EPI_ISL_1465219	Northeast	Northeast	2021-01-23
EPI_ISL_1465259	Northeast	Northeast	2021-01-22
EPI_ISL_1465261	Northeast	Northeast	2021-01-20
EPI_ISL_1465186	Northeast	Northeast	2021-01-19
EPI_ISL_1465274	South	Northeast	2021-01-03
EPI_ISL_6208347	Northeast	Northeast	2021-01-11
EPI_ISL_1465268	Northeast	Northeast	2021-01-11
EPI_ISL_1465267	Northeast	Northeast	2021-01-12
EPI_ISL_2196263	South	Northeast	2021-03-10
EPI_ISL_2196265	Northeast	Northeast	2021-02-26
EPI_ISL_2196268	South	Northeast	2021-03-19
EPI_ISL_2443556	Northeast	Northeast	2021-03-04
EPI_ISL_2274089	Northeast	Northeast	2021-03-05
EPI_ISL_2274090	North	Northeast	2021-03-10
EPI_ISL_2274083	Northeast	Northeast	2021-03-12
EPI_ISL_2274085	Northeast	Northeast	2021-03-01
EPI_ISL_2274077	Northeast	Northeast	2021-03-09

EPI_ISL_2274081	Northeast	Northeast	2021-03-26
EPI_ISL_2274082	Northeast	Northeast	2021-03-02
EPI_ISL_2645414	Northeast	Northeast	2021-05-16
EPI_ISL_2645418	Northeast	Northeast	2021-05-16
EPI_ISL_2983211	Northeast	Northeast	2021-04-09
EPI_ISL_2983258	Northeast	Northeast	2021-04-11
EPI_ISL_2983261	Northeast	Northeast	2021-04-14
EPI_ISL_2983265	Northeast	Northeast	2021-04-16
EPI_ISL_2983268	Northeast	Northeast	2021-04-20
EPI_ISL_2983274	Northeast	Northeast	2021-04-20
EPI_ISL_2983276	Southeast	Northeast	2021-04-23
EPI_ISL_2983280	Northeast	Northeast	2021-04-02
EPI_ISL_2983285	Northeast	Northeast	2021-04-26
EPI_ISL_2983290	Northeast	Northeast	2021-05-01
EPI_ISL_2983295	Northeast	Northeast	2021-05-04
EPI_ISL_2983296	Northeast	Northeast	2021-05-04
EPI_ISL_2983297	Northeast	Northeast	2021-05-08
EPI_ISL_2983299	Northeast	Northeast	2021-05-09
EPI_ISL_2983304	Northeast	Northeast	2021-04-22
EPI_ISL_3434756	Northeast	Northeast	2021-06-14
EPI_ISL_6173117	Northeast	Northeast	2021-07-12
EPI_ISL_4880287	Northeast	Northeast	2021-07-30
EPI_ISL_6173108	Northeast	Northeast	2021-08-08
EPI_ISL_6172442	Northeast	Northeast	2021-08-28
EPI_ISL_6172400	Northeast	Northeast	2021-09-21
EPI_ISL_1181414	Northeast	Northeast	2021-01-01
EPI_ISL_1181413	Northeast	Northeast	2021-01-03
EPI_ISL_3828018	Northeast	Northeast	2021-01-04
EPI_ISL_1181401	Northeast	Northeast	2021-01-15
EPI_ISL_12140068	Northeast	Northeast	2021-05-26
EPI_ISL_2248779	Northeast	Northeast	2021-01-01
EPI_ISL_2298750	Northeast	Northeast	2021-01-04
EPI_ISL_2298752	Northeast	Northeast	2021-01-07
EPI_ISL_2298772	Northeast	Northeast	2021-02-01
EPI_ISL_2298778	Northeast	Northeast	2021-02-09
EPI_ISL_2298836	Northeast	Northeast	2021-02-20
EPI_ISL_11406416	Northeast	Northeast	2021-05-26
EPI_ISL_11468167	Northeast	Northeast	2021-06-07
EPI_ISL_10706067	RHD XV	Southeast	2022-02-07
EPI_ISL_10706083	SJdRP	Southeast	2022-02-09
EPI_ISL_5195467	Southeast	Southeast	2021-07-26
EPI_ISL_12042531	SJdRP	Southeast	2022-03-07
EPI_ISL_5195473	RHD XV	Southeast	2021-06-22
EPI_ISL_5195477	Southeast	Southeast	2021-06-28
EPI_ISL_5195478	North	Southeast	2021-07-06
EPI_ISL_5195483	Southeast	Southeast	2021-07-01
EPI_ISL_5195485	North	Southeast	2021-07-09
EPI_ISL_5195490	RHD XV	Southeast	2021-07-14
EPI_ISL_5195507	Northeast	Southeast	2021-08-11
EPI_ISL_7132914	Southeast	Southeast	2021-08-25
EPI_ISL_7132996	RHD XV	Southeast	2021-08-24
EPI_ISL_7132998	Southeast	Southeast	2021-09-03
EPI_ISL_8630210	Southeast	Southeast	2021-09-10
EPI_ISL_8630217	Southeast	Southeast	2021-09-14
EPI_ISL_8630240	Southeast	Southeast	2021-09-22
EPI_ISL_3031302	North	Southeast	2021-03-20
EPI_ISL_8630269	Southeast	Southeast	2021-10-13
EPI_ISL_8630287	Southeast	Southeast	2021-10-22
EPI_ISL_8630301	North	Southeast	2021-11-08
EPI_ISL_8630303	Southeast	Southeast	2021-11-05
EPI_ISL_3061855	Southeast	Southeast	2021-02-25
EPI_ISL_8630318	Southeast	Southeast	2021-11-26
EPI_ISL_8630322	Southeast	Southeast	2021-12-01

EPI_ISL_8630335	Southeast	Southeast	2021-03-29
EPI_ISL_8630344	Southeast	Southeast	2021-05-17
EPI_ISL_8630348	Southeast	Southeast	2021-05-14
EPI_ISL_3031316	Southeast	Southeast	2021-03-09
EPI_ISL_9274957	Southeast	Southeast	2021-12-07
EPI_ISL_9274958	RHD XV	Southeast	2021-12-10
EPI_ISL_9274972	Southeast	Southeast	2021-12-22
EPI_ISL_9274978	RHD XV	Southeast	2021-12-23
EPI_ISL_9275005	SJdRP	Southeast	2022-01-03
EPI_ISL_10706103	Southeast	Southeast	2022-01-17
EPI_ISL_2551530	Northeast	Southeast	2021-04-07
EPI_ISL_1182555	South	Southeast	2021-01-30
EPI_ISL_7796051	North	Southeast	2021-12-06
EPI_ISL_1239124	Southeast	Southeast	2021-01-05
EPI_ISL_1133272	Southeast	Southeast	2021-01-13
EPI_ISL_1494975	South	Southeast	2021-02-09
EPI_ISL_1494977	North	Southeast	2021-02-26
EPI_ISL_1495026	Southeast	Southeast	2021-03-03
EPI_ISL_1495041	Northeast	Southeast	2021-02-01
EPI_ISL_1133265	Southeast	Southeast	2021-01-11
EPI_ISL_3155972	Southeast	Southeast	2021-03-08
EPI_ISL_6508478	Midwest	Southeast	2021-03-24
EPI_ISL_6508480	Southeast	Southeast	2021-03-26
EPI_ISL_15039295	SJdRP	Southeast	2022-03-25
EPI_ISL_3553533	Southeast	Southeast	2021-04-04
EPI_ISL_1358309	South	Midwest	2021-02-03
EPI_ISL_2756488	Southeast	Midwest	2021-03-22
EPI_ISL_3671913	Northeast	Midwest	2021-03-07
EPI_ISL_3691387	Midwest	Midwest	2021-03-27
EPI_ISL_3691386	Midwest	Midwest	2021-04-01
EPI_ISL_3545811	South	Midwest	2021-05-11
EPI_ISL_3545812	Northeast	Midwest	2021-05-09
EPI_ISL_2274091	Midwest	Midwest	2021-02-26
EPI_ISL_9800143	RHD XV	Midwest	2021-07-04
EPI_ISL_9800154	RHD XV	Midwest	2021-07-09
EPI_ISL_9800071	Midwest	Midwest	2021-07-10
EPI_ISL_9800174	Midwest	Midwest	2021-07-19
EPI_ISL_9800263	RHD XV	Midwest	2021-07-31
EPI_ISL_9800123	RHD XV	Midwest	2021-08-02
EPI_ISL_9800102	RHD XV	Midwest	2021-08-06
EPI_ISL_9800105	Midwest	Midwest	2021-08-13
EPI_ISL_9800439	Northeast	Midwest	2021-08-23
EPI_ISL_9800168	RHD XV	Midwest	2021-08-26
EPI_ISL_9800149	RHD XV	Midwest	2021-09-08
EPI_ISL_9800471	South	Midwest	2021-09-12
EPI_ISL_9800406	Midwest	Midwest	2021-09-28
EPI_ISL_9800080	SJdRP	Midwest	2021-10-21
EPI_ISL_9800331	Midwest	Midwest	2021-10-31
EPI_ISL_9800318	Midwest	Midwest	2021-11-09
EPI_ISL_9800339	Midwest	Midwest	2021-11-24
EPI_ISL_9800329	Midwest	Midwest	2021-11-25
EPI_ISL_12043471	North	Midwest	2021-12-24
EPI_ISL_3946595	South	Midwest	2021-02-08
EPI_ISL_3946598	Southeast	Midwest	2021-02-09
EPI_ISL_15456933	Midwest	Midwest	2021-12-08
EPI_ISL_15456935	RHD XV	Midwest	2021-12-15
EPI_ISL_15456936	Midwest	Midwest	2021-12-17
EPI_ISL_2958880	Northeast	Midwest	2021-01-17
EPI_ISL_2958886	North	Midwest	2021-03-23
EPI_ISL_3316173	Southeast	Midwest	2021-03-30
EPI_ISL_3316224	Midwest	Midwest	2021-04-01
EPI_ISL_3316226	Midwest	Midwest	2021-05-10
EPI_ISL_2894547	RHD XV	Midwest	2021-06-22

EPI_ISL_2896234	Northeast	Midwest	2021-06-22
EPI_ISL_2896237	North	Midwest	2021-06-18
EPI_ISL_2896239	Northeast	Midwest	2021-06-23
EPI_ISL_13131324	Midwest	Midwest	2021-07-05
EPI_ISL_13131323	Midwest	Midwest	2021-07-03
EPI_ISL_13131314	Midwest	Midwest	2021-07-31
EPI_ISL_13131315	Midwest	Midwest	2021-07-31
EPI_ISL_13131317	Midwest	Midwest	2021-08-03
EPI_ISL_13960027	North	Midwest	2021-05-03
EPI_ISL_13960034	Northeast	Midwest	2021-12-01
EPI_ISL_14670067	RHD XV	Midwest	2021-12-01
EPI_ISL_14670064	Midwest	Midwest	2021-05-24
EPI_ISL_13960035	Midwest	Midwest	2021-09-16
EPI_ISL_14674983	South	Midwest	2021-08-04
EPI_ISL_14674380	Midwest	Midwest	2021-12-20
EPI_ISL_14674348	North	Midwest	2021-12-28
EPI_ISL_14674388	Northeast	Midwest	2022-01-02
EPI_ISL_11407010	Midwest	Midwest	2021-08-26
EPI_ISL_2645887	North	North	2021-02-28
EPI_ISL_2645888	North	North	2021-03-03
EPI_ISL_2645891	North	North	2021-03-09
EPI_ISL_2645424	North	North	2021-05-04
EPI_ISL_2645427	North	North	2021-05-13
EPI_ISL_3539238	North	North	2021-07-24
EPI_ISL_3539239	North	North	2021-07-25
EPI_ISL_4061291	Southeast	North	2021-08-02
EPI_ISL_4061295	North	North	2021-08-10
EPI_ISL_4080767	North	North	2021-04-30
EPI_ISL_4632922	North	North	2021-08-23
EPI_ISL_6172470	Northeast	North	2021-09-04
EPI_ISL_6172495	Northeast	North	2021-09-07
EPI_ISL_6172483	RHD XV	North	2021-09-06
EPI_ISL_6172477	North	North	2021-09-21
EPI_ISL_5689778	North	North	2021-09-27
EPI_ISL_5689787	North	North	2021-10-01
EPI_ISL_6173257	North	North	2021-10-15
EPI_ISL_7455348	North	North	2021-10-02
EPI_ISL_7455358	RHD XV	North	2021-10-06
EPI_ISL_7455761	North	North	2021-11-01
EPI_ISL_7455762	Northeast	North	2021-11-02
EPI_ISL_8013130	RHD XV	North	2021-11-20
EPI_ISL_8266308	North	North	2021-11-26
EPI_ISL_12036871	North	North	2021-12-11
EPI_ISL_2645844	North	North	2021-01-29
EPI_ISL_2645832	North	North	2021-02-12
EPI_ISL_12140058	Northeast	North	2021-03-17
EPI_ISL_12140062	North	North	2021-04-07
EPI_ISL_6721832	North	North	2021-07-19
EPI_ISL_2298758	North	North	2021-02-06
EPI_ISL_11359367	North	North	2021-01-25
EPI_ISL_3368626	North	North	2021-03-04
EPI_ISL_3368625	North	North	2021-03-29
EPI_ISL_3356369	Northeast	North	2021-03-01
EPI_ISL_3356377	Northeast	North	2021-03-15
EPI_ISL_11403608	South	North	2021-08-12
EPI_ISL_11403541	RHD XV	North	2021-08-19
EPI_ISL_11495519	SJdRP	North	2021-09-10
EPI_ISL_1213388	Northeast	Northeast	2021-01-24
EPI_ISL_1213439	South	Northeast	2021-01-09
EPI_ISL_2241573	Northeast	Northeast	2021-01-15
EPI_ISL_2157578	South	Northeast	2021-01-12
EPI_ISL_2157591	Northeast	Northeast	2021-03-02
EPI_ISL_2157380	Northeast	Northeast	2021-02-20

EPI_ISL_3061882	Northeast	Northeast	2021-04-24
EPI_ISL_3061884	Northeast	Northeast	2021-04-03
EPI_ISL_3061887	Northeast	Northeast	2021-04-10
EPI_ISL_2536327	Southeast	Northeast	2021-03-31
EPI_ISL_2536333	Southeast	Northeast	2021-03-26
EPI_ISL_2536336	Northeast	Northeast	2021-04-11
EPI_ISL_2536345	Northeast	Northeast	2021-04-09
EPI_ISL_2536305	Northeast	Northeast	2021-02-16
EPI_ISL_2536306	Northeast	Northeast	2021-02-22
EPI_ISL_2536312	Northeast	Northeast	2021-03-10
EPI_ISL_2536318	Northeast	Northeast	2021-03-12
EPI_ISL_2536323	Southeast	Northeast	2021-05-02
EPI_ISL_4220296	Northeast	Northeast	2021-08-20
EPI_ISL_4633830	Northeast	Northeast	2021-08-27
EPI_ISL_4633834	Southeast	Northeast	2021-08-23
EPI_ISL_6172907	Northeast	Northeast	2021-09-04
EPI_ISL_6172639	Northeast	Northeast	2021-09-09
EPI_ISL_6172667	Northeast	Northeast	2021-09-24
EPI_ISL_6173372	Northeast	Northeast	2021-10-04
EPI_ISL_6173378	Northeast	Northeast	2021-10-02
EPI_ISL_6898917	North	Northeast	2021-10-12
EPI_ISL_6898919	Northeast	Northeast	2021-10-17
EPI_ISL_8005701	North	Northeast	2021-11-16
EPI_ISL_8266368	Northeast	Northeast	2021-11-12
EPI_ISL_8633941	Northeast	Northeast	2021-11-29
EPI_ISL_8633949	Northeast	Northeast	2021-12-01
EPI_ISL_8633965	Northeast	Northeast	2021-12-13
EPI_ISL_8633975	Northeast	Northeast	2021-12-18
EPI_ISL_13947745	Northeast	Northeast	2022-02-21
EPI_ISL_2308416	Northeast	Northeast	2021-03-31
EPI_ISL_5530113	Northeast	Northeast	2021-04-09
EPI_ISL_1181362	Northeast	Northeast	2021-01-07
EPI_ISL_2221873	Northeast	Northeast	2021-01-10
EPI_ISL_2221885	Northeast	Northeast	2021-03-04
EPI_ISL_2821266	Northeast	Northeast	2021-01-13
EPI_ISL_2821268	Northeast	Northeast	2021-02-23
EPI_ISL_3835353	Northeast	Northeast	2021-02-24
EPI_ISL_2821274	Northeast	Northeast	2021-05-03
EPI_ISL_3134661	North	Northeast	2021-05-11
EPI_ISL_3046288	Northeast	Northeast	2021-07-01
EPI_ISL_3134705	Northeast	Northeast	2021-07-06
EPI_ISL_3703704	North	Northeast	2021-07-16
EPI_ISL_3447589	Northeast	Northeast	2021-04-30
EPI_ISL_3447590	Northeast	Northeast	2021-05-22
EPI_ISL_3703726	RHD XV	Northeast	2021-07-27
EPI_ISL_3835251	Northeast	Northeast	2021-08-02
EPI_ISL_3835286	RHD XV	Northeast	2021-08-05
EPI_ISL_4486962	Northeast	Northeast	2021-08-19
EPI_ISL_4486991	Northeast	Northeast	2021-08-10
EPI_ISL_5254347	Northeast	Northeast	2021-08-31
EPI_ISL_5254350	Northeast	Northeast	2021-09-19
EPI_ISL_5254352	Northeast	Northeast	2021-09-18
EPI_ISL_5254364	Northeast	Northeast	2021-09-22
EPI_ISL_5915336	Northeast	Northeast	2021-09-03
EPI_ISL_5915338	Northeast	Northeast	2021-10-03
EPI_ISL_6573754	Northeast	Northeast	2021-10-05
EPI_ISL_6573847	Northeast	Northeast	2021-03-01
EPI_ISL_6810912	Northeast	Northeast	2021-10-12
EPI_ISL_6810934	Southeast	Northeast	2021-10-20
EPI_ISL_8025138	Northeast	Northeast	2021-11-12
EPI_ISL_8025175	Northeast	Northeast	2021-11-23
EPI_ISL_8025226	Northeast	Northeast	2021-12-08
EPI_ISL_8466866	Northeast	Northeast	2021-11-30

EPI_ISL_8466868	Northeast	Northeast	2021-12-11
EPI_ISL_8466870	North	Northeast	2021-12-28
EPI_ISL_8708704	SJdRP	Northeast	2021-12-16
EPI_ISL_8708712	Northeast	Northeast	2022-01-04
EPI_ISL_9266661	Southeast	Northeast	2022-01-05
EPI_ISL_940613	Northeast	Northeast	2021-01-19
EPI_ISL_2241598	Northeast	Northeast	2021-02-04
EPI_ISL_2241599	Northeast	Northeast	2021-02-05
EPI_ISL_2241575	Northeast	Northeast	2021-03-10
EPI_ISL_4600555	Northeast	Northeast	2021-02-17
EPI_ISL_8055904	North	Northeast	2021-09-11
EPI_ISL_8055911	North	Northeast	2021-09-21
EPI_ISL_8055915	Northeast	Northeast	2021-09-22
EPI_ISL_4488032	Northeast	Northeast	2021-04-26
EPI_ISL_4487931	RHD XV	Northeast	2021-05-11
EPI_ISL_4488044	Northeast	Northeast	2021-03-26
EPI_ISL_4487962	Midwest	Northeast	2021-05-08
EPI_ISL_4487679	RHD XV	Northeast	2021-06-26
EPI_ISL_4206971	RHD XV	Northeast	2021-06-09
EPI_ISL_2983428	South	South	2021-05-27
EPI_ISL_3190384	South	South	2021-07-01
EPI_ISL_3801852	South	South	2021-06-26
EPI_ISL_4237179	South	South	2021-08-11
EPI_ISL_1181394	Northeast	South	2021-01-26
EPI_ISL_6172968	South	South	2021-08-30
EPI_ISL_6172970	SJdRP	South	2021-08-31
EPI_ISL_4955788	South	South	2021-09-14
EPI_ISL_7106318	Northeast	South	2021-09-22
EPI_ISL_6173403	South	South	2021-09-28
EPI_ISL_6173437	Northeast	South	2021-10-05
EPI_ISL_7104389	South	South	2021-10-07
EPI_ISL_8005225	South	South	2021-02-02
EPI_ISL_8005227	South	South	2021-02-05
EPI_ISL_8005232	North	South	2021-02-13
EPI_ISL_8005240	Southeast	South	2021-02-21
EPI_ISL_8005241	North	South	2021-02-20
EPI_ISL_8005252	South	South	2021-03-01
EPI_ISL_8005292	South	South	2021-03-20
EPI_ISL_8005302	South	South	2021-04-06
EPI_ISL_8005304	South	South	2021-04-07
EPI_ISL_8005364	South	South	2021-05-22
EPI_ISL_8005365	South	South	2021-05-25
EPI_ISL_8005380	South	South	2021-05-31
EPI_ISL_8005384	South	South	2021-06-01
EPI_ISL_8005401	SJdRP	South	2021-06-10
EPI_ISL_8005421	RHD XV	South	2021-06-30
EPI_ISL_8005440	Southeast	South	2021-07-27
EPI_ISL_8005483	South	South	2021-01-05
EPI_ISL_8005494	South	South	2021-01-30
EPI_ISL_8005495	South	South	2021-01-31
EPI_ISL_8005510	South	South	2021-03-12
EPI_ISL_8005513	South	South	2021-03-18
EPI_ISL_8005541	South	South	2021-06-24
EPI_ISL_8152435	South	South	2021-11-03
EPI_ISL_8152439	Southeast	South	2021-11-06
EPI_ISL_8152476	SJdRP	South	2021-12-01
EPI_ISL_13459805	South	South	2021-05-05
EPI_ISL_13459814	South	South	2021-10-22
EPI_ISL_13459826	South	South	2021-10-19
EPI_ISL_13459827	North	South	2021-10-12
EPI_ISL_13459840	South	South	2021-03-02
EPI_ISL_13459851	North	South	2021-11-27
EPI_ISL_13459865	South	South	2021-03-27

EPI_ISL_13690321	South	South	2021-10-06
EPI_ISL_13565059	South	South	2021-09-03
EPI_ISL_4880357	South	South	2021-02-01
EPI_ISL_4880331	South	South	2021-02-14
EPI_ISL_12862768	Southeast	South	2022-03-09
EPI_ISL_12862763	South	South	2021-11-16
EPI_ISL_12862757	South	South	2021-12-21
EPI_ISL_1182575	Northeast	South	2021-01-03
EPI_ISL_12425078	South	South	2021-05-09
EPI_ISL_12425121	North	South	2021-04-29
EPI_ISL_12425170	RHD XV	South	2021-04-05
EPI_ISL_12425177	Northeast	South	2021-03-30
EPI_ISL_12425219	Northeast	South	2021-03-14
EPI_ISL_12425257	South	South	2021-04-19
EPI_ISL_12425267	South	South	2021-04-14
EPI_ISL_12425297	Northeast	South	2021-03-28
EPI_ISL_12425799	SJdRP	South	2021-06-02
EPI_ISL_12425944	South	South	2021-07-08
EPI_ISL_12425946	South	South	2021-07-07
EPI_ISL_12425966	South	South	2021-07-14
EPI_ISL_12425983	SJdRP	South	2021-07-22
EPI_ISL_12425992	South	South	2021-07-23
EPI_ISL_12426024	North	South	2021-08-01
EPI_ISL_12426033	RHD XV	South	2021-08-02
EPI_ISL_12426042	Northeast	South	2021-08-03
EPI_ISL_12426049	Southeast	South	2021-08-04
EPI_ISL_12426127	SJdRP	South	2021-08-25
EPI_ISL_2758698	South	South	2021-01-11
EPI_ISL_2758712	South	South	2021-01-06
EPI_ISL_1213312	Southeast	Southeast	2021-01-29
EPI_ISL_16084301	Southeast	Southeast	2021-09-30
EPI_ISL_16084303	Southeast	Southeast	2021-10-01
EPI_ISL_14534233	Southeast	Southeast	2021-08-02
EPI_ISL_14534256	Southeast	Southeast	2021-08-05
EPI_ISL_2274100	RHD XV	Southeast	2021-03-07
EPI_ISL_2614134	Southeast	Southeast	2021-01-03
EPI_ISL_2614158	Northeast	Southeast	2021-01-11
EPI_ISL_2557391	RHD XV	Southeast	2021-03-13
EPI_ISL_2614360	North	Southeast	2021-03-02
EPI_ISL_2614084	Southeast	Southeast	2021-02-01
EPI_ISL_2614089	North	Southeast	2021-03-05
EPI_ISL_2614165	South	Southeast	2021-01-12
EPI_ISL_1533991	Southeast	Southeast	2021-01-26
EPI_ISL_2614074	Southeast	Southeast	2021-02-05
EPI_ISL_2614079	Northeast	Southeast	2021-02-23
EPI_ISL_2614369	Southeast	Southeast	2021-03-22
EPI_ISL_2157525	Southeast	Southeast	2021-04-06
EPI_ISL_2443580	Northeast	Southeast	2021-01-15
EPI_ISL_2196333	North	Southeast	2021-04-22
EPI_ISL_2196242	Southeast	Southeast	2021-04-20
EPI_ISL_2443606	RHD XV	Southeast	2021-04-30
EPI_ISL_6172741	Southeast	Southeast	2021-05-04
EPI_ISL_2443634	Southeast	Southeast	2021-05-10
EPI_ISL_6970988	Southeast	Southeast	2021-05-12
EPI_ISL_4237345	Midwest	Southeast	2021-05-15
EPI_ISL_4237342	Southeast	Southeast	2021-05-18
EPI_ISL_2614183	Southeast	Southeast	2021-01-21
EPI_ISL_8004636	Southeast	Southeast	2021-05-30
EPI_ISL_8004651	Southeast	Southeast	2021-06-03
EPI_ISL_2982725	Southeast	Southeast	2021-06-08
EPI_ISL_3045470	Southeast	Southeast	2021-06-11
EPI_ISL_3045505	Southeast	Southeast	2021-06-18
EPI_ISL_2982735	Southeast	Southeast	2021-06-25

EPI_ISL_8004710	Southeast	Southeast	2021-07-05
EPI_ISL_12059845	Southeast	Southeast	2021-07-07
EPI_ISL_1181393	Southeast	Southeast	2021-01-27
EPI_ISL_8004764	Northeast	Southeast	2021-07-22
EPI_ISL_8004793	Southeast	Southeast	2021-07-31
EPI_ISL_8004799	Southeast	Southeast	2021-07-30
EPI_ISL_3190272	Southeast	Southeast	2021-01-04
EPI_ISL_8004826	Southeast	Southeast	2021-08-08
EPI_ISL_2614196	Midwest	Southeast	2021-02-02
EPI_ISL_8004871	Southeast	Southeast	2021-08-14
EPI_ISL_8004876	Southeast	Southeast	2021-08-13
EPI_ISL_4880252	Southeast	Southeast	2021-08-20
EPI_ISL_8004902	Southeast	Southeast	2021-08-21
EPI_ISL_6898933	Southeast	Southeast	2021-08-31
EPI_ISL_4212779	Southeast	Southeast	2021-09-01
EPI_ISL_4220331	Southeast	Southeast	2021-08-27
EPI_ISL_4880262	Southeast	Southeast	2021-09-13
EPI_ISL_5390689	Southeast	Southeast	2021-09-20
EPI_ISL_6173027	Southeast	Southeast	2021-10-02
EPI_ISL_6173455	Northeast	Southeast	2021-10-21
EPI_ISL_6899048	Northeast	Southeast	2021-10-28
EPI_ISL_2614215	Northeast	Southeast	2021-02-10
EPI_ISL_2614310	Southeast	Southeast	2021-03-03
EPI_ISL_2614271	Northeast	Southeast	2021-02-20
EPI_ISL_2614274	North	Southeast	2021-02-21
EPI_ISL_9225768	Northeast	Southeast	2022-01-12
EPI_ISL_9225849	North	Southeast	2022-01-11
EPI_ISL_12883418	RHD XV	Southeast	2022-01-18
EPI_ISL_12883431	RHD XV	Southeast	2022-01-25
EPI_ISL_12883435	Southeast	Southeast	2022-01-13
EPI_ISL_12883430	SJdRP	Southeast	2022-01-05
EPI_ISL_1858878	Southeast	Southeast	2021-03-28
EPI_ISL_2101661	Northeast	Southeast	2021-04-08
EPI_ISL_2101670	Southeast	Southeast	2021-04-12
EPI_ISL_2385709	RHD XV	Southeast	2021-04-29
EPI_ISL_2691615	Southeast	Southeast	2021-05-24
EPI_ISL_3245340	Southeast	Southeast	2021-06-26
EPI_ISL_3014464	Southeast	Southeast	2021-07-01
EPI_ISL_3245453	Northeast	Southeast	2021-07-12
EPI_ISL_5501474	Southeast	Southeast	2021-09-03
EPI_ISL_6970485	North	Southeast	2021-10-29
EPI_ISL_6970493	North	Southeast	2021-10-31
EPI_ISL_9321075	RHD XV	Southeast	2022-01-03
EPI_ISL_2629740	Southeast	Southeast	2021-03-04
EPI_ISL_2629824	Southeast	Southeast	2021-04-19
EPI_ISL_16085136	Southeast	Southeast	2021-07-16
EPI_ISL_16085326	Southeast	Southeast	2021-09-21
EPI_ISL_1213173	Northeast	Northeast	2021-02-01
EPI_ISL_1213329	Northeast	Northeast	2021-01-28
EPI_ISL_2241508	Northeast	Northeast	2021-02-06
EPI_ISL_2466205	Northeast	Northeast	2021-04-08
EPI_ISL_4418877	Northeast	Northeast	2021-08-25
EPI_ISL_6173086	Northeast	Northeast	2021-09-03
EPI_ISL_5328913	Northeast	Northeast	2021-08-27
EPI_ISL_6943958	Northeast	Northeast	2021-09-21
EPI_ISL_6898942	Northeast	Northeast	2021-10-14
EPI_ISL_6898943	Northeast	Northeast	2021-10-15
EPI_ISL_8325350	Northeast	Northeast	2021-12-13
EPI_ISL_11016314	Southeast	Northeast	2021-12-23
EPI_ISL_15720262	Northeast	Northeast	2021-01-07
EPI_ISL_15729223	Northeast	Northeast	2021-11-17
EPI_ISL_5915287	Northeast	Northeast	2021-05-16
EPI_ISL_3190392	Northeast	Northeast	2021-06-28

EPI_ISL_11681268	Northeast	Northeast	2021-01-22
EPI_ISL_11681270	Northeast	Northeast	2021-03-18
EPI_ISL_11681274	Northeast	Northeast	2021-04-16
EPI_ISL_11681278	North	Northeast	2021-02-08
EPI_ISL_11681279	Northeast	Northeast	2021-03-08
EPI_ISL_11681283	Northeast	Northeast	2021-06-08
EPI_ISL_11681288	Northeast	Northeast	2021-08-06
EPI_ISL_2385528	Northeast	Northeast	2021-05-03
EPI_ISL_2691575	Northeast	Northeast	2021-05-25
EPI_ISL_1293053	North	North	2021-01-18
EPI_ISL_1303502	North	North	2021-01-19
EPI_ISL_1520108	Northeast	North	2021-03-02
EPI_ISL_1520109	North	North	2021-03-03
EPI_ISL_1493600	Northeast	North	2021-01-04
EPI_ISL_1493599	North	North	2021-01-05
EPI_ISL_1493595	Southeast	North	2021-01-22
EPI_ISL_1494924	North	North	2021-02-09
EPI_ISL_1493584	Southeast	North	2021-02-12
EPI_ISL_1493579	RHD XV	North	2021-02-25
EPI_ISL_1493578	North	North	2021-02-26
EPI_ISL_5658257	Northeast	North	2021-07-27
EPI_ISL_5658401	North	North	2021-07-06
EPI_ISL_5658396	North	North	2021-07-07
EPI_ISL_5658201	Southeast	North	2021-08-03
EPI_ISL_5658171	SJdRP	North	2021-08-05
EPI_ISL_5658170	North	North	2021-08-06
EPI_ISL_5658131	RHD XV	North	2021-08-18
EPI_ISL_5658094	North	North	2021-08-25
EPI_ISL_8623163	North	North	2021-10-04
EPI_ISL_8623174	North	North	2021-10-05
EPI_ISL_8623229	Midwest	North	2021-10-25
EPI_ISL_9414732	North	North	2021-11-04
EPI_ISL_9414738	North	North	2021-11-08
EPI_ISL_9414752	North	North	2021-12-27
EPI_ISL_9414753	North	North	2021-12-28
EPI_ISL_9636845	SJdRP	North	2022-01-03
EPI_ISL_9636859	North	North	2021-12-17
EPI_ISL_6840926	North	North	2021-06-08
EPI_ISL_7982770	RHD XV	North	2021-07-04
EPI_ISL_15457733	North	North	2021-12-01
EPI_ISL_5193544	North	North	2021-06-02
EPI_ISL_5193488	North	North	2021-08-02
EPI_ISL_5193578	South	North	2021-05-03
EPI_ISL_5193575	Southeast	North	2021-05-05
EPI_ISL_5193573	North	North	2021-05-06
EPI_ISL_5193512	North	North	2021-07-06
EPI_ISL_5193479	North	North	2021-08-06
EPI_ISL_5193542	North	North	2021-06-07
EPI_ISL_5193508	North	North	2021-07-08
EPI_ISL_5193568	North	North	2021-05-10
EPI_ISL_5193537	RHD XV	North	2021-06-10
EPI_ISL_5193556	Northeast	North	2021-05-20
EPI_ISL_5193492	North	North	2021-07-20
EPI_ISL_5193468	North	North	2021-08-21
EPI_ISL_5193524	North	North	2021-06-22
EPI_ISL_5193464	North	North	2021-08-25
EPI_ISL_8360696	North	North	2021-09-27
EPI_ISL_8360698	Northeast	North	2021-10-25
EPI_ISL_8360723	North	North	2021-09-30
EPI_ISL_8360705	North	North	2021-10-13
EPI_ISL_8360731	North	North	2021-10-06
EPI_ISL_11811106	North	North	2021-12-07
EPI_ISL_11811126	Midwest	North	2021-12-27

EPI_ISL_12140002	North	North	2021-03-22
EPI_ISL_12140005	North	North	2021-03-15
EPI_ISL_12140007	North	North	2021-04-26
EPI_ISL_2245108	North	North	2021-02-15
EPI_ISL_2298762	North	North	2021-01-03
EPI_ISL_2298764	North	North	2021-02-02
EPI_ISL_2298765	North	North	2021-02-01
EPI_ISL_2245111	North	North	2021-01-28
EPI_ISL_1121316	North	South	2021-01-10
EPI_ISL_2661768	RHD XV	South	2021-03-16
EPI_ISL_2661782	South	South	2021-01-01
EPI_ISL_2661794	South	South	2021-01-09
EPI_ISL_2443672	North	South	2021-03-18
EPI_ISL_2443673	South	South	2021-05-03
EPI_ISL_2603532	South	South	2021-05-28
EPI_ISL_2982786	South	South	2021-06-09
EPI_ISL_1181411	Northeast	South	2021-01-04
EPI_ISL_3235278	South	South	2021-07-10
EPI_ISL_3539915	South	South	2021-07-20
EPI_ISL_4271132	Southeast	South	2021-08-02
EPI_ISL_4271133	South	South	2021-08-06
EPI_ISL_4271136	South	South	2021-08-09
EPI_ISL_6940655	RHD XV	South	2021-09-01
EPI_ISL_6940656	South	South	2021-09-06
EPI_ISL_8004525	South	South	2021-10-01
EPI_ISL_8004528	South	South	2021-10-06
EPI_ISL_2661843	South	South	2021-02-12
EPI_ISL_2661863	Northeast	South	2021-02-10
EPI_ISL_2249357	South	South	2021-02-24
EPI_ISL_2249355	South	South	2021-01-03
EPI_ISL_2249372	South	South	2021-01-18
EPI_ISL_2139495	South	South	2021-03-10
EPI_ISL_2139496	South	South	2021-03-17
EPI_ISL_2344423	RHD XV	South	2021-01-21
EPI_ISL_15456882	South	South	2021-11-30
EPI_ISL_15456884	South	South	2021-12-01
EPI_ISL_15456886	South	South	2021-12-02
EPI_ISL_15456892	South	South	2021-12-14
EPI_ISL_3048770	South	South	2021-02-14
EPI_ISL_3048775	South	South	2021-02-25
EPI_ISL_3048793	Northeast	South	2021-04-10
EPI_ISL_3048808	South	South	2021-04-26
EPI_ISL_985318	South	South	2021-01-08
EPI_ISL_2677150	South	South	2021-02-24
EPI_ISL_5529917	South	South	2021-07-13
EPI_ISL_5529918	South	South	2021-07-30
EPI_ISL_5529920	South	South	2021-08-05
EPI_ISL_1533997	South	South	2021-02-02
EPI_ISL_1533990	South	South	2021-01-17
EPI_ISL_2677149	South	South	2021-03-14
EPI_ISL_2196250	RHD XV	South	2021-03-19
EPI_ISL_3061899	South	South	2021-05-03
EPI_ISL_2536264	South	South	2021-03-21
EPI_ISL_2536265	Southeast	South	2021-03-01
EPI_ISL_12059875	South	South	2021-06-04
EPI_ISL_2677285	South	South	2021-01-13
EPI_ISL_2677249	South	South	2021-01-02
EPI_ISL_2677250	South	South	2021-01-01
EPI_ISL_3540020	South	South	2021-07-17
EPI_ISL_4170355	South	South	2021-08-12
EPI_ISL_4237360	South	South	2021-08-20
EPI_ISL_4414912	South	South	2021-09-03
EPI_ISL_4632931	South	South	2021-09-01

EPI_ISL_5328992	South	South	2021-09-14
EPI_ISL_5329029	RHD XV	South	2021-08-24
EPI_ISL_7106139	South	South	2021-09-24
EPI_ISL_7109982	South	South	2021-09-20
EPI_ISL_6771811	South	South	2021-10-15
EPI_ISL_6771972	South	South	2021-11-03
EPI_ISL_8004440	South	South	2021-11-16
EPI_ISL_8013267	South	South	2021-10-18
EPI_ISL_8151561	South	South	2021-12-08
EPI_ISL_8266439	South	South	2021-12-13
EPI_ISL_2677097	South	South	2021-01-28
EPI_ISL_2677128	South	South	2021-02-11
EPI_ISL_2677098	South	South	2021-01-09
EPI_ISL_2677104	South	South	2021-01-23
EPI_ISL_2677121	South	South	2021-02-04
EPI_ISL_2677126	Northeast	South	2021-02-09
EPI_ISL_12060046	South	South	2022-01-14
EPI_ISL_13088660	Midwest	South	2022-01-23
EPI_ISL_2677134	North	South	2021-02-08
EPI_ISL_7744032	South	South	2021-04-03
EPI_ISL_7744025	Northeast	South	2021-04-08
EPI_ISL_8880333	South	South	2021-11-01
EPI_ISL_7803802	South	South	2021-01-21
EPI_ISL_2241609	Northeast	Northeast	2021-01-06
EPI_ISL_2241584	Northeast	Northeast	2021-01-15
EPI_ISL_2241541	North	Northeast	2021-01-20
EPI_ISL_2241555	Northeast	Northeast	2021-02-22
EPI_ISL_2308476	Northeast	Northeast	2021-02-27
EPI_ISL_2308454	Northeast	Northeast	2021-02-28
EPI_ISL_2660617	Northeast	Northeast	2021-02-23
EPI_ISL_2660627	Northeast	Northeast	2021-02-04
EPI_ISL_2157454	Northeast	Northeast	2021-03-10
EPI_ISL_2157427	Northeast	Northeast	2021-03-09
EPI_ISL_2660684	Northeast	Northeast	2021-01-07
EPI_ISL_2863625	Northeast	Northeast	2021-05-08
EPI_ISL_2863626	Northeast	Northeast	2021-05-27
EPI_ISL_2863641	North	Northeast	2021-06-12
EPI_ISL_4061453	Northeast	Northeast	2021-06-02
EPI_ISL_4212824	Northeast	Northeast	2021-07-31
EPI_ISL_4212839	Northeast	Northeast	2021-07-09
EPI_ISL_4212835	Northeast	Northeast	2021-08-05
EPI_ISL_4212838	Northeast	Northeast	2021-07-22
EPI_ISL_4474541	Northeast	Northeast	2021-08-16
EPI_ISL_5329051	North	Northeast	2021-08-31
EPI_ISL_5329012	Southeast	Northeast	2021-09-09
EPI_ISL_6971007	Northeast	Northeast	2021-09-17
EPI_ISL_6970901	Northeast	Northeast	2021-09-30
EPI_ISL_6971010	Northeast	Northeast	2021-09-13
EPI_ISL_6971024	Northeast	Northeast	2021-09-25
EPI_ISL_6173536	Northeast	Northeast	2021-09-26
EPI_ISL_6173539	Northeast	Northeast	2021-10-02
EPI_ISL_7456287	Northeast	Northeast	2021-10-13
EPI_ISL_7456292	North	Northeast	2021-10-18
EPI_ISL_7456285	South	Northeast	2021-10-11
EPI_ISL_9670608	Northeast	Northeast	2021-12-03
EPI_ISL_2660692	Northeast	Northeast	2021-01-17
EPI_ISL_1133261	Southeast	Northeast	2021-01-08
EPI_ISL_861684	Southeast	Southeast	2021-01-02
EPI_ISL_882669	South	Southeast	2021-01-11
EPI_ISL_882670	Southeast	Southeast	2021-01-12
EPI_ISL_875689	Southeast	Southeast	2021-01-15
EPI_ISL_906073	Southeast	Southeast	2021-01-19
EPI_ISL_940629	Southeast	Southeast	2021-01-25

EPI_ISL_1121324	South	Southeast	2021-02-02
EPI_ISL_1381068	Southeast	Southeast	2021-03-02
EPI_ISL_1533697	South	Southeast	2021-01-30
EPI_ISL_1533707	Northeast	Southeast	2021-02-21
EPI_ISL_1752650	Southeast	Southeast	2021-03-17
EPI_ISL_1731602	Southeast	Southeast	2021-03-23
EPI_ISL_1731604	Northeast	Southeast	2021-03-31
EPI_ISL_2614571	Southeast	Southeast	2021-04-06
EPI_ISL_2614572	Southeast	Southeast	2021-04-11
EPI_ISL_2614580	North	Southeast	2021-04-16
EPI_ISL_2614581	Northeast	Southeast	2021-04-20
EPI_ISL_2841607	North	Southeast	2021-02-22
EPI_ISL_2919268	RHD XV	Southeast	2021-06-09
EPI_ISL_2919269	Southeast	Southeast	2021-06-10
EPI_ISL_2919275	South	Southeast	2021-06-11
EPI_ISL_2919287	North	Southeast	2021-06-13
EPI_ISL_3259737	RHD XV	Southeast	2021-06-17
EPI_ISL_3259744	South	Southeast	2021-06-21
EPI_ISL_3316270	SJdRP	Southeast	2021-06-25
EPI_ISL_1795232	RHD XV	RHD XV	2021-03-23
EPI_ISL_1795233	RHD XV	RHD XV	2021-03-21
EPI_ISL_1795234	RHD XV	RHD XV	2021-03-24
EPI_ISL_1795235	RHD XV	RHD XV	2021-03-24
EPI_ISL_1795236	RHD XV	RHD XV	2021-03-24
EPI_ISL_1795237	RHD XV	RHD XV	2021-03-24
EPI_ISL_1795238	RHD XV	RHD XV	2021-03-24
EPI_ISL_1795239	RHD XV	RHD XV	2021-03-23
EPI_ISL_1795240	RHD XV	RHD XV	2021-03-24
EPI_ISL_1795241	RHD XV	RHD XV	2021-03-24
EPI_ISL_1795242	RHD XV	RHD XV	2021-03-23
EPI_ISL_1795389	RHD XV	RHD XV	2021-03-24
EPI_ISL_1795243	RHD XV	RHD XV	2021-03-23
EPI_ISL_1795244	RHD XV	RHD XV	2021-03-23
EPI_ISL_1795245	RHD XV	RHD XV	2021-03-23
EPI_ISL_1795415	Northeast	RHD XV	2021-03-23
EPI_ISL_1795246	RHD XV	RHD XV	2021-03-23
EPI_ISL_1795247	RHD XV	RHD XV	2021-03-23
EPI_ISL_1795248	RHD XV	RHD XV	2021-03-23
EPI_ISL_1795249	RHD XV	RHD XV	2021-03-23
EPI_ISL_1795250	RHD XV	RHD XV	2021-03-23
EPI_ISL_1795251	RHD XV	RHD XV	2021-03-23
EPI_ISL_1795252	RHD XV	RHD XV	2021-03-23
EPI_ISL_1795253	RHD XV	RHD XV	2021-03-23
EPI_ISL_1795254	RHD XV	RHD XV	2021-03-23
EPI_ISL_1795255	RHD XV	RHD XV	2021-03-23
EPI_ISL_1795256	RHD XV	RHD XV	2021-03-23
EPI_ISL_1795257	RHD XV	RHD XV	2021-03-23
EPI_ISL_1795258	RHD XV	RHD XV	2021-03-24
EPI_ISL_1795259	RHD XV	RHD XV	2021-03-24
EPI_ISL_1795260	South	RHD XV	2021-03-24
EPI_ISL_1795261	RHD XV	RHD XV	2021-03-24
EPI_ISL_1795262	RHD XV	RHD XV	2021-03-24
EPI_ISL_1795263	RHD XV	RHD XV	2021-03-24
EPI_ISL_1795264	RHD XV	RHD XV	2021-03-24
EPI_ISL_1795265	RHD XV	RHD XV	2021-03-24
EPI_ISL_1795266	RHD XV	RHD XV	2021-03-23
EPI_ISL_1795267	RHD XV	RHD XV	2021-03-24
EPI_ISL_1795268	RHD XV	RHD XV	2021-03-24
EPI_ISL_1795269	RHD XV	RHD XV	2021-03-23
EPI_ISL_1795270	RHD XV	RHD XV	2021-03-23
EPI_ISL_1795416	South	RHD XV	2021-03-24
EPI_ISL_1795271	RHD XV	RHD XV	2021-03-24
EPI_ISL_1795272	RHD XV	RHD XV	2021-03-23

EPI_ISL_1795079	RHD XV	RHD XV	2021-03-24
EPI_ISL_1795273	RHD XV	RHD XV	2021-03-24
EPI_ISL_1795276	RHD XV	RHD XV	2021-03-21
EPI_ISL_1795277	SJdRP	RHD XV	2021-03-22
EPI_ISL_1795278	RHD XV	RHD XV	2021-03-23
EPI_ISL_1795279	RHD XV	RHD XV	2021-03-22
EPI_ISL_1795280	RHD XV	RHD XV	2021-03-22
EPI_ISL_1795281	RHD XV	RHD XV	2021-03-22
EPI_ISL_1795282	RHD XV	RHD XV	2021-03-22
EPI_ISL_1795283	RHD XV	RHD XV	2021-03-22
EPI_ISL_1795284	RHD XV	RHD XV	2021-03-23
EPI_ISL_1795285	RHD XV	RHD XV	2021-03-22
EPI_ISL_1795286	RHD XV	RHD XV	2021-03-22
EPI_ISL_1795287	RHD XV	RHD XV	2021-03-24
EPI_ISL_1795288	RHD XV	RHD XV	2021-03-24
EPI_ISL_1795289	RHD XV	RHD XV	2021-03-24
EPI_ISL_2551534	Northeast	Southeast	2021-02-01
EPI_ISL_1464677	Northeast	Southeast	2021-03-01
EPI_ISL_1464661	Southeast	Southeast	2021-03-04
EPI_ISL_1464652	Southeast	Southeast	2021-03-08
EPI_ISL_1464650	Southeast	Southeast	2021-03-09
EPI_ISL_1464673	Southeast	Southeast	2021-03-15
EPI_ISL_4271189	South	Southeast	2021-06-07
EPI_ISL_4271185	Southeast	Southeast	2021-06-08
EPI_ISL_3835358	Southeast	Southeast	2021-08-05
EPI_ISL_5030099	Southeast	Southeast	2021-07-11
EPI_ISL_4081136	RHD XV	Southeast	2021-07-14
EPI_ISL_4081143	RHD XV	Southeast	2021-07-18
EPI_ISL_5640179	SJdRP	Southeast	2021-08-30
EPI_ISL_5640180	RHD XV	Southeast	2021-09-12
EPI_ISL_6840960	Southeast	Southeast	2021-01-14
EPI_ISL_6854924	Southeast	Southeast	2021-09-28
EPI_ISL_6854925	RHD XV	Southeast	2021-10-22
EPI_ISL_7982799	Southeast	Southeast	2021-11-17
EPI_ISL_7982803	South	Southeast	2021-11-22
EPI_ISL_7898655	North	Southeast	2021-11-23
EPI_ISL_2445526	RHD XV	RHD XV	2021-05-12
EPI_ISL_2445516	RHD XV	RHD XV	2021-05-12
EPI_ISL_2473790	RHD XV	RHD XV	2021-05-13
EPI_ISL_2473792	RHD XV	RHD XV	2021-05-13
EPI_ISL_2445508	RHD XV	RHD XV	2021-05-12
EPI_ISL_2445529	RHD XV	RHD XV	2021-05-10
EPI_ISL_2445525	RHD XV	RHD XV	2021-05-12
EPI_ISL_2445522	North	RHD XV	2021-05-12
EPI_ISL_2473788	RHD XV	RHD XV	2021-05-12
EPI_ISL_2445528	RHD XV	RHD XV	2021-05-12
EPI_ISL_2473791	RHD XV	RHD XV	2021-05-13
EPI_ISL_2445524	RHD XV	RHD XV	2021-05-12
EPI_ISL_2445543	RHD XV	RHD XV	2021-05-13
EPI_ISL_2473693	RHD XV	RHD XV	2021-05-12
EPI_ISL_2445538	RHD XV	RHD XV	2021-05-13
EPI_ISL_2445533	SJdRP	RHD XV	2021-05-12
EPI_ISL_2445523	RHD XV	RHD XV	2021-05-12
EPI_ISL_2445520	RHD XV	RHD XV	2021-05-12
EPI_ISL_2445513	RHD XV	RHD XV	2021-05-12
EPI_ISL_2445542	RHD XV	RHD XV	2021-05-13
EPI_ISL_2445531	RHD XV	RHD XV	2021-05-11
EPI_ISL_2445527	SJdRP	RHD XV	2021-05-12
EPI_ISL_2445521	RHD XV	RHD XV	2021-05-12
EPI_ISL_2445512	RHD XV	RHD XV	2021-05-12
EPI_ISL_2473787	RHD XV	RHD XV	2021-05-12
EPI_ISL_2445539	RHD XV	RHD XV	2021-05-12
EPI_ISL_2445536	RHD XV	RHD XV	2021-05-13

EPI_ISL_2445535	RHD XV	RHD XV	2021-05-13
EPI_ISL_2445519	RHD XV	RHD XV	2021-05-12
EPI_ISL_2445515	RHD XV	RHD XV	2021-05-12
EPI_ISL_2445532	RHD XV	RHD XV	2021-05-12
EPI_ISL_2473789	RHD XV	RHD XV	2021-05-12
EPI_ISL_2445514	RHD XV	RHD XV	2021-05-12
EPI_ISL_2445518	RHD XV	RHD XV	2021-05-12
EPI_ISL_2445544	RHD XV	RHD XV	2021-05-13
EPI_ISL_2445534	RHD XV	RHD XV	2021-05-12
EPI_ISL_2445537	RHD XV	RHD XV	2021-05-13
EPI_ISL_2473786	RHD XV	RHD XV	2021-05-12
EPI_ISL_2473696	RHD XV	RHD XV	2021-05-12
EPI_ISL_2445530	RHD XV	RHD XV	2021-05-10
EPI_ISL_2473718	RHD XV	RHD XV	2021-05-19
EPI_ISL_2473719	RHD XV	RHD XV	2021-05-19
EPI_ISL_2445200	RHD XV	RHD XV	2021-05-19
EPI_ISL_2473715	RHD XV	RHD XV	2021-05-19
EPI_ISL_2445213	RHD XV	RHD XV	2021-05-19
EPI_ISL_2473720	RHD XV	RHD XV	2021-05-20
EPI_ISL_2445199	RHD XV	RHD XV	2021-05-19
EPI_ISL_2445201	RHD XV	RHD XV	2021-05-19
EPI_ISL_2445214	RHD XV	RHD XV	2021-05-19
EPI_ISL_2445210	RHD XV	RHD XV	2021-05-20
EPI_ISL_2473716	RHD XV	RHD XV	2021-05-19
EPI_ISL_2445215	North	RHD XV	2021-05-19
EPI_ISL_2445209	RHD XV	RHD XV	2021-05-20
EPI_ISL_2445203	RHD XV	RHD XV	2021-05-19
EPI_ISL_2445217	RHD XV	RHD XV	2021-05-19
EPI_ISL_2445207	RHD XV	RHD XV	2021-05-19
EPI_ISL_2473818	RHD XV	RHD XV	2021-05-19
EPI_ISL_2473817	RHD XV	RHD XV	2021-05-19
EPI_ISL_2445218	RHD XV	RHD XV	2021-05-19
EPI_ISL_2445211	RHD XV	RHD XV	2021-05-20
EPI_ISL_2473723	RHD XV	RHD XV	2021-05-19
EPI_ISL_2445205	RHD XV	RHD XV	2021-05-19
EPI_ISL_2445202	RHD XV	RHD XV	2021-05-19
EPI_ISL_2473721	RHD XV	RHD XV	2021-05-19
EPI_ISL_2445208	Northeast	RHD XV	2021-05-19
EPI_ISL_2445198	RHD XV	RHD XV	2021-05-19
EPI_ISL_2445204	RHD XV	RHD XV	2021-05-19
EPI_ISL_2445212	RHD XV	RHD XV	2021-05-20
EPI_ISL_2445206	RHD XV	RHD XV	2021-05-19
EPI_ISL_2445216	RHD XV	RHD XV	2021-05-20
EPI_ISL_2473714	RHD XV	RHD XV	2021-05-19
EPI_ISL_2473717	RHD XV	RHD XV	2021-05-19
EPI_ISL_2473722	RHD XV	RHD XV	2021-05-19
EPI_ISL_2378751	RHD XV	RHD XV	NA
EPI_ISL_2378753	RHD XV	RHD XV	NA
EPI_ISL_2378754	RHD XV	RHD XV	NA
EPI_ISL_2500975	RHD XV	RHD XV	2021-05-19
EPI_ISL_2493161	RHD XV	RHD XV	2021-05-19
EPI_ISL_2493177	RHD XV	RHD XV	2021-05-19
EPI_ISL_2494337	RHD XV	RHD XV	2021-05-19
EPI_ISL_2493426	RHD XV	RHD XV	2021-05-19
EPI_ISL_2493180	RHD XV	RHD XV	2021-05-19
EPI_ISL_2493413	RHD XV	RHD XV	2021-05-19
EPI_ISL_2493405	RHD XV	RHD XV	2021-05-19
EPI_ISL_2493414	RHD XV	RHD XV	2021-05-19
EPI_ISL_2493416	RHD XV	RHD XV	2021-05-19
EPI_ISL_2493422	RHD XV	RHD XV	2021-05-19
EPI_ISL_2493408	RHD XV	RHD XV	2021-05-19
EPI_ISL_2493412	RHD XV	RHD XV	2021-05-19
EPI_ISL_2493423	RHD XV	RHD XV	2021-05-19

EPI_ISL_2493402	RHD XV	RHD XV	2021-05-19
EPI_ISL_2493429	RHD XV	RHD XV	2021-05-19
EPI_ISL_2493424	RHD XV	RHD XV	2021-05-19
EPI_ISL_2493420	RHD XV	RHD XV	2021-05-19
EPI_ISL_2493400	RHD XV	RHD XV	2021-05-19
EPI_ISL_2493406	RHD XV	RHD XV	2021-05-19
EPI_ISL_2493404	RHD XV	RHD XV	2021-05-19
EPI_ISL_2493418	RHD XV	RHD XV	2021-05-19
EPI_ISL_2493425	RHD XV	RHD XV	2021-05-19
EPI_ISL_2493409	RHD XV	RHD XV	2021-05-19
EPI_ISL_2493415	RHD XV	RHD XV	2021-05-19
EPI_ISL_2493410	RHD XV	RHD XV	2021-05-19
EPI_ISL_2493401	RHD XV	RHD XV	2021-05-19
EPI_ISL_2493421	Midwest	RHD XV	2021-05-19
EPI_ISL_2493430	RHD XV	RHD XV	2021-05-19
EPI_ISL_2493431	RHD XV	RHD XV	2021-05-19
EPI_ISL_2493417	SJdRP	RHD XV	2021-05-19
EPI_ISL_2493411	RHD XV	RHD XV	2021-05-19
EPI_ISL_2493407	RHD XV	RHD XV	2021-05-19
EPI_ISL_2493419	SJdRP	RHD XV	2021-05-19
EPI_ISL_2493663	RHD XV	RHD XV	2021-05-19
EPI_ISL_2493664	RHD XV	RHD XV	2021-05-20
EPI_ISL_2493655	RHD XV	RHD XV	2021-05-19
EPI_ISL_2493659	RHD XV	RHD XV	2021-05-20
EPI_ISL_2493661	RHD XV	RHD XV	2021-05-19
EPI_ISL_2494297	RHD XV	RHD XV	2021-05-20
EPI_ISL_2493662	RHD XV	RHD XV	2021-05-19
EPI_ISL_2493656	RHD XV	RHD XV	2021-05-19
EPI_ISL_2493658	RHD XV	RHD XV	2021-05-20
EPI_ISL_2493666	RHD XV	RHD XV	2021-05-20
EPI_ISL_2493665	RHD XV	RHD XV	2021-05-20
EPI_ISL_2378744	RHD XV	RHD XV	NA
EPI_ISL_5649576	RHD XV	RHD XV	2021-05-26
EPI_ISL_5649566	RHD XV	RHD XV	2021-05-27
EPI_ISL_5649585	RHD XV	RHD XV	2021-05-26
EPI_ISL_5649639	RHD XV	RHD XV	2021-05-27
EPI_ISL_5650052	RHD XV	RHD XV	2021-05-26
EPI_ISL_5649667	RHD XV	RHD XV	2021-05-27
EPI_ISL_5649594	RHD XV	RHD XV	2021-05-26
EPI_ISL_5649653	RHD XV	RHD XV	2021-05-26
EPI_ISL_5649579	RHD XV	RHD XV	2021-05-26
EPI_ISL_5649619	RHD XV	RHD XV	2021-05-27
EPI_ISL_5650058	RHD XV	RHD XV	2021-05-27
EPI_ISL_5649624	RHD XV	RHD XV	2021-05-26
EPI_ISL_5649586	RHD XV	RHD XV	2021-05-26
EPI_ISL_5649636	Midwest	RHD XV	2021-05-27
EPI_ISL_5650067	RHD XV	RHD XV	2021-05-26
EPI_ISL_5649584	RHD XV	RHD XV	2021-05-26
EPI_ISL_5649654	RHD XV	RHD XV	2021-05-26
EPI_ISL_5649625	RHD XV	RHD XV	2021-05-26
EPI_ISL_5650074	RHD XV	RHD XV	2021-05-26
EPI_ISL_5649567	RHD XV	RHD XV	2021-05-27
EPI_ISL_5650076	RHD XV	RHD XV	2021-05-26
EPI_ISL_5650081	RHD XV	RHD XV	2021-05-26
EPI_ISL_5649678	RHD XV	RHD XV	2021-05-26
EPI_ISL_5649609	Northeast	RHD XV	2021-05-26
EPI_ISL_5649626	RHD XV	RHD XV	2021-05-26
EPI_ISL_5649677	RHD XV	RHD XV	2021-05-26
EPI_ISL_5649666	RHD XV	RHD XV	2021-05-26
EPI_ISL_5649685	RHD XV	RHD XV	2021-05-26
EPI_ISL_5649686	RHD XV	RHD XV	2021-05-26
EPI_ISL_5649587	RHD XV	RHD XV	2021-05-27
EPI_ISL_5649687	RHD XV	RHD XV	2021-05-26

EPI_ISL_5650084	RHD XV	RHD XV	2021-05-26
EPI_ISL_5650085	RHD XV	RHD XV	2021-05-26
EPI_ISL_5649655	RHD XV	RHD XV	2021-05-26
EPI_ISL_5649621	RHD XV	RHD XV	2021-05-27
EPI_ISL_5649600	RHD XV	RHD XV	2021-05-26
EPI_ISL_5650092	RHD XV	RHD XV	2021-05-27
EPI_ISL_5650099	RHD XV	RHD XV	2021-05-26
EPI_ISL_5650107	RHD XV	RHD XV	2021-05-26
EPI_ISL_5650112	RHD XV	RHD XV	2021-05-26
EPI_ISL_5649656	RHD XV	RHD XV	2021-05-26
EPI_ISL_5649601	RHD XV	RHD XV	2021-05-26
EPI_ISL_5649602	RHD XV	RHD XV	2021-05-27
EPI_ISL_5650120	RHD XV	RHD XV	2021-05-26
EPI_ISL_5650126	RHD XV	RHD XV	2021-05-27
EPI_ISL_5650134	RHD XV	RHD XV	2021-05-26
EPI_ISL_5649645	SJdRP	RHD XV	2021-05-27
EPI_ISL_5649657	RHD XV	RHD XV	2021-05-27
EPI_ISL_5650141	RHD XV	RHD XV	2021-05-26
EPI_ISL_5650150	RHD XV	RHD XV	2021-05-26
EPI_ISL_5650157	South	RHD XV	2021-05-27
EPI_ISL_5650159	RHD XV	RHD XV	2021-05-27
EPI_ISL_5650165	RHD XV	RHD XV	2021-05-26
EPI_ISL_5650166	RHD XV	RHD XV	2021-05-26
EPI_ISL_5650167	RHD XV	RHD XV	2021-05-26
EPI_ISL_5650168	RHD XV	RHD XV	2021-05-26
EPI_ISL_5650169	RHD XV	RHD XV	2021-05-26
EPI_ISL_5650170	RHD XV	RHD XV	2021-05-26
EPI_ISL_5650171	North	RHD XV	2021-05-27
EPI_ISL_5650172	SJdRP	RHD XV	2021-05-27
EPI_ISL_5650173	RHD XV	RHD XV	2021-05-27
EPI_ISL_5650174	RHD XV	RHD XV	2021-05-26
EPI_ISL_5650175	RHD XV	RHD XV	2021-05-26
EPI_ISL_5650176	RHD XV	RHD XV	2021-05-26
EPI_ISL_5650177	RHD XV	RHD XV	2021-05-27
EPI_ISL_5649658	RHD XV	RHD XV	2021-05-26
EPI_ISL_5650178	RHD XV	RHD XV	2021-05-26
EPI_ISL_5650179	RHD XV	RHD XV	2021-05-26
EPI_ISL_5650180	RHD XV	RHD XV	2021-05-26
EPI_ISL_5650410	RHD XV	RHD XV	2021-05-26
EPI_ISL_5650181	RHD XV	RHD XV	2021-05-27
EPI_ISL_5650182	North	RHD XV	2021-05-26
EPI_ISL_5650183	RHD XV	RHD XV	2021-05-26
EPI_ISL_5650184	RHD XV	RHD XV	2021-05-26
EPI_ISL_5650185	RHD XV	RHD XV	2021-05-26
EPI_ISL_5649679	RHD XV	RHD XV	2021-05-27
EPI_ISL_5649659	RHD XV	RHD XV	2021-05-26
EPI_ISL_5650186	RHD XV	RHD XV	2021-05-27
EPI_ISL_5650187	RHD XV	RHD XV	2021-05-27
EPI_ISL_5649610	RHD XV	RHD XV	2021-05-26
EPI_ISL_5650188	RHD XV	RHD XV	2021-05-26
EPI_ISL_5650189	RHD XV	RHD XV	2021-05-26
EPI_ISL_5650190	RHD XV	RHD XV	2021-05-27
EPI_ISL_5650191	RHD XV	RHD XV	2021-05-27
EPI_ISL_5650192	RHD XV	RHD XV	2021-05-27
EPI_ISL_5649660	RHD XV	RHD XV	2021-05-26
EPI_ISL_5650193	RHD XV	RHD XV	2021-05-26
EPI_ISL_5650194	RHD XV	RHD XV	2021-05-26
EPI_ISL_5650195	RHD XV	RHD XV	2021-05-26
EPI_ISL_5650196	RHD XV	RHD XV	2021-05-26
EPI_ISL_5650197	RHD XV	RHD XV	2021-05-26
EPI_ISL_5650198	RHD XV	RHD XV	2021-05-26
EPI_ISL_5650199	RHD XV	RHD XV	2021-05-26
EPI_ISL_5650200	RHD XV	RHD XV	2021-05-26

EPI_ISL_5649607	RHD XV	RHD XV	2021-05-26
EPI_ISL_2443061	RHD XV	SJdRP	NA
EPI_ISL_2443059	SJdRP	SJdRP	2021-04-09
EPI_ISL_2443058	North	SJdRP	2021-04-09
EPI_ISL_2443065	SJdRP	SJdRP	2021-04-09
EPI_ISL_2443060	SJdRP	Southeast	2021-04-09
EPI_ISL_2443064	SJdRP	SJdRP	2021-04-09
EPI_ISL_2443063	Southeast	Southeast	2021-04-09
EPI_ISL_2443062	SJdRP	SJdRP	2021-04-09
EPI_ISL_2444191	SJdRP	SJdRP	2021-04-28
EPI_ISL_2444156	SJdRP	SJdRP	2021-04-26
EPI_ISL_2444217	SJdRP	SJdRP	2021-05-03
EPI_ISL_2444207	SJdRP	SJdRP	2021-05-02
EPI_ISL_2444176	SJdRP	SJdRP	2021-04-27
EPI_ISL_2444200	RHD XV	SJdRP	2021-05-02
EPI_ISL_2444168	SJdRP	SJdRP	2021-04-26
EPI_ISL_2444177	SJdRP	SJdRP	2021-04-27
EPI_ISL_2444158	SJdRP	SJdRP	2021-04-26
EPI_ISL_2444160	Northeast	SJdRP	2021-04-26
EPI_ISL_2444159	SJdRP	SJdRP	2021-04-26
EPI_ISL_2444215	SJdRP	SJdRP	2021-05-03
EPI_ISL_2444214	SJdRP	SJdRP	2021-05-03
EPI_ISL_2444167	RHD XV	SJdRP	2021-04-26
EPI_ISL_2444185	RHD XV	SJdRP	2021-04-27
EPI_ISL_2444153	SJdRP	SJdRP	2021-04-26
EPI_ISL_2444169	SJdRP	SJdRP	2021-04-26
EPI_ISL_2444178	SJdRP	SJdRP	2021-04-27
EPI_ISL_2444162	SJdRP	SJdRP	2021-04-26
EPI_ISL_2444166	SJdRP	SJdRP	2021-04-26
EPI_ISL_2444161	SJdRP	SJdRP	2021-04-26
EPI_ISL_2444179	SJdRP	SJdRP	2021-04-27
EPI_ISL_2444208	SJdRP	SJdRP	2021-05-02
EPI_ISL_2444171	SJdRP	SJdRP	2021-04-26
EPI_ISL_2444204	SJdRP	SJdRP	2021-05-02
EPI_ISL_2444205	SJdRP	SJdRP	2021-05-02
EPI_ISL_2444219	SJdRP	SJdRP	2021-05-03
EPI_ISL_2444190	SJdRP	SJdRP	2021-04-28
EPI_ISL_2444172	SJdRP	SJdRP	2021-04-26
EPI_ISL_2444218	SJdRP	SJdRP	2021-05-03
EPI_ISL_2444222	SJdRP	SJdRP	2021-05-03
EPI_ISL_2444163	SJdRP	SJdRP	2021-04-26
EPI_ISL_2444182	SJdRP	SJdRP	2021-04-27
EPI_ISL_2444154	RHD XV	SJdRP	2021-04-26
EPI_ISL_2444213	SJdRP	SJdRP	2021-05-03
EPI_ISL_2444180	SJdRP	SJdRP	2021-04-27
EPI_ISL_2444188	SJdRP	SJdRP	2021-04-27
EPI_ISL_2444220	SJdRP	SJdRP	2021-05-03
EPI_ISL_2455396	RHD XV	SJdRP	2021-04-27
EPI_ISL_2444165	SJdRP	SJdRP	2021-04-26
EPI_ISL_2444198	SJdRP	SJdRP	2021-05-02
EPI_ISL_2444175	SJdRP	SJdRP	2021-04-27
EPI_ISL_2444221	SJdRP	SJdRP	2021-05-03
EPI_ISL_2444216	SJdRP	SJdRP	2021-05-03
EPI_ISL_2444164	SJdRP	SJdRP	2021-04-26
EPI_ISL_2444181	SJdRP	SJdRP	2021-04-27
EPI_ISL_2444194	SJdRP	SJdRP	2021-05-02
EPI_ISL_2444209	SJdRP	SJdRP	2021-05-02
EPI_ISL_2444196	SJdRP	SJdRP	2021-05-02
EPI_ISL_2444197	SJdRP	SJdRP	2021-05-02
EPI_ISL_2455397	SJdRP	SJdRP	2021-05-02
EPI_ISL_2444201	SJdRP	SJdRP	2021-05-02
EPI_ISL_2444183	SJdRP	SJdRP	2021-04-27
EPI_ISL_2444189	SJdRP	SJdRP	2021-04-27

EPI_ISL_2444193	RHD XV	SJdRP	2021-05-02
EPI_ISL_2444187	RHD XV	SJdRP	2021-04-27
EPI_ISL_2444170	SJdRP	SJdRP	2021-04-26
EPI_ISL_2444211	SJdRP	SJdRP	2021-05-03
EPI_ISL_2444157	RHD XV	SJdRP	2021-04-26
EPI_ISL_2444206	SJdRP	SJdRP	2021-05-02
EPI_ISL_2444174	SJdRP	SJdRP	2021-04-27
EPI_ISL_2494144	RHD XV	RHD XV	2021-05-26
EPI_ISL_5650416	RHD XV	RHD XV	2021-05-31
EPI_ISL_5650415	RHD XV	RHD XV	2021-05-31
EPI_ISL_5649569	RHD XV	RHD XV	2021-05-31
EPI_ISL_5649634	RHD XV	RHD XV	2021-05-31
EPI_ISL_5649629	RHD XV	RHD XV	2021-05-31
EPI_ISL_5649603	RHD XV	RHD XV	2021-05-31
EPI_ISL_5649581	RHD XV	RHD XV	2021-05-31
EPI_ISL_5650203	RHD XV	RHD XV	2021-05-31
EPI_ISL_5649583	RHD XV	RHD XV	2021-05-31
EPI_ISL_5649571	RHD XV	RHD XV	2021-05-31
EPI_ISL_5650208	RHD XV	RHD XV	2021-05-31
EPI_ISL_5649642	RHD XV	RHD XV	2021-05-31
EPI_ISL_5649683	RHD XV	RHD XV	2021-05-31
EPI_ISL_5650216	RHD XV	RHD XV	2021-05-31
EPI_ISL_5650220	RHD XV	RHD XV	2021-05-31
EPI_ISL_5650222	RHD XV	RHD XV	2021-05-31
EPI_ISL_5650224	RHD XV	RHD XV	2021-05-31
EPI_ISL_5650226	RHD XV	RHD XV	2021-05-31
EPI_ISL_5650230	RHD XV	RHD XV	2021-05-31
EPI_ISL_5650333	RHD XV	RHD XV	2021-06-01
EPI_ISL_5650334	RHD XV	RHD XV	2021-06-01
EPI_ISL_5650335	RHD XV	RHD XV	2021-05-31
EPI_ISL_5650337	RHD XV	RHD XV	2021-05-31
EPI_ISL_5650341	RHD XV	RHD XV	2021-06-01
EPI_ISL_5650344	RHD XV	RHD XV	2021-06-01
EPI_ISL_5649589	RHD XV	RHD XV	2021-06-01
EPI_ISL_5649591	RHD XV	RHD XV	2021-05-31
EPI_ISL_5650352	RHD XV	RHD XV	2021-06-01
EPI_ISL_5650353	RHD XV	RHD XV	2021-06-01
EPI_ISL_5650356	RHD XV	RHD XV	2021-05-31
EPI_ISL_5650357	SJdRP	RHD XV	2021-06-01
EPI_ISL_5650358	RHD XV	RHD XV	2021-05-31
EPI_ISL_5649592	RHD XV	RHD XV	2021-06-01
EPI_ISL_5650359	RHD XV	RHD XV	2021-05-31
EPI_ISL_5650361	RHD XV	RHD XV	2021-06-01
EPI_ISL_5650371	RHD XV	RHD XV	2021-06-01
EPI_ISL_5650374	RHD XV	RHD XV	2021-06-01
EPI_ISL_5650377	RHD XV	RHD XV	2021-05-31
EPI_ISL_5650381	RHD XV	RHD XV	2021-06-01
EPI_ISL_5650385	RHD XV	RHD XV	2021-06-01
EPI_ISL_5650386	RHD XV	RHD XV	2021-06-01
EPI_ISL_5645571	RHD XV	RHD XV	2021-05-31
EPI_ISL_5645573	RHD XV	RHD XV	2021-05-31
EPI_ISL_5645574	RHD XV	RHD XV	2021-06-01
EPI_ISL_5645575	SJdRP	RHD XV	2021-05-31
EPI_ISL_5645576	RHD XV	RHD XV	2021-05-31
EPI_ISL_5645577	RHD XV	RHD XV	2021-06-01
EPI_ISL_5645579	SJdRP	RHD XV	2021-06-01
EPI_ISL_5645966	RHD XV	RHD XV	2021-05-19
EPI_ISL_5645969	RHD XV	RHD XV	2021-05-19
EPI_ISL_5645974	RHD XV	RHD XV	2021-05-19
EPI_ISL_5645975	RHD XV	RHD XV	2021-05-19
EPI_ISL_5645976	RHD XV	RHD XV	2021-05-19
EPI_ISL_5645977	RHD XV	RHD XV	2021-05-19
EPI_ISL_5645979	RHD XV	RHD XV	2021-05-19

EPI_ISL_5645981	RHD XV	RHD XV	2021-05-19
EPI_ISL_5645982	RHD XV	RHD XV	2021-05-19
EPI_ISL_5645984	RHD XV	RHD XV	2021-05-19
EPI_ISL_5645986	RHD XV	RHD XV	2021-05-19
EPI_ISL_5645987	RHD XV	RHD XV	2021-05-19
EPI_ISL_5645988	RHD XV	RHD XV	2021-05-19
EPI_ISL_5645989	RHD XV	RHD XV	2021-05-19
EPI_ISL_5645993	RHD XV	RHD XV	2021-05-19
EPI_ISL_5645994	RHD XV	RHD XV	2021-05-19
EPI_ISL_5645995	RHD XV	RHD XV	2021-05-19
EPI_ISL_5645997	RHD XV	RHD XV	2021-05-19
EPI_ISL_5645998	RHD XV	RHD XV	2021-05-19
EPI_ISL_5646000	RHD XV	RHD XV	2021-05-19
EPI_ISL_5646003	RHD XV	RHD XV	2021-05-19
EPI_ISL_5646004	RHD XV	RHD XV	2021-05-19
EPI_ISL_5646005	RHD XV	RHD XV	2021-05-19
EPI_ISL_5646006	SJdRP	RHD XV	2021-05-19
EPI_ISL_5646007	RHD XV	RHD XV	2021-05-19
EPI_ISL_5646010	RHD XV	RHD XV	2021-05-19
EPI_ISL_5646011	RHD XV	RHD XV	2021-05-19
EPI_ISL_5646012	RHD XV	RHD XV	2021-05-19
EPI_ISL_5646013	RHD XV	RHD XV	2021-05-19
EPI_ISL_5646014	RHD XV	RHD XV	2021-05-19
EPI_ISL_5646015	RHD XV	RHD XV	2021-05-19
EPI_ISL_5646016	RHD XV	RHD XV	2021-05-20
EPI_ISL_5646018	RHD XV	RHD XV	2021-05-19
EPI_ISL_5646023	RHD XV	RHD XV	2021-05-19
EPI_ISL_5646024	RHD XV	RHD XV	2021-05-19
EPI_ISL_5646025	RHD XV	RHD XV	2021-05-20
EPI_ISL_5646028	RHD XV	RHD XV	2021-05-20
EPI_ISL_5646029	RHD XV	RHD XV	2021-05-19
EPI_ISL_5646031	RHD XV	RHD XV	2021-05-19
EPI_ISL_5646032	RHD XV	RHD XV	2021-05-19
EPI_ISL_5646033	RHD XV	RHD XV	2021-05-19
EPI_ISL_5646304	RHD XV	RHD XV	2021-05-31
EPI_ISL_5646309	RHD XV	RHD XV	2021-05-31
EPI_ISL_5646319	RHD XV	RHD XV	2021-05-31
EPI_ISL_5646320	RHD XV	RHD XV	2021-05-31
EPI_ISL_5646321	RHD XV	RHD XV	2021-05-31
EPI_ISL_5646322	RHD XV	RHD XV	2021-05-31
EPI_ISL_5646323	RHD XV	RHD XV	2021-05-31
EPI_ISL_5646329	RHD XV	RHD XV	2021-05-31
EPI_ISL_5646332	RHD XV	RHD XV	2021-05-31
EPI_ISL_5646345	RHD XV	RHD XV	2021-05-31
EPI_ISL_5646355	RHD XV	RHD XV	2021-05-31
EPI_ISL_5646363	RHD XV	RHD XV	2021-05-31
EPI_ISL_5646368	RHD XV	RHD XV	2021-05-31
EPI_ISL_5646369	RHD XV	RHD XV	2021-05-31
EPI_ISL_5646374	RHD XV	RHD XV	2021-05-31
EPI_ISL_5646382	RHD XV	RHD XV	2021-05-31
EPI_ISL_5646383	RHD XV	RHD XV	2021-05-31
EPI_ISL_5646871	RHD XV	RHD XV	2021-06-07
EPI_ISL_5646875	RHD XV	RHD XV	2021-06-07
EPI_ISL_5646876	RHD XV	RHD XV	2021-06-08
EPI_ISL_5646877	RHD XV	RHD XV	2021-06-07
EPI_ISL_5646880	RHD XV	RHD XV	2021-06-08
EPI_ISL_5646881	RHD XV	RHD XV	2021-06-07
EPI_ISL_5646882	RHD XV	RHD XV	2021-06-07
EPI_ISL_5646884	RHD XV	RHD XV	2021-06-07
EPI_ISL_5646885	RHD XV	RHD XV	2021-06-07
EPI_ISL_5646888	RHD XV	RHD XV	2021-06-07
EPI_ISL_5646889	RHD XV	RHD XV	2021-06-07
EPI_ISL_5646894	RHD XV	RHD XV	2021-06-08

EPI_ISL_5646896	RHD XV	RHD XV	2021-06-08
EPI_ISL_5646897	RHD XV	RHD XV	2021-06-07
EPI_ISL_5646901	RHD XV	RHD XV	2021-06-07
EPI_ISL_5646902	North	RHD XV	2021-06-07
EPI_ISL_5646907	RHD XV	RHD XV	2021-06-08
EPI_ISL_5646910	RHD XV	RHD XV	2021-06-07
EPI_ISL_5646913	RHD XV	RHD XV	2021-06-08
EPI_ISL_5646914	RHD XV	RHD XV	2021-06-08
EPI_ISL_5646915	RHD XV	RHD XV	2021-06-08
EPI_ISL_5646916	North	RHD XV	2021-06-07
EPI_ISL_5646917	RHD XV	RHD XV	2021-06-07
EPI_ISL_5646921	RHD XV	RHD XV	2021-06-07
EPI_ISL_5646930	RHD XV	RHD XV	2021-06-08
EPI_ISL_5646931	RHD XV	RHD XV	2021-06-07
EPI_ISL_5646932	SJdRP	RHD XV	2021-06-07
EPI_ISL_5646933	RHD XV	RHD XV	2021-06-07
EPI_ISL_5646943	RHD XV	RHD XV	2021-06-07
EPI_ISL_5646962	RHD XV	RHD XV	2021-06-07
EPI_ISL_5646963	RHD XV	RHD XV	2021-06-07
EPI_ISL_5646967	RHD XV	RHD XV	2021-06-07
EPI_ISL_5646968	RHD XV	RHD XV	2021-06-07
EPI_ISL_5646976	RHD XV	RHD XV	2021-06-07
EPI_ISL_5646977	SJdRP	RHD XV	2021-06-07
EPI_ISL_5646979	RHD XV	RHD XV	2021-06-06
EPI_ISL_5646982	RHD XV	RHD XV	2021-06-06
EPI_ISL_5646983	RHD XV	RHD XV	2021-06-07
EPI_ISL_5646986	SJdRP	RHD XV	2021-06-07
EPI_ISL_5646987	SJdRP	RHD XV	2021-06-06
EPI_ISL_5646993	RHD XV	RHD XV	2021-06-07
EPI_ISL_5646995	RHD XV	RHD XV	2021-06-06
EPI_ISL_5647002	RHD XV	RHD XV	2021-06-06
EPI_ISL_5647003	RHD XV	RHD XV	2021-06-06
EPI_ISL_5647110	RHD XV	RHD XV	2021-05-31
EPI_ISL_5647113	RHD XV	RHD XV	2021-06-01
EPI_ISL_5647114	RHD XV	RHD XV	2021-05-31
EPI_ISL_5647115	SJdRP	RHD XV	2021-05-31
EPI_ISL_5647117	RHD XV	RHD XV	2021-05-31
EPI_ISL_5647119	RHD XV	RHD XV	2021-05-31
EPI_ISL_5647121	RHD XV	RHD XV	2021-05-31
EPI_ISL_5647123	RHD XV	RHD XV	2021-05-31
EPI_ISL_5647124	RHD XV	RHD XV	2021-06-01
EPI_ISL_5647127	RHD XV	RHD XV	2021-06-01
EPI_ISL_5647129	RHD XV	RHD XV	2021-05-31
EPI_ISL_5647132	RHD XV	RHD XV	2021-05-31
EPI_ISL_5647133	SJdRP	RHD XV	2021-05-31
EPI_ISL_5647136	RHD XV	RHD XV	2021-05-31
EPI_ISL_5647137	RHD XV	RHD XV	2021-05-31
EPI_ISL_5647147	RHD XV	RHD XV	2021-05-31
EPI_ISL_5647150	RHD XV	RHD XV	2021-05-31
EPI_ISL_5647153	RHD XV	RHD XV	2021-05-31
EPI_ISL_5647154	Midwest	RHD XV	2021-05-31
EPI_ISL_5647156	RHD XV	RHD XV	2021-05-31
EPI_ISL_5647157	RHD XV	RHD XV	2021-05-31
EPI_ISL_5647158	RHD XV	RHD XV	2021-06-01
EPI_ISL_5647168	RHD XV	RHD XV	2021-05-31
EPI_ISL_5647169	RHD XV	RHD XV	2021-05-31
EPI_ISL_5647171	RHD XV	RHD XV	2021-05-31
EPI_ISL_5647173	RHD XV	RHD XV	2021-06-01
EPI_ISL_5647177	RHD XV	RHD XV	2021-05-31
EPI_ISL_5647187	RHD XV	RHD XV	2021-05-31
EPI_ISL_5647189	RHD XV	RHD XV	2021-05-31
EPI_ISL_5647190	RHD XV	RHD XV	2021-05-31
EPI_ISL_5647191	RHD XV	RHD XV	2021-05-31

EPI_ISL_5647193	RHD XV	RHD XV	2021-05-31
EPI_ISL_5647197	RHD XV	RHD XV	2021-06-01
EPI_ISL_5647204	RHD XV	RHD XV	2021-06-07
EPI_ISL_5647205	RHD XV	RHD XV	2021-06-07
EPI_ISL_5647209	Northeast	RHD XV	2021-06-07
EPI_ISL_5647210	RHD XV	RHD XV	2021-06-08
EPI_ISL_5647212	RHD XV	RHD XV	2021-06-07
EPI_ISL_5647213	RHD XV	RHD XV	2021-06-07
EPI_ISL_5647214	RHD XV	RHD XV	2021-06-08
EPI_ISL_5647216	RHD XV	RHD XV	2021-06-06
EPI_ISL_5647219	RHD XV	RHD XV	2021-06-07
EPI_ISL_5647220	RHD XV	RHD XV	2021-06-07
EPI_ISL_5647227	RHD XV	RHD XV	2021-06-07
EPI_ISL_5647228	RHD XV	RHD XV	2021-06-06
EPI_ISL_5647229	RHD XV	RHD XV	2021-06-07
EPI_ISL_5647233	RHD XV	RHD XV	2021-06-06
EPI_ISL_5647236	RHD XV	RHD XV	2021-06-07
EPI_ISL_5647240	RHD XV	RHD XV	2021-06-06
EPI_ISL_5647241	RHD XV	RHD XV	2021-06-07
EPI_ISL_5647243	RHD XV	RHD XV	2021-06-06
EPI_ISL_5647244	RHD XV	RHD XV	2021-06-07
EPI_ISL_5647245	SJdRP	RHD XV	2021-06-07
EPI_ISL_5647247	RHD XV	RHD XV	2021-06-07
EPI_ISL_5647249	RHD XV	RHD XV	2021-06-06
EPI_ISL_5647251	RHD XV	RHD XV	2021-06-07
EPI_ISL_5647254	RHD XV	RHD XV	2021-06-07
EPI_ISL_5647257	RHD XV	RHD XV	2021-06-08
EPI_ISL_5647259	RHD XV	RHD XV	2021-06-07
EPI_ISL_5647260	RHD XV	RHD XV	2021-06-07
EPI_ISL_5647261	RHD XV	RHD XV	2021-06-07
EPI_ISL_5647262	RHD XV	RHD XV	2021-06-08
EPI_ISL_5647263	RHD XV	RHD XV	2021-06-07
EPI_ISL_5647266	RHD XV	RHD XV	2021-06-07
EPI_ISL_5647267	RHD XV	RHD XV	2021-06-08
EPI_ISL_5647268	RHD XV	RHD XV	2021-06-07
EPI_ISL_5647271	SJdRP	RHD XV	2021-06-08
EPI_ISL_5647272	RHD XV	RHD XV	2021-06-07
EPI_ISL_5647275	RHD XV	RHD XV	2021-06-08
EPI_ISL_5647292	RHD XV	RHD XV	2021-06-07
EPI_ISL_5647294	RHD XV	RHD XV	2021-06-07
EPI_ISL_5647657	Southeast	RHD XV	2021-06-14
EPI_ISL_5647663	RHD XV	RHD XV	2021-06-14
EPI_ISL_5647676	RHD XV	RHD XV	2021-06-15
EPI_ISL_5647687	RHD XV	RHD XV	2021-06-15
EPI_ISL_5647689	RHD XV	RHD XV	2021-06-14
EPI_ISL_5647697	RHD XV	RHD XV	2021-06-15
EPI_ISL_5647698	RHD XV	RHD XV	2021-06-14
EPI_ISL_5647704	RHD XV	RHD XV	2021-06-15
EPI_ISL_5647710	RHD XV	RHD XV	2021-06-15
EPI_ISL_5647715	RHD XV	RHD XV	2021-06-14
EPI_ISL_5647716	RHD XV	RHD XV	2021-06-15
EPI_ISL_5647717	RHD XV	RHD XV	2021-06-15
EPI_ISL_5647721	RHD XV	RHD XV	2021-06-15
EPI_ISL_5647724	RHD XV	RHD XV	2021-06-15
EPI_ISL_5647729	RHD XV	RHD XV	2021-06-15
EPI_ISL_5647731	RHD XV	RHD XV	2021-06-15
EPI_ISL_5647733	RHD XV	RHD XV	2021-06-14
EPI_ISL_5647736	RHD XV	RHD XV	2021-06-14
EPI_ISL_5647754	SJdRP	RHD XV	2021-06-14
EPI_ISL_5647757	Southeast	RHD XV	2021-06-14
EPI_ISL_5647759	RHD XV	RHD XV	2021-06-14
EPI_ISL_5647760	RHD XV	RHD XV	2021-06-15
EPI_ISL_5647761	RHD XV	RHD XV	2021-06-15

EPI_ISL_5647762	RHD XV	RHD XV	2021-06-14
EPI_ISL_5647774	RHD XV	RHD XV	2021-06-15
EPI_ISL_5647776	RHD XV	RHD XV	2021-06-14
EPI_ISL_5647778	RHD XV	RHD XV	2021-06-15
EPI_ISL_5647779	RHD XV	RHD XV	2021-06-14
EPI_ISL_5647781	RHD XV	RHD XV	2021-06-14
EPI_ISL_5647786	RHD XV	RHD XV	2021-06-14
EPI_ISL_5647787	RHD XV	RHD XV	2021-06-14
EPI_ISL_5647790	RHD XV	RHD XV	2021-06-14
EPI_ISL_5647791	SJdRP	RHD XV	2021-06-15
EPI_ISL_5647792	RHD XV	RHD XV	2021-06-14
EPI_ISL_5647793	RHD XV	RHD XV	2021-06-14
EPI_ISL_5647800	RHD XV	RHD XV	2021-06-14
EPI_ISL_5647803	RHD XV	RHD XV	2021-06-14
EPI_ISL_5647807	RHD XV	RHD XV	2021-06-15
EPI_ISL_5647811	RHD XV	RHD XV	2021-06-14
EPI_ISL_5647812	RHD XV	RHD XV	2021-06-14
EPI_ISL_5647813	RHD XV	RHD XV	2021-06-14
EPI_ISL_5647820	RHD XV	RHD XV	2021-06-15
EPI_ISL_5647831	RHD XV	RHD XV	2021-06-14
EPI_ISL_5647832	RHD XV	RHD XV	2021-06-14
EPI_ISL_5647835	RHD XV	RHD XV	2021-06-14
EPI_ISL_5647836	RHD XV	RHD XV	2021-06-14
EPI_ISL_5647843	RHD XV	RHD XV	2021-06-15
EPI_ISL_5647848	RHD XV	RHD XV	2021-06-14
EPI_ISL_5647850	RHD XV	RHD XV	2021-06-14
EPI_ISL_5647852	RHD XV	RHD XV	2021-06-14
EPI_ISL_5647859	RHD XV	RHD XV	2021-06-14
EPI_ISL_5647860	RHD XV	RHD XV	2021-06-14
EPI_ISL_5647958	RHD XV	RHD XV	2021-06-14
EPI_ISL_5647962	RHD XV	RHD XV	2021-06-14
EPI_ISL_5647968	RHD XV	RHD XV	2021-06-14
EPI_ISL_5647979	North	RHD XV	2021-06-14
EPI_ISL_5647983	RHD XV	RHD XV	2021-06-14
EPI_ISL_5647999	RHD XV	RHD XV	2021-06-14
EPI_ISL_5648011	RHD XV	RHD XV	2021-06-14
EPI_ISL_5648032	RHD XV	RHD XV	2021-06-14
EPI_ISL_5648033	RHD XV	RHD XV	2021-06-14
EPI_ISL_5648038	RHD XV	RHD XV	2021-06-14
EPI_ISL_5648044	RHD XV	RHD XV	2021-06-14
EPI_ISL_5648047	RHD XV	RHD XV	2021-06-13
EPI_ISL_5648050	RHD XV	RHD XV	2021-06-14
EPI_ISL_5648129	RHD XV	RHD XV	2021-06-13
EPI_ISL_5648131	RHD XV	RHD XV	2021-06-14
EPI_ISL_5648134	RHD XV	RHD XV	2021-06-15
EPI_ISL_5648135	RHD XV	RHD XV	2021-06-15
EPI_ISL_5648138	RHD XV	RHD XV	2021-06-14
EPI_ISL_5648139	RHD XV	RHD XV	2021-06-15
EPI_ISL_5648140	RHD XV	RHD XV	2021-06-14
EPI_ISL_5648141	RHD XV	RHD XV	2021-06-14
EPI_ISL_5648144	RHD XV	RHD XV	2021-06-14
EPI_ISL_5648145	RHD XV	RHD XV	2021-06-14
EPI_ISL_5648146	RHD XV	RHD XV	2021-06-14
EPI_ISL_5648147	RHD XV	RHD XV	2021-06-14
EPI_ISL_5648149	RHD XV	RHD XV	2021-06-14
EPI_ISL_5648150	RHD XV	RHD XV	2021-06-14
EPI_ISL_5648151	RHD XV	RHD XV	2021-06-14
EPI_ISL_5648153	RHD XV	RHD XV	2021-06-14
EPI_ISL_5648155	RHD XV	RHD XV	2021-06-14
EPI_ISL_5648156	RHD XV	RHD XV	2021-06-14
EPI_ISL_5648157	RHD XV	RHD XV	2021-06-14
EPI_ISL_5648158	RHD XV	RHD XV	2021-06-14
EPI_ISL_5648159	RHD XV	RHD XV	2021-06-14

EPI_ISL_5648171	RHD XV	RHD XV	2021-06-14
EPI_ISL_5648173	RHD XV	RHD XV	2021-06-14
EPI_ISL_5648174	SJdRP	RHD XV	2021-06-15
EPI_ISL_5648175	RHD XV	RHD XV	2021-06-14
EPI_ISL_5648177	RHD XV	RHD XV	2021-06-15
EPI_ISL_5648180	RHD XV	RHD XV	2021-06-14
EPI_ISL_5648184	RHD XV	RHD XV	2021-06-14
EPI_ISL_5648185	RHD XV	RHD XV	2021-06-14
EPI_ISL_5648188	RHD XV	RHD XV	2021-06-14
EPI_ISL_5648195	RHD XV	RHD XV	2021-06-14
EPI_ISL_5648196	RHD XV	RHD XV	2021-06-14
EPI_ISL_5648197	RHD XV	RHD XV	2021-06-14
EPI_ISL_5648198	RHD XV	RHD XV	2021-06-14
EPI_ISL_5648200	RHD XV	RHD XV	2021-06-14
EPI_ISL_5648202	RHD XV	RHD XV	2021-06-14
EPI_ISL_5648206	RHD XV	RHD XV	2021-06-14
EPI_ISL_5648207	RHD XV	RHD XV	2021-06-14
EPI_ISL_5648208	RHD XV	RHD XV	2021-06-14
EPI_ISL_5648209	RHD XV	RHD XV	2021-06-14
EPI_ISL_5648210	RHD XV	RHD XV	2021-06-15
EPI_ISL_5648211	RHD XV	RHD XV	2021-06-15
EPI_ISL_5648212	Southeast	RHD XV	2021-06-14
EPI_ISL_5648214	RHD XV	RHD XV	2021-06-14
EPI_ISL_5648215	RHD XV	RHD XV	2021-06-14
EPI_ISL_5648216	RHD XV	RHD XV	2021-06-14
EPI_ISL_5648217	RHD XV	RHD XV	2021-06-14
EPI_ISL_5648218	RHD XV	RHD XV	2021-06-14
EPI_ISL_5648219	RHD XV	RHD XV	2021-06-14
EPI_ISL_5648220	RHD XV	RHD XV	2021-06-14
EPI_ISL_5648221	RHD XV	RHD XV	2021-06-14
EPI_ISL_5648223	RHD XV	RHD XV	2021-06-14
EPI_ISL_5648266	RHD XV	RHD XV	2021-06-21
EPI_ISL_5648278	RHD XV	RHD XV	2021-06-21
EPI_ISL_5648305	RHD XV	RHD XV	2021-06-21
EPI_ISL_5648381	RHD XV	RHD XV	2021-06-22
EPI_ISL_5648388	RHD XV	RHD XV	2021-06-21
EPI_ISL_5648434	RHD XV	RHD XV	2021-06-21
EPI_ISL_5648437	RHD XV	RHD XV	2021-06-21
EPI_ISL_5648438	RHD XV	RHD XV	2021-06-22
EPI_ISL_5648449	RHD XV	RHD XV	2021-06-22
EPI_ISL_5648461	RHD XV	RHD XV	2021-06-21
EPI_ISL_5648484	RHD XV	RHD XV	2021-06-22
EPI_ISL_5648488	RHD XV	RHD XV	2021-06-21
EPI_ISL_5648498	RHD XV	RHD XV	2021-06-21
EPI_ISL_5648503	RHD XV	RHD XV	2021-06-22
EPI_ISL_5648505	RHD XV	RHD XV	2021-06-21
EPI_ISL_5648508	Northeast	RHD XV	2021-06-21
EPI_ISL_5648546	RHD XV	RHD XV	2021-06-22
EPI_ISL_5648570	RHD XV	RHD XV	2021-06-22
EPI_ISL_5648572	RHD XV	RHD XV	2021-06-21
EPI_ISL_5648580	RHD XV	RHD XV	2021-06-21
EPI_ISL_5648581	RHD XV	RHD XV	2021-06-21
EPI_ISL_5648588	RHD XV	RHD XV	2021-06-21
EPI_ISL_5648592	RHD XV	RHD XV	2021-06-21
EPI_ISL_5648595	RHD XV	RHD XV	2021-06-21
EPI_ISL_5648598	RHD XV	RHD XV	2021-06-21
EPI_ISL_5648606	RHD XV	RHD XV	2021-06-21
EPI_ISL_5648612	RHD XV	RHD XV	2021-06-21
EPI_ISL_5648623	RHD XV	RHD XV	2021-06-22
EPI_ISL_5648624	RHD XV	RHD XV	2021-06-22
EPI_ISL_5648655	RHD XV	RHD XV	2021-06-21
EPI_ISL_5648663	RHD XV	RHD XV	2021-06-21
EPI_ISL_5648670	RHD XV	RHD XV	2021-06-22

EPI_ISL_5648691	RHD XV	RHD XV	2021-06-21
EPI_ISL_5648699	RHD XV	RHD XV	2021-06-21
EPI_ISL_5648722	RHD XV	RHD XV	2021-06-22
EPI_ISL_5648728	RHD XV	RHD XV	2021-06-21
EPI_ISL_5648732	RHD XV	RHD XV	2021-06-21
EPI_ISL_5648733	RHD XV	RHD XV	2021-06-21
EPI_ISL_5648741	RHD XV	RHD XV	2021-06-21
EPI_ISL_5648745	RHD XV	RHD XV	2021-06-21
EPI_ISL_5648746	RHD XV	RHD XV	2021-06-21
EPI_ISL_5648750	RHD XV	RHD XV	2021-06-21
EPI_ISL_5648752	RHD XV	RHD XV	2021-06-21
EPI_ISL_5648767	RHD XV	RHD XV	2021-06-21
EPI_ISL_5648772	RHD XV	RHD XV	2021-06-21
EPI_ISL_5648776	RHD XV	RHD XV	2021-06-22
EPI_ISL_5648779	RHD XV	RHD XV	2021-06-21
EPI_ISL_5648800	RHD XV	RHD XV	2021-06-21
EPI_ISL_5648806	RHD XV	RHD XV	2021-06-22
EPI_ISL_5648809	RHD XV	RHD XV	2021-06-21
EPI_ISL_5648837	RHD XV	RHD XV	2021-06-22
EPI_ISL_5648839	RHD XV	RHD XV	2021-06-22
EPI_ISL_5648861	RHD XV	RHD XV	2021-06-21
EPI_ISL_5648872	RHD XV	RHD XV	2021-06-21
EPI_ISL_5648898	RHD XV	RHD XV	2021-06-22
EPI_ISL_5648907	RHD XV	RHD XV	2021-06-21
EPI_ISL_5648938	RHD XV	RHD XV	2021-06-21
EPI_ISL_5648943	RHD XV	RHD XV	2021-06-21
EPI_ISL_5648964	RHD XV	RHD XV	2021-06-22
EPI_ISL_5648971	RHD XV	RHD XV	2021-06-21
EPI_ISL_5648972	RHD XV	RHD XV	2021-06-21
EPI_ISL_5648978	North	RHD XV	2021-06-21
EPI_ISL_5648980	RHD XV	RHD XV	2021-06-21
EPI_ISL_5649005	RHD XV	RHD XV	2021-06-21
EPI_ISL_5649015	RHD XV	RHD XV	2021-06-21
EPI_ISL_5649026	RHD XV	RHD XV	2021-06-21
EPI_ISL_5649031	RHD XV	RHD XV	2021-06-21
EPI_ISL_5649032	RHD XV	RHD XV	2021-06-22
EPI_ISL_5649038	RHD XV	RHD XV	2021-06-21
EPI_ISL_5649039	RHD XV	RHD XV	2021-06-21
EPI_ISL_5649051	RHD XV	RHD XV	2021-06-21
EPI_ISL_5649052	RHD XV	RHD XV	2021-06-21
EPI_ISL_5649083	Northeast	RHD XV	2021-06-21
EPI_ISL_5649101	RHD XV	RHD XV	2021-06-22
EPI_ISL_5649135	RHD XV	RHD XV	2021-06-22
EPI_ISL_5649136	RHD XV	RHD XV	2021-06-21
EPI_ISL_5649139	RHD XV	RHD XV	2021-06-21
EPI_ISL_5649433	RHD XV	RHD XV	2021-06-21
EPI_ISL_5649445	RHD XV	RHD XV	2021-06-21
EPI_ISL_5649457	RHD XV	RHD XV	2021-06-21
EPI_ISL_5649464	RHD XV	RHD XV	2021-06-21
EPI_ISL_5649465	RHD XV	RHD XV	2021-06-21
EPI_ISL_5649484	RHD XV	RHD XV	2021-06-21
EPI_ISL_5659520	RHD XV	RHD XV	2021-06-21
EPI_ISL_5659533	RHD XV	RHD XV	2021-06-21
EPI_ISL_5659536	RHD XV	RHD XV	2021-06-21
EPI_ISL_5659537	RHD XV	RHD XV	2021-06-21
EPI_ISL_5659538	RHD XV	RHD XV	2021-06-21
EPI_ISL_5659539	RHD XV	RHD XV	2021-06-21
EPI_ISL_5659541	RHD XV	RHD XV	2021-06-21
EPI_ISL_5659542	RHD XV	RHD XV	2021-06-21
EPI_ISL_5659543	RHD XV	RHD XV	2021-06-21
EPI_ISL_5659544	RHD XV	RHD XV	2021-06-21
EPI_ISL_5659545	RHD XV	RHD XV	2021-06-21
EPI_ISL_5659546	RHD XV	RHD XV	2021-06-21

EPI_ISL_5659547	RHD XV	RHD XV	2021-06-21
EPI_ISL_5659548	RHD XV	RHD XV	2021-06-21
EPI_ISL_5659549	RHD XV	RHD XV	2021-06-21
EPI_ISL_5659550	RHD XV	RHD XV	2021-06-21
EPI_ISL_5659551	RHD XV	RHD XV	2021-06-21
EPI_ISL_5659552	RHD XV	RHD XV	2021-06-22
EPI_ISL_5659553	RHD XV	RHD XV	2021-06-22
EPI_ISL_5659554	RHD XV	RHD XV	2021-06-21
EPI_ISL_5659555	RHD XV	RHD XV	2021-06-21
EPI_ISL_5659556	SJdRP	RHD XV	2021-06-21
EPI_ISL_5659557	RHD XV	RHD XV	2021-06-21
EPI_ISL_5659558	RHD XV	RHD XV	2021-06-21
EPI_ISL_5659559	RHD XV	RHD XV	2021-06-21
EPI_ISL_5659560	RHD XV	RHD XV	2021-06-21
EPI_ISL_5659561	RHD XV	RHD XV	2021-06-21
EPI_ISL_5659562	RHD XV	RHD XV	2021-06-21
EPI_ISL_5659563	RHD XV	RHD XV	2021-06-20
EPI_ISL_5659564	RHD XV	RHD XV	2021-06-21
EPI_ISL_5659565	RHD XV	RHD XV	2021-06-21
EPI_ISL_5659566	RHD XV	RHD XV	2021-06-22
EPI_ISL_5659567	RHD XV	RHD XV	2021-06-21
EPI_ISL_5659568	RHD XV	RHD XV	2021-06-21
EPI_ISL_5659569	RHD XV	RHD XV	2021-06-21
EPI_ISL_5659570	RHD XV	RHD XV	2021-06-21
EPI_ISL_5659571	RHD XV	RHD XV	2021-06-21
EPI_ISL_5659572	RHD XV	RHD XV	2021-06-22
EPI_ISL_5659573	RHD XV	RHD XV	2021-06-21
EPI_ISL_5659574	RHD XV	RHD XV	2021-06-21
EPI_ISL_5659575	RHD XV	RHD XV	2021-06-21
EPI_ISL_5659576	RHD XV	RHD XV	2021-06-22
EPI_ISL_5659577	RHD XV	RHD XV	2021-06-21
EPI_ISL_5659578	RHD XV	RHD XV	2021-06-22
EPI_ISL_5659579	RHD XV	RHD XV	2021-06-21
EPI_ISL_5659580	RHD XV	RHD XV	2021-06-21
EPI_ISL_5659581	RHD XV	RHD XV	2021-06-22
EPI_ISL_5659582	RHD XV	RHD XV	2021-06-21
EPI_ISL_5659583	RHD XV	RHD XV	2021-06-21
EPI_ISL_5659584	RHD XV	RHD XV	2021-06-21
EPI_ISL_5659585	RHD XV	RHD XV	2021-06-21
EPI_ISL_5659586	RHD XV	RHD XV	2021-06-22
EPI_ISL_5659587	RHD XV	RHD XV	2021-06-21
EPI_ISL_5659588	RHD XV	RHD XV	2021-06-21
EPI_ISL_5659589	RHD XV	RHD XV	2021-06-21
EPI_ISL_5659590	RHD XV	RHD XV	2021-06-21
EPI_ISL_5659591	RHD XV	RHD XV	2021-06-21
EPI_ISL_5659592	RHD XV	RHD XV	2021-06-21
EPI_ISL_5659593	RHD XV	RHD XV	2021-06-20
EPI_ISL_5659594	RHD XV	RHD XV	2021-06-22
EPI_ISL_5659595	RHD XV	RHD XV	2021-06-21
EPI_ISL_5659596	RHD XV	RHD XV	2021-06-21
EPI_ISL_5659597	RHD XV	RHD XV	2021-06-21
EPI_ISL_5659598	RHD XV	RHD XV	2021-06-22
EPI_ISL_5659599	RHD XV	RHD XV	2021-06-22
EPI_ISL_5659600	RHD XV	RHD XV	2021-06-21
EPI_ISL_5659601	RHD XV	RHD XV	2021-06-21
EPI_ISL_5659602	RHD XV	RHD XV	2021-06-22
EPI_ISL_5659603	RHD XV	RHD XV	2021-06-21
EPI_ISL_5659604	RHD XV	RHD XV	2021-06-21
EPI_ISL_5659605	RHD XV	RHD XV	2021-06-21
EPI_ISL_5659606	RHD XV	RHD XV	2021-06-22
EPI_ISL_5659607	RHD XV	RHD XV	2021-06-21
EPI_ISL_5659608	RHD XV	RHD XV	2021-06-22
EPI_ISL_5659609	RHD XV	RHD XV	2021-06-21

EPI_ISL_5659610	RHD XV	RHD XV	2021-06-21
EPI_ISL_5659611	RHD XV	RHD XV	2021-06-21
EPI_ISL_5659612	RHD XV	RHD XV	2021-06-21
EPI_ISL_5659616	RHD XV	RHD XV	2021-06-21
EPI_ISL_5659617	RHD XV	RHD XV	2021-06-21
EPI_ISL_5659618	RHD XV	RHD XV	2021-06-21
EPI_ISL_5659619	RHD XV	RHD XV	2021-06-21
EPI_ISL_5659620	RHD XV	RHD XV	2021-06-21
EPI_ISL_5659621	RHD XV	RHD XV	2021-06-21
EPI_ISL_5659622	RHD XV	RHD XV	2021-06-21
EPI_ISL_5659623	RHD XV	RHD XV	2021-06-21
EPI_ISL_5659624	RHD XV	RHD XV	2021-06-21
EPI_ISL_5659625	RHD XV	RHD XV	2021-06-21
EPI_ISL_5659626	RHD XV	RHD XV	2021-06-22
EPI_ISL_5659627	RHD XV	RHD XV	2021-06-21
EPI_ISL_5659628	RHD XV	RHD XV	2021-06-21
EPI_ISL_5659629	RHD XV	RHD XV	2021-06-21
EPI_ISL_5659630	RHD XV	RHD XV	2021-06-21
EPI_ISL_5659631	RHD XV	RHD XV	2021-06-21
EPI_ISL_5659632	RHD XV	RHD XV	2021-06-21
EPI_ISL_5659633	RHD XV	RHD XV	2021-06-21
EPI_ISL_5659634	RHD XV	RHD XV	2021-06-21
EPI_ISL_5659635	RHD XV	RHD XV	2021-06-21
EPI_ISL_5659636	RHD XV	RHD XV	2021-06-21
EPI_ISL_5659637	RHD XV	RHD XV	2021-06-21
EPI_ISL_5659638	RHD XV	RHD XV	2021-06-21
EPI_ISL_5659639	RHD XV	RHD XV	2021-06-22
EPI_ISL_5659640	RHD XV	RHD XV	2021-06-21
EPI_ISL_5659656	RHD XV	RHD XV	2021-06-28
EPI_ISL_5659660	RHD XV	RHD XV	2021-06-29
EPI_ISL_5659686	RHD XV	RHD XV	2021-06-28
EPI_ISL_5659819	RHD XV	RHD XV	2021-06-29
EPI_ISL_5659939	RHD XV	RHD XV	2021-06-29
EPI_ISL_5660106	RHD XV	RHD XV	2021-06-29
EPI_ISL_5660147	RHD XV	RHD XV	2021-06-29
EPI_ISL_5660203	RHD XV	RHD XV	2021-06-29
EPI_ISL_5660292	RHD XV	RHD XV	2021-06-29
EPI_ISL_5660317	RHD XV	RHD XV	2021-06-28
EPI_ISL_5660351	RHD XV	RHD XV	2021-06-28
EPI_ISL_5660447	RHD XV	RHD XV	2021-06-28
EPI_ISL_5660631	RHD XV	RHD XV	2021-06-29
EPI_ISL_5660670	RHD XV	RHD XV	2021-06-28
EPI_ISL_5660878	RHD XV	RHD XV	2021-06-29
EPI_ISL_5660981	RHD XV	RHD XV	2021-06-29
EPI_ISL_5661059	RHD XV	RHD XV	2021-06-28
EPI_ISL_5661108	RHD XV	RHD XV	2021-06-28
EPI_ISL_5661123	RHD XV	RHD XV	2021-06-28
EPI_ISL_5666184	RHD XV	RHD XV	2021-06-28
EPI_ISL_5666189	RHD XV	RHD XV	2021-06-28
EPI_ISL_5666199	RHD XV	RHD XV	2021-06-29
EPI_ISL_5666208	RHD XV	RHD XV	2021-06-28
EPI_ISL_5666213	RHD XV	RHD XV	2021-06-28
EPI_ISL_5666225	RHD XV	RHD XV	2021-06-27
EPI_ISL_5666237	RHD XV	RHD XV	2021-06-28
EPI_ISL_5666249	South	RHD XV	2021-06-28
EPI_ISL_5666253	RHD XV	RHD XV	2021-06-28
EPI_ISL_5666265	RHD XV	RHD XV	2021-06-29
EPI_ISL_5666274	RHD XV	RHD XV	2021-06-28
EPI_ISL_5666281	RHD XV	RHD XV	2021-06-28
EPI_ISL_5666312	RHD XV	RHD XV	2021-06-28
EPI_ISL_5666322	RHD XV	RHD XV	2021-06-28
EPI_ISL_5666329	RHD XV	RHD XV	2021-06-28
EPI_ISL_5666335	Northeast	RHD XV	2021-06-28

EPI_ISL_5666342	RHD XV	RHD XV	2021-06-28
EPI_ISL_5666349	RHD XV	RHD XV	2021-06-29
EPI_ISL_5666374	RHD XV	RHD XV	2021-06-29
EPI_ISL_5666390	RHD XV	RHD XV	2021-06-28
EPI_ISL_5666400	RHD XV	RHD XV	2021-06-28
EPI_ISL_5666403	RHD XV	RHD XV	2021-06-28
EPI_ISL_5666414	RHD XV	RHD XV	2021-06-28
EPI_ISL_5666427	RHD XV	RHD XV	2021-06-28
EPI_ISL_5666434	RHD XV	RHD XV	2021-06-28
EPI_ISL_5666437	RHD XV	RHD XV	2021-06-28
EPI_ISL_5666469	RHD XV	RHD XV	2021-06-28
EPI_ISL_5666479	RHD XV	RHD XV	2021-06-28
EPI_ISL_5666487	RHD XV	RHD XV	2021-06-28
EPI_ISL_5666494	RHD XV	RHD XV	2021-06-28
EPI_ISL_5666502	SJdRP	RHD XV	2021-06-28
EPI_ISL_5666509	RHD XV	RHD XV	2021-06-28
EPI_ISL_5666517	RHD XV	RHD XV	2021-06-28
EPI_ISL_5666531	RHD XV	RHD XV	2021-06-28
EPI_ISL_5666540	RHD XV	RHD XV	2021-06-28
EPI_ISL_5666557	Northeast	RHD XV	2021-06-29
EPI_ISL_5666564	RHD XV	RHD XV	2021-06-28
EPI_ISL_5666569	RHD XV	RHD XV	2021-06-28
EPI_ISL_5666585	RHD XV	RHD XV	2021-06-28
EPI_ISL_5666601	RHD XV	RHD XV	2021-06-28
EPI_ISL_5666608	RHD XV	RHD XV	2021-06-28
EPI_ISL_5666619	RHD XV	RHD XV	2021-06-28
EPI_ISL_5666629	RHD XV	RHD XV	2021-06-28
EPI_ISL_5667268	RHD XV	RHD XV	2021-06-28
EPI_ISL_5667282	RHD XV	RHD XV	2021-06-28
EPI_ISL_5667291	North	RHD XV	2021-06-28
EPI_ISL_5667297	RHD XV	RHD XV	2021-06-28
EPI_ISL_5667315	RHD XV	RHD XV	2021-06-28
EPI_ISL_5667318	RHD XV	RHD XV	2021-06-28
EPI_ISL_5667325	RHD XV	RHD XV	2021-06-28
EPI_ISL_5667337	RHD XV	RHD XV	2021-06-28
EPI_ISL_5667356	RHD XV	RHD XV	2021-06-28
EPI_ISL_5667368	RHD XV	RHD XV	2021-06-28
EPI_ISL_5667374	RHD XV	RHD XV	2021-06-29
EPI_ISL_5667381	RHD XV	RHD XV	2021-06-28
EPI_ISL_5667387	RHD XV	RHD XV	2021-06-28
EPI_ISL_5667393	RHD XV	RHD XV	2021-06-28
EPI_ISL_5667399	RHD XV	RHD XV	2021-06-29
EPI_ISL_5667406	RHD XV	RHD XV	2021-06-28
EPI_ISL_5667423	RHD XV	RHD XV	2021-06-28
EPI_ISL_5667431	RHD XV	RHD XV	2021-06-28
EPI_ISL_5667445	SJdRP	RHD XV	2021-06-29
EPI_ISL_5667457	RHD XV	RHD XV	2021-06-28
EPI_ISL_5667490	RHD XV	RHD XV	2021-06-28
EPI_ISL_5667495	RHD XV	RHD XV	2021-06-28
EPI_ISL_5667507	RHD XV	RHD XV	2021-06-28
EPI_ISL_5667526	RHD XV	RHD XV	2021-06-28
EPI_ISL_5667534	RHD XV	RHD XV	2021-06-28
EPI_ISL_5667543	RHD XV	RHD XV	2021-06-28
EPI_ISL_5667562	RHD XV	RHD XV	2021-06-28
EPI_ISL_5667572	RHD XV	RHD XV	2021-06-29
EPI_ISL_5667580	RHD XV	RHD XV	2021-06-28
EPI_ISL_5667593	RHD XV	RHD XV	2021-06-28
EPI_ISL_5667605	RHD XV	RHD XV	2021-06-28
EPI_ISL_5667652	RHD XV	RHD XV	2021-06-28
EPI_ISL_5667654	RHD XV	RHD XV	2021-06-29
EPI_ISL_5667682	SJdRP	RHD XV	2021-06-28
EPI_ISL_5667685	RHD XV	RHD XV	2021-06-28
EPI_ISL_5667687	RHD XV	RHD XV	2021-06-28

EPI_ISL_5667698	RHD XV	RHD XV	2021-06-28
EPI_ISL_5667702	RHD XV	RHD XV	2021-06-29
EPI_ISL_5667708	RHD XV	RHD XV	2021-06-28
EPI_ISL_5667723	RHD XV	RHD XV	2021-06-28
EPI_ISL_5667727	RHD XV	RHD XV	2021-06-28
EPI_ISL_5667737	RHD XV	RHD XV	2021-06-28
EPI_ISL_5667747	RHD XV	RHD XV	2021-06-28
EPI_ISL_5667752	RHD XV	RHD XV	2021-06-28
EPI_ISL_5667757	RHD XV	RHD XV	2021-06-28
EPI_ISL_5667763	RHD XV	RHD XV	2021-06-28
EPI_ISL_5667773	RHD XV	RHD XV	2021-06-28
EPI_ISL_5667776	RHD XV	RHD XV	2021-06-28
EPI_ISL_5667783	RHD XV	RHD XV	2021-06-28
EPI_ISL_5667794	RHD XV	RHD XV	2021-06-28
EPI_ISL_5667803	RHD XV	RHD XV	2021-06-29
EPI_ISL_5667809	North	RHD XV	2021-06-29
EPI_ISL_5667870	RHD XV	RHD XV	2021-06-28
EPI_ISL_5667887	RHD XV	RHD XV	2021-06-29
EPI_ISL_5667902	RHD XV	RHD XV	2021-06-29
EPI_ISL_5667941	RHD XV	RHD XV	2021-06-29
EPI_ISL_5667949	RHD XV	RHD XV	2021-06-28
EPI_ISL_5667962	RHD XV	RHD XV	2021-06-28
EPI_ISL_5667977	RHD XV	RHD XV	2021-06-28
EPI_ISL_5667983	RHD XV	RHD XV	2021-06-29
EPI_ISL_5668014	RHD XV	RHD XV	2021-06-28
EPI_ISL_5668123	RHD XV	RHD XV	2021-06-29
EPI_ISL_5668145	RHD XV	RHD XV	2021-06-29
EPI_ISL_5668151	RHD XV	RHD XV	2021-06-29
EPI_ISL_5668168	RHD XV	RHD XV	2021-06-28
EPI_ISL_5668212	RHD XV	RHD XV	2021-06-28
EPI_ISL_5668253	RHD XV	RHD XV	2021-06-29
EPI_ISL_5668300	RHD XV	RHD XV	2021-06-28
EPI_ISL_5668347	RHD XV	RHD XV	2021-06-29
EPI_ISL_5668359	RHD XV	RHD XV	2021-06-29
EPI_ISL_5668369	RHD XV	RHD XV	2021-06-28
EPI_ISL_5668375	RHD XV	RHD XV	2021-06-29
EPI_ISL_5668378	RHD XV	RHD XV	2021-06-28
EPI_ISL_5668394	RHD XV	RHD XV	2021-06-29
EPI_ISL_5668402	RHD XV	RHD XV	2021-06-29
EPI_ISL_5672042	RHD XV	RHD XV	2021-07-05
EPI_ISL_5672056	RHD XV	RHD XV	2021-07-05
EPI_ISL_5672085	RHD XV	RHD XV	2021-07-05
EPI_ISL_5672090	RHD XV	RHD XV	2021-07-05
EPI_ISL_5672120	RHD XV	RHD XV	2021-07-05
EPI_ISL_5672129	RHD XV	RHD XV	2021-07-05
EPI_ISL_5672134	RHD XV	RHD XV	2021-07-05
EPI_ISL_5672174	RHD XV	RHD XV	2021-07-05
EPI_ISL_5672180	RHD XV	RHD XV	2021-07-05
EPI_ISL_5672185	RHD XV	RHD XV	2021-07-05
EPI_ISL_5672289	RHD XV	RHD XV	2021-07-05
EPI_ISL_5672319	RHD XV	RHD XV	2021-07-05
EPI_ISL_5672334	RHD XV	RHD XV	2021-07-05
EPI_ISL_5672370	RHD XV	RHD XV	2021-07-05
EPI_ISL_5672422	RHD XV	RHD XV	2021-07-05
EPI_ISL_5672434	RHD XV	RHD XV	2021-07-05
EPI_ISL_5672485	RHD XV	RHD XV	2021-07-05
EPI_ISL_5672497	RHD XV	RHD XV	2021-07-05
EPI_ISL_5672502	RHD XV	RHD XV	2021-07-05
EPI_ISL_5672514	RHD XV	RHD XV	2021-07-05
EPI_ISL_5672530	RHD XV	RHD XV	2021-07-05
EPI_ISL_5672550	RHD XV	RHD XV	2021-07-05
EPI_ISL_5673245	Southeast	RHD XV	2021-07-05
EPI_ISL_5673293	RHD XV	RHD XV	2021-07-05

EPI_ISL_5673342	RHD XV	RHD XV	2021-07-05
EPI_ISL_5673352	RHD XV	RHD XV	2021-07-05
EPI_ISL_5673372	RHD XV	RHD XV	2021-07-05
EPI_ISL_5673393	SJdRP	RHD XV	2021-07-05
EPI_ISL_5673399	RHD XV	RHD XV	2021-07-05
EPI_ISL_5673420	RHD XV	RHD XV	2021-07-05
EPI_ISL_5673429	RHD XV	RHD XV	2021-07-05
EPI_ISL_5673454	RHD XV	RHD XV	2021-07-05
EPI_ISL_5673464	RHD XV	RHD XV	2021-07-05
EPI_ISL_5673562	RHD XV	RHD XV	2021-07-05
EPI_ISL_5673583	RHD XV	RHD XV	2021-07-05
EPI_ISL_5673637	RHD XV	RHD XV	2021-07-05
EPI_ISL_5673654	RHD XV	RHD XV	2021-07-05
EPI_ISL_5673679	RHD XV	RHD XV	2021-07-05
EPI_ISL_5673686	RHD XV	RHD XV	2021-07-05
EPI_ISL_5673714	RHD XV	RHD XV	2021-07-05
EPI_ISL_5673742	RHD XV	RHD XV	2021-07-05
EPI_ISL_5673758	RHD XV	RHD XV	2021-07-05
EPI_ISL_5673763	RHD XV	RHD XV	2021-07-06
EPI_ISL_5673773	RHD XV	RHD XV	2021-07-05
EPI_ISL_5673807	RHD XV	RHD XV	2021-07-05
EPI_ISL_5673867	RHD XV	RHD XV	2021-07-05
EPI_ISL_5673883	SJdRP	RHD XV	2021-07-05
EPI_ISL_5673888	SJdRP	RHD XV	2021-07-05
EPI_ISL_5673920	RHD XV	RHD XV	2021-07-05
EPI_ISL_5673922	RHD XV	RHD XV	2021-07-06
EPI_ISL_5674030	RHD XV	RHD XV	2021-07-05
EPI_ISL_5674086	RHD XV	RHD XV	2021-07-05
EPI_ISL_5674112	RHD XV	RHD XV	2021-07-05
EPI_ISL_5674148	RHD XV	RHD XV	2021-07-06
EPI_ISL_5674177	SJdRP	RHD XV	2021-07-05
EPI_ISL_5674220	RHD XV	RHD XV	2021-07-05
EPI_ISL_5674278	RHD XV	RHD XV	2021-07-06
EPI_ISL_5674400	RHD XV	RHD XV	2021-07-05
EPI_ISL_5674434	RHD XV	RHD XV	2021-07-05
EPI_ISL_5674515	SJdRP	RHD XV	2021-07-05
EPI_ISL_5674624	RHD XV	RHD XV	2021-07-05
EPI_ISL_5674630	RHD XV	RHD XV	2021-07-05
EPI_ISL_5674809	RHD XV	RHD XV	2021-07-05
EPI_ISL_5674869	RHD XV	RHD XV	2021-07-05
EPI_ISL_5674874	RHD XV	RHD XV	2021-07-05
EPI_ISL_5674983	RHD XV	RHD XV	2021-07-05
EPI_ISL_5674987	RHD XV	RHD XV	2021-07-05
EPI_ISL_5675204	SJdRP	RHD XV	2021-07-05
EPI_ISL_5675352	RHD XV	RHD XV	2021-07-05
EPI_ISL_5675359	RHD XV	RHD XV	2021-07-05
EPI_ISL_5675365	RHD XV	RHD XV	2021-07-05
EPI_ISL_5675370	RHD XV	RHD XV	2021-07-05
EPI_ISL_5675381	RHD XV	RHD XV	2021-07-05
EPI_ISL_5675387	RHD XV	RHD XV	2021-07-06
EPI_ISL_5675394	RHD XV	RHD XV	2021-07-05
EPI_ISL_5675399	RHD XV	RHD XV	2021-07-05
EPI_ISL_5675408	RHD XV	RHD XV	2021-07-05
EPI_ISL_5675411	RHD XV	RHD XV	2021-07-05
EPI_ISL_5675415	RHD XV	RHD XV	2021-07-05
EPI_ISL_5675454	RHD XV	RHD XV	2021-07-05
EPI_ISL_5675464	RHD XV	RHD XV	2021-07-05
EPI_ISL_5675470	RHD XV	RHD XV	2021-07-05
EPI_ISL_5675487	RHD XV	RHD XV	2021-07-05
EPI_ISL_5675494	RHD XV	RHD XV	2021-07-05
EPI_ISL_5675588	RHD XV	RHD XV	2021-07-12
EPI_ISL_5675709	RHD XV	RHD XV	2021-07-12
EPI_ISL_5675719	RHD XV	RHD XV	2021-07-12

EPI_ISL_5675774	RHD XV	RHD XV	2021-07-12
EPI_ISL_5675793	RHD XV	RHD XV	2021-07-12
EPI_ISL_5675937	RHD XV	RHD XV	2021-07-12
EPI_ISL_5675943	RHD XV	RHD XV	2021-07-12
EPI_ISL_5676069	SJdRP	RHD XV	2021-07-12
EPI_ISL_5676108	RHD XV	RHD XV	2021-07-12
EPI_ISL_5676155	RHD XV	RHD XV	2021-07-12
EPI_ISL_5676188	RHD XV	RHD XV	2021-07-12
EPI_ISL_5676211	RHD XV	RHD XV	2021-07-12
EPI_ISL_5676361	RHD XV	RHD XV	2021-07-12
EPI_ISL_5676375	RHD XV	RHD XV	2021-07-12
EPI_ISL_5676582	RHD XV	RHD XV	2021-07-12
EPI_ISL_5676643	SJdRP	RHD XV	2021-07-12
EPI_ISL_5676683	RHD XV	RHD XV	2021-07-12
EPI_ISL_5676806	RHD XV	RHD XV	2021-07-12
EPI_ISL_5676834	RHD XV	RHD XV	2021-07-12
EPI_ISL_5676848	RHD XV	RHD XV	2021-07-12
EPI_ISL_5676849	RHD XV	RHD XV	2021-07-12
EPI_ISL_5676853	RHD XV	RHD XV	2021-07-12
EPI_ISL_5676855	RHD XV	RHD XV	2021-07-12
EPI_ISL_5676865	RHD XV	RHD XV	2021-07-12
EPI_ISL_5676939	RHD XV	RHD XV	2021-07-12
EPI_ISL_5677013	RHD XV	RHD XV	2021-07-12
EPI_ISL_5677044	RHD XV	RHD XV	2021-07-12
EPI_ISL_5677126	RHD XV	RHD XV	2021-07-12
EPI_ISL_5677156	RHD XV	RHD XV	2021-07-12
EPI_ISL_5677164	RHD XV	RHD XV	2021-07-12
EPI_ISL_5677214	RHD XV	RHD XV	2021-07-12
EPI_ISL_5677220	RHD XV	RHD XV	2021-07-12
EPI_ISL_5677241	RHD XV	RHD XV	2021-07-12
EPI_ISL_5677292	RHD XV	RHD XV	2021-07-12
EPI_ISL_5677294	RHD XV	RHD XV	2021-07-12
EPI_ISL_5677355	RHD XV	RHD XV	2021-07-12
EPI_ISL_5677397	RHD XV	RHD XV	2021-07-12
EPI_ISL_5677496	RHD XV	RHD XV	2021-07-12
EPI_ISL_5677513	RHD XV	RHD XV	2021-07-12
EPI_ISL_5677539	RHD XV	RHD XV	2021-07-12
EPI_ISL_5677591	RHD XV	RHD XV	2021-07-12
EPI_ISL_5677596	RHD XV	RHD XV	2021-07-12
EPI_ISL_5677602	RHD XV	RHD XV	2021-07-12
EPI_ISL_5677632	South	RHD XV	2021-07-12
EPI_ISL_5677640	RHD XV	RHD XV	2021-07-12
EPI_ISL_5677680	RHD XV	RHD XV	2021-07-12
EPI_ISL_5677690	RHD XV	RHD XV	2021-07-12
EPI_ISL_5677698	RHD XV	RHD XV	2021-07-12
EPI_ISL_5677716	RHD XV	RHD XV	2021-07-12
EPI_ISL_5677719	RHD XV	RHD XV	2021-07-12
EPI_ISL_5677730	RHD XV	RHD XV	2021-07-12
EPI_ISL_5678004	RHD XV	RHD XV	2021-07-12
EPI_ISL_5678082	RHD XV	RHD XV	2021-07-12
EPI_ISL_5678358	RHD XV	RHD XV	2021-07-12
EPI_ISL_5678549	RHD XV	RHD XV	2021-07-12
EPI_ISL_5678590	RHD XV	RHD XV	2021-07-12
EPI_ISL_5678641	RHD XV	RHD XV	2021-07-12
EPI_ISL_5678672	RHD XV	RHD XV	2021-07-12
EPI_ISL_5678759	RHD XV	RHD XV	2021-07-12
EPI_ISL_5678791	RHD XV	RHD XV	2021-07-12
EPI_ISL_5678843	RHD XV	RHD XV	2021-07-12
EPI_ISL_5678874	SJdRP	RHD XV	2021-07-12
EPI_ISL_5678888	RHD XV	RHD XV	2021-07-12
EPI_ISL_5678947	RHD XV	RHD XV	2021-07-12
EPI_ISL_5679027	RHD XV	RHD XV	2021-07-12
EPI_ISL_5679073	RHD XV	RHD XV	2021-07-12

EPI_ISL_5679078	RHD XV	RHD XV	2021-07-12
EPI_ISL_5679162	RHD XV	RHD XV	2021-07-12
EPI_ISL_5679546	RHD XV	RHD XV	2021-07-12
EPI_ISL_5679647	RHD XV	RHD XV	2021-07-13
EPI_ISL_5679742	RHD XV	RHD XV	2021-07-12
EPI_ISL_5679751	RHD XV	RHD XV	2021-07-12
EPI_ISL_5679792	Northeast	RHD XV	2021-07-12
EPI_ISL_5679863	RHD XV	RHD XV	2021-07-12
EPI_ISL_5679987	RHD XV	RHD XV	2021-07-12
EPI_ISL_5680023	RHD XV	RHD XV	2021-07-12
EPI_ISL_5680027	RHD XV	RHD XV	2021-07-12
EPI_ISL_5680060	RHD XV	RHD XV	2021-07-12
EPI_ISL_5680098	RHD XV	RHD XV	2021-07-12
EPI_ISL_5680114	RHD XV	RHD XV	2021-07-12
EPI_ISL_5680120	RHD XV	RHD XV	2021-07-12
EPI_ISL_5680124	RHD XV	RHD XV	2021-07-12
EPI_ISL_5680127	RHD XV	RHD XV	2021-07-12
EPI_ISL_5680139	RHD XV	RHD XV	2021-07-12
EPI_ISL_5680146	RHD XV	RHD XV	2021-07-12
EPI_ISL_5680156	RHD XV	RHD XV	2021-07-12
EPI_ISL_5680165	RHD XV	RHD XV	2021-07-12
EPI_ISL_5680172	RHD XV	RHD XV	2021-07-12
EPI_ISL_5680182	RHD XV	RHD XV	2021-07-12
EPI_ISL_5680189	RHD XV	RHD XV	2021-07-12
EPI_ISL_5680195	RHD XV	RHD XV	2021-07-12
EPI_ISL_5680202	RHD XV	RHD XV	2021-07-12
EPI_ISL_5680214	RHD XV	RHD XV	2021-07-12
EPI_ISL_5680218	RHD XV	RHD XV	2021-07-12
EPI_ISL_5680223	RHD XV	RHD XV	2021-07-12
EPI_ISL_5653794	RHD XV	RHD XV	2021-07-19
EPI_ISL_5651828	RHD XV	RHD XV	2021-07-19
EPI_ISL_5651841	RHD XV	RHD XV	2021-07-19
EPI_ISL_5651847	RHD XV	RHD XV	2021-07-19
EPI_ISL_5651855	RHD XV	RHD XV	2021-07-19
EPI_ISL_5651866	RHD XV	RHD XV	2021-07-19
EPI_ISL_5651917	RHD XV	RHD XV	2021-07-19
EPI_ISL_5650469	RHD XV	RHD XV	2021-07-19
EPI_ISL_5650433	RHD XV	RHD XV	2021-07-19
EPI_ISL_5651920	RHD XV	RHD XV	2021-07-19
EPI_ISL_5650652	RHD XV	RHD XV	2021-07-19
EPI_ISL_5650668	RHD XV	RHD XV	2021-07-20
EPI_ISL_5651921	RHD XV	RHD XV	2021-07-19
EPI_ISL_5651922	RHD XV	RHD XV	2021-07-19
EPI_ISL_5651924	RHD XV	RHD XV	2021-07-20
EPI_ISL_5651925	RHD XV	RHD XV	2021-07-20
EPI_ISL_5651929	RHD XV	RHD XV	2021-07-20
EPI_ISL_5651930	SJdRP	RHD XV	2021-07-19
EPI_ISL_5651931	RHD XV	RHD XV	2021-07-19
EPI_ISL_5651932	RHD XV	RHD XV	2021-07-19
EPI_ISL_5650523	RHD XV	RHD XV	2021-07-20
EPI_ISL_5651935	RHD XV	RHD XV	2021-07-20
EPI_ISL_5651936	RHD XV	RHD XV	2021-07-19
EPI_ISL_5651937	RHD XV	RHD XV	2021-07-19
EPI_ISL_5651938	RHD XV	RHD XV	2021-07-19
EPI_ISL_5651939	RHD XV	RHD XV	2021-07-20
EPI_ISL_5650427	RHD XV	RHD XV	2021-07-20
EPI_ISL_5651941	RHD XV	RHD XV	2021-07-19
EPI_ISL_5651945	RHD XV	RHD XV	2021-07-20
EPI_ISL_5651948	RHD XV	RHD XV	2021-07-19
EPI_ISL_5651949	RHD XV	RHD XV	2021-07-19
EPI_ISL_5651950	RHD XV	RHD XV	2021-07-19
EPI_ISL_5651952	RHD XV	RHD XV	2021-07-19
EPI_ISL_5651954	RHD XV	RHD XV	2021-07-20

EPI_ISL_5651955	RHD XV	RHD XV	2021-07-19
EPI_ISL_5651956	RHD XV	RHD XV	2021-07-20
EPI_ISL_5651958	RHD XV	RHD XV	2021-07-19
EPI_ISL_5651961	RHD XV	RHD XV	2021-07-19
EPI_ISL_5650430	RHD XV	RHD XV	2021-07-19
EPI_ISL_5651963	RHD XV	RHD XV	2021-07-20
EPI_ISL_5651966	Southeast	RHD XV	2021-07-19
EPI_ISL_5651968	RHD XV	RHD XV	2021-07-19
EPI_ISL_5651969	RHD XV	RHD XV	2021-07-19
EPI_ISL_5651970	RHD XV	RHD XV	2021-07-19
EPI_ISL_5651971	RHD XV	RHD XV	2021-07-19
EPI_ISL_5651972	RHD XV	RHD XV	2021-07-19
EPI_ISL_5650500	RHD XV	RHD XV	2021-07-19
EPI_ISL_5651974	RHD XV	RHD XV	2021-07-19
EPI_ISL_5651975	RHD XV	RHD XV	2021-07-19
EPI_ISL_5651976	RHD XV	RHD XV	2021-07-19
EPI_ISL_5651978	RHD XV	RHD XV	2021-07-19
EPI_ISL_5651980	RHD XV	RHD XV	2021-07-19
EPI_ISL_5651982	RHD XV	RHD XV	2021-07-19
EPI_ISL_5651983	RHD XV	RHD XV	2021-07-19
EPI_ISL_5651985	RHD XV	RHD XV	2021-07-19
EPI_ISL_5651986	RHD XV	RHD XV	2021-07-19
EPI_ISL_5655103	RHD XV	RHD XV	2021-07-19
EPI_ISL_5655042	RHD XV	RHD XV	2021-07-19
EPI_ISL_5654099	RHD XV	RHD XV	2021-07-19
EPI_ISL_5652012	RHD XV	RHD XV	2021-07-19
EPI_ISL_5652016	RHD XV	RHD XV	2021-07-19
EPI_ISL_5652017	RHD XV	RHD XV	2021-07-19
EPI_ISL_5652019	RHD XV	RHD XV	2021-07-19
EPI_ISL_5653868	RHD XV	RHD XV	2021-07-19
EPI_ISL_5652020	SJdRP	RHD XV	2021-07-19
EPI_ISL_5652021	RHD XV	RHD XV	2021-07-17
EPI_ISL_5652022	North	RHD XV	2021-07-19
EPI_ISL_5650451	RHD XV	RHD XV	2021-07-19
EPI_ISL_5650597	RHD XV	RHD XV	2021-07-19
EPI_ISL_5650598	RHD XV	RHD XV	2021-07-19
EPI_ISL_5652025	RHD XV	RHD XV	2021-07-19
EPI_ISL_5652026	RHD XV	RHD XV	2021-07-19
EPI_ISL_5650599	RHD XV	RHD XV	2021-07-19
EPI_ISL_5652028	RHD XV	RHD XV	2021-07-19
EPI_ISL_5652029	RHD XV	RHD XV	2021-07-19
EPI_ISL_5652030	RHD XV	RHD XV	2021-07-19
EPI_ISL_5652031	RHD XV	RHD XV	2021-07-19
EPI_ISL_5652033	RHD XV	RHD XV	2021-07-19
EPI_ISL_5652034	RHD XV	RHD XV	2021-07-19
EPI_ISL_5652035	RHD XV	RHD XV	2021-07-19
EPI_ISL_5652038	RHD XV	RHD XV	2021-07-19
EPI_ISL_5650600	RHD XV	RHD XV	2021-07-19
EPI_ISL_5650452	RHD XV	RHD XV	2021-07-19
EPI_ISL_5650653	RHD XV	RHD XV	2021-07-19
EPI_ISL_5650453	RHD XV	RHD XV	2021-07-19
EPI_ISL_5650650	RHD XV	RHD XV	2021-07-19
EPI_ISL_5652047	RHD XV	RHD XV	2021-07-19
EPI_ISL_5652048	RHD XV	RHD XV	2021-07-19
EPI_ISL_5652050	RHD XV	RHD XV	2021-07-19
EPI_ISL_5652051	RHD XV	RHD XV	2021-07-19
EPI_ISL_5652056	RHD XV	RHD XV	2021-07-20
EPI_ISL_5652057	RHD XV	RHD XV	2021-07-19
EPI_ISL_5654212	RHD XV	RHD XV	2021-07-19
EPI_ISL_5652060	SJdRP	RHD XV	2021-07-19
EPI_ISL_5652061	RHD XV	RHD XV	2021-07-19
EPI_ISL_5652063	RHD XV	RHD XV	2021-07-19
EPI_ISL_5652064	RHD XV	RHD XV	2021-07-19

EPI_ISL_5652065	RHD XV	RHD XV	2021-07-19
EPI_ISL_5652066	RHD XV	RHD XV	2021-07-19
EPI_ISL_5652067	RHD XV	RHD XV	2021-07-19
EPI_ISL_5652069	RHD XV	RHD XV	2021-07-19
EPI_ISL_5652070	RHD XV	RHD XV	2021-07-19
EPI_ISL_5652071	RHD XV	RHD XV	2021-07-19
EPI_ISL_5652072	RHD XV	RHD XV	2021-07-19
EPI_ISL_5652073	RHD XV	RHD XV	2021-07-19
EPI_ISL_5652074	RHD XV	RHD XV	2021-07-19
EPI_ISL_5653764	RHD XV	RHD XV	2021-07-26
EPI_ISL_5654905	RHD XV	RHD XV	2021-07-27
EPI_ISL_5654855	RHD XV	RHD XV	2021-07-26
EPI_ISL_5654727	RHD XV	RHD XV	2021-07-26
EPI_ISL_5654439	RHD XV	RHD XV	2021-07-26
EPI_ISL_5652086	RHD XV	RHD XV	2021-07-26
EPI_ISL_5652090	RHD XV	RHD XV	2021-07-26
EPI_ISL_5652092	RHD XV	RHD XV	2021-07-25
EPI_ISL_5650601	RHD XV	RHD XV	2021-07-26
EPI_ISL_5652095	RHD XV	RHD XV	2021-07-26
EPI_ISL_5650556	RHD XV	RHD XV	2021-07-26
EPI_ISL_5653869	RHD XV	RHD XV	2021-07-26
EPI_ISL_5652098	RHD XV	RHD XV	2021-07-25
EPI_ISL_5652103	RHD XV	RHD XV	2021-07-25
EPI_ISL_5652105	RHD XV	RHD XV	2021-07-25
EPI_ISL_5652129	RHD XV	RHD XV	2021-07-26
EPI_ISL_5650635	RHD XV	RHD XV	2021-07-26
EPI_ISL_5652139	Southeast	RHD XV	2021-07-25
EPI_ISL_5652142	RHD XV	RHD XV	2021-07-26
EPI_ISL_5652150	RHD XV	RHD XV	2021-07-26
EPI_ISL_5652158	RHD XV	RHD XV	2021-07-26
EPI_ISL_5652163	RHD XV	RHD XV	2021-07-26
EPI_ISL_5652167	RHD XV	RHD XV	2021-07-26
EPI_ISL_5652168	RHD XV	RHD XV	2021-07-25
EPI_ISL_5652170	RHD XV	RHD XV	2021-07-26
EPI_ISL_5652171	RHD XV	RHD XV	2021-07-26
EPI_ISL_5652177	RHD XV	RHD XV	2021-07-26
EPI_ISL_5652179	RHD XV	RHD XV	2021-07-26
EPI_ISL_5652181	RHD XV	RHD XV	2021-07-27
EPI_ISL_5652185	RHD XV	RHD XV	2021-07-26
EPI_ISL_5652194	RHD XV	RHD XV	2021-07-26
EPI_ISL_5652209	RHD XV	RHD XV	2021-07-27
EPI_ISL_5652217	RHD XV	RHD XV	2021-07-26
EPI_ISL_5652218	RHD XV	RHD XV	2021-07-27
EPI_ISL_5652219	RHD XV	RHD XV	2021-07-26
EPI_ISL_5652220	RHD XV	RHD XV	2021-07-26
EPI_ISL_5652224	RHD XV	RHD XV	2021-07-26
EPI_ISL_5652247	RHD XV	RHD XV	2021-07-26
EPI_ISL_5652255	RHD XV	RHD XV	2021-07-26
EPI_ISL_5652258	RHD XV	RHD XV	2021-07-26
EPI_ISL_5652263	RHD XV	RHD XV	2021-07-27
EPI_ISL_5652269	RHD XV	RHD XV	2021-07-26
EPI_ISL_5652272	RHD XV	RHD XV	2021-07-26
EPI_ISL_5655121	RHD XV	RHD XV	2021-07-26
EPI_ISL_5655111	RHD XV	RHD XV	2021-07-26
EPI_ISL_5655101	RHD XV	RHD XV	2021-07-27
EPI_ISL_5652282	RHD XV	RHD XV	2021-07-27
EPI_ISL_5652284	RHD XV	RHD XV	2021-07-26
EPI_ISL_5652286	RHD XV	RHD XV	2021-07-26
EPI_ISL_5652296	RHD XV	RHD XV	2021-07-27
EPI_ISL_5652307	RHD XV	RHD XV	2021-07-26
EPI_ISL_5652310	RHD XV	RHD XV	2021-07-26
EPI_ISL_5652311	RHD XV	RHD XV	2021-07-26
EPI_ISL_5652312	RHD XV	RHD XV	2021-07-26

EPI_ISL_5652313	RHD XV	RHD XV	2021-07-26
EPI_ISL_5652314	RHD XV	RHD XV	2021-07-26
EPI_ISL_5653801	RHD XV	RHD XV	2021-07-26
EPI_ISL_5655087	RHD XV	RHD XV	2021-07-26
EPI_ISL_5655071	RHD XV	RHD XV	2021-07-26
EPI_ISL_5654863	RHD XV	RHD XV	2021-07-26
EPI_ISL_5652319	RHD XV	RHD XV	2021-07-26
EPI_ISL_5652320	RHD XV	RHD XV	2021-07-26
EPI_ISL_5652330	RHD XV	RHD XV	2021-07-26
EPI_ISL_5650637	RHD XV	RHD XV	2021-07-26
EPI_ISL_5652343	RHD XV	RHD XV	2021-07-26
EPI_ISL_5650454	RHD XV	RHD XV	2021-07-26
EPI_ISL_5650605	RHD XV	RHD XV	2021-07-26
EPI_ISL_5652368	RHD XV	RHD XV	2021-07-26
EPI_ISL_5652375	RHD XV	RHD XV	2021-07-26
EPI_ISL_5652376	RHD XV	RHD XV	2021-07-26
EPI_ISL_5652377	RHD XV	RHD XV	2021-07-26
EPI_ISL_5652381	RHD XV	RHD XV	2021-07-26
EPI_ISL_5652398	RHD XV	RHD XV	2021-07-26
EPI_ISL_5650455	RHD XV	RHD XV	2021-07-26
EPI_ISL_5652413	RHD XV	RHD XV	2021-07-27
EPI_ISL_5650606	RHD XV	RHD XV	2021-07-26
EPI_ISL_5652457	RHD XV	RHD XV	2021-07-26
EPI_ISL_5652486	RHD XV	RHD XV	2021-07-26
EPI_ISL_5652487	RHD XV	RHD XV	2021-07-26
EPI_ISL_5652488	RHD XV	RHD XV	2021-07-26
EPI_ISL_5652492	RHD XV	RHD XV	2021-07-26
EPI_ISL_5652499	RHD XV	RHD XV	2021-07-26
EPI_ISL_5652504	RHD XV	RHD XV	2021-07-26
EPI_ISL_5652536	RHD XV	RHD XV	2021-07-27
EPI_ISL_5653871	RHD XV	RHD XV	2021-07-26
EPI_ISL_5652651	RHD XV	RHD XV	2021-07-26
EPI_ISL_5652753	RHD XV	RHD XV	2021-07-27
EPI_ISL_5652772	RHD XV	RHD XV	2021-07-27
EPI_ISL_5653633	RHD XV	RHD XV	2021-08-02
EPI_ISL_5654747	RHD XV	RHD XV	2021-08-03
EPI_ISL_5654733	RHD XV	RHD XV	2021-08-02
EPI_ISL_5654446	RHD XV	RHD XV	2021-08-02
EPI_ISL_5654107	RHD XV	RHD XV	2021-08-02
EPI_ISL_5654047	RHD XV	RHD XV	2021-08-02
EPI_ISL_5654124	RHD XV	RHD XV	2021-08-02
EPI_ISL_5654009	RHD XV	RHD XV	2021-08-02
EPI_ISL_5654011	RHD XV	RHD XV	2021-08-03
EPI_ISL_5654055	RHD XV	RHD XV	2021-08-02
EPI_ISL_5654022	RHD XV	RHD XV	2021-08-02
EPI_ISL_5653207	RHD XV	RHD XV	2021-08-02
EPI_ISL_5650457	RHD XV	RHD XV	2021-08-02
EPI_ISL_5650458	RHD XV	RHD XV	2021-08-02
EPI_ISL_5653215	RHD XV	RHD XV	2021-08-01
EPI_ISL_5650459	RHD XV	RHD XV	2021-08-02
EPI_ISL_5650587	RHD XV	RHD XV	2021-08-02
EPI_ISL_5653219	RHD XV	RHD XV	2021-08-01
EPI_ISL_5653223	RHD XV	RHD XV	2021-08-03
EPI_ISL_5653228	RHD XV	RHD XV	2021-08-01
EPI_ISL_5653244	SJdRP	RHD XV	2021-08-02
EPI_ISL_5653245	RHD XV	RHD XV	2021-08-02
EPI_ISL_5653254	RHD XV	RHD XV	2021-08-02
EPI_ISL_5653255	RHD XV	RHD XV	2021-08-03
EPI_ISL_5653266	RHD XV	RHD XV	2021-08-02
EPI_ISL_5653271	RHD XV	RHD XV	2021-08-03
EPI_ISL_5650503	RHD XV	RHD XV	2021-08-02
EPI_ISL_5653272	RHD XV	RHD XV	2021-08-02
EPI_ISL_5650448	RHD XV	RHD XV	2021-08-02

EPI_ISL_5653278	RHD XV	RHD XV	2021-08-03
EPI_ISL_5653280	RHD XV	RHD XV	2021-08-02
EPI_ISL_5653281	Northeast	RHD XV	2021-08-02
EPI_ISL_5653284	RHD XV	RHD XV	2021-08-02
EPI_ISL_5653286	RHD XV	RHD XV	2021-08-02
EPI_ISL_5653292	RHD XV	RHD XV	2021-08-02
EPI_ISL_5653316	RHD XV	RHD XV	2021-08-03
EPI_ISL_5653317	RHD XV	RHD XV	2021-08-02
EPI_ISL_5653318	RHD XV	RHD XV	2021-08-02
EPI_ISL_5653323	RHD XV	RHD XV	2021-08-03
EPI_ISL_5650460	RHD XV	RHD XV	2021-08-02
EPI_ISL_5653332	RHD XV	RHD XV	2021-08-02
EPI_ISL_5653334	RHD XV	RHD XV	2021-08-02
EPI_ISL_5653345	RHD XV	RHD XV	2021-08-01
EPI_ISL_5653347	RHD XV	RHD XV	2021-08-01
EPI_ISL_5653348	RHD XV	RHD XV	2021-08-02
EPI_ISL_5650461	RHD XV	RHD XV	2021-08-02
EPI_ISL_5653354	RHD XV	RHD XV	2021-08-02
EPI_ISL_5650612	RHD XV	RHD XV	2021-08-02
EPI_ISL_5653358	RHD XV	RHD XV	2021-08-02
EPI_ISL_5653360	RHD XV	RHD XV	2021-08-02
EPI_ISL_5653362	Southeast	RHD XV	2021-08-02
EPI_ISL_5650462	RHD XV	RHD XV	2021-08-02
EPI_ISL_5653367	RHD XV	RHD XV	2021-08-02
EPI_ISL_5653377	RHD XV	RHD XV	2021-08-02
EPI_ISL_5650613	RHD XV	RHD XV	2021-08-02
EPI_ISL_5650588	RHD XV	RHD XV	2021-08-02
EPI_ISL_5653380	RHD XV	RHD XV	2021-08-02
EPI_ISL_5653385	SJdRP	RHD XV	2021-08-02
EPI_ISL_5650614	RHD XV	RHD XV	2021-08-02
EPI_ISL_5653389	Southeast	RHD XV	2021-08-03
EPI_ISL_5653401	RHD XV	RHD XV	2021-08-01
EPI_ISL_5653408	RHD XV	RHD XV	2021-08-02
EPI_ISL_5653410	RHD XV	RHD XV	2021-08-02
EPI_ISL_5653416	RHD XV	RHD XV	2021-08-02
EPI_ISL_5653417	RHD XV	RHD XV	2021-08-02
EPI_ISL_5653418	SJdRP	RHD XV	2021-08-02
EPI_ISL_5653420	RHD XV	RHD XV	2021-08-01
EPI_ISL_5653422	RHD XV	RHD XV	2021-08-02
EPI_ISL_5653818	RHD XV	RHD XV	2021-08-02
EPI_ISL_5655036	RHD XV	RHD XV	2021-08-02
EPI_ISL_5654706	RHD XV	RHD XV	2021-08-02
EPI_ISL_5654238	RHD XV	RHD XV	2021-08-02
EPI_ISL_5654037	RHD XV	RHD XV	2021-08-02
EPI_ISL_5653433	RHD XV	RHD XV	2021-08-02
EPI_ISL_5653435	RHD XV	RHD XV	2021-08-02
EPI_ISL_5653441	RHD XV	RHD XV	2021-08-02
EPI_ISL_5653444	RHD XV	RHD XV	2021-08-02
EPI_ISL_5653448	RHD XV	RHD XV	2021-08-02
EPI_ISL_5653453	RHD XV	RHD XV	2021-08-02
EPI_ISL_5653458	RHD XV	RHD XV	2021-08-02
EPI_ISL_5653461	RHD XV	RHD XV	2021-08-02
EPI_ISL_5653465	RHD XV	RHD XV	2021-08-02
EPI_ISL_5653466	Midwest	RHD XV	2021-08-02
EPI_ISL_5653467	RHD XV	RHD XV	2021-08-02
EPI_ISL_5653469	RHD XV	RHD XV	2021-08-02
EPI_ISL_5653473	RHD XV	RHD XV	2021-08-02
EPI_ISL_5653474	RHD XV	RHD XV	2021-08-03
EPI_ISL_3579312	RHD XV	RHD XV	2021-08-02
EPI_ISL_3579343	SJdRP	RHD XV	2021-08-02
EPI_ISL_3944899	SJdRP	RHD XV	2021-08-16
EPI_ISL_3944866	SJdRP	RHD XV	2021-08-16
EPI_ISL_4168740	RHD XV	RHD XV	2021-08-23

EPI_ISL_4168738	RHD XV	RHD XV	2021-08-23
EPI_ISL_4168706	Southeast	RHD XV	2021-08-23
EPI_ISL_4468889	SJdRP	SJdRP	2021-08-25
EPI_ISL_4468887	SJdRP	SJdRP	2021-08-25
EPI_ISL_4468886	SJdRP	SJdRP	2021-08-25
EPI_ISL_4468885	SJdRP	SJdRP	2021-08-25
EPI_ISL_4468884	SJdRP	SJdRP	2021-08-24
EPI_ISL_4468883	SJdRP	SJdRP	2021-08-24
EPI_ISL_4468882	SJdRP	SJdRP	2021-08-23
EPI_ISL_4468880	SJdRP	SJdRP	2021-08-23
EPI_ISL_4468879	SJdRP	SJdRP	2021-08-23
EPI_ISL_4468878	SJdRP	SJdRP	2021-08-23
EPI_ISL_4468877	SJdRP	SJdRP	2021-08-23
EPI_ISL_4468891	SJdRP	RHD XV	2021-08-25
EPI_ISL_4468890	SJdRP	SJdRP	2021-08-25
EPI_ISL_4468888	SJdRP	SJdRP	2021-08-25
EPI_ISL_4468881	SJdRP	SJdRP	2021-08-23
EPI_ISL_4275661	SJdRP	SJdRP	2021-09-01
EPI_ISL_4275660	SJdRP	SJdRP	2021-09-01
EPI_ISL_4275659	SJdRP	SJdRP	2021-09-01
EPI_ISL_4275658	Southeast	SJdRP	2021-09-01
EPI_ISL_4275657	SJdRP	SJdRP	2021-09-01
EPI_ISL_4275656	SJdRP	SJdRP	2021-09-01
EPI_ISL_4275655	SJdRP	SJdRP	2021-09-01
EPI_ISL_4275654	SJdRP	SJdRP	2021-09-01
EPI_ISL_4275645	RHD XV	RHD XV	2021-08-31
EPI_ISL_4273252	Southeast	SJdRP	2021-08-30
EPI_ISL_4275644	RHD XV	RHD XV	2021-08-30
EPI_ISL_4275650	SJdRP	SJdRP	2021-08-31
EPI_ISL_4275653	SJdRP	SJdRP	2021-08-31
EPI_ISL_4275651	SJdRP	SJdRP	2021-08-31
EPI_ISL_4275652	SJdRP	SJdRP	2021-08-31
EPI_ISL_4275649	SJdRP	SJdRP	2021-08-31
EPI_ISL_4275648	SJdRP	SJdRP	2021-08-31
EPI_ISL_4275646	SJdRP	SJdRP	2021-08-31
EPI_ISL_4275643	SJdRP	SJdRP	2021-08-30
EPI_ISL_4275647	SJdRP	SJdRP	2021-08-31
EPI_ISL_4275642	SJdRP	RHD XV	2021-08-30
EPI_ISL_4275641	RHD XV	RHD XV	2021-08-31
EPI_ISL_4275640	RHD XV	RHD XV	2021-08-31
EPI_ISL_4275635	SJdRP	RHD XV	2021-08-31
EPI_ISL_4275629	RHD XV	RHD XV	2021-08-31
EPI_ISL_4275628	RHD XV	RHD XV	2021-09-01
EPI_ISL_4275627	RHD XV	RHD XV	2021-09-01
EPI_ISL_4275617	RHD XV	RHD XV	2021-08-31
EPI_ISL_4275610	RHD XV	RHD XV	2021-08-31
EPI_ISL_4275593	RHD XV	RHD XV	2021-08-31
EPI_ISL_4275592	RHD XV	RHD XV	2021-08-31
EPI_ISL_4746615	SJdRP	SJdRP	2021-09-07
EPI_ISL_4746841	SJdRP	SJdRP	2021-09-07
EPI_ISL_4746454	SJdRP	SJdRP	2021-09-08
EPI_ISL_4746785	SJdRP	SJdRP	2021-09-08
EPI_ISL_4746702	SJdRP	SJdRP	2021-09-08
EPI_ISL_4746595	SJdRP	SJdRP	2021-09-08
EPI_ISL_4746840	SJdRP	SJdRP	2021-09-08
EPI_ISL_4746839	Northeast	SJdRP	2021-09-04
EPI_ISL_4746838	SJdRP	SJdRP	2021-09-04
EPI_ISL_4746705	South	SJdRP	2021-09-08
EPI_ISL_4746449	SJdRP	SJdRP	2021-09-08
EPI_ISL_4746679	SJdRP	SJdRP	2021-09-08
EPI_ISL_4746786	SJdRP	SJdRP	2021-09-05
EPI_ISL_4746534	SJdRP	SJdRP	2021-09-05
EPI_ISL_4746692	South	SJdRP	2021-09-05

EPI_ISL_4746712	Northeast	RHD XV	2021-09-08
EPI_ISL_4746703	RHD XV	RHD XV	2021-09-06
EPI_ISL_4746480	RHD XV	RHD XV	2021-09-07
EPI_ISL_4747285	RHD XV	RHD XV	2021-09-07
EPI_ISL_4747284	RHD XV	RHD XV	2021-09-07
EPI_ISL_4746732	RHD XV	RHD XV	2021-09-08
EPI_ISL_4747281	RHD XV	RHD XV	2021-09-06
EPI_ISL_4747280	RHD XV	RHD XV	2021-09-08
EPI_ISL_4746447	SJdRP	RHD XV	2021-09-07
EPI_ISL_4746633	Southeast	RHD XV	2021-09-06
EPI_ISL_4746726	RHD XV	RHD XV	2021-09-05
EPI_ISL_5689107	RHD XV	RHD XV	2021-09-27
EPI_ISL_5689106	Midwest	RHD XV	2021-09-27
EPI_ISL_5689266	RHD XV	RHD XV	2021-09-27
EPI_ISL_5689136	SJdRP	RHD XV	2021-09-27
EPI_ISL_5689137	RHD XV	RHD XV	2021-09-27
EPI_ISL_5688395	RHD XV	RHD XV	2021-09-27
EPI_ISL_5688458	RHD XV	RHD XV	2021-09-28
EPI_ISL_5688468	RHD XV	RHD XV	2021-09-28
EPI_ISL_5688470	RHD XV	RHD XV	2021-09-28
EPI_ISL_5689259	SJdRP	RHD XV	2021-09-27
EPI_ISL_5688526	Southeast	RHD XV	2021-09-27
EPI_ISL_5688641	RHD XV	RHD XV	2021-09-27
EPI_ISL_5688651	RHD XV	RHD XV	2021-09-27
EPI_ISL_5898584	RHD XV	RHD XV	2021-10-04
EPI_ISL_5898585	RHD XV	RHD XV	2021-10-03
EPI_ISL_5898586	RHD XV	RHD XV	2021-10-04
EPI_ISL_5898772	SJdRP	RHD XV	2021-10-05
EPI_ISL_5898815	Northeast	RHD XV	2021-10-04
EPI_ISL_5898840	RHD XV	RHD XV	2021-10-04
EPI_ISL_5898881	RHD XV	RHD XV	2021-10-04
EPI_ISL_5898884	RHD XV	RHD XV	2021-10-04
EPI_ISL_5898888	RHD XV	RHD XV	2021-10-04
EPI_ISL_6509075	RHD XV	RHD XV	2021-10-12
EPI_ISL_6508692	RHD XV	RHD XV	2021-10-12
EPI_ISL_6508722	SJdRP	RHD XV	2021-10-13
EPI_ISL_6508724	RHD XV	RHD XV	2021-10-13
EPI_ISL_6508730	SJdRP	RHD XV	2021-10-13
EPI_ISL_6509937	North	SJdRP	2021-10-13
EPI_ISL_6509911	SJdRP	SJdRP	2021-10-11
EPI_ISL_6510558	SJdRP	SJdRP	2021-10-18
EPI_ISL_6510552	SJdRP	SJdRP	2021-10-18
EPI_ISL_6510529	SJdRP	SJdRP	2021-10-11
EPI_ISL_6509837	RHD XV	SJdRP	2021-10-11
EPI_ISL_6509900	RHD XV	RHD XV	2021-10-18
EPI_ISL_6509972	SJdRP	SJdRP	2021-10-18
EPI_ISL_6509913	RHD XV	RHD XV	2021-10-18
EPI_ISL_6509908	SJdRP	SJdRP	2021-10-18
EPI_ISL_6509891	SJdRP	SJdRP	2021-10-18
EPI_ISL_6510572	SJdRP	SJdRP	2021-10-18
EPI_ISL_6509905	SJdRP	SJdRP	2021-10-12
EPI_ISL_6510557	SJdRP	SJdRP	2021-10-12
EPI_ISL_6509912	SJdRP	SJdRP	2021-10-11
EPI_ISL_6510454	SJdRP	SJdRP	2021-10-18
EPI_ISL_6510508	SJdRP	SJdRP	2021-10-18
EPI_ISL_6510504	SJdRP	SJdRP	2021-10-18
EPI_ISL_6510496	SJdRP	SJdRP	2021-10-12
EPI_ISL_6509592	SJdRP	SJdRP	2021-10-11
EPI_ISL_6509951	North	SJdRP	2021-10-11
EPI_ISL_6510567	SJdRP	SJdRP	2021-10-11
EPI_ISL_6510480	Northeast	SJdRP	2021-10-18
EPI_ISL_6510509	SJdRP	SJdRP	2021-10-18
EPI_ISL_6510489	SJdRP	SJdRP	2021-10-18

EPI_ISL_6510488	SJdRP	SJdRP	2021-10-18
EPI_ISL_6510447	SJdRP	SJdRP	2021-10-12
EPI_ISL_6510456	SJdRP	SJdRP	2021-10-11
EPI_ISL_6510517	SJdRP	SJdRP	2021-10-11
EPI_ISL_6510486	SJdRP	SJdRP	2021-10-11
EPI_ISL_6509904	SJdRP	SJdRP	2021-10-11
EPI_ISL_6509857	RHD XV	RHD XV	2021-10-18
EPI_ISL_6509882	RHD XV	RHD XV	2021-10-18
EPI_ISL_6509766	RHD XV	RHD XV	2021-10-18
EPI_ISL_6570076	RHD XV	RHD XV	2021-10-27
EPI_ISL_6570455	RHD XV	RHD XV	2021-10-26
EPI_ISL_6570078	RHD XV	RHD XV	2021-10-26
EPI_ISL_6570106	Southeast	RHD XV	2021-10-26
EPI_ISL_6570107	Southeast	RHD XV	2021-10-26
EPI_ISL_6813370	SJdRP	RHD XV	2021-11-01
EPI_ISL_6813261	RHD XV	RHD XV	2021-11-02
EPI_ISL_6813614	Northeast	RHD XV	2021-11-03
EPI_ISL_6813376	Northeast	RHD XV	2021-11-01
EPI_ISL_6894178	SJdRP	SJdRP	2021-10-27
EPI_ISL_6894187	SJdRP	SJdRP	2021-10-27
EPI_ISL_6894190	SJdRP	SJdRP	2021-10-27
EPI_ISL_6894196	SJdRP	SJdRP	2021-10-27
EPI_ISL_6894203	SJdRP	SJdRP	2021-10-27
EPI_ISL_6894204	SJdRP	RHD XV	2021-10-27
EPI_ISL_6894210	SJdRP	SJdRP	2021-10-26
EPI_ISL_6894214	SJdRP	SJdRP	2021-10-27
EPI_ISL_6894219	SJdRP	SJdRP	2021-10-25
EPI_ISL_6894226	SJdRP	SJdRP	2021-10-25
EPI_ISL_6894231	RHD XV	RHD XV	2021-10-23
EPI_ISL_6894232	RHD XV	RHD XV	2021-10-23
EPI_ISL_6894238	Southeast	RHD XV	2021-10-23
EPI_ISL_6894242	RHD XV	RHD XV	2021-10-25
EPI_ISL_6894246	SJdRP	SJdRP	2021-10-25
EPI_ISL_6894252	SJdRP	SJdRP	2021-10-26
EPI_ISL_6896380	RHD XV	RHD XV	2021-11-08
EPI_ISL_7132225	SJdRP	RHD XV	2021-11-16
EPI_ISL_7132711	Southeast	SJdRP	2021-11-20
EPI_ISL_7132480	RHD XV	RHD XV	2021-11-22
EPI_ISL_7132523	RHD XV	RHD XV	2021-11-19
EPI_ISL_7132482	SJdRP	SJdRP	2021-11-16
EPI_ISL_7132491	RHD XV	SJdRP	2021-11-21
EPI_ISL_7666520	SJdRP	SJdRP	2021-11-26
EPI_ISL_7666647	SJdRP	SJdRP	2021-11-26
EPI_ISL_7666486	SJdRP	SJdRP	2021-11-25
EPI_ISL_7666458	SJdRP	SJdRP	2021-11-28
EPI_ISL_7666541	SJdRP	SJdRP	2021-11-26
EPI_ISL_7666698	Midwest	RHD XV	2021-11-24
EPI_ISL_7666523	RHD XV	RHD XV	2021-11-24
EPI_ISL_7666603	SJdRP	SJdRP	2021-11-26
EPI_ISL_7666253	SJdRP	RHD XV	2021-11-23
EPI_ISL_7665166	RHD XV	RHD XV	2021-11-22
EPI_ISL_7665165	RHD XV	RHD XV	2021-11-22
EPI_ISL_7665139	SJdRP	RHD XV	2021-11-22
EPI_ISL_7809144	SJdRP	RHD XV	2021-11-29
EPI_ISL_7809145	RHD XV	RHD XV	2021-11-30
EPI_ISL_7809334	SJdRP	SJdRP	2021-11-30
EPI_ISL_7809172	RHD XV	SJdRP	2021-11-29
EPI_ISL_7809383	SJdRP	SJdRP	2021-11-29
EPI_ISL_7809386	SJdRP	SJdRP	2021-11-29
EPI_ISL_7899761	RHD XV	RHD XV	2021-12-08
EPI_ISL_8401504	South	RHD XV	2021-12-13
EPI_ISL_8401525	SJdRP	RHD XV	2021-12-13
EPI_ISL_8401538	Northeast	RHD XV	2021-12-13

EPI_ISL_9305175	SJdRP	SJdRP	2021-12-31
EPI_ISL_9305176	SJdRP	SJdRP	2021-12-31
EPI_ISL_9305178	SJdRP	SJdRP	2021-12-30
EPI_ISL_9305179	SJdRP	SJdRP	2021-12-30
EPI_ISL_9305180	SJdRP	SJdRP	2021-12-30
EPI_ISL_9305182	SJdRP	SJdRP	2021-12-30
EPI_ISL_9305183	SJdRP	SJdRP	2021-12-30
EPI_ISL_9305185	SJdRP	SJdRP	2022-01-01
EPI_ISL_9305186	SJdRP	SJdRP	2021-12-31
EPI_ISL_9305187	SJdRP	SJdRP	2021-12-31
EPI_ISL_9305188	RHD XV	SJdRP	2021-12-29
EPI_ISL_9304344	SJdRP	SJdRP	2021-12-31
EPI_ISL_9304397	SJdRP	SJdRP	2021-12-31
EPI_ISL_9304398	SJdRP	SJdRP	2021-12-31
EPI_ISL_9304399	SJdRP	SJdRP	2021-12-31
EPI_ISL_9304400	RHD XV	SJdRP	2021-12-31
EPI_ISL_9304401	SJdRP	SJdRP	2021-12-31
EPI_ISL_9304402	SJdRP	SJdRP	2021-12-31
EPI_ISL_9304403	RHD XV	SJdRP	2022-01-02
EPI_ISL_9304404	SJdRP	SJdRP	2022-01-02
EPI_ISL_9304405	SJdRP	SJdRP	2022-01-02
EPI_ISL_9304406	RHD XV	RHD XV	2022-01-03
EPI_ISL_9304407	RHD XV	RHD XV	2022-01-01
EPI_ISL_9304408	SJdRP	RHD XV	2022-01-02
EPI_ISL_9304409	RHD XV	RHD XV	2022-01-03
EPI_ISL_9304410	RHD XV	RHD XV	2022-01-03
EPI_ISL_9304411	RHD XV	RHD XV	2022-01-03
EPI_ISL_9304412	RHD XV	RHD XV	2022-01-03
EPI_ISL_9304413	SJdRP	RHD XV	2022-01-03
EPI_ISL_9304414	RHD XV	RHD XV	2022-01-03
EPI_ISL_9304415	RHD XV	RHD XV	2022-01-03
EPI_ISL_9304416	RHD XV	RHD XV	2022-01-04
EPI_ISL_9304418	RHD XV	RHD XV	2022-01-02
EPI_ISL_9304419	SJdRP	RHD XV	2022-01-02
EPI_ISL_9304421	RHD XV	RHD XV	2022-01-02
EPI_ISL_9304422	RHD XV	RHD XV	2022-01-03
EPI_ISL_9304423	RHD XV	RHD XV	2022-01-01
EPI_ISL_9304437	RHD XV	RHD XV	2022-01-03
EPI_ISL_9304438	RHD XV	RHD XV	2022-01-07
EPI_ISL_9304439	RHD XV	RHD XV	2022-01-06
EPI_ISL_9304442	RHD XV	RHD XV	2022-01-06
EPI_ISL_9304443	RHD XV	RHD XV	2022-01-06
EPI_ISL_9304444	RHD XV	RHD XV	2022-01-06
EPI_ISL_9304445	RHD XV	RHD XV	2022-01-06
EPI_ISL_9304446	SJdRP	RHD XV	2022-01-06
EPI_ISL_9304447	RHD XV	RHD XV	2022-01-06
EPI_ISL_9304449	RHD XV	RHD XV	2022-01-06
EPI_ISL_9304450	SJdRP	SJdRP	2022-01-09
EPI_ISL_9304452	SJdRP	SJdRP	2022-01-14
EPI_ISL_9304453	RHD XV	SJdRP	2022-01-14
EPI_ISL_9304454	RHD XV	SJdRP	2022-01-14
EPI_ISL_9304456	RHD XV	RHD XV	2022-01-10
EPI_ISL_9304458	RHD XV	RHD XV	2022-01-10
EPI_ISL_9304459	RHD XV	RHD XV	2022-01-10
EPI_ISL_9304460	RHD XV	RHD XV	2022-01-12
EPI_ISL_9304461	RHD XV	RHD XV	2022-01-13
EPI_ISL_9304462	RHD XV	RHD XV	2022-01-12
EPI_ISL_9304463	RHD XV	RHD XV	2022-01-12
EPI_ISL_9304465	SJdRP	RHD XV	2022-01-12
EPI_ISL_9304466	RHD XV	RHD XV	2022-01-12
EPI_ISL_9304468	RHD XV	RHD XV	2022-01-12
EPI_ISL_9304469	RHD XV	RHD XV	2022-01-12
EPI_ISL_9304470	SJdRP	RHD XV	2022-01-11

EPI_ISL_9304476	RHD XV	RHD XV	2022-01-12
EPI_ISL_9304477	SJdRP	RHD XV	2022-01-12
EPI_ISL_9304478	RHD XV	RHD XV	2022-01-12
EPI_ISL_9304479	RHD XV	RHD XV	2022-01-12
EPI_ISL_9304480	RHD XV	RHD XV	2022-01-12
EPI_ISL_9304481	RHD XV	RHD XV	2022-01-11
EPI_ISL_9304482	SJdRP	RHD XV	2022-01-11
EPI_ISL_9304486	RHD XV	RHD XV	2022-01-11
EPI_ISL_9304487	RHD XV	RHD XV	2022-01-11
EPI_ISL_9304488	RHD XV	RHD XV	2022-01-12
EPI_ISL_9304489	RHD XV	RHD XV	2022-01-12
EPI_ISL_9304492	SJdRP	RHD XV	2022-01-11
EPI_ISL_9304493	RHD XV	RHD XV	2022-01-11
EPI_ISL_9304494	RHD XV	RHD XV	2022-01-11
EPI_ISL_9304495	RHD XV	RHD XV	2022-01-11
EPI_ISL_9304496	RHD XV	RHD XV	2022-01-11
EPI_ISL_9304498	RHD XV	RHD XV	2022-01-12
EPI_ISL_9304499	RHD XV	RHD XV	2022-01-11
EPI_ISL_9304500	RHD XV	RHD XV	2022-01-11
EPI_ISL_9304501	RHD XV	RHD XV	2022-01-11
EPI_ISL_9304502	RHD XV	RHD XV	2022-01-11
EPI_ISL_9476185	RHD XV	RHD XV	2022-01-19
EPI_ISL_9476186	RHD XV	RHD XV	2022-01-18
EPI_ISL_9476187	RHD XV	RHD XV	2022-01-18
EPI_ISL_9476188	RHD XV	RHD XV	2022-01-18
EPI_ISL_9476189	RHD XV	RHD XV	2022-01-18
EPI_ISL_9476190	RHD XV	RHD XV	2022-01-19
EPI_ISL_9476191	SJdRP	RHD XV	2022-01-20
EPI_ISL_9476192	RHD XV	RHD XV	2022-01-20
EPI_ISL_9476193	RHD XV	RHD XV	2022-01-20
EPI_ISL_9476194	RHD XV	RHD XV	2022-01-20
EPI_ISL_9476195	RHD XV	RHD XV	2022-01-20
EPI_ISL_9476196	SJdRP	RHD XV	2022-01-20
EPI_ISL_9476439	SJdRP	SJdRP	2022-01-20
EPI_ISL_9476199	RHD XV	SJdRP	2022-01-12
EPI_ISL_9476200	SJdRP	SJdRP	2022-01-12
EPI_ISL_9476201	SJdRP	SJdRP	2022-01-12
EPI_ISL_9476202	SJdRP	SJdRP	2022-01-12
EPI_ISL_9476205	SJdRP	SJdRP	2022-01-11
EPI_ISL_9476206	SJdRP	SJdRP	2022-01-09
EPI_ISL_9476208	SJdRP	SJdRP	2022-01-11
EPI_ISL_9476209	SJdRP	SJdRP	2022-01-11
EPI_ISL_9476211	SJdRP	SJdRP	2022-01-12
EPI_ISL_9476212	SJdRP	SJdRP	2022-01-11
EPI_ISL_9476213	SJdRP	SJdRP	2022-01-12
EPI_ISL_9476214	SJdRP	SJdRP	2022-01-12
EPI_ISL_9476215	SJdRP	SJdRP	2022-01-12
EPI_ISL_9476216	SJdRP	SJdRP	2022-01-12
EPI_ISL_9476217	RHD XV	RHD XV	2022-01-19
EPI_ISL_9476218	RHD XV	RHD XV	2022-01-19
EPI_ISL_9476219	RHD XV	RHD XV	2022-01-19
EPI_ISL_9476220	RHD XV	RHD XV	2022-01-19
EPI_ISL_9476221	RHD XV	RHD XV	2022-01-19
EPI_ISL_9476223	RHD XV	RHD XV	2022-01-19
EPI_ISL_9476224	RHD XV	RHD XV	2022-01-19
EPI_ISL_9476226	RHD XV	RHD XV	2022-01-19
EPI_ISL_9476227	RHD XV	RHD XV	2022-01-19
EPI_ISL_9476229	RHD XV	RHD XV	2022-01-18
EPI_ISL_9476230	RHD XV	RHD XV	2022-01-18
EPI_ISL_9476231	SJdRP	RHD XV	2022-01-18
EPI_ISL_9476232	RHD XV	RHD XV	2022-01-11
EPI_ISL_9476239	RHD XV	RHD XV	2022-01-11
EPI_ISL_9476240	RHD XV	RHD XV	2022-01-18

EPI_ISL_9476242	RHD XV	RHD XV	2022-01-20
EPI_ISL_9476243	RHD XV	RHD XV	2022-01-20
EPI_ISL_9476244	SJdRP	RHD XV	2022-01-20
EPI_ISL_9476247	SJdRP	SJdRP	2022-01-12
EPI_ISL_9476250	SJdRP	SJdRP	2022-01-11
EPI_ISL_9476251	RHD XV	SJdRP	2022-01-10
EPI_ISL_9476254	RHD XV	SJdRP	2022-01-10
EPI_ISL_9476255	SJdRP	SJdRP	2022-01-10
EPI_ISL_9476256	RHD XV	SJdRP	2022-01-10
EPI_ISL_9476489	RHD XV	RHD XV	2022-01-19
EPI_ISL_9476260	RHD XV	RHD XV	2022-01-19
EPI_ISL_9476263	RHD XV	RHD XV	2022-01-19
EPI_ISL_9476264	RHD XV	RHD XV	2022-01-19
EPI_ISL_9805204	RHD XV	RHD XV	2022-01-27
EPI_ISL_9805383	SJdRP	RHD XV	2022-01-28
EPI_ISL_9805156	RHD XV	RHD XV	2022-01-28
EPI_ISL_9805251	RHD XV	RHD XV	2022-01-28
EPI_ISL_9805320	RHD XV	RHD XV	2022-01-28
EPI_ISL_9805381	SJdRP	RHD XV	2022-01-27
EPI_ISL_9805352	SJdRP	RHD XV	2022-01-28
EPI_ISL_9805388	SJdRP	RHD XV	2022-01-28
EPI_ISL_9805336	RHD XV	RHD XV	2022-01-27
EPI_ISL_9805294	RHD XV	RHD XV	2022-01-27
EPI_ISL_9805308	SJdRP	RHD XV	2022-01-27
EPI_ISL_9805219	RHD XV	RHD XV	2022-01-27
EPI_ISL_9805210	RHD XV	RHD XV	2022-01-28
EPI_ISL_9805412	RHD XV	RHD XV	2022-01-27
EPI_ISL_9805196	Southeast	RHD XV	2022-01-27
EPI_ISL_9805398	RHD XV	RHD XV	2022-01-27
EPI_ISL_9805194	SJdRP	RHD XV	2022-01-27
EPI_ISL_9805193	RHD XV	RHD XV	2022-01-27
EPI_ISL_9805382	RHD XV	RHD XV	2022-01-27
EPI_ISL_9805222	Midwest	RHD XV	2022-01-27
EPI_ISL_9805386	RHD XV	RHD XV	2022-01-28
EPI_ISL_9805343	RHD XV	RHD XV	2022-01-28
EPI_ISL_9805208	RHD XV	RHD XV	2022-01-28
EPI_ISL_9805340	RHD XV	RHD XV	2022-01-27
EPI_ISL_9805221	RHD XV	RHD XV	2022-01-28
EPI_ISL_9805212	RHD XV	RHD XV	2022-01-28
EPI_ISL_9805330	RHD XV	RHD XV	2022-01-28
EPI_ISL_9805191	RHD XV	RHD XV	2022-01-28
EPI_ISL_9805377	RHD XV	RHD XV	2022-01-28
EPI_ISL_9805232	SJdRP	RHD XV	2022-01-28
EPI_ISL_9805199	RHD XV	RHD XV	2022-01-28
EPI_ISL_9805391	RHD XV	RHD XV	2022-01-27
EPI_ISL_9805189	RHD XV	RHD XV	2022-01-27
EPI_ISL_9805300	RHD XV	RHD XV	2022-01-27
EPI_ISL_9805311	RHD XV	RHD XV	2022-01-27
EPI_ISL_9805147	RHD XV	RHD XV	2022-01-27
EPI_ISL_9805372	RHD XV	RHD XV	2022-01-27
EPI_ISL_9805285	RHD XV	RHD XV	2022-01-27
EPI_ISL_10309367	SJdRP	RHD XV	2022-02-06
EPI_ISL_10309015	RHD XV	RHD XV	2022-02-06
EPI_ISL_10309477	RHD XV	RHD XV	2022-02-04
EPI_ISL_10309026	RHD XV	RHD XV	2022-02-04
EPI_ISL_10309279	RHD XV	RHD XV	2022-02-04
EPI_ISL_10309420	RHD XV	RHD XV	2022-02-04
EPI_ISL_10309060	RHD XV	RHD XV	2022-02-04
EPI_ISL_10309111	RHD XV	RHD XV	2022-02-02
EPI_ISL_10309012	RHD XV	RHD XV	2022-02-03
EPI_ISL_10309159	RHD XV	RHD XV	2022-02-04
EPI_ISL_10309353	RHD XV	RHD XV	2022-02-04
EPI_ISL_10309521	SJdRP	RHD XV	2022-02-03

EPI_ISL_10309373	RHD XV	RHD XV	2022-02-03
EPI_ISL_10309537	SJdRP	RHD XV	2022-02-04
EPI_ISL_10309013	RHD XV	RHD XV	2022-02-04
EPI_ISL_10309509	RHD XV	RHD XV	2022-02-04
EPI_ISL_10309058	RHD XV	RHD XV	2022-02-04
EPI_ISL_10309456	RHD XV	RHD XV	2022-02-04
EPI_ISL_10309385	RHD XV	RHD XV	2022-02-05
EPI_ISL_10309419	SJdRP	RHD XV	2022-02-04
EPI_ISL_10309063	RHD XV	RHD XV	2022-02-04
EPI_ISL_10309494	SJdRP	RHD XV	2022-02-04
EPI_ISL_10309215	RHD XV	RHD XV	2022-02-04
EPI_ISL_10309047	RHD XV	RHD XV	2022-02-06
EPI_ISL_10309422	SJdRP	SJdRP	2022-02-07
EPI_ISL_10309384	RHD XV	RHD XV	2022-02-06
EPI_ISL_10309220	SJdRP	SJdRP	2022-02-04
EPI_ISL_10309016	RHD XV	RHD XV	2022-02-03
EPI_ISL_10309257	RHD XV	RHD XV	2022-02-04
EPI_ISL_10309281	RHD XV	RHD XV	2022-02-03
EPI_ISL_10309250	Northeast	RHD XV	2022-02-03
EPI_ISL_10309065	RHD XV	RHD XV	2022-02-03
EPI_ISL_10309029	RHD XV	RHD XV	2022-02-03
EPI_ISL_10309072	SJdRP	RHD XV	2022-02-03
EPI_ISL_10309444	RHD XV	RHD XV	2022-02-03
EPI_ISL_10309468	RHD XV	RHD XV	2022-02-02
EPI_ISL_10309100	RHD XV	RHD XV	2022-02-02
EPI_ISL_10309069	RHD XV	RHD XV	2022-02-02
EPI_ISL_10309386	RHD XV	RHD XV	2022-02-03
EPI_ISL_10309092	RHD XV	RHD XV	2022-02-03
EPI_ISL_10309541	SJdRP	SJdRP	2022-02-01
EPI_ISL_10309041	RHD XV	SJdRP	2022-02-02
EPI_ISL_10309899	RHD XV	RHD XV	2022-02-05
EPI_ISL_10309269	SJdRP	RHD XV	2022-02-04
EPI_ISL_10309023	RHD XV	RHD XV	2022-02-03
EPI_ISL_10309698	RHD XV	RHD XV	2022-02-05
EPI_ISL_10309140	RHD XV	RHD XV	2022-02-04
EPI_ISL_10309200	RHD XV	RHD XV	2022-02-05
EPI_ISL_10309255	RHD XV	RHD XV	2022-02-04
EPI_ISL_10309900	RHD XV	RHD XV	2022-02-05
EPI_ISL_10309901	RHD XV	RHD XV	2022-02-06
EPI_ISL_10309289	RHD XV	RHD XV	2022-02-05
EPI_ISL_10309180	RHD XV	RHD XV	2022-02-05
EPI_ISL_10309102	RHD XV	RHD XV	2022-02-07
EPI_ISL_10309103	SJdRP	RHD XV	2022-02-04
EPI_ISL_10309062	RHD XV	RHD XV	2022-02-03
EPI_ISL_10309043	RHD XV	RHD XV	2022-02-03
EPI_ISL_10309296	RHD XV	RHD XV	2022-02-02
EPI_ISL_10309240	RHD XV	RHD XV	2022-02-02
EPI_ISL_10309484	SJdRP	RHD XV	2022-02-03
EPI_ISL_10309309	RHD XV	RHD XV	2022-02-05
EPI_ISL_10309699	RHD XV	RHD XV	2022-02-05
EPI_ISL_11002774	RHD XV	RHD XV	2022-02-19
EPI_ISL_11002762	RHD XV	RHD XV	2022-02-18
EPI_ISL_11002746	SJdRP	RHD XV	2022-02-19
EPI_ISL_11002430	RHD XV	RHD XV	2022-02-17
EPI_ISL_11002770	RHD XV	RHD XV	2022-02-18
EPI_ISL_11002724	SJdRP	RHD XV	2022-02-16
EPI_ISL_11002758	RHD XV	RHD XV	2022-02-17
EPI_ISL_11002704	RHD XV	RHD XV	2022-02-17
EPI_ISL_11002699	SJdRP	RHD XV	2022-02-16
EPI_ISL_11002683	RHD XV	RHD XV	2022-02-17
EPI_ISL_11002700	RHD XV	RHD XV	2022-02-17
EPI_ISL_11002710	RHD XV	RHD XV	2022-02-17
EPI_ISL_11002775	SJdRP	RHD XV	2022-02-17

EPI_ISL_11002695	RHD XV	RHD XV	2022-02-17
EPI_ISL_11002763	RHD XV	RHD XV	2022-02-17
EPI_ISL_11002714	RHD XV	RHD XV	2022-02-18
EPI_ISL_11002693	RHD XV	RHD XV	2022-02-18
EPI_ISL_11002711	RHD XV	RHD XV	2022-02-17
EPI_ISL_11002696	RHD XV	RHD XV	2022-02-19
EPI_ISL_11002395	RHD XV	RHD XV	2022-02-17
EPI_ISL_11002757	RHD XV	RHD XV	2022-02-17
EPI_ISL_11002698	RHD XV	RHD XV	2022-02-19
EPI_ISL_11002694	RHD XV	RHD XV	2022-02-17
EPI_ISL_11002687	RHD XV	RHD XV	2022-02-19
EPI_ISL_11002709	RHD XV	RHD XV	2022-02-18
EPI_ISL_11002706	RHD XV	RHD XV	2022-02-18
EPI_ISL_11002713	RHD XV	RHD XV	2022-02-18
EPI_ISL_11002690	RHD XV	RHD XV	2022-02-18
EPI_ISL_11002721	RHD XV	RHD XV	2022-02-18
EPI_ISL_11002705	RHD XV	RHD XV	2022-02-18
EPI_ISL_11002717	RHD XV	RHD XV	2022-02-17
EPI_ISL_11002697	RHD XV	RHD XV	2022-02-17
EPI_ISL_11002732	RHD XV	RHD XV	2022-02-17
EPI_ISL_11002765	RHD XV	RHD XV	2022-02-16
EPI_ISL_11002715	RHD XV	RHD XV	2022-02-18
EPI_ISL_11002689	RHD XV	RHD XV	2022-02-16
EPI_ISL_11002691	RHD XV	RHD XV	2022-02-18
EPI_ISL_11002728	RHD XV	RHD XV	2022-02-17
EPI_ISL_11002688	RHD XV	RHD XV	2022-02-18
EPI_ISL_11002684	RHD XV	RHD XV	2022-02-18
EPI_ISL_11002707	RHD XV	RHD XV	2022-02-16
EPI_ISL_11002733	RHD XV	RHD XV	2022-02-18
EPI_ISL_11002407	RHD XV	RHD XV	2022-02-18
EPI_ISL_11002767	RHD XV	RHD XV	2022-02-18
EPI_ISL_11002737	RHD XV	RHD XV	2022-02-17
EPI_ISL_11002771	RHD XV	RHD XV	2022-02-19
EPI_ISL_11002702	RHD XV	RHD XV	2022-02-17
EPI_ISL_11002692	RHD XV	RHD XV	2022-02-17
EPI_ISL_11002722	RHD XV	RHD XV	2022-02-17
EPI_ISL_11002759	RHD XV	RHD XV	2022-02-19
EPI_ISL_11002768	RHD XV	RHD XV	2022-02-18
EPI_ISL_11002725	RHD XV	RHD XV	2022-02-18
EPI_ISL_11002753	RHD XV	RHD XV	2022-02-18
EPI_ISL_11027774	RHD XV	RHD XV	2022-02-25
EPI_ISL_11027246	RHD XV	RHD XV	2022-02-24
EPI_ISL_11027631	RHD XV	RHD XV	2022-02-24
EPI_ISL_11027648	RHD XV	RHD XV	2022-02-21
EPI_ISL_11027796	SJdRP	RHD XV	2022-02-25
EPI_ISL_11027297	RHD XV	RHD XV	2022-02-21
EPI_ISL_11027298	RHD XV	RHD XV	2022-02-21
EPI_ISL_11027884	RHD XV	RHD XV	2022-02-21
EPI_ISL_11027647	RHD XV	RHD XV	2022-02-25
EPI_ISL_11027299	RHD XV	RHD XV	2022-02-21
EPI_ISL_11027300	SJdRP	RHD XV	2022-02-21
EPI_ISL_11027301	RHD XV	RHD XV	2022-02-20
EPI_ISL_11027781	RHD XV	RHD XV	2022-02-20
EPI_ISL_11027494	RHD XV	RHD XV	2022-02-22
EPI_ISL_11027889	RHD XV	RHD XV	2022-02-22
EPI_ISL_11027940	SJdRP	RHD XV	2022-02-24
EPI_ISL_11027472	SJdRP	SJdRP	2022-02-24
EPI_ISL_11027826	SJdRP	SJdRP	2022-02-24
EPI_ISL_11027475	SJdRP	RHD XV	2022-02-24
EPI_ISL_11027471	RHD XV	RHD XV	2022-02-24
EPI_ISL_11027785	RHD XV	RHD XV	2022-02-24
EPI_ISL_11027779	SJdRP	SJdRP	2022-02-24
EPI_ISL_11027482	SJdRP	SJdRP	2022-02-24

EPI_ISL_11027773	RHD XV	RHD XV	2022-02-24
EPI_ISL_11027470	SJdRP	SJdRP	2022-02-24
EPI_ISL_11027777	SJdRP	RHD XV	2022-02-24
EPI_ISL_11027783	SJdRP	SJdRP	2022-02-24
EPI_ISL_11027621	SJdRP	SJdRP	2022-02-24
EPI_ISL_11027798	SJdRP	SJdRP	2022-02-24
EPI_ISL_11027473	Southeast	SJdRP	2022-02-24
EPI_ISL_11027833	RHD XV	RHD XV	2022-02-20
EPI_ISL_11027267	SJdRP	RHD XV	2022-02-20
EPI_ISL_11027268	RHD XV	RHD XV	2022-02-20
EPI_ISL_11027641	SJdRP	RHD XV	2022-02-21
EPI_ISL_11027302	RHD XV	RHD XV	2022-02-26
EPI_ISL_11027842	RHD XV	RHD XV	2022-02-21
EPI_ISL_11027303	RHD XV	RHD XV	2022-02-21
EPI_ISL_11027304	RHD XV	RHD XV	2022-02-21
EPI_ISL_11027775	RHD XV	RHD XV	2022-02-21
EPI_ISL_11027772	RHD XV	RHD XV	2022-02-25
EPI_ISL_11027442	RHD XV	RHD XV	2022-02-25
EPI_ISL_11027768	RHD XV	RHD XV	2022-02-20
EPI_ISL_11027305	RHD XV	RHD XV	2022-02-25
EPI_ISL_11027306	RHD XV	RHD XV	2022-02-20
EPI_ISL_11027439	RHD XV	RHD XV	2022-02-22
EPI_ISL_11027307	RHD XV	RHD XV	2022-02-20
EPI_ISL_11027428	RHD XV	RHD XV	2022-02-22
EPI_ISL_11027871	RHD XV	RHD XV	2022-02-20
EPI_ISL_11027479	RHD XV	SJdRP	2022-02-24
EPI_ISL_11027666	SJdRP	SJdRP	2022-02-24
EPI_ISL_11027845	RHD XV	SJdRP	2022-02-24
EPI_ISL_11027801	RHD XV	SJdRP	2022-02-24
EPI_ISL_11027766	RHD XV	RHD XV	2022-02-24
EPI_ISL_11027477	SJdRP	RHD XV	2022-02-24
EPI_ISL_11027784	RHD XV	SJdRP	2022-02-24
EPI_ISL_11027474	RHD XV	SJdRP	2022-02-24
EPI_ISL_11027847	SJdRP	SJdRP	2022-02-24
EPI_ISL_11027778	RHD XV	RHD XV	2022-02-24
EPI_ISL_11027905	SJdRP	SJdRP	2022-02-24
EPI_ISL_11027453	RHD XV	SJdRP	2022-02-24
EPI_ISL_11027478	RHD XV	RHD XV	2022-02-24
EPI_ISL_11027782	SJdRP	RHD XV	2022-02-21
EPI_ISL_11027690	RHD XV	RHD XV	2022-02-21
EPI_ISL_11027851	SJdRP	SJdRP	2022-02-23
EPI_ISL_11027843	RHD XV	SJdRP	2022-02-21
EPI_ISL_11027269	RHD XV	RHD XV	2022-02-20
EPI_ISL_11027821	RHD XV	RHD XV	2022-02-20
EPI_ISL_11027839	RHD XV	RHD XV	2022-02-20
EPI_ISL_11027469	RHD XV	RHD XV	2022-02-21
EPI_ISL_11027642	RHD XV	RHD XV	2022-02-20
EPI_ISL_11027308	RHD XV	RHD XV	2022-02-20
EPI_ISL_11115431	RHD XV	RHD XV	2022-03-04
EPI_ISL_11115432	SJdRP	SJdRP	2022-03-02
EPI_ISL_11115435	SJdRP	SJdRP	2022-03-04
EPI_ISL_11115436	SJdRP	RHD XV	2022-03-04
EPI_ISL_11115437	RHD XV	RHD XV	2022-03-02
EPI_ISL_11115438	SJdRP	SJdRP	2022-03-02
EPI_ISL_11115440	SJdRP	RHD XV	2022-03-02
EPI_ISL_11115441	RHD XV	SJdRP	2022-03-05
EPI_ISL_11115442	SJdRP	SJdRP	2022-03-02
EPI_ISL_11115444	RHD XV	SJdRP	2022-03-04
EPI_ISL_11115445	RHD XV	SJdRP	2022-03-04
EPI_ISL_11115446	SJdRP	SJdRP	2022-03-04
EPI_ISL_11115447	SJdRP	SJdRP	2022-03-04
EPI_ISL_11115448	RHD XV	RHD XV	2022-03-04
EPI_ISL_11115449	RHD XV	RHD XV	2022-03-03

EPI_ISL_11115450	SJdRP	SJdRP	2022-03-03
EPI_ISL_11115451	RHD XV	RHD XV	2022-03-03
EPI_ISL_11115452	RHD XV	RHD XV	2022-03-01
EPI_ISL_11115453	RHD XV	RHD XV	2022-02-28
EPI_ISL_11115454	RHD XV	RHD XV	2022-02-28
EPI_ISL_11115455	RHD XV	RHD XV	2022-02-27
EPI_ISL_11115456	RHD XV	RHD XV	2022-03-02
EPI_ISL_11115457	SJdRP	SJdRP	2022-03-05
EPI_ISL_11115458	RHD XV	RHD XV	2022-03-04
EPI_ISL_11115459	RHD XV	RHD XV	2022-03-01
EPI_ISL_11115460	SJdRP	RHD XV	2022-02-27
EPI_ISL_11115461	SJdRP	SJdRP	2022-03-02
EPI_ISL_11115462	RHD XV	SJdRP	2022-03-02
EPI_ISL_11115463	RHD XV	SJdRP	2022-03-02
EPI_ISL_11115464	RHD XV	RHD XV	2022-03-02
EPI_ISL_11115465	SJdRP	SJdRP	2022-03-02
EPI_ISL_11115466	SJdRP	SJdRP	2022-03-05
EPI_ISL_11115468	RHD XV	SJdRP	2022-03-04
EPI_ISL_11115469	RHD XV	RHD XV	2022-03-04
EPI_ISL_11115470	RHD XV	RHD XV	2022-02-28
EPI_ISL_11115471	RHD XV	RHD XV	2022-03-01
EPI_ISL_11115472	SJdRP	SJdRP	2022-03-02
EPI_ISL_11115473	RHD XV	RHD XV	2022-02-28
EPI_ISL_11115474	RHD XV	RHD XV	2022-02-27
EPI_ISL_11115475	SJdRP	SJdRP	2022-03-02
EPI_ISL_11115476	RHD XV	RHD XV	2022-02-27
EPI_ISL_11115477	RHD XV	RHD XV	2022-02-27
EPI_ISL_11115478	SJdRP	SJdRP	2022-03-02
EPI_ISL_11412076	RHD XV	SJdRP	2022-03-07
EPI_ISL_11412077	RHD XV	RHD XV	2022-03-06
EPI_ISL_11412078	RHD XV	RHD XV	2022-03-07
EPI_ISL_11412079	SJdRP	RHD XV	2022-03-12
EPI_ISL_11412080	SJdRP	RHD XV	2022-03-12
EPI_ISL_11412081	RHD XV	RHD XV	2022-03-12
EPI_ISL_11412082	RHD XV	RHD XV	2022-03-12
EPI_ISL_11412083	RHD XV	RHD XV	2022-03-12
EPI_ISL_11412084	RHD XV	RHD XV	2022-03-12
EPI_ISL_11412085	SJdRP	SJdRP	2022-03-12
EPI_ISL_11412086	RHD XV	SJdRP	2022-03-12
EPI_ISL_11412087	Northeast	RHD XV	2022-03-10
EPI_ISL_11412088	RHD XV	RHD XV	2022-03-09
EPI_ISL_11412089	RHD XV	RHD XV	2022-03-07
EPI_ISL_11412090	SJdRP	SJdRP	2022-03-11
EPI_ISL_11412091	SJdRP	SJdRP	2022-03-11
EPI_ISL_11412092	SJdRP	RHD XV	2022-03-07
EPI_ISL_11412093	SJdRP	SJdRP	2022-03-09
EPI_ISL_11412094	SJdRP	SJdRP	2022-03-09
EPI_ISL_11412095	SJdRP	SJdRP	2022-03-09
EPI_ISL_11412096	RHD XV	RHD XV	2022-03-07
EPI_ISL_11412097	SJdRP	RHD XV	2022-03-07
EPI_ISL_11412098	RHD XV	RHD XV	2022-03-07
EPI_ISL_11412099	SJdRP	SJdRP	2022-03-09
EPI_ISL_11412100	SJdRP	SJdRP	2022-03-09
EPI_ISL_11412101	RHD XV	RHD XV	2022-03-09
EPI_ISL_11412102	SJdRP	SJdRP	2022-03-09
EPI_ISL_11412103	SJdRP	SJdRP	2022-03-09
EPI_ISL_11412104	SJdRP	SJdRP	2022-03-09
EPI_ISL_11412105	SJdRP	SJdRP	2022-03-09
EPI_ISL_11412107	RHD XV	RHD XV	2022-03-08
EPI_ISL_11412108	RHD XV	RHD XV	2022-03-08
EPI_ISL_11412109	SJdRP	SJdRP	2022-03-08
EPI_ISL_11412110	Northeast	SJdRP	2022-03-07
EPI_ISL_11412111	RHD XV	RHD XV	2022-03-06

EPI_ISL_11412112	RHD XV	RHD XV	2022-03-07
EPI_ISL_11412113	SJdRP	RHD XV	2022-03-12
EPI_ISL_11412114	RHD XV	RHD XV	2022-03-07
EPI_ISL_11412115	SJdRP	SJdRP	2022-03-12
EPI_ISL_11412116	RHD XV	RHD XV	2022-03-12
EPI_ISL_11412117	RHD XV	RHD XV	2022-03-12
EPI_ISL_11412118	SJdRP	SJdRP	2022-03-12
EPI_ISL_11412119	SJdRP	SJdRP	2022-03-12
EPI_ISL_11412120	RHD XV	RHD XV	2022-03-07
EPI_ISL_11412121	SJdRP	SJdRP	2022-03-12
EPI_ISL_11412122	SJdRP	SJdRP	2022-03-12
EPI_ISL_11412123	RHD XV	RHD XV	2022-03-09
EPI_ISL_11412124	RHD XV	RHD XV	2022-03-09
EPI_ISL_11412125	RHD XV	RHD XV	2022-03-09
EPI_ISL_11412126	RHD XV	RHD XV	2022-03-09
EPI_ISL_11412127	SJdRP	SJdRP	2022-03-11
EPI_ISL_11412128	RHD XV	SJdRP	2022-03-11
EPI_ISL_11412129	RHD XV	RHD XV	2022-03-11
EPI_ISL_11412130	SJdRP	SJdRP	2022-03-09
EPI_ISL_11412131	RHD XV	RHD XV	2022-03-09
EPI_ISL_11412132	RHD XV	RHD XV	2022-03-07
EPI_ISL_11412133	RHD XV	RHD XV	2022-03-07
EPI_ISL_11412134	RHD XV	RHD XV	2022-03-07
EPI_ISL_11412135	SJdRP	SJdRP	2022-03-09
EPI_ISL_11412136	RHD XV	SJdRP	2022-03-09
EPI_ISL_11412137	RHD XV	SJdRP	2022-03-09
EPI_ISL_11412138	SJdRP	SJdRP	2022-03-07
EPI_ISL_11412139	RHD XV	RHD XV	2022-03-08
EPI_ISL_11412140	RHD XV	RHD XV	2022-03-08
EPI_ISL_11412141	RHD XV	RHD XV	2022-03-07
EPI_ISL_11412142	RHD XV	SJdRP	2022-03-08
EPI_ISL_11412143	SJdRP	RHD XV	2022-03-08
EPI_ISL_11412144	RHD XV	RHD XV	2022-03-08
EPI_ISL_11412145	RHD XV	SJdRP	2022-03-08
EPI_ISL_11763884	SJdRP	SJdRP	2022-03-19
EPI_ISL_11763885	RHD XV	RHD XV	2022-03-19
EPI_ISL_11763886	RHD XV	RHD XV	2022-03-19
EPI_ISL_11763887	SJdRP	SJdRP	2022-03-19
EPI_ISL_11763888	RHD XV	RHD XV	2022-03-17
EPI_ISL_11763889	RHD XV	SJdRP	2022-03-17
EPI_ISL_11763891	RHD XV	RHD XV	2022-03-16
EPI_ISL_11763892	SJdRP	SJdRP	2022-03-15
EPI_ISL_11763893	RHD XV	RHD XV	2022-03-15
EPI_ISL_11763894	RHD XV	RHD XV	2022-03-15
EPI_ISL_11763895	RHD XV	SJdRP	2022-03-15
EPI_ISL_11763896	RHD XV	RHD XV	2022-03-15
EPI_ISL_11763897	SJdRP	SJdRP	2022-03-15
EPI_ISL_11763898	SJdRP	RHD XV	2022-03-14
EPI_ISL_11763899	SJdRP	SJdRP	2022-03-14
EPI_ISL_11763900	SJdRP	SJdRP	2022-03-14
EPI_ISL_11763901	RHD XV	RHD XV	2022-03-15
EPI_ISL_11763902	SJdRP	RHD XV	2022-03-17
EPI_ISL_11763903	RHD XV	SJdRP	2022-03-15
EPI_ISL_11763904	RHD XV	SJdRP	2022-03-14
EPI_ISL_11763905	SJdRP	SJdRP	2022-03-14
EPI_ISL_11763906	RHD XV	SJdRP	2022-03-14
EPI_ISL_11763907	RHD XV	RHD XV	2022-03-16
EPI_ISL_11763908	SJdRP	RHD XV	2022-03-19
EPI_ISL_11763909	SJdRP	SJdRP	2022-03-16
EPI_ISL_11814907	RHD XV	RHD XV	2022-03-21
EPI_ISL_11814927	SJdRP	RHD XV	2022-03-21
EPI_ISL_11814928	SJdRP	SJdRP	2022-03-21
EPI_ISL_11814929	SJdRP	SJdRP	2022-03-21

EPI_ISL_11814948	RHD XV	SJdRP	2022-03-21
EPI_ISL_11814949	RHD XV	RHD XV	2022-03-21
EPI_ISL_11814950	RHD XV	SJdRP	2022-03-20
EPI_ISL_11814977	SJdRP	RHD XV	2022-03-21
EPI_ISL_11814990	SJdRP	SJdRP	2022-03-23
EPI_ISL_11814993	RHD XV	RHD XV	2022-03-26
EPI_ISL_11814994	RHD XV	RHD XV	2022-03-26
EPI_ISL_11814995	RHD XV	RHD XV	2022-03-21
EPI_ISL_11814996	SJdRP	SJdRP	2022-03-21
EPI_ISL_11814997	SJdRP	SJdRP	2022-03-24
EPI_ISL_11814999	RHD XV	RHD XV	2022-03-24
EPI_ISL_11815000	SJdRP	SJdRP	2022-03-24
EPI_ISL_11815001	SJdRP	SJdRP	2022-03-24
EPI_ISL_11815002	RHD XV	SJdRP	2022-03-24
EPI_ISL_11815003	SJdRP	SJdRP	2022-03-24
EPI_ISL_11815004	RHD XV	SJdRP	2022-03-23
EPI_ISL_11815005	SJdRP	SJdRP	2022-03-22
EPI_ISL_11815020	SJdRP	SJdRP	2022-03-23
EPI_ISL_11815021	RHD XV	SJdRP	2022-03-23
EPI_ISL_2425443	Northeast	Southeast	2021-01-06
EPI_ISL_2344459	Southeast	Southeast	2021-01-21
EPI_ISL_7614788	SJdRP	2021-09-19	2021-09-21
EPI_ISL_7614791	SJdRP	2021-09-19	2021-09-21
EPI_ISL_7614801	RHD XV	2021-09-19	2021-09-23
EPI_ISL_7614802	RHD XV	2021-09-19	2021-09-23
EPI_ISL_7614795	SJdRP	2021-09-19	2021-09-23
EPI_ISL_7614803	SJdRP	2021-09-19	2021-09-23
EPI_ISL_7614800	SJdRP	2021-09-19	2021-09-30
EPI_ISL_7614796	SJdRP	2021-09-19	2021-09-30
EPI_ISL_2551533	Southeast	Southeast	2021-02-20
EPI_ISL_11940591	RHD XV	RHD XV	2021-06-28
EPI_ISL_11940592	RHD XV	RHD XV	2021-08-10
EPI_ISL_11940593	RHD XV	RHD XV	2021-08-08
EPI_ISL_11940594	RHD XV	RHD XV	2021-08-09
EPI_ISL_11940595	RHD XV	RHD XV	2021-08-09
EPI_ISL_11940596	RHD XV	RHD XV	2021-08-09
EPI_ISL_11940597	RHD XV	RHD XV	2021-08-09
EPI_ISL_11940598	RHD XV	RHD XV	2021-08-09
EPI_ISL_11940599	SJdRP	RHD XV	2021-08-09
EPI_ISL_11940600	RHD XV	RHD XV	2021-08-09
EPI_ISL_11940601	RHD XV	RHD XV	2021-08-09
EPI_ISL_11940602	RHD XV	RHD XV	2021-08-09
EPI_ISL_11940603	RHD XV	RHD XV	2021-08-09
EPI_ISL_11940604	RHD XV	RHD XV	2021-08-09
EPI_ISL_11940605	RHD XV	RHD XV	2021-08-09
EPI_ISL_11940606	RHD XV	RHD XV	2021-08-09
EPI_ISL_11940607	RHD XV	RHD XV	2021-08-09
EPI_ISL_11940608	RHD XV	RHD XV	2021-08-09
EPI_ISL_11940609	RHD XV	RHD XV	2021-08-10
EPI_ISL_11940610	RHD XV	RHD XV	2021-08-10
EPI_ISL_11940611	RHD XV	RHD XV	2021-08-10
EPI_ISL_11940612	RHD XV	RHD XV	2021-08-10
EPI_ISL_11940613	RHD XV	RHD XV	2021-08-10
EPI_ISL_11940614	RHD XV	RHD XV	2021-08-09
EPI_ISL_11940615	RHD XV	RHD XV	2021-08-09
EPI_ISL_11940616	RHD XV	RHD XV	2021-08-09
EPI_ISL_11940617	RHD XV	RHD XV	2021-08-09
EPI_ISL_11940619	RHD XV	RHD XV	2021-08-09
EPI_ISL_11940620	RHD XV	RHD XV	2021-08-09
EPI_ISL_11940621	RHD XV	RHD XV	2021-08-09
EPI_ISL_11940622	RHD XV	RHD XV	2021-08-09
EPI_ISL_11940623	RHD XV	RHD XV	2021-08-09
EPI_ISL_11940624	RHD XV	RHD XV	2021-08-09

EPI_ISL_11940625	RHD XV	RHD XV	2021-08-09
EPI_ISL_11940626	RHD XV	RHD XV	2021-08-09
EPI_ISL_11940627	RHD XV	RHD XV	2021-08-17
EPI_ISL_11940628	RHD XV	RHD XV	2021-08-16
EPI_ISL_11940629	RHD XV	RHD XV	2021-08-16
EPI_ISL_11940630	SJdRP	RHD XV	2021-08-16
EPI_ISL_11940631	RHD XV	RHD XV	2021-08-16
EPI_ISL_11940632	Southeast	RHD XV	2021-08-16
EPI_ISL_11940633	RHD XV	RHD XV	2021-08-16
EPI_ISL_11940634	RHD XV	RHD XV	2021-08-16
EPI_ISL_11940635	RHD XV	RHD XV	2021-08-16
EPI_ISL_11940636	RHD XV	RHD XV	2021-08-16
EPI_ISL_11940637	RHD XV	RHD XV	2021-08-16
EPI_ISL_11940638	RHD XV	RHD XV	2021-08-16
EPI_ISL_11940640	RHD XV	RHD XV	2021-08-16
EPI_ISL_11940641	RHD XV	RHD XV	2021-08-17
EPI_ISL_11940642	RHD XV	RHD XV	2021-08-17
EPI_ISL_11940643	RHD XV	RHD XV	2021-08-16
EPI_ISL_11940644	RHD XV	RHD XV	2021-08-16
EPI_ISL_11940645	RHD XV	RHD XV	2021-08-16
EPI_ISL_11940646	RHD XV	RHD XV	2021-08-17
EPI_ISL_11940647	RHD XV	RHD XV	2021-08-17
EPI_ISL_11940648	RHD XV	RHD XV	2021-08-17
EPI_ISL_11940649	RHD XV	RHD XV	2021-08-17
EPI_ISL_11940650	RHD XV	RHD XV	2021-08-16
EPI_ISL_11940651	RHD XV	RHD XV	2021-08-17
EPI_ISL_11940652	RHD XV	RHD XV	2021-08-16
EPI_ISL_11940653	RHD XV	RHD XV	2021-08-16
EPI_ISL_11940654	RHD XV	RHD XV	2021-08-16
EPI_ISL_11940655	RHD XV	RHD XV	2021-08-16
EPI_ISL_11940656	RHD XV	RHD XV	2021-08-16
EPI_ISL_11940657	Midwest	RHD XV	2021-08-16
EPI_ISL_11940658	RHD XV	RHD XV	2021-08-16
EPI_ISL_11940659	RHD XV	RHD XV	2021-08-16
EPI_ISL_11940660	RHD XV	RHD XV	2021-08-16
EPI_ISL_11940661	RHD XV	RHD XV	2021-08-16
EPI_ISL_11940662	RHD XV	RHD XV	2021-08-16
EPI_ISL_11940663	RHD XV	RHD XV	2021-08-16
EPI_ISL_11940664	RHD XV	RHD XV	2021-08-16
EPI_ISL_11940665	RHD XV	RHD XV	2021-08-16
EPI_ISL_11940666	RHD XV	RHD XV	2021-08-16
EPI_ISL_11940667	RHD XV	RHD XV	2021-08-16
EPI_ISL_11940668	RHD XV	RHD XV	2021-08-16
EPI_ISL_11940669	RHD XV	RHD XV	2021-08-16
EPI_ISL_11940670	RHD XV	RHD XV	2021-08-16
EPI_ISL_11940671	RHD XV	RHD XV	2021-08-16
EPI_ISL_11940672	RHD XV	RHD XV	2021-08-16
EPI_ISL_11940673	SJdRP	SJdRP	2021-08-19
EPI_ISL_11940674	SJdRP	SJdRP	2021-08-19
EPI_ISL_11940675	North	SJdRP	2021-08-18
EPI_ISL_11940676	SJdRP	SJdRP	2021-08-19
EPI_ISL_11940677	SJdRP	SJdRP	2021-08-19
EPI_ISL_11940678	SJdRP	SJdRP	2021-08-19
EPI_ISL_11940679	SJdRP	RHD XV	2021-08-19
EPI_ISL_11940680	SJdRP	SJdRP	2021-08-19
EPI_ISL_11940681	RHD XV	SJdRP	2021-08-19
EPI_ISL_11940682	SJdRP	SJdRP	2021-08-19
EPI_ISL_11940683	SJdRP	SJdRP	2021-08-19
EPI_ISL_11940684	Southeast	SJdRP	2021-08-19
EPI_ISL_11940685	RHD XV	SJdRP	2021-08-19
EPI_ISL_11940686	SJdRP	SJdRP	2021-08-19
EPI_ISL_11940687	SJdRP	SJdRP	2021-08-19
EPI_ISL_11940688	RHD XV	SJdRP	2021-08-19

EPI_ISL_11940689	RHD XV	SJdRP	2021-08-19
EPI_ISL_11940690	RHD XV	RHD XV	2021-08-19
EPI_ISL_11940691	SJdRP	RHD XV	2021-08-19
EPI_ISL_11940692	RHD XV	RHD XV	2021-08-19
EPI_ISL_11940693	SJdRP	SJdRP	2021-08-19
EPI_ISL_11940694	SJdRP	SJdRP	2021-08-18
EPI_ISL_11940695	SJdRP	SJdRP	2021-08-18
EPI_ISL_11940696	SJdRP	SJdRP	2021-08-18
EPI_ISL_11940697	SJdRP	SJdRP	2021-08-18
EPI_ISL_11940698	SJdRP	SJdRP	2021-08-18
EPI_ISL_11940699	SJdRP	SJdRP	2021-08-18
EPI_ISL_11940700	RHD XV	SJdRP	2021-08-18
EPI_ISL_11940701	RHD XV	RHD XV	2021-08-18
EPI_ISL_11940702	SJdRP	RHD XV	2021-08-18
EPI_ISL_11940703	RHD XV	RHD XV	2021-08-18
EPI_ISL_11940704	RHD XV	RHD XV	2021-08-18
EPI_ISL_11940705	RHD XV	RHD XV	2021-08-18
EPI_ISL_11940706	RHD XV	RHD XV	2021-08-18
EPI_ISL_11940707	RHD XV	RHD XV	2021-08-18
EPI_ISL_11940708	RHD XV	RHD XV	2021-08-18
EPI_ISL_11940709	SJdRP	RHD XV	2021-08-19
EPI_ISL_11940710	SJdRP	SJdRP	2021-08-18
EPI_ISL_11940711	SJdRP	SJdRP	2021-08-18
EPI_ISL_11940712	SJdRP	SJdRP	2021-08-18
EPI_ISL_11940713	SJdRP	SJdRP	2021-08-18
EPI_ISL_11940714	RHD XV	SJdRP	2021-08-18
EPI_ISL_11940715	RHD XV	RHD XV	2021-08-19
EPI_ISL_11940716	SJdRP	SJdRP	2021-08-19
EPI_ISL_11940717	RHD XV	SJdRP	2021-08-19
EPI_ISL_11940718	SJdRP	SJdRP	2021-08-19
EPI_ISL_11940719	SJdRP	SJdRP	2021-08-19
EPI_ISL_11940720	SJdRP	SJdRP	2021-08-19
EPI_ISL_11940721	SJdRP	SJdRP	2021-08-19
EPI_ISL_11940722	Midwest	SJdRP	2021-08-19
EPI_ISL_11940723	SJdRP	SJdRP	2021-08-19
EPI_ISL_11940724	SJdRP	SJdRP	2021-08-19
EPI_ISL_11940725	SJdRP	SJdRP	2021-08-19
EPI_ISL_11940726	SJdRP	SJdRP	2021-08-19
EPI_ISL_11940727	RHD XV	SJdRP	2021-08-18
EPI_ISL_11940728	SJdRP	SJdRP	2021-08-18
EPI_ISL_11940729	RHD XV	SJdRP	2021-08-18
EPI_ISL_11940730	RHD XV	RHD XV	2021-08-19
EPI_ISL_11940731	RHD XV	RHD XV	2021-08-18
EPI_ISL_11940732	RHD XV	RHD XV	2021-08-18
EPI_ISL_11940733	RHD XV	RHD XV	2021-08-18
EPI_ISL_11940734	RHD XV	RHD XV	2021-08-18
EPI_ISL_11940735	RHD XV	RHD XV	2021-08-18
EPI_ISL_11940736	RHD XV	RHD XV	2021-08-18
EPI_ISL_11940737	Northeast	SJdRP	2021-08-19
EPI_ISL_11940738	SJdRP	SJdRP	2021-08-19
EPI_ISL_11940739	SJdRP	SJdRP	2021-08-19
EPI_ISL_11940740	SJdRP	SJdRP	2021-08-19
EPI_ISL_11940741	SJdRP	SJdRP	2021-08-19
EPI_ISL_11940742	SJdRP	SJdRP	2021-08-19
EPI_ISL_11940743	Midwest	SJdRP	2021-08-19
EPI_ISL_11940744	SJdRP	SJdRP	2021-08-18
EPI_ISL_11940745	SJdRP	SJdRP	2021-08-19
EPI_ISL_11940746	SJdRP	RHD XV	2021-08-19
EPI_ISL_11940747	SJdRP	SJdRP	2021-08-19
EPI_ISL_11940748	RHD XV	RHD XV	2021-08-24
EPI_ISL_11940749	RHD XV	RHD XV	2021-08-24
EPI_ISL_11940750	RHD XV	RHD XV	2021-08-24
EPI_ISL_11940751	RHD XV	RHD XV	2021-08-24

EPI_ISL_11940752	RHD XV	RHD XV	2021-08-23
EPI_ISL_11940753	RHD XV	RHD XV	2021-08-23
EPI_ISL_11940755	SJdRP	RHD XV	2021-08-23
EPI_ISL_11940756	RHD XV	RHD XV	2021-08-23
EPI_ISL_11940757	RHD XV	RHD XV	2021-08-23
EPI_ISL_11940758	RHD XV	RHD XV	2021-08-23
EPI_ISL_11940759	RHD XV	RHD XV	2021-08-23
EPI_ISL_11940760	RHD XV	RHD XV	2021-08-23
EPI_ISL_11940761	RHD XV	RHD XV	2021-08-23
EPI_ISL_11940762	RHD XV	RHD XV	2021-08-23
EPI_ISL_11940763	Midwest	RHD XV	2021-08-23
EPI_ISL_11940764	RHD XV	RHD XV	2021-08-23
EPI_ISL_11940765	RHD XV	RHD XV	2021-08-23
EPI_ISL_11940766	RHD XV	RHD XV	2021-08-23
EPI_ISL_11940767	RHD XV	RHD XV	2021-08-23
EPI_ISL_11940768	RHD XV	RHD XV	2021-08-23
EPI_ISL_11940769	RHD XV	RHD XV	2021-08-23
EPI_ISL_11940770	RHD XV	RHD XV	2021-08-23
EPI_ISL_11940771	RHD XV	RHD XV	2021-08-23
EPI_ISL_11940772	RHD XV	RHD XV	2021-08-24
EPI_ISL_11940773	RHD XV	RHD XV	2021-08-25
EPI_ISL_11940774	RHD XV	RHD XV	2021-08-25
EPI_ISL_11940775	RHD XV	RHD XV	2021-08-23
EPI_ISL_11940776	RHD XV	RHD XV	2021-08-23
EPI_ISL_11940777	RHD XV	RHD XV	2021-08-25
EPI_ISL_11940778	RHD XV	RHD XV	2021-08-25
EPI_ISL_11940779	RHD XV	SJdRP	2021-08-25
EPI_ISL_11940780	Northeast	SJdRP	2021-08-25
EPI_ISL_11940781	SJdRP	SJdRP	2021-08-25
EPI_ISL_11940782	SJdRP	SJdRP	2021-08-25
EPI_ISL_11940783	SJdRP	SJdRP	2021-08-25
EPI_ISL_11940784	SJdRP	SJdRP	2021-08-25
EPI_ISL_11940785	RHD XV	SJdRP	2021-08-25
EPI_ISL_11940786	SJdRP	SJdRP	2021-08-25
EPI_ISL_11940787	SJdRP	SJdRP	2021-08-25
EPI_ISL_11940788	SJdRP	SJdRP	2021-08-25
EPI_ISL_11940789	RHD XV	SJdRP	2021-08-25
EPI_ISL_11940790	SJdRP	SJdRP	2021-08-23
EPI_ISL_11940791	RHD XV	RHD XV	2021-08-23
EPI_ISL_11940792	RHD XV	RHD XV	2021-08-23
EPI_ISL_11940793	RHD XV	RHD XV	2021-08-23
EPI_ISL_11940794	SJdRP	SJdRP	2021-08-23
EPI_ISL_11940795	RHD XV	SJdRP	2021-08-23
EPI_ISL_11940796	SJdRP	SJdRP	2021-08-23
EPI_ISL_11940797	SJdRP	SJdRP	2021-08-23
EPI_ISL_11940798	SJdRP	SJdRP	2021-08-23
EPI_ISL_11940799	RHD XV	SJdRP	2021-08-23
EPI_ISL_11940800	RHD XV	SJdRP	2021-08-23
EPI_ISL_11940801	SJdRP	SJdRP	2021-08-23
EPI_ISL_11940802	SJdRP	SJdRP	2021-08-23
EPI_ISL_11940803	RHD XV	RHD XV	2021-08-25
EPI_ISL_11940804	RHD XV	RHD XV	2021-08-25
EPI_ISL_11940805	RHD XV	RHD XV	2021-08-25
EPI_ISL_11940806	RHD XV	RHD XV	2021-08-25
EPI_ISL_11940807	RHD XV	RHD XV	2021-08-25
EPI_ISL_11940808	RHD XV	RHD XV	2021-08-25
EPI_ISL_11940809	RHD XV	RHD XV	2021-08-25
EPI_ISL_11940810	SJdRP	SJdRP	2021-08-25
EPI_ISL_11940811	SJdRP	SJdRP	2021-08-25
EPI_ISL_11940812	RHD XV	RHD XV	2021-08-23
EPI_ISL_11940813	RHD XV	RHD XV	2021-08-23
EPI_ISL_11940814	RHD XV	RHD XV	2021-08-23
EPI_ISL_11940815	RHD XV	RHD XV	2021-08-23

EPI_ISL_11940816	SJdRP	RHD XV	2021-08-23
EPI_ISL_11940817	SJdRP	SJdRP	2021-08-23
EPI_ISL_11940818	SJdRP	SJdRP	2021-08-23
EPI_ISL_11940819	SJdRP	SJdRP	2021-08-23
EPI_ISL_11940820	SJdRP	SJdRP	2021-09-01
EPI_ISL_11940821	RHD XV	SJdRP	2021-09-01
EPI_ISL_11940822	SJdRP	SJdRP	2021-09-01
EPI_ISL_11940823	SJdRP	SJdRP	2021-09-01
EPI_ISL_11940824	RHD XV	SJdRP	2021-09-01
EPI_ISL_11940825	SJdRP	SJdRP	2021-09-01
EPI_ISL_11940826	RHD XV	SJdRP	2021-09-01
EPI_ISL_11940827	RHD XV	RHD XV	2021-08-31
EPI_ISL_11940828	North	RHD XV	2021-08-31
EPI_ISL_11940829	RHD XV	RHD XV	2021-08-31
EPI_ISL_11940830	SJdRP	RHD XV	2021-08-30
EPI_ISL_11940831	RHD XV	RHD XV	2021-08-30
EPI_ISL_11940832	RHD XV	RHD XV	2021-08-30
EPI_ISL_11940833	SJdRP	SJdRP	2021-08-31
EPI_ISL_11940834	RHD XV	SJdRP	2021-08-31
EPI_ISL_11940835	North	SJdRP	2021-08-31
EPI_ISL_11940836	Southeast	SJdRP	2021-08-31
EPI_ISL_11940837	SJdRP	RHD XV	2021-09-01
EPI_ISL_11940838	RHD XV	RHD XV	2021-08-31
EPI_ISL_11940839	RHD XV	RHD XV	2021-08-31
EPI_ISL_11940840	RHD XV	RHD XV	2021-08-31
EPI_ISL_11940841	RHD XV	RHD XV	2021-09-01
EPI_ISL_11940842	RHD XV	RHD XV	2021-09-01
EPI_ISL_11940843	RHD XV	RHD XV	2021-09-01
EPI_ISL_11940844	RHD XV	RHD XV	2021-08-31
EPI_ISL_11940845	RHD XV	RHD XV	2021-08-31
EPI_ISL_11940846	RHD XV	RHD XV	2021-08-31
EPI_ISL_11940847	RHD XV	RHD XV	2021-08-31
EPI_ISL_11940848	RHD XV	RHD XV	2021-08-31
EPI_ISL_11940849	RHD XV	RHD XV	2021-08-31
EPI_ISL_11940850	RHD XV	RHD XV	2021-08-30
EPI_ISL_11940851	RHD XV	RHD XV	2021-08-31
EPI_ISL_11940852	SJdRP	SJdRP	2021-09-07
EPI_ISL_11940853	SJdRP	RHD XV	2021-09-07
EPI_ISL_11940854	RHD XV	SJdRP	2021-09-08
EPI_ISL_11940855	RHD XV	RHD XV	2021-09-04
EPI_ISL_11940856	SJdRP	SJdRP	2021-09-08
EPI_ISL_11940857	SJdRP	SJdRP	2021-09-05
EPI_ISL_11940858	SJdRP	SJdRP	2021-09-04
EPI_ISL_11940859	SJdRP	RHD XV	2021-09-05
EPI_ISL_11940860	SJdRP	RHD XV	2021-09-07
EPI_ISL_11940861	RHD XV	RHD XV	2021-09-06
EPI_ISL_11940862	RHD XV	RHD XV	2021-09-06
EPI_ISL_11940863	RHD XV	RHD XV	2021-09-06
EPI_ISL_5800168	RHD XV	RHD XV	2021-04-07
EPI_ISL_5800176	RHD XV	RHD XV	2021-04-07
EPI_ISL_5800177	RHD XV	RHD XV	2021-04-07
EPI_ISL_5800178	RHD XV	RHD XV	2021-04-07
EPI_ISL_5800180	Southeast	RHD XV	2021-04-07
EPI_ISL_5800185	RHD XV	RHD XV	2021-04-07
EPI_ISL_5800186	RHD XV	RHD XV	2021-04-07
EPI_ISL_5800187	RHD XV	RHD XV	2021-04-07
EPI_ISL_5800188	RHD XV	RHD XV	2021-04-07
EPI_ISL_5800189	RHD XV	RHD XV	2021-04-07
EPI_ISL_5800190	RHD XV	RHD XV	2021-04-07
EPI_ISL_5800191	RHD XV	RHD XV	2021-04-07
EPI_ISL_5800193	RHD XV	RHD XV	2021-04-07
EPI_ISL_5800195	RHD XV	RHD XV	2021-04-07
EPI_ISL_5800196	RHD XV	RHD XV	2021-04-07

EPI_ISL_5800198	RHD XV	RHD XV	2021-04-07
EPI_ISL_5800199	RHD XV	RHD XV	2021-04-07
EPI_ISL_5800200	RHD XV	RHD XV	2021-04-08
EPI_ISL_5800451	RHD XV	RHD XV	2021-04-14
EPI_ISL_5800452	RHD XV	RHD XV	2021-04-14
EPI_ISL_5800453	RHD XV	RHD XV	2021-04-14
EPI_ISL_5800458	SJdRP	RHD XV	2021-04-14
EPI_ISL_5800459	RHD XV	RHD XV	2021-04-14
EPI_ISL_5800460	RHD XV	RHD XV	2021-04-14
EPI_ISL_5800466	RHD XV	RHD XV	2021-04-15
EPI_ISL_5800467	RHD XV	RHD XV	2021-04-14
EPI_ISL_5800471	RHD XV	RHD XV	2021-04-15
EPI_ISL_5800474	RHD XV	RHD XV	2021-04-15
EPI_ISL_5800475	RHD XV	RHD XV	2021-04-15
EPI_ISL_5800477	RHD XV	RHD XV	2021-04-14
EPI_ISL_5800478	RHD XV	RHD XV	2021-04-15
EPI_ISL_5800480	RHD XV	RHD XV	2021-04-15
EPI_ISL_5800481	Southeast	RHD XV	2021-04-15
EPI_ISL_5800482	RHD XV	RHD XV	2021-04-15
EPI_ISL_5800483	RHD XV	RHD XV	2021-04-15
EPI_ISL_5800484	RHD XV	RHD XV	2021-04-15
EPI_ISL_5800485	RHD XV	RHD XV	2021-04-14
EPI_ISL_5800486	RHD XV	RHD XV	2021-04-14
EPI_ISL_5800487	RHD XV	RHD XV	2021-04-15
EPI_ISL_5800489	RHD XV	RHD XV	2021-04-15
EPI_ISL_5800490	RHD XV	RHD XV	2021-04-15
EPI_ISL_5800491	RHD XV	RHD XV	2021-04-15
EPI_ISL_5800492	RHD XV	RHD XV	2021-04-15
EPI_ISL_5800493	RHD XV	RHD XV	2021-04-15
EPI_ISL_5800757	RHD XV	RHD XV	2021-04-20
EPI_ISL_5800759	Northeast	RHD XV	2021-04-20
EPI_ISL_5800760	RHD XV	RHD XV	2021-04-20
EPI_ISL_5800779	Midwest	RHD XV	2021-04-20
EPI_ISL_5800780	Southeast	RHD XV	2021-04-20
EPI_ISL_5800791	RHD XV	RHD XV	2021-04-20
EPI_ISL_5800792	RHD XV	RHD XV	2021-04-21
EPI_ISL_5800793	RHD XV	RHD XV	2021-04-21
EPI_ISL_5800794	RHD XV	RHD XV	2021-04-21
EPI_ISL_5800797	RHD XV	RHD XV	2021-04-20
EPI_ISL_5800801	RHD XV	RHD XV	2021-04-20
EPI_ISL_5800802	RHD XV	RHD XV	2021-04-21
EPI_ISL_5800803	RHD XV	RHD XV	2021-04-21
EPI_ISL_5800804	RHD XV	RHD XV	2021-04-21
EPI_ISL_5800805	RHD XV	RHD XV	2021-04-21
EPI_ISL_5800809	RHD XV	RHD XV	2021-04-21
EPI_ISL_5800810	South	RHD XV	2021-04-21
EPI_ISL_5800811	RHD XV	RHD XV	2021-04-21
EPI_ISL_5800812	RHD XV	RHD XV	2021-04-21
EPI_ISL_5800813	RHD XV	RHD XV	2021-04-20
EPI_ISL_5800814	RHD XV	RHD XV	2021-04-22
EPI_ISL_5800816	RHD XV	RHD XV	2021-04-22
EPI_ISL_5800818	RHD XV	RHD XV	2021-04-20
EPI_ISL_5800819	RHD XV	RHD XV	2021-04-22
EPI_ISL_5800821	RHD XV	RHD XV	2021-04-20
EPI_ISL_5800822	RHD XV	RHD XV	2021-04-20
EPI_ISL_5800823	RHD XV	RHD XV	2021-04-22
EPI_ISL_5800824	RHD XV	RHD XV	2021-04-22
EPI_ISL_5800825	RHD XV	RHD XV	2021-04-22
EPI_ISL_5800826	SJdRP	RHD XV	2021-04-20
EPI_ISL_5800827	RHD XV	RHD XV	2021-04-20
EPI_ISL_5800828	RHD XV	RHD XV	2021-04-21
EPI_ISL_5800829	Southeast	RHD XV	2021-04-20
EPI_ISL_5800830	RHD XV	RHD XV	2021-04-22

EPI_ISL_5800831	RHD XV	RHD XV	2021-04-20
EPI_ISL_5800833	RHD XV	RHD XV	2021-04-22
EPI_ISL_5800834	RHD XV	RHD XV	2021-04-22
EPI_ISL_5800835	RHD XV	RHD XV	2021-04-21
EPI_ISL_5800836	RHD XV	RHD XV	2021-04-22
EPI_ISL_5800837	RHD XV	RHD XV	2021-04-21
EPI_ISL_5800838	RHD XV	RHD XV	2021-04-22
EPI_ISL_5800839	RHD XV	RHD XV	2021-04-22
EPI_ISL_5800841	RHD XV	RHD XV	2021-04-22
EPI_ISL_5800842	RHD XV	RHD XV	2021-04-22
EPI_ISL_5800844	RHD XV	RHD XV	2021-04-21
EPI_ISL_5800847	RHD XV	RHD XV	2021-04-20
EPI_ISL_5800849	RHD XV	RHD XV	2021-04-21
EPI_ISL_5800850	RHD XV	RHD XV	2021-04-22
EPI_ISL_5800853	RHD XV	RHD XV	2021-04-22
EPI_ISL_5801938	RHD XV	RHD XV	2021-03-17
EPI_ISL_5801939	RHD XV	RHD XV	2021-03-17
EPI_ISL_5801940	RHD XV	RHD XV	2021-03-17
EPI_ISL_5801941	RHD XV	RHD XV	2021-03-17
EPI_ISL_5801942	RHD XV	RHD XV	2021-03-18
EPI_ISL_5801943	RHD XV	RHD XV	2021-03-18
EPI_ISL_5801944	RHD XV	RHD XV	2021-03-18
EPI_ISL_5802040	RHD XV	RHD XV	2021-03-23
EPI_ISL_5802149	RHD XV	RHD XV	2021-03-30
EPI_ISL_5802150	RHD XV	RHD XV	2021-03-30
EPI_ISL_5802154	RHD XV	RHD XV	2021-03-30
EPI_ISL_5802160	RHD XV	RHD XV	2021-03-30
EPI_ISL_5802162	RHD XV	RHD XV	2021-03-30
EPI_ISL_5802165	Southeast	RHD XV	2021-03-30
EPI_ISL_5802166	RHD XV	RHD XV	2021-03-30
EPI_ISL_5802167	Northeast	RHD XV	2021-03-31
EPI_ISL_5802169	RHD XV	RHD XV	2021-03-30
EPI_ISL_5802170	RHD XV	RHD XV	2021-03-30
EPI_ISL_5802171	RHD XV	RHD XV	2021-03-30
EPI_ISL_5802172	RHD XV	RHD XV	2021-03-31
EPI_ISL_5802173	RHD XV	RHD XV	2021-03-31
EPI_ISL_5802174	Northeast	RHD XV	2021-03-30
EPI_ISL_5802175	RHD XV	RHD XV	2021-03-30
EPI_ISL_3761618	SJdRP	SJdRP	2021-08-10
EPI_ISL_3761529	Northeast	SJdRP	2021-08-10
EPI_ISL_3761619	SJdRP	SJdRP	2021-08-10
EPI_ISL_3761620	SJdRP	SJdRP	2021-08-10
EPI_ISL_3761559	RHD XV	SJdRP	2021-08-10
EPI_ISL_3761621	SJdRP	SJdRP	2021-08-10
EPI_ISL_3761566	SJdRP	SJdRP	2021-08-10
EPI_ISL_3761534	SJdRP	SJdRP	2021-08-10
EPI_ISL_3761622	SJdRP	SJdRP	2021-08-10
EPI_ISL_3761623	SJdRP	SJdRP	2021-08-10
EPI_ISL_3761635	SJdRP	SJdRP	2021-08-10
EPI_ISL_3761765	RHD XV	RHD XV	2021-08-10
EPI_ISL_3761538	RHD XV	RHD XV	2021-08-10
EPI_ISL_3761527	RHD XV	RHD XV	2021-08-10
EPI_ISL_3761766	RHD XV	RHD XV	2021-08-10
EPI_ISL_3761541	RHD XV	RHD XV	2021-08-10
EPI_ISL_3761591	RHD XV	RHD XV	2021-08-09
EPI_ISL_3761549	RHD XV	RHD XV	2021-08-09
EPI_ISL_3761550	SJdRP	RHD XV	2021-08-09
EPI_ISL_3761592	RHD XV	RHD XV	2021-08-09
EPI_ISL_3761593	RHD XV	RHD XV	2021-08-09
EPI_ISL_3761594	RHD XV	RHD XV	2021-08-09
EPI_ISL_3761595	South	RHD XV	2021-08-09
EPI_ISL_3761533	RHD XV	SJdRP	2021-08-11
EPI_ISL_18620411	SJdRP	SJdRP	2021-09-01

EPI_ISL_18620412	SJdRP	SJdRP	2021-09-05
EPI_ISL_18620413	SJdRP	SJdRP	2021-09-08
EPI_ISL_5254491	RHD XV	RHD XV	2021-09-19
EPI_ISL_5254492	SJdRP	SJdRP	2021-09-20
EPI_ISL_5254493	SJdRP	SJdRP	2021-09-20
EPI_ISL_5254494	SJdRP	SJdRP	2021-09-20
EPI_ISL_5254495	SJdRP	SJdRP	2021-09-20
EPI_ISL_5254496	SJdRP	SJdRP	2021-09-20
EPI_ISL_5254497	SJdRP	SJdRP	2021-09-20
EPI_ISL_5254498	SJdRP	SJdRP	2021-09-20
EPI_ISL_5254499	SJdRP	SJdRP	2021-09-20
EPI_ISL_5254500	SJdRP	SJdRP	2021-09-20
EPI_ISL_5254501	SJdRP	SJdRP	2021-09-20
EPI_ISL_5254502	SJdRP	SJdRP	2021-09-20
EPI_ISL_5254503	SJdRP	SJdRP	2021-09-20
EPI_ISL_5254504	SJdRP	SJdRP	2021-09-20
EPI_ISL_5254559	RHD XV	RHD XV	2021-09-20
EPI_ISL_5254564	RHD XV	RHD XV	2021-09-20
EPI_ISL_5254505	RHD XV	RHD XV	2021-09-20
EPI_ISL_5254506	SJdRP	SJdRP	2021-09-22
EPI_ISL_5254507	SJdRP	SJdRP	2021-09-22
EPI_ISL_5254508	SJdRP	SJdRP	2021-09-22
EPI_ISL_5254509	SJdRP	SJdRP	2021-09-22
EPI_ISL_5254510	SJdRP	SJdRP	2021-09-22
EPI_ISL_5254511	SJdRP	SJdRP	2021-09-22
EPI_ISL_5254512	SJdRP	SJdRP	2021-09-22
EPI_ISL_5254513	SJdRP	SJdRP	2021-09-22
EPI_ISL_5254514	SJdRP	SJdRP	2021-09-22
EPI_ISL_5254515	SJdRP	SJdRP	2021-09-22
EPI_ISL_5254516	SJdRP	SJdRP	2021-09-22
EPI_ISL_5254517	SJdRP	SJdRP	2021-09-22
EPI_ISL_5254518	SJdRP	SJdRP	2021-09-22
EPI_ISL_5254519	SJdRP	SJdRP	2021-09-22
EPI_ISL_5254520	SJdRP	SJdRP	2021-09-22
EPI_ISL_5254521	SJdRP	SJdRP	2021-09-22
EPI_ISL_5254522	SJdRP	SJdRP	2021-09-22
EPI_ISL_5254523	SJdRP	SJdRP	2021-09-22
EPI_ISL_5254524	SJdRP	SJdRP	2021-09-22
EPI_ISL_5254525	Southeast	SJdRP	2021-09-22
EPI_ISL_5254565	SJdRP	SJdRP	2021-09-22
EPI_ISL_5254526	SJdRP	SJdRP	2021-09-22
EPI_ISL_5254527	SJdRP	SJdRP	2021-09-22
EPI_ISL_5254528	RHD XV	RHD XV	2021-09-22
EPI_ISL_5254561	RHD XV	RHD XV	2021-09-22
EPI_ISL_5254563	RHD XV	RHD XV	2021-09-22
EPI_ISL_5254562	RHD XV	RHD XV	2021-09-22
EPI_ISL_5254529	SJdRP	SJdRP	2021-09-29
EPI_ISL_5254530	SJdRP	SJdRP	2021-09-29
EPI_ISL_5254531	North	SJdRP	2021-09-29
EPI_ISL_5254532	SJdRP	SJdRP	2021-09-29
EPI_ISL_5254533	SJdRP	SJdRP	2021-09-29
EPI_ISL_5254534	Northeast	SJdRP	2021-09-29
EPI_ISL_5254535	SJdRP	SJdRP	2021-09-29
EPI_ISL_5254536	SJdRP	SJdRP	2021-09-29
EPI_ISL_5254537	SJdRP	SJdRP	2021-09-29
EPI_ISL_5254538	SJdRP	SJdRP	2021-09-29
EPI_ISL_5254539	SJdRP	SJdRP	2021-09-29
EPI_ISL_5254540	SJdRP	SJdRP	2021-09-29
EPI_ISL_5254541	SJdRP	SJdRP	2021-09-29
EPI_ISL_5254542	SJdRP	SJdRP	2021-09-29
EPI_ISL_5254543	SJdRP	SJdRP	2021-09-29
EPI_ISL_5254544	SJdRP	SJdRP	2021-09-29
EPI_ISL_5254545	SJdRP	SJdRP	2021-09-29

EPI_ISL_5254546	SJdRP	SJdRP	2021-09-29
EPI_ISL_5254547	SJdRP	SJdRP	2021-09-29
EPI_ISL_5254548	SJdRP	SJdRP	2021-09-29
EPI_ISL_5254560	RHD XV	RHD XV	2021-09-29
EPI_ISL_5254549	RHD XV	RHD XV	2021-09-29
EPI_ISL_5254550	South	RHD XV	2021-09-29
EPI_ISL_5254551	RHD XV	RHD XV	2021-09-29
EPI_ISL_5254552	RHD XV	RHD XV	2021-09-29
EPI_ISL_5254553	SJdRP	RHD XV	2021-09-29
EPI_ISL_5254554	RHD XV	RHD XV	2021-09-29
EPI_ISL_18620414	RHD XV	SJdRP	2021-10-26
EPI_ISL_18620415	SJdRP	SJdRP	2021-10-27
EPI_ISL_18620416	SJdRP	SJdRP	2021-10-31
EPI_ISL_18620417	SJdRP	SJdRP	2021-10-31
EPI_ISL_18620396	RHD XV	RHD XV	2021-10-29
EPI_ISL_18620397	RHD XV	RHD XV	2021-11-01
EPI_ISL_18620398	RHD XV	RHD XV	2021-11-02
EPI_ISL_18620399	RHD XV	RHD XV	2021-11-02
EPI_ISL_18620400	RHD XV	RHD XV	2021-11-02
EPI_ISL_18620418	SJdRP	SJdRP	2021-10-29
EPI_ISL_18620419	SJdRP	SJdRP	2021-10-29
EPI_ISL_18620420	SJdRP	SJdRP	2021-10-29
EPI_ISL_18620421	SJdRP	SJdRP	2021-10-29
EPI_ISL_18620422	SJdRP	SJdRP	2021-10-29
EPI_ISL_18620423	SJdRP	SJdRP	2021-11-01
EPI_ISL_18620424	SJdRP	SJdRP	2021-11-01
EPI_ISL_18620425	SJdRP	SJdRP	2021-11-01
EPI_ISL_18620426	RHD XV	SJdRP	2021-11-02
EPI_ISL_18620427	SJdRP	SJdRP	2021-11-08
EPI_ISL_18620428	SJdRP	SJdRP	2021-11-16
EPI_ISL_18620429	SJdRP	SJdRP	2021-11-26
EPI_ISL_18620430	SJdRP	SJdRP	2021-11-26
EPI_ISL_18620431	SJdRP	SJdRP	2021-11-26
EPI_ISL_18620432	RHD XV	SJdRP	2021-11-26
EPI_ISL_18620433	SJdRP	SJdRP	2021-11-25
EPI_ISL_18620434	North	SJdRP	2021-11-26
EPI_ISL_18620435	SJdRP	SJdRP	2021-11-30
EPI_ISL_18620503	RHD XV	RHD XV	2021-12-09
EPI_ISL_18620402	RHD XV	RHD XV	2021-12-10
EPI_ISL_18620403	SJdRP	RHD XV	2021-12-12
EPI_ISL_18620404	RHD XV	RHD XV	2021-12-13
EPI_ISL_18620438	SJdRP	SJdRP	2021-12-17
EPI_ISL_18620439	North	SJdRP	2021-12-17
EPI_ISL_18620440	SJdRP	SJdRP	2021-12-18
EPI_ISL_18620441	SJdRP	SJdRP	2021-12-20
EPI_ISL_18620443	SJdRP	SJdRP	2021-12-20
EPI_ISL_18620444	RHD XV	SJdRP	2021-12-21
EPI_ISL_18620445	SJdRP	SJdRP	2021-12-21
EPI_ISL_18620446	RHD XV	SJdRP	2021-12-21
EPI_ISL_18620447	SJdRP	SJdRP	2021-12-21
EPI_ISL_18620504	RHD XV	RHD XV	2021-12-29
EPI_ISL_18620401	RHD XV	RHD XV	2022-01-03
EPI_ISL_18620436	SJdRP	SJdRP	2022-01-02
EPI_ISL_18620500	RHD XV	RHD XV	2022-01-11
EPI_ISL_18620501	SJdRP	RHD XV	2022-01-11
EPI_ISL_18620502	SJdRP	RHD XV	2022-01-11
EPI_ISL_18620448	SJdRP	SJdRP	2022-01-11
EPI_ISL_18620405	RHD XV	RHD XV	2022-02-03
EPI_ISL_3761624	SJdRP	SJdRP	2021-08-11
EPI_ISL_3761625	SJdRP	SJdRP	2021-08-11
EPI_ISL_3761626	South	SJdRP	2021-08-11
EPI_ISL_3761627	RHD XV	SJdRP	2021-08-11
EPI_ISL_3761628	SJdRP	SJdRP	2021-08-11

EPI_ISL_3761596	SJdRP	RHD XV	2021-08-11
EPI_ISL_3761629	RHD XV	SJdRP	2021-08-11
EPI_ISL_3761630	SJdRP	SJdRP	2021-08-11
EPI_ISL_3761574	RHD XV	RHD XV	2021-08-11
EPI_ISL_3761631	RHD XV	SJdRP	2021-08-11
EPI_ISL_3761632	SJdRP	SJdRP	2021-08-11
EPI_ISL_3761604	SJdRP	RHD XV	2021-08-11
EPI_ISL_3761633	RHD XV	SJdRP	2021-08-11
EPI_ISL_3761606	RHD XV	RHD XV	2021-08-11
EPI_ISL_3761634	RHD XV	SJdRP	2021-08-11
EPI_ISL_5254558	SJdRP	SJdRP	2021-09-20
EPI_ISL_5254555	North	SJdRP	2021-09-20
EPI_ISL_5254556	SJdRP	SJdRP	2021-09-20
EPI_ISL_5254557	SJdRP	SJdRP	2021-09-20
EPI_ISL_18620449	SJdRP	SJdRP	2021-12-15
EPI_ISL_18620409	Northeast	RHD XV	2021-12-15
EPI_ISL_18620450	SJdRP	SJdRP	2021-12-20
EPI_ISL_18620451	SJdRP	SJdRP	2021-12-20
EPI_ISL_18620452	SJdRP	SJdRP	2021-12-20
EPI_ISL_18620410	SJdRP	RHD XV	2021-12-20
EPI_ISL_18620453	SJdRP	SJdRP	2021-12-20
EPI_ISL_18620454	SJdRP	SJdRP	2021-12-22
EPI_ISL_18620455	SJdRP	SJdRP	2021-12-22
EPI_ISL_18620456	SJdRP	SJdRP	2021-12-22
EPI_ISL_18620494	SJdRP	RHD XV	2022-01-03
EPI_ISL_18620457	RHD XV	SJdRP	2022-01-03
EPI_ISL_18620458	SJdRP	SJdRP	2022-01-03
EPI_ISL_18620506	SJdRP	RHD XV	2022-01-03
EPI_ISL_18620459	SJdRP	SJdRP	2022-01-03
EPI_ISL_18620460	SJdRP	SJdRP	2022-01-03
EPI_ISL_18620461	SJdRP	SJdRP	2022-01-03
EPI_ISL_18620462	SJdRP	SJdRP	2022-01-03
EPI_ISL_18620495	RHD XV	RHD XV	2022-01-03
EPI_ISL_18620463	North	SJdRP	2022-01-03
EPI_ISL_18620464	SJdRP	SJdRP	2022-01-03
EPI_ISL_18620465	SJdRP	SJdRP	2022-01-03
EPI_ISL_18620493	RHD XV	RHD XV	2022-01-03
EPI_ISL_18620466	SJdRP	SJdRP	2022-01-03
EPI_ISL_18620467	SJdRP	SJdRP	2022-01-03
EPI_ISL_18620496	RHD XV	RHD XV	2022-01-03
EPI_ISL_18620505	RHD XV	RHD XV	2022-01-03
EPI_ISL_18620468	SJdRP	SJdRP	2022-01-03
EPI_ISL_18620469	SJdRP	SJdRP	2022-01-03
EPI_ISL_18620470	SJdRP	SJdRP	2022-01-03
EPI_ISL_18620471	SJdRP	SJdRP	2022-01-03
EPI_ISL_18620472	SJdRP	SJdRP	2022-01-05
EPI_ISL_18620473	SJdRP	SJdRP	2022-01-05
EPI_ISL_18620474	SJdRP	SJdRP	2022-01-05
EPI_ISL_18620475	SJdRP	SJdRP	2022-01-05
EPI_ISL_18620476	SJdRP	SJdRP	2022-01-05
EPI_ISL_18620498	SJdRP	RHD XV	2022-01-05
EPI_ISL_18620499	RHD XV	RHD XV	2022-01-05
EPI_ISL_18620477	SJdRP	SJdRP	2022-01-05
EPI_ISL_18620478	SJdRP	SJdRP	2022-01-05
EPI_ISL_18620479	SJdRP	SJdRP	2022-01-05
EPI_ISL_18620480	SJdRP	SJdRP	2022-01-05
EPI_ISL_18620497	SJdRP	RHD XV	2022-01-05
EPI_ISL_18620481	SJdRP	SJdRP	2022-01-05
EPI_ISL_18620482	RHD XV	SJdRP	2022-01-05
EPI_ISL_18620483	SJdRP	SJdRP	2022-01-05
EPI_ISL_18620484	SJdRP	SJdRP	2022-01-05
EPI_ISL_18620485	SJdRP	SJdRP	2022-01-05
EPI_ISL_18620486	SJdRP	SJdRP	2022-01-05

EPI_ISL_18620487	SJdRP	SJdRP	2022-01-05
EPI_ISL_18620488	SJdRP	SJdRP	2022-01-05
EPI_ISL_18620489	SJdRP	SJdRP	2022-01-05
EPI_ISL_18620490	SJdRP	SJdRP	2022-01-05
EPI_ISL_18620491	SJdRP	SJdRP	2022-01-05
EPI_ISL_18620492	SJdRP	SJdRP	2022-01-05
EPI_ISL_18620406	SJdRP	RHD XV	2022-01-19
EPI_ISL_18620407	RHD XV	RHD XV	2022-01-19
EPI_ISL_1785611	SJdRP	SJdRP	2021-01-16
EPI_ISL_2155088	SJdRP	RHD XV	2021-01-16
EPI_ISL_2544840	South	SJdRP	2021-01-16
EPI_ISL_2008906	SJdRP	SJdRP	2021-04-27
EPI_ISL_2008907	SJdRP	SJdRP	2021-04-27
EPI_ISL_2008908	SJdRP	SJdRP	2021-04-27
EPI_ISL_2008909	SJdRP	SJdRP	2021-04-27
EPI_ISL_2008910	SJdRP	SJdRP	2021-04-27
EPI_ISL_2008911	RHD XV	RHD XV	2021-04-27
EPI_ISL_2008912	RHD XV	SJdRP	2021-04-27
EPI_ISL_2008913	SJdRP	SJdRP	2021-04-27
EPI_ISL_2008914	RHD XV	RHD XV	2021-04-27
EPI_ISL_2008915	RHD XV	SJdRP	2021-04-27
EPI_ISL_2008916	SJdRP	SJdRP	2021-04-27
EPI_ISL_2008917	SJdRP	SJdRP	2021-04-27
EPI_ISL_2008918	SJdRP	SJdRP	2021-04-27
EPI_ISL_2008919	SJdRP	SJdRP	2021-04-27
EPI_ISL_2008920	SJdRP	SJdRP	2021-04-27
EPI_ISL_2008921	SJdRP	SJdRP	2021-04-27
EPI_ISL_2008922	SJdRP	SJdRP	2021-04-27
EPI_ISL_2008923	SJdRP	SJdRP	2021-04-27
EPI_ISL_2008924	RHD XV	SJdRP	2021-04-27
EPI_ISL_2008925	RHD XV	SJdRP	2021-04-27
EPI_ISL_2008926	SJdRP	SJdRP	2021-04-27
EPI_ISL_2008927	SJdRP	SJdRP	2021-04-27
EPI_ISL_2008928	SJdRP	SJdRP	2021-04-27
EPI_ISL_2107292	RHD XV	RHD XV	2021-04-27
EPI_ISL_2008929	SJdRP	SJdRP	2021-04-27
EPI_ISL_2107293	SJdRP	SJdRP	2021-04-27
EPI_ISL_2107294	SJdRP	SJdRP	2021-04-27
EPI_ISL_2008930	SJdRP	SJdRP	2021-04-27
EPI_ISL_2008931	SJdRP	SJdRP	2021-04-27
EPI_ISL_2107301	SJdRP	SJdRP	2021-04-27
EPI_ISL_2107296	SJdRP	SJdRP	2021-04-27
EPI_ISL_2008932	SJdRP	SJdRP	2021-04-27
EPI_ISL_2107298	RHD XV	SJdRP	2021-04-27
EPI_ISL_2107295	SJdRP	SJdRP	2021-04-27
EPI_ISL_2008933	SJdRP	SJdRP	2021-04-27
EPI_ISL_2008934	RHD XV	RHD XV	2021-04-27
EPI_ISL_2107297	SJdRP	SJdRP	2021-04-27
EPI_ISL_2008935	RHD XV	RHD XV	2021-04-27
EPI_ISL_2544856	SJdRP	RHD XV	2021-01-17
EPI_ISL_1754186	RHD XV	SJdRP	2021-03-11
EPI_ISL_1941583	Midwest	SJdRP	2021-02-26
EPI_ISL_2544857	RHD XV	RHD XV	2021-01-18
EPI_ISL_2544858	RHD XV	RHD XV	2021-01-18
EPI_ISL_2543759	SJdRP	SJdRP	2021-01-16
EPI_ISL_1785613	South	SJdRP	2021-01-16
EPI_ISL_1785612	SJdRP	SJdRP	2021-03-08
EPI_ISL_2013854	SJdRP	SJdRP	2021-01-16
EPI_ISL_2544859	RHD XV	RHD XV	2021-01-26
EPI_ISL_2544860	RHD XV	RHD XV	2021-01-26
EPI_ISL_2544861	Southeast	SJdRP	2021-01-26
EPI_ISL_2544862	RHD XV	RHD XV	2021-01-26
EPI_ISL_2544837	Northeast	SJdRP	2021-01-16

EPI_ISL_2544841	Northeast	SJdRP	2021-01-16
EPI_ISL_2544863	RHD XV	SJdRP	2021-01-26
EPI_ISL_2544864	North	SJdRP	2021-01-26
EPI_ISL_2544865	South	SJdRP	2021-01-26
EPI_ISL_2544866	Northeast	SJdRP	2021-01-26
EPI_ISL_2544867	RHD XV	SJdRP	2021-01-26
EPI_ISL_2544868	RHD XV	SJdRP	2021-01-26
EPI_ISL_2155377	South	RHD XV	2021-01-16
EPI_ISL_2544869	SJdRP	SJdRP	2021-01-26
EPI_ISL_2544870	Northeast	SJdRP	2021-01-26
EPI_ISL_2544871	RHD XV	RHD XV	2021-01-26
EPI_ISL_2544872	SJdRP	SJdRP	2021-01-26
EPI_ISL_2544873	South	RHD XV	2021-01-26
EPI_ISL_2544842	Southeast	RHD XV	2021-01-16
EPI_ISL_2544874	RHD XV	RHD XV	2021-01-25
EPI_ISL_2155841	RHD XV	RHD XV	2021-01-16
EPI_ISL_2544875	RHD XV	RHD XV	2021-01-25
EPI_ISL_2013855	South	SJdRP	2021-01-16
EPI_ISL_2544876	RHD XV	RHD XV	2021-01-26
EPI_ISL_2156051	Southeast	RHD XV	2021-01-16
EPI_ISL_2544843	Southeast	SJdRP	2021-01-16
EPI_ISL_2544844	South	SJdRP	2021-01-16
EPI_ISL_2544845	South	SJdRP	2021-01-16
EPI_ISL_2008940	SJdRP	SJdRP	2021-03-24
EPI_ISL_2154703	SJdRP	SJdRP	2021-03-25
EPI_ISL_2154956	SJdRP	SJdRP	2021-03-25
EPI_ISL_2156096	Northeast	SJdRP	2021-01-16
EPI_ISL_2544846	Northeast	SJdRP	2021-01-16
EPI_ISL_2544847	SJdRP	SJdRP	2021-01-16
EPI_ISL_2544848	SJdRP	SJdRP	2021-01-16
EPI_ISL_2544849	Northeast	SJdRP	2021-01-16
EPI_ISL_2544850	South	SJdRP	2021-01-16
EPI_ISL_2156152	SJdRP	SJdRP	2021-01-16
EPI_ISL_2544838	SJdRP	SJdRP	2021-01-16
EPI_ISL_2156261	SJdRP	SJdRP	2021-01-16
EPI_ISL_2544851	Northeast	SJdRP	2021-01-16
EPI_ISL_2544852	Northeast	SJdRP	2021-01-16
EPI_ISL_2544853	Northeast	SJdRP	2021-01-16
EPI_ISL_1785614	South	SJdRP	2021-01-16
EPI_ISL_3048954	RHD XV	SJdRP	2021-04-15
EPI_ISL_3048955	SJdRP	SJdRP	2021-04-14
EPI_ISL_3048956	RHD XV	SJdRP	2021-04-19
EPI_ISL_3048957	RHD XV	SJdRP	2021-04-19
EPI_ISL_3761525	SJdRP	SJdRP	2021-04-19
EPI_ISL_3048958	SJdRP	SJdRP	2021-04-19
EPI_ISL_2008944	RHD XV	SJdRP	2021-04-19
EPI_ISL_2008945	SJdRP	SJdRP	2021-04-19
EPI_ISL_2008946	SJdRP	SJdRP	2021-04-19
EPI_ISL_2008947	RHD XV	RHD XV	2021-04-19
EPI_ISL_2008948	SJdRP	SJdRP	2021-04-19
EPI_ISL_2008949	RHD XV	RHD XV	2021-04-19
EPI_ISL_3761749	SJdRP	SJdRP	2021-04-20
EPI_ISL_2008950	SJdRP	SJdRP	2021-04-19
EPI_ISL_2008951	SJdRP	SJdRP	2021-04-19
EPI_ISL_2107303	SJdRP	SJdRP	2021-04-19
EPI_ISL_2008952	SJdRP	SJdRP	2021-04-19
EPI_ISL_2008953	RHD XV	RHD XV	2021-04-19
EPI_ISL_2008954	SJdRP	SJdRP	2021-04-19
EPI_ISL_2008955	RHD XV	SJdRP	2021-04-19
EPI_ISL_2008956	SJdRP	SJdRP	2021-04-19
EPI_ISL_2008957	RHD XV	SJdRP	2021-04-19
EPI_ISL_2107305	Northeast	RHD XV	2021-04-19
EPI_ISL_2008958	RHD XV	RHD XV	2021-04-19

EPI_ISL_2008959	Southeast	SJdRP	2021-04-19
EPI_ISL_2008960	North	SJdRP	2021-04-19
EPI_ISL_2008961	RHD XV	SJdRP	2021-04-19
EPI_ISL_2107302	RHD XV	RHD XV	2021-04-19
EPI_ISL_2008962	RHD XV	SJdRP	2021-04-19
EPI_ISL_2008963	SJdRP	SJdRP	2021-04-19
EPI_ISL_2008964	RHD XV	SJdRP	2021-04-19
EPI_ISL_2008965	SJdRP	SJdRP	2021-04-19
EPI_ISL_2008966	RHD XV	SJdRP	2021-04-19
EPI_ISL_2008967	SJdRP	SJdRP	2021-04-19
EPI_ISL_3048959	SJdRP	SJdRP	2021-04-26
EPI_ISL_3048960	SJdRP	SJdRP	2021-04-26
EPI_ISL_3761750	SJdRP	SJdRP	2021-03-05
EPI_ISL_3048961	SJdRP	SJdRP	2021-05-03
EPI_ISL_3761763	RHD XV	RHD XV	2021-05-03
EPI_ISL_3761751	SJdRP	SJdRP	2021-03-05
EPI_ISL_3048962	SJdRP	SJdRP	2021-05-03
EPI_ISL_3048963	SJdRP	SJdRP	2021-05-04
EPI_ISL_3048964	SJdRP	SJdRP	2021-05-05
EPI_ISL_3048965	SJdRP	SJdRP	2021-05-05
EPI_ISL_3048966	RHD XV	SJdRP	2021-05-06
EPI_ISL_3761752	SJdRP	SJdRP	2021-05-06
EPI_ISL_3048967	RHD XV	SJdRP	2021-05-06
EPI_ISL_3048968	North	SJdRP	2021-05-10
EPI_ISL_3048969	RHD XV	SJdRP	2021-05-10
EPI_ISL_3048970	SJdRP	SJdRP	2021-05-10
EPI_ISL_3048971	SJdRP	SJdRP	2021-05-10
EPI_ISL_3761613	RHD XV	RHD XV	2021-05-10
EPI_ISL_3761616	RHD XV	RHD XV	2021-05-12
EPI_ISL_3048972	RHD XV	SJdRP	2021-05-13
EPI_ISL_3048973	RHD XV	SJdRP	2021-05-17
EPI_ISL_3048974	SJdRP	SJdRP	2021-05-17
EPI_ISL_3048975	Southeast	SJdRP	2021-05-17
EPI_ISL_3048976	RHD XV	SJdRP	2021-05-18
EPI_ISL_3048977	SJdRP	SJdRP	2021-05-19
EPI_ISL_3048978	SJdRP	SJdRP	2021-05-20
EPI_ISL_3761570	SJdRP	SJdRP	2021-05-20
EPI_ISL_3048979	SJdRP	SJdRP	2021-05-24
EPI_ISL_3048980	SJdRP	SJdRP	2021-05-25
EPI_ISL_3761636	SJdRP	SJdRP	2021-05-06
EPI_ISL_3761576	RHD XV	RHD XV	2021-05-11
EPI_ISL_3761637	RHD XV	SJdRP	2021-05-25
EPI_ISL_3761617	SJdRP	RHD XV	2021-05-26
EPI_ISL_3761638	SJdRP	SJdRP	2021-05-26
EPI_ISL_3761639	RHD XV	SJdRP	2021-05-26
EPI_ISL_3761640	SJdRP	SJdRP	2021-05-26
EPI_ISL_3761641	RHD XV	SJdRP	2021-05-26
EPI_ISL_3761642	SJdRP	SJdRP	2021-05-26
EPI_ISL_3761643	RHD XV	SJdRP	2021-05-27
EPI_ISL_3761644	RHD XV	SJdRP	2021-05-27
EPI_ISL_3048981	RHD XV	SJdRP	2021-05-31
EPI_ISL_3048982	SJdRP	SJdRP	2021-05-31
EPI_ISL_3048983	RHD XV	RHD XV	2021-05-31
EPI_ISL_3048984	North	SJdRP	2021-05-31
EPI_ISL_3048985	SJdRP	SJdRP	2021-05-31
EPI_ISL_3048986	Northeast	SJdRP	2021-05-31
EPI_ISL_3048987	SJdRP	SJdRP	2021-05-31
EPI_ISL_3048988	RHD XV	SJdRP	2021-05-31
EPI_ISL_3048989	RHD XV	SJdRP	2021-05-31
EPI_ISL_3048990	SJdRP	SJdRP	2021-05-31
EPI_ISL_3048991	SJdRP	SJdRP	2021-05-31
EPI_ISL_3048992	SJdRP	SJdRP	2021-05-31
EPI_ISL_3048993	SJdRP	SJdRP	2021-05-31

EPI_ISL_3048994	SJdRP	SJdRP	2021-05-31
EPI_ISL_3048995	Northeast	SJdRP	2021-05-31
EPI_ISL_3048996	RHD XV	SJdRP	2021-05-31
EPI_ISL_3048997	SJdRP	SJdRP	2021-05-30
EPI_ISL_3761645	SJdRP	SJdRP	2021-05-19
EPI_ISL_3048998	RHD XV	SJdRP	2021-06-09
EPI_ISL_3048999	SJdRP	SJdRP	2021-06-09
EPI_ISL_3049000	SJdRP	SJdRP	2021-06-09
EPI_ISL_3761646	SJdRP	SJdRP	2021-05-15
EPI_ISL_3055544	SJdRP	SJdRP	2021-06-14
EPI_ISL_3049002	SJdRP	SJdRP	2021-06-15
EPI_ISL_3049003	North	SJdRP	2021-06-15
EPI_ISL_3049004	RHD XV	SJdRP	2021-06-15
EPI_ISL_3049005	SJdRP	SJdRP	2021-06-15
EPI_ISL_3049006	RHD XV	SJdRP	2021-06-16
EPI_ISL_3049007	RHD XV	SJdRP	2021-06-16
EPI_ISL_3761753	SJdRP	SJdRP	2021-06-16
EPI_ISL_3049008	SJdRP	SJdRP	2021-06-16
EPI_ISL_3055542	RHD XV	SJdRP	2021-06-16
EPI_ISL_3049009	SJdRP	SJdRP	2021-06-16
EPI_ISL_3049010	RHD XV	SJdRP	2021-06-16
EPI_ISL_3049011	SJdRP	SJdRP	2021-06-17
EPI_ISL_3049012	SJdRP	SJdRP	2021-06-17
EPI_ISL_3049013	RHD XV	SJdRP	2021-06-21
EPI_ISL_3049014	SJdRP	SJdRP	2021-06-21
EPI_ISL_3049015	SJdRP	SJdRP	2021-06-21
EPI_ISL_3049016	SJdRP	SJdRP	2021-06-21
EPI_ISL_3049017	RHD XV	SJdRP	2021-06-21
EPI_ISL_3049018	SJdRP	SJdRP	2021-06-21
EPI_ISL_3049019	SJdRP	SJdRP	2021-06-21
EPI_ISL_3049020	SJdRP	SJdRP	2021-06-21
EPI_ISL_3761760	SJdRP	SJdRP	2021-06
EPI_ISL_3761647	SJdRP	SJdRP	2021-06-21
EPI_ISL_3055529	SJdRP	SJdRP	2021-06-21
EPI_ISL_3761648	SJdRP	SJdRP	2021-06-21
EPI_ISL_3761649	RHD XV	SJdRP	2021-06-21
EPI_ISL_3761650	SJdRP	SJdRP	2021-06-22
EPI_ISL_3761651	SJdRP	SJdRP	2021-06-22
EPI_ISL_3761652	SJdRP	SJdRP	2021-06-22
EPI_ISL_3761653	RHD XV	SJdRP	2021-06-22
EPI_ISL_3761654	SJdRP	SJdRP	2021-06-22
EPI_ISL_3761655	RHD XV	SJdRP	2021-06-22
EPI_ISL_3761656	SJdRP	SJdRP	2021-06-22
EPI_ISL_3761587	SJdRP	RHD XV	2021-06-23
EPI_ISL_3761657	SJdRP	SJdRP	2021-06-22
EPI_ISL_3761658	RHD XV	SJdRP	2021-06-23
EPI_ISL_3761659	SJdRP	SJdRP	2021-06-23
EPI_ISL_3761660	SJdRP	SJdRP	2021-06-23
EPI_ISL_3761661	SJdRP	SJdRP	2021-06-23
EPI_ISL_3761662	SJdRP	SJdRP	2021-06-23
EPI_ISL_3761663	RHD XV	SJdRP	2021-06-23
EPI_ISL_3761599	RHD XV	RHD XV	2021-06-23
EPI_ISL_3761664	SJdRP	SJdRP	2021-06-23
EPI_ISL_3761535	SJdRP	SJdRP	2021-06-23
EPI_ISL_3761665	SJdRP	SJdRP	2021-06-24
EPI_ISL_3761666	SJdRP	SJdRP	2021-06-24
EPI_ISL_3761667	RHD XV	SJdRP	2021-06-24
EPI_ISL_3761668	SJdRP	SJdRP	2021-06-24
EPI_ISL_3761669	SJdRP	SJdRP	2021-06-24
EPI_ISL_3761670	Northeast	SJdRP	2021-06-24
EPI_ISL_3761671	SJdRP	SJdRP	2021-06-24
EPI_ISL_3761672	RHD XV	SJdRP	2021-06-24
EPI_ISL_3761673	SJdRP	SJdRP	2021-06-24

EPI_ISL_3761761	SJdRP	SJdRP	2021-06-12
EPI_ISL_3761754	RHD XV	SJdRP	2021-06-28
EPI_ISL_3049021	RHD XV	RHD XV	2021-06-29
EPI_ISL_3049023	SJdRP	SJdRP	2021-06-29
EPI_ISL_3049024	SJdRP	SJdRP	2021-06-29
EPI_ISL_3049025	SJdRP	SJdRP	2021-06-29
EPI_ISL_3049026	SJdRP	SJdRP	2021-06-29
EPI_ISL_3049027	RHD XV	SJdRP	2021-06-29
EPI_ISL_3049028	RHD XV	SJdRP	2021-06-29
EPI_ISL_3049029	Southeast	RHD XV	2021-06-29
EPI_ISL_3055532	SJdRP	SJdRP	2021-06-29
EPI_ISL_3055533	Northeast	SJdRP	2021-06-29
EPI_ISL_3049030	SJdRP	SJdRP	2021-06-29
EPI_ISL_3761755	SJdRP	SJdRP	2021-06-29
EPI_ISL_3761586	RHD XV	RHD XV	2021-06-29
EPI_ISL_3761756	RHD XV	SJdRP	2021-06-29
EPI_ISL_3761523	SJdRP	SJdRP	2021-06-30
EPI_ISL_3761757	SJdRP	SJdRP	2021-06-30
EPI_ISL_3761758	SJdRP	SJdRP	2021-06-30
EPI_ISL_3761528	RHD XV	SJdRP	2021-06-30
EPI_ISL_3761759	RHD XV	SJdRP	2021-06-30
EPI_ISL_3761563	RHD XV	RHD XV	2021-07-01
EPI_ISL_3761522	RHD XV	RHD XV	2021-07-01
EPI_ISL_3761674	SJdRP	SJdRP	2021-07-01
EPI_ISL_3761588	SJdRP	RHD XV	2021-07-01
EPI_ISL_3761675	SJdRP	SJdRP	2021-07-01
EPI_ISL_3761558	RHD XV	SJdRP	2021-07-01
EPI_ISL_3761676	SJdRP	SJdRP	2021-07-01
EPI_ISL_3761539	RHD XV	RHD XV	2021-07-01
EPI_ISL_3761677	SJdRP	SJdRP	2021-07-01
EPI_ISL_3761678	SJdRP	SJdRP	2021-07-01
EPI_ISL_3761679	RHD XV	SJdRP	2021-07-01
EPI_ISL_3761680	SJdRP	SJdRP	2021-07-01
EPI_ISL_3761573	RHD XV	SJdRP	2021-07-01
EPI_ISL_3761681	RHD XV	SJdRP	2021-07-05
EPI_ISL_3761682	SJdRP	SJdRP	2021-07-05
EPI_ISL_3761683	RHD XV	SJdRP	2021-07-05
EPI_ISL_3761530	SJdRP	SJdRP	2021-07-05
EPI_ISL_3761684	SJdRP	SJdRP	2021-07-05
EPI_ISL_3761685	SJdRP	SJdRP	2021-07-05
EPI_ISL_3761686	SJdRP	SJdRP	2021-07-05
EPI_ISL_3761614	RHD XV	RHD XV	2021-07-05
EPI_ISL_3761687	RHD XV	SJdRP	2021-07-05
EPI_ISL_3761688	Northeast	SJdRP	2021-07-06
EPI_ISL_3761689	RHD XV	SJdRP	2021-07-06
EPI_ISL_3761690	Southeast	SJdRP	2021-07-06
EPI_ISL_3761691	SJdRP	SJdRP	2021-07-06
EPI_ISL_3761605	RHD XV	RHD XV	2021-07-06
EPI_ISL_3761589	RHD XV	RHD XV	2021-07-07
EPI_ISL_3761537	RHD XV	RHD XV	2021-07-07
EPI_ISL_3761597	RHD XV	RHD XV	2021-07-07
EPI_ISL_3761692	RHD XV	SJdRP	2021-07-07
EPI_ISL_3761693	RHD XV	SJdRP	2021-07-07
EPI_ISL_3761567	RHD XV	RHD XV	2021-07-07
EPI_ISL_3761694	RHD XV	SJdRP	2021-07-07
EPI_ISL_3761695	SJdRP	SJdRP	2021-07-07
EPI_ISL_3761546	SJdRP	SJdRP	2021-07-07
EPI_ISL_3761696	SJdRP	SJdRP	2021-07-07
EPI_ISL_3761697	SJdRP	SJdRP	2021-07-07
EPI_ISL_3761545	SJdRP	SJdRP	2021-07-07
EPI_ISL_3761561	RHD XV	RHD XV	2021-07-07
EPI_ISL_3761554	SJdRP	SJdRP	2021-07-07
EPI_ISL_3761547	RHD XV	RHD XV	2021-07-07

EPI_ISL_3761608	RHD XV	RHD XV	2021-07-07
EPI_ISL_3761698	RHD XV	SJdRP	2021-07-07
EPI_ISL_3761600	RHD XV	RHD XV	2021-07-07
EPI_ISL_3761569	RHD XV	SJdRP	2021-07-07
EPI_ISL_3761544	SJdRP	SJdRP	2021-07-07
EPI_ISL_3761556	SJdRP	SJdRP	2021-07-07
EPI_ISL_3761699	SJdRP	SJdRP	2021-07-07
EPI_ISL_3761565	SJdRP	SJdRP	2021-07-12
EPI_ISL_3761700	RHD XV	SJdRP	2021-07-12
EPI_ISL_3761609	SJdRP	RHD XV	2021-07-12
EPI_ISL_3761701	RHD XV	SJdRP	2021-07-12
EPI_ISL_3761702	RHD XV	SJdRP	2021-07-12
EPI_ISL_3761764	RHD XV	RHD XV	2021-07-12
EPI_ISL_3761703	SJdRP	SJdRP	2021-07-12
EPI_ISL_3761704	RHD XV	SJdRP	2021-07-12
EPI_ISL_3761572	RHD XV	SJdRP	2021-07-12
EPI_ISL_3761705	SJdRP	SJdRP	2021-07-12
EPI_ISL_3761706	SJdRP	SJdRP	2021-07-12
EPI_ISL_3761707	RHD XV	SJdRP	2021-07-12
EPI_ISL_3761708	RHD XV	SJdRP	2021-07-12
EPI_ISL_3761709	SJdRP	SJdRP	2021-07-12
EPI_ISL_3761555	RHD XV	SJdRP	2021-07-13
EPI_ISL_3761531	SJdRP	SJdRP	2021-07-13
EPI_ISL_3761536	SJdRP	SJdRP	2021-07-13
EPI_ISL_3761710	SJdRP	SJdRP	2021-07-13
EPI_ISL_3761551	SJdRP	SJdRP	2021-07-13
EPI_ISL_3761519	SJdRP	SJdRP	2021-07-13
EPI_ISL_3761524	Southeast	SJdRP	2021-07-13
EPI_ISL_3761568	RHD XV	SJdRP	2021-07-13
EPI_ISL_3761526	SJdRP	SJdRP	2021-07-13
EPI_ISL_3761711	SJdRP	SJdRP	2021-07-13
EPI_ISL_3761712	SJdRP	SJdRP	2021-07-13
EPI_ISL_3761713	SJdRP	SJdRP	2021-07-13
EPI_ISL_3761714	RHD XV	SJdRP	2021-07-14
EPI_ISL_3761715	SJdRP	SJdRP	2021-07-14
EPI_ISL_3761716	SJdRP	SJdRP	2021-07-14
EPI_ISL_3761610	RHD XV	RHD XV	2021-07-14
EPI_ISL_3761717	SJdRP	SJdRP	2021-07-14
EPI_ISL_3761601	RHD XV	RHD XV	2021-07-14
EPI_ISL_3761718	SJdRP	SJdRP	2021-07-14
EPI_ISL_3761719	SJdRP	SJdRP	2021-07-14
EPI_ISL_3761602	RHD XV	RHD XV	2021-07-14
EPI_ISL_3761532	North	SJdRP	2021-07-14
EPI_ISL_3761720	SJdRP	SJdRP	2021-07-14
EPI_ISL_3761615	RHD XV	RHD XV	2021-07-14
EPI_ISL_3761721	SJdRP	SJdRP	2021-07-14
EPI_ISL_3761564	SJdRP	SJdRP	2021-07-14
EPI_ISL_3761548	SJdRP	SJdRP	2021-07-14
EPI_ISL_3761722	SJdRP	SJdRP	2021-07-14
EPI_ISL_3761590	RHD XV	RHD XV	2021-07-14
EPI_ISL_3761723	SJdRP	SJdRP	2021-07-14
EPI_ISL_3761724	RHD XV	SJdRP	2021-07-15
EPI_ISL_3761725	SJdRP	SJdRP	2021-07-15
EPI_ISL_3761726	RHD XV	SJdRP	2021-07-15
EPI_ISL_3761603	SJdRP	RHD XV	2021-07-15
EPI_ISL_3761585	SJdRP	RHD XV	2021-07-15
EPI_ISL_3761727	SJdRP	SJdRP	2021-07-15
EPI_ISL_3761728	SJdRP	SJdRP	2021-07-15
EPI_ISL_3761729	SJdRP	SJdRP	2021-07-15
EPI_ISL_3761730	SJdRP	SJdRP	2021-07-15
EPI_ISL_3761731	SJdRP	SJdRP	2021-07-15
EPI_ISL_3761732	Southeast	SJdRP	2021-07-15
EPI_ISL_3761733	RHD XV	SJdRP	2021-07-15

EPI_ISL_3761518	SJdRP	SJdRP	2021-07-15
EPI_ISL_3761521	SJdRP	SJdRP	2021-07-19
EPI_ISL_3761540	SJdRP	SJdRP	2021-07-19
EPI_ISL_3761734	SJdRP	SJdRP	2021-07-19
EPI_ISL_3761611	RHD XV	RHD XV	2021-07-19
EPI_ISL_3761735	SJdRP	SJdRP	2021-07-19
EPI_ISL_3761736	SJdRP	SJdRP	2021-07-19
EPI_ISL_3761598	RHD XV	RHD XV	2021-07-19
EPI_ISL_3761762	Northeast	RHD XV	2021-07-19
EPI_ISL_3761737	SJdRP	SJdRP	2021-07-19
EPI_ISL_3761738	SJdRP	SJdRP	2021-07-19
EPI_ISL_3761739	SJdRP	SJdRP	2021-07-19
EPI_ISL_3761740	SJdRP	SJdRP	2021-07-19
EPI_ISL_3761741	SJdRP	SJdRP	2021-07-19
EPI_ISL_3761742	Southeast	SJdRP	2021-07-19
EPI_ISL_3761577	RHD XV	RHD XV	2021-07-20
EPI_ISL_3761571	RHD XV	SJdRP	2021-07-20
EPI_ISL_3761743	RHD XV	SJdRP	2021-07-20
EPI_ISL_3761744	RHD XV	SJdRP	2021-07-20
EPI_ISL_3761552	RHD XV	RHD XV	2021-07-20
EPI_ISL_3761562	RHD XV	SJdRP	2021-07-20
EPI_ISL_3761745	SJdRP	SJdRP	2021-07-20
EPI_ISL_3761612	RHD XV	RHD XV	2021-07-20
EPI_ISL_3761543	RHD XV	SJdRP	2021-07-20
EPI_ISL_3761560	SJdRP	SJdRP	2021-07-20
EPI_ISL_3761578	SJdRP	SJdRP	2021-07-20
EPI_ISL_3761557	RHD XV	SJdRP	2021-07-20
EPI_ISL_3761746	SJdRP	SJdRP	2021-07-20
EPI_ISL_3761542	SJdRP	SJdRP	2021-07-20
EPI_ISL_3761575	RHD XV	SJdRP	2021-07-20
EPI_ISL_3761747	RHD XV	SJdRP	2021-07-20
EPI_ISL_3761553	SJdRP	SJdRP	2021-07-20
EPI_ISL_3761748	SJdRP	SJdRP	2021-07-20
EPI_ISL_2544854	RHD XV	RHD XV	2021-01-14
EPI_ISL_2156471	SJdRP	RHD XV	2021-01-14
EPI_ISL_2544855	RHD XV	RHD XV	2021-01-15
EPI_ISL_1785609	Southeast	RHD XV	2021-01-16
EPI_ISL_2107304	Southeast	RHD XV	2021-01-16
EPI_ISL_2008939	Northeast	RHD XV	2021-01-18
EPI_ISL_1785610	SJdRP	SJdRP	2021-01-16
EPI_ISL_2544839	Northeast	SJdRP	2021-01-16
EPI_ISL_2008938	SJdRP	SJdRP	2021-02-27
EPI_ISL_1303509	North	North	2021-02-01
EPI_ISL_1358301	North	North	2021-02-22
EPI_ISL_1358303	South	North	2021-02-23
EPI_ISL_2756436	North	North	2021-03-11
EPI_ISL_2756449	North	North	2021-01-30
EPI_ISL_2756453	Northeast	North	2021-01-04
EPI_ISL_2756456	North	North	2021-02-15
EPI_ISL_2756471	North	North	2021-04-07
EPI_ISL_2919207	Southeast	North	2021-05-10
EPI_ISL_2919211	Northeast	North	2021-05-14
EPI_ISL_2919224	North	North	2021-04-15
EPI_ISL_2983418	North	North	2021-06-17
EPI_ISL_2983422	North	North	2021-06-16
EPI_ISL_3190411	SJdRP	North	2021-06-28
EPI_ISL_10322494	North	North	2022-01-07
EPI_ISL_3235314	RHD XV	North	2021-07-01
EPI_ISL_3235306	North	North	2021-07-03
EPI_ISL_4080803	North	North	2021-07-28
EPI_ISL_4080802	North	North	2021-07-31
EPI_ISL_8152492	North	North	2021-11-29
EPI_ISL_8430554	North	North	2021-09-21

EPI_ISL_8430557	Southeast	North	2021-09-23
EPI_ISL_8430560	Northeast	North	2021-09-27
EPI_ISL_8430606	North	North	2021-11-15
EPI_ISL_8430613	North	North	2021-11-23
EPI_ISL_12040004	North	North	2021-11-01
EPI_ISL_12040013	South	North	2021-11-03
EPI_ISL_12508303	RHD XV	North	2021-10-31
EPI_ISL_12690896	RHD XV	North	2022-03-16
EPI_ISL_15463294	North	North	2021-12-02
EPI_ISL_7661122	North	North	2021-10-14
EPI_ISL_7657567	North	North	2021-08-20
EPI_ISL_7661124	North	North	2021-11-11
EPI_ISL_7661119	Northeast	North	2021-10-13
EPI_ISL_7661120	North	North	2021-11-04
EPI_ISL_7359128	South	North	2021-08-03
EPI_ISL_7657568	North	North	2021-08-25
EPI_ISL_7249088	North	North	2021-07-07
EPI_ISL_7657560	North	North	2021-09-14
EPI_ISL_10083540	North	North	2021-12-14
EPI_ISL_7494055	RHD XV	RHD XV	2021-09-28
EPI_ISL_7494054	RHD XV	RHD XV	2021-09-30
EPI_ISL_7494134	RHD XV	RHD XV	2021-09-28
EPI_ISL_7494052	RHD XV	RHD XV	2021-09-29
EPI_ISL_7494130	RHD XV	RHD XV	2021-09-30
EPI_ISL_7494151	RHD XV	RHD XV	2021-09-29
EPI_ISL_5020917	RHD XV	RHD XV	2021-09-13
EPI_ISL_5020930	RHD XV	RHD XV	2021-09-13
EPI_ISL_5020945	RHD XV	RHD XV	2021-09-13
EPI_ISL_5020952	Northeast	RHD XV	2021-09-13
EPI_ISL_5020965	RHD XV	RHD XV	2021-09-13
EPI_ISL_5020970	RHD XV	RHD XV	2021-09-13
EPI_ISL_5020990	RHD XV	RHD XV	2021-09-13
EPI_ISL_5020994	RHD XV	RHD XV	2021-09-13
EPI_ISL_5020996	RHD XV	RHD XV	2021-09-13
EPI_ISL_5020998	RHD XV	RHD XV	2021-09-13
EPI_ISL_5021002	RHD XV	RHD XV	2021-09-13
EPI_ISL_5021069	RHD XV	RHD XV	2021-09-14
EPI_ISL_5021071	RHD XV	RHD XV	2021-09-14
EPI_ISL_5021073	RHD XV	RHD XV	2021-09-14
EPI_ISL_5021081	RHD XV	RHD XV	2021-09-13
EPI_ISL_5021094	RHD XV	RHD XV	2021-09-13
EPI_ISL_5021222	North	RHD XV	2021-09-13
EPI_ISL_5021248	Northeast	RHD XV	2021-09-14
EPI_ISL_5021504	SJdRP	SJdRP	2021-09-13
EPI_ISL_5021505	SJdRP	SJdRP	2021-09-13
EPI_ISL_5021511	SJdRP	SJdRP	2021-09-13
EPI_ISL_5021517	SJdRP	SJdRP	2021-09-13
EPI_ISL_5021521	SJdRP	SJdRP	2021-09-13
EPI_ISL_5021522	South	SJdRP	2021-09-13
EPI_ISL_5021530	SJdRP	SJdRP	2021-09-13
EPI_ISL_5021535	SJdRP	SJdRP	2021-09-13
EPI_ISL_5021539	SJdRP	SJdRP	2021-09-13
EPI_ISL_5021540	SJdRP	SJdRP	2021-09-13
EPI_ISL_5021543	SJdRP	SJdRP	2021-09-13
EPI_ISL_5021548	SJdRP	SJdRP	2021-09-13
EPI_ISL_5021549	SJdRP	SJdRP	2021-09-13
EPI_ISL_5021556	SJdRP	SJdRP	2021-09-14
EPI_ISL_5021564	RHD XV	SJdRP	2021-09-11
EPI_ISL_5021574	RHD XV	RHD XV	2021-09-12
EPI_ISL_5021575	RHD XV	RHD XV	2021-09-12
EPI_ISL_5021579	RHD XV	RHD XV	2021-09-13
EPI_ISL_5021582	RHD XV	RHD XV	2021-09-13
EPI_ISL_5021583	RHD XV	RHD XV	2021-09-13

EPI_ISL_5049591	SJdRP	RHD XV	2021-09-13
EPI_ISL_5049592	RHD XV	RHD XV	2021-09-14
EPI_ISL_5049593	RHD XV	RHD XV	2021-09-13
EPI_ISL_5049594	SJdRP	SJdRP	2021-09-14
EPI_ISL_5049595	Southeast	RHD XV	2021-09-14
EPI_ISL_5049596	SJdRP	SJdRP	2021-09-15
EPI_ISL_5049597	Northeast	SJdRP	2021-09-14
EPI_ISL_5049598	SJdRP	SJdRP	2021-09-14
EPI_ISL_5049599	SJdRP	SJdRP	2021-09-14
EPI_ISL_5049600	SJdRP	SJdRP	2021-09-14
EPI_ISL_5049601	SJdRP	SJdRP	2021-09-14
EPI_ISL_5049602	SJdRP	SJdRP	2021-09-15
EPI_ISL_5049603	SJdRP	SJdRP	2021-09-15
EPI_ISL_5049604	SJdRP	SJdRP	2021-09-15
EPI_ISL_5049605	SJdRP	SJdRP	2021-09-15
EPI_ISL_5049606	SJdRP	SJdRP	2021-09-15
EPI_ISL_5049607	SJdRP	SJdRP	2021-09-15
EPI_ISL_5049608	SJdRP	SJdRP	2021-09-15
EPI_ISL_5049609	SJdRP	SJdRP	2021-09-15
EPI_ISL_5049610	SJdRP	SJdRP	2021-09-15
EPI_ISL_5049611	SJdRP	SJdRP	2021-09-15
EPI_ISL_5049612	SJdRP	SJdRP	2021-09-16
EPI_ISL_5049613	SJdRP	SJdRP	2021-09-16
EPI_ISL_5049614	North	SJdRP	2021-09-16
EPI_ISL_5049618	SJdRP	SJdRP	2021-09-16
EPI_ISL_5049619	SJdRP	SJdRP	2021-09-15
EPI_ISL_5049620	RHD XV	SJdRP	2021-09-15
EPI_ISL_5049621	SJdRP	SJdRP	2021-09-15
EPI_ISL_5049622	SJdRP	SJdRP	2021-09-15
EPI_ISL_5049623	RHD XV	RHD XV	2021-09-15
EPI_ISL_5049624	RHD XV	RHD XV	2021-09-15
